# Discrimination of psychrophilic enzymes using machine learning algorithms with amino acid composition descriptor

**DOI:** 10.3389/fmicb.2023.1130594

**Published:** 2023-02-13

**Authors:** Ailan Huang, Fuping Lu, Fufeng Liu

**Affiliations:** ^1^College of Biotechnology, Tianjin University of Science & Technology, Tianjin, China; ^2^Key Laboratory of Industrial Fermentation Microbiology, Ministry of Education, Tianjin Key Laboratory of Industrial Microbiology, Tianjin, China

**Keywords:** psychrophilic enzyme, machine learning, support vector machine, amino acid composition, structural flexibility

## Abstract

**Introduction:**

Psychrophilic enzymes are a class of macromolecules with high catalytic activity at low temperatures. Cold-active enzymes possessing eco-friendly and cost-effective properties, are of huge potential application in detergent, textiles, environmental remediation, pharmaceutical as well as food industry. Compared with the time-consuming and labor-intensive experiments, computational modeling especially the machine learning (ML) algorithm is a high-throughput screening tool to identify psychrophilic enzymes efficiently.

**Methods:**

In this study, the influence of 4 ML methods (support vector machines, K-nearest neighbor, random forest, and naïve Bayes), and three descriptors, i.e., amino acid composition (AAC), dipeptide combinations (DPC), and AAC + DPC on the model performance were systematically analyzed.

**Results and discussion:**

Among the 4 ML methods, the support vector machine model based on the AAC descriptor using 5-fold cross-validation achieved the best prediction accuracy with 80.6%. The AAC outperformed than the DPC and AAC + DPC descriptors regardless of the ML methods used. In addition, amino acid frequencies between psychrophilic and non-psychrophilic proteins revealed that higher frequencies of Ala, Gly, Ser, and Thr, and lower frequencies of Glu, Lys, Arg, Ile,Val, and Leu could be related to the protein psychrophilicity. Further, ternary models were also developed that could classify psychrophilic, mesophilic, and thermophilic proteins effectively. The predictive accuracy of the ternary classification model using AAC descriptor *via* the support vector machine algorithm was 75.8%. These findings would enhance our insight into the cold-adaption mechanisms of psychrophilic proteins and aid in the design of engineered cold-active enzymes. Moreover, the proposed model could be used as a screening tool to identify novel cold-adapted proteins.

## Introduction

Psychrophilic enzymes are also called cold-adaptive enzymes, maintaining catalytic efficiency and function under low temperatures (0–25°C; Siddiqui and Cavicchioli, 2006; Sarmiento et al., 2015). This types of enzymes are mainly isolated from glaciers, polar regions, and deep seas. Possessing high catalytic activity at low and moderate temperatures and heat-labile properties, psychrophilic enzymes could be used in various industries such as detergent, food, medical, and bioremediation (Saeki et al., 2007; Al-Ghanayem and Joseph, 2020; Gupta et al., 2020; Mangiagalli et al., 2020; Kumar et al., 2021; Mhetras et al., 2021), thus they offer huge economic benefits. For example, the addition of cold-adapted proteases, lipases, and cellulases in detergents can remove dirt efficiently under low temperatures, which is eco-friendly and cost-effective as does not require an extensive heating process. Cold-active lipases additives can prevent spoilage and adverse changes of substrates that are used in food processing. The application of cold-adapted lipases in the synthesis of chiral organic compounds has also been reported in several reviews (Mhetras et al., 2021). Additionally, psychrophilic enzymes are not only vital enzymes in industrial applications, but also are valuable research models in the basic research of protein folding and catalysis (Feller and Gerday, 2003; Siddiqui and Cavicchioli, 2006; Åqvist et al., 2017).

According to the Arrhenius equation $K=Ae\frac{-E_{a}}{RT}$ , the reaction rate decays exponentially with the decrease of temperatures (Struvay and Feller, 2012; Åqvist et al., 2017). The main issue of psychrophilic enzymes is how to maintain the catalytic rate at low temperatures. The first resolved psychrophilic protein structure is alpha-amylase derived from *Alteromonas haloplanctis* (Aghajari et al., 1996). The increasing resolved 3D structures of psychrophilic enzymes shed light on the molecular basis of cold-adaption mechanisms (Arnorsdottir et al., 2005). The comparison with the mesophilic and thermophilic homologous proteins shows that psychrophilic enzymes have evolved some structural features responsible to maintain the low-temperature catalytic activity, such as more flexible structures, decreased core hydrophobicity, increased surface hydrophobicity, fewer disulfide bonds (Schrøder Leiros et al., 2000), and reduced hydrogen bonds (Schrøder Leiros et al., 2000; Aghajari et al., 2003; Siddiqui and Cavicchioli, 2006; Almog et al., 2009). Comparative structural analysis showed that different family enzymes adopt one or a combination of several structural features to adapt to low-temperatures (Struvay and Feller, 2012; Tribelli and López, 2018).

Unlike wet experiments that are time-consuming and costly, *in silico* method is a reliable and powerful tool. Machine learning (ML) is a data-driven technology and has been applied to various fields, such as protein structure prediction (Senior et al., 2020; Jumper et al., 2021), protein engineering (Saito et al., 2018; Wang et al., 2018; Mazurenko et al., 2019; Wu et al., 2019; Yang et al., 2019), protein function prediction (Han et al., 2006; Bonetta and Valentino, 2020; Zhang Y. H. et al., 2021), enzyme substrate scope prediction (Mou et al., 2021), screening of novel pharmaceutical candidates (Chandak et al., 2020) and efficient catalysts (Niu et al., 2021). Computational methods have been conducted to classify acidic and alkaline enzymes effectively based on the protein sequence (Zhang et al., 2009; Khan et al., 2015). Similarly, predictive models have also been developed to discriminate thermophilic proteins from mesophilic proteins (Gromiha and Suresh, 2008; Lin and Chen, 2011; Ai et al., 2018; Feng et al., 2020; Guo et al., 2020; Wang et al., 2020; Ahmed et al., 2022). These models that are composed of different descriptors based on protein sequences achieved reliable prediction performance. Many comparative analyzes have shown that different types of amino acids have a tendency among mesophilic and thermophilic proteins, and amino acid composition (AAC) descriptor could discriminate mesophilic and thermophilic proteins using the support vector machines (SVM), *K*-nearest neighbor (KNN), random forest (RF), and naïve Bayes (Bayes) algorithms. In addition, other sequence descriptors such as dipeptide combinations (DPC) were also utilized to establish the predictive model.

Due to the essential role of psychrophilic enzymes in industrial applications and scientific research, many efforts have also been carried out to investigate cold-adapted enzymes. A previous study has shown that the random forest model using AAC descriptor and hydrophobic residue patterns as input features could discriminate psychrophilic from mesophilic proteins, with an accuracy of 70.3% (Nath et al., 2012). To achieve the interpretability of the model, a cascade model was also proposed, and the percentage of different amino acid composition ranges was used as input features, in which the attribute with the highest discriminability was the serine, lysine, glutamic acid and alanine amino acid composition. The rotation forest reached the highest accuracy with 70.5% (Nath and Subbiah, 2014). Although these models achieved good accuracy, there are also several issues needed to be addressed. On the one hand, the influence of different features on predictive accuracies should be investigated. Though the AAC descriptor alone proved to be a very useful feature for discriminating psychrophilic and mesophilic proteins, the DPC descriptor has not been explored. On the other hand, the feasibility of the ternary classification model (psychrophilic-mesophilic-thermophilic) is also worth exploring.

In this concern, the iLearnPlus software was exploited to develop computational model, where feature extraction, feature selection, model construction, and result visualization were all deployed in the software (Chen et al., 2021). Considering the ability of the AAC descriptor to identify psychrophilic and mesophilic proteins, the AAC descriptor was utilized in this study, while the DPC descriptor was also tested and the ability of AAC, DPC, and AAC + DPC to distinguish psychrophilic from non-psychrophilic proteins was compared. The results indicated that the binary and ternary classification model could be used for discriminating psychrophilic from mesophilic and thermophilic enzymes. In addition, the accuracies of different models were studied and AAC frequency distributions among psychrophilic and non-psychrophilic proteins were also explored.

## Materials and methods

### Datasets preparation

The thermophilic and mesophilic proteins were obtained from (Lin and Chen, 2011). The psychrophilic proteins were extracted from the UniProt web server, the search keywords including the “psychrophilic, cold-adaptive, and low-temperature.” Firstly, all queried protein sequences must be reviewed and manually annotated; secondly, entries which be a part of other proteins were excluded; finally, to avoid redundancy and homology bias, the CD-HIT program (Huang et al., 2010) was used with a cutoff of 40% sequence identity. The dataset included 2,400 protein sequences, among which the thermophilic, mesophilic, and psychrophilic proteins were 915, 793 and 692, respectively. The training and test sets were split in a 4:1 ratio, so there were 731, 574, and 554 thermophilic, mesophilic, and psychrophilic proteins in the training set, and 184, 219, and 138 in the test set. The sequences of the datasets could be downloaded from the supporting material.

### Feature extraction

Protein feature descriptors are generated from protein sequences. The feature descriptor extraction and model construction were implemented using iLearnPlus, a machine-learning platform that served as protein sequence analysis and prediction. It has been reported that the AAC and DPC descriptors can discriminate the thermophilic from mesophilic proteins effectively (Zhang and Fang, 2006b; Gromiha and Suresh, 2008; Lin and Chen, 2011; Ai et al., 2018; Guo et al., 2020; Wang et al., 2020), therefore, the two descriptors were calculated for each protein sequence.

AAC refers to the occurrence of each amino acid in the protein sequence (Wang et al., 2011; Khan et al., 2015; Guo et al., 2020; Sun et al., 2020; Charoenkwan et al., 2021), as that there are 20 kinds of naturally-occurring amino acids, that is ACDEFGHIKLMNPQRSTVWY. Therefore, each residue frequency in a sequence can be calculated by the following formula:


$$Comp\left(i\right)=\frac{n\left(i\right)}{n},i\in A,C,D,E,F,\ldots W,Y$$

DPC calculates dipeptide composition and generates a 400-dimensional feature vector.(Charoenkwan et al., 2021) and it was defined as:


$$Comp\left(i,j\right)=\frac{n\left(i,j\right)}{n-1},i,j\in A,C,D,E,F,\ldots W,Y$$

The CHI2 algorithm (Chen et al., 2009) was used for DPC feature selection and dimensional reduction.

### Model construction

Several machine learning algorithms were tested to distinguish between psychrophilic and non-psychrophilic proteins. Considering the reliable performance of SVM, RF, KNN, and Bayes in classifying thermophilic and mesophilic proteins, these algorithms were used in our study (Cortes and Vapnik, 1995; Breiman, 2001). The RF is an ensemble of decision trees. The algorithm performs better than decision trees by building and merging multiple decision trees to obtain more accurate results. For a new sample, the RF assigns the class label based on the prediction by each tree. The n_trees was set to 300. The SVM is a simple but powerful supervised machine learning algorithm used in classification and/or regression. It seeks a hyperplane to classify samples. When the sample is linearly inseparable in the low-dimensional space, the kernel function is used to map the sample to the high-dimensional space to achieve linear separability. The radial basis function (RBF) was selected in the kernel function of SVM and the optimized γ and C were 8.0 and 15.0, respectively. KNN is also one of the most basic algorithms in supervised machine learning. It assumes that similar things are near to each other, and the Euclidean distance between samples was calculated to solve the classification and regression of data. The top K value was set to 3. Naïve Bayes method is a set of supervised machine learning algorithms based on Bayes’ Theorem. It obeys the assumption that every pair of the feature are independent and every feature is equal to the value of the class variable. It states the following relationship, and is mathematically expressed as the following equation:


$$P\left(A|B\right)=\frac{P\left(B|A\right)P\left(A\right)}{P\left(B\right)}$$

where A and B are events and P(B) ≠ 0. All the parameters in four machine learning algorithms were optimized by grid search.

### Performance evaluation

The data set was randomly divided into training set and test set in a ratio of 4:1. The 5-fold cross-validation was also used in this study, out of which the datasets were randomly divided into 5 subsets, one of which was used to test the model, and the remaining 4 subsets were used as the training set to train the model and optimize the parameters. This process was repeated 5 times until each subset was used as the test set only once to validate the model. Four indicators were adopted to evaluate the model performance, that is sensitivity (Sn), specificity (Sp), accuracy (ACC), and Matthews correlation coefficient (MCC). The calculation formulas of these indicators were as follows:

**Table:** 

$\mathrm{Sensitivity}=\frac{\mathrm{TP}}{\mathrm{TP}+\mathrm{FN}}$
$\mathrm{Specificity}=\frac{\mathrm{TN}}{\mathrm{TN}+\mathrm{FP}}$
$\mathrm{Accuracy}=\frac{\mathrm{TP}+\mathrm{TN}}{\mathrm{TP}+\mathrm{TN}+\mathrm{FP}+\mathrm{FN}}$
$\mathrm{MCC}=\frac{\left(\mathrm{TP}\times \mathrm{TN}\right)-\left(\mathrm{FP}\times \mathrm{FN}\right)}{\sqrt{\left(\mathrm{TP}+\mathrm{FP}\right)\left(\mathrm{TP}+\mathrm{FN}\right)\left(\mathrm{TN}+\mathrm{FP}\right)\left(\mathrm{TN}+\mathrm{FN}\right)}}$

Where TP, TN, FP, and FN represent the number of correctly predicted positive samples, correctly predicted negative samples, incorrectly predicted positive samples, and incorrectly predicted negative samples, respectively. For a multi-classification task, the ACC was calculated as follows:


$$ACC=\frac{TP\left(i\right)+TN\left(i\right)}{TP\left(i\right)+TN\left(i\right)+FP\left(i\right)+FN{\left(i\right)}^{\prime }}$$

Where TP(i), TN(i), FP(i), and FN(i) represent the number of the samples that are correctly predicted as i-th class, the number of samples that are classified correctly as not to be i-th class, the number of samples not in i-th class that is classified wrongly as belonging to i-th class, the number of samples in i-th class that are predicted incorrectly as not in i-th class, respectively. Additionally, ROC (Receiver Operating Characteristic) curves were also utilized to visualize the predictive performance of the classifiers.

## Results and discussion

### Performance of models for discriminating psychrophilic and non-psychrophilic proteins

The predictive performance of the machine learning model based on AAC, DPC, and the combination of the two descriptors were listed in Table 1. Among the models using with AAC descriptor, the SVM model achieved the highest prediction accuracy with 80.6%. The prediction accuracy of RF was lower than 0.4% of the SVM. And the two other models, Bayes and KNN, the accuracies were less than 80%, especially the Bayes model had the lowest prediction accuracy with 73.8%. All the trained models were public in github^1^website.

**Table 1:** Prediction results of AAC and DPC descriptors for psychrophilic and non-psychrophilic proteins.

Descriptor	Model	Sn	Sp	Acc	MCC
AAC	RF	0.524	0.919	0.802	0.497
	SVM	0.780	0.859	0.806	0.546
	Bayes	0.711	0.749	0.738	0.439
	KNN	0.667	0.808	0.766	0.470
DPC	RF	0.266	0.940	0.740	0.300
	SVM	0.548	0.874	0.747	0.370
	Bayes	0.654	0.696	0.684	0.348
	KNN	0.461	0.823	0.716	0.304
AAC + DPC	RF	0.529	0.943	0.790	0.497
	SVM	0.785	0.850	0.801	0.546
	Bayes	0.743	0.702	0.714	0.439
	KNN	0.629	0.817	0.761	0.470

Sn: sensitivity, Sp: specificity, Acc: Accuracy, MCC: Matthews correlation coefficient.

DPC descriptor generates 400-dimensional vectors, and the CHI2 algorithm was used for feature dimension reduction. The results indicated that the prediction accuracy of the models based on DPC descriptor decreased compared with AAC descriptor, which declined by about 5–7%. Similar to the AAC descriptor, the model with DPC descriptor using the SVM algorithm also achieved the best accuracies.

In addition, the two descriptors were integrated to construct the classification model. Compared with the DPC descriptor, the accuracies of the AAC + DPC descriptors had been improved to varying degrees. While compared to AAC descriptors, the accuracies of SVM and KNN models were almost unchanged, RF and Bayes models even dropped by 1.2 and 2.4%, respectively. The models constructed by AAC have achieved best accuracy *via* four machine learning algorithms in this study. Of course, DPC is also an important feature to distinguish psychrophilic proteins from non-psychrophilic proteins, which has also achieved relatively good prediction accuracy. However, the addition of DPC to the descriptor may cause redundancy of features, which makes the accuracy decrease slightly. In a report of using AAC and DPC to distinguish thermophilic and mesophilic proteins, AAC and DPC achieved 0.9256 and 0.9157 prediction accuracy, respectively. The accuracy of AAC and DPC combination to distinguish thermophilic and mesophilic proteins also decreased, though DPC contained more parameters (Lin and Chen, 2011).

**Figure 1:**
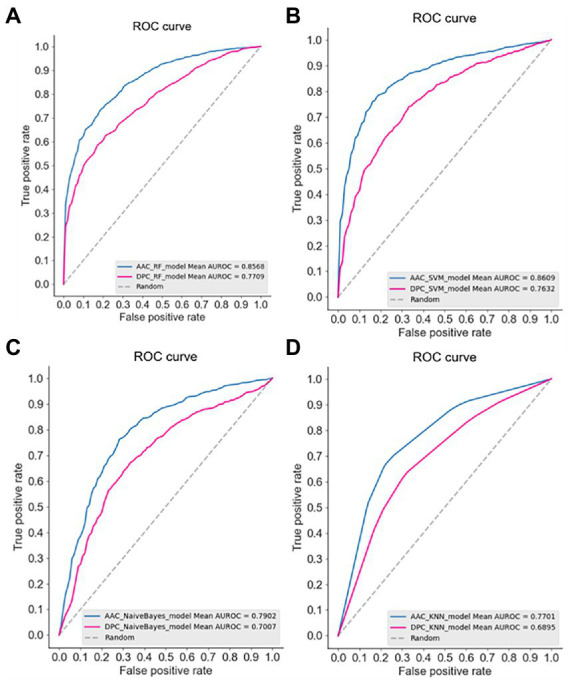
The receiver operation characteristic (ROC) curve of the four machine learning models using AAC and DPC descriptors. **(A)** Random forest, RF; **(B)** SVM; **(C)** Bayes; **(D)** KNN.

The ROC curves of four models using AAC and DPC descriptors were plotted (Figure 1), it also showed that the AAC descriptors outperformed the DPC descriptors. In a comparison of the frequencies of amino acids between thermophilic and non-thermophilic proteins, it is proposed that the AAC captures the thermostability of the protein (Sun et al., 2020). Same as thermostability, it is also demonstrated that the psychrophilicity is highly related to the AAC descriptor in this study.

In addition to the higher predictive performance of the AAC descriptor, it is easy to find in Figure 1 that the SVM model achieved the best predictive accuracy among the four models (AUC 0.8609). It has been shown in many studies that the SVM model based on AAC descriptor had achieved good predictive performance in discriminating thermophilic from mesophilic enzymes. For example, the SVM model constructed by Michael Gromiha et al. using AAC descriptors could distinguish thermophiles from mesophiles with an accuracy of 89% (Gromiha and Suresh, 2008). And employing AAC descriptors with only 16 dimensions to discriminate thermophilic and non-thermophilic proteins with 93% accuracy (Guo et al., 2020).

### Performance of ternary classification for discriminating psychrophilic, mesophilic, and thermophilic proteins

**Figure 2:**
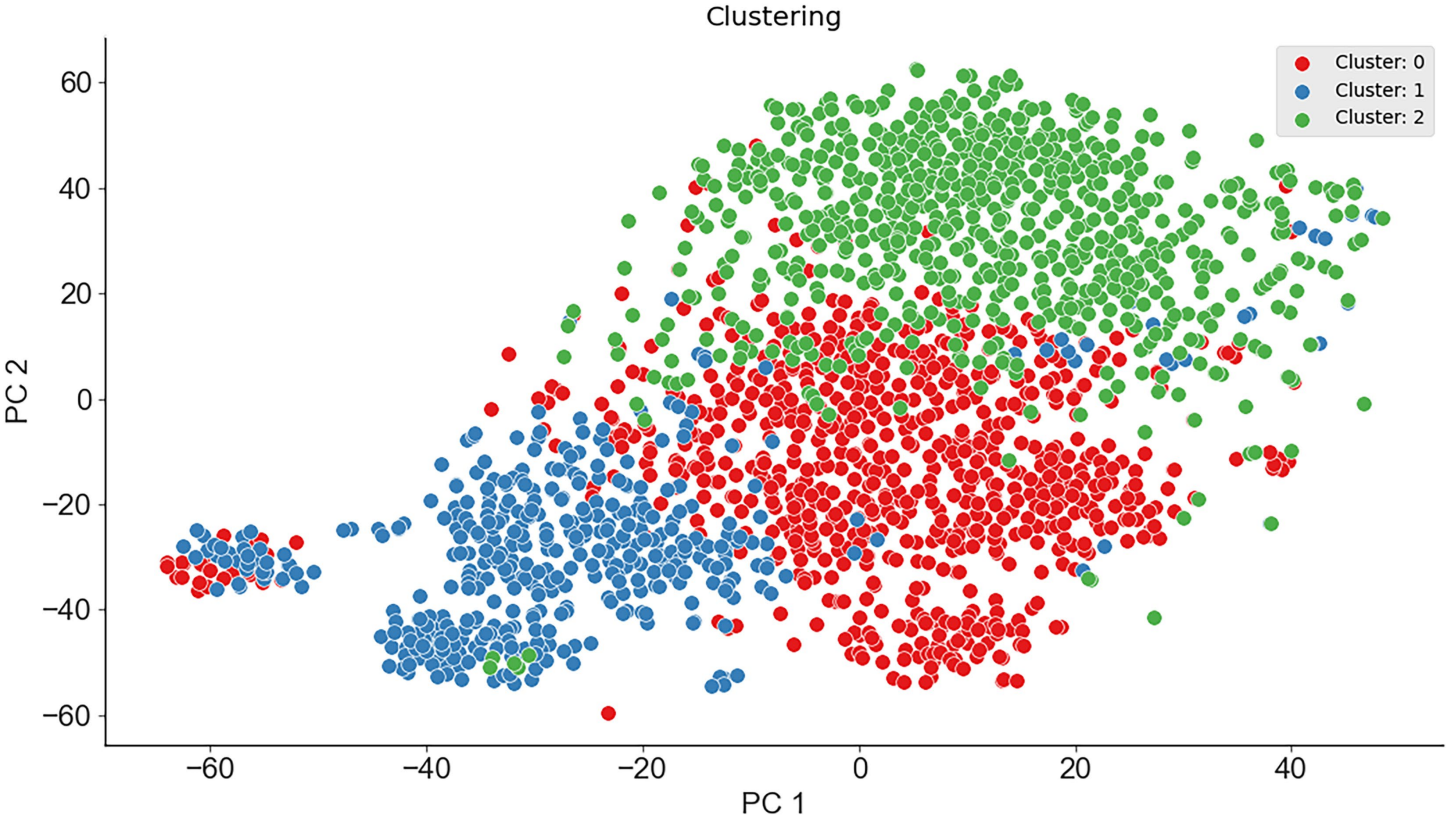
The scatter plot of the dimension reduction of the three enzymes.

To verify the feasibility of ternary classification, the scatter diagram of the three types of enzymes was calculated employing the K-means clustering method, where psychrophilic proteins were labeled as 1, mesophilic proteins were labeled as 0, and thermophilic proteins were labeled as 2. As seen from Figure 2, three types of proteins had different distribution patterns on principal component 1 and principal component 2, which indicated that multi-class classification is feasible. Therefore, the ternary classification model was established, and the predictive accuracies of the models for psychrophilic (P), mesophilic (M), and thermophilic (T) proteins were listed in Table 2; Supplementary Table S1.

**Table 2:** Prediction accuracies of ternary classification model for psychrophilic, mesophilic, and thermophilic proteins.

Class	Descriptor	RF	SVM	Bayes	KNN
P-M-T (P)^a^	AAC	0.738 (0.731)	0.758 (0.761)	0.756 (0.717)	0.746 (0.724)
	DPC	0.700 (0.710)	0.721 (0.703)	0.702 (0.703)	0.671 (0.688)
	AAC + DPC	0.736 (0.717)	0.761 (0.753)	0.716 (0.710)	0.688 (0.710)

a The combined accuracies with three descriptors for psychrophilic (P), mesophilic (M) and thermophilic (T) proteins, and the accuracies for psychrophilic proteins is listed in bracket.

The results showed that the accuracies of ternary classification were slightly lower than that of binary classification. The model predictive accuracy of AAC + DPC descriptors by SVM method was 76.1%, which was 4.0% lower than that of binary classification model with the same descriptors and method. In general, the SVM model performed well in discriminating three types of enzymes. As an ensemble classifier, the RF also achieved relatively good prediction accuracy with 73.8% solely using AAC descriptor. Among the four models, the predictive accuracy of the KNN model was relatively lower than other models, the prediction accuracy based on DPC descriptor was 67.1%. Taken together, the AAC descriptor achieved the highest prediction accuracy, which indicated the capacity of the amino acid composition in distinguishing psychrophilic proteins.

### Differences of amino acid composition in psychrophilic, mesophilic, and thermophilic proteins

**Figure 3:**
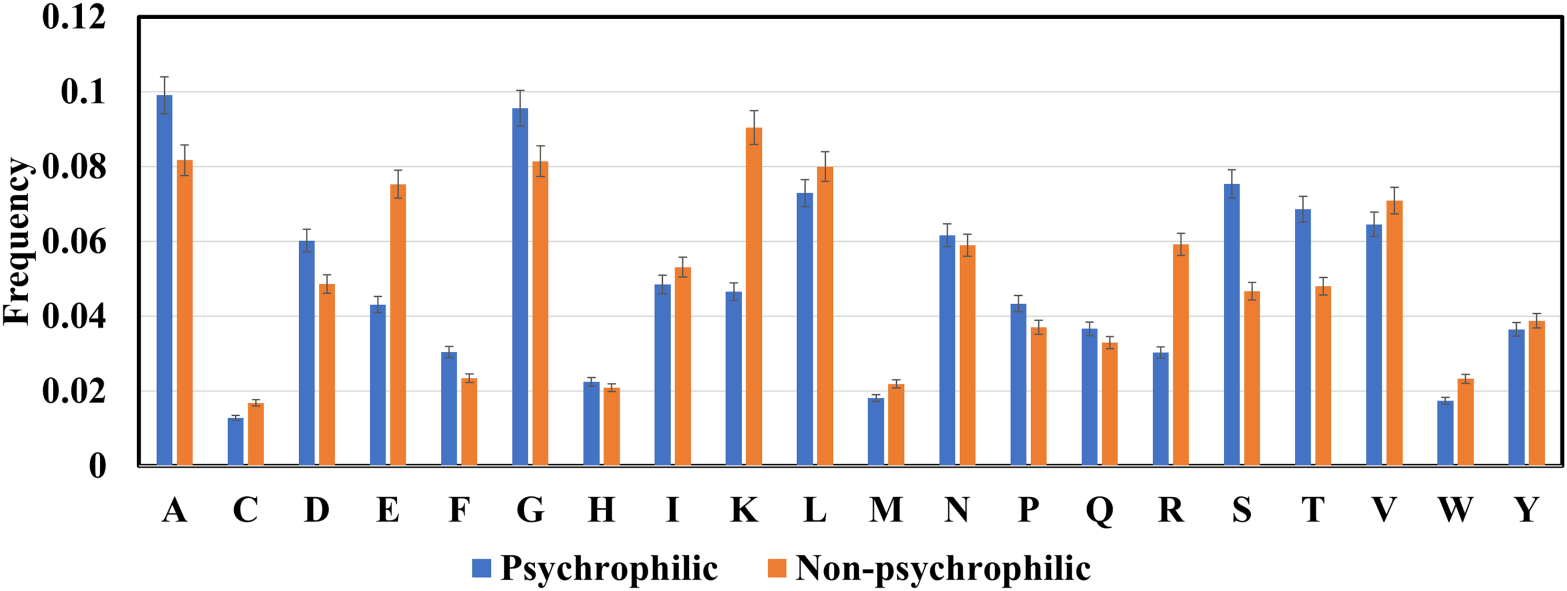
The amino acids composition in the psychrophilic and non-psychrophilic proteins.

The frequencies of 20 amino acids in psychrophilic, mesophilic, and thermophilic proteins were computed (Figure 3). Ala, Gly, Ser, and Thr amino acids in psychrophilic enzymes were higher than those in non-psychrophilic proteins, whereas the other amino acids Glu, Lys, and Arg were lower than, the non-psychrophilic proteins, and aliphatic amino acids Ile,Val, and Leu were slightly lower than non-psychrophilic proteins.

Many studies have demonstrated that psychrophilic proteins maintain their high catalytic activity at low temperatures mainly due to their more flexible structures (Siddiqui and Cavicchioli, 2006; Santiago et al., 2016; Åqvist et al., 2017; Arcus and Mulholland, 2020). Several factors contribute to conformational flexibility, such as reduced inter-domain and inter-subunit interactions, fewer inter-protein disulfide bonds, and reduced hydrogen bonds and electrostatic interactions. Glycine and alanine are very small amino acids, and the side chains are a hydrogen atom and a methyl group, respectively. And the comparative analysis focused on the dataset from psychrophilic and mesophilic proteins also showed that Ala and Gly residues are over-represented. Increased levels of Gly residue have been suggested to be related to psychrophilicity.

A higher percentage of serine and threonine is also found in the psychrophilic proteins. The study of Subbiah *et.al* on the classification rules for psychrophilic and mesophilic proteins showed that when the percentage of Ser and Thr is higher than certain values, the proteins would be classified as psychrophilic proteins (Nath et al., 2012). Meanwhile, a pairwise comparison of proteins from cold-adapted archaea revealed that there was higher content of non-charged polar residues, especially threonine (Berthelot et al., 2019; Bargiela et al., 2020). Ser and Thr are uncharged polar amino acids and prefer to reside on the surface of the psychrophilic proteins (Jahandideh et al., 2007), therefore they tend to have more interactions with water molecules around proteins (Sun et al., 2020). Structural and molecular dynamics (MD) analysis of homologous psychrophilic, mesophilic, and thermophilic counterparts of serine proteases (Tiberti and Papaleo, 2011; du et al., 2017) and serine hydroxy methyltransferases (Zhang Z. B. et al., 2021) reported that psychrophilic proteins formed more hydrogen bonds with solvent water molecules. Further analysis revealed that the content of serine in psychrophilic proteases and hydroxy methyltransferases is greater than in homologous mesophilic and thermophilic proteins. Although these studies only include several types proteins, it seems that serine and threonine involve increasement of surface hydrophilicity *via* forming more H-bonds with water molecules to enhance the mobility and flexibility of psychrophilic enzymes.

The charged amino acids in proteins are divided into two groups: basic amino acids which are lysine, arginine, and histidine; while acidic amino acids including glutamic acid and aspartic acid. Basic and acidic amino acids have positive and negative charges under physiological conditions and thus form higher number of salt bridges and electrostatic interactions. Therefore, more charged residues were found in the non-psychrophilic than in psychrophilic proteins to maintain the conformational stability of protein structures (Gianese et al., 2002; Tiberti and Papaleo, 2011; Wu et al., 2017). However, Figure 3 indicated that Asp amino acid favors psychrophilic proteins. It seems that Asp is unstable at high temperatures, thus the increased content of Asp contributes to the structural flexibility of the psychrophilic proteins. Another acidic amino acid, Glu, contributes to the formation of helical structures, and structure comparative analysis shows that the content of helical structures is lower in the psychrophilic proteins than in the mesophilic proteins (Metpally and Reddy, 2009), thus the reduced content of Glu maintains the thermolability of psychrophilic proteins. In contrast, the higher charged amino acids in thermophilic proteins are essential to protein stabilization at high temperatures (Zhang and Fang, 2006a,b; Gromiha and Suresh, 2008; Taylor and Vaisman, 2010; Ai et al., 2018). For example, a model only using Lys residue feature to classify thermophilic and non-thermophilic proteins reached 76.41% accuracy, a striking difference between the thermophilic and non-thermophilic proteins (Guo et al., 2020).

The content of three aliphatic acids (valine, leucine, and isoleucine) in psychrophilic is slightly lower than in the non-psychrophilic proteins. The aliphatic amino acids maintain the conformational stability of the protein structure through hydrophobic interactions. Many findings have demonstrated that psychrophilic enzymes possess reduced core hydrophobicity (Lonhienne et al., 2000; Feller and Gerday, 2003; Siddiqui and Cavicchioli, 2006; Åqvist et al., 2017; Arcus and Mulholland, 2020). Such as fewer Ile residue were found on the core of the psychrophilic citrate synthase, trypsins, and AHA (Siddiqui and Cavicchioli, 2006). In other comparative studies, fewer Leu residues were proposed to contribute to the reduced hydrophobic interaction within the protein (Zhou et al., 2008).

In conclusion, psychrophilicity is the consequence of numerous characteristics, and different families of psychrophilic enzymes may adopt one or several strategies to adapt to low temperatures, which causes no structural features that is always presented in all psychrophilic enzymes.

### Feature importance

To identify the key amino acids, the influence of different features subset on the accuracy of the model was investigated. According to the residue differences between psychrophilic and non-psychrophilic proteins, the feature of hydrophobic (ILV), charged (KRED), aromatic (WYF), and polar uncharged (STQ) residues were explored. These residual features were removed, respectively, and the remaining residues were used to build the classification model. It is demonstrated that by removing the descriptors, the performance of all established models was decreased, especially the sensitivity values decreased significantly (Table 3). The largest degradation in performance was the models that excluded from the KRED and STQ residues. It is deduced that the charged amino acids and non-polar amino acids play a vital role in discriminating psychrophilic from non-psychrophilic proteins. However, the Acc and MCC values did not decrease significantly, because the number of psychrophilic proteins was smaller than that of non-psychrophilic proteins, thus the subtle change of TP values had little effect on Acc and MCC.

**Table 3:** Prediction results of using different AAC descriptors for psychrophilic and non-psychrophilic proteins.

Descriptor	Sn	Sp	Acc	MCC
AAC	0.780	0.859	0.806	0.550
AAC-WYF*	0.667	0.860	0.803	0.536
AAC- ILV*	0.660	0.869	0.807	0.544
AAC-KRED*	0.615	0.879	0.800	0.520
AAC-STQ*	0.631	0.865	0.795	0.514
AAC-KREDSTQ*	0.537	0.866	0.768	0.426

AAC-WYF* means the model constructed by the deletion of WYF amino acids frequency features, and other descriptors in the table were constructed similarly to this descriptor.

## Conclusion

In this study, the iLearnPlus platform was utilized to develop binary and ternary classification machine learning models to identify psychrophilic proteins. The models were constructed based on AAC, DPC, and the combination of two descriptors, respectively. In the binary classification models, the SVM model using AAC descriptor achieved the highest prediction accuracy with 80.6%. Whereas, the accuracy of the SVM model using the DPC descriptor was 74.7%. It indicated that AAC descriptor can better distinguish psychrophilic and non-psychrophilic proteins than DPC descriptor. At the same time, the distribution frequency difference of AAC in psychrophilic and non-psychrophilic proteins was compared, and the influence of different amino acid composition in AAC descriptor on the accuracy of the model was identified. This also provides the interpretability of the model for AAC descriptor could better distinguish psychrophilic from non-psychrophilic proteins. The frequency of amino acid composition results demonstrated that the abundance of Ala, Gly amino acids in psychrophilic proteins might provide greater conformational mobility. Meanwhile, a higher number of Ser and Thr amino acids in psychrophilic enzymes could enhance the interaction between the protein with water molecules, thus inducing the protein structural flexibility. Moreover, the decreased charged amino acids in psychrophilic proteins tend to form fewer salt bridges and hydrogen bonds within the protein and be important for the structural plasticity of cold-adapted enzymes. Non-psychrophilic proteins showed favor for aliphatic residues (Leu, Ile, Val) than psychrophilic proteins. In a word, the sequence changes of psychrophilic proteins are related to the protein structural flexibility. Additionally, compared with binary classification, the feasibility of ternary classification was also investigated. The proposed machine learning model is expected to be useful for the identification of psychrophilic enzymes and can provide meaningful guidance for the modification of cold-adaption of enzymes.

## Data availability statement

The datasets presented in this study can be found in online repositories. The names of the repository/repositories and accession number(s) can be found in the article/Supplementary material.

## Author contributions

AH: conceptualization, methodology, software, investigation, and writing-original draft. FpL: supervision and funding acquisition. FfL: writing—review & editing and funding acquisition. All authors contributed to the article and approved the submitted version.

## Funding

This work was supported by the National Key R&D Program of China (2021YFC2102700), National Natural Science Foundation of China (No. 32272269) and Tianjin Research Innovation Project for Postgraduate Students (2021YJSB214).

## Conflict of interest

The authors declare that the research was conducted in the absence of any commercial or financial relationships that could be construed as a potential conflict of interest.

## Publisher’s note

All claims expressed in this article are solely those of the authors and do not necessarily represent those of their affiliated organizations, or those of the publisher, the editors and the reviewers. Any product that may be evaluated in this article, or claim that may be made by its manufacturer, is not guaranteed or endorsed by the publisher.

## Footnotes

^1^https://github.com/ailanhuang/A-machine-learning-model-for-psychrophilic-proteins
