# Supplementary material for: Discrimination of psychrophilic enzymes using machine learning algorithms with amino acid composition descriptor
Source: Front Microbiol. 2023 Feb 13;14:1130594. doi: 10.3389/fmicb.2023.1130594 (PMC9968940; doi:10.3389/fmicb.2023.1130594)
Supplement: Supplementary file 1 [file Data_Sheet_1.docx]

**Discrimination of psychrophilic enzymes using machine learning algorithms with amino acid composition descriptor**

Ailan Huang^1^, Fuping Lu^1,2^, Fufeng Liu^1,2*^

^1^ College of Biotechnology, Tianjin University of Science & Technology, Tianjin, P. R. China

^2^ Key Laboratory of Industrial Fermentation Microbiology, Ministry of Education; Tianjin Key Laboratory of Industrial Microbiology; Tianjin, P. R. China

^*^Correspondence

Fufeng Liu, Phone: +86-22-60602717; Fax: +86-22-60602298; E-mail: fufengliu@tust.edu.cn

**Table S1.** Prediction accuracies of ternary classification model for

mesophilic and thermophilic proteins

| Class | Descriptor | RF | SVM | Bayes | KNN |
| --- | --- | --- | --- | --- | --- |
| Mesophilic(M) | AAC | 0.717 | 0.717 | 0.703 | 0.740 |
|  | DPC | 0.657 | 0.676 | 0.676 | 0.630 |
|  | AAC+DPC | 0.699 | 0.726 | 0.689 | 0.648 |
| Thermophilic(T) | AAC | 0.821 | 0.804 | 0.793 | 0.755 |
|  | DPC | 0.739 | 0.788 | 0.734 | 0.707 |
|  | AAC+DPC | 0.793 | 0.804 | 0.750 | 0.717 |

**Sequences of psychrophilic proteins**

>sp_P29957

MKLNKIITTAGLSLGLLLPSIATATPTTFVHLFEWNWQDVAQECEQYLGPKGYAAVQVSPPNEHITGSQWWTRYQPVSYELQSRGGNRAQFIDMVNRCSAAGVDIYVDTLINHMAAGSGTGTAGNSFGNKSFPIYSPQDFHESCTINNSDYGNDRYRVQNCELVGLADLDTASNYVQNTIAAYINDLQAIGVKGFRFDASKHVAASDIQSLMAKVNGSPVVFQEVIDQGGEAVGASEYLSTGLVTEFKYSTELGNTFRNGSLAWLSNFGEGWGFMPSSSAVVFVDNHDNQRGHGGAGNVITFEDGRLYDLANVFMLAYPYGYPKVMSSYDFHGDTDAGGPNVPVHNNGNLECFASNWKCEHRWSYIAGGVDFRNNTADNWAVTNWWDNTNNQISFGRGSSGHMAINKEDSTLTATVQTDMASGQYCNVLKGELSADAKSCSGEVITVNSDGTINLNIGAWDAMAIHKNAKLNTSSASSTESDWQRTVIFINAQTQSGQDMFIRGGIDHAYANANLGRNCQTSNFECAMPIRHNNLKNVTTSPWKANDNYLDWYGIENGQSSEAEGSATDWTTNVWPAGWGAEKTVNTDGFGVTPLNIWGEHYWMLDVDMDCSKAVNGWFELKAFIKNGQGWETAIAQDNAPYTSTNHMAQCGKINKFEFNNSGVVIRSF

>sp_Q8GIX7

MKAAVLHEFGQSLQIEEVDIPTPGAGEIVVKMQASGVCHTDLHAVEGDWPVKPSPPFIPGHEGVGLITAVGEGVTHVKEGDRVGVAWLYSACGHCTHCLGGWETLCESQQNSGYSVNGSFAEYVLANANYVGIIPESVDSIEIAPVLCAGVTVYKGLKMTDTKPGDWVVISGIGGLGHMAVQYAIAMGLNVAAVDIDDDKLAFAKKLGAKVTVNAKNTDPAEYLQKEIGGAHGALVTAVSAKAFDQALSMLRRGGTLVCNGLPPGDFPVSIFDTVLNGITIRGSIVGTRLDLQESLDMAAAGKVKATVTAEPLENINDIFERMRQGKIEGRIVIDYTM

>sp_C7F6X3

MSLLSIITIGLAGLGGLVNGQRDLSVELGVASNFAILAKAGISSVPDSAILGDIGVSPAAATYITGFGLTQDSSTTYATSPQVTGLIYAADYSTPTPNYLAAAVANAETAYNQAAGFVDPDFLELGAGELRDQTLVPGLYKWTSSVSVPTDLTFEGNGDATWVFQIAGGLSLADGVAFTLAGGANSTNIAFQVGDDVTVGKGAHFEGVLLAKRFVTLQTGSSLNGRVLSQTEVALQKATVNSPFVPAPEVVQKRSNARQWL

>sp_P16304

MNLVLMGLPGAGKGTQGERIVEDYGIPHISTGDMFRAAMKEETPLGLEAKSYIDKGELVPDEVTIGIVKERLGKDDCERGFLLDGFPRTVAQAEALEEILEEYGKPIDYVINIEVDKDVLMERLTGRRICSVCGTTYHLVFNPPKTPGICDKDGGELYQRADDNEETVSKRLEVNMKQTQPLLDFYSEKGYLANVNGQQDIQDVYADVKDLLGGLKK

>sp_Q12XX5

MKHIIFTDLDGTLIDHDTYSYDAARPALDLLKEKEIPLIFCTSKTRAELEVYVDELECHHPFISENGGAIFIPKDHFSIELKDVHEIGNYKVIEFGTSYTRIRGVLEDIRKKTGFKITGFGDLDAEGVSKDTGLDIRSAKLAKLREYDEAFRLEEDENATAKVIELIHAAGLNYTKGGRYWHIMGDNDKGKAVRALTEIYRQQFTEVVTIALGDSLNDLPMLKAVDIPFLVQKPDGKYDPSIILTEIKHAEGIGPVGWNNAIMDLIGKNNNI

>sp_Q9KI47

MINDKLPKIWHGGDYNPEQWDSKEIWDEDVRMFKLAGIDVATLNVFSWALNQPNEDTYNFDWLDEKINRLYENGIYTCLATSTAAHPAWMAKKYPDVLRVDFYGRKRKFGSRHNSCPNSPTYRKYSERIAETLAERYKDHPAVLIWHVSNEYGGYCYCDNCQDAFRNWLSDKYGTLEKLNKAWNTGFWGHTFYEWDEIVAPNMLSEKREDNVSDFQGISLDYRRFQSDRLLDCYKLEYNAIRKHVPTSIPITTNLMGTYPMLDYFKWAKEMDVVSWDNYPSIDTPFSYTAMTHDLMRGLKGGKPFMLMEQTPSQQNWQPYNSLKRPGVMRLWSYQAIGRGADTILYFQLRRSVGACEKYHGAVIEHVGHEHTRVFNEVAQLGQELNGLSDTLLDARVNAKVAIVFDWENRWATELSSGPSVSLDYVNEVHKYYDALYKLNVQVDMIGVEEDLSKYDVVIAPVLYMVKEGYAAKVEKFVENGGTFLTTFFSGIVNETDIVTLGGYPGELRKVLGIWAEEIDALHPDETNQIVVKGSRGILSGKYSCNLLFDLIHTEGAEAVAEYGSDFYKGMPVLTVNKFGKGKAWYVASSPDAEFLVDFLQTVCEEAGVEPLLDVPAGVETTERVKDGQTYLFVLNHNNDEVTIELHGSQYREVLTDEQVSGNLVLKEKGVLILAKV

>sp_Q9K4Y9

MENLLSVKDLSKQQILDLLALAKAVKANPAEYSQALAGKSIVTIYEKPSLRTRVTFDIGIHKLGGHAVYLDAQNGAIGERETVKDFAANISRWADAIVARVVSHKTLEGLVEHGSVPVVNSLCDLYHPCQALADFLTISEHYEDVSKVKLAYVGEGNNVTHSLMLTGAILGAEVTAVCPRGSSPDAQIVKQAMALAEISGGKINVTDNLDDIVDYDVIYGDTWVSMGDDTPLAQVKEKYMPYQINKALLMRTGIKHVLHCQPAHRELEITSEVMDGEHSLIFDQAENRMHAQNAVLLTLLK

>sp_P50921

MRHPVVMGNWKLNGSKEMVVDLLNGLNAELEGVTGVDVAVAPPALFVDLAERTLTEAGSAIILGAQNTDLNNSGAFTGDMSPAMLKEFGATHIIIGHSERREYHAESDEFVAKKFAFLKENGLTPVLCIGESDAQNEAGETMAVCARQLDAVINTQGVEALEGAIIAYEPIWAIGTGKAATAEDAQRIHAQIRAHIAEKSEAVAKNVVIQYGGSVKPENAAAYFAQPDIDGALVGGAALDAKSFAAIAKAAAEAKA

>sp_Q489W3

MIYQGKSLSAQLLEDGIVEFKFDAQGSVNKFDQATFEEYIAVVAAINNCSEAKGVIVTSGKSTFIVGADITEFLVSFSQPEDALASWAKKASDVFDSFEDIQLPTIAAINGIALGGGCEMTLACDYRVAATTASIGLPEVKLGLMPGFGGTVRLPRLIGFDNAATWMSTGKAFKPAAALAQGAIDAVVEPENLQAAAISMLKLAIDGKLDWRAKRQPKLEALKLSPTELIMSSTTCKGMIAAKAGKHYPAPMVMINTLIASANLDRTGAMAAENTGFAKLAKTDAATAQIGLFMADQVIKGKAKKASKLATKAVNKAAVLGAGIMGGGIAYQSAYKGTPIIMKDINDQALDLGLTTATGILTKQVERGRMNAKKMAGVLNNITPSLSYDSVKDVDIVVEAVVENPKVKGMVLAEVEGVIGEDAILTSNTSTISIDLLAQSVKRPQNFCGMHFFNPVNKMPLVEVIRGKDTSDETVAAVVAYAAKMGKSPIVVNDCPGFYVNRVLFPYFAGFSQLVLEGADFTAIDKVMEKQFGWPMGPAYLLDVVGVDTADHCTGVMSSGFPTRMKKIDNDPVSTLYANERLGQKNGKGFYDHIKDKRGRPMKVPAPVAYELLGQHCADKKDFSSEEIIARMMIPMVNEVVRCLEEGVVDTAAEADMGLIYGVGFPPFRGGAIRYLETLGLDNFIAMADKYTDLGEIYHVTDGLREMAKSGKSYFTTDVKLA

>sp_Q47UP5

MSEFKEVTVTKAASVYFDGKVTSRKVTFNDGSFKTLGIMMPGEYKFGTNEEELMEITAGECEILLAGATEWQNISDGQSFGVPANSSFEVRAKTLIDYCCTYIS

>sp_Q47UE0

MSLSVVILAAGKGTRMRSSLPKVLHSVAEKPMVGHVIDSARQLGASNIYVVYGFGGDVLKATLTKDNTGDDLTFVEQVEQLGTGHAVDQASPFLTDDEDVLVLYGDVPLTKVSTLESLLAAKPTDGMALLTVHLANPMGYGRIVRQEISGKQQVVGIIEQKDANEEQLKINEANTGILLANGGDLKRWLSNLSSDNAQGEYYLTDIIAAAHGEGKVVATAHPETEIEVEGANNRVQLATLERAYQARIAEELMIAGASLRDPARIDVRGNLTTGTEVSIDINCIFEGEVSLADNVQIGANCIIKNSTIGANVEIKPNSIIEDTIIEADCSVGPFARLRPGSVMKQDSHVGNFVEMKKTTLGVGSKAGHLSYLGNAEIGTKVNIGAGTITCNYDGVNKSTTEIGDNAFIGSNSSLVAPVIIGNSATVGAGSVISKEVEDNDLALTRAKQRNIAGWQRPVKKS

>sp_P41560

MTNKIIIPTTGDKITFIDGKLSVPNNPIIPYIEGDGIGVDVTPPMLKVVNAAVAKAYGGDRKIEWLEVYAGEKATKMYDSETWLPEETLNILQEYKVSIKGPLTTPVGGGMSSLNVAIRQMLDLYVCQRPVQWFTGVPSPVKRPSEVDMVIFRENTEDIYAGIEYKAGSDKAKSVIKFLIEEMGASNIRFTENCGIGIKPVSKEGSQRLVRQAIQYAIDNNKDSVTLVHKGNIMKFTEGAFKDWGYELAIEEFGASLLHGGPWCSLKNPNTGKEIIIKDVIADAMLQQVLLRPAEYSVIATLNLNGDYLSDALAAQVGGIGIAPGANLGDEVAVFEATHGTAPKYAGKNKVNPGSVILSAEMMLRHMGWLEADLLLKGMSGAIQAKTVTYDFERLMDDATLVSCSAFGDCIIDHM

>sp_Q25QU7

MKVTIVGAGNVGATCADVISYRGIASEVVLLDIKEGFAEGKALDIMQCATNTGFNTKVSGVTNDYSKTAGSDVVVITSGIPRKPGMTREELIGINAGIVKTVAENVLKHSPNTIIVVVSNPMDTMTYLALKATGVPKNRIIGMGGALDSSRFRTYLSLALDKPANDISAMVIGGHGDTTMIPLTRLASYNGIPVTEFLSEEVLQKVAADTMVGGATLTGLLGTSAWYAPGASVAYLVDSILNDQKKMIACSVFVEGEYGQNDICIGVPCIIGKNGVEEILDIKLNDQEKALFAKSADAVRGMNDALKSILV

>sp_Q12WS1

MADIIIKNGYVLTMDPEVDDIPNGVVVIEGGKIVEVAETTSATANTVIDAQGGVVMPGFVNTHTHAGMTLFRGYADDLPLAQWLQEHIWPAEAELTASDVLAGTRLACLEMIKSGTIAFADMYFFMEEVGKAVEECGLRAALSYGMIELWDDEKGTNELKKGREFVKEWNGKAEGRISVMYGPHAPNTCSKEFLSKVKEQAIADNVKIHIHVLETEAELNQMKEQYGMCSVNMLDTIDFFGPGVLAAHCIWLSDGDMDILADNNVNIAHNPVSNMKLASGVAPVMKLLDKGANVCLGTDGCASNNNLDMFDEMKTAALLQKVDTMDPTALPAKQVLEMATVNGAKALDINSGVLRKDYNADVIIIDMNKAHLSPLFDVPSQLVYSATGNDVRTTIVNGVVLMDERKVLCMNEQQVINDAKQAASDLVSRVDAKN

>sp_Q47WB7

MSDVSGKVNLLNFDHKSMREYLESIGEKPFRADQIMKWIYHFGYSDFEQMTNINKKLREKLQRNCIISAPDISEKQVSEDGTIKYALKLEGGQEVETVWIPENDRATLCVSSQVGCALECTFCATAQQGFNRNLSMAEIIGQVWRVANDIGATRIAGTRPITNIVMMGMGEPLLNMKNLIPALDTMLNDLGYGLSKRRVTVSTSGVVPALDMLKAKIDCALAISIHAPNNKLRDELVPINKKYPLEDFIAAAGRYIEGSKANKQATIEYVMLDHVNDSTDQAHELAHALKGLPSKINLIPFNPYPGSPYSRSSNSRIDRFDKVLQSYGLTVITRRTRGEDIDAACGQLAGDVFDRTKRSVINAAKNAEISSLKNQPKADTISIKVV

>sp_Q487C7

MKFIVKLQAEITIKSRPVRKRFTKILESSVKNVLRRIDEQVTTRMNWDNIEVNTKDNSPENRERLVEALKCIPGIPMFLEVQQSEFVDVHDIYEKTLAVHAESIENKTFCVRAKRTGNHDFNSLKVEQYVGGGLNQQVESAKVKLKNPDVTIHLEIKNKDLFIVTQRHKGLGGFPIATQEDVLSLMSGGFDSGVSSYQMIKKGARTHYCFFNLGGSAHEVGVKQISYYLWNKFGASHKVKFFAVDFEPVVAEILENVENSQMGVVLKRMMIRAATKIAERGKIQALVTGESLGQVSSQTLTNLNVINRVTDTLILRPLAAYDKQDIIDIARKIGTEEFSKTIPEYCGVISKKPTVKAVLSKVEEEEGNFDFDVLDKVVSETRVYDIRDIGKEAEEEIHAVDLVENIPENAVVVDIRSPEEEEDKPLELGDVEVKHIPFYKLSTQFGDLDMTKEYLLYCDHGVMSKLQALYLLDNGFKNVKVYRP

>sp_Q9S5V6

MTLQNFYRDTWAEINLDAIFENAANMKKHLPPEITLFAVVKANAYGHGDVEVAETAIQAGAGYLAVAFLDEALALRKKGITAPILVLGASRPEDAQIAARESITLTVFQAGWLEAAQSFLEGTVLTIHLKCDSGMGRIGIRKQEEMNEIERFLQKTKCFVLEGIFTHFATADQLDTEYFSKQLARFEEMLTWLKEKPKYVHAANSAALLRFPNAVFNSVRMGISLYGLSPSMEMKEVLPFALRPAFSLKTKLVHVKNISKGQSVSYGATYTAEEDTWIGTLPIGYADGWIRMLQGQEVLLEGGRSPLVGRICMDQCMVKLSREFPVGTEVTLIGKNGTECITVDDIAEKLNTINYEVTCMISSRVPRMYLKDGRITGVVNQLI

>sp_Q47WQ9

MTTRYIFVTGGVVSSLGKGIAAASLAAILEARGLKVTMLKLDPYINVDPGTMSPIQHGEVFVTEDGAETDLDLGHYERFIRTKMTKRNNFTSGRIYQDVLARERKGEFLGATIQVIPHITNDIKRRVIEGAEGYDIAMVEIGGTVGDMESQPFLEAIRQLALEVGRDRAMFMHLTLVPYLAAAGEIKTKPTQHSVKDLRSIGIFPDILVCRSDRAIPNAERAKISLFTNVEEKAVVSMRDVDSIYKIPALLKAQGTDEIVVKRFGLDVPEADLTEWEEVLYHEANPIGEVTIGMVGKYTELPDAYKSVNEALKHAGLKNQVTVNIKYIDSQDVEVKGVEILANLDAILVPGGFGERGVEGKILTAQYARENKVPYLGICLGMQVALIEFARNVAGLTDAHSTEFNSETPHPVVGLISEWLDEEGQVEYRNEQSDLGGTMRLGSQLCHLVKGTKACDVYGSETINERHRHRFEVNNNYREQLSKAGLIFSGLSSDKSLVEVIEIADHPWFIAGQFHPEFNSTPRDGHPLFESFVAASFKLQKS

>sp_Q12Y03

MVEMFYDKDADLGALKGKIIAVMGYGSQGHAQAQNLHDSGLDVVIGLREGSRRWKQAEDDGLKVMTVADAAKAADVVQILLPDEIQSKVYYSEIEPGLEAGNSLVFSHGFNIHYNQIVPSKDLDVYMVAPKSPGHLVRRTYKEGAGVPGLIAVYQDASGNALEMALAHAKGVGCTRAGVIETTFREETETDLFGEQVDLCGGVASLIKTSFEVLVEAGYQPEMAYFETLHELKLIVDLIHEGGLEKMWYSVSNTAEYGGLTVGPQIINEESREAMYVALERIQNGEFAREFVLEGQTNHAVLTSMERLEKEHPVEVVGKKLRAMMPWLNSELNEE

>sp_P85302

ATDIRQVVDSTVEPLMQQQDIAGLSVAVIQNGKAQYFNYGVANKDSKQPITENTLFEIGSVSKTFTATLAGYALANGKLKLSDPASQYLPALRGDKFDHISLLNLGTYTAGGLPLQFPEESDNTGKMISYYQHWKPAFAPGTQRLYSNPSIGLFGHLAAQSLGQPFEKLMEQTVLPKLGLKHTFISVPETQMSLYAQGYDKAGKPVRVSPGALDAEAYGIKTSTSDLIHYVEVNMHPAKLEKPLQQAIAATHTGYYTVDGMTQGLGWEMYPYPIKVDALVEGNSTQMAMEPHKVNWLTPPQAAPLDTLVNKTGSTGGFGAYVAYVPSKGLGVVILANKNYPNAERVKAAHAILSAMDQ

>sp_Q47VH8

MKLIIVSGRSGSGKSVALRVLEDLGYYCVDNIPINLLPALTHTVINDYENVAVSLDVRNLPKDPEDIPEIIAYLPKAVDVNTLFLDADDNDLIRRFSETRRLHPLIKENMALDQAIALEKSLLEPISTNADLYINTSQLSPHQLADLVRERILGKKTGSMVLVFESFGFKHGIPVDADYVFDARFLPNPFWEKSLKGQTGVDQEVKDFLASQAIVTKFIWQINSFMMTWLPHLERNNRSYVTIAIGCTGGKHRSVYIAEMLAKNFRKERDDIQTHHRDIDIKST

>sp_Q481U4

MIYSTTESIPGKEIEEIVGVVTGNVVQAKHIGRDIMAGLKSIVGGEIRGYTEMLTDARDIAIQRLVANAEEKGADAVVGIRFTTSAIMDGSSEIMVFGTAVKLKK

>sp_Q489A5

MLTNMSLFNEKKLISFWHDNKELLFYFTRRVRREQIQVVAGYLSYVCLMSLVPLIVVMLSVMTAFPLFAELQQSIEQFVYQNFVPAAGDVVQQYLTGFVANASKMSAVAISFLFLAALLLISSIDNTFNKIWRVTDKRRTITSFAMYWMVLTLGPILVGASIALSSYLVSIVAVDEYDVLGLSDMFLRMLPLLSSIIAFIILYIAVPNKAVPFRFALSGAIVAGVLFELAKKAFALYITAFPSYQVIYGALATIPIIFLWVYVSWIIVLTGALITVSLQEYEILKEKKVKESDRAQVKDEEIS

>sp_Q47Y78

MAHVIDLQVACTPTKLPTKEQFQLWVDTALAEVSSSPNQDFELTIRLVNNEESQQLNKQYRDKDKPTNVLSFPFEVPDGIELNLLGDLIICIEVMKQEAQEQNKALFEHWAHLVIHGCLHLVGFDHISDTEALEMESIEITILEKLGISNPYLEQ

>sp_Q12TI1

MRQPTVAGKFYPLSTKALRKEIVKCFHGLEIMSEDVIGAVVPHAGYVYSGPVAAHAFARLPKADTYVIFGPNHTGYGSPVAMSQDVWNTPFGDVETDRELGKLLAGTIIDMDEVAHRYEHSVEVQIPFLQYLFGSDFKVLPICMGMQDEDTAVEVGLEVARAVKESGKKVVFIASSDLSHYVPQEKAEKSDNYLIDAILDMDVPEIYRRKYEKDITACGYGPITAMLTAAKECGAKNTELVKYGTSGDVTGDPMVVGYAAIIVK

>sp_Q47YL9

MITKLPQVLSPIQVASIIQLIEHGSFNSGKDTAGWHAKAVKNNLQWQGETELNEQIQTGIQGALTQHPQFTGAAYAKSMMPFIISESTLGGGYGDHIDDALMVNETVLRTDISCTLFLTPPQDYEGGELVMNLSGMEMAFKLNAGDAIIYPSTTLHRVNPVTSGSRKVALTWIESHIPQASQREILFDLDCARKDIMEHHGKTDAFDRITKTHANLLRQWAMT

>sp_Q12WH6

MNNVPNVEKQIGIDLYTTKTPGIGGKLRQQTEDFGVIEITNREEGTEGKYLILELTKRNWETHHLIRDLTRILRISQKRVGFAGTKDKRAVTTQKISIYDMEEEALQNVHLKDTELKILGRSNKSLELGDLTGNEFIITVRDIDLDEKELESRLSQTTASIKEQGGVPNFFGIQRFGALRPITHVVGESIVRNDIEKAAMAYIAASYPDEPEDTQEVRNRVFETKDYIEGLKGYPLQLRYERAMMHHLVSKPEDYAGSFETLPANIRKMFVHAYQSYIYNTIICSRIKKGLPLNRAVVGDIVCFKNKAGLPDKSRNERVTEDNIDGMNNLVKRNRAFVTAPLVGYSSELASGVPGEIEREVIEELNVPIEGFKVPSMEELSSKGLRREILLSTDPKYFIEEDELNEGKYRVTLDFSLPKGSYATTILREYMKVEPLKMS

>sp_Q12YU9

MCGYQVRIHDMPSEDRPRERLLKHGPGFLSNAELLSVILRTGSKDENVVSMSSRILSEYNLKQLSQANISQLTKIRGIGPAKASQIAALFELARKLEIFTDDPKRKIRSANDVYSLLYPRHRELKKEHLTALYLDTKNNIIKEEVISIGSLNANIVHPREVFKSALMESSASVILTHNHPSGDPAPSREDIAVTEKLVEGGKILGISVLDHVIIGDGRYVSLKEEGYIS

>sp_Q47XK4

MRYALGIEYDGKNYCGWQRQNNVITVQEKLEKALSKIADEKIEVVCAGRTDTGVNATNQVIHFDTEKKRKDTAWTLGVNTHLPSDVAVAWVKKVDDDFHARFSATARNYRYIIYNKPLRSAILSHGISHCHFALDENLMQQGADYLLGKHDFTSFRTVHCQSHSAVRTIKHCRVTRQGDYLVVDIKANAFLHHMVRNVVGSLMRVGQSQETPHWMKEVLLAKNRCVAGVTAPPEGLYFVDVDYPENFELPKSRLGPLFL

>sp_Q47U34

MAKNNSTPQKLVITGIKGYQRFISPLLGSNCRFTPSCSAYATEAINRFGVIKGGWLASKRILRCHPLNDGGEDPVPPIKKSK

>sp_Q486M0

MSSLENQLLIAMPSLGDPYFNKTVTYICEHNEDGAMGLIINLPVNITLADLLKQIEPDEGDKTGNVNSNSELTKSDDVNDITLVTDITNSLEQLVLAGGPIAQQRGFVLHSSQPGWSSSLVLSKELMITTSKDILMALGTQQAPEQFIVTLGYAGWGPGQLEQELQANSWLTTPADIEILFKTPIEQRWKKATEKLGIDLAHLSTDIGHA

>sp_Q488Y7

MRHRQSGRQLNRNSSHRQAMFRNMASSLVKHGVIKTTVAKAKELRRVLEPLITLAKTDSVANRRLAFARTQDKEVVGILFNELGARYQERPGGYTRILKCGFRTGDKAPMAYIELVDRPVVEDAPEVVEESAEA

>sp_Q47Y27

MIDAEGYRANVGIVIINDMGQVFWARRYGQHSWQYPQGGVDEGETAEQTMYRELHEEVGLKPEHVKIVASTKHWLKYKLPKRYIRHDSKPVCIGQKQKWFLLKLTAAESSVDLLHSSHPEFDDWRWVSYWYPVRQVVSFKRDVYRMVMKEFANFALPLTYERRPDRRKRRA

>sp_Q12XS4

MDEQMNKKYLANKLFETQFALEDLREIVRQSLPTGMSNDQSEHYDEIVSGLGGLLEDLGKKNGQTQPVKIVGDLMCRTREEEFINIQPIQAGGRLTPEARKAVIAYGDGYSTCDNCRKPFRLDKIEKPSISTFHTDLAEFVGMDQARVVPGARRGFQAVASSIIEKGDTVVVSTFAHYTEFLAVEGAGGIVREAPVNEHNILTAESVAHKIDEVKRETGKLPALIMIDHFDYLFCNEHDIYGIGKVAQEYGIPFLYNGAYTVGIMPVNGQKIGADFVVGSGHKSMASAAPSGVLATTEEWADKIFRTTQMVGDVTGRKFGIKEVEFLGCTLMGAPLLSMMASFPHVKERTKHWDEEVKKSNYFINEFLRIEGNEVLSEFPRKHALSKVDTTGSFDKVAKTHKRKGYFFSDELKKRGIAGEFPGATRSWKMSTYGLSWDQIHYLSNSFIEIADKYDININ

>sp_Q47VK0

MKYRIKALLLASSLIITTITSVQAKEELLDRVAAIVNTGVVLESEVNDLLVNIKQQAKKNNQSLPSDKALRIQVMDKLINDSLLSQMGQRMGIQISDAQLDQTLNNMAREDKLTLAQFRQQVIDEGTSYEKYRENVRIELVSGEVSRNSVRRRIFVSPQEVDNLLKVMKEQSSNNVEYHLGHILIEFPADASQEDLAAAKTRATKVVELLNDGSDFAKIAITSSGDANALKGGDLGWKNINEMPTLFSELINDKPKDTIVGPIRTGLGYSIVKVLDIRGRKVVEVEEVKASHILIKPSIILSDEKAKSLLQGFLNQIDAGEATFEELAKEHSEGPTSVRGGDLGWADPKNYDPAFTEALATMKKGGYHKPFRSSFGWHIIKLEDRRMVDATSQLNENRAYQILFNRKYGMESTRWLKETRDEAYIEIFEQDNK

>sp_Q47ZS4

MNLDTTTIDSIVAQAEVAIAQASDPTALDQVRVNFLGKKGLFTEQMKGLGKLPKEEKPKMGQVINIAKQAVQKLLTERGELLRAQEIKEKLAAESIDVTLPGRGTQIGGLHPVTRTIARIESFFGDLGFEVKDGPEVEDDYHNFDALNIPEHHPARQDHDTFYFNPKLVLRTQTSGVQIRTMEVEQPPLRIISPGKVYRNDYDQTHTPMFHQVEGLMVDKDVSFTHLKGILHDFLHHFFEEEVEIRFRPSYFPFTEPSAEVDIMGKNGKWLEVLGCGMVHPNVLRSVGIDPEVYTGFAFGMGVERLTMLRYGVNDLRAFFENDLRFLKQFK

>sp_Q12V97

MFLDFIDQVTSILNDAVSSAGFEADDMELGPSQHADLSSRIAFRLASVAKQSPKDVAEKIAGEIVIPKGSFVEKVEALGPYLNIWAGRNFIEGTVLAIREQKEAFGGNFSEGRILLEHTSANPNGPLHVGHIRNSIIGDTLGRILKRAGYDVELHYYVNDMGRQIAIVSWALGYFEFDESSKPDHAIADVYIKANAELNAHPEKVAEIDKLMQLVEKGDAATIETFDKAVDLAVSGIKETLKKMNVAHDEFPKESSFIRSGDVSRIIEEIKATGRTEIDNGALVVNLQDYGFKKTLVIQRTDGTSLYTTRDLAYHEWKGERADRIIDVFGADHKLISGQLKATLNAIGKKEPEFVIFEFVSLPEGSMSTRSGKFISADDLLDQIKTQAYEEVDKRRPDMPDDFKATVAEIVGIGAVRYDIVKVSPEKSTVFDWKEALDFEKQGGPFIQYSHARACSILQKAKDEGLWSSEEPINTTLLVEDSEVSLIKKMAMFDNMLDQCAKELRPHTFAIYARELADAFNQFYRFVSVLNAEDEQLRSSRIALVDCARMVLANTLDTLGLGAPESM

>sp_Q934T0

MPNTHDTKNNVSPSEYAKFDPSTIHQRLNTSLSRPQLNSDGSIRHFLGVEGLNKAQLQAIIAKALFFEPSTRTRTTFEVAEKRLGANVLNLDIASSSAKKGESLRDTLWNLQAMTADIFVVRHSASGAAHFMATEVTPDIAIINGGDGWHAHPTQGMLDMLTIHREAPRPFEELSVAIIGDVKHSRVARSDISALQTLGVKDIRVIAPRTLLPKGIERFGVQVYEDMNSCVRDCDVIMGLRIQNERIGSPLLASSSEYYKQYGITPERVALAKPDALIMHPGPMNRGVEIASSVADGPQSVILKQVSNGVAIRMAVLALTMEGQRAHQANRG

>sp_Q480B1

MPKMKTNKGAAKRFKKTASGYKFKQAGLRHILTKRRTKVKRHLRAKCMIAASDIKSVKKLLRHG

>sp_Q488A1

MKVSELKAKSIEELNAELLELLREQFNYRMQASTGQLAQTHLLRIVRRNIARVKTIITEKAGK

>sp_Q47VQ1

MELDTTHISVLLNEAVDGLAITDDGCYIDCTFGRGGHSSVILSKLSDNGRLIAIDRDPTAITAAEKFKDDKRFLIEHQGFAALAEIAEKHELTGKVDGILLDLGVSSPQLDEAERGFSFMKDGPLDMRMDTSKGQTAAEWLAVADVEDITWVLRTFGEEKHAWRIANAIVDTREETPLTRTSQLAKLIKTTAPQREIKKHPATRSFQAIRMYINSELDQIEKALVASLDVLAEGGRLVVISFHSLEDRLVKQFMKKHSQGKKVPRGLPISEIELNKGKKLSLVGRRLKPSQTEVEENVRSRSSVLRVAERLERNTD

>sp_Q12XH7

MVRKILQKYGIRGGCHDQHFLIDERSLDSIVDQAELSEKDVVLEIGGGIGNLTERLLEKAGKVYVIELDPALVHVLKDRFSDNEKLEIIPGDVLKLDLPKFNKVVANLPYSISSPITFKLFKHEFELGILMYQYEFAQRMVAKANTENYSRLSVNTHYFADADIIMKIPPSAFSPPPEVWSAVVKVVPRPSSFHTEDPQFFLDLVTAVFLQRRKKLRNAIVKGNHLLNVPNIKQIVAELPEEFMSKRAENLEPHELAEIANFIFKMRSTS

>sp_Q12V70

MIDIDGSYGEGGGQIVRNAIALSAVTGKATSIKNIRKDRPNPGLSAQHVKAIGIAALLCDAKVEGIKIGSTNIAFFPQEIRGGKYTIDIGTAGSIALLLQCIMPIATYSNTNIKLEIKGGTDVSWAPSIDYLKNVTLSALSKMGYRCNIDILKRGYYPRGGGIVNAIIEPSHLVPDRFSEERGTIRGISHCSNLPEHVAQRQADKAKAILENAGHECSIETCRTDFTSTGSGITLYCGMKGSFVPGKRGTTAEKVGNDAATSLLDELLTPSSVDIHLADQLIPYLGLAEGGSFTVKEISPHTKTNIWVTEKFLDVKFKIEKRNDIVKISIQ

>sp_Q486U0

MSDYKQTLNLPATSFAMKGNMANREPNMLKYWAAKDLYGKIREAKKGKKSFILHDGPPYANGNIHLGHAVNKILKDIIVKSKNLSDFNSPFVPGWDCHGLPIELMVEKKVGKPGHKISASDFRQKCREYAAKQVNGQREDFKRLGIFADWEKPYLTMDFGTEANIIRSLGKIAENGHLHQGFKPVHWCTDCGSSLAEAEVEYKDKQSPAIDVKFTISDESVADKFSHPEGHKGEGEIGAVIWTTTPWTLPANRAIAVNAEVEYTLVQCEQAGEKQRLIIASDLVTTCMDRFGFDKYHALGFCKGSELELVQCQHPFYDFTVPVVLGEHVTTDSGTGCVHTAPGHGVEDFVVGKLYDLEVANPVGANGVYLEDTPLLAGQHVFKANASVVELLKEKGALVHHHALDHSYPHCWRHKTPLIFRATPQWFISMDKKGLRQDSLNEIEKTQWIPDWGQRRIESMVEGRPDWCISRQRTWGVPMALFIHQDSGALHPRSIELIEEVALLVEKSGIQAWFDLEAIELIGDDAKEYIKVPDTLDVWFDSGTTHESVIKARDEFDGIADLYLEGSDQHRGWFMSSMISSVAMNGAAPYKQVLTHGFVVDAKGHKMSKSLGNVITPKEITNNLGADILRLWTASVNYTQEITAGDEIFKRQADAYRRIRNTSRFLLSNLTGFEPANHMVAVEDMVALDRWVIDKAARLQEEIINAYDEYEFHVVVHKLMNFCTNELGGFYLDIIKDRQYTAKSDSNARRSCQTAMYLIAEAMTAWMAPILSFTAQEIWEALPLPVSGERDEFVFTGVWFDGLMKQESKQDESTESSDELGNEYWTELLTVRGEVNRALEQARKDKSVGKALEAQVTLFATADLAAKLAKLGDELRFVLITSKATIETVTSAPENALETEVEGLWLTVAPAEGIKCERCWHVTTDIGESEKHPTLCGRCITNIDGEGETRQFA

>sp_Q12X17

MTHDAAIGRLIKARTISDAWYRGLNVIWNHGSLITDERGSQIREFMNLMVVIEDPYSNEIPEDSAWNHERLEEYAKQLITGENAQDFEYTYGQRLRNWDGKVDQIEYVIEKLTNNKTTRRATAVTWVPTIDTKVDEVPCMIIDDFKIRDDTVHLTTLFRSHDFAGAYPANLYGLSKLLEYVADKVGLAPGTITTMSVSAHIYDHDWDKIEKIIKGVQ

>sp_Q12WL0

MTKEYKTIVEVSGPLIFLEKTEPVGYGELVQINLPDGTTKRGQVLDTSADMVVVQVFEGTVGLNEESGVVFSGETIKLPVSKDMLGRILSGAGEPLDGGPRIIPDKRVDINGASMNPYSRMPPEDFIQTGISTIDGTNTLVRGQKLPIFSGSGLPHNEIALQIARQAKVPGSDEPFAVVFAAMGITNEEAQYFMDDFEKTGALERAVVFLNLADDPAVERIVTPRMALTAAEYLAYEHDMHVLVILTDITNYCEALRQMGAAREEVPGRRGYPGYMYTDLASLYERAGVIKGIKGSVTQFSILTMPGDDITHPIPDLSGYITEGQIVVSRELHRKGIYPPINVLPSLSRLMNSGIGEGKTRDDHKAVSDQMYAAYAEGRDLRGLVAIVGKEALSERDRKMLEFADLFEDRFVRQSRDEDRTIDDTLRIAWEILAELPEAQLTRIDNKYLDKYHPAHQKSE

>sp_Q487B0

MSVQQQQVDLRRTFAIISHPDAGKTTITEKVLLFGQALQRAGTVKGKKSGQHAKSDWMEMEKERGISITTSVMQFPYNDCLVNLLDTPGHEDFSEDTYRTLTAVDSCLMVIDVAKGVEARTVKLMEVTRLRDTPIITFMNKMDRDVRDPMEVMDEVEEVLKIKCAAITWPIGMGKEFKGIYHILDDEITLYQSGLGHMIQEKRVIKGLNNPELDKIIGNYADDLREELELVSGASHDFNLEEFLKGELTPVFFGTALGNFGVDHMLDGLTKWAPKPLPRKTDLREVTAEEEKFSGFVFKIQANMDPKHRDRIAFMRICSGKYEKGMKMKQVRLAKDVKIADAVTFMAGDRSNVEEAFAGDIIGLHNHGSIQIGDTFTAGEMMKFSGIPNFAPEMFRRIRLRDPLKAKQLQKGLIQLSEEGAVQVFRPFINNDMIVGAVGVLQFEVVVQRLKTEYKVDAIYEAISVATARWCTCDDERTLEQFKKKSGDYLALDGGNNLTYIAPTMVNLSLAQERNPDIVFHSTREH

>sp_Q12TX1

MAESIEWVEKYRPQSLTDIVGNKKSVVDMREWAQSWLSGTPEKRAIILHGPAGVGKTSAAHALARDLDWETIELNASDQRTAGVIERVAGSASKMSSLTGTTAKRLIILDEADNIHGNADRGGARAIGGIIKNTDQPIVLIANDLYGLTPSVRSLCIELKFNSVQGRSMIPAMKRICVEEKIMCGVGVLEKLAESAGGDLRSAIKDLQAVATGRDEIHIEDIATSERDTKESIFKVLGKIFKSTDPKKALEATYGLDETPENLIHWIDENLPLQYGTEEGTQEDLITGYEYLAKADRYLGRVRKRQSYRLWRYAGALMTCGTVVSKTHVGRGFTKYQPPSFWRKMGQLRAKRDMRDNIASKIADHANKSMRYSRTDLAHLYGRMLEENEYAADVTFDLELSIDEMVYLTGKKKVTKDIQRIHDLAQAKRRSLGRDEGKAFFEKKPKKQTPDKKQMDLTQIINSTPQEDKVEKKETENVPPVKKSASKAKPQKTLFDF

>sp_Q12TG0

MTNRKEIIGDAEKIVIKIGTTSISREDGSLNNEFMDTIASQVSELHRAGKQIILVSSGSIGIGIEILDLGCRPKEIPVRQAAAAVGQGVLMQHWTEAFQKYGLNVAQILLTYDSFTNRLTYLNLRNSISTLLSYGVIPIINENDPICVHEIEATLGDNDKLSAMVASKMEADLLILFTDIDGLYDKNPKRHDDAVLLRTVEEITPTIESYGGNPTSMKGVGGMRTKIDAAKICNISGCYMVIANSNVDDGIRRILDGEELGTLFLTNQFVHKNRIRWIILARSSGSIVVDTGAKEALAKRMSLLPSGVLGVAGTFDRGDIVKLECDGVVFGKGITDYTSEELKAIKGKQTNEIADILGYKNYDHVVQKDNIGLFK

>sp_Q483R6

MSVTAKIIPQEIIRLKRDGKILDEQAINGFVSGLVDGNFSDSQVGAMAMAIFQQGMSIDERVNFTKAMMRSGEVLSWEGFDGPIVDKHSTGGVGDKVSFMLAAIVAACGGYVPMISGRGLGHTGGTADKLESIAGFNVQPSISEFKRIVKDVGVAIISQTDNLAPADKRLYSIRDVTATVESIPLITASILSKKLAAGLDVLVMDVKVGNGAMMNNLDDAKALAQSITSVANGAGVKTQAIITDMNQVLGTSAGNAIEMYETVKYLTGKQREPRLHKIVQALASAMLINTNLASSEKDAREKIDKVLNSGLAAEKFDRMVSALGGPKNFIEKPWDSMKKANVITEVRALQHGYIAQTDTRAIGMSVVGLGGGRTAPTQQVDHSVGFDRILPLGVQVNRGEVIARLHAKDEDSANRAIEQFNNAITYSEESPELPPVIY

>sp_Q12VZ8

MVTIKIECRTIARGVAEGEVLLSEDALSFLGNVDPKTGVVVDPGHAIYGECIRDKILVFPHGKGSTVGSYVIYQLKKNNVSPAAMINIDSEPIVAVGAIISDIPLVDRLDKDPFTIFKNGDRVKVDSTSGFVELMDR

>sp_Q488Z4

MHLNTLSPAPGSHKARKRCGRGIGSGIGKTGGRGHKGQKSRSGGSVRPGFEGGQMPLKQRLPKFGFTSRKSLVRAEVRLHELNLITGDVVDIHALKDAGLITRNIVAVKVMLSGEITRPITLRGIAVTKGAQAAIEAAGGKVEE

>sp_Q482K3

MAVQKSKKSRSRRGMRRSHDAVTPENLSVDPVSGETHRRHHITADGFYKGVKVIAV

>sp_Q12WR3

MARDRRDTYYWRAKDEGYRSRAAYKLFQINEKHEVIKEDDTIVDLGAAPGGWLEVAKKISGGKIVGVDLRRIKEIEGVETIKGDITSDETIKKIIELVGEGGADVVICDAAPNLSGNWSLDHARSIDLTTSALECAKKILKPKGHFIVKVFQGDMFKEYMDKVRESFTYTRAFSPKASRPESAEIYVIGKKLLTAPLKIDDKFDVTIKKIGAKGNGIAFVEDFVVFMQDEVKKGENVRIKIVDVKPEFAFAIVIGRYDEELNEKNEE

>sp_Q47UV8

MSISKDDILNAVAEMSVMDVVALIEAMEEKFGVSASAAVAAAGPAEAADEQTEFNVVMTSFGEKKVAVIKAVRGATGLGLKEAKDLVESLGVVKEGVEKAEAEELKKTLEEAGASVEIK

>sp_Q47Z13

MKRQKRDRQSRAHTRGYQAGISGRSKEHCPYQIDAIKSQWLGGWREAIEDKQQGLFK

>sp_Q487Z2

MSNQRIRIRLKAFDHRLIDQSTAEIVDTAKRTGAQVRGPIPLPTRKERYTILTSPHVNKDARDQYEIRTHKRMIDIVEPTEKTVDALMRLDLAAGVDVQISLG

>sp_Q485G7

MIDEIIEDAQDRMGKSIEALKTSLTKIRTGRAHASLLDNIVVEYYGMDTPLNQVGNVSVPDARNLSITVFDKSMISAVEKAIMKSDLGLNPQSNGTLIRIPLPPLTEERRKDLVKVVRGEAEGGKVAIRNIRRDANSDFKSLLKEKEISEDDHHQAEDSIQKITDVFVKQVDEVLVKKEAELMEI

>sp_Q9S0R0

MAKSPSRSPRKRVRKQVADGMAHIHASFNNTIITITDRQGNALSWATSGGSGFRGSRKSTPFAAQVAAERAGVAAQDYGVKNLEVFVKGPGPGRESAIRALNSVGYKITNITDVTPIPHNGCRPPKKRRV

>sp_Q47UW1

MATINQLVRKPRVRQVTKSNVPALQACPQRRGVCTRVYTTTPKKPNSALRKVARVRLTNGFEVTSYIGGEGHNLQEHSVILIRGGRVKDLPGVRYHTVRGALDCSGVSDRRQGRSKYGAKRPKS

>sp_Q47XL5

MLQIYNTLSRQKETFTPINAGKVGLYVCGCTVYDLCHIGHGRTYISFDNIARYLRFSGYDVNYVRNITDVEDKIINRANENNETTEALTERTIAAMHQDFDSLNMARPDLEPRVTTHMNEIIAMIETLVTKEHAYVAGANAASTGQGDVLFDVSSYNDYGKLSGQNLEQLQSGSRVEVDQNKNNPLDFVLWKSAKPGEPSWSSPWGEGRPGWHIECSAMNAKELGHHFDIHGGGSDLTFPHHENEIAQSCCALNTPYVNYWMHTGMVQVDQEKMSKSLGNFFTIRDVLAQYDAETVRFFLTTGHYRSQLNYSTDNLTQARASVERIYTSLRDVNIESDYVINKDSSFVKQFCQAMDDDFNTPQALAVLFEISKELNVAKAAANNALAIDLASTLVALGEIVGLLQLDPAAFLQGDNDNDEVAIIEALIVQRNQARIDKDWAMADDARDKLNAMNIVLEDSAGKTTWRKA

>sp_Q47VQ9

MSVNHGQGNKDLAKTLLVMAGGTGGHIFPGIAVADELKAQGWKIHWLGTADRMEAQIVPMHGYDISFINISGLRGKNLLTTLVMPFKLLRSLFQARRVIKTVKPDVVIGMGGYASAPGGLAAWLSKIPLIVHEQNAAAGLSNRLLARIANKVCCAFPNAFVSGIDVEVVGNPLRASIGQQALVSENIDQSHEGSKNILVVGGSLGAQVLNKVMPDSFKDLSESDEKYCIWHQTGDNNQALVTASYKQEYIDTGKVRVTEFITDIAAAYQWADIVICRAGALTVSELAMAATPAIFVPLPHAVDDHQTKNALYLVKRDAAKLLPQAELNNESITSLIIELFDQPQTLADMAKASLSAATSDASQKVAKLCQQLSISNGAKLRNNEEKI

>sp_Q12W27

MELKILEKSDDEMKMEIAGESHTLLNMLKIILLEDERVHTASYDMKHVTISEPVLFIKTENADPIDVVKAAVAKLITECEEFVTVFNKAVE

>sp_Q47XW2

MKFSPLVQELIDSLKCLPGVGAKSAQRMAFQLLERNRRGGSKLANTLAKAMTDIGHCQQCRNFTEEALCEICQSPKRQLSTTLCIVETPGDVIAIEQTGEFFGKYFVLMGHLSPIDGIGPDDLGLDILAKQFATGQFSEVILATNPTVEGEATAHFIAELAQQHQVNISRIAHGVPVGGELEYVDGNTLSHALSGRKSYQI

>sp_Q12ZT8

MSNPMRNPKVEKVVVHMGVGESGQHLVDAEGILETITGQTVVRSYAKRTLPAFTIKKGEPIGCKVTLRGEAAEGFLETSLGIVEKRLNESQFDIFGNVSFGVEEHTDYPGMRYDPNIGIFGMDITVVVNRPGYRVSKRRIAKRKIPTSHKITKEDTISFFKDKYAVEVE

>sp_Q483C7

MRSLYCGEVNESHIGQEITLCGWVNKRRDLGAVIFLDLRDREGLVQVVYDPDLPEVIKKANTLRNEFCVQIKGKVRARPEGQVNKGMKTGGIEVLGLELTILNKSAPLPLDSNQVNSEELRLKYRYLDLRRVEMTERLRFRAKVTSAVRSSLESQGFLDIETPILTAATPEGARDYLVPSRTHKGQFFALPQSPQLFKQLLMMSGMERYYQIVKCFRDEDLRADRQPEFTQIDIETSFMSSDQVMEVTEKMIRELFQELLDVDLGEFPRMPYSEAMTRFGSDKPDLRNPLELIDVDDILKDVEFKVFSGPANDENGRVAVICLPQGAAKFSRKGLDELTKFVGIYGAKGMPWLKVNDIDAALSTGVEGLQSPILKFLSSDEAIALLKRTNAKTGDIIFFGADQYNVVTESLGALRLKLGEELDLLQGEWKPLWVVDFPMFEEVDGHMHAIHHPFTAPTNLTAEQLEANPVGALSDAYDMVLNGCELGGGSVRIHNQDMQAAVFRILGISDEEAEEKFGFLLEALQYGAPPHAGLAFGLDRLVMLMTGASSIRDVMAFPKTNTAACPLTNAPGKANPEQLRELGVAVLEVKKAEENKDEEQA

>sp_P0CW89

METIGRGTWIDKLAHELVEREEALGRDTEMINVESGLGASGIPHMGSLGDAVRAYGVGLAVGDMGHSFRLIAYFDDLDGLRKVPEGMPSSLEEHIARPVSAIPDPYGCHDSYGMHMSGLLLEGLDALGIEYDFRRARDTYRDGLLAEQIHRILSNSSVIGEKIAEMVGQEKFRSSLPYFAVCEQCGKMYTAESVEYLADSRKVRYRCGDAEVGGRKIAGCGHEGEADTGGAGGKLAWKVEFAARWQAFDVRFEAYGKDIMDSVRINDWVSDEILSSPHPHHTRYEMFLDKGGKKISKSSGNVVTPQKWLRYGTPQSILLLMYKRITGARELGLEDVPSLMDEYGDLQREYFAGGGRGGKAREAKNRGLFEYTNLLEAQEGPRPHAGYRLLVELSRLFRENRTERVTKKLVEYGVIDGPSPGIERLIALAGNYADDMYSAERTEVELDGATRGALSELAEMLGSAPEGGLQDVIYGVAKSHGVPPRDFFKALYRIILDASSGPRIGPFIEDIGREKVAGMIRGRL

>sp_Q47WT5

MTDKANNTPVAQDENKLIAERRVKLEKIRSNCSANGFPNDFNREHLAADIQAEHGEKTKEELEELQVTYAIAGRVMAKRGPFLVIQDSSGRIQGYAEKTVQKEIRAKWGSLDIGDIVGIKGILHKSGKGDLYVNMDHYSLLTKSLRPLPEKFHGLSDQETKYRQRYIDLIINEDTRNTFKMRSKIVAGIRNFLTQRDFMEVETPMLQIIPGGATAKPFMTHHNTFDLDMYLRIAPELNLKRLVVGGFDRVFEINRSFRNEGISTRHNPEFTMIEFYQAYADYHDLMNTTEEMLRTIAQDVLGTTTIRNTVKNSEGEVVEEKFYDLGKPFVRLSMVDAILQYGKDHRGAEQLDEAALRDPENNFDAIKAMAKAVGVKENSASKVWGPGKYICEIFEEVAEHLLDQPTFITEYPWEVSPLARRNDENSFITDRFEFFVGGRELANGFSELNDAEDQAERFQKQVAEKDAGDDEAMHYDADYINALEYGLPPTAGEGIGIDRLVMLFTDSPTIKDVILFPHMRPEAE

>sp_Q47W35

MRILGIETSCDETGIAIYDDGLGDSPEGILAHRLYSQIAVHADYGGVVPELASRDHVRKTIPLIKEVLADANLTPKDLDGVAYTAGPGLVGALLVGCSIGRSLAYGWELPAVPVHHMEGHLLAPMLEDDVPEFPFVALLVSGGHTMLVRVDAIGEYKLLGESVDDAAGEAFDKTAKLLGLDYPGGPALSKMAESGEAGRFKLPRPMTDRPGLDFSFSGLKTAAGTLVRKECLNLSDSDLKQTHADIANAFQQAVVDTLAIKCKRALQQEKLSRLVIAGGVSANTALREQLAITTKKLGGSVFYPRPEFCTDNGAMIAYAGLQRLKAGTDADLTFKANPRWALDSLPPVK

>sp_Q48AU1

MSEKNVVEAFKNGGIIAYPTEAVFGLGCDPDNKEALKRLLKLKQRSPEKGLILLAGSYSQLLPYIDDSKIPQDKRLTLLSRWPDGITQLVPKNNNISSLLSGSFDTIAVRITSQPDVVALCQQTNKPIVSTSANLSGQEPAKTWQSLDLVLSKQVDFILKGVTLGRTSPSKIIDALTGKIIRN

>sp_Q12Y24

MKIALLSRSRKLYSSRRLIEAAEERGHEIQVIDVLRAYMNITSHKPSIHYKGEELEGFDVVIPRIGASVTFYGASVLRQFEMMGVYPLNESVAITRSRDKLRSLQLLSRKGIGMPVTGYASKPDDIKDVIKMVGGAPLVVKLLEGTQGIGVVLAETQKAAESVIEGFMGVKANILVQEYIKEANGADIRCFVIGGKVVASMKRQAPNGEFRSNLHRGGSAEVIRITPEERSTAVSAAKIMGLNVAGVDLLRSNHGPVVMEVNSSPGLRGIETATGKDIAGMIIEYIEKSGKVGKTKTRGKG

>sp_Q12WK9

MGAKEVKPTRSELIELKKKIKLSEGGHKLLKMKRDGLILEFFDILSKAKDVRSELDAAYEKANVKIGIAESVEGRITIKSTAFAMKDAPQIVLESHNIMGVVVPKIESSSVRKPINKRGYGLLGTSSYIDEAVDSYEELVEKIILAAEIETTMKKLLDDIEKTKRRVNALEFKVIPELTEAMVFIRLRLEEMERENTFRLKRIKKA

>sp_O74045

MLDPRTRPRVVNVVSTSDLVQRVSAKKMAAMPCCMYDEAVYGGRCGYIKTPGMQGRVTVFISGKMISVGARSVRASFGQLHEARLHLVRNGAAGDCKIRPVVRNIVATVDAGRNVPIDRISSRMPGAVYDPGSFPGMILKGLDSCSFLVFASGKMVIAGAKSPDELRRSSFDLLTRLNNAGA

>sp_Q482F9

MNDPKVVVALDFDRKQDALSFVDKIQPTDARLKVGKEMFTYFGPEFVKQLTGKGFDVFLDLKFHDIPNTVAKAVTAAADLGVWMVNVHASGGSQMMTKAKQALDNYGNDAPLLIAVTVLTSMGQEDLHGLGINKTPAEQVNFLANLTKQSGLDGVVCSAWEAEQLKADLGKEFKLITPGIRPAGSAQDDQQRIMTPKQAIDVGVDYLVIGRPITKAVDPQLVLQQINGTIR

>sp_Q47ZY2

MKKLVVIYSGGMDSFTALNKAVKEGFDVYALSFDYGQKHNKELIYAQNVCNELNVPHKILDIKSISTLFTSSSLVSDDINVPDGHYEADNMKSTVVPNRNMILISLAIGYAVDIEAEGVWYGAHSGDHLIYPDCRPEFVKVMDQASKVANFEPVYVHAPYLNTDKIGILKDGIKMGLDYSKTWTCYQGKEKACGTCGSCVERLEAFQANNIDDPVQYSI

>sp_Q47WV1

MSKILEMLEQEQMKTDLPAFAPGDTVVVQVKVTEADKSRLQAFEGVVIAVKSRGLHSAFTVRKISNGVGVERVFQTHSPIVDSIEVKRRGDVRQAKLYYLRELSGRKARIKEKLAKK

>sp_Q47U32

MKRTFQPSVLKRKRNHGFRARMATKNGRAVIARRRAKGRARLSA

>sp_Q488A2

MSETKIRTLQGVVVSNKMDKSIVVLIERRVKHPMYGKYMTRSTKLKAHDETNVCNEGDLVTITEVAPISKSKNWKLVDVITKA

>sp_Q12ZU5

MAVEKKFVQDGYVKASMDEYFAKQLSRAGYGGMDINRTPMGTQITVYAEKPGMVIGKAGKVIRKLTRDVDRLYDLDNPQIDAQEVKRPELNAQMMASRLASSIERGWYFRKAGHNTMRAVMNAGALGCEIVISGKLTGSRSRVEKMVNGYIKHAGKPVDDIVDDGFATAVKKLGTLGCKVRIIHPDAVLPDSYRLKNAEELGSLVSTPVEEEKSAGIEELVEVEAKGEVAEAEEAVVEETAKAEAETVEETVEEVAASEVEADMITGESVTEEGEERREVNGVWQHKHGGHDYWHPTGRMHRES

>sp_Q12UP8

MAELHHSEHIPQWKKDEIEEIKSLIESYPLFGVIGIEGIPAKQLQSMRRDLKDFAVLKVCRNSLTRRALDQSSDDVKKMDDYIDVQTALIFTKQNPFKLYKLLEKSKTPSPIKAGMVATSDIIVEKGPTSFPPGPILGDMQGAGIPAAIDGGKVVIKETKAVAKAGEVVSQKLAAMLTRLEIYPLEVGLDLRAVLEEGSIFTPDVLAIDEEQIFSNFVQAAQQAFNMSVNAAYPTAMNINTLLAKAASDSRNVAVNATVYEPGIMDILLGKAYSKMMAIASAASSNDDALDDELKEALGAASSAVSAVEEVVEEQEEVKEEEEEESDMASGLGALFG

>sp_Q47Y88

MTDLLTLEAEVRTDLGKGASRRLRHANKVPAILYGENEEPISLTLEHKNVFRAQQEEAFYSQVLTLNIAGKPVECLIKDMQRHPFKQVVMHLDFLRIDAKHAVHANAPIHFLNEDEAAKTGANISHHMNEIAITCLPKDLPEFISIDLAGLELGQTIHLSDVTFPAGVTSDELAKGEDHDLAVVSANAPKAAKVSTDDEAAAPAEEAPAAE

>sp_Q12Z95

MEQRKCSFCGELLEPGTGLLFAKRDGSTYYFCSSKCKGNFDLGRLPRRTVWTEQGRIYLKKA

>sp_Q9S0R1

MARIAGINIPDQKHTVIALTGIFGIGRTRARAICAATSIAEDAKIKELSEAQIDILREAVAEYTVEGDLRREVSMNIKRLMDLGCYRGIRHRRSLPLRGQRTKTNARTRKGPRKPIRK

>sp_Q482T7

MAKRRKGRQVNGVLLLDKPHGLSSNHALQTVKRIYFAQKAGHTGALDPLATGMLPICLGEGTKFSQYLLDTDKTYQVTAKLGIRTTTSDAGGEVVSEKTVDVSSEQLAKALDSFRGTTKQVPSMYSALKHQGQPLYKYAREGIEVPREARDITVFNLELLRFEHDEVELNIHVSKGTYIRTIVDDLGELLGCGAHVAHLRRSAVGNYPVEKMITLPELEALLEQANADEITPSDVLDPLLLPMNSAVDGMHCVYVDDMSANFLRHGNPVQAYNQPEAGSVQVYLGEDENDADAEFIGVGFINDDGLVAPKRIVVLEQY

>sp_Q487E7

MLIEQAYLHGKPESSGLLRSQISDFQVFEELPFLPCGEGEHLFVHIRKTGANTLFVARELAKYFEVKEQLVSYAGLKDRFAVTEQWFGIHVPGKQEYNLDDLNIEGVEVLSYKRHNKKLRTGALTGNRFELILREVTAIKAFTERWQKIVEQGVPNYFGEQRFGIGGGNIERALSLFSGQKVKDKKKRGMYLSAARSHIFNSVLNERIQQQCFDKVAVGDVLMLAGTQSVFHLDEVDSAIQQRFTDKDVDITAPMWGAGELMTSNAPQVLEQEVATKNLEFCEGLPRFGLKQERRRIRLTVSDTDIELLSAEEDSAQEESNAVKISFFLPAGCYATTVLRELLNYQDMTTRIDKRETAVNSNQQDSA

>sp_Q12ZU3

MEALPSNLIFHELIGLYAEVFESTNPKLINICGRVIDETRNMLIIETEDTHEKMVPKNGTTFVFHLPSSSADHDQRVKIFGTLLLSQPENRVKNIRKIRMR

>sp_Q12W43

MVRKPASMYRNVKSRSFTRRKYMGGVPGSQVIHFDMGNKTADFPVKITLLADERCQIRHTALEAARITANRAMTTAAGRSGFHMKLRVYPHEVLRENKQATGAGADRVSSGMRAAYGKNVSTAARVSARQKIFTISVNKEHFIIAKDALRRAGQKLPTPVTIVVDQGQELVR

>sp_Q47YB4

MTSTNKTLTQAILNFSKSYSQQHVEQFGHLPTVEHDEQWPSPCDLGSHDTSHHYWQAVAMESVQLADNKEEALSFENVESALNIELHPDIKIYFTTIFSGDIEAQSDDGELSLLFAWNKDDFERLQENIIGHILMKQKLKQVETVFFAVTDEEDMIISVDNSSGEVWVEQVGCKPHKKLSDSLAEFISQLTHKNVVSEKS

>sp_Q47WR3

MTNSSSQLAQLKQMTTVVADTGDIEAIAKFQPQDATTNPSLLLKAASLPNYQGLVKDSVAWAKTQSDNAEQQVIDAADKISVLIGLEILKIVPGRISTEVDARLSFDTSASITKAHKLIAMYNEAGISNDRILIKLASTWEGIKAAEQLEQEGINCNLTLLFSFAQARACAEAGAYLISPFVGRILDWYKKDTGRNDYASNEDPGVVSVTSIFNYYKLQGFNTVVMGASFRNIGEILELAGCDRLTISPQLMEELANSTDTVIQKLTACEATAEKEAALSQAEFRWQMNEDPMATEKLAEGIRNFTIDQVKLEKQLTDLL

>sp_Q12ZL9

MKKQIDKDYHLHIFHAKQCDPKKCTGKKMARFELARIFDKVQKIPRGSILLDPMAKQALSPADKHEQNITVLDCSWETVEEVFPHLMRLHLQHRALPYLVATNPVNFGRPFKLTSVEAFAAALYILGNKKQAEKILSKFNWGHVFLDMNKEPLEDYSKAKDSNEIIKIQSEYM

>sp_Q47VU1

MLWNNKTSAKNTSSTDKGQVTESYAQQYLSKQGLRFIERNFHSRQGEIDLIMLDGDTYVFVEVKYRKSKGFGGAIAAISASKQNKVKHCITFYLHQNGLNEYNTPCRVDVVALEGDITQPQVTWLKNAF

>sp_Q47WR5

MLLVVSPAKKLDFESPLATEKFSQPGLLEQSQLLIDDCIKLSPSEIASLMKLSDKLAGLNAARFGQWSTPFTQDNARQAILSFNGDVYTGLDAQSFSDEDFEFAQKNFRILSGLYGLLKPLDLMQAYRLEMGCKLGNSRGDNLYQFWGEIITDELNKTLSELDDDVLINLASTEYFKSVKKKSLNATIITPTFKDWKNGQYKIISFFAKKARGLMARYIIQNKLTSVEQIKTFDLAGYQYNEAMSKDNDWVFTRKES

>sp_Q12V98

MADQSSHQKYEFKKKLESLRGKKGRGTELISLYIPPDKQISDVVSQLRNEHSQASNIKSKLTKTNVQGAIDSIMSRLRYGTVPENGIVYFTGAVDVGANKTNMETTIVEPPQPIITYRYHCDSSFFLTPLEDMLKEAKTYGLLVLDRREATVGLLTGKQIEAFRNLTSTVPGKQRKGGQSAHRFQQLRLIAIHDFYKRIGDAASEVFLAVEQKDFEGVLIGGPSPTKEEFEAGNFFHHEIEKKVLDLFDVAYTDESGLSELVNAASERLEDLDLMVEKKLMQQFFRELVSDSGKATYGEDNVRENLIIGAVDILLISEDLRAVRETVKCTSCDYEQKTSREFKPGDSSSPTGNCPKCGSYLEITEKVDVVDQLSEICDQMGTRVEFISTDFEEGSQLLKAFGGVVAILRFNTGI

>sp_Q487Z8

MQAIAIHKFARGSAQKARLVADQIRGVNVEKALEILTFSNKKAAELVKKVLNSAIANAEHNEGADIDELFVKTILIDDGPTMKRIMPRAKGRADRIIKRTSHITVIVSDS

>sp_Q47YA5

MTSIVLFCRPGFEKECGAEIQEKAAWNEMYGYLELKINQGLVFFHLHESAHGEALMNKLPLKRLIFARQWFVTVTDKIDLPDYNRVEAITEALGNDWQYSDLRMEMADDNDGKSLSKFCRKLSVPLRQALRKNKVLTQKGNNSADDPEGAILHALFLSGQEVILGFSLARNSSPHVMGIPRLKFPSASPSRSTLKLDEAFLHFIPRDEWDERLTSGMNAVDLGSAPGGWTYQLVRRGMMVTAIDNGLMAESLMETGQVKHKMMDGFKYVPLKQNVYWLVCDMIEKPQRVAKLMSEWLLHGHCKEAMFNLKLPMKGRYQQVTNDLQTIKDAFTKHNVKYELYAKHLYYDREEVTVHARLLSPAPLRAKE

>sp_Q489U1

MRHYEIVFMVHPDQSEQVSGMIQRYTDLINAAEGKIHRLEDWGRRQLAYPINKLHKAHYVLMNIEAPQVVIDELETAFRYNDVVIRNMIMRTKDAVTEASPMAAAKEERRDDRREVKKDVAAAPVEAKEDSVEEKSEEAASEE

>sp_Q12UF1

MSSEMCPVCGLPSELCICEEVAKEQQRITVKVNRRRYGKEVTVVEGFDANEIDLHELSTYLKSKFACGGTVKGSAVELQGNHLSRMKAVLVKKGFSPEQIKD

>sp_Q485F0

MTSEKPQNESFIQKIKSTSLVSQIIVAIILASLLAVISPESAKAFGMLGSLFVNALKAVAPILVLVLVTSAIANQKMDSSAELKPIVGLYFLGTLSAALVAVLLSFAFPTELTLDLAGATANPPQKLTEVLATLAFKVVENPVTAVASGNFIAVLAWGLGLGFSLKHASESSKGMVHDLSEAISSVVRIVIRFAPLGIFGLVANTIATTGFSALGEYSHLVAVLLSAMLIIALLVNPLIVFIITKKNPYPLVFTCLKESGITAFFTRSSAANIPVNMALCKKLDLHEDTYSVSIPLGATINMAGAAITITVLTLAAAYSLNIEVSFATAILLSVVASISACGASGVAGGSLLLIPLACSLFGINNDVAMQVVAIGFIIGVIQDSAETALNSSTDVVFSAAASHHITKE

>sp_Q12YV9

MTDSNDPVVRGYLLQLIGEEGIEMIENMPEGEVTDEQIAEASGVMLNIVRRTLFIMNENNLAVCRRERDSSSGWLTYLWQLDLSDIESHLVKEKKRITKNLEIRYNFEVDSVFYTCPEGCVRFEFKEASKCEFMCPACGEDMMFEDNSVMVKKLRDRLDALEASS

>sp_Q47YA8

MSAHLILIDALNLIRRVYAVQERPFIQIKQDHDDELSASTLKQVLFNTQNTCVNALIKIIDQHQPTHALTVFDSQEPCWRYQLFEGYKKGRKKMPDHLANKLIDIQDAFMEQGVDSLTSDEDEADDLIATLAVKMALHGQKVTIISTDKGFLPLLSPNIHIYDYFNRRYLDEEHVQSKFSVKTSQLIDFWTLTGDNTNKIEGVSGIGQVTAAKLLNQYGSLKAILEATDLKDSLAEKLTQSLEQMDLARKLLTLKQDIPLGFNLKDIRLTTSSSAHEINANINLTTDDKS

>sp_Q482U1

MAKFEQKLTDLLRPAVEETGKTLHGIEYISAGNNSVLRLFIDHENGINVDDCAEVSRQVGAILDVEDPISSEYSLEVSSPGVDRPLFELAHFQEVIGETINVKISMPLNGRRKFKGPLVAIENDTLIVEVDSIDYELAISNIDKANLVAKF

>sp_Q488Z5

MRMSKTVKVTQLKSSIGRLPKHRATLKGLGLRRINHTVELEDTPSVRGMINKVYYMVKVED

>sp_Q12VH3

MARFPEAEERILNKKICMKCSARNAVRATRCRKCGYTNLRVKNKDVRRG

>sp_Q483C5

MIGRLRGMLVEKNSPEILIECAGVGYEVTMPMTSIYALPELEQQATIYTHFVVREDAQLLYGFANKVERKLFRLLIKVNGVGPKLALAILSNMSADQFVSCVRHDDISAIVKIPGVGKKTAERLLIEMRDRLKDWQAQQIHLVSDDGVIPEQLSAELSQETTFVNDNKGDAINALLSLGYKQVQADKAVKSVYNRGMSSENIIRDALKSMI

>sp_Q47VN0

MSCPMSLSNPSQILLRNSELLVAKVPLFINLPEDGFIEAYTEIHKPDTIHCFNTNFIDYQAITKKHCSPTKTKNVKATFASTYQTTSCHDLVIIAFPKSKAELNFTLAMITHCINDETKIILVGEKKGGIQSAAKLTQHIFSCCQKVDAARHCLLFVGLFQPERLSDVFNLQDWFKKYQITVEGIELTIASLPGVFSQQKLDVGTALLLSNLPSKMTGKVLDFGCGAGVISCFIGKKFSGTNLSLLDVSALALTSAQESLALNGLSGNVFPSNSLSDVNEHYQHVVSNPPFHQGVKTHYQASEDFLAGINKKLNKQGNITIVANSFLRYQPIMETHIGNTRVITKDKGFTIYRAQLS

>sp_Q483C4

MIEADRLIEPIASVEDERVDRAIRPKMLQDYTGQQHVKAQMEIFIPAAKNRGEPLDHLLIFGPPGLGKTTLANIVANEMGVNIRTTSGPVLEKAGDLAALLTNLEENDILFIDEIHRLSAVVEEILYPAMEDYQLDIMIGEGPAARSIKLDLPPFTLIGATTRAGALTSPLRDRFGIVQRLEFYNVADLSTIVSRSAHFLNLTIDEEGAFEVARRSRGTPRIANRLLRRVRDYADIKSHGVVNQQTAAAALDMLEVDSEGFDIMDRKLLHAIIDKFMGGPVGLDNVAAAIGEERETIEDVIEPFLIQQGFLQRTPRGRIATDRAYQHFGITKDQTKD

>sp_Q485H8

MTQDEMKNAAAIKALEFIENDTIVGVGTGSTVNFFIEALASMKDKIAGAVSSSEESTKRLKAHGIEVFDLNSVDVLDVYVDGADEITRHMSMIKGGGAALTREKIVAAVAKKFICIADDSKQVKVLGNFPLPVEVIPMARSYVARELVKLGGDPVYRQGVTTDNGNVILDVYNLEILDPKALETQINAIVGVVTNGLFALRGADILVLGSKDGIQVIQ

>sp_Q12ZC2

MAERSFYDLSIHYVPDGKNTAEELISMAKHLGFAGIGLSNHSTAEGPLKSDGTEGFDIFRTVELVASNPSKLHGLVGKYRNKVDVLAVHGGDEGINRAAVENSNVDILIHPFTPKGSGLNHVLAKSASENNVAIAFDIDSLIMSRGGRRVHSLSHFRDYLALSRKYDVPMLLTSNALSIFGLRAPREIIALAALFGMEKDEAIMALSETPLKIVQKKRHDKNYVCEGVEIFEPSPSVLGDE

>sp_Q12WS3

MSKFSLLDHELIPHHEIMDEDDLKTVLTHYNVEREQLPKLKVTDPIALEIGAEPGDVVKVIRKSQTAGEALYYRYVIG

>sp_Q12UQ2

MAESILKSAKINRNVGQVLKSYLRVLKLSKKPSREEFLMISKVAGAGILVIGFVGFLIYVLLTEVPKWV

>sp_Q47V69

MNNDNIAQLIADFPLIEKMVELKEVSWFNPNITSLQEGLPYVGLDNNNIQDASDRLARFAPYMVKAFPETAITNGIIESDVVEISAMKSQLEQQYEVEIQGKLLLKKDSHLPISGSIKARGGIYEVLTHAEQLAIKAEVLELSDDYSKLFTEEFKSFFSQYSIAVGSTGNLGMSIGIMSAKLGFSVSVHMSADARQWKKEKLRAHGVNVVEYNEDYSVAVEQGRQEAENDPKCFFIDDENSQTLFLGYSVAGERLKQQFDAMHIVVDENNPLFVYLPCGVGGGPGGVAFGLKMAFGDNVHCIFAEPTHSPCMLLGILTGLHDGIAVQDIGIDNITAADGLAVGRASGFVGRAMERLLDGFYTISDQRMYDLLGLLNKVEGIQLEPSALAGMLGPIVVTNNDEYLKRINMSNEKLVNATHLIWATGGGMVPAIEMEKYLSKAGS

>sp_Q47W67

MILQVQPAKLIKRYKRFLADIELNCGEETTIHCANTGAMKGCAEPDDTVWYTTSTNTKRKYPFSWEITQSQDDHFICVNTLRANQLVEEALHLDLIKELSGFNELKREVKYGNENSRVDFLATYNNAPDTYIEVKSVTLLESGHGYFPDAVTTRGQKHLRELMDMVAQGHKAVLLFAVLHSGINDISAASHVDPIYAKLLKEARLAGVEIIAYKAGFSLQLGELDVKLVEKIPFIER

>sp_Q482U6

MAFWIASSPHNHNHTKTPNLMRLVIYATLPGVLAQWYFFGWGNLIHIGLAMTTAIICEFTVLSLRKKPISHELFDGSALLTALLLGICLPALAPWWITVIGTMFAIVIVKQLYGGLGHNPFNPAMAGYVMLLVSFPLQMTLWQPPLTLVAVDLNFTNTLTTILTGFTVEGYSVEQVRTSIDGITMATPLDTLKTNTGLGLTVLESIKNPIFGENLALGWEWVNAGFLLGGLFLISRKAIAWQTPISFLLSLFICSFIGYSISPDSSASTMFHWFSGATMLGAFFILTDPVTGATSNKGRIIVGLLAGLLVYLIRTSGGYPDGVAFAILLCNMSAPLIDQYTRPRTYGHLKGDK

>sp_Q48AG4

MVATQNKNLKPNRKHQILESLALMLQNCPGQRITTAKLAAEVGFSEAALYRHFPSKARMFEGLIDFIEESIFSRINLILADHKEALVRCHHILHVLVVFAERNPGMCRILAGDALMGENERLRGRVHQFFEKLESQFKQVLRERKLREGKTFTINEAALASLLVSFAEGKISQYVRSGYSKKPSTDFNEQWQFLMANK

>sp_Q482T6

MSLNATEKAAIVAEYAQSEGDTGSPEVQVALLTTQINHLQGHFKAHIHDHHSRRGLLRMVAQRRKLLDYLKGKNVDRYGALIGKLGLRR

>sp_Q12TT2

MAVKDYYKVNGESIERLRQFCPRCGDGVYLADHKDRLTCGKCGYTEFKK

>sp_Q48AK7

MSPIIASEHLSNIALGYVDSVDSEYAKRLAKKWQFNYLGHVAAASKQPKLEFMLQMHHHALELCKLDEPKLGAIKVDFVEGAVAHRRKFGGGRGQDIAKAVGLKHGFKPHVLDATAGLGRDAFVLASLGCKLTLLERMPVVAALLDDGIERAKLNHEVADIANNMQLIHGSSLEEVDLAIEPDVVYLDPMYPHREKSAAVKKEMRIFQSLVGEDLDADDLLEPALALAKYRVVVKRPSYAPPLANKKPSMSINMKKNRFDVYVKQAIPKPIT

>sp_Q488J6

MSINTSGKITSATERADSADFRTIIRNKIPLMDVRAPVEFGKGAFDLSINYPLMNNDERADVGICYKQNGQEAAIKLGNALVSGQSKARRLTQWQTFARENPQGYLYCFRGGLRSQTVQRWLKESGINYPLITGGYKALRQFLLNELEQLALHPLTILAGNTGSGKTPFLAHCANSLDLEGAAGHRGSSFGSFVTPQSNQINFENKLAEQYIRGDFSLEQRLVLEDEGRLIGSVHLPESLRNAMTLAPLVVIEESLEYRLEQIYQEYIIKMTAQYQAAYGVEQGFTAFSEYLEHGLFKVRKRLGTQRYGQLKEIQSTALTQQNKGQPQYHLDWLKPLLSEYYDPMYQYQLGLKSDRVIFRGNNSECLAFLRD

>sp_Q12UB6

MEKQLLDVLVELNGVWLSRSGLLHGIRNFEITTKHIHIETDCGARFTVRNSRSSRSARSLRHNKYRKPCKRCRPADEQIDRFVKKTFKEKRQTVSVFSSPKKHVPKKPKVAVIKSFSISTPSPKEASVSNSIPTPSISVVKDEVKVPEVKYTPSQIERLKTLMSPDDKIPIQDELPEFKVLEKELIQRRRDDLKKMYEEDREDRLGKLERDITEFFVDRGFLEIKSPIMIPFEYIERMGIDKDDHLNKQIFRVDESMCLRPMLAPCLYNYLRKLDKVLPDPIRIFEIGPCYRKESDGSSHLEEFTMVNFCQMGSGCTRENMEALIDEFLEHLGIEYEIEADNCMVYGDTIDIMHGDLELSSAVVGPIPLDREWGVNKPWMGAGFGLERLLKVRHNYTNIRRASRSELYYNGINTNL

>sp_Q486R8

MWFKNLYFFAFTRPFEWSEQDLEKHLSEHLFTPLGSTEISHFGWINALGKHGDTSVHSANGNFLICARKEEKMLPASVIKDMIEEKVNLLEQEQGRGATKKEKEQFKEDITFELLPRAFSRITDTHAYISPVNNIIVINSSSRGKAEDLLALLRKVLGTLPVSSLEPDVCADETMTDWLNEKSLGGKFTLGMEAEFNALGDDGAVVRVKNQDLLSEEIKSHLDADKYVVKVALEWDESLSFILCDDLAIKRVKFFDVIHEQNDDIDSDDVIAKLDADFALMSGELNRFIIELLAEFSMKTTDLLEKD

>sp_Q48AL2

MLALRSFFYLLLKFPLKLLVRCKIITDSQNITDQPNQPIFYIVRHQSASDLLALQSACKKQNLPDPLGKVTINGESFNRTLCLAKSTPLCSWRKSSKTTATAQGLALLNQHVIDENIDAKLIPANLIWGRTPTKERKNLNIGTLLADQESPNWLRKFFIVLFLGRDTLVRFSEAFSLRNISDNHGSDEAAAHKFLRVARFHFHRQTIAAKGPRLMHRKQMFTALFANPSVKRIISDEAKNKKVSEAEIKKKALVMMNEIAGDYSVSWLRFGEIILHWLWKRLYSAIKVSNAKVLRKLAQDGHEIIYVPCHRSHMDYLLLSYVILQEGLVMPRIAAGINLNFWPAGTIFRKGGAFFIRRSFGGNRLYSTIFREYLGLLFERGYGVKYYTEGGRSRTGRVLAPKTGMLAMTIQSLLRGIDRPLTLVPVYLGYEHVMEVGTYHKELSGSEKKGESMFGVLKAIKSLRNYGNGYVNFGEPMNINEFLNKQVPDWKDSIDPIDPQKPSWLTPTVNVLADQVMENINKSAALNGVALIALILHASKNKALSKLELETQLDFFLNIQRQAPFSEQLTIPEETGAELLTHVISLNKVTITEDSFGSLVSLSETANTEMRYYRNNILHTYVVPALVCRLLDKHSKINQDELVIKVQNVTALLKEDLYLYQDSTHVEQQTLRVLTVLKEMEIAKQTKAGFWSLSDDVGLLSQVHAMAECIDESLQRLAIITSLTSRLAPLSKRDLETKVVAIAKRLSVLNNINAPEFIDKRAQSTLIATIREQGYIDLDDDGLLIASSTMAEIKATVINLVDIEVLQSIAR

>sp_Q12VF4

MKQRFVLDTTALTDLQTREMLEIEDLCQGMRAILDLIAESRLHLQISCYVPFPSVYKELHEFAKNNGCDDEIIAMIDTWLVKKTPDRHEVKIPSHIFHEYVSYMRERINKGMTVAEEAIREASIQCLSIESEIEDTRKVEDNTDREVIGPIVGKFRNKYRSALRYGILDSAPDIDVLLLAKELDAAVVASDYGIQKWAEQLGVRFVPANTFPMMLKEYLRHTPSDGTHQK

>sp_Q12ZU6

MARIKYTTELDAETSAKAMGSELHISPKKSRELCKAIKGMRTNAARQYLEDVVILKQAVPFGRHNDSLGHRKGPMAAGRYPVKVASEMLKLLKNAESNAEYKGLNPEHMFIAHTAMNRGRVIHGMRPRARGRASPENTETVNLEMIISEVR

>sp_Q47W08

MKKDIHPNYVEFNATCSCGNVVTTRSTIGKDIHLDVCSACHPFYTGKQKAAETGGRVDKFNKRFAAIGKK

>sp_Q489A1

MAKLHDLYKDTVVAELQKQFGYKSVMQVPRIEKITLNMGVGEAISDKKVLEHATNDLTAISGQKPITTVARKSVAGFKIREGYPIGTKVTLRGERMWEFLERLISISIPRIRDFRGLNPKSFDGRGNYSMGVREQIIFPEIEYDKIDKIRGLDITITTSAKDNEEGLALLSAFDFPFKKKV

>sp_Q47Z09

MKESTQQFLALTSPGIEVLLFDEIKSLGAFNVIQKPEGVYFQASLALGYKISLWTRLATRIMLKLGEGEAKDKDELFKAASSINWPDIFKSTTTFAVDFVGYSEEIRNSQFGGLTIKDAIVDQFRDQGFERPNVDKKAPQISFQARLLRDNVTIFLDFSGRGLFQRGYREHSGAAPLKENLAAALIIRSGWLNDTSKPLVDPMCGSGTILIEAVSMAAKQAPAINRQSWGFEAWLSHDNAVWQKQLTLAIDSSEENMSNLKVKVFGIDIDERVLRTAQQNARNAQLHQYIEFTCKNTNDMDNTFGQPGTILFNPPYGERIGELPELVENFVLFGQKLKMQFKDWRIAILTANVDLLSMLKLSSFKRYKFKNGPLDCQLALYNLDAKQGAKDAINPQSDFAEEDSAFANRLKKNRKNLKGWLKSNEIEAYRLYDGDIPEYNVAIDIYGEYLVIQEYAAPKTIEEDKAKKRLQEVIYWAPKVLNIPTDKVILKTRAKQRGANQYERLEKTKQSLTINEHGALFKINLWDYLDTGLFLDHRKTRQIVAKKSQGKSLLNLFAYTGSVSVQAAVQGAASITTVDMSNTYLNWAEDNFALNKLNGHKYQFIQADCLDWLKKNVNKFDVIFIDPPTFSNSKRMEDSFDVQRDHVDLITDALKSLNRGGEIFFTNNKRNFKIDFEALDELGLTAEAMSDVTRDKDFARNKHIHNSWSIKRTAT

>sp_Q47UV9

MAYSYFEKKRIRKDFGKSVQVMEYPFLLSIQLDSFRKFIDTDPTGETGLEAAFRSIFPIKAYSGSSELQYVSYRLGEPLFDVKECQIRGVTYSAPLRVKLRLVVYDKEAAAGTVKDIKEQEVYMGEIPLMTDNGTFVINGTERVIVSQLHRSPGVFFDHDKGKTHSSGKVLYNARVIPYRGSWLDFEFDPKDNLFVRIDRRRKLPASIILRALEYSTEEILAMFYDTTDYTIKGDKLIMDLIPERLRGETAIFDISIKKGEVLVESGRRITARHIRALSKAKLEKLEVPADYIVGRVLSKAYIDKSTGEVIAEANAIITLELLAELSQAGHKVLSTLYMNEFDVGSYMSDTLRVDSSTNKLEALVEVYRMMRPGEPPTKDAAEGLFSNLFFASERYDLSTVGRMKFNRRVGNADDVGTGILSKEDIISVMKTLIGIRDGKGEVDDIDHLGNRRIRSVGEMAENQFRVGLVRVERAVRERLSLGDLDAIMPQDLINAKPISAAVKEFFGSSQLSQFMDQNNPLSEVTHKRRISALGPGGLTRERAGFEVRDVHPTHYGRVCPIETPEGPNIGLINSLSCYARTNDFGFLETPYRKVIDGLVTDEIDYLSAIEEGNFVIAQANAERNDSNKLVQDLVNCRHRNEFILKASAEVQYMDVSPQQIISVAASLIPFLEHDDANRALMGSNMQRQAVPTLRVDKPLVGTGMEKVVAVDSGVTAVAKRGGVVSYVDASRIVVKVNENEMHAGEAGIDIYNLTKYTRSNQNTCINQRPVCRMGEPVVRGDVLADGPSTDMGELALGQNMRIAFMPWNGYNFEDSMLLSERVAIEDRFTTIHIQELTCIARDTKLGSEEITADIPNVGESALSKLDEAGVVYIGAEVNGGDILVGKVTPKGETQLTPEEKLLRAIFGEKAADVKDSSLRVPNSVKGTIIDVQIFTRDGVEKDARAVEIEQMQLKEVKKDLGDELSILEDGIYARTKKLLLSAGLNESDLTSMSRDKWLTQNLADEGQQAELEQIAEQFDNIKEDFDKKFEVKRRKITQGDDLQPGVLKIVKVYLAVKRHIQPGDKMAGRHGNKGVISNVVPVEDMPYDQFGVPVDIVLNPLGVPSRMNIGQILETHLGMACRGIGEKINRMLEAQQEIHKLRNFIQEVYNVGESRQVVDVASFSDDEVLRLAGNLRAGLPIATPAFDGAAEKEIKELFVLADMPQSGQFVLTDGRTGREFERPVTVGYMYMLKLNHLVDDKMHARSTGSYSLVTQQPLGGKAQFGGQRFGEMEVWALEAYGAAYTLQEMLTVKSDDVNGRTKMYKNLVDGDHRMEPGIPESFNVLLKEIRSLGINIELDKD

>sp_Q12ZM1

MVRNDLVMEIPWDIEATLLSRPNRFLGIVEMDESTSSGPFQEKVHIHDPGRLEDLLYPGNRLLLRKATNPKRKTGWDVIAAKADDGWILINSIFHRRIAEWAIANKVCSCFENVLEVIPEQKFGDSRLDFLLKKSDTELWVEVKGCTLIYGNTATFPDAPTTRGKRHVGELKKALESGSEALILIIILRKDALCFKANASIDPDFAEVFKDAVNAGVQVCPLVFGYEGRELFYKGMVPLCTEEYNSI

>sp_Q12TM7

MAKMHTRTKGKSGSTKPIRSESPAWSTATTEEITKVVLDLWKQGNSTSVIGMVLRDNYGVPDVKLATGKKVTEILRDNSEEPNVPEDLYNLIVKAIGLRKHLVVNNKDVHNKRSLQSAESKIRRLVKYYQSTKVLPIDWKYKPETAEMLITR

>sp_Q487H5

MIKSTAEVRQAFLDFFATKQHQIVKSSSLVPGNDATLLFTNAGMVPFKDVFLGAETRSYTRATSAQRCVRAGGKHNDLENVGYTARHHTFFEMMGNFSFGDYFKNDAISYAWEFLTGELGLAKEKLLVTVYATDEEAFSYWRDEVGVPEDKIIRIGDKSANKKYESDNFWSMGDTGPCGPCSEIFYDHGEDIFGGPPGSPDEDGDRFIEIWNIVFMQFNRQSDGRMDPLPNPSIDTGMGLERISAIMQNVHSNYEIDIFQALIKDTAALLDCSDLEHKSLRVIGDHIRSCSFLIVDGVVPSNEGRGYVLRRIIRRAIRHGHKLEATGHFFHKLVASLIAQMGEAYPELAQQQAIIEKLLRIEEEQFGRTLDRGMILLEDILANLSGDIIKGDDVFKLYDTYGFPADLTADIARERNLKIDKDGFDVAMKQQRERAQQASQFGTDYNQQLKSDQNTAFKGYDNDSYSATVVELFNSQDQDPVSQLNSGEQGIVILDHTPFYAESGGQVGDSGLLHLDGGVFEVTDTIKLGNAFAHRGTAHTDVGLNRRVKAEINVERRAAIVKNHTATHLLHEALRKVLGEHVTQKGSLCDSDKLRFDFSHFEGVTAQELHDVEQMVNNEIRRNHAKQTESMYIEEAKAKGAMALFGEKYDDEVRVVTLGDFSIELCGGVHVNRTGDIGFLKIVSESGIAAGVRRIEAVTGTGALDFINQQTASLTTIAALVKSDVTNASSKVELLISRSKQLEKEIGQLKQELAAQAGSDLVNNTIEINGVKVLIADLGSVESKALRGMVDELKNKMQSGVIMLATANGPKVGLIAGVTKDLVGRVKAGDLVNMVAQQVGGKGGGRPDMAQAGGSQPENITSALESVSAWLTEKLA

>sp_Q484Q5

MEAIYNPQAIEATVQKFWTDNNTFQAIENPDKEKFYCLAMFPYPSGRLHMGHVRNYSLGDVISRYQRMQGKNVMQPMGWDAFGLPAENAAIKNKTAPGKWTYENIDYMRNQLQSLGFGYDWGRELATCKPDYYRWEQWFFTQLFEKGLVYKKNATVNWDPVDQTVLANEQVIDGRGWRSGALVERKQIPQWFIKITDYAEELLDDLDQLTEWPEQVKTMQRNWIGRSQGVEMTFAVADSTESFDIYTTRPDTLMGVTYVALAAQHPLAVAAAVDNADLAAFIDECKNSKTTEADMAAMEKKGVDTGLKAIHPLTGKLVPVWAANFVLMDYGSGAVMSVPGHDQRDYEFALKYGLAIEQVIAGQEADDINKAAITEKSTLINSGEFDGLDFEEAFKAISDKLISENKGKTTTNYRLRDWGVSRQRYWGTPIPMINLANGESVPVPTNELPVVLPEDVVMNGTTSPIKADPEWAKTLYNGEEALRETDTFDTFMESSWYYARYCSPNDDTQMIDPAKANYWLPVDQYIGGIEHAILHLLYSRFFHKLLRDVGLVKCDEPFKKLLCQGMVLAETYYREADNGAQEWIAPTDVEVERDEKGQITSSISKIDGQPVLSAGMSKMSKSKNNGIDPQEVIEKYGADTVRLFIMFTSPPEQTLEWSDAGVEGAHRFVKRVYKLAHEFVESTNNSAVVDIAELTLNADHKKLRRELHKTIAKVTDDIGRRNTFNTAIAAIMELMNHLGKAKVNSDEDKAVMQEAVRAVVLMLTPITPHLCHHLWQLVGGSDENVEDASWPVVDNSALVEDEKLIIVQVNGKVRAKITVAADASKEDVEALGLNDESVLKFTDGNTIRKVIYIPGKLLNIVAN

>sp_Q480K7

MFYPAIRKVLFQFDAETIHELTIKGLKSTGKSPFNAFYKQTVQDKPLTVMGINFPNPVGLAAGLDKNGECINAFDAMGFGFVEVGTVTPRPQPGNDKPRIFRLPEANAVINRMGFNNKGVDYLVSQVQAANFKGILGINIGKNKDTPEENAKDDYLHCMRKVYDLATYITVNISSPNTPGLRSLQYGDALNELLAALKAEQTILTEKYGKYIPLAVKIAPDLTGDEVKSIAKSLIDNGIDGVIATNTTLSREGVEGLQFGTEQGGLSGQPVKEKSTLVIKLLSEALNNKLPIIGVGGIASSDDANEKLEAGASLVQVYTGFIYQGPPLVKEIVNGL

>sp_Q482T8

MAREYARTDRVGQQIQKEIATILMREIKDPRLSMTTVSAVEVTRDLAYAKIFVTFFNDNQDEIKASLEVLAEAEGYIRSLLGKRLRARIMPHLRFVYDSSMSEGVRMSALVDQAVASDKNGDAEVDDTQVDDEPSVDSEKGE

>sp_Q12ZV1

MAKGHRPRRGSLAYSPRKRSQSHIPRFRSWPESDAEPKLQGFAGYKVGMTHVIMIDDVKHSLTEGTEISVPVTIIETPAIRVAAIRAYGKDTYGEIAIAEAWTDVLDKDLSRRLKTAKNPDVNASLEKLETLVESGRANDIRLITYTLPSTLTGVPKKVPDVMETGVSGSDVKAKFEYAKTVLGTMVEISDVFDNGKIVDVAAITTGHGTQGPVKRWGINLMKNKHSRQGSLRQVGTLGPWTPAHVSWRVPQAGQMGYHQRTDYNKRILKMSSDVDEVNPAGGFVNYGLVRGNYILIKGSVPGPSKRLIRLREPTRSKVSSIGEPQIMHVSTQTLQG

>sp_Q489A0

MAKTSMKAREAKRTKLVAQYAEKRAALKAIISGTDSSDEERWDAVLKLQSLPRDSSSSRQRNRCNITGRPHGFLRKFGLSRIKLRETMMRGEVPGLKKASW

>sp_Q480Q2

MLDAKLLRTDLDNIAQQLTKRNFVLDTEALSALEELRKAIQVKTQELQNERNTRSKSIGQAKARGEDIAPLLAHVSQLGNDLDAAKTEQDEVLAKIDAIASAIPNLLDDSVPEGKDEDDNVEVKRWGTPREFDFEVKDHVDLGFSVDKGLDFESGAKLAGTRFVVMRGKIARLHRAIAQFMLDTHTEEHGYQEMYVPYLVNAASLYGTGQLPKFGEDLFHTDLSNKKFSLIPTSEVPLTNLVRDEIVDEDDLPIKMTAHTPCFRSEAGSGGRDIRGLIRQHQFDKVEMVQIVKPETSMTALDELTRHAEIILEKLELPYRTVQLCSGDVGFSAAKTFDLEVWLPAQDTYREISSCSNMGAFQARRMQARFRNNETNKPELLHTLNGSGLAVGRTLVAILENYQQADGSINVPTVLQPYMGGLTNIG

>sp_Q47W53

MSVFEIKHPLVQHKISLMRAKDMSTRSFRQLSAEVGSLLTYEATKDLELENFQMEGWDGEITGQRLVGKKATVVPILRAGIGMLDGVLELMPSAKISVVGLYRDEETLEPVTYFEKLAGDIDQRLALIIDPMLATGGSMNATIDILKKAGCNDIRALVLVAAPEGIEKVKSAHPDVDIYTASIDDHLNESGYIIPGLGDAGDKIFGTK

>sp_Q47YC9

MLCAIYKSARKAQTYLFVNKRDDFSSVPEGLMKTFGTPNLVTLINLATKDKLAMADLEKVKKNLIEKGFYLQLPPPQEDLLKEHKAAMAAEKEQGEL

>sp_Q47YJ3

MANKKHKASSQRWLDEHFDDEYVKKAQRLGLRSRAVFKLEEINIKDRLIKPGMKVVDLGAAPGGWSEYAVKVVGDKGQVVACDILPMDAIVGVDFLEGDFREEEVLDALLTRINGKNIDVVMSDMAANMTGNESADSARSMYLVELALDMCSQVLKPNGAFVVKVFQGAGFEDYMKATRAVFKAVKTRKPESSRARSREVYLVATGYKG

>sp_Q12ZT1

MAYEYNEEWVPQTRLGKLVFDGQVTSMDEAMDSGLPIRESKIVDLLLPELEDEVLDINMVQRMTDSGRRVKFRATVIVGNGDGFVGLGQAKDVQVGPAIRKAIDNAKINITRIKRGCGSWECGCGLPHTVPSEVNGKAGSVTVVLKPAPRGLGLAAGGTARKVLEKAGVKDVWTRTEGQTRTTLNFAKATYNALMNTGTVRRPIVFDETEA

>sp_Q47YC2

MASTSAASNELVSSTINTILPTLVDGLDLNQRQSHDFFQQVLQGNIDPALMASVLTALKIKGETPEEIAGAAIAIRAAATPFPERNKEDIVADCVGTGGDGANTINISTTAAVLAAACGLKMAKHGNRSVSSMSGSADLLEAFGVNLSMSPETANHCLAQTNLCFLYAPAYHSGFKYAGPVRKAMGIRTLFNILGPLVNPAKPNIMLLGVYTPELLMPMAQALQLTGVKRAFVVHGSGLDEIALHGNTQAIEINNGELIERTISPQDFGLKNYTLEEIKGGTPAENADIIRDILSGQGKDAHNAAVIVNCAALLYLHDKAESLTQAAQLATEVLASGKGLSTLLTLVKLSNQDVSSTQTELKADK

>sp_Q12WL1

MEVNGEIYRVAGPVVTVIGIKPRMYDVVKVGHEGLMGEVIRIKGEQATVQVYEDTSGLKPGEPVMNTGLPLSVELGPGLLESIYDGIQRPLPVLQEKMGNFIQRGVTANGLDRERVWEFKPTVSKGDEVKGGNILGLVQETKNIEHKIMVPPSISGTIKEIKAGSFKVDETICVLTDGTEISMMQKWPVRGPRPVAKKLMPTKPLITGQRILDGMFPIAKGGTAAIPGPFGSGKTVTQQQLAKWSDTDIVVYIGCGERGNEMADVLNEFPELEDPKTGRPLMERTVLIANTSNMPVAAREASVYTGITIAEYYRDMGYDVSLMADSSSRWAEAMREISSRLEEMPGEEGYPAYLSARLSEFYERAGAVNSLAGLDGSITVIGAVSPPGGDFSEPVTQNTLRIVKVFWALDAKLSQRRHFPSINWLTSYSLYTQGLADWYSENVGADWTQLRDDAMDLLQQESELQEIVQLVGSDALPEDQQLTLEVARMVREYFLQQNAFHPVDTYCPFDKQYKLLKSITRYGELATAALESGVPMNKIITIKSKDELAKVKFEENFDAALDVVMKKMDEEFAQIGGN

>sp_Q12ZT0

MFAVVRVRGPVNVSGKIEDTLGMLRLHKVNHCVFLQDTPNNKGMIQKSKDYIAYGTVDVESLTEVLSKRGRLEGDARLTDEYVKENSEFASIASFAEALVNDDAKITDVPLLKPVFRLHPPRKGHAGIKRTAQQGGVLGNHGDDIKALLNKMR

>sp_Q488Z8

MSRVAKAPVVVPAGVTITLSGQDITVKGPIGELSRTIHSDVVVSQEENNIITNIVADVKGAWAQAGTTRALINNMVEGVSKGFEKKLVLQGVGYRAKAAGKSLDLSLGFSHPIKHAIPEGITCETPSQTEVTLKGCDKHLVGQTAANIRAYRKPEPYKGKGVRYVDEYVRRKEAKKK

>sp_Q487Z3

MTIGLVGRKVGMTRVFTEDGVSTPVTVIEVEANRVAQVKTVDNDGYSALQVTTGKRKASRVTKPAAGHFAKAGIEAGRGLWEFRLNENEGSDIEAGSEITVEVFNDTKLVDVTGTSKGKGFQGGIKRWNFTMQHATHGVSLSHRSNGSLGQCQTPGRVFKGKKMSGHMGAVRCTTQNLELVRVDAERNLLLIKGAVPGAINGNVIIKPAVKA

>sp_Q48AS2

MTTETLLIELGTEELPPKSLKTLATAFYDNIKGQLDSHNLSYSDIKWFATPRRFAVQVFDLVEKQDDKIVEKRGPAVNVAFDDAGNASKAAQGWARSNGIEVDQAERLVTGKGEWLLHRATVSGKAVVELIPDMVTTALNKLPIAKPMRWGAERTQFIRPVQTLTMLFGSDIIAGEALGVSSSNQVQGHRFHHEGLVTINHANDYQAELAKAYVEVDFNERQNKIVAQIKQVANDIDAVALIDEELLNEVTALVEWPVTLVGTFDEDFLNVPAEPLIYSMKDHQKYFPVTDKNGQLVNKFIFVTNIESKDPNTIIFGNEKVIRPRLADAEFFFKTDKKQSLESRLKSLESVLFQKQLGTLKAKSERIASLSQFIAEQLNENAQDAYRAGLLSKTDLMSDMVLEFPQVQGTMGKYYALHDGENENIAQALEDQYRPRFAGDSLPEANIGCAVAISDKIDSLVGIFGINQAPKGDKDPFALRRAAIGSIRIIIEKQLDLDLSTLINKSIELFGDKLVNENTATDVLEFIMGRFRAFYQEQGISVDVIQAVLAKKPSAPLDFEKRIKAVTFFGELPEAATLAAANKRVGNILAKFDGELYQSFNTDLATEQAERDLADIYRDISLKVAPLMADKNYQAALSELAQLKAPIDTFFDGVMVMSDDEAVKINRLTLLNQIRNSFFAIADISVLQ

>sp_Q12X87

MEKKNRLILALDVTDRENALRIANEVSDYVDSIKVGYPLVLGEGLSIVKELVEIAPVIADFKVADIPNTDRLICEHVFNAGAAGIITHGFTGRDSLDSCVKVANEFGTDVYVVTEMSHPGGVEFFRPVAEDIASMAVEAGASGVVAPATRPERVKDIRKIIGEELSIISPGVGAQGGSAADVIRAGADWVIVGRSIYNSDSPAEAAKKICDEMNC

>sp_Q489A2

MASKIRRDDEVIILAGKDKGKTGKVTKVLVEDGKVFVEGINLIKKHTKPVPQLQQPGGIVEKEAPLQVSNVAIVNSATGKADRVGFRIEDGKKVRFFKSNNELI

>sp_Q488Z7

MDKRSSRLRRAKRARAKISELGANRLVIFRTPRHIYAQLIAPTGSEVIASASTLDKEVSAQIEKTGNVAAATAVGKAIAERAVAKGITKIAFDRSGFLYHGRVKALAEAAREAGLQF

>sp_Q12ZU0

MVSKQPRKQRKARYEAPLHMKQKYMAAPLSKALRGKYGRSANVIVGDTVIVMRGDHAGTKGKVEALSLKSGTIVVEGVSVSKVDGTEVPRPIYPSNVMITSLEMNDKHRDSILSRGR

>sp_Q47VL2

MYAVFQSGGKQHRVTEGQTVRLEKLELEVGATVEFDNVLMIANGDNINVGAPYVAGGKVVAEVVTQARAPKITIVKFKRRKHSRKQAGHRQWFTEVKITGING

>sp_Q48AD6

MRDKIRLVSSAGTGHFYTTDKNKKTMPEKMEIKKFDPTIRKHVIYKEAKIK

>sp_Q12ZV0

MTTANIIDLSGNSKGEIALPEVFSEIFRPDLIKKAVLSAQANRLQPYGTKLYAGMQTSAHSWGSGRGVAQVPRISNGSRVARIPQAVGGRRAHPPKTETDRTEKINKKEKRLAVRSAIAATIDADLVKARGHKFTAEVPFVADDAIEGLVKIKEVISFLQAAGLYDDIIRAKEGKHIRAGKGKRRGRKYKNRKSVLIVTGEESLLSKAANNLPGVDVATVTALNAELLAPGTHAGRLTVWTQSAITNMEGMFL

>sp_Q12ZR6

MADEEIRHLVRIMNTDLQGKKPVKYALTGIRGIGLRTSRVIVDSTGIDPNAVIGYLSDEDIKKLDSTIDKFEEQLPKWMLNRQFDPLTGENKHLLGQDIILTLKEDLNDLKKSRAYRGLRHERGLKVRGQRTKSTGRRGSTIGVRKKK

>sp_Q47WV0

MWIGVISLFPEMFNAITEYGVTGRAIRNGLIDFHLWNPRDFTHDKHRTVDDRPYGGGPGMLMMVQPLRDAIAAARKAAEVNGGKAKVIYLSPQGRKLDQQGVSELAQHDRLVFIAGRYEGIDERLIASDIDEEWSVGDYILSGGELPAMNVIDAVARLVPGVLGHKESAEQDSFSNGLLDCPHYTRPEVLTTPQGDEMSVPKVLLSGNHEHIRLWRQEQSLLRTWTRRPELLNNLALTAEQEKALTLIKKQVK

>sp_Q489F4

MDTPRPIKRALLSVSDKAGIVEFAQRLSEKGVDLLSTGGTAKLLAENGIKVTEVSDYTGHPEIMDGRVKTLHPKVHGGILARRGIDEVVMDENGISAIDMVVVNLYPFANAVSDENCSLENAIENIDIGGPTMVRAAAKNHKDVTIVVNASDYERVLTELDNNNDSLTYKTRFDLAIAAYEHTASYDGMIANYFGKMLPAYGETESKASLENKVKFPRTFNSQFIKTQDLRYGENSHQDAAFYKEENPEEASVSTATQLQGKALSYNNIADTDAALECVKEFDEPACVIVKHANPCGVAIGDDILAAYEGAYKTDPTSAFGGIIAFNRELDADTAEAIVSRQFVEVIIAPSVSDAAAQIVAAKPNLRLLECGQWDNKTTGFDFKRVNGGLLVQDTDQGRVTSDDLTVVTKRQPTDEEMRDLQFCWKVAKFVKSNAIVYVKNSSTIGVGAGQMSRVYSAKVAGIKAADENLEVKGSVMASDAFFPFRDGLDAAAEAGITAVIQPGGSMRDDEVIAAADEHNIAMVFTGMRHFRH

>sp_P96175

MKSNHMQVEAICNGYVIDHIPSGQGVKILRLFSLTDTKQRVTVGFNLPSHDGTTKDLIKVENTEITKSQANQLALLAPNATVNIIENFKVTDKHSLALPKEVENVFPCPNSNCITHGEPVISSFTIKMIKGNIGLKCKYCEKTFSKEIVTAQV

>sp_Q48A17

MGKNVVVLGTQWGDEGKGKIVDLLTDKAKYVVRYQGGHNAGHTLVIDGEKTVLHLIPSGVLRDNVKCLIGNGVVLCPKALMTEITMLEAKGVPVRERLLISDACPLILPYHNALDVAREVARGNKAIGTTGRGIGPAYEDKVARRGLRVGDLFCAETFAAKLKEIVEYHNFSLVNYYKVEPVNYEEVLADALAVADTIKKMTADISEILDQARIAGESIMFEGAQGTLLDIDHGTYPYVTSSNTTAGGVATGCGVGPRHLDYILGITKAYTTRVGSGPFPTELDDEVGNHLGTVGHEFGATTGRERRCGWFDAVAMHRAIQVNSVTGFCLTKLDVLDGLETLKICTGYKTEAGDIITVPPTAAEGYDKITPVYEEMPGWTESTVGATSVDVLPENALAYIKRIEEITGIPVDIISTGPDRVETIIKVNPFL

>sp_Q485G8

MTTNPKPAYRRILLKLSGEALMGDEGFGIDPKVLDRMAQEIKELVEMGIQVGLVIGGGNLFRGAGLAEAGMNRVVGDQMGMLATVMNGLAMRDALHRAFVNTRLMSAIDLAGVCERYNWANAISLLKSGRVVIFSAGTGNPFFTTDSAACLRGIEIEADAVLKATKVDGVYSEDPAKNPEAELYSELSYDEVLDKELKVMDLAAFTLARDHNIPIRVFNMNKPGALKSVIMGNTEGTVISHKKRTET

>sp_Q12ZT5

MAKEIKKIISIPEGVTVTFEKNVLSASGPKGTNTRFLWYPGVNIEVNGSEIIVDSASSRKKQKAMVGTYTSHITNMMKGVVDGFEYHMKVVYSHFPMQIKVNGKKFVINNFLGEKKPRVSNILGETSVKASGDEVIVSGINKEDVGQTAANIEQKTKIKRFDPRVFQDGIYIVDKRV

>sp_Q12VB0

MHRINALKHLALLGALKKPVKISSSEFTRYTSTGSKTAARILKQLEEEGSIDRLIIPEGQMISITEKGHKWLESEFSDYKHIFCGDEDKVELYGNVITGLGEGQYYIAQDGYGSQFEEKLGFKPYPGTLNVRLTSHSADILKRKSQKNIIPISGFTDGQRTFGGCNCYFVEVEGVRGAVVTPERSHYPHDLLEIISPVHLRKTLELNDGDEVKIMIEDRSACE

>sp_Q12TI4

MIPVRCFTCGKVIAGSWEEYKRRTGEGEDPATVLDDLKFVRYCCRRMFLAHVELVDTMAPYQ

>sp_Q48AN3

MRPSGRTLGQIRPVTITRQFTTHAEGSVLIEFGDTKVICTATVEVGVPRFLKGQGKGWVTAEYGMLPRSTHTRMRREAASGKQSGRTLEISRLIARALRAAVDLKALGENTISVDCDVIQADGGTRTAAITGACVALVDALNYMRAKDIIKTNPLKHMIAAVSVGIYKGEPVADLDYPEDSAADTDMNVVMTDTGKLIEVQGTAEEEPFSFEEMQAMLELAKNGINELFDLQKAALS

>sp_Q482U3

MTDYLLLLVGTVLVNNFVLVQFLGLCPFMGVSSRTETAIGMSFATVFVMTLASLLSYLVTTYLLVPLNLEYLTTMSFILVIAVVVQFTEMVVHKTSASLYRLLGIFLPLITTNCAVLGVALLNLRLQHGFFESIIYGFGAALGFSLVLIMFSAMREKLANADVPAPFKGTAIAMITAGLMSLAFLGFTGLVKI

>sp_Q485J2

MNIIEGSFEAKGKKFAIVVSRFNHFIVDSLLDGAVDALKRHGNVNDEDITIVRVPGAYELPLAAKKIAKKGEADAIIAIGAVIRGGTPHFDFVAGESNKGLAQVCLESEIPVSFGVITTDSIEQAIERAGTKAGNKGAEAALGALEMVNVLAQI

>sp_Q48AD7

MSKICQVTGKVPMVGNNRSHARNATRRRFLPNLQSHRFWVESENRFVKLRLTPKGMRIIDKKGIDVVLTDIRARGEKV

>sp_Q12TL1

MKYYLQKLIDGHDLSMVESEAAMGQILESATDAQVGAFVMGMKMKGETSDEVAGFAKGMLNVANMIRPKVDGILVDTCGTGGDRHNTINISTAAAIVAAAAGVTVAKHGNHSFTSLSGSADVFKELGVKIDLEPDLVKSSIEDIGIGFMLAPKFHPAMKRMVGPRKELAVRTMFNILGPLTNPTGAKAQVIGVFDKDLCNLMAEVLKKLGKEHVMVFHGDGMDEISTLSETFVAELKDGIISNYTLTPEELGVARAKATDIVGGTPEENAHDLLYILNGEKGAKRDIVVVNAAAAIYVAGLAISIKDAIPLAEEAIDSRKALNKLKELVEFTSGNKVDNEAFNTGQSLRNEVC

>sp_Q489T8

MEVILLDKIAKLGGLGDKVSVKSGYARNYLLPQGKAVFASEANVEHFEARRADIEAKLADVLATAEARAAKVVALAEVTIASKSGDEGKLFGSIGTRDIADAITEAGVEITKAEVRLPLGAIRETGEFEIAIHLHHDVDTSIKVVVIAEA

>sp_Q488Q3

MISPFIVNDKNLFLSRFPVSQVNRSLQAWDSADEYLINHVHDQNLINAQTKVAIFNDAFGALAVNFCQSTSENPEVISINDSYISSEGASYNIEQNSLDDSHFTQLNSLDSLPNNIDVILYKIPKSKSLLIEQLIQIKKSVNENCIFIAADRAKEIHSSTLKVFEKHLGTTKTSLAVKKARLVFCQFDNKQVHQSPFPTVWSIPHKSTNDLPSRELTISNHANVYAREKLDIGARYFIENLPTVAANSTVIDLGCGNGVIGLTVLANQPEAHVQFIDESTMAISSAKQNIMTNLPDVIEQCEFTLNDSLTDIEGGSVDLILCNPPFHQNTATTDHIAWQMFKDSHRVLKKGGELRIIGNQKLAYHIKLQRLFGNETLIASNDKFVTQSAIKR

>sp_Q12YP2

MPIITLQYEDLESLTKADKDTIIDRVPMIGADIERIEKEAIDIEFFPDRPDLYSVEGVARAMRGFMNIETGLCQYEVTPSGVHIELDEKIKEVRPVLGCAIVKGINFTSSSIKSLMDLQEDLHWGLGRNRKKVSIGVHDMTNIEPPFKYQAVSPDFEFVPLDFTEPMTMDEVLEKHPKGVRFANILEGMEKYPLITDSEGKVLSFPPIINGTLTRVEESTTDLFIDVTGLGDAVYTALSIVVSALAERGGKIESVKVVYPDGTEKVTPDMTPRTLNVPRSDIDSLIGIKLTDDEIINELKRMRFDASVLEDGDMFEISVPTYRADILHNFDIVEDIAIGYGFDKIKSEFPKSATIGCAHPISVTRGVMREIMVSLGYSEVMPFTLTSQKVHFEWMNREETDDVTFVLHPISEDQTMVRTTILPNLIEIFSLNQHHELPQRLFEVGEVVVNSKNRLHLAAASIHAQANFTEIREVLDAVMRERDIEYEVVVSEDPAFIEGRRADILVNGEKVGMMGELYPEVIINFGLGQPIVGFEIDLTE

>sp_Q47ZS5

MKFSESWLREWVNPALSSDDLAHQITMAGLEVDGVDPVAGEFSGVVIGEVVECGPHPDADKLQVTKISLGDYSSTTVEKGELVTIVCGAKNCRLGLKVAVATVGAVLPGDFKIKKAKLRGVPSFGMLCSESEIGLADDSDGIMELASDAPLGQCVREYLDLNDVTIDVDLTANRGDCLGLKGLAREVGVLNSLEVSEPTITAVAPTIDDVITINIEANEACPRYLGRVIKGINPNATTPLWMVEKLRRCGTRSIDPVVDVTNYILLELGHPMHAFDLAKLDGGINVRFANKDEKLTLLDENEVTLKEGTLVIADENKALAMAGIFGGLESGVTNNTTDLFLESAFFAPLAILGKARQYGLHTDSSHRYERGIDPTLQHDAIERATELLLSIVGGQAGPVVEAKSDADIPQTKDVNLRRKMLDSRIGHHIEDAQVSEILTRLGFTVTTTGEGEAKVWQVIVPAYRFDIKIEVDLIEEVARIFGYNNIPNIAPKATLKMCEQKEANLSLSNLKQTLVNRDYQEAITYSFVDPKVQALLHPGQEVMTLPHPISSEMSVMRLSLWTGLLQSMVYNQNRQQGRIRLFETGLRFVPDESAENGVRQQNMIAGVISGLRVDEHWSMEKAATDFYDIKGDVEALLALTCDAQGYEFSKAEVDALHPGQTAQITKNGVFVGCVGTLHPELERKLGLNGRTLIFELLLSEVLVQKIPEATDISRFPANRRDLAIVVKEDVDAKNVLQLIEKVGGNYLIDLNLFDVYKGQGIDDGFKSLAIALVLQDTSKTLEEKDITDVIDRVVATLKTELNASLRD

>sp_Q12WD4

MQLLLIHSDYIEYEVKKSTPVAEEIEESFKQGRLEDALTAFMAVESFDEANPQEIIDRAVSEIENVAGQVKAENIMLYPYAHLSSDLSSPKVAVSVLKGIENALEGKYNVMRAPFGWYKAFRISCKGHPLSELSRTIRLEGAVPCGKVVSLDAEKKEVVSEALKAEDSAKSYWRILTPDGELHDAETFDLTGHDNLQKFVDYEISKNRNIEKAPPHVELMRRLEIADYEPGSDSGNMRYYPKGRLMKSLIENFVLEESSKIGAMEVETPLMYDMNHPTLKKYLDRFPARQYSIESDKRHMFLRFAACFGQFLMNHDMTISYKNLPLKMIEMTRYSFRKEQRGELVGLRRLRAFTMPDMHTLCPDMEAAISQFGEQYSMCIDILRKIGIDVSDFEVAIRFTREFYDDNREFITELAKKVDKPVLVEMWDTRFFYFVLKFEFNFVDAIAKASALSTVQIDVENAERYDINYVDANGKINRPTILHCSPSGAIERCIYALLEKAAMDAEEGKVPNLPVWLSPTQVRVIPIAERHMDFAQEVADSLLCRADIDDREETVGKKIRDAGREWIPYVAVIGDSEVESGKVTVTIRAESEPKKPMKVEMTAEELSERVFNEIGDMPYRSLPLAKLLSMRPKFI

>sp_Q488M6

MLQQNNEQLLNLLPDKMMEKTFNPTDIEQSLYTSWEEQGYFSPTGEGDSYSIAIPPPNVTGSLHMGHAFQQTIMDTLIRYQRMQGKNTLWQTGCDHAGIATQMVVERKIAAEEDKTRHDYGREGFIDKIWEWKEESGGTIGKQMRRLGNSIDWSRERFTMDDGMSEAVQEVFVRLFEDDLIYRGKRLVNWDPKFHTAISDLEVENKDKKGHMWHLRYPLANGAKTAEGLDYLVVATTRPETMLGDTGVAVNPEDPRYKDLIGKQVLLPLVNRLIPIVGDDHADMEKGTGCVKITPGHDFNDNEVGKRHALPQINILDKDAAILATAEVYDTKGEVCNAYDTGLPSEFAGMDRFVARKAIVAKFDELGLLVEVKDHDLVAPYGDRSGVIIEPLLTDQWYVRVEKLAGPAVDAVKDGQIEFVPKQYENMYFSWMNNIQDWCISRQLWWGHRIPAWYDENEKVYVGRTEEEVRANNDIAADMKLRQDDDVLDTWFSSALWTFSTLGWPKDTEDLKTFHPTDVLVTGFDIIFFWVARMIMMTMHFNKDENGKAQIPFKKIYMTGLIRDENGDKMSKSKGNVVDPLDMIDGISLEDLLQKRTGNMMQPKLAKKIEKLTRKEYPEGIEAHGTDALRFTLTSVATTGRDISWDMKRLEGYRNFTNKLWNASRYVMMNTEEFDCGQSSPEGKAGDMELSLADRWIIGQFEQTVKTVHEAFDTYRFDLASQALYEFTWNQFCDWYLELTKPVLFKENEAQQRGTRHTLVNVLEALLRLMHPIMPFITETIWQRVQPLSDFSKNGDSIMVQAFPQFDESKCDQQAIDDLEWVKQFIIAIRNIRGEMDISPSKELPVLLKNVNDNDQRRLDENEQFLSSLAKLESITVLADDEQGPASASAVVGDLSVLIPMAGLIDKEAELARLDKAIEKLEKEAGRVRGKLGNENFVSKAPAAVIEKEQAKLADAESTLAKILEQKIQIAAL

>sp_Q48AT8

MTTTIFQQTNHTLDAIGLRCPEPVMMVRMNIRKIASGETLLIKCDDPSTARDIPSFCRFMEHELLAKQTDTLPFLYVIKKN

>sp_Q482P5

MSARISLNVGREKSLLRKHPWIFSKAVNKIKGNPMLGDTVDVFDSKGNWLAKGAYSPESQIRIRVWSFDEHEEIDRDFFFAKLQSAQARRDWFISQGKLTGYRLIAGESDGLPGVTIDKYDNFIVCQLLSAGADFHRYTIVNCLTELYPGCHIYERSDVDVRKKEGLEPVTGWLTEPQDSTECIIEEHGIKIHVDIATGHKTGFYLDQRDSRLAAGKFAKDKTVLNCFSYTSTFALHCAANNAKEVINVDVSQDALDMGERNLALNGLSDANVSFVKEDVFKLLRRYREEKRTFDMIILDPPKFVESKAQLTGACRGYKDINMIAMQLLNPGGLLLTFSCSGLMEASLFQKVVADAALDAKRKAYFVERLQQAADHPISSNYPEGYYLKGLVCQVE

>sp_Q12XG0

MKFDPESIKKAAKEDFDSAWTSGKDLIKKTGLNQQYPHTSFHFGKAHPVYDTIAKLREAYLRMGFDEMMNPLIVDEKEVYKQFGHEALAVLDRCYYLAGLPRPNVGISDQRIAKIKEMLGGIDDEGIETIRKVLHSYKKGEVEGDDLVPEIALKLNVSDALVVEMIDKVFPEFKELTPQATTKTLRSHMTSGWFISLSGILERSRPPFHLFSIDRSFRREQQEDASRLMTYYSASCVIMDEDVTVDHGKAVAQGLLAQFGFEKFMFRPDEKRSKYYVPDTQIEVFAYHPKLVGSNTKYSDGWIEIATFGIYSPTALAEYNIPCPVMNLGLGVERLAMILHDSTDLRGMTYPQLPQYAEWELKDNELARMIFVDKLPETPEGQEILEGIVMQCDMHGSEPSPCEFVAWEGILKGKKVKVSVIEPEEDTKLCGPAAYNEVLVHENDVLGLPNNKKWKKAFENHSARTGVRFIEAFAAQAAKEIEEAVEKGEKECETRVRIVKVPSEINIKLDPLAQRYITGKKQKIDIRGPVFTTVRAEIE

>sp_Q12ZB8

MDRKIVIPGQLLSEKKEMAGPGTYVKNGNIYSLLYGIANFKGRVSVVPFSGKYIPSRKDFIIVNVIDVTPSNWIMETGSPYDGLLHVSEFPKRVDSSEMRKFMDVGDCVIVRVKDVSKAMKVELSMREQGSRALSKGRIIEVVPSKVPRVIGHGGSMVSILKKESNCDVFVGKNGRIWINGKDKDMDRLTTAIEMIESESHMSGLTDKVGMFLRGEPEGTEGSDEEQLVDEEVAGVSLEDDDVTEETSRKVDVLLDNDTDETN

>sp_Q482S1

MNLHEYQAKQLFAEYGLPVSEGFACDTPQEAAEAADKIGGDMWVVKTQVHAGGRGKAGGVKLVKSKEEIKEFAQHWLGKNLVTYQTDANGQPVAKILVESCTDIANELYLGAVVDRASQRVVFMASTEGGVDIEKIAEETPELIHQAEIDPLVGAQPYQARELGFKLGLNPTQMKQFVKIFMGLAKMFEDCDFALLEINPLVITDEGNLHCLDGKIGIDGNAIYRQPKMRAFHDPSQEDEREAHAAQWELNYVALDGTVGCMVNGAGLAMGTMDIVNLHGGKPANFLDVGGGANKERVSEAFKIILSDDNVKAVLVNIFGGIVRCDMIAEGIIGAVKEVGVKVPVVVRLEGTNAELGREVLKNSGLDIIAAESLTDAATKVVAAAEGK

>sp_Q47UW2

MPRRRVVGQRKILPDPKFHNELLAKFINILMVDGKKSTAEKIVYGALDILAQKSEKDQLELFEEALDNIRPSVEVKSRRVGGSTYQVPVEVRPVRRNALAMRWLVEAARKRGEKSMAQRLANEMLDASDSKGSAVKKREDVHRMADANKAFAHYRW

>sp_Q47Y31

MRAYLDLCQRIIDQGTWVENERTGKRCLTVINADLEYNVGNNEFPLITTRKSFFKSAIAEFIGYIRGYDSAADFRKLGTKTWDANANLNDAWLNNTHRKGEDDMGRVYGIQGRAWAKPDGGTIDQLKKIVDNLKDGIDDRAEIMTFYNPGEFHMGCLRPCMHTHNFSLLGDTLHLTSFQRSCDVPLGLNFNQVQVFVFLALMAQITGKKAGMAYHKIVNAHIYEDQLPLMKEVQLKREPLALPKLIINPEIKSLEDLETWVTMDDFKVEGYECHEAIKYPFAV

>sp_Q480C7

MKKLTICSEYTPSGDQPTAIKQLLEGIESGLAHQTLLGVTGSGKTYTIANVIEKLNRPTMMLAPNKTLAAQLYGEMKEFFPDNAVEYFVSYYDYYQPEAYVPTTDTFIEKDASVNEHIEQMRLSATKALLERRDVIIIASVSAIYGLGDPDSYLKMMLHISRGDIINQRDILRRLAELQYTRNDVAFARATYRVRGDVIDIFPAESDRLALRVELFDEEIERISQFDPLTGQVERTLDRVTVYPKTHYATPKEKIIAAVDKIKIELKHRSQQLKDNNKLVEEQRLTQRTQFDIEMMTELGYCSGIENYSRYLSGREEGGAPPTLFDYLPDDGLLIIDESHVTVPQIGAMYKGDRSRKENLVEYGFRLPSALDNRPMKFEEFEAISPQTIYVSATPSKFELEKCGSDIAEQVVRPTGLLDPEIEVRPVETQVDDLLSEINKRLPLDERVLATTLTKRMAEDLTDYLYDHGIKARYLHSDVDTVERVEIIRDFRLGKFDVLVGINLLREGLDMPEVSLVAILDADKEGFLRSDRSLIQTIGRAARNLNGRAILYGDRITGSMRRAIDETERRRVKQHQYNLDNNITPQGVVRRITDVMGVGSYSDAKSLDKVAEANTNYHINQQAEEPLLTTSQIDTKIVELEKLMQGHAQNLEFEQAAAMRDKIAKLRIQQLST

>sp_Q12UF8

MKTEELYSGKAKTIYKTENPNELISEFRDSLTAFDGKKKSEATNKGYYNAQISKKIFEMLEEEGIKTHYLGMVSGNEMLVKKVDIILIEVIPRNIAAGSITRKYPVEEGTVFKEPVLVFDYKSDEFGDPMINDDIAVVMGIATREEIDFIRSMALKINAILKSYLESNGFLLPDFKLEFGRVDGEIVLADEISCDTCRFWDVETGESMDKDLFRFDKGDLSKAYEKVARRLVPEIFEE

>sp_Q47UV7

MAINLDDKKAIVAEVQEAANGAHSVVIADARGVAVEAITVLRKQARENGVWMKVVRNTLARRAVEGTEFECVSDVFKGPSLIAFSSDHPGAAARLFTDFAKANENFELKAAAFEGNAVDVNMLAKLPTYDEAISRLMSVMKEASAGKLARTFAAIRDQKEQEAA

>sp_Q47UV6

MAKLSKRARLIREKVDVTKEYDINEAVSLLKEFATANFRESVDVAVNLGIDARKSDQNVRGATVLPNGTGREVRVAVFTQGENAEKAKAAGADIVGMEDLAAQVKAGEMNFDVVIASPDAMRVVGQLGQILGPRGLMPNPKVGTVTPDVAGAVKNAKSGQIRYRNDKNGIIHTTIGKADFEPAQLQENLESLLEALKKAKPANAKGQYLKKVSLSTTMGAGVVVNQATLTQA

>sp_Q12UP9

MVEETTLDLVKQLIEGSPERKFSESLDIAINLKNLDMSQPKNRVDEEIILPNGLGKTMKIAVFAKGEVGLNAKDAGCDYILTEEDIKELGEDKSKARSLANECDFFIAEVQYMAQIGKALGAILGPRGKMPVPLTPDKNVADLINSTKNSVRIRSKDKLTFHVSVGRRDMDVEKLAENIETVLGRLEHSLEKGKHNLKSVYVTTTMGNSVRLV

>sp_Q480B2

MARVKRGVQARARHKKVLKQAKGYYGARSRVYRVAYQAVTKAGQYAYRDRRQRKRQFRQLWIARINAAARQNGLSYSKFINGLKKASIEIDRKILADIAVYDKAAFTFLVEKAQASLAA

>sp_Q12WL2

MELAVVGSSEFVTGFRLAGIKKIYEAKSDELESVVTKVLKDSDVGIFVIHEDDFNKLPEILRDTLSESVDPTVVTLGGTGESSNLREKIKQSVGVDLWK

>sp_Q12TV0

MRTLVFEPFSGASGDMILAGLIDLGADKGEIVEVIQASVDVSVTIEDITKCGIRATDVNIHTKDSARIRSFGELIDIIKDANLPEEVEKNAIAVFRIIGDAEAKVHGMSLEQLHFHEVGQDDALADVIGSCYAIHRMKVENILCTPVNVGGGSVRTAHGTLPVPVPATTEILSGSGLEVHSNGDRELLTPTGAALLTYFANPSDQLPTGKILTTGYGAGDAETDMPNVLRTMLMETTGNLSRDHMEVLETNVDDVTGEVLGNLFETLMKEGAKDVTITPATMKKGRTGHIIHVIAHPENSERIARELIRQTGTLGVRILPTKHRFIAERKMEKVNIIIGKQTFQVTVKIAHDRSDEVLHISAEFEDCRRISQECGLPLKEVIRRAEEKAWNNILKK

>sp_Q12VQ2

MLIVLSVGGSILAKNLDPKHFSAYASALKELARKHQIVVVTGGGVAARNYIDVARGVGANEVVCDFIGIDITRLNAQLLIAALGNTAHPEPPTTYKEAESALASGKIVVMGGVIPGQTTDAVAAILAEYLNADMMVIATSVDGVYSSDPREDPNAKKFDVMTAKELVGIVISTEMKAGSKSPVDPLASKIIERCNIDTIIMDGTDPQDVLEVVLQEAVKTDKVTGVRLGTRIIG

>sp_Q12U80

MKTIKEQLLSSCKPLDELLGGGFESGVVTQIFGEAGSGKTNICLQLAIECVKKGKKAIFIDTEGLSADRFKQIAGENARKIAQDIIIFEPHSFEEQYSAVRETEKISTENVGVIIVDSATAYYRFELDDEDSSIRTRRELSNQIGFLHSLARKRDIVVVITNQVYSDIKSNSLKPIGGSSLEHISKTIIQLEKTGTGSRRAKIWKHRSRPEGTTCEFTITADGVR

>sp_Q12Y46

MLSTKVTEGSTTVSVPVPPEGVPFPPSEAPVFYNPHMELNRDISVAATSACAKRILLKKDMELKDITYLDAMSASGIRGLRIANEVGITSILNDWSDDAYGLILENIGLTGVSDIAEATCKNANVLMHERRFNIVDLDPFGSPTPYLDAATRSAVHFLEVTATDTAPLCGAHFNSGMRKYAAVPLNNEFHSEMGVRILLGKIARELAKHDKGMTPLLSHATRHYVRTYLQVKKGAKLADKAFKDLGFLSHCEHCGHREIFHGMAISISDHCSSCGNDVYIAGPLWLGQLHEPQYCDEVLLELEDRSLGTKEQAKKLILACRDELDIPFFYDQHVICKMIGVSAPAMDMFIAALRETGANVSRTHFTGISFKTDAKIDTIKDVLRSL

>sp_Q487C3

MPSMDIVSETDLEEVRNAVDNANREITTRFDFRGVEASFEWKKPNIILKAEGDFQLKQMCDLLRSQLAKRNVDAKAMAVGDASASGRNWSQQVTFKEGIEQDMAKQLVKLIKGEKLKVQAAIQGEQVRVTGKKRDELQAVMQLVRTAELEQSFQFTNFKD

>sp_Q48AN9

MITLSAEQCRIIGVMLEKETTTPEQYPLSLNGITTGCNQKSNRDPVMSLSESDVQNLVDELVQMNQLMVDQKASTRVNKYFHRFCDTEFGNLKFTPQQRAVICVLFLRGPQTPGELRTRTNRLADFADVSEVDNTLTQLQDLNGLTLVRKLEREPGKRESRYVHLLSDVDESSFTQAVTTQTEVVLSEEQTSLTQRVTELEQQVASLTEQINCITELLNDD

>sp_Q484P6

MKLLSNIVFGALIAFTSVKAMAALEIVITGGVDSARPIAIIPFNWTGTGERPELLSKVISDDLLRSGKFSPIAIDRFPQTISDAKAIDYSAWANLGAEAVLLGNINEVAPGRYQVSYQLVDVIRGQITGGETSMLSNGELVNPKDHILAQSSANIQLNQARRYAHSISNVIYEKLTGSKGAFLSKIAYVIVRDQGKYPYQLAFADYDGFNEHVLLSSKEPLMSPSWHPNGDQLAYVSFENRQAQIHSIDIYTGVRKSISSFNGINSAPRFSPDGKSMAMVLSKDGNPDLYVMELATMKLRRITRNRAIDTEPSWTPDGNSLIFSSERGGKPQLYRVDLAGGKVRRLTFDGEVNLGGSVTPDGKQLIMVNRTRGKYRLAKQELSSGLFQVLTETRLDESPSVSPNGGMIIYSTLHNNRQVLGLVSVDGRFKARLPALDGQVKAPAWSPFL

>sp_Q47WK1

MGRAYQNRKLSMAKTAGAKTKVYSKYGKAIYVCAKTGGIDPDGNLSLRGLIDRAKKDQVPTHVIDNAIKKASGAGGEDFVEARYEGYGPGNCMVIIDCLTDNGNRTIKDVRQCFSKNHAKIGTSGSVSHMFDHQAVFAFKGEDDEAVLETLMMADIDVTDVELEDGIITVYAPHTEFFKIKTEFTNSMPEYELEIEELSWVPQNYIDVEGEENLENFEKFIAMLDDCDDVQHVYHNAEIKD

>sp_Q12ZU7

MAKKSTSRLPKRKGEYTFRGKTVAQLQEMSFEDFAELLPAKERRSIRRGFSDSQKGVLQQFRDGKESVRTHFRNMIIFPEMIGKNLEVYNGKEFVKIEIMPEMIGHRFGEYSPTRNRVSHGSAGVGATRSSKFVPLK

>sp_O74044

MARSPVLIINCKNYKEAAGGRIDSLAAAAAGAAAKYGVRIALAPPQHLLGAVKGEDLTVLAQHIDDKGVGSTTGYVVPELLGESGVSGALINHSEHRVSADQVASLVPRLRGLDMISVVCVKDSAEAANLSRHRPDYIAIEPPELIGSGRSVSSERPELIGEAAEAIRGADGTKLLCGAGITSGADVRKALELGSKGILVASGVVKSSDPAAAIAELAQAMS

>sp_Q12U30

MGNRPLDILNDALNTSVIVRLKGAREFRGVLQGYDVHMNLVLDEAEELKDGEIVRKIGGVVIRGDNVVYVSP

>sp_Q12ZC6

MAEQEKEATLPSKENFSEWYNGMLQIAEIMDVRYPVKGSYVWYPFGFSIRRNVYDIIRGLLDKDHQETLFPLLIPENEFMKEAEHIKGFEEEVYWVLNGGTTPLDVKLALRPTSETAIYPMYRLWVRSHADLPLKLYQIVNTFRYETKHTRPLIRLREITSFKEAHTVHATWDEAASQVEEAIRLYIEFYKRLAIPVLPSKRPSWDKFPGADYTIAVDSLMPDGKTLQVGTAHHLGDNFAKTFDIKYEDVDGEQVYAHQTCYGVSERCIATLLSTHGDDKGLVLPPEVAPTQVVIIPIIFKEPEAVLNACNDVKAELEAAGVRVTIDDSDKRPGSKYYKWEMKGVPLRIEIGPRDLKNEAAMLARRDTGEKEQVPLASIKDEVLSRFKIIQTSLLEKATSELNERIFDCSTVDDVKEKVQDGIALVPWCGEEKCGLDLDEQVGAGILGIPTDMDEDGTYKCPICSKETRTRVYVARTY

>sp_Q48AM0

MKQQIVNFPITFPITLSGQWQSPSAVSFSSLSTPLKDWLLDEGSLTARLKKHCDNFQVKVIGEQQQPCSAAESCDLIKVGEPVLVREVILYCDDVPQVFARSLLPIASLTGEEQILANLGEQPLGQVLFNNPSLQRLRLELSPFVNDSSVVALAAKLTAQHASNSPLVKASVIPKQELWGRRSIFMLENKPLMVAEVFLPDAFAYQ

>sp_Q12V29

MAKDILIHQIIEVLERAGFMVSNQCNIRPRSFDLAARQGNTLLFCKVLYNIDGLNEETAREMKSLAKYLNGYPILIGAKTRDQLLEDSVVYMRYDIPALSIQTLYDYFVENVPPLVSAAPGGLYVSIDGDVLKEARMNVSMSLGALASELGVSRRTISKYEEGQMDASIDIVLHLEEILDMALAKSIDILRSFEKELDPANVKEEAHEMKTPPNDNILNLIYTLGYDVLSTNQAPFKAVSKDTSSTFLTGVSEYSNAMVKRAHLMSSISNVIETQSVFIIEGKSRYKFVEDTVLIERDELNTIADSDDLDTLIHERAKRHKEE

>sp_Q12Z15

MKEHFQLKETIVTIVADDQSYIELAKESIAVHRLKLEEYIRSDPYFKVTLEPYECSSDAPEVVKRLVAAGNSVGIGPMSAVAGTIAALAVGSMVDAGATSAIVDNGGDIAFLNDRPIVIGIYAGQSSIRNIGFTLEPSDHIRGICTSSGTVGPSINFGCADAAVVFSDDVSLADSAATELSNATGIGHEGVENAFDTISSIINIDGAVVIQGEHMGMWGTIPQITRADMQYECITKG

>sp_Q47VS2

MTLKVPCPQCQKTVVWQASSEFRPFCSKRCQLIDLGEWAEESHKISQNIQVDTVLSEEMLDAMEDEFLLNNKFFVEPE

>sp_P84612

AFELPSLPYAIDALEPHISKETLEFHHGKHHNTYVVKLNGLIPGTKFENKSLEEIVCSSDGGVFNNAAQIWNHTFYWNSLSPNGGGAPTGAVADAINAKWGSFDAFKEALNDKAVNNFGSSWTWLVKLADGSLDIVNTSNAATPLTDDGVTPILTVDLWEHAYYIDYRNVRPDYLKGFWSLVNWEFANANFA

>sp_Q47XH8

MAKKKSKSSNSNTIALNKKARHNYSLTDKFEGGMSLQGWEIKSIRSGKVNISDCYVHIKDREAYLLGAEISPLNAASSHVVCDPNRDRKLLLNRRELDKIIAAVERDGYSLIATAMYWKACWVKLEFYLGKGKKDHDKRSDIKDREWAVDKGRLMKNKNLDR

>sp_Q47Y68

MKIWVDADACPVVIKEILFKAADRTKIAITLVANHHVRIPPSPFISFMQVSSGFDVADDEIVKRIEANDLVITSDIPLADEVIDKSGIALSPRGELYTKENIKSRLNIRDFMDTMRASGVHTGGPPALNQTDRQNFANHLDRIITQFKKTQ

>sp_Q481E4

MPIVSKYSNERVEKIIQDLLDVLVKEEVTPDLALMCLGNAVTNIIAQVPESKRVAVVDNFTKALKQSV

>sp_Q01893

MQAWVIGNWKQNPATSHDVNALLDDLCAAISTTKQMSHDNSARCQIMVAPSLIHLAAVNGRLKDTSILCAAQDVSGHSASVGAYTGECSAQQIVDAGATWTILGHSERRQYHQESNDALLQKMIHALSQDLGVVFCIGESQEQYDTKQTLTVIDNQLVVIKEFITQQPELIDALPTRLIIAYEPVWAIGTGKVPTVAEVSATHKHIKQTLAGFADSLSNMTVLYGGSVNADNANSFAADPMIDGALVGGASLKADSFLTIATAFSQASI

>sp_Q12WL3

MRLLQKFTRKSSLKQSGSSSNYAYVTARVRAMKSNLLPREVYPRLMNMGIDEITRFIEESQYKQDVDELARTYDGVDLFEHALNRNLAVTFTKLINISEGELNYLISEYLRKYDIWSIKTILRGKYCGASVEEINDSIVSAGQLSYPFLLSLSEKESYESIIDALSGTDYYPTLKEYDGTNLSDIENKLDKMYYTGLSTTVNNPKSNDSKLFSKFIRTEIDIKNLSTLFRLKNAGVEKDEIADLILEGGLHLSIKEIEKLLPLPFSEFVQSLEKYPYWEDISGIVKTEMDSLIELETQLTRSNIKSASSFSHVYPLSIVPIMDYILNKTNEVHNLRIILRGKAANLDEEIIRNQLVI

>sp_Q12UK2

MKPLIVLNLKTYLEGTGEGAVRIARACKEVGEASGIEIAIAPQFCDIYRVASQVDVPVYSQHLDGVGAGSFTGHAFAKCIKDAGAVGTLINHSECRLKLADIEASVTAAKGEGLRTIICTNNIATTAAAAALGPDYVAVEPPELIGSGIPVSKADPEVVTGSVAAVERIDPAVKVLCGAGISKGEDLKAAIELGSVGVLLASGIVKAKDPKAALEDLVSLI

>sp_Q47W31

MSTLEIIILALLQGLTEFLPISSSAHLILPSQVLGWQDQGLAFDVAVHVGTLLAVMMYFRKELGVMAVAWLGTVGVGPEKGRGGFDAKLSWWILLATIPAGLFGLLGKDFIEEHLRSALVIAMTTLLFGFLLGFADIKAGKRTEHKPMEKLGLKGAMLIGLAQAVALIPGTSRSGITMTIGLMLGLSRDNAARFSFLLSIPAIAMAGSYLTLKLILSTESVDWFAMGLGSLLAFVSAYACIHYFLILLEKLGMMPFVIYRLILGVGLLWFIL

>sp_Q12X58

MEDMDTTITLIGTRLAKEGVEFFFDGDTPECEQCKLKNTCMSLEKGKKYRVVKVRNNTLHECFVHDKGAMVVDVVKAPIFALLDSKKAIEGSKIRYQAPKCDEKLDAETYELCYPKGLRNGERCTVLKVMGTVEMEADPSITLKKVELLP

>sp_Q12TZ6

MADLIGLAIVFLIFALVAYVLGARGVAGLSMTIAKWLVIIFIVLAIITILL

>sp_Q47YH9

MEDNFRNVLIILSAIVITAIFIHGLWTLRKQKNPYKLKTSKDKADPITRDFDRKGFDQDGVGQVKVKPSAENDKINLENEAVTEHFITEGIQLPDDLSKSQIIIKNDVLGDLAGQTSKAKQEELDNNSIQELDSSLEDDWFKEDNSGQFSKGELGDELTPAPAVEKVKKKPTKPKAVHIEPLYEQPVTQAKPARTPINKVSKTPSKATLKRDQIEIDFDNQMSEQAAAPKKIKTQLEPQVIILSVVMPANQQMLGAALLPSLLTLGLKYGEMNIFHRHEDNAGKGKVTFSLANIMNPGSFDLDNMENFATRGVSLFMTLPNAGDPFSVFEQMLNAAKQLAQEFNAQVLDDKRNVMTKQTEQHYLSKIREFDRKSRIALVE

>sp_Q12ZJ1

MDDLEAIRQKRLAELQQQQSSPQNDAQAAYQQEQAQAERDEQVKAVLRQVMTPEARERLTRLRLSRKELVEQLESQLVMLAQNGRLQTKIDDEKLKVLLTQMQPQKRQTSITRM

>sp_Q483B4

MAFDTKLMEILACPVCKGKLDYDKAAQELICHFDRLAYSIEKDIPVLLENEAREINANQSTEQDG

>sp_Q487Z9

MGQKVNPTGIRLGITKPFASTWFASNKDFASNLDGDHKVREFLKEKLKRASLSKVVIERPAKSIRVTIHTARPGVVIGKKGEDVEKLRLAVSKIAGVPAQINIAEVRKPEMDAQLVADSIASQLERRVMFRRAMKRAVQNAMRLGAKGIKVQVSGRLGGADIARAEWYREGRVPLHTLRADIDYAIARGNTTYGVIGIKVWIFKGEIIGNMPLQAEVPAAKPKRKTNRKPK

>sp_Q47VY5

MAEENQVENSEAAQQSPEFAIQRIYTKDVSFETPNSPAVFQLDWKPEIQLDLDTRSTKLADNTYEVVLSVTVTATVEDKTAFLAEVQQAGIFTIGNLPEAQLAHTIGAFCPTTLFPYARETVASLVNRGSFPQFNLTPVNFEGLYASYVQQRATQENVAQSSETH

>sp_Q482G8

MSSQPLNVNEDEIAKFEQVASQWWDLTGDFKPLHQINPLRVQFISQHIALQVEGIDSKNGFYDMQIIDVGCGGGILSESLAKLGANVTGIDMGTEPLNVAKLHALETGVSINYQKITAEEKALENPGTFDVVTCMEMLEHVPDPASVIQACSTLVKPGGLIFFSTLNKSIKSYLLAILAAEKLLKIVPDGTHDHDKFIRPSQLIGWAEEHGLKCIDASGIHYNPITGNHKLNDSLDVNYILCCRKL

>sp_Q482I0

MQLFIEYFPLLIFFIINSIAGIYWATGSLIVAAFVQIFYYKIKKEKIPAKQWIIFGLIVVFGGLTIYLQNDAFLKWKVTIINAFFAAALLVSNTFFNKNIIKEFLAESLSLPENIWSRLNLAWALFFLFCSGLNYYIAFNYDLDTWVNFKVFGLTGLMFLFSITSILFLYKYLEVEEEINDTDTINNEKTKEST

>sp_Q487Z6

MAIVKCKPTSPGRRHLVKVVNKELHTGKPYAPLLDTKSKSGGRNNNGRITVRHIGGGHKQHYRIVDFKRLKDGIPAKVERLEYDPNRSANIALVLYADGERRYILAPKGLSAGDSILSGVGAPIKPGNTMPLRNIPLGSVIHAIELKPGKGAQIARAAGTYAQLVAKDGAYVTLRLRSGEMRKIESDCRATLGEIGNSEHMLRSLGKAGASRWRGVRPTVRGVAMNPVDHPHGGGEGKTSGGRHPVSPWGVPTKGYKTRSNKRTDKFIVRRRAK

>sp_Q47WM2

MTEKNEKTHKTIEQAEQEGKYIRKVRSFVKREGRLTNNQERAINDHWQTMGLNHSDGVIDAPTLFANENPVVLEIGFGMGKSLVEMAKAAPELNFIGIEVHKPGVGACISSAVEESVSNLKVCEHDAIEILADCIPDDTLTTVQLFFPDPWHKKKHHKRRIVSAEFVETIRQKLKVGGVFHMATDWENYAECMLEDMQSAPGYNNLSESNDYVPRPDSRPLTKFENRGQNLGHGVWDLQFAKKA

>sp_Q12UR3

MSVGIVSYGAYIPKFRIKVEDIARVWGDDADILSAGLMVYEKSVPDLDEDTATIAVEAARSAVLRNNIDAKRIGAVYTGSESHPYAVKPTSTIVAEAIEATPVLTAADFEFACKAGTAAMQACMGLVGSGMVDLGMAIGADVSQGAPGDALEYTAAAGGVSYIIGNKESEMIAVIEDTFSFTTDTPDFWRREGMPYPEHGGRFTGEPGYFKHVTGAANGLMEKMGTKPSDYDYAVFHQPNGKFPSRVAKMLGFTKEQIKPGLVVPWLGNTYSGSCMMGIAATLDQAKPGDRIFATAFGSGAGGDAFSFRVTDKIDEVRDAAPKVLDLLKDPVYMDYAMYAKHKGKIRLA

>sp_Q48AL9

MLKRIQQKWLAIKLITRMDKPIGTYLLLWPTYWALWIASDGWPNLQLLLVFSLGVFIMRSAGCVINDYADRKIDGEVERTKNRPLVNGMMTSGEAINLFGVLIGMAFGLVLMLSWSTIYLSVVAVLLAAIYPFMKRHTQLPQLFLGAAFSWGMIMAFSEAQGEIPLVAWLLFTANLCWTIAYDTMYAMVDRDDDVKIGVKSTAILFAENDKRVIGFLQLMTLALLWTVGDILAFGWPYQLCIIAAAGLFSYQQLLIVNRERDACFQAFLHNHWVGLVVFVGIAIEYL

>sp_Q12XB7

MTNYHVTLEAAWLVRDVETADDAIGVAISEAGKRLNPKKLDFVEVDVGTTYCPACSEPFGSVFIAANTALVGLVLEMKVFDAESDEHASRIAKSVIGKALTDVPLNVVDVEEFD

>sp_Q47Z18

MLHNTTDNVENNQTIKASTFDSEAFLRVVTEQAGVYRMYDSKQVVIYVGKAKQLKKRLASYFRKDVGSVKTQVLVKQIAAIEVTVTHTEGEALILENNYIKKYQPKYNILLRDDKSYPYLLITAHKHPKLGLHRGGKKVKGEYFGPFPTVGAVWESLRLMQKIFPIRQCEDSYYRARSRPCLQHQLGRCSAPCVDKISVDDYKEQVNLAKLFLQGKSSAVIEQLVARMELASNELHFELAAKYRDQIVTLRKVQQQQHVSGHVAELDVVGLYRDKTQVCIHLLFIRQHKILGSKSYFPTVPSESSDSEILQAFIAQHYLSNEMLSHGKVQSSIPKEIVIKESIEQVVELARLLSEQAEYDVKISTNTRSERAQYLKLAGTNAHTALVTRNSHKESMQARFVALNEVFELENGIQRIECFDISHTMGQQTVASNVVFNQEGPLKTDYRRYNVFGITPGDDYAAMAFALNKRYGKLKANPIKPEEHGALEKLPDIVFIDGGKGQLAKAEEFFSQLALTRTPLLVGVAKGESRKPGLETLILAGSHQLISLPATSPALHLVQHIRDESHRFAITGHRAKRQKVSKKSRLESIEGIGAKKRQSLLTFLGGLQEVMQADITALAKVPGISHVLAEKIHNALHDK

>sp_Q482K2

MSLNTDLTIALDVMGGDQGPLITISSAITAISHQPNLHLILCGDEIIITETLAHFEITKENLATHKQLSIFPTSQVVLMSDKPIVALRTKKDSSMRKALDLVHEGRAQACVSAGNTGALFSMAHFVLKNIPGVERPALISSLPTHDKDKHVFMLDLGANVFCDSHVLYQFGVMGSVMAEQVDGINKPRVALLNMGEEAIKGSDHIKLAALELTENKDINYVGFIEGSDIFSNKADVIVCDGFVGNVALKTCEGVARLVYEKSKTAFSASLVAKLFGSLLKPSFKKLFKTMNPDQYNGASLIGLRGIVVKSHGNANSSAFLSAIEEAVKEVERQVPEKIKTSLEHGFTCR

>sp_Q12UA1

MQFQGRSRRKYTGAKLKSARGKRKFELGREPAATHVNDTKRKNVPTHGGNRKVRLLQENIANVTNPADGKTIVSAIETVVDNAANAHYVRRNIITKGSVIQTAAGNARVTSRPGQDGVVNAILIE

>sp_Q47VT2

MADNQYYGTGRRKSSTARVFMKAGNGAITINKRDISEYFGRETARMVVRQPLELVEMLEKFDFNISVVGGGISGQAGAIRHGITRALMVFDETLRGELRKAGFVTRDARKVERKKVGLHKARKKPQFSKR

>sp_Q488Z6

MANHNQENSQQSDMAEKLIAVNRVSKVVKGGRIFSFTALTVVGDGNGRVGFGYGKAREVPAAIQKAMEKAHRNIVTIDLKGTTLQHVITGKHSGSKVFMKPASEGTGIIAGGAMRAVLEVAGVQNVLSKAYGSTNPINVVRATVSALVNMHSPEGMAAKRGKRVDEILG

>sp_Q12XP4

MLIHPEEILETIHMIKAENFDIRTVTMGINLRGCCHSDIDVFNKNIYNKITGYAKELVRTTEEVQNLYGIPITNKRIAVTPIAIVAESCNTEDYVSIAKTLDRAAEDVGIDFIGGFSALVHKGITPGDMKLINSIPQALASTKKVCASINVATTKAGINMDAVAMMGHIVKKTAEATKDADGIGCAKLVIFANAPEDNPFMAGAFHGIGEPDCVINVGVSGPGVVNSAVRELKDPDLGEISEAIKKTAFKITRMGEMVGREVSRRLNVDFGVLDLSLAPTPEIGDSVAAILEAMGLETCGTHGTTAALALLNDAVKKGGSMASSYVGGLSGAFIPVSEDAGMIRAVELGALSLEKLEAMTSVCSVGLDMIAIPGDTSAATISAIIADEMAIGMINKKTTAVRLIPAPGKKVGDSVEFGGLLGRAPVMKVSEFSSEKFIARGGRIPAPIQALTN

>sp_Q12XG1

MLGASEPEDVIKQCTQVLEHIANDNSVPRNIRRSANDILATLKNEAEPLFLRTSSSISILEDISNDPNIPLHTRTLIWNVASQLETIPVDE

>sp_Q47XR0

MMKGGMGNLMKQAQQMQAKMAKAQEELAQMEVVGEAGAGMVKVTMTGSHSVRKVELDDSLMEDDKDMIEDLLAAAVNDAVRRVEEQNKDKMGALTGGMQLPPGMKMPF

>sp_Q47WN5

MTITTSKQLPSLAQLPETYLIELSEFGAISLSGEEQSKYLQGQVTCDVNSITESNLLVGAHCDAKGKVFSVFRLINRSSAHLLLQPTASIEGSLKELKKFGVFAKVTIDIAEELGFIALIGKQASSLIQQEFSQVPDSLTPVVQIGSTSLVYLSGEQPRYIIIDDKATITAITEKLALPTYSQSVWNLLEITQGFPILTANTSGHYVPQMLNLQAINGISFTKGCYLGQETVARMQYLGKNKRALFCLNSQLEQPFQSDDVIEKQLGENWRKAGDILAHYQADDGSCVIQAILANDGDLPILRIASQADSVVTNQTLPYTLIAE

>sp_Q12TI2

MENTDQNIEITAETSAAAENTESTSLVPIDEYLAAGVHIGTQQKTQNMMKFVYRVRTDGLYVLDIQSTDERIRSIAHFLSMYDPSRILVVSARQYGQYPATMFSKSVGAVSKVGRFIPGSLTNPVQEGFFEPDVVIVTDPAGDAQVIREAVNVGIPVVALCDTNNMTSNVDLVIPTNNKGRKALSLVYWLLAREIANERDIPFNYEASEFETGL

>sp_Q12ZT6

MVLLDPLADALSIIKNAEAVGKDSCTIRPASKLIGNVLKVMNDRGYIGDFEFVEDGKAGVYTVELIGRINKCGAIKPRYSVGVTEFERWEKQFLPAKNFGVLILTTPKGVISQYEARENNVGGQLLSFVY

>sp_Q47Z40

MSDTENRPTNFIRNIIDADLDSGKHTGVQTRFPPEPNGFLHIGHAKAICLNFGIAQDYNGLCNLRFDDTNPEKEDIDYVHAIQKDVKWLGFEWAGEIHYSSNYFDQLHGFAVELIEKGLAYVCFLNAEETREYRGTLNKPGKNSPYRDTSVEENLALFAKMKNGEFEEGICALRAKIDMTSSFMCLRDPIIYRVRFAHHHQTGDKWCIYPMYDFTHCLSDALEGITHSICTLEFQDNRRLYDWVIEQVSVPSTPHQYEFSRLNLEYTLMSKRKLNTLVEEKLVDSWDDPRMPTIAAFRRRGYTPASMREFAKRIGVTKMENTIEMSVLEACIREDLNDNAPRAMAVLDPIKLVIENYPEDKNEDLIVKNHPSDDEQGTRIVPFSKELYIEAEDFREEANKKYKRLVIDKAVRLRGAYVVTATRCDKDEQGNVTTVYCTYNEDTLGKNPTDGTKPKGVIHWVDANKSLDAIVRLYDRLFTVPNPAAADDFNSVINPQSLVTITGAKVEPSLAEAKPEFAYQFERQGYFCLDNDIKEPGALVFNRTVGLRDTWAKISQ

>sp_Q47ZU5

MTEAQLTLPTTKLSPTQKTQFIKLEKKLRRNVGQAIAQYNMIEDGDKVMVCLSGGKDSYAMLSILMLLKESAPIHFDIIAVNLDQKQPGFPEHILPEYLDKLGIEYHIVEEDTYGIVKEKVPEGKTTCSLCSRLRRAVLYKAAKKIGATKIALGHHRDDMIETLMLNMFYGGKMKAMPAKLVSDNGEHVVIRPLAFCKESELIQYSELKHFPIIPCNLCGSQPNMQRQNIKRMLNDWHDQFPGRIESMFTAMQNVVPSHLCDSNLFDFKSINSTSGIINGGDTAFDEVAIEAPQDLSMKNQPNADQLLNVVEVK

>sp_Q12W02

MRPDISNIPELPGVYLMKDISDNIIYIGKAKSLKKRVSQYFQSSKNHSSKTRAMVRKIADVDYIVTESEVAALILEANLVKKNRPHYNIDLKDDKRYPYVKVTVNTKFPKIFITRRRLMDGALYFGPYTNVKPVRQTLDMISQIFRIKRCNRRVDGKGKRACLNYHIDRCYAPCNGSITPEEYRNNVMEAVKLFKGETSGTIKELQEKMNIHAIAQEYESAAVIRDQIDALKSLSRQQTATAGNDDSDIIATASDDETVFVQIFYIRDGNMVGKADMSLSWGDATGNIARVTEEFIKQYYQDAPVPPEILVQYPIPEKELVIKWLSEKAARSVQIQVPQRGNKKRLMEMAEQNAQMTLEQSHLKQSDKEQALQALLQLRDALALSTLPAHIEGFDISNISGTDAVGSMVVFENGLPANSKYRHFNIKTVKGIDDFAMMAEVVKRRYTHQKAEKDKLPDLILIDGGPGQVSAAMGSLKELQLDIPLVGLAKRFEHIIVPKDGTDEVVILPHTSEALKVLMRVRDESHRFAVSSHRRRRTARLSHSELDTIPGIGASRKKALLNHFSSIEQIRHASVEELTAVEGISKGLAERIVAHFNKERVENQ

>sp_Q12VJ7

MRVALKIAYIGSNFHGSQVQLNDPTVEGELFKVLKELGIMEDPRTANFISSGRTDSGVHAMGQVVAFDTDAPNLAMPRVINSKLPGTIWAWAHAIVPENFDPRRHALSRSYRYILYGEQFDISKIRSASKLLLGSHDFSNFSTSRGSKKTVRIVKRIDIRVSGNLTRIDVEANSFLWNMVRKIVAALMMVGSGVRDEEWLGHMLDPESYEEGLEPAHGYGLVLMDVNYPIPLEWVEDGYAIRRARERVHDHLVRYRVMADILSHLFPSESSDELL

>sp_Q12WL4

MGLETVIKDIMSAAQTEVNVINADADAEVSQILDDARQTAKKIMGDRLAKAEDDIKRLRQQEISSANLEVKRAMLNARKEVLDKVYNNAIDSIVSLPGSKQEELLKAIIDENDSNGSNIYSNKDSEKLVRKLSSLEYAGNIDCIGGLTIENSDGTVRLDYTYDMILKNVNEQSLKQTSDILFG

>sp_Q12WB4

MLSASEGEQTVRLARNTIESFLKDGEQSDSIDLPEVFGELRGVFVTLTKNGNLRGCIGHPYADSVLESAIVDSAISAATRDPRFPMVDISEMSDIIVEVTVLTQPELVDVLPDKLPEVIEIGRHGLIAKMGMYQGLLLPQVAPENDFDAIDLLNHTCLKAGLPQDAWLTGAQMYWFEGQIFKEVEPRGDIEENKFNSCCK

>sp_Q486M1

MSTKTKSPIGQRTVIGFDFGKKYIGVAVGQEMTGSATPLGSVKATDGIPHWDNLAKYLKEWQPDFIVVGLPLNMDGSEQQLTLDAKKFGNRIHGRFGIQVEFQDERLTTADAKEQLFARGGFKNLKKDNIDAESARLIIESYFEQQYT

>sp_Q12ZP8

MYNLYCTYMTGKRENVLIEALPYIREFHDSVMVIKVGGHAMVDPQVMSDIVQDIVLLRFVGIHPVIVHGGGPEITEKMDRMGKKSEFIGGLRITDDETMEIARMVLVGNINTRIVSLISKHGGKGVGLSGKDGNMILAKKKPTQKILIEDIEHDVDLGWVGDTEIINPEIINIVTANGYIPVISPIAMDSEGNALNINADTVAGDLADALNAKKLILMTDVPGVLRDQTDISTRISRIGVDEVEQLIEDGVLSGGMIPKMRSAKASVEGGVDRVHVIDGSISHSVLLELFTDQGIGTMVYKDTK

>sp_Q9K4Z5

MTMTATMTTPLVLKLGGALLENETALEQLFTALSEYKSTSSRPLVLVHGGGCFVDELLAKMNIVSEKKNGLRITPFSDIGYITGALAGTANKVLMAQGLKSGAKVVGLSLADGGIATVTQSTAGLGAVGECEAGDPTLLTALLSGGFLPIISSIGIDAQGQLLNVNADQAATAICETLDADLVMLSDVAGILDADMQLIPEMNSNYAAELIAAGVINGGMEVKVKAALKAAASLNRDIKLASWKVPERLVALLNGEVEGTKVSS

>sp_Q9K4Z6

MLKTILVGATGYTGAELAHYITKHPELELAGLYVSEHSLDAGKPFSSLYGHLLGVVDQTIEPLAVSNIKNICDDVDIVVLATAHEVSHDIAAEFLAQDTVVFDLSGAFRVNDPAFYENYYGFKHNFDKELQSAVYGLAEWASADIAEANLIAVPGCYPTASLSALKPLAKHGLISTDQKPIINAVSGVSGAGRKASLASAFCEVSHAPYGVFNHRHQPEISTHLGHEVIFTPHLGSFKRGILATINVKLAAGVTPEQVTAAYQEAYQDQPMVRLLPSGWPSIKAVEKTAYCDLAWQQQGQDLIVVSAIDNLLKGAAAQAMQCINIRFGFAMTTSLV

>sp_Q12Z64

MQLKVQPIDVKVGKYKVILNTIDAKELGVHEGDRVRIKNHVTLTAIVDFTEDMISPGMIGLYHEVKEALSKEWTETVEVFPAEKPKSTYIIRKTMDGQKLTKEEIDILVKDIVEENLAEIEIAAFLTATYINDMTDDETEWLTRAMIDSGDKLEFDTHPIMDKHSIGGVPGNKISLLIVPIVAANGLLIPKTSSRAITGAGGTADLMEILAPVEFDAAEIKRMTEEVGGVLVWGGATNIAPADDKLIKVEYPLSIDPHCQMLASIMAKKGAIGADHVVMDIPTGPGTKIKNVQEGRKLARDLINLGDRLGMDVDCALTYGASPVGRTIGPALEVIEALKVLESFEGPNSLIEKSASLAGMLLEMGNVAGKDKGYDLAIETLKNGKALTKFKEIIKIQGGNPDVTHKDISVGEFTEDIIAPNNGYILEMDNKRLVQIARLAGAPNDKGAGILLHRKQGEPLKEGDPVMTIYAEKKSKLENAVKSAKERPPFIVEGMMLERIQSFKEI

>sp_Q12X18

MKVTVGRSGVHGEIFAPASKSYTHRAITVAALSKESIIHRPLISADTQSTIKACEMLGAYIEKDGDKLLISGVDGEPQTPDNVIDVGNSGTTLRFMTAIAALGQGTTVLTGDNSIRSRPNGPLLQVLNDLGVQSISTRGDGCAPIVVTGGLKGAIAKIDGSISSQFISALLLACPLTKNSTTLSIKGELKSRPYVDVTLDILEKAGAEIYLEDNQNLKFIIPGNQKYRLKEYTVPGDFSSASYLLAAAAMTDTKIKVNNLYPSMQGDAAIIDILKEMGANIYWNKEEGTVEVNGGKLHGITMDAGATPDLVPTVAVLGAVAEGETVITNAEHVRYKETDRLHAMAVELDKMGISTSEEKDKLTIKGGELKGADVHGWHDHRIVMSLTLAGMIAGDTTIDTAEAIFISYPNFFDSMRSIGADVILSEQ

>sp_Q482G5

MEQLTLNPIGKINGEIFLPGSKSLSNRALLIAALANGVTKITNLLVSDDINHMLNALKSLGIEYTLSDCGTECTVIGNGGFFNAKKPLELYLGNAGTAMRPLCAALAASEGEFILTGEPRMKERPIGHLVDALAQLDADIEYLENKDYPPVKIKGKALTGNTVTIDGSISSQFLTAILMIAPLLETNTTIEIDGELVSKPYIDITLDIMRRFNVSVQNNDYKSFIVNGKQSYQALDKYMVEGDASSASYFLAAGAIKGGEVTVHGIGKLSVQGDKHFADVLEKMGAEIHWKDESITVIGKPLTAVDMDMNHIPDAAMTIATTALFATGTTTIRNIYNWRVKETDRLNAMATELRKVGAEVVEGKDYISITPPKSLKHAEIDTYNDHRVAMCFSLVALSDTPVTINDPKCTAKTFPDYFDKLAQVSC

>sp_Q12XK2

MTFTGQAYALGAGTVINAIATWKGAAFGVDLKTFADVELTKESSSFIGTIEGVPHGDTTLIERSMDLVLEHFGIEMGGTVVTRSEVPLASGLKSSSAAANATILATLDALGETLEPLDAVKMGVRAAKDAGVTITGAFDDACASFFGGIVVTDNRTNELVKRTEKEMDVVIFAPDRQSFSSQTNVHNSELLAPWVDMAYDLALDGEYEKAMTLNGFLYCGALGFSTDVMMEALKCGVKGVSLSGTGPAYSALVDRKMADTLTKVWENLGTSGKVINTKINNDGLTKL

>sp_Q48AW1

MAVGKEIKTKIASVKNTQKITSAMEMVAASKMRKAQEGMAASRPYATNIRNVIGHIALGNLEYRHPYMEERETKRVGYIVVSTDRGLCGGLNINLFKKVLADAAEKQASGAEVEFGVVGSKATSFFNNMGAKVSAQISGLGDSPSLTDLVGSVAVMLKAYDNGEIDKLYVVYNKFVNTMTQDATIDQLLPLPKSDDEEISHRWDYIYEPDANSLLDKLLVRYIESQVYQGVVENIACEQAARMVAMKAATDNAGDLIDDLQLVYNKARQAAITQELGEIVAGAAAVG

>sp_Q483A0

MNLTHLKTLEAESIHIFREVAAEFDNPVMLYSVGKDSAVLLHLARKAFAPGKIPFPLLHVDTNWKFKEMIAFRDQMAKDYDFELLVHKNPEGIEMGMGPFTHGSATHTDVMKTQGLKQALNKYGFDAAFGGARRDEEKSRAKERVYSFRDENHRWDPKSQRPELWNIYNGKVNKGESIRVFPLSNWTELDIWQYIYLESIPIVPLYLAEKRPVVERDGTLIMVDDDRMPIGEDEEVQMKSVRFRTLGCYPLTGAVESTANTLPEIIQEMLLTKTSERQGRVIDHDSAGSMEKKKMEGYF

>sp_Q12ZG2

MFEGVLPALITPFTKDDTIDRTGLIKNIEFAENGKVTGVVVCGTTGESATLSTAEHMEVIDIAVECANVPVVAGTGSNNTAEAVELTKHAEEAGASGALVISPYYNKPNKAGLISHFRTIAEAVEIPIVLYNVPSRTGQDISLEVITELAKIDNIVGIKEASGNLDKASQIIENTMDEDFKVTSGDDGLTLPIMSIGGCGVISVVANIVPDRMSRLVNAFNEGDTATAQQLHYEIAPLIRALFTETNPVPIKRAMNLVGLNAGHLRPPLAPISAENNKLLANCLKELGCLQ

>sp_Q485H5

MTDLKHLASVIEQAFEERASISPATVSSEIKNAVLDALAALNNGSARVAEKVDGSWHVNQWLKKAVLLSFRIWDNQVIDGAESTFFDKVPMKYEGYTQAMFEADGVRVVPGASVRTGSFIGKNVVVMPSFVNIGAFVDEGCMVDAWATVGSCAQIGKNVHLSGGVGIGGVLEPLQAGPTIIEDNCFIGARSEIVEGVIVEEGAVISMGVYIGQSTRIFDRETGEVHYGRVPAGSVVVPGNLPSACGTYSLYAAIIVKKVDAKTLAKVGINELLRAVSEE

>sp_Q47XK5

MSWIEKILPKAKTTQKSSIPEGVWSKCSSCNAVLYKAELERQISVCPKCDHHMRISARKRIDSFLDHDNRMELGEEFEAQDILKFKDSKRYKDRLSAAQKNTGEKDALVVMKGELHGMPVVVAAFEFAFLGGSMASVVGARFVKGVEYCLEHNLPFICFSASGGARMQEALFSLMQMAKTSAALAKMSEKGLPYVSVLTDPTMGGVSASLAMLGDINVAEPKALIGFAGPRVIEQTVREKLPEGFQRSEFLLEKGAIDMIIDRREMKITLARMLGKFMGQTSVVS

>sp_Q47Y90

MTCASNSNSFLNKAIEFPSPAKINLFLHIVGQREDGYHNLETLFQFIDHSDTLTLTATETPDIELLTPIDGVNNDDNLIVKAARLLKNRSNTDLGVKISINKILPMGGGLGGGSSNAATVLVALNLLWQCEFSLSELSSLGLSLGADVPIFIHGFSAFAQGVGDHLTAIKPQESWYLITKPECSISTKEIFTAVDLPRNTKKLDPTALNTSDFVTESFHNDCQTLVIKQYPEVAKLLAWLVEYAPSRMTGTGACVFTQFSSYQEARSLQAKLPKGISSFVAQGLNKSPLCSVIAKLSLSE

>sp_Q12ZS5

MLLTISGLPGSGTTTVGKLLAEHYSVDIISAGDVFRGLAKERGVTLAEFGRLAESDPSIDVEIDKRQSDIANSSDNLILEGRLAGQMAKKALKIWIKAPLEVRVKRIVDREGSSFDVRMQETVEREASEALRYKEIHSIDIHDLSVYDLVIDSSRWDQFVITDMLKKAIDASGSF

>sp_Q47VQ0

MFRGTSAITLDSKNRITIPTKYREELFADCQGKMVCTVDIQHPCLLLYPLPEWEEIELKLCNLSSMNPQERLLQQVILGNASDCEMDKNGRLLINGPLRQHASLEKNVMLVGQLKKFEIWHDTAWQSQMLQGISKIQSGEIELTERLLDLSL

>sp_Q12U18

MFKATIDAYLLKDSIETLSVLVDEARFRISPEGVVVRAVDPANVAMVSFDLTPEAFDDFEANDCELGLDLSRINDILGVADRDDKVQMELDEESKKLKIQIGGFSYTISLLDPSTIRAEPRIPQLELPAEIVLNGKDLQKAVKAAEKISDHMLLGVEGESFFMEAEGDTDRVKLTMTRDQLIDIKPSQVRSLFSLDYLSDIIKPASKSNEISLHLGNDFPIKINFSIANGKGTIGYLLAPRIESD

>sp_Q12UL8

MNEKHLTYADSGVDIEKEESTIKALTNGMTYKREGIGAPLTSIGHYAGLIDFGEYALAMATDGVGSKVLIANEMKRWNTVGIDCIAMNVNDLLAIGAEPISFVDYLALEKHSDDFASQIGEGLVKGAEISRMSIVGGETATLPEIVNGFDLAGTCLGMVKKEEVITGEKVRLGDVLVGIPSNGVHSNGYTLVRDIIKESGHSYHEDFSYNTETTIGDELLIPTRIYMEVLDVIKECDVHGLAHITGSGLLKLKRVTGLGFDFTDPIEPGNIFKFLQEEGNVDDLEMYRTFNMGMGFLIILPEADAEKAAEMTGGKIVGKIVESGIRVRDLEIV

>sp_Q12ZN9

MSSDDQHIREIRVHPIENGTVIDHINAGQALNVLKILNIPTSSSRVVSVLINAPSVHGRKDVVKIEGRELNVEEVDKIALIAPNATINIIRDFEVSDKDIVHIHSQIEGVVRCINPNCISNSNEPVTSKFAVSSNGQRTILRCSYCERIISDDIGEHLL

>sp_Q12XL7

MSELTTGRFSISDLDNVQITINNIVGAIEKQSDDIDVEMGPTVKPGVSSLRDWDHNILDRYNPVYTPMCDQCCYCTFGPCDLSGNKEGACGINLEGHNAREFMLRVITGAAAHSGHGRHLLHHLIGLYGKDHPLDVGATNIIAPNVQLVTGVQPKTLGDLDSVLSYVEEQITQLLAAIHVGQEGAAIDFESKALHGGMIDHVGMEISDIAQISCLDFPKSDEEAPLADIGMGCLDASKPTLIVIGHNVAAVTDIIDYMEDKGLNDKIELGGLCCTALDMTRYKTGDRTLPRAKVVGTLAKELKTIRSGIPDVIIVDEQCIRADVLKEASKLMIPVITTNDKVMYGLKDRSNDSIEDILEDLTTGKEKGALMFDYVKLGELAPRLTMMMSEIRKQKGIKALPTDEELKELADSCVHCLKCEVACPNSLPISEAMTALSEGDLSKFELLHDKCIACGRCEYACPKDIDIVNVIEKSSQRVISEEVGKVRVGRGPISDPEIREEGVNLVLGTTPGIVALVGCSNYPDGTKDLFTIADEMLRRSYIVVVSGCSAMDLGMYKGEDGLTLYEKYPSRFKSGGLLNTGSCVSNAHITGAVIKVASIFAQKNISGNYEEIADYTLNRVGAVGVAWGAYSQKAASIGTGCSRLGIPVILGPHGSKYRRALIAKPYEEEKWKVYDARNGSEMQIPAAPDYLLTTAETVEEMMPMLAKSCIRPSDNNMGRMIKLTHYMELSQKYLGIMPEDWYKFVRTETDLPLAKREKLLKILEEEHGWEIDWKRKKILSGPTMKSDVSAQPTNLKRLCKEA

>sp_Q48AV5

MTQQTPDQYRVFGNPIEQSKSPAIHHIFADKSQQNIDYQKQLVDTKDFSNAVADFIRHGGKGANVTAPFKEQALAIADELTERATLAGAVNTLTFKNGKIFGDNTDGEGLVQDLITNKVILNESRVLLLGAGGAARGVLLPLLAQNPRSIVIANRTASKAATLCQHFSDIRLSASGYQDLEQQHFDVIINATSASLSGNLPPIPTSLLSQNVVCYDMVYGKDETPFLKWAKEHGAMKVIDGLGMLVGQAAVSFEVWRGVTPEVQPVIDKLRASLK

>sp_Q48AW6

MSSGAVLTSQEYITHHLSNLKVGEGFWAVHLDSLGWSVFLGLVFLTIFRSVAKKATSGVPGKLQCAVEMVVGFVDDSVKSTFHGKNALIAPLSLTIFVWILLMNSMDWVPVDLLPHLIHWVTGMPLGDIYMKPVPTADPNITFGLALGVFILIIYYSIKVKGVGGFMKELTTQPFGHWSLYPVNFILETVTMLARPLSLALRLFGNLYAGELIFLLIATIGVFQLPVHFLWAAFHLLVIPLQAFIFMMLTIVYLSLAHEDH

>sp_Q48AW3

MSELTTVARPYAKAAFEFAVEAKAIDSWLVQLTFAAEVAKDETIKGFLSSGASVEQAQTLFLNVCGEQVDSQGQNFLKVMAINERLLVLPQVLEQFIALKADFDQEVSVDVTSAVEVTAEQKTTLSAALEKRLARKVKLNCFVDASIVSGLVIKAGDMVIDGSIKGKLNRLATTLQS

>sp_Q48AB7

MPSVINTGHHHFSISNIFANWRAYYDITKPKVVALLVLTALVGMSLSVPGALPWQRLIPAMLGIGLLSSAAAAINHIVDEKIDTVMGRTHNRPLPAGKISVTNAIVFATSIALLGFIILYALVNPLTAFLTLAGLVGYSFVYTMYLKRATPQNITIGGLAGAIPPLLGWTAMTNEVVPNALLLVLIIFTWTPPHFWALAIHRKNDYAKVNIPMLPVTHGVSFTKTQILLYTVLLFVVCLLPYLVGMSNWLYLIGACSLNLIFFGYAWQLKFNAKEGTAMATFKFSIIHLMLLFIILLLDHYWLPMG

>sp_Q47ZU7

MITLLHIDTSARRTDNDVKEYNSISKSLAAHFMDKWITLNSKDKVIYRDLGLNPPDFISQDWIAAVFTPDEKQSEEQKSLLTLSDTLIDEVDQADIIVISSPMYNYGMPAVLKAWFDQVVRINKTFTFDLARGDFPIEPIMSGKKLILISSSGEFGFEIGGIREKMNYLAPHVETASKYLGVEEFYEIKSEYQEFADARHEESLSNAYRGVEELVKQLV

>sp_Q47UW8

MSNNFTKVENPVLIVEGKQADNERLKAESDYLRGTIKDDLQDRMTGGFTSDNFQLIRTHGMYQQDDRDIRAERQKQKLEPLHNVMLRARLPGGIINPTQWLAIDKFADDYTSYGSIRLTTRQTFQFHGVLKPNIKLMHQTLNSVGLDSIATAGDVNRNVLCTSNPVESALHQEAYEWATKISEHLLPKTRAYAEIWLDEEKVETTEADEIEPILGSNYLPRKFKTTVVIPPNNDIDVHANDLNFVAISEGGELIGFNVLVGGGLAMTHGDKATYPRCADDFGFIPKEHTLAIAAAVVTTQRDWGNRVNRKNAKTKYTLDRVGVDTFKAEVERRAGIKFSESRSYEFTHRGDSFGWVEGIDGKNHLTLFIENGRILDFNGANSDSKALKTGMREIAKIHKGDFRLTANQNLIVAGVSAEDKTIIEQLAREHGLINDGVSNQRKSSMACVAFPTCPLAMAEAERYLPGLVDDVEAILEKNGLKDDSIILRVTGCPNGCGRAMLAEIGLVGKGPGKYNMYLGSDLAGSRVPKLYKENVDEAGVLSEIDALSARWSAERNDGEAFGDFVIRAGIVEQVIVSFRDFHHA

>sp_Q12TN0

MKLFGSSGIRGITNKEVTTDLALKVGLALGKTKRSAVVGRDPRIAGEMIEHAIISGLLSAGCDVVRIGMVSTPTLAYATKDYDCGVMITASHNPAEYVGIKLWNPDGMAFDSSQQEEIEECIEKEDFEPVNWDQIGNVSEDANAIRQHTNMILQNVKRSSKRVIIDCGCGAGSTITPYVLRKMGCEVITLNSQPDGYFPARNPEPNDTNLTLLKIAVKEFGADIGIAQDGDADRMMAIDEKGEFITGDEMLALFARHECDEGAIIVVPVDTSMMVDDALPGSTVIRTRVGDVYVAEEIKRCNADIGGEPSGSWIFPKISYCPDGIFASAKIMELIENRTLSELKKELPHYPTYRGTVKCDNERKAHVMEVVHSKLEKCGKISDIDGIRVEMDNGWVLVRPSGTEPKIRITAEAREGADRLFSMAENIIKEALN

>sp_Q12WD5

MNTTDDVSCAEIYVSGRVQGVYFRGFTQKTATSLGLMGYAQNLPDGRVKVVAQGKRSCISELLDHLHIGPELSNVECIEVGWMGLSDTFTDFFIKR

>sp_Q47VK9

MSGLQKQEALVQEFKDLLKQEQFGSQGEIVDALKANGFDNISQSKISRMLSKFGAVRTRNARQEMVYCLPAELGVPTAQSPLKQLVLEIEHNEVMIIIQTSPGAAQLIARLLDSLSKSDGVLGTIAGDDTIFIAPSDVSKINETIKKLEQLFSKNLT

>sp_Q9K3D6

MALWGGRFSQAADARFKSFNDSLRFDYRLAEQDITGSVAWSKALVSVGILTQDEQLTIEAALNDLKLAVLENPEQILQSDAEDIHSWVETQLIAKVGDLGKKLHTGRSRNDQVATDLKLWCKQQGQQLLMQLDKTQQQLVSLAREHQHTVLPGYTHLQRAQPVTFSHWCLAYVEMLERDFSRLTDCLKRLDTCPLGSGALAGTAYPMDRTELAHSLGFGSATLNSLDSVSDRDHVMELMCTASMSMIHLSRLAEDLIFYNSGESNFIELADAVTSGSSLMPQKKNPDALELIRGKTGRVFGSLSAMLMTLKALPLAYNKDMQEDKEGLFDALDTWSDCLEMAAMSLVGMKINEARTKEAALGGYSNATELADYLVAKGVPFRDSHHIVGEAVVAAIAKGVPLEALTLAEFKAFDVLIEDDVYHHLSLDETLAKRKALGGVSPVQVEFALTNAEKRLEERDTSGISIRAARLTDLDDIERMVNYWANIGENLPRSRSDLVKAVGTFAVTEKHNQVTGCASIYVYDTGLAELRSLGIEPGYQGGGQGKAVVEYMLRKAEQMAIQKVFVLTRVPEFFMKLGFRSTSKSMLPEKVLKDCDMCPRQHACDEVALEFKLNVVGQTINLKAEKLAS

>sp_Q47V03

MINNKTVAEGRYYIGLMSGTSADGIDLALVDFTDKGQQPRLVASFYQSYSAIIADKITSLYQPGSNEIDRAFHLDVELAQLFSQAINALLNQEKLTPEDIIAIGNHGQTIRHRPSGDNPFTLQIGCCQTLATLTGIRVVGQFRRKDMALGGQGAPLVPIFHQQLFTQTTAANFVVNIGGIANITFLPTRDSNQAVLGFDTGPGNALLDDWFTKHHPNSDDCFDKNGAWATTGQVIPLLLEQLMQDDYINSAAPKSTGREYFHLEWLEQQLTAFKEATHSQHETVINHNADIQATLLAFTAQSISDAIMALTAQGKVYLCGGGVHNKALVEALSSRLTASDTVFEINTMQALNIDGDILEAMAFAWLAYAFDQGLDSNLPAVTGASASCTLGSAFLP

>sp_Q47VJ7

MAIYLVGDIQGCFNELSSLLLQVNFDRNNDVLYLAGDLVARGPNSLETLRFVKSLGESAKVVLGNHDLHLLSVHAGIKKAKKSDNLSALLAAPDVNELMDWLAAQPLLQEIPNTCSNSNAINQANNNSAYMSHAGISPQWQLSVALEQAKFIQTKLASSDRNTWLALMYGEKPNDWHQAITEIERFRYSINAFTRMRFCFTDGTLEFEQKDSPENITLTNIVPWYELSQTINNTSWVFGHWASLMGKSSHPNIYPLDTGCVWGNQLTMLRWHDKKYFIQSSELSD

>sp_Q12UJ9

MKKIFAVLGDPIEHSLSPIMHNSAFEALDMDCTYHAFRVEKNDLENALQGAKAMGFGGLNLTVPLKETALKFVDADSLAAKIGAINTIDFKDGIKGYNTDGIGAKRTIEDEGVDIKDKNVLILGAGGAARAIAFTFAEAGANVNIANRTPERAMQLAAEIGDAKGYGLDIVDNGLEDIDILINTTTVGLGNSNGTLVTAEQMHSDLAVFDIVYNPLMTKLLQEAETAGARPITGIMMLVYQGAEAFRIWTGKEPPINVMKKTVMETLDI

>sp_Q47XX0

MSAPKVQLLLTGNELMTGDIVDSNSAMMAQVLKDIGLGVNRKVTVADDLALLVNEITYMASTSDILIINGGLGPTVDDLTAQALALAIEDELSQHPQALTHLTNWCHQRGAELNGPNLKQAILPKSCQIIANKNGSAVGFYVRFNHCDIYCTPGVPHELETMLIKQIVPAISADLPSDLITDVTRLQVFGLGESSLQKIINEQLPQWPTAIDLGFRAGMPLLEVKLTTNTKKGLALKPIWHNKLADVLGDHLISEIQDKPKSLAEHLLHQLQQHNLKVTTAESCTGGLIASKLTEISGSSMNFEAGYVTYSNKMKTAMLDVPAKLFEQYGAVSEQVVVAMAKGSLIKSTADLTIAVSGVAGPNGGTEEKPVGTVWLAWGSIDNIKTQCLLLPYKRVKFQEFVAAIGLDLLRRYQQNITSIPNYIAERAFTDQ

>sp_Q483C9

MHDSDTDLIYSQAHNQVKNFTFDAQVVEVFPDMISRSVPGYKTIIDTIGRLSERFTQDDSNIYDLGCSLGAATLAMRKGITANNCKIIGVDNSIDMVKRCKMHVDAFKGDTPVTIIEGNIQDIDIENASMVVLNFTLQFIEKSQRQALLSKIAQGLKPGGLLVLSEKISSDDNVIDDVLINLHHNFKRDNGYSELEVAQKRSALEKVMLTDSLDVHKERLTQAGFQHVSLWFQCFNFTSLIAIK

>sp_Q480C4

MSNWKELFDTEGIVEESVKEQIEEPAKYYVFLLNDDYTPMDFVVDVLCNFFNKSEEQATDIMLTIHYKGKALCGTYTAEIAETKVEQVVQYAFENQHPLKCVMTKA

>sp_O93637

MGRRKKMVERVTALMSNPVMIRNIAIIAHIDHGKTTLSDNLLAGAGMISKDLAGRQLFMDSDEEEQERGITIDSANVSMVHEFEDEEYLINLIDTPGHVDFGGDVTRAMRAVDGAVVVIDAVEGTMPQTETVLRQALKEHVRPVLFINKVDRLINELQVDAQEMQIRLGKLIDHVNKLIKGMNEERYNQGWRVDAAEGTVAFGSALYNWAISVPMMQKTGVSFGEVFDYCRAEDMKSLGEKCPLHEAVNDMVIRFLPSPIDAQEDRVGVIWHGDLEAGIGKQMAVADATGDLAFMVTDISMDPHAGEVSTGRLFSGSLSRGMEVYVSGAAKPNRIQQVGVFMGPERLEVDKIPAGNIAAVTGLRDAIVGSTVTTLDGMSPFESIRHASEPVVTVAVEAKHMKDLPKLVEVLRQVAKEDPTLKITLDEETGEHLMAGMGELHLEVIAHRIERDKGVEISTTPPIVVYRETITGTAGPVEGKSPNRHNRFYVVVEPLEPEVRELIREGEISMRMPELERREKLIAAGLDKDEAKRIADIFESNAYFDMTKGIQHLNETMELVLEGFVEVMKAGPLSKEPCMGVKVKLMDAKLHEDAVHRGPAQVIPASRQAIQAAMLMADDTLFEPYQKVFIQTPQEQMGGATKEIQGRRGIIIDMTSEGDTTIIESKAPVSELFGFAGDIRSATEGRAMWSTEFVGFEPLPTNMITEVVSGIRERKGLKKDLPQAQDFMSM

>sp_Q48AR8

MLVNFSKMHGLGNDFLVLDNVTQNVFLSPEQITKFANRNFGVGFDQLLVVEPPYDPDLDFHYRIYNADGSEVGQCGNGARCFAKFVRMKGLCNKHKIKVSTSTGKMNLHIERDGNISVTMPVPQFEPKKIPFTAQKTEGTYILRSESETVLCGAVSMGNPHCVVTVDSVAEADVESLGKELSVHERFPEDANVGFMEIVSPNYIKLRVYERGAAETLACGSGACAAVVIGYMQKKLAKQVTVELPGGKLRIFWQGPGHPVKMSGPATHVFDGQISI

>sp_Q8L3D3

MSKRDYYETLEVSQDASEKEIKKAYKKLAMKYHPDRTQGDKSKEETFKEVKEAYEILNDDQKRAAYDQYGHAAFEQGGHGGGGGGHGGGFGQDFGDIFGDIFGGGGGGGRGRQRQQRGSDLRYNVELSLEDAVKGKSLEIKVPTYVSCEPCDGSGAKKGTSAKTCSTCHGHGQVQMRQGLFAVQQTCPTCSGKGKVIADKCTSCRGQGRVEKTKTLSVKIPAGVDTGDRIRLSGEGEAGEHGAPAGDLYVQVNVRDHDIFVRDENHLYCEVPISFVTASLGGEIEVPTLGGKVKLKVPKETQTGKMFRLRGKGVKSVRSTSTGDLMCKVVIETPVNLSGDQADLLRQLEEKMASSSKKHSPKETGFFDGVKKFFDDLKS

>sp_Q483D3

MDIKKLSSLAHCAKTRSIVSLFDQKERANDFSLSTSHLYLDYSKQNITDVELEQLIEIAEDVGLSESITGQFNGDKINNTEGRSVLHTILRAPQVIKQQILGDTLANEVEAAELQMAKVVNDVQKGILTSHTGQRFTDVLAIGIGGSYYGVKVSLSALEHYRDLALSVHVIANVDGGALEEKLKTLNFETTLVVVISKTFTTQETMLNAKAVKQWMLSCASVKDLELNNVPLIIEKQWFAVSSNIEAAKEFGINIKHILPMWDWVGGRFSIWSTVGLPLALAIGNDNFNKLKQGAYEMDVHFKSTDFKNNMPVIMALLGIWNRNALEYPTLAILPYAHSLRALPGYLQQTDMESNGKSVSKSGDKLSWLTAPVVFGQEGTNGQHAFMQLMHQSDDIIPTDFIVALKGRSQYTENHKVLVANCFAQSEALMQGKTLTQVESELEMSALSTAEISLIAPHKTMKGNTPSNTLVMDLLTPETIGSLLALYEHKIFVQGVLWQVNSFDQWGVELGKQLGTRILSAIDGAEDDLLSASSQSLIARFRARSNVTPSV

>sp_Q12VH1

MTKWLSISDVKEKIAATSAEEVTASYLELIDKSSINGYTCTSDGALDTAKMVDKGEVAGPLAGVPIAIKDNISTKGLATTCSSKILEGYVPPYDAHVIERLKEAGAVIIGKTNMDEFAMGTSTESSCYGVTLNPWDHERVPGGSSGGSAAVVAAGEAPISLGSDTGGSVRCPAAFCGVVGLKPTYGAVSRYGLISYANSLEQIGPMATCVEDIAAVMDVIGGYDARDSTSIDKKIDHQAALIDDVKGLKIGVPDEYFGEGVDSGTENAVWDAINKYEEMDASWEKVSMPNTKYALAAYYTIAMSEASSNLARFDGTRYGPRNDGENWHVMASKTRAENFGKEVQRRILLGTYALSAGYQDKYYLKALQVRTLVKQDFDRAFANFDVLMAPTMPLPAFKIGEMVEDPLSQYLIDVNTVPMNLAGVPCISVPCGSSDGLPVGLQIIGNHFDEAALIRAAYSFEKNTDHHKARPGEVA

>sp_Q12VF3

MKTYLELMRAGNCAMAAFAGLIGVLIAYNILSSASPYVSLSLFDTSLIFAIVFLVTGAGNGLNDYFDIEIDKVNKPSRPIPSGKISLKSALYFSLFLFITGITLAFLVNPLCGIIALFNSMVLILYAQSLKRTPFFGNASVGYLTGSTFLFGGAVFGMAGLQALVVLFLLATLATIAREIVKDVEDIVGDKKDGARTLPILIGAKKASYIAAAFGFTAMLASPVPYLQSILNEQYLFVVAIADIFFLIAVYQILGKKDAARSSKLFKFAMLFALISFIVGA

>sp_Q483Q8

MPTPHIEAQDGEFAETVLMPGDPLRAKFIADNFLDDAKCITQVRNMLGYTGTYKGKRVSVMGSGMGVPSISIYATELYKDYGVEKIIRIGSCGAVRDDIKIRDIVIGMAASTDSNVNRQRFHNVDFAACADFSLLKSVVDTAEKLGKPVHVGNIFTADLFYTPQPEKFATMEKYGILAVEMEAAGLYGVAAEYGKKALTVLTVSDHIKTGEKTTSEERETTFKDMMELTLESVL

>sp_Q12UJ7

MDKTIWIKADKGHWEAHKDRITTGLESGANCVLVNSDEVEKVRELGDIQVAAFTYDDKSGADIVVVGKGGEGDGTKPLSPDPVGSLDMITAIRLKEKGLTVGAYVVIQNKKYEEFAAEIGKECDFLIIVGTDWKVIPLENLIAALQDSDVKIIAGVRDQDEAKLALETMEHGSEGVLLDSDDPNTIKATVAVAERSGIEDLKLVPGKVTKVEAVGMGDRVCVDTCNMMTKGEGMLVGSQASGMFLVHSESEESPYVASRPFRVNAGAVHAYVKVGDRTRYLSELSSGDEVTIVNAGGKQRTGIVGRVKIERRPLMLVEAEVNGEIIKNILQNAETIKLVDINGEPISVADLKPGNEVMVYYEGGARHFGMKVEETIIEK

>sp_Q12YW2

MKDQYDLVVVGAGPAGSIAATTAAKKGLSVLMLEKRQEIGEPVRCAEGVGKKRLRQHIELDEKWLCGEVSSAKIISPNGTTLTMAEEDAGSEVGYVLDRKIFDRTLVELSGEAGVDIMVKARVTGLIIEENTVCGVEMMHLGKTYSIRSKLVIGADGVESKVGRWAGIDTSLKPSHIETCAQFLVSGVDIDQSSCYFYMGNKVAPGGYVWVFPKGNNMANVGIGILGSRAGEKKPIEYLTDFVEANYPNGSIIEQVAGAVPASGPIEKTIANGLMLVGDAARQSDPFTGGGISNAMDAGLYAGEVAAEAIAQDDVSEKILQKYEKRWRGSFGNEIANNLIVKETFFSLSDEDLDSLALSIKDVDFKKMDLIDFIAALFKANKKLLWNLRPLFTQKLKQKFSGLTKFKR

>sp_Q47XU3

MKNSQDTQVINLVADIGGTNIRLAITDKDNNINEIKTYQCKDFPHLSNVIYHYLKENGLLNSQVNACLAIACPVDTDSISMTNLPWKFSQKQLKEELKLHSLTLINDYTAIAMAIPLLSDKQKVKIGHGEAENKQPIAVCGPGTGLGVANLVNINNHWYCLGGEGGHTDFAPVDELDVKIFQQLKTTKKRLSYEQLLSGYGLEQIYQALVIINNQEATNAEQSKLSAKEISTQAIAGTCPICQQALSQFCKILGSFSGNLALTTGSFGGVYIAGGIVPRFIDYLKNSEFRARFETKGRMSHLNEQIPTYIITESQPGLLGAAAYLNQVFP

>sp_Q487A4

MKDELALTIANTIINGFERHFAIFTEITQSARNRFQQCQWNEIHRSARARTNFYDERVKETFNDIKEDFNISSLDDALWQRVKAVYSDLLINHKQPELAETFYNSVFCHLFERKYYHNDYIYVESTAHRLDDKTQPEIYTSYQPKELGLKQTICDIMNSHRTVIPFEDLDRDVDALINTFRRKAHKTRVKLEDLKFDILNFTFYRNKGAYLIGRVLSPAGETPFIIAVLNNEKGGLYIDALLTSSESMAVVFGFARAYFFVDCEHPYALVNFLQGLMPHKTKADLYSAIGFHKQGKTQFYRDFLNHLDSSDDQFELAAGIKGMVMSVFTLPSYPYVFKIIKDKFSPSKNITKKDVKGKYRLVKLHDRVGRMADTMEYSEVAFPKSRFNDELLAELQKVAPSIIRYEGEGEEALIIIEHLYIERRMVPLNLYLMDALKNKAQQKIDDALFGYGQAIKQLISADIFPGDMLLKNFGVTRHGRVIFYDYDEIAYMNEINFRVKPKAVTEEQLYAAEPWYSVMPGDMFPEELATFALANPSYLKAFKIHHEDLLTAAYWQQCQQDVANGIYKDVFPYPDKYRFCNLSFGSIKR

>sp_Q481G0

MNSILRHNWNLKEVEALFAMPFNDLMFKAQTIHRENFNPNEVQVSTLLSIKTGACPEDCKYCSQSARNKTDLEKESLLAVEKVLEAAQRAKEMGSTRFCMGAAWRNPKERDMPYVLDMVKSVKALGMETCMTLGMLSGDQADQLNGAGLDYYNHNLDTSPEHYNQIITTRTFQDRLDTLSNVRSAGMKVCSGGIVGLGEKAVDRSSLLIQLANLNPQPESVPINMLVKVEGTPLADIDDLESFDFIRCIAVARIMMPHSHVRLSAGRTAMNEQMQAMCFLAGANSIFYGCKLLTAENPETNQDIALFEKLGINTETVAGDTERSDNIVKTAIVDQQNSDLFYNASA

>sp_Q12X66

MSDILRRGRLASVPDEEIINFTSSMNADKWIFKADILVDLAHTIMLKERKIIKAEDCKKILEGLLTIKEEGIEKLDHTYEDIHISLESRLIDMVGEDTGGRMHSGRSRNDEVATCIRLTLRNDLLLLMEELIALRNTLNDTSSENLNTLMPGFTHLQHAQPTTLAHHLTAHANAIGRDLERTMDCYKRVNLSPLGAAAFASTGFDLDRERTCKLLGFDGLIENSMDAVSSRDFLIESASVFANLMINLSKVAEEIVIWSTSEFAFIELDDRYASTSSIMPQKKNPDTAELLRGKSGVTIGSLMSLLAICKALPLSYNRDLQEATPNIMQSLETTRASVRIMNGMIATMSINKENMAGLATAGFTTATELADTMVRVCDIPFRTAHQIVGVLARGSGEPTLGEIDAVAHNVIGESLSSRGLTEKMVKEALDPILNVSKRSVIGGPSPESMERLIESSRERIANNTEILESLIANRDNAIESLFCEVEKCIDV

>sp_Q12VF1

MLKIGVFGCGAIGTELCKAIDSGHIEVELYAVYDRHEQSIINLKEQLKNTDPKVLEIVEMVKHVDLVVECASQQAVYDVVPTTLHAKCDVMVISVGAFADKKLLDTTFDIAKEYGCKIYFPSGAIVGLDGLKSASAASIYSVTLTTQKHPRSFEGAPYIVQNNIDLDSIKGKTVLFEGMASEAVKAFPSNVNVAASLSIAGIGFDKTKVKIIANPALTRNIHEITVEGEFGMFTTRVENVPAPSNPKTSYLAALSAISTLKKIADPLQVGT

>sp_Q480Q1

MTNAISNFTIYAFVALGGACGASLRFYISQLVLNWLGKGFPFATLMVNIIGSFTMGLLYQLIEHEILNVSVHRTLIGIGFLGAFTTFSTFSLDSLLLLQQGDVLKAAINILLNVSLCIAAAALGMFMVTTLTK

>sp_Q12WF6

MPEFVTFSRNVFIPVTNICRNLCGYCTFRRDAGHPEAHLMSMSEIRPILERGEKAGCTEALFVFGEYAEEVPEYLLELEKLGYSTTVEYVADLCKLAIEIGLLPHTNAGILNRKELEILKPLNISMGLMLETTAELKAHSESPGKKPSTRIEMIRTAGKLKIPFTTGILVGIGETKDDRKRSLNTIADIHKEFGHIQEVIIQNFMPKPDTPMADHAPPSKEEMIDTVAIAREILPSDVAVQVAPNLIDPYSLIKAGASDLGGISPTTIDWINPEAEWPDVIELQKMIKEIELRERLPIYPQHIKKGWYSNNLSYLIETLTDKNGFKRKQR

>sp_Q12W15

MKKYIVKCPGCGEVRDQYALHCPDDDALPRTEYFKKQIVPADMPGMWRYYDWLPVNGIIEKGSGRPVTYKSEGFAKELRLSDLNITFNGYWPENEGFIRTCSFKDLESFPTMQRLLENNERRVLVVASAGNTARAFAHVASITGYPLLLIVPKNSTHRLWTTEEDTSSVCTVTVDGDYYQAIAMAEKIAARDGFVSEGGARNVARRDGMGTVMLDAVLTTKSLPQHYFQAVGSGTGGISAWEAAMRLIEDGRFGNNMPRLHLAQNLPCAPLYSTWTGEQTNGNCPEEMYDDVLFNRKPPYLATGGVKDALDDTNGIIYGITNKEADEARKIFEENEGIDILPAPAIACAAIMKALEKGEIKADENIVLNITGGGQKRLEEELPTRQLSVDLALSPDDKDAENKILEKVAELLKNGGY

>sp_Q12ZB3

MLITSSRKPSANTRTMCKYLASFFNCKYMTRGKMGLIDIVSLCENGLLMVVGDYHGSPGSIMFYDSHGVELLSIHLSVFYPDGYKYTPLKALEPSINGDGELFNLLSYYLDIPEGECYYDSKCLIASDDHLEFVYLDNMLFRLNIKNYRKMVMSE

>sp_Q484U8

MNILRLDASMRKTGSYSRILTDKLIEQLTSGKNNEVTIRDLADGIPLIDENWIKANFTDVDERTCEQKVCLVASDILVDELYKAEHIVIGLPIYNFGVPAAFKAWIDQVVRSKLTFRYGDNGPVGLVENKKAYIIIASGGTKLGTEIDFISDYLRHILGFIGITDVTFIDSSGLGRDESQTLAHAHKAIERV

>sp_Q485P0

MKNKNRLNTIKLLSISLLIAVTTACSNTVELSKALPNDPDFAPIMPEEEEERIVPSGSLFKPHYVNNIYSDSKAHRVGDIISVILSEKTQAKKNAKTELKKANETNLDAVTGLGGVPVSINGESLQFGISQDSNFKGDAKADQGNSLSGNISVHVLRVLPNGNLMIRGEKWLTLNNGDEYIRLTGVIRSKDINSNNTILSNKVANARIQYAGTGSFADSNEQGWLVKFFNSTWWPF

>sp_Q48AD4

MPELPEVEVCRLGISPHVIAQEVSEVIIRNKRLRWPIPDEVCSAVGLPVLKVERRAKYLLLRFSTGTLLLHLGMSGTIRVIEQDTPVAKHDHFDLVFKHGKSLRLNDPRRFGAVLWLANDEDELGLLAKLGPEPLSDDFAEGYLFSKAKNRKVPIKTFLMNNHVVVGVGNIYANEALFQAGILPTAKAKDIDEHRMNSLTAIIKKVLSAAIAQGGTTLKDFTQADGRPGYFAQSLMVYGRAGEACVTCKTKLQEIRQSNRSSVFCPSCQQD

>sp_Q487R8

MTAKFTVLVLNGPNLNMLGKREPTIYGNQGLSEIIADLGLQADQKNIVLKHLQSNAEHELVDAIHNGYQQVDFIIINPAAFTHTSVAIRDALLSVAIPFIEVHLSNVHAREAFRKHSYLSDIATGVICGFGAQGYSFALDAAYTYLNKAQVDK

>sp_Q12TL2

MTANNANKKHLLVLGTASHVGKSAIVTALCRIFSADHKVAPFKAQNMSLNSWITVDGKEIGIAQAIQAKAAGVEPTADMNPVLLKPKGDRVSQVILLGEPYADKSAGAYYDSIEETHDVLKGALKRLEAEYDLIVMEGAGGAAEINLYDRDIVNIGTARITDAPIILVGDIERGGVFASLYGTIQLLPEDVRKNVRGLIINKFRGDPAILESGLTELEELTGIPVLGVMPYFKLRIPSEDSVSIGDKSADDGEHYDVDIAVIRLTRISNFTDFEPLEHMAKVRYVDLSDDLGNPDAIIIPGTKNTTSDLNDLVESGMADKIKSFYGMVPILGICGGYQMLGKSIVDSGIEGGESARLDGLGLLDIETVFDAYEKRTVQVTKTVKESGPIFDSIKGEDVKGYEIHMGISSSKRPVFGDDGCADDSGLVIGTYLHGLFDNVNIRRALISYLLEKKGLEFKEEEIPDKDPYDELADVARENLDMNKIYGMIGLEPEKV

>sp_Q48AS7

MDHSPWQRCLSVLQEELPAQQFSMWIRPLQCVINDNVMTLYAPNRFVLDWVRDKYVNRINELLTINESNNPLLLRFDVGSKPTIDNSVTNSPVSRNTGGNESLFAKATSAPKVAEPESNIPKKTNVRLNYTFENFVEGKSNQLARAAASQVADNPGTAYNPLFIYGGTGLGKTHLLHAVGNGILLNKPNAKIAYMHSERFVQDMVRALQNNAMEKFKQYYRSVDALLIDDIQFFAGKERTQEEFFHTFNALLEGNQQVILTSDRYPKEINGVDDRLKSRFGWGLTLAIEPPELETRVAILKRKAQESQINLADEVAFFIAKRLRSNVRELEGALNRVIANANFTGRAITIDFVREALRDLLALQDKLVTIDNIQRTVAEYYKIKIADLLSKRRNRSVARPRQIAMALSKELTNHSLPEIGDAFGGRDHTTVLHACRKVKSLREETHDIKEDYSNLIRTLSS

>sp_Q12WF5

MIPDEIKERAYQGTTTKEDALTLLEIEPFELFELADQIRAKAVGDNVTYIVNRNINFTDICIGTCGFCAFKDKKGYLLSIDQIKDKIKEAHVSGATEVCIQGGLLPNVKIDLYIDILKAVKSDYPHIHTHCFSPMEVNHAAKASGLSVEETLKTLKANGLNTMPGTAAEILVDKVRNIICPDKLTRQEWIDTVTLAHKLGIQTTATMMYGHVDTWEDRIEHILTIRRIQKDTGGFSEFVPLSFMPYNNPIGEKMMEEGRFMNTGIDDLKIYAIARILLNTHINNIQTSWVKLGKKLAQMALYCGANDMGGTLMEESISSSAGASSGEAISAEELEWIIRATDRKPVQRDTLYRSIR

>sp_Q48AW7

MNDSQYNLIAEALLLAIEEAIEDSGVDIDYEGVGGLLTLTFKNNSKVIINKQAPLHEIWVATKFNGHHFVLNNDSWTDKRSGEEFWQFLSNAVSTQAETELTLSAQ

>sp_Q482Z9

MSHQSDLIEEDIQAYLKQHENKELVRFLTCGSVDDGKSTLIGRLLHDSKMIFEDQLAAIEKDSKKSGTTGEAIDLALLVDGLQSEREQGITIDVAYRYFSTDKRKFIIADTPGHEQYTRNMATGASTCDIAIILIDARYGVQTQTRRHSFICSLLGIKHIVVAVNKMDLVDYSQERYQEIKKEYREFTESLEFSDVRFVPLSALNGDNVVDESVNMPWYPGATLMKLLNTIDVKTQEQFTQLRFQVQYVNRPNLDFRGFAGTLASGHVLVGDTIVALPSGKESVVKEIVTYDGNLERADKGMAVTLTLEDEIDISRGEIIVKKGSLPISAKEFSATVVWMHENELEPGREYFIKHGSKMTTGHAQNIVSKYDVNTMESLSSSQLAINDIGIVNFVAGETLHFDAYEDNQGTGAFIIIDRLSNVTVGAGMINHAIDEKAQEYSAFELELNALVRKQFPHWGARDITK

>sp_Q12XL6

MVDVIKNTQIHCTYGCKSSKAVQPDVAGKMISKAKRPLFIVGSQILKDEELLKRSIEIAKKADLPVAATGHSMSGLVDQGVNAKYINVHALATYLCDPNWTGLDGKGQYDTIIVLGHFKYYIDQVLSGLKSFSKLKSIAIEREYIQNATMSFGNITPAVHIEALDELIDNL

>sp_Q12X56

MSEKIGILAIGHGSRLPYNKEVVSEIAATIAKKHPDYVIKAGFMENTLPTVMEALADFDGTGVTKIIAVPVFLASGVHITEDIPEILKLDPETNEGKITVDGNEIPVTFGKPLGHHELLADLVFERAMEVM

>sp_Q483D0

MKQFNEFYQKIATNRLGHWLNTLPAQLSHWHESELHGEFKHWQKTLDALPVVDANSAIDITNTVKVGESGDLDQGQFKRLENLMKKFKPWRKGPYHIHGLHIDTEWRSDFKWDRLAEHISDLSGKYVLDIGCGSGYHLWRMRGAGAKFVVGIDPTQLFLMQFNAIKHFIDDSPVHLLPLGVEQLPELKAFDTVFAMGVLYHRRSPIDFLYQLKAQLVKGGELVLETLIVDGDENTVLVPGERYAKMRNVWFLPSEKAMCAWLERCGFNNVRVVNTDITALDEQRKTEWIDTESLQDFLDPNDSSKTIEGYPAPKRAIFIANA

>sp_Q47XL8

MFNSQITSQRSSQVSSQSSATGIESALVPMVVEQTAKGERSYDIYSRLLKERVIFLCGQVEDHMANLIIAQLLFLESESPDKDIYLYINSPGGSVTAGMAIYDTMKFIKPNISTVCIGQAASMGAFLLSGGEKGKRYCLPNARVMIHQPLGGFQGQASDFEIHAKEILFIKDKLNKLMAEHTGQTLDKVSQDTDRDNFLSAEAAVEYGLVDSILEQRNDK

>sp_Q12UY1

MKVFLSGSIRGGRQMLPTYQFICRFLRNKGHEVLSWHVADSEVEGKESLLTETQIYERDMSFLQDSECMIAEVSMPSIGVGYEVCSAIKKGIPVMCVHMPDSNVSAMLLGNTYADISVRLWG

>sp_Q487H4

MLILTRRVGETLMIGDEVTVTVLGVKGNQVRIGVNAPKEISVHREEIYMRIQAEKGDNEASGNKV

>sp_Q47WL9

MSRTVFCQNLNKEAEGLGFQLYPGEIGKRIFDNISKEAWTIWQKKQTMLINEKKMNMMNVDDRAFLEAAMVAYLFEGKEPEIEGYVPPSK

>sp_Q48AS9

MVTPLNIIFAGTPEFAAQHLAALINSEHNIVAVYCPPDKPAGRGKKLTACATKLLAIEHDIIVEQPINFKNEEDQQQLAKYNADIMVVVAYGLLLPEVILNSPRLGCINVHGSILPKWRGAAPIQRSLEAGDKKTGVTIMQMDKGLDTGDMILSAECEIENTDTSASLYEKLANLGPTALVNTLTIMAEPDYQASNHNIAQDDELATYAKKLDKTEAELNWQFSADELHRKIRAYIPWPVAQFTFTESEGKQHRLRIWQASVQEYRGNADPGTIIKADKEGIEVATTSGSLRLEVIQLPGKKALAVKDILNGRSDWFVVGSTINKLG

>sp_Q47W03

MSNMTPREIVHELDSHIVGQSDAKRAVAIALRNRWRRMQLDKDLRNEVTPKNILMIGPTGVGKTEIARRLAKLAHAPFIKVEATKFTEVGYVGKEVETIIRDLADMAIKMVKESEMDRVKHLAEEAAEERILDVLLPPARDGFGNDEKSDDSNTRQIFRKKLREGKLDDKEIELDLAAPQVGVEIMAPPGMEDMTSQLQNMFQNMSSEKTNKRKLKIKDALKALQEEEAAKIVNQDDIKQKAIDAVEQNGIVFIDEIDKICKRADSSGGGDVSREGVQRDLLPLVEGSTVSTKHGMIKTDHILFIASGAFQMTKPSDLIPELQGRLPIRVELQALTADDFVRILTEPFASLTEQYIALLATEGVSVTFTDDGIKAIADSAWQVNETTENIGARRLHTMMERLVEDLSFNADQRSGETISIDQAYVTKILSEVVKDEDLSRFIL

>sp_Q47XB7

MIDKLAREELVDMVPYQSARRLFASGDNEQANSRTWLNANEAPGQGQYQLSSENINRYPDFQPQALLKAYSNYCNLPVDNILATRGADEGIELIIRSFCRAYQDSVLICPPTYGMYAISAENHGAGIISVPLVNTPEAQCQLDLEGLKQQVGKAKVVFLCSPGNPTGNTLSSAQIKAAIEIFKDSAMVVVDEAYYEYTNKELGAEQVNIKLISQYDNVIILRTLSKAFALAGLRCGFTLSNKAVITLLSKVIAPYPIAAPVAEIASKVLTNDLDVMQARVISANSLREQLSEWLKQQKWCSDVFDSNANFVLFRCNNIDEKNKVFNLLVEHNILIRDQSKQQQLENCLRISIGSEDEIAQLKQLLETL

>sp_Q484R7

MHSNSTIEKSVNSLADSLVIRQLNTMDYSQVWHAMKDFTDNRDDTTADELWLVEHPAVFTQGQAGKAEHLLVPGDIEVVKVDRGGQVTYHGPGQLVVYVMINLRRKKIGVRQLVTLIENSIVSALTDYDIAAYAKADAPGVYVDEKKIASLGLRVRKGCSFHGLAMNVNMDLSPFLRINPCGYAGLEMVQTADLQGPKDTASASTALVKHLINLLKANNVSHQVGLPNENNKYHE

>sp_Q487R0

MSIRPLHDRVIVKRKKVESKSAGGIVLTGSAAEKSTRGEVIAVGNGRILENGEVRPLDVKVGDQVIFSEGYGVKTEKIDGEEVLILSESDILAIVE

>sp_Q489P3

MALAKKTIKKVVLAYSGGLDTSAIIPWLKENYDGCEVIAFCADVGQGDEELEGVKEKAIASGASECYVVDLKEEYVKEYIYPILKTGSVYEGQYLLGTSMARPIIAKAHIEVALKVGADAVCHGCTGKGNDQVRFESCFAALAPELTVIAPWREWDMVSREDLLDYLAERNIPCAASLTKIYSRDANAWHISHEGGELEDPWCEPSKEVWTMTVDPMDAPDVPEKVQLSFKEGELVGIDGKDFSQHGAGAYEALMYLNEKGSAHGVGRIDIVENRLVGMKSRGCYETPGGTILMAAYKGLETLILDKESLKYRESVGLEFSHVIYDGRWFTPLAKAQLASAASFAEKLTGDVVVKLYKGMAQVIQRRSPNSLYSEAFATFGADDVYDQKHAEGFIRLFSLSSRITALSQKDSLKDKAE

>sp_Q47ZC3

MSGNTFGKLFTVTSFGESHGLGLGAIIDGCPPGLELTEADLQIDLDRRRPGTSRYTTARREADEVKILSGVFEGKTTGTPIGLMIENTDQRSKDYGNIADSFRPGHADYTYWQKYGLRDYRGGGRSSARETAMRVAAGAIAKKYLAEKFGMTIQACVTQIGDIVASGPKGAPFDVNTVDWSSVEDNPFFFPDETKIEQLGEYLRDIIKEKDSIGAKVTVVATNVPVGLGEPIFDRLDADIAHGLMSINAVKGVEVGDGFAVVNQKGSEHRDELTPEGFSTNHSGGVLGGISSGQQIIAHLALKPTSSIGVSGKTVNLTGEATDIITKGRHDPCVGIRAVPIAEAMLALTLMDHFLRHRGQNADVQCNTPDIEA

>sp_Q487I6

MNPRRKKRLAIVGSILIGIGVVSGLVLYALSQNIDLFFTPSEITQGKKETGLKPSLGQRIRIGGLVVPGSVKRDPENLKVSFRLSDMAMPIVFKDSDPMVTVYYEGILPDLFREGQGIVANGTLTEHPPTGLSIEASEVLAKHDENYMPAELAEAAGQKHDKATYSDKQLESKKTNSY

>sp_Q47XL9

MTDIKSGGDNGKLLYCSFCGKSQHEVRKLIAGPSVFVCDECVELCNDIIREEISEISPKESKEALPSPIEIRESLDEYVIGQDHAKKVLAVAVYNHYKRLRNGDNHNGIELGKSNILLIGPTGSGKTLLAQTLARLLDVPFTMADATTLTEAGYVGEDVENIIQKLLQKCDYDVEKAQRGIVYIDEIDKISRKSDNPSITRDVSGEGVQQALLKLIEGTVASVPPQGGRKHPQQEFLQVDTSKILFICGGAFAGLDKVVEQRNHTGTGIGFGAEVRGKDQEISLTDRLADVEPQDLVKYGLIPEFIGRLPVLATLRELDEAALIQILQEPKNALTKQFTALFDMENVELEFRSDALHAIARKAMDRKTGARGLRSIVEAVLLDTMYELPSMENVSKIVVDENTIKGESKPIVIYDSKQEQAASE

>sp_Q489A4

MIALIQRVSQASVTVNGEIIGEIEKGLLVFLAIEPLDNEQKAKRLAERVAGYRVFNDENDKMNLNVKQAEGNILVVSQFTLAADTSSGMRPSFTTAAKPEFSNHLYQFFVTQLRDKGFDVPTGEFAADMKVALINDGPVTFTLTI

>sp_Q47XD5

MTIRIAINGFGRIGRSVVRALYESGKTDLFTLVSINELAPASGIAHLLKYDSTHGRFPFSVSEKENQLIINGDEIALTHIGNLNSLPWQQQNIDIVLDCTGKYGNKADGLSHINRGAKKVLFSHPGSQDIDATIIYGINHQTLTSSDRVVSNGSCTTNCIVPVIKVIDEAFGVESGSITTIHSSMHDQQVIDAYHKDLRLSRAASQSIIPVDTKLAAGIERILPKFKGRFEAIAVRVPTINVTAMDLSLTVNNDVCICDINQAIQAATSNHDLYGVLSYTEEPLVSVDFNHDPHSCIVDGNQTRVSHKRLIKMLVWCDNEWGFANRMLDTAMAMHQAEN

>sp_Q12XB4

MLDFVGLGLFDEKDISLKGLEKIHNADKVYVEFYTSILMGTDLEKMEMLYKKKITVLSREDVEQHAEDWLVDAKDSNVVFLTGGDTMVSTTHVDLRLRAADMGIKTTLIHGASIASAICGLSGLQNYRFGKSVTIPHPYVSNRGVRVVSQTPYDTIKNNIEAGLHTAVFLDIDKDKGYMTVNQAMEILLEVEGKLGEGVMVDRLAVGIARAGSPSPVVKADYIEALRDYYLGGPLHIVVIPAELHFVEAEALVKLAGAPEGILENID

>sp_Q487D3

MITNLADYPLLSQINIPEDLRNMPQEQLTRISNELRSFLLNSVSKSSGHFASGLGTIELTVALHYVYNTPFDHLIWDVGHQAYPHKILTGRRDQLHTIRQKGGLHPFPWREESEYDTLSVGHSSTSISAALGLAVAAEKEAKNRKTVAVIGDGAMTAGMAFEALNHAGDIKKDMLIILNDNDMSISKNVGALNNHLAKLLSGSIFTGFRESSKKLLGNIPPIKELASRAEEHLKGMVVPSTFFEELGFNYIGPIDGHDVESLVTTIKNMRNLKGPQFLHVVTTKGKGYQAAEQDPIKYHAVPKFNPEETNLPQSKPSLPTYSKIFGDWLCKTAEIDKKLVAVTPAMAEGSGMVEFSQRFPDQYYDVAIAEQHSVTYAAGLAIGGLKPVVAIYSSFLQRGYDQFIHDVAIQNLPVMFAIDRAGIVGADGATHQGVFDLSFLRCIPNTVIMAPSNERECQLMLNTGYKLDGPSVVRYPRGNGTGEILPSVDETIELGKGVTILTATVIESQEQTNKSIAILSFGSMLGEAKKAALELNATLVDMRFVKPLDETLIDTLNAKHDCLVTVEDNAIAGGAGSGVNEYLLAQGKPVTILNIGVTDHFVKHGTQEEMHHELELDAEGIVIKIKRFIN

>sp_Q47WR1

MSKISKILAREIMDSRGNPTVEADVYLESGAFGRAAAPSGASTGSREALELRDGDKARYLGKGVLKAVAAINVNIQAALIGQSALDQANIDQIMIDLDGTENKEQFGANAILAVSLANAKAAANEKKVQLFEHIADLNGTPGVYSLPLPMMNIINGGEHADNNVDIQEFMVQPVGAKSFREALRMGAEIFHALKKVLSSKGMSTSVGDEGGFAPNLESNADALAVIKVAVEAAGYELGKDVTLAMDCAASEFYDADKGIYDLTGEGKQFTANEFSDFLGELCKEYPIVSIEDGLDESDWDGFKYQTDLLGDKVQIVGDDLFVTNTKILARGIENGIGNSILIKFNQIGTLTETLAAIKMAKDAGFTAVISHRSGETEDATIADLAVGTAAGQIKTGSLCRSDRVSKYNQLLRIEEFLGDKAIFNGLSEVKGQ

>sp_Q48A85

MKLQEKEVLLFDLDGTLVDSAPDLALAVNRTLKDLNKATFDQDTIHHWVGNGAKVLIERALSGSAIIDKELDETLTKDALTIFLAHYQQCLCIESVLYDDVQEGLLSLKAAGFRLAIITNKPAIFIQPILTGLGIDNLFELLIGGDTLADKKPHPAPLHYAMKQLNVVAEQCVMIGDSKNDILAAKAANIDSVGLTYGYNYGEDINQYGPQWCFDTFNELLISLKR

>sp_Q12WD9

MGEVAATMKIMPEGVDTDLDDLKIRLEAVLPEGASIFGSEIEPVAFGLKALKLVVLVGDLEGGTEPVEEAFAAVPGVESVQVTELGRPV

>sp_Q485F9

METVKKPIDLNEIKTLIPHRYPMLLVDKVIDHEPGKTLHAIKNVTINEPVFTGHFPELAIFPGVLILEALAQATGILGFKSTEGRGDNEMYLFASIDKAKFKKPVLPGDTMHLHVEFLKERRGMWKFYGEARVDGKVVCSADLMCARRPL

>sp_Q47VB1

MQRLAPALRQDNVPLDLISLIKTILAATKEISFRVSQGHLGDVMGSTLDENIQGEVQKQLDVVANELFKDILLESGFVKAISSEEEDHSVAGDENGKYIVSFDPLDGSSNIDINSLIGTIFSIHEAPKDIAAGDDDMFKQAGDKQVCAGYVLYGPSTMLVMTTGSGTHFYVLDRTHGGFLLVERNVQVPADTQEFAVNMSNQRFWQAPMQNYISDLLAGDTGPREKNFNMRWIAAMVGDIHRVLCRGGIFTYPADSRKPEQPYKLRLMYEANPMAFLLEQAGGLAMTSEGRIMDIEPNSIHQRVEVIMGSKNEVEKCLSYYN

>sp_Q489W4

MNEVVIVDCIRTPMGRSKAGVFRNVRAEALSAHLMKQILVRNPALNPEDIEDVIWGCVKQTKEQGFNIARNASLLAGLPKSIGGVTVNRLCGSSMEALHQASTSIMSGQGDVFLIGGVEHMGHVPMMYDVDFDPALNKNIALASGNMGLTAELLGKQHGITREMQDAFGARSHQKAHEAHLAGRWDNEIVATQGHDATGALTLVEHDEVIRPETTAESLSALRPVFDPVNGTVTAGTSSALSDGASAMLVMSAAKAKELGLTPRVKIRGMAVAGCDPATMGFGPVPATKKALKRAGLSIADIELFEFNEAFAAQALSCVRSLKVEDKMDQINLNGGAIALGHPLGCSGSRISGTLINLMEGQDVNIGLATMCIGLGQGIATVFERV

>sp_Q47YI0

MSNVEKKISQLQQQLNQYNHEYYVLDQPSVPDAEYDRLMTALIDLEKTNPELKTIDSPSQKVGGQALKSFTQVTHQLPMLSLDNVFSLDDFHAFVKRVKDRLNDNQAIVFCAEPKLDGLAVSLRYEHGQLIQAATRGDGSVGENITTNIRTIKSIPLKLMGTPGKDFPDIVEVRGEVFMPKASFDALNTLAKKRGEKGFANPRNAAAGSLRQLDSKITAKRNLAFYAYSLGFVGKLSDGGAESTDLTNDFFANSHHERLCQLKRLGLPMCPEVRLLESEQACDAFYQDILAKRSALSYEIDGTVLKVDEISLQKRLGFVARAPRWAIAYKFPAEEELTCVEDVEFQVGRTGAITPVARLKPVFVGGVTVSNATLHNQDEITRLGLKVNDFVVIRRAGDVIPQIVSVVLDKRPDNAVDIVFPTSCPVCDSAVAKPEGEAVLRCTAGLFCAAQRKEAIKHFASRKAHDVDGLGDKLVEQLVDEKLINTPADLFKLTEIQVSTIDRMGKKSATNLINGLEQAKSTTLAKFIYGLGIREVGEATAANLANHFYTLAAIESASLEDLQNVSDVGEVVAKNIINFFKEEHNLAIVSGLSEVMHWPTIEIKSAEELPLAEQIFVLTGTLTQMGRTEAKTALQSLGAKVSGSVSKNTHFVVAGDKAGSKLTKAQDLGISVLTEDGLVALLAEHGITI

>sp_Q12V67

MQNTDTTKYIIHSKISADGIIERPDIVGAIFGQTEGLLGSDLDLRDLQKTGRIGRIEVAVGAKGGKTKGNIFIPSSLDRVETSILAASLETIDRVGPCAAKIEITQVEDVRATKRKHIIERAKFILTDMFDENLPESQEIADEVRQSVRIEEMQYYGKNKIPCGPNVVESDAIVVVEGRADVLNLLRYGIKNTICVGGTNVPPEVAELTKKKTVTAFTDGDRGGKLIIKELLQVADIDYIARAPDGKSVEDLVQREIVRSLRQKIPVEQALENYTIKTDAKPVTTETPAKGSRITRLPKRKERPELSVPAVKQLPNKPSRSEEKPGVTREPRTTREPRTTREARTTRETRSPRETRSPRETRSPRETREPKEAKPAMLSPQALRFKPHSDALIGTLGARLLDSKDEIIEETAVRDLVNTLKENDEGIKSVVFDGVVTQRILDIASDKGIENLIGVKKGNIAKSPASVNVLTASDF

>sp_Q12VH4

MQVEEYLNDIAEREGTVHLTLIDPASQSPEAAAEIAKAAVAGGTDAILIGGSTGAVGVALDQTLIKIKEQVDVPTILFPGNAGGVSTHADAIFFMSLLNSRDINYIITNQVMGAPVVYKSGIEPISMAYIISEPGGTVGWVGDAKLIPRNKPEIAAAYSLAGKYMGMHYTYLEAGSGADRPITPETIGAVKHVLGDNKLIVGGGIRDGKTAKICADAGADMIVTGTIVEETDDVRKKIEEIVSAIKK

>sp_Q487R3

MKNLLSLCFLMLAAFTLNPAAAQEKQSIFDVSNSSLFSNDDEFLKVDQAFAFNFYQKNNLLEVSFDIAPEYYLYRHQFKFKGKNTQFTSVLLPDGIDHEDEFFGVQKIFTENLAFTVNLENVSNDASIKITYQGCAEKGLCYPPTSKVIKLSKFILGESTSAPSSDAAQQTNEGEVKKSEQHQLSDMLKQDSLLLTLIAFFVGGLLLSFTPCVFPMYPILTGIIVGQGEGLTTKKAFTLSFFYVQGMAITYTLLGVVVAMAGAKFQAVFQHPIVLIGLSILFIFLALSMFGVFNLALPASWQNKLNNVSNKQKGGSITGVLMMGVISGLVASPCTTAPLTGALLYISQTGDVVLGASALYALSLGMGLPLLILGSSGGKLLPKAGAWMNIIKNIFGLLLLAVPVFLLERFIPEVASQALWALLILVSASYFYVANQNHAAAQNVQQGKGFWYGLRSLVIFLMLFFGANLAYQLIYPSSNNVTNNAQHASFKQVTSLAQLEDEVKKANMQGKTVMVDLYADWCIACKEFEKYTFVDADVQKALSNSVWLQIDMTEFDSTDNAELVQHYTILGLPSILFFDLQGNELTKQRTTGFMKAAEFSAHVKSIFK

>sp_Q12X65

MDFELGDRIRIEKDGNTYEGIVMPSNTDHIVVKMVSGYNAGVDPEGASITLLEKAGPKNAPKAKPDKAKGQKKLPKVTILSTGGTIASKVDYRTGAVTAQFSADDIVDAIPELTEIADINGRVLYNILSENMKAEYWTELAGAVAQEIENGADGIIIAHGTDTMMYSAAALSFMLKTPVPVVFVGSQRSADRPSSDNAMNAICATKVAVSDIAEVCVVMHDTTSDDRCAIHFGTKVRKMHTSRRDAFQSINSDPIGYIDHSTHKIETVLPFSKRDSQKLELKNTLEPKCSLVKFVPGANPDVLSYYIDSGYKGLVIEGTGLGHVSTDWIPNIKRATENGIPVIMTSQCISGRVCDRVYDTGRDILKAGAIEGEDMLPEVALVKLMWALGQSNDVDEIKEIMRSNIGHEMTDSTLK

>sp_Q487P4

MANFSTNQFKAGLKIMLDGEPCNILENELVKPGKGQAFSRVKIRKLVSGKVLEKTFKSGETVEGADVMEVELAYLYADGEFWHFMNNETFEQIGAEEKALGETVKWLVEGDICTITLWNGTPITVTAANFVEIDITETDPGLKGDTAGTGGKPATLATGAVVRVPLFVQIGEKVRIDTRSGEYVSRATKAQ

>sp_Q487Z1

MADLSKYRNIGIFAHVDAGKTTTTERILKLTGQIHKTGEVHDGESTTDFMEQEAERGITIQSAAVSCFWNDHRFNVIDTPGHVDFTVEVYRSLKVLDGGVGVFCGSGGVEPQSETNWRYANDSKVARIIMVNKLDRLGADFYRVCKQVKDVLGANPLIMTLPIGTEDEFVGVVDLLSEKAYIWDDTGLPENYEVTDIPADMVEQAAEYRVKLIETALEVDEDMLMEFLEGEMVPTIEQIKACIRTGTRDMTFFPTYGASAFKNKGIQLILDAVVDYLPSPTDVNPQPLTDEEGTPNGEFAIVSADETFKALAFKITDDRFGTLTFVRIYSGTLKKGDTILNAATGKTERVGRMCEMQADDRNELTSAQAGDIIAIVGMKSNVQTGHTLCDPKHPIVLEAMVFPKPVISISVTPKDKGSTEKMGLAIGKMVAEDPTFKVETDIDSGETILSGMGELHLDIKVDILKRTYGVELEVGKPQVAYRETITTAIEDSYTHKKQSGGSGQFGKIDYRIKPGEPGSGFVFSSVVVGGNVPKEFFPAIEKGFKGMMDTGVLAGFPVLDVEIELYDGGFHAVDSSAVAFELAARGAFRQSIPKAGAQLIEPIMKVDVFTPDDHVGDVIGDLNRRRGMIGGQEAGVSGVRIKADVPLSEMFGYIGTLRTMTSGRGQFSMEFSHYMPCPNNVAEIVIAEVKAAKIAKDAARK

>sp_Q12TW7

MRSDNTKKGDARAPNRSLLKAIGVTDSEMKKPFIAVVNSWTEFIPGHIHLDKVAEAVKAGIRNAGGVPFEFHTIGVCDGIAMGHEGMKYSLPSREAIEDTIEIMIQGQQMDGMVMVTSCDKITPGHLMAAGRVDIPAIVVTGGPMLPGFVDDKYTDLVSVFEGVGSCQSGAVSSEKLKQLEDLCCCGAGSCAGMFTANTMACMTEALGLSLPGCATAHAVDAKKMRMAKESGERIVEMVSEGLTARKIVTDKSFENAIRVDLAVGGSTNTTLHLPAIAHEFGLELPLEKFNELSKTTPHLIGLRPGGENFMIDFERAGGVQAIMKRLVTKLNLDEKTITGKTVGENIDEFVIVNPKTNARVITTIEEPLHEEGGIAVLKGNLAPDGSVVKQSAVHEKMLRHTGPARVFDSEEEAMETILKGDIKSGDVVVIRYEGPKGGPGMREMLSPTSAIAGMGLIDSVALITDGRFSGGTRGPCIGHISPEAYEGGPIGLIQEGDIIEIDMPERRLELKVSEEDLEKRRVLFKPVEKEATGYLSRYRKIVSSASKGAIRE

>sp_Q47XI4

MTTESTSNKEELSAEQLADDIVQQAEEQVEDQHDHAHEVISAEQEKINELELALATAQSTVADQKDSVIRAKAEVDNIRRRAAQDVEKARKFALEKFAGEMLTSVDNLERALQNIDKEDESNKGVIEGVELTLQGLITSLDKFGVKAVDPQDQPFNPELHQAMSMQEVPGVAPNTVIAVMQKGYELNGRLIRPAMVMVSKAAPTVDATA

>sp_Q12WC6

MSFEVIPAVDMKGGKCVQLVQGVPGSEMISLDDPVEVALDWVSQGARTLHLIDLDGAIEGNRTNAPIIKKIVEKCKPQGIYIQVGGGIRSFEDAATLLDIGIDKVILSTAALKDPELIKKLSDEFGSEHINVALDSKNGKISIEGWTKESEHTAVEMGSQFEEKGAGSILFTNIDSEGLLNGVNPKPTEELVNAVTIPVIASGGVTTLEDIVTLKNTGAAGVVVGSALYKKRFTLTEAINIISDKN

>sp_Q47Y92

MSIVAVGINHKTAPVAVREKISFNPDKLSIALQEMLNAVQCREVAILSTCNRTELYLVQDGDFDVTQQRLIKWLESFHNVPASTILPSLYWHKDQQAVNHMMRVACGLDSLVLGEPQILGQMKQAYSQAKAAGSMSLIMDRLFQRTFGVAKQVRTETEIGASAVSVAFASVNLAKHIFGGLEKTKVLLVGAGETIELVAKHLYENNVGKITVANRTLARAENMATKIGADVITLAQIPEHMCNADIVISSTGSTLPIIGKGMVEQALASRKHQPIFMVDLAVPRDIEEQVSELEDVFLYTVDDLQGIIAKNIANRRKAAVQAESIVNSQSDNFMAWLRGLNTQDTVISYRKQCLDNRDVLLEKAFIQLKNGKNSEAVLAELANKLTNKFMHAPTSALQSAAQGGELDKLIYLRDIFNIDSQE

>sp_Q482J9

MSNIEERVKKITVEQLGVSEAEVKIDSSFVDDLGADSLDTVELVMALEEEFDTEIPDEEAEKITTVQAAIDYVTANQ

>sp_Q486G0

MPVNLPEIVPASLTPIQGIRLGWAESNIKTQNRKDLLVIEICDGSAVSGVFTQNRFCAAPVTLCKKHLDAVKNNATTGGNVSGIKALVVNTGNANAGTGEQGMLDALTTCQHLAEIMAIPVESILPFSTGVILEHLPMDKLLAGLPLAVTNLTTDNWADAASAIMTTDIAPKAYATQVKVGGESINITGISKGAGMIHPNMATMLGYVATDANITQSLLDSMTKEIADLSFNCISVDGDTSTNDSFIVIATGKSNAVAITSPNDKGYQEVFDALLETSQYLAKAIVRDGEGATKFITVTVKGALSIDEAKTIGFSIGKSPLVKTAMFASDPNLGRVLAAIGYASRECDSLADLDTNQLELYFGGLLVAEKGGRAASYKEDEGQAIMNEAEIDITVQLHRGNEESTIWTCDFSYDYVKINAEYRT

>sp_Q12UL5

MKYIILIGDGMADHPLEELGGMTALQKANTPNMDQMTKNGLAGLAINVPEGYSPGSDVANMSVMGYDPALYYSGRAPLEAASMGIPLEVNDVAFRCNLITIRDGLITDHSAGHITSEEARELIEAVDAELGSEGLKFYPGISYRHLLVASNGLGANADCTPPHDVIDGEINDHMPRGDGSDVLGKLIEGSIPILEGHPINEKRISEGKNPGNSVWFWGQGYAPSFRTFEDLYGLTGSVISAVDLIMGLGIYAGLDVIEVPGATGYLDTNYVGKAEFAMASLKDKDFVVVHVEAPDEAGHMGDIEAKLQAIEDFDEKVVGTVLRAARESDEDYTIVVLPDHPTPIALRTHTSEPVPFVMYSTLEDEVDDVETFDEDAMKKGSLGIVRGCDLVQLMMERAKQA

>sp_Q47XQ8

MNKTQQQLLINAIHTIPDYPVEGIMFRDVTSLLEDAEAFKLVMELLENKYKGRGFTKIVGTEARGFLFGAPLALALNIGFIPVRKPGKLPRPTYSQAYQLEYGEDILEIHQDALTPEDNVLIIDDLLATGGTIEATTKLIRRLGAQVQEAGFVISLPDLGGEERLAELNITPYSLIQYQGE

>sp_Q48AV9

MALLTVNLNVVSAEESLFSGSIKSLQITGSEGELGIMPGHAPLLTSLKPGMALITKADGTEEVIYLSGGMLEVQPNNVTVLADVATRAADLDEEAALAAKQRAEDNMNAHGGDVDYAAVAAELARAVAQLRVIQATSKHS

>sp_Q48AW0

MSTGKVVQIIGAVVDVEFPQDAVPQVYDALKITEGDLEGLVLEVQQQLGGGVVRTIAMGTSDGLRRGLNVVNTGQGIQVPVGVETLGRIMNVLGEPIDEAGPIGEKDRWSIHREAPAYAEQSMSNELLETGIKVIDLVCPFAKGGKVGLFGGAGVGKTVNMMELIRNIAIEHSGYSVFAGVGERTREGNDFYHEMNDSNVLDKVSLVYGQMNEPPGNRLRVAFTGLTMAEKFRDEGRDVLFFVDNIYRYTLAGTEVSALLGRMPSAVGYQPTLAEEMGVLQERITSTNKGSITSIQAVYVPADDLTDPSPATTFAHLDATVVLSRDIASQGIYPAIDPLDSSSRQLDPLVVGTEHYETARGVQTVLQRYKELKDIIAILGMDELSEEDKQTVSRARKIQRYLSQPFFVAEVFTGSPGKYVSLKDTIAGFKGILAGEYDDMPEQAFYMVGGIEEAIEKANKM

>sp_Q48AW2

MQLNSTEIAELIKNRIEQFNVVSEARNEGTIVSVTDGIIRINGLADVMQGEMIELPGSRFAIALNLDRDSVGAVVMGPYADLAEGQKVKGTGRILEVPVGRGLLGRVVNTLGEPIDGKGPIENDGFSPVEVIAPGVIDRKSVDEPVQTGIKSIDAMIPIGRGQRELIIGDRQIGKSAIALDAIINQKNTGIKSIYVAIGQKASTVANVVRSLEEHGALSNTIVVVASASEAAALQYLAPYAGCSMGEYFRDRGEDALIVYDDLSKQAVAYRQISLLLRRPPGREAYPGDVFYLHSRLLERAARVNEAYVEKFTNGEVKGKTGSLTALPIIETQAGDVSAFVPTNVISITDGQIFLQSDLFNSGIRPAVNAGISVSRVGGAAQTKIIKKLGGGIRLALAQYAELAAFAQFASDLDDATRAQLEHGQRVTELMKQKQYSPLSIAETAVSLFAAEKGFLNDVAINKVVDFEEALHAYMSNEQAALMATINEKGDYNKDIEASLKTALENFKSTQTW

>sp_Q48AW4

MDINMTLVGQLIAFVVFVIFCMKYVWPPIIGAIEDRQATIADGLAASDRAAKDLELAQEKATAQLKEAKVQAASIVDAAKKHEAKIVDEAAGKAQVEKERILASGHAEIETERNRAKEELRKEVAVLAIAGAEKILERSIDAAAHSDILDKLVAEL

>sp_Q480H7

MRLCDKDIEQYLDDEKIIIEPKPDSSMISGVSVDIRLGNEFRVFQDHTAPYIDLSAPKGEVQEAMNSIMSDEIFIADGDAFFLHPGELALAVTYESVTLPDNIVGWLDGRSSLARLGLMVHVTAHRIDPGWSGQIVLEFYNSGKLPLALRPKMKIAALNFETMSSSAARPYNKREDAKYRDQKGAVASRISQDEKVNK

>sp_Q47YJ7

MSDRKYFGTDGIRGLVGQYPITPEFVMKLGYAAGKVLAGQGTKKVLIGKDTRISGYMLESALEAGFSAAGIDVGLLGPMPTPGIAYLTKTFRAEAGIVISASHNPFYDNGIKFFSNTGEKLPDAVELAIEAELDNPMGCVESAKLGKASRVNDAAGRYIEFCKSNFPSKISLKDLTIVVDCAHGATYHIAPNVFRELGATVIEIGTAPNGTNINQGCGATSMAAISKAVVEHKADLGIALDGDGDRIMMVDHTGYVIDGDEIIYIIACNDLKTGRKEGGVVGTLMSNMGLELALKELDIEFARSNVGDRHVMELLREKGWQLGAENSGHVINLNHTSTGDGIIAALNVLTAVTKQEKSLFELRQGLTMLPQLLVNVRFSGQNNPLNDADVLAAVDEVNETLTGRGRVLLRKSGTEPLIRVMVEGPDMDEVTLLANKIADLVKLVK

>sp_Q12TT4

MPDSLRPLLRKPFGVLYTGIGSDAVKSLVKDLNNPTKLISVGDVTTFHLLDSNIIPDILIVDDRTKRAPASSQVVFGTKHKGFAEITVDNPPGVITEDLINVISDAIVSDKNVRIFVQGEEDLAALPAILMAPLNSVVLYGQPDEGVMLVRVTESLKAELKDLFDKILEKQDHKEQLLHNVRRKLNGY

>sp_Q12X51

MRAEIVKDRVLVEKKAINEFYNNGYYGRPKSSGLELTLIEAVYLAFRGKIEVEHEGKVLEFSDLFKEASILQPSFELKYIVYKDLRERGFYVQPGVTDFRVYPRGSHPGKGAAKQFIYVRSERAPMPLRDLLRSLAAAENVRKQMVLAIVDEESDITFYDVKRPRLKGEMKEPLYPDINADATFLEDRVVVWDEEASKTLFENGFYGKPLDSQRLQLSLVESRYLLEKGVLNINNRQDESMDVDAFSKMASEIEPEFNLKSSVYTDLRDKGVVPKTGFKFGSHFRVYSQVESPTKIPHSEYLIHSIPMDHEFTLPVMSRAIRLANSVRKRMLYAILTDDGVDYIDIGRLKM

>sp_Q485F2

MSLNFLDFEQPIAELDAKIEELQLVNNGQELDLDIEDQISQLREKNKEQTKKIFSNLDAWQTARVARHPQRPYSLDYIPRIFTEFDELAGDRAYANDNAIVGGTARLDGKPVMIIGHQKGRSTAEKVKRNFGMPRPEGYRKALRLMEMAERFNMPIITFIDTPGAYPGVGAEERGQSEAIARNLKVMARLSVPIICTVIGEGGSGGALAIGVGDRVNMLQYATYSVISPEGCASILWKTAEKAPTAAAAMGITAQRIKELDLINSIVEEPLGGAHRDMDVMAAHLKQAIKKDLSELEGLSKDELIEQRYDRLMSFGYC

>sp_Q488Y0

MSHANPVQFINGQWQAGLGHDVSSSNPARNEVIWQGKTASKDQVNDAVLSARQAFESWANISLEARVAVVTKFAELLAENKDALATTIALETGKPKWETTGEAGAMVAKVAISLKAYNERTGTVENPMPGAKAFIRHKPHGVVAIFGPYNFPGHLPNGHIVPALIAGNTIVFKPSELTPRVAQEMLKLWEQAGLPNGVINLVQGEVETGKALASHKLIDGLFFTGSSNTGHILHEQFAGQPGKILALEMGGNNPLVVKDVSDIDAVVHDIVQSAFVTTGQRCTCARRLFIEANEQGDAILARLIEVTKNLTIGYYDDEAQPFMGSMISEKAALSLVDAQAKLLALGAKSVLDLKHLEVGTGFVSPGIIDVSDIIADIPDEEYFGPLVKLYRYNDFDKAIDEANNTGFGLSAGLLSDSEASYNHFFTRIRAGIVNWNKPITGASSAAPFGGIGASGNHRASAFYAADYCAYPIASVEAEKVSLPETLTPGMKF

>sp_Q12UJ8

MLKIGTFDLEERPAIVAAISNEPLQQCKTAAEHGADILEIRFDLLGITTSKEAANLLRMLKGTTSLPCIATNRLQSQGGNWEGTEENRIALLEDIMHLTDAVDIELETDEQLRDRIVKKAKEESKTTIISSHDFERTPDKETLKSILDHSHDAGADIAKLAVMPENMQDVLNLLEVTLEVDDVCTISMGKLGKHTRIIAPLYGSKLTYASVSDAVAPGQLKVEDLKKAMEMME

>sp_Q47Z95

MSKIEQKNQSEVIKLAIDLMSRASVTPEDAGCQKLMAQRLAQLGFTNESMIFADTTNLWSRRDSTNATKEDDLVFCFAGHTDVVPAGNLELWNTPPFEPTIIDGMLYGRGAADMKGSLAAMIVATERFVQDHPDHHGSITYLITSDEEGPFINGTTKVIDTLEARNEKITYCIVGEPSSTHAVGDIVKNGRRGSISAEVDIKGKQGHVAYPDHVRNPIHLAMPALTELSQVQWDNGNDYFPATSFQLSNINAGTGATNVVPGHINALFNLRYSTELTDQIIVEQVESILDKHQLDYDIKWTFNGKPFITEHVESEHGFLNAVSQAILSVTGTETQLSTSGGTSDGRFIAPTGAQVIELGPCNATIHQVNESVSCDDLEKLVDIYYHCLVNVLCTHKTIS

>sp_Q12VD1

MSIIDPVNKSKVPQEWINKSNIPGDELEEGIMSGMLVVLSDGSILKRGYTTGTTATVAAKAAVLSLKKEIDHVSVPTPVGLRAHMDVKAKDGHAVAVKLNNDHESDITRGLEFVARAVESDKITITAGEGIGIVTRGGLQSKKGYPAINPRPMQQIMEAVVEAVEEIGIKGASVEISLPRGAEIAKQTLNGRIGVEGGISILGTTGFVEPWNDHLGEMKSDLIRDAAKVVLTTGRIGIRYSTMLFPDYTVVLAGSRISEFMESATGKVAICGLPGLVLKWGDPDMLKDSGFATVSEMIEVEPQGEHIRRAFEKTVEKGKGARIVVVDRDGTVLMDSGEQE

>sp_Q489Q8

MTELKNDTYLRALLKQPVDYTPVWMMRQAGRYLPEYREVRKNAGDFMSVCKNAELACEVTIQPLRRFPLDAAILFSDILTIPDAMGLGLYFETGEGPKFERPITCKADVDKIAVPDPEDELGYVMNAVRTIRKELKGEVPLIGFSGSPWTLATYMIEGGSSKAFTKIKKMMFAEPQTLHLLLDKLADSVISYLNAQIAAGAQSVMVFDTWGGVLSPRDYNEFSLQYMAKIVDGLTRHNEGRQVPVTLFTKNGGMWLESIAATGCDAVGLDWTIDIENAKARVGDKVALQGNMDPSMLYAPLPRIEQEVSKILSGFGEGGTGHVFNLGHGIHPDVNPDHAGHFIESVHRLSKPYHK

>sp_Q487I2

MSKKNSTPLISAVNLTCIREERLLFDELSLQINAGDIVQVEGPNGSGKTSLLRILSGLSQPYDGQILYREQLISHCREEFHQNLLYFGHLSGVKGEMTAEENLDFNLALHGNKTQESLSYLAKVNLSGFEECLASHLSAGQHRRIALARLYQSNVPIWILDEPFTAIDKQGVASLERLFSLHAERGGCVILTTHQDLISIKPEQIKKITLDYSYDSAVD

>sp_Q12W53

MIIFSGGTGTPKLLDGLRHIVPEDELTVVVNTAEDVWVSGNLITPDIDTILYLLSGRIDRDKWWGVKDDTFQTHREMKELGHDESMMIGDLDRVTHIMRSDLLRQGLSLSESIHELLSVYGIGVNVLPMSDDSVRTIVETPSGHVHFQDFWVKQHGVPEVLSVEQEGIEEASICSLVLEALESDDEVLIGPSNPITSIGPIISLPGMSRILRKKKVVAVSPIIGNEAVSGPAGKFMTARGFDVSSRGIADCYREFLDVLVLDDRDTTSPEQFQKMGVDVVSTNTLMKSLEISKDLSKKIVSIFANI

>sp_Q47YD3

MNYLSNITTSQRPWLLLALAALGLELSALYFQYVLGLAPCIMCVYQRLAILAIFSAGAIGAIGHQNIVARILAYVLWGVGAIWGLIIALEHVEMQKNSGSLFFSCEFIPNFPTWAPLHEWIPFLFEATGDCGEISWRFFNYSMPQWMVVVYALFSIVFSIVLINRLVCAKKP

>sp_O74038

MDLEREYRAKTGGSARIFARSKKYHVGGVSHNIRFYEPYPFVTRSASGKHLVDVDGNKYVDYWMGHWSLILGHAPAPVRSAVEGQLRRGWIHGTVNEQTMNLSEIIRGAVSVAEKTRYVTSGTEAVMYAARLARAHTGRKIIAKADGGWHGYASGLLKSVNWPYDVPESGGLVDEEHSISIPYNDLEGSLDVLGRAGDDLACVIIEPLLGGGGCIPADEDYLRGIQEFVHSRGALLVLDEIVTGFRFRFGCAYAAAGLDPDIVALGKIVGGGFPIGVICGKDEVMEISNTISHAKSDRAYIGGGTFSANPATMTAGAAALGELKKRKGTIYPRINSMGDDARDKLSKIFGNRVSVTGRGSLFMTHFVQDGAGRVSNAADAAACDVELLHRYHLDMITRDGIFFLPGKLGAISAAHSKADLKTMYSASERFAEGL

>sp_Q47WD1

MSKDIHDHRILILDFGSQYTQLIARRVREIGVYCELWSWDVTEEQIKGFNPTGIILAGGPESVTEANSPRAPEYVYTAGVPVLGICYGMQTMAEQLGGGVESSSHKEFGYAAVELIAQSALFNKVEDSIGDNGNALLDVWMSHGDKVSAIPEGFVTVAQTASCAYGAMANEEKQFYGVQFHPEVTHTKQGSRILENFVVDICKCEKLWTSASIIDDAIAKMKAQVGDDEVILGLSGGVDSSVVAMLLHRAIGDKLTCVFVDNGLLRLDEGQQVMDMFGDHFGLNIIKIEAEDRFLNRLAGESEPEAKRKIIGNVFIDVFEEESNKLDNAKWLAQGTIYPDVIESAASATGKAHVIKSHHNVGGLPDYMKLGLVEPLRELFKDEVRKIGLELGLPYDMLYRHPFPGPGLGVRILGEVKKEYADLLRRADAIFIEELHKHDLYTKVSQAFTVFLPVKSVGVMGDARKYDWVVSLRCVETIDFMTARWSHLPYDFLGLVSNRIINEIDGISRVVYDISGKPPATIEWE

>sp_Q12XX8

MNFFMDIRDKVFAATGKVKADTIFFGGLLINVNTKEMLYRDIAVKEGYIVGIGDVSSLKGDETEMIDVTGKHLCPGLMDGHVHFESSMVTLSQFAVPALAHGTTSVVIDPHEIANVLGRGGIELVLDEAATLPLNAFVAVSSCVPATSFETAGASIDVDDIVSLIANENVVGLGEMMDYPGVVFCDETKLSMIRTALKERLVVDGHCPALSREQLFGYMCAGISTDHESIEYEEALEKLRLGMKLMIREGSAAKALDKFLPRLIGDGVSLENVFFVTDDKHPSDLLKGYMDVIVRRAIELGLSPLDAISMCTINAAKHYRVDHIVGSLSMGRKADIIVLEDLEKFIIDSVYASGRPVESFVPSYEYPDTVFNTVKFDAVTATDLQIMSDADKDHRVRVIKVVPDLIVTENETFVLHSDRHGILMPDVENDVLSVAVIERHGKNGNIGTGFIKGMGLRNGAIGQSIGHDSHNVVVTGVDHSDMALCANTIRSMNGGICVVSNGKVVEQLELPFAGLLSTLPAEEVEKKLTDLHKAVKEIGCALPAPFITHSFIALPVIPSLRLTDMGLFDVDKFSLVSPIDEVME

>sp_Q489N3

MAILNLDLGERSYPIYIDSGLINKTDLLSSHIRAKRVCIVTNDIVAPLYLDSLKAKLTDFEVDEVILPDGEAEKNLANFEVIISHLLTLEHGRDTTLIALGGGVIGDITGFAAACYQRGIDFIQIPTSLLSQVDSSVGGKTAVNHPLGKNMVGAFYQPKAVFIDIDSLTTLPIREFNAGMAEVIKYGILGDKEFFLWLEDNISAIKAGEKQVLAQMIEKCCQCKADIVASDEKESGVRALLNLGHTFGHAIEAEQGYGKWLHGEAVATGMVLAAKLALAMNLLEVSEFRRIEKLISAFDLPITAPKNMGFAEFIRHMRRDKKNIAGKLRFIIPTAIGQSEIRDDVTQDTLQEIL

>sp_Q489N4

MAEKRNIFLIGPMGAGKSTIGREIADRLHLEFFDSDQEIERRTGADIAWVFDLEGEEGFRKREETVIEDLSEKHGIVLATGGGSVISTNVRNHLSARGIVVYLETTIDKQVARTQRDRRRPLLQTSEEPRTVLENLAVERNPLYEDIADIIVQTDDQSAKVVAHKIIERLDF

>sp_Q12YX1

MIKTLVVDDSALMRRAIRDMLESADDIEVIGTAKNGKEAVENTNKLKPEVIVMDVNMPIMDGLAAVKAIMKTTPIPIIMFSSLTKKGSIEALEALRLGAIDFITKPSGLQEISKIENELVTKVRNLYNSNVNIIRLLNLKKFKGEVINGNWNCPDQNLGILIGSSTGGPSSLEQIIPRLPGDLPASVFIVQHMPEGNFCSQLAARLDAISELEVKEAENNEKVKIGVAYIAPGGYHMEIRKALDVTRIKIIKGKPMHAVMPSVDVTVESFVKVYGNNSVAIILTGMGVDGASGFKKINESNGATIACSEDTCVVFGMPKAAIEAGAIDVVKPIFEIPEQIVRMIEVKCNGN

>sp_Q485Q8

MPKASDIKKNAAIEHNGKVLIVRDITRSVPQGRAGGSLYRMRLYDVVTGGKVDETFKAEDMLNFADLSRRPSMFSYIDGEEYVFMDNEDYTPYNLNKESIADEILFIDEETQGISVIIVDDAPVGIALPASVELLITETDPSIKGASASARTKPALLSTGLTIQVPEHISTGDKVKVNTEERKFMGRADSK

>sp_P41561

MSTDNSKIIYTITDEAPALATYSLLPIIQAYTASSGINVETRDISLAGRILANFPKYLTKEQRIDDALAELGELAQTPEANIIKLPNISASIPQLEAVIKELQAKGYDLPHYPAEPQNEAEESIKLTYAKILGSAVNPVLREGNSDRRAPASVKQYARNNPHSMGAWSKESKSHVAHMASGDFYGSEKSVTIDGATSVNIEFVAKNGDVTLLKSKLPLLDKEIIDASVMSKSALVEFFETEINKAKEEDVLLSLHLKATMMKVSDPVMFGHAVRVFYKDVFAKHAATFEQLGVDADNGIGDVYAKIARLPAAQKEEIEADLQAVYATRPEMAMVDSDKGITNLHVPSDVIIDASMPAALRASGMMWGPDGKQKDTKFMIPDRNYAGVFSAVVDFCRENGAFNPATMGTVPNVGLMAQKAEEYGSHDKTFTMKAAGTVRVVNSQGERLIEQEVAQGDIYRMCQVKDAPIQDWVKLAVTRARATGTPTVFWLDENRGHDEQMIKKVNTYLADHDTTGLDIQILEPVKACEFTLARVAKGEDAISVTGNVLRDYLTDLFPILELGTSAKMLSIVPLMNGGGLFETGAGGSAPKHVQQFEKENHLRWDSLGEFLALAASLEHVAVTTGNARAQILADTLDAATGKFLDTNKSPSRKVGELDNRGSHFYLAMYWAQALAAQTTDTELQASFSSVAQALTKQEEKIVAELNAAQGPAIDLNGYYFADTKLAEKAMRPSETFNTILSALL

>sp_Q12VH0

MITKDEVEHVGWLARIEIGAAEAEDYAVKLSSVLDYFGQLDEVDTEGVEPTYHVADIMNVFREDVVKPSLDQKDVLANTAEEKDGYIKAPRII

>sp_Q47VY6

MSLAAKITVLGAGSYGTALAICLARNGHKTLLWGRDDNHVAAMEQDRENNKYLADCPFPENLALEADLEKAVQASDNLLVVVPSHAFADMLKQIKPMLTENAKIAWATKGLDPQTGDLLQNVARTVLGDRVSLAVLSGPTFAKEMASGLPTAISLSSEDDEFVAELSDLLHCEKRFRVYSNKDFIGVQLGGAVKNVIAIAAGIADGIGFGANARTALITRGLAEMTRLGLALNAEPATFMGMAGLGDLVLTCTDNQSRNRRFGLALGQGKEVEQAITDIGQVVEGYRNTKEVYMLAQRHDVEMPIVEQVYQVLYRGKDAKLAAADLLSRDKKFE

>sp_Q488M4

MEFSVKSGSPEKQRSACIVVGVFEPRRLSGTAEQLDEISEGYISNLLRKGDLEGKSGQMLLLHHVPNILSERVLLVGCGKERELDERQYRQIITKTINTLNETGSMEAVCFLSELHVKGRDIYWKVRQAVEAAQDGLYSFDSLKTRKAEARRPLRKVVFNVPTRRELPIGERAVSHALAIAEGITTCKNVANMPPNICNPAYLAEQAKILENDYDKVTTTIVDEKEMEELGMGSYLAVGRGSVNESLMSIIKYDGAGDDSKPLVLVGKGLTFDSGGISLKPGAGMDEMKYDMGGAAGVLGAMHALVELNLPINVIGVLAGCENMPSSNAYRPGDILTTMSGQTVEVLNTDAEGRLVLCDALTYVERFNPEAVIDVATLTGACVVALGAHATGLLSSHNPLAHELLNASEQSGDRAWRMPLWDDYQDQLESPFADFTNLGGKEAGTITAACFLSRFTKKYNWAHLDIAGTAWRSGKNKGATGRPVSMLTQFLLNRSGQEQGE

>sp_Q12U14

MLAKDSLSWVVTVVTLTGIFLTAAVLTGIDLVWYLFYMSLFLTFFVIWFFRDPDRTTRICDHCMFSAADGKVMDVSGRRVCVFMNVHNVHVNRTPISGVVKSITHKKGGYLPAFHKDSERNERTVTVIKSSHGEVNVTQIAGVMVRRIVSYINVGDELVNGEKIGMIRFGSRVDVTIPDDFDIACKVGDRVYAGETVIAKKKNFKVRK

>sp_Q47XG4

MKNTYTSGELEQFFSSDLSSTDGAVQVAIDLEEARQNQQIELIASENIVSKAVMEAQGTVLTNKYAEGYPGRRYYGGCEHVDLVETLAIDRAKLIFKADFVNVQPHSGAQANGAVMLALVKPGDTILGMSLDAGGHLTHGAKPAQSGKWFNAIHYGVRKDDMRIDYDQVLALAIEHQPKMIIAGGSAIPRQIDFAKFREIADQVGAILMVDMAHIAGLVAAGAHQNPLPFADVVTTTTHKTLRGPRGGLILTNNPDVAKKINSAVFPGLQGGPLMHVIAAKAVALGEVLEPSFGAYIKQVLSNARVLASTLQQRGCDIVTDGTDTHLMLVDLRPKGLKGNTTEESLERAGITCNKNGIPFDSEKPMVTSGIRLGTPAGTSRGFGNDEFELIGQWIGDVLDGLVANPEDNSVAEQKVLQQVQQLCLRFPLYS

>sp_Q487D1

MAKKKLENLSFEESLNELDTIVQSLEQGELSLEESMTLFERGLNLSQLSQVKLQAAEQKVQILLDKNGTAKLTDFDSSAGES

>sp_Q480P2

MAKEENIEMQGTVLDTLPNTMFRVELENGHVVTAHISGKMRKNYIRILTGDKVTVELTPYDLSKGRIIFRAR

>sp_Q12YN5

MKKNNGGRGSKTDAPAVTRVRTPRRENNEILATVSALLGGKRVTLQCMDGIVRMGRIPGSKKKRMWVREGDIVIITPWDFQDSKAEVIWKYTRPQVEWLERKGFLK

>sp_Q47U36

MTSISQTTTIAAQATAPGRGGVGIIRVSGPEAKNVAQAILGKLPEVRKAEYLPFLDCTSTDKTQVLDQGIALYFKAPNSFTGEDVIEFQGHGGPVILDMLLKVILAQPKVIMAKPGEFSEQAFLNDKLDLTQAEAIADLINSSSEQAARSALHSLQGDFSKLVNEMVESIIHLRMYVEAAIDFPEEEIDFLADKKIVTDLKAIISRVEDVRKQAQQGSIIREGMRVVIAGRPNAGKSSLLNALSGKQTAIVTDIAGTTRDVLAEQIHIDGMPLHIIDTAGLRDSDDVVEKIGIERAWQEINQADRVLLMVDASEDHSILEDDQDIKDFYPEFFAKLPEKIGLTLIRNKADVNDAKTGFTEFTDTDGTQHAIITLSAKTGKGVDSLKEHLKTIMGYQGGTEGGFMARRRHLVALENTHQHLLTGLDQLESYVAGEILAEELRICQQELDQITGEFTSDDLLGKIFSSFCIGK

>sp_Q47VJ4

MDAFKVIGGKPLRGDVVISGAKNAALPILMSALLSKTPVVFSNVPQLNDILTTVKLLGQLGAKTKWLNEEKLMIDASSIDVCRAPYDLVKTMRASILVLGPLLARMGHAEVSLPGGCAIGARPVNLHIQGLKLMGADITVEDGYIVAKKQGRLTGATIFMDTVSVTGTENLMMAAALAEGITIIENAAREPEIVDLANCLISMGAKITGAGTDTLTIEGVSELCGKEYSVMPDRIETGTFLVAAAVTQGHIKCLNTDPSSLEAVLSKLQEAGATITTGDDWIELEMSAPAKAVNVRTAPHPAFPTDMQAQFMTMNVLAEGTATVIETIFENRFMHVPELQRMGADIALEGNTAIVKGVASLNGAQVMATDLRASASLVIAGLVAKSPTQVDRIYHIDRGYLCIEGKLQSLGADITRIKVD

>sp_Q12TR2

MTNENLCAGRNRYDGVSIDFFSDADLRAIDSATMDVFQNPGIQVSDAESRALFKEAGCDVNEKTMVVKIPEHVVRRAIMTAPSKITLYGREKQHTFTQQAGGKVHYTCFGTGVKMCKYEAPGVFKTVDSTEEDLANTARVCDWADNIDSYSLAVSARDWAGKGAQDVHETFTPLMNTSKHFFHIDPVGENVEYYWDILKAYYGGDEEQARSRPIFSELLCPTSPLKIGTNACQLILKSVNFGIPINVISMAMSGASCPVHLAGTLVTHNAEVLSGIVLAQLASPGAKVWYGSSTTAFDLKHGTAPVGSPELGLISAAVAKLGQYYDLPTYVASTOTDAKVPDGQAGHEKTLTNLLPALAGANTLYGAGMLELGMTFSMEQLMIDNDIISMGKKVMKGIPVNDETLGLASIQKVGIGNNFLAHKETRDNINLVSSPDIFDRDMFGDWAAAGSKDIATVAHEKVTEILKNHEVTPIDSDLVRDMKAVVDRADADFRSSM

>sp_Q482J5

MSTGKFIVIEGMEGAGKSSAIAVIESTLNKHGIEYINTREPGGTPLAESLRDMVKSVDHQEKLTVETELLLMYASRSQLLANKILPALAAGKWVIGDRHDLSSRAYQGGGRGFDETIMNTISDITLKGFRPDITLYLDIDPHIGLSRAKARGDLDRIELEKMEFFIRVHNKYRELAEQDDSIITVDAAQAMLKVHQDVEKAVIGFITNTDKG

>sp_Q47VK1

MFFSRTLSFTLVALLVVCLSQKAHANVEQKSQSPETRLMQCPLPEFDDIAIGIPEVVDSTIRISSSQASIQQDQIALFNGSVILVDKGQKIIADQLSFNRLKMQIEAIGNIHYQGKQINIFADKLSASKIDKSTEMTAASYQLDGNPGHGKAGKLSINSDGMMSLVDSTFTTCLQEVPDWQIKASEINLSAKGDFGEAYHAQFRVLDVPVFYVPYFSFPISKERLTGFLYPELSTSSSLGIEFTAPFYWNIAENYDATITPRYMSERGTQLQTEFRYLMDQQSGKIDLEYLNKDKKIKTNDDPRYLARFQHIGTFSEDFRAYIDYTTISDDNYLVDIGSKQYNSNDAYLYQIGELAYFGEQWQATLQLQDFEVLGDHQSSYKTLPHLELSAQQPLNFLSGQFELYSEMTNFQPSEKDQVSANRYHVEAGFTFPITRPSWFLNSEVKLMHTYYQQDNISAGSSLEETVDRTLPKVRIHAGVNFDRQLLAFGQSYRHTLEPQLQYLYVPDEDQSNIGLYDTTILQDDFHGIFRDTRYSGLDRIAAANQFTWGITSRLLDEENLEIIRVSLGRIQYLGENNTDLANDALLTDNGGIDNKQSSVAADLFYRINHQWQVSGDIQYNTLEDFTNKGQVNLDYQINKYNLIQLNHRYTRNVSGDSLEQASLLTSIAINENWAFVGRLTQDLQQDRSLESYAGFQYESCCWAVRIAYQRHINSNLDAASFVSEGREQFDTGISIKLIIKGLDGKQSAIGTQEMFDKSIFGYKRPYYLQN

>sp_Q485F5

MTTQNQNTHQQTVFAMVVGEHSGDTLGAGLITSLRQTHPHAKFIGIGGPKMLALGFESLFAMDELSVMGLVEVLGRIRRLLHVRKTLTDFFITNKPDVFIGIDAPDFNIGLELKLKVKGIKTVHYVSPSVWAWREKRIFKIAKATDMVLALLPFEKAFYDKHNVPCTFVGHPLADDIPMQSDKVLARDKLGLAQDKKILALMPGSRGGELSRLLEDFFESAKQLQAQDSELLFVAPMISEQRANQFNALKAELAPDLDIEIVLNQTQQVMAASDCLLTASGTVTLEAALIKRPMVICYKFSPITFFLGRRFVKLKWFSLPNLLTNKSLVPELLQKDVCPENIVPLVKERLYQDQSQLNDSFTAIHQQLKCDASKQAAKAVLDVLSSKLLSNNK

>sp_Q47YG8

MSKVLQCCQLSKSYIQGDIETKVLNDLELSVDKGELLAVVGSSGCGKSTFLHLAGALDSPSSGKVLINNIDIHQLSDKERAKFRNEHIGFIYQFHHLMMEFNAQENVAMPLMIRGEKPKDALLAAKEMLDQVGLSHRIDYRPSQLSGGERQRVAIARALVTKPSLVLADEPTGNLDSDTAEQIYQLIRSLNKTAQTSFVIVTHDLVLANRMDRQVKLVQGQLRPLSDNSEQALPPTSSITDPANNIKDNEPQANERHV

>sp_Q12XS2

MSERSVGIVATNYHTIEGEFQLEGGHTLKNIRLAYETYGNLNKEKSNAILVCHALTGDAHAAGRHSDDDKKPGWWDDIIGPGKALDTDRYFVLCSNVLGGCKGTTGPASLDPDTGRQYGITFPVITIRDMVNVQKRLIDHMGITTLFAVVGGSMGGMQTLQWCVAYPELVKKAVVIASTAVSSPQQIAFNEVGRNAIISDPDWNGGDYYEGEPPVNGLSTARMIAHITYLSDASMHEKFGRRLQQGESYKFDMSNDFQVGSYLKYQGDTFTGRFDANSYLYATKAVDYFDLSMNGSLAEGLKYVQAKMLVISITSDWLYSPYHSKKIVEGLTVKEHDVSYREIESSYGHDAFLLESGQINYVIHNFLTHTSVADVMTEKVATIREGASIDTAAKVMFEEALTHLPVVNENGCLVGIVTSWDISKAVALKCSKLENIMTRDVLTAFPDEPIVAAAKRMERHSISALPVVDEKNRLIGIIDSEDINRLIG

>sp_Q47Y80

MSKKLYIKTWGCQMNEYDSQKMAELLDSTHGFSLVEEAEQADVILLNTCSIREKAQEKVFHQLGRWKNLKDKKPDLLIGVGGCVASQEGDSIRKRAPFVDMIFGPQTLHRLPEMLNQLQHSKSPIIDVSFPEIEKFDRLPEPKADGASAFVSIMEGCSKYCTFCVVPYTRGEEVSRPLDDVLYEIAQLAEQGVREVNLLGQNVNAYRGETHDGSICRFADLVRLVATIDGIDRIRYTTSHPVEFTDDIIEAYTDVPELVNHLHLPVQSGCDRILTQMKRGHTALEYKSQIRKLKKVRPELSMSSDFIIGFPGETDEDFTATMDLIKAVDFDLSFSFIYSARPGTPAADLPDDISDQTKKDRLKLLQEQITHQALRIARQMLNTEQRVLVEGPSRKNPMELRGKTENNRTVNFVAPHSVIGQFVDIKITDVVANSLRGELVREEKEMGLRIAHSPADILANNHHMATPSNLDELGVGTFTP

>sp_Q47VS4

MSKLVIGLTGGIGSGKTTITNYFLALGVEIIDADIIAREVVAINSPALKAIAKHFGDDYIQADGQLNRPLLRNRIFSNKADKLWLNKLLHPLIRVNIVTQTKEAKSPYCILVAPLLIENNLLELVDRVLIVDVNESTQITRTLVRDSSSEQEIKAIIASQTSRAARVNVADDIINNDDSPLSEIKEAVLSLDKKYLTLTKMV

>sp_Q12WF3

MRAVIPYKNENAKSRLSPILSKKDREEFVELMLKDVIKALDDAEVVNIDILTTSAEGIPNDFNGNVTITEPGLNDSINEYLQNANEPILIIMADLPLVTGDHIRKIISFSEDVVIVPGKGGGTNILFIRHPNEFTVKYHDCSFISHCEITDELDKSMHIFDSFLASTDIDEPHDIVELMLHGKGQAKEYAEKRFGSETGKGRVKISHLSKLSGFV

>sp_Q12XH1

MTLLSQNELRELVLANPPLVENMIDMDTQLQPNGVEMTLKEIRTIKSPGAVDFDNSGRRLSEGDTIEFNEDGWIHLDPGVYKVLLNEIVNIPKDLAAIAKPRSTLIRCGATLETAVWDAGYSGRSECMIVVHNKDGFDLKKDARIMQLLFYHLHTEVEEGYSGSYQNENI

>sp_Q12UQ4

MTEDNEVQIAGMSIVIVCSVVDPASQNIKEHLLKLRDWVEMSVPGGIFDDLSAVYQSGNFYIIEVTEHHIYQDGIDRKIEEAGLDCDLLIFASKHKSADGRRLLTAHFTGNPGSADFGGYPGELSMAAPFALRCLLRNMAELSESIGFDVSMESTHHGPSDLDVPSVYAEIGSSEVEWVDQDAGDIVARSILSVRSGFCPVGIGFGGGHYAARQSELVLGSDISFGHNFPNYQLQFVDVDMFRKAVERSGADLVYCDRKAMSSDEKKRINELADEFGLDVLRESDIKGMEGVCWDIFRIFWHKVRDEGLSGRVKVPVGLKDKLSENVCDIFDFDVSNVVTVVIDNELLKLVRSVDAGGVKRLLDMSNVVYSERDDATISNHFYTFWNRDAEDFLTFIVDECIKILKGRYDTEYVFEENVLYISDERFSPELARKWGVPSGPMFGELAKGQSVMIEGNTVLPEMVHERTQKSLVLRNVIF

>sp_Q485N9

MKTVINIFILFTFLASLSANAQRIKDLADVAGVRSNQLIGYGLVVGLPGTGEQSPFTEQSFKTMLSNFGITMPDKLKPKIKNVAAVAVHAELSAFTKPGQTIDVTVSSMGSAQSLRGGTLIQTILMGIDGNAYAVAQGSLIVSGLGAQGLDGSQVLVNIPTVGRIANGGIVEREVKSPFSSGDHITFNLRHSDFTTAKLLSDTINDLIEGSAKALDATSVRVRAPRDISDRVSFLSVLENLEFEPASPAAKIIVNSRTGTIVIGSEVTLLAAAITHGGITVTINEIQDVSQPNAFAEGETVVTNQSDINVSNSDARMFVFKPGVTLETLVRAINEVGAGPGDVMAILEALDQAGAIRGELVII

>sp_Q12UT1

MGTDIGDLLLKDTIEIAGLSNKVVAIDAYNTLYQFLSIIRQRDGTPLKDSRGQITSHLSGILYRLTSLIEAGVKPIFVFDGKPPDFKSDTLAKRHEVRESATAKWEDAKAQGLEEEAYKYAQASSKVTREMIDDSVRLLELMGIPYVKAPSEGEAQASYMVQKGDADYIGSQDYDSFLFGAPQVVRNLTITGKRKLPKKNIYVDVKPEVLSLVDSLGELGITRQQLIDIAMCVGTDYNTGLENIGPKRALKLVKEHGDIKVVLKELGKDIEDLDAKRDFFMNPPVTDDYELKWIKPDRAGVIDLLCKKHDFSEERVNKALDRLEANIGGSQSTLDQWF

>sp_Q47VM6

MTTSMPDIANHTTAQTEGTLDWVGMSNIEMPIMVASKGESERMVSAHIDAFVNLKDAQAKGIHMSRLYLLIDELSTSNILNYQSLVSLLDGFISSHQELSDQAKVQFCFDYHLRRKSLISGKEGWKAYPVTLTGNLNQGKLTIELTIDVPYSSTCPCSAALARQLIQKAFQDKFAQQSELALTDVHDWLGTTEGIVATPHSQRSVAEVKVKLNSSINDFPITEIVDLVENSLKTPVQAAVKREDEQEFARLNGQNLMFCEDAARRLQHSLNQTDQFDDFWLRINHLESLHAHDAVSVTTKGIKDGYQP

>sp_Q47VY2

MANKKSTVLMILDGWGYREETSSNAIHQANTPVLDNLKAKYPNMLIDTSGMAVGLPEGQMGNSEVGHVNLGAGRVVYQDFTRITKAISDGDFIENPTLCHAVDTATSNNKAVHIFGLLSPGGVHSHEEHIFAMMELAKKRGAQKVYLHAFLDGRDTPPRSAQASLEKAQQKFSKLFTETDTGEGQIASVIGRYYAMDRDQRWDRVEAAYNLMVNGEGLHQYNSALDALAAAYERNENDEFVGASAITSPSGKAIKVNDGDALIFMNFRADRARQFSRCFTDTNFNGFERKRIPAISNFVMLTQYAADIDAPSAFAPTPLTNVMGEWLAKHNKTQLRISETEKYAHVTFFFSGGKEDMFTGEERILVPSPDVATYDLQPEMNSTLLTDKLVGAIESGKYDFIVCNYPNGDMVGHTGSFDAAVKACEAVDTCVGRVVKAAQDNGGECLITADHGNAEQMQDPVSGQAHTAHTCEPVPLIYVGRNASPAASGTLSDISPSVLHLMGMEQPQEMTGSVLMQLIK

>sp_Q486Z0

MKLPIYFDYSATTPVDKRVAEKMMQYMTNDGHFGNPASRSHKFGWQAEEAVDIARNQIAELINADPREIVFTSGATESNNLAIKGAANFYNKKGKHIITCKTEHKAVLDTCRELERQGFEVTYLDPEENGLIDLNKLNDAMRDDTILVSIMQVNNEIGVIQDISEIGEMCRARKIVFHVDAAQSAGKINIDMQALKVDLMSISAHKMYGPKGIGALYVSRKPRIRLEAQTHGGGHERGMRSGTLATHQIVGMGEACRLAKEEMAQDQAHVTAMRDRLWAGLNSMEQVFINGDADKRYPGNLNVSFNFVEGESLIMALKDLAVSSGSACTSASLEPSYVLRALGLNDEMAHSSIRFSFGRFTTTEEIDYAIELIKGAIGHLRDMSPLWEMFKDGIDLDSIEWAAH

>sp_Q47WB0

MPQSQRPLYIPYAGPSLLETPLLNKGSAFSKEERGSFNLTGLLPPRFESIDEQAERAFRQYSCFQTNINKHIYLRAIHDNNETLFFKLVQNNLAEMMPIIYTPTVGDACEQFSDIYRSSRGLFISYEDRFNIDDMLRNATKNKVKVIVVTDGERILGLGDQGIGGMGIPIGKLSLYTACGGISPAHTLPVMLDVGTNNQKLLDDPMYMGARHKRIDQDSYDEFLELFISAVKRRWPNVLLQFEDFAQPNAMPLLQRYKDRICCFNDDIQGTASVTVGTLLAACRSKGSKLSELNVAFVGAGSAGCGIAEQIISQMMNEGLGAEQARSQVFMVDRFGLLTQGMGELRDFQQKLVQSNEAIAEWDIAGEFASLVEVMHGAKPDILIGVSGQAGLFTEKVITAMKSHCEMPIIFPLSNPSRQVEATPSQVINWTQGQVIIATGSPFDPIEYQGKTFPIAQCNNSYIFPGVGLAVVAANISRITDGMLQVASETLAAASPLANGESDELLPPLTSIAQLSRDIAFAIAKVAYKQGLALELTDDELLAKIEHNFWKPEYRQYRRTSL

>sp_Q481H1

MALLDYFLRKKEKQVTTASKAKERLQIIVAHERNSRNKQPDYLPQLTEDILKVLRKYIKVSDESFSINLDKKDGDLNVLELNIELHDEQTAD

>sp_Q12VA2

MEENGKTKEEILLFLKKAKSADASYERVLSSMCTYPHEIAVLAHTQFIESNMGDPGLFPGTFNLEKQVLAMFGKMLHHKNSPEKAGYLTTGGTESNIQAIRSMHNFRHDISRPNIVMPESAHFSFDKVANLSGIEIRKASLDKLLKVDLDSVRSLIDKNTIGLVGIAGTTEFGQLDPINELSKIAIEKGIFLHIDAAFGGFVIPFMDIDYTYDFRLEGVTSMTIDPHKMALSTIPSGGLLFKEPEYFECLEIHTPYLSVNKQYSLTGTRSGAGVASTYAVMKHLGRKGYKKVVSDCMSVTKKLVDGAEKLGINTVIDPVLNIVALDVPEADLVRKKLLDEYGWHVSITRNPRALRIVIMPHIKNETIELFLKDLAKVIK

>sp_Q487E8

MRIGHGFDVHKFGGKGPLVLGGVKIPFELGFIAHSDGDVAIHALCDAILGALCLADIGNHFPDTDGQYENISSRILLRHVVSLMTDKGFQLGNADITIVAQAPKMAPHLVAMRTCLSEDLKTTIEQVNVKATTTEKLGYTGRKEGISVHAVVLLIAIEHPLPLIEEIQNI

>sp_Q48A23

MSVQSITESNQPPVICLMGPTASGKTALAMALKDALPCDIVSVDSALIYRDMDIGTAKPTKSELVQYPHRLIDLRDASESYSAADFCRDALVEIAEIRSNGRIPLLVGGTMMYFKSLIEGISPLPTANPEIRQAIEAEALSKGWQAMHDQLAEIDPVSAERIHPNDPQRITRALEVYRLTSNTLTQLTQIKGAKLAGDVLQLAITPRERSTLHERIALRYQQMIDLGFEQEVIKLKSRDDLHQDLPSIRCVGYRQMWQHLEGEFDHDEMIFRGVCATRQLAKRQLTWLRNWPDLHWLTTDDKTNLAQVLSLLEAKH

>sp_Q47WC5

MLPVVALVGRPNVGKSTLFNRLTRSRDALVADYPGLTRDRQYGQAEVEEHPFIVIDTGGINGDEQGIDVKMAEQSLMAIEEADAVLFLVDARDGLTAADHGIADHLRKQNKKIFVVANKIDGIHGDSAVAEFYSLGLGEHVHQIAAAHGRGVTQLLTIALTPHIEELGKPKVEEGEASEGEFDDAFFSDKDVELTEEELAKKLEDEPQENDKIKLAIIGRPNVGKSTLTNRILGEERVVVYDMPGTTRDSVYIPMERNGREYTLIDTAGIRRRKNVTDVVEKYSVIKTLRAIEDANVCLLIIDAQEGISDQDLSLLGFILEAGRSLVLAVNKWDGLEDHEKDRIKTELDRRLGFIDFARVHFISALHGTGVGHLYESVEEAFVSATKRISTAMVTKILDMAVFDHQPPLHQGRRIKLKYAHAGGYNPPIIIIHGNSAKKLPMSYKRYLMNYYRKSLKMMGTPIRIQFKDTLNPFAGKKKLNYTEQKKIARATQGYKKD

>sp_Q12X08

MTDFKEFADVCKQIEHISSSLEMTDVVSDMLKSISTEELPVVTHFVMGDVFPAWSVEQLGVGTSLLYSALSESSGLSLKEIEDLVRSTGDIGETAVAALGKKKKNKKKNQASLSFFSEDAASVSISIMDVFERFLDISRYSGAGSQSSKMRNLQFLFNSSSSEEARYLARLTIEDLRIGVGEGIVRDAISKAFDVPAGDIERGFMLTNDLGLVAVAAKEGGIEEISKLRMELDRPIKMMLAQVTPSIEAAIKDLGMLAVEWKFDGARVQIHKKGDSINIFSRRLENVTLSLPDIVEAVKLHVKADSAILEGEAVAVDENGAPRAFQDILKRFRRKYDVETMVREIPLTLNLFDILYLNGDVLMDQSLLRRREQLVACVENCDSIKVDEQVLTDDVNVVNDIYAAALNGGHEGVMLKNPEASYSPGKRGKNWLKKKPIMETLDLVVIAAEWGYGKRANLIGSYALACFDPEDGKFLPIGKVATGFSDEQLAELTEVFSELIIGESGREIELKPDVVFEIAFEEIQKSTNYGSGYALRFPRLVNVREDKSPEEAETIDRIESIYLSQRG

>sp_Q12XM4

MTKVKVAINGYGTIGKRVADAVALQDDMEIIGIAKTRPNFETVMAKDKGFNVYTLADRVGAMEKEGIEVSGTVEEMIKAADVVVDCTPGKVGATNKDLYEKAGIKAIWQGGEAHTLTGCSFNAETNYDEALGKDFVRVVSCNTTGLCRVLSPLDKEFGVKKARVTLLRRAADPGDIKTGPINAIVPNPIKLPSHHGPDVKTVIPNIDIATTAVKLPTTLMHLHTINLELEKECTAEDVESVLAEQSRVRFVGQGITSTAEIMELAKDLGRSRGDMWENCIWNESITMYEGELYFFQAIHQESDVIPENIDAIRAMMELESDASRSIEITNKTMGI

>sp_Q47ZS6

MALTKAEVAEHLFEKVGLSKRDAKDMVEIFFEEIRETLESGDQVKLSGFGNFDLRLKSERPGRNPKTGEDIPISARKVVTFRPGQKLKSRVEDGNSD

>sp_Q12Z93

MVVKDNLRTPIVCVMGHVDHGKTSLLDMIRGSAVVSGEAGAITQHIGATEVPISAIVEKCGNPGLLDKFMVPGLLFIDTPGHHAFTTLRSRGGALADLAVVIVDINEGFKPQTIESLNILQQHKTPFVVVANKIDKIHGWNPQKGAPFMTSYNKQSEHVRGSLDTKFYEVVGELYNHGFSSDRYDRVNDFQHNIGVIPISAITGEGIPDLLMVLLGLAQRFLESNLHYNAEGPGVGTVLEVKEERGLGTTLDLILYDGVLKKGDTIVVGCLGEPIQTKVRAVLKPRALSEINVEDKFKQVSKVTAAVGVKISAPHLDGALSGGSVRVATAETLDAVVEEVRNEIEDVQIDTDQSGITIKADTIGSLEALVNELKKEDIPIRKANVGDISNRDIMEAFAIEDPFHSVIVGFNVNILPDAKEKVRSTGVKVFMNDVIYRLIDDYRDWVKEQRAISEKAVSETIVKPAMFTIMPDCVFRQSKPAVVGVRVIGGTIKTKVDVATGDGTVVGIVKGLQSRGENVSVATIGMEVAMSIEGPTVGRQINEGDILHANIPERHVKILEQELYDSLSADELEALDSFLDIKRRDNPFWAK

>sp_Q12UV8

MTDADMSVLNEVFDVIMDRKNNPVEGSYVCSLLDHRKGINKILEKVGEETAETILAVKDNDRAEIIYETSDLLFHLLVMLAATDITLDDIAEEMKKRRH

>sp_Q48AW8

MNNTTVRIATRKSALALWQAEYVKAQLEHFHDGINVELVPMTTKGDIILDTPLAKVGGKGLFVKELEVAMLEDRADIAVHSMKDVPVDFPEGLGLEVICPREDPRDAFVSNTIKSLSDLPQGSIVGTSSLRRQCQLKASRPDLDIRDLRGNVNTRLRKLDEGQYDAIILAAAGLIRLEMSERIAQFIEPEEMLPANGQGAVGIECRNDDATIKALLAPLECATTRIRVLAERAMNRALQGGCQVPIGSYGVISADGKNIHLRGLVGSVDGSEMIESEITGPVEEGEALGNKLAQELLSRGADKILQQVYSENDIKNS

>sp_Q482G4

MQESTPVITIDGPSGAGKGTVARIVADQLGWHLLDSGAIYRVLAVAIQHHQLSLDDEEPLIPMAAHLDVQFEINSQGEAKVILEGENVTEIIRTEEVGGLASKVAAFPRVREALLRRQRAFSVSPGLIADGRDMGTVVFPKAPVKIFLTASAEERADRRFNQLKEKGIDVNIGRLLDDIRQRDERDQNRKVAPLIPAEGALTIDSTDISITEVVNKILMFANGKLT

>sp_Q12W64

MGNKLRKCTQCNIYTLKDNCPECGESSGNPLPARFSPLDTYGKYRRISKKREMEHA

>sp_Q47ZC7

MIEVIILAIALSMDAFAVSIGLGATKQQSKVAPLGIIVALYFGLFQGIMPIIGYLGGKGVLSWAESYTPWIAFLLLFLIGVKMIFDSFSEGIEEDISKITHRVLLILAIATSIDAMAAGFSLTLLPVNPLIACLIIASVTFIFSWLGVLVGTKGGTWLENKAEFVGGITLIVMAIKIIITS

>sp_Q487F0

MRVFTAILLILLVLLQYRLWFGKNSVPDYLVLKENVVRQQSANEKLQQRNKLLFADTDDLKLGLEAIEERARNELGMIKENETFFRLIPKENSTRNVNN

>sp_Q483H8

MTHKVKIHRLSDLSAEQRNKLLQRTESNLDNFIDIVKPIIENVKLNGDKALSEYAKKFDKAEVSTDQIQVTQAEFDEAFTLVDEEVIQTLSYSIDNIKKFHEAQMPEEMWMKQIRPGCYAGDRFTPINAVACYIPRGKGSFPSVAIMTAVPAIVAGVPTAIIITPPGTDGKVDAATLVVAKLVGIDKVFKCGGAQGIAAVAYGTNTVPKCDKVVGPGSPFVVAAKKLLADIIHPGTPAGPSEAIVLADDTANPKLAALDLLVEAEHGPDSSAFLVTNSKELAEQAQVAINEYWQHMDRLRVDFSSTVLSGDNGGIVLTSTFEEAVDFCNDYAAEHLLILSKSPFDHLGKIINAGEILLGENTPISIANYTLGPNAVLPTSMAAKTASPLSVFDYLKSCSIGYLTREGYEELAPHTYRFAKYEGFDAHANAVSHLRDEAIKSEKKIK

>sp_Q47XB8

MINQLINWQELSPQQKNSALARPAIADSALLSTQVANILSQVKNQGDKAILALTEQFDGIALSTLSVSSAQVAQAKLALTDKRLKAIHTAYKQIKSFHSAQTASDITVETTPGVKCTLKTEAIESVGLYIPAGSAPLPSTVLMLGVPAQLTGCQRTVLVCPPDKNGQLADEILVAADLCGITEIYTVGGAQAIAALAYGTETIPAVNKVFGPGNRYVTEAKTQLSQQVAGFAIDMPAGPSEVLVIADGQANPAFIAADLLSQAEHGVDSQVILLSDSESLISKVSTEIAQQLTLLSRCKIAEQALKQSRLILTKDLAQAVEVSNEYGPEHLIIQTEDAPTLLSKLRNAGSIFVGAYTPESAGDYASGTNHVLPTYGYSKVISSLSLADFSRRFTVQEITKAGLQSLAECIIELTDAEGLDAHQRAVTIRLEEGS

>sp_Q47V96

MNKSEQLFEQAQKIIPGGVNSPVRAFNGVGGTPCFIKRAQGAYIYDADDKAYIDYVGSWGPMILGHNHPAILEAVITTAKNGLSFGAPTEIEITMAEKVRELVPSMESLRMVSSGTEATMSAIRLARGYTGRDKILKFEGCYHGHADALLVKAGSGALTLGVPNSPGIPEDFAKHTLTVSYNNIDEVKEIFAKYADEIACIIVEPVAGNMNCIPPVEGFLEGLRDVCDQYSSVLIFDEVMTGFRVALGGAQAHYNIKPDLTTLGKVIGGGMPVGAFGGKQEIMDYIAPVGPVYQAGTLSGNPIAMAAGLASLTELAQGNKHQQLSSATEKLAMGLKAAAERNGVSLSVNYVGAMFGFFFTEDKNPITTYEQATQCDGEMFKRFFHLMLDEGVYLAPSSYETGFLSTSHTDDIIEKTLVAADKCFAQL

>sp_Q47XB5

MTSAKNVIVDTGCANLSSVKFAVERLGFEVTITDDITIIQQAEKVIFPGVGSAKHAMKNIKAKNLEAALQGLTQPVLGFCLGMQLMTESSTEGKKSSTEGNNDDNTSHIVPCLNLIPTNVEPLKAQGNRLPHMGWNTLTQVSNHPIFKGISEGDYFYFVHSFAAPISEYTIASCEYGSTFSAAIAKDNFIGCQFHPERSSALGSKIIQNFLELDSTELNQELVNL

>sp_Q48A22

MAKGQSLQDPFLNALRRDRIPVAIYLVNGIKLQGQVESFDQFVILLKNTVSQMVYKHAISTVVPSRAVNTAPVPGPDDQSSDA

>sp_Q12ZJ5

MIKTVNIYDNPVLGVFATCTEDVAIVPIGTAGKAIDLLAEQLDVKVISTLINGSIVVGSLSKGNSNGFLISRDANVSDLKDVEVPVEVLPDMLTAVGNVILANDTAALVHPEMTDSSIEVISRVLGVDVHRGTIAGLGTVGMAGVVTNRGLLVHPMVTPDELSVLEDVFALPIELGTTNYGSQAVGSGLLANSKGYVAGSNTTGHELGRVEDALFFA

>sp_Q47UP4

MSNYFNTLSLREKLGQLGKCRFMKREEFNDGCNFIKDWNIVIVGCGAQGLNQGLNMRDSGLNISYTLRDAAISEKRQSWQWATENGFTVGSYAELIPQADLVLNLTPDKQHTSAVTAVMPLMKQGATLAYSHGFNIVEEGMQIRSDITVVMVAPKCPGTEVREEYKRGFGVPTLIAVHPENDPQGNGLAIAKAYASATGGDRAGVLESSFIAEVKSDLMGEQTILCGMLQTAAVLGHKQLIAQGMDAAYARKLLQYGLETTTEGLKHGGITNMMDRLSNPAKILAFDMAEELKVILRPLFQKHMDDIIEGRFSATMMADWANDDVNLLTWRAETAETSFEKSPECDTEISEQEYYDKGIFVVAMIKAGVELAFDAMVDSGIINASAYYESLHETPLIANCIARNMLHEMNVVISDTAEYGNYLFTHAAVPLLADYTASLTLEQLGEGLKESSNNVDNARLIEVNEAIRSHGVEVIGKELRGYMTDMKKIASAK

>sp_Q12WC8

MIRNIKVEHLHETPIEKQETELVERKGVGHPDSISDGLAEAVSRALCKEYIDKCGAILHHNTDETQIVAGRSRPEFGGGEVLKPIYTLLVGRATMEFDGMEIPAETVALQAAREYVRNTIPAMDLERDMIIDCKLGTGSSDLRDVFTRDHVPMANDTSFGVGHAPFSELEQVVYNTERQLLTDLKKKKIPGIGEDIKVMGLRENNDISLTICCGMVGRHIDDMDHYINAKEEMTEYVLDLATKYTDRTVSARINAADKVDGGCDCVFLTVTGTSAEMGDDGSVGRGNRSNGLITPSRPMSMEATSGKNPINHIGKIYNLLSTQMARDVVSAVDEVSDVHIKLLSQIGMPIDQPLVASAQVIPEDGANFAHIQSEAVVVIDDWLENITKITDMVVKGELDTF

>sp_Q47Y99

MTIKSVSVKGIDVANEQPFVLFGGMNVLESRDLAMKIAEHYVEVTQKLGIPYVFKASFDKANRSSVNSYRGPGLDEGLKIFEEIKSTFNVPIITDVHESYQAQPVSEVVDVIQLPAFLARQTDLVVAMAKTGAVINVKKPQFLAAHEMKHIITKFGEAGNENIILCERGSCYGYNNLVVDMLAMDEMKNYAPVIFDATHALQKPGGRSDSADGRRAQAAQLARSGMAIGIAGLFIEAHPDPSAAKCDGPCALPLDKLEPYLAQMKALDDLVKGFTPLITD

>sp_Q47UB3

MIVPGNLFILSAPSGAGKSSLINALLKPDNQASARAMQVSISHTTRDARPGENNGEHYHFVSVEEFKKQISLNAFYEYAEVFGNYYGTSEAAIDAQLSQGIDVFLDIDWQGAQQVRMKKPGVTTIFISPPSKEELESRLRGRGQDSDEVIASRMAQAQAECSHYNEFDYVIVNDDFEQALLDLTTIVNNQRLKCRQQSIAQQSLFSKLLNIEAVEPTE

>sp_Q483R5

MAQLYFYYSSMNAGKSTHLLQSSYNYQERGLVTAIYTAKIDDRFAKGKVASRLGIDADAFLFDEKINLFKDVDQKHQAEKIDCVLIDEAQFLSTEQVKQLTDIVDLLHIPVLAYGIRTDFLGQTFSGSAALLAWADKLVELKTICHCGRKANFVIRQDANGKAVQNGEQVEVGGNERYEPLCRAHFKQLVWL

>sp_Q12VU4

MEPLMGMGVLALMGAAATIAGTTEDLESDVGSQSNPNSQVQLAPQMMYPHRIYNKAISGEPPSNALICAIGGTVASVLMTANLSVIFAIAIGALVASAVHGTYCITAYMGRTASQKRFRQPIYLDILRSHTPVMMGYAFITTFCILVVSYIMVAVLAHPFPLTLLAFIWGITVGAIGSSTGDVHYGAEREFQNVEFGSGLNAANSGNIVRKAESGLRNGIDNSWFCAKFGGPVTGLAFGMTVFLSGWVTAVFNPAISLTMGWLSVAAGVILVLLLIIWNRKIEVAARKAFGPYKEEEEVAA

>sp_Q12VF9

MKGKLITLEGIDGSGKSTITRFLNSHPAFANAVFTKEPTTSWIGDAVYKAIQSDTDELAELMLFTADHADHISTLIRPAIEEGKIVISDRYSDSRYAYQGVTLKERMEEPMEWIQMIHRGWTIIPDLTLLFDIDPAVAVQRCGKRGEQTKFEKTDLLKGVRENYLKLAEKEPERFVVIDTDRDLKEIEKDVLQAITSIIES

>sp_Q47UY4

MILLIDIGNSRTKYVQLISGELSATTQLNNSEFSAEYFTKYFNQASQLIVANVAKSALTDELATWCAREKISYKQVHSEQKKNTLISAYQEPTTLGIDRWLALLGTIHLYPQENVLIIDAGTATTVDLLTSNGQHQGGWILAGINALFTSILSHSTLVHAKSKTMPSLAFGANTSDNVNNACWAATLGMIERAIEQAQQLGDINRIILTGGDGKALTRLLLAQTTENILAVENIQFIDNLIFFGLQEYA

>sp_Q487H6

MGNQKKIIHIDMDCFYAAIEMRDFPEYQNIPLAVGGDGPRSVLCTSNYQARQFGVRSAMPAIKAKQLCPHLKIVHGRMDVYKETSKNIREIFSRYTDLIEPLSLDEAYLDVTDATMCQGSATLIAERIRADIFNELNLTASAGIAPNKFLAKIASDENKPNGQCVITPDKVANFVEQLSLKKIPGIGPKTFEKLNRHGYVTCADVRQSNIRALQNIVGKFANSLYLKSHGVDNRDLEVSRQRKSLAIETTLAHDISTQDECKLVIDSLYQKLLTRLAPHSNREIIRQGVKLKFTDFNQTTVETQSNECQQALFISLLSKAYSRSNKRGVRLVGLTLGFADSPGESQQLSLSL

>sp_Q483W3

MKTLTTDVAIIGAGPVGLFQIFELGLQGLSTVVIDSLPEIGGQCSELYPDKPIYDIPALPNAKASEVIDNLWQQAAIFDPTFLLAERVEHIEKVSEHSFIVTTHKQTQIHCRAVVIAAGNGAFSPVKLKLPLIDKFEDTQLFYRISNIEHFRDKNVVVLGGGDSALDWSLTLQKTAKSVLLIHRSSNFKAAKSSVNKMYELCEQLKMQFLCGQVSSFQEKENKLTGLTITSKDGVNRRVELDELVVLFGMSPKLGPIDNWQLEMHQHQIKVDTQSFQTSVTGIYAVGDINYYPGKRKLILSGFHEAALAAFSIAETVLEKDRIPTLYTTTSPVVHQRLGVEHSLEAMLS

>sp_Q487G4

MTINRREFIKANAIAAAAAVAGVSVPAVASNLITSSDITKLKWDKAACRFCGTGCSVNVGVMDGKVVATHGDIKSPVNKGLSCVKGYFLSKIMYGKDRLTTPLLRMTNGKYDKEGEFTPISWDQAFNVMAEKANDALKKDGPEALGMFGSGQWTVQEGYAAVKLMKAGFRTNNIDPNARHCMASAVGGFMRTFGIDEPMGCYDDLEAADAFVLWGSNMAEMHPILWTRLTDRRLSAPHVKVAVLSTFEHRSFDLADNGMIFTPQTDLAILNYIANYIIQTGRVNKDFVSKHTNFRLGETDIGYGLRPEHPLEQKAKNNGKTKGSSTPIDFDEYAKFVSTYTVESVSKLSGVPEHKLKELAEMYADPKIKVTSFWTMGFNQHTRGVWANNLVYNIHLLTGKISTPGNSPFSLTGQPSACGTAREVGTFSHRLPADMVVKNPKHRAIAEKIWDLPEGTIPAKPGYHAVLQNRMLKDGKLNFYWVQCNNNMQAAANINEEAYPGYRNPKNFIVVSDPYPTVTAQAADLILPTAMWVEKEGMYGNAERRTQSWYQMVEAPEGAKSDMWQLVEFSKRFKVEDVWPEELIAKKPEVRGKTLFDVLYKDASVGKFPLSEIPEDRLNDESRDFGFYIQKGLFEEYASFGRGHAHDLAPYDRYHKERGLRWPVVNGKETKWRFKEGSDPYCKPGSDWDFYGKPDGRAVIFALPYEPAAESPDEEYDLWLSTGRVLEHWHSGSMTQRVPELYKAMPDALVYMHPDDAKKRNMRRGDLVKLISRRGEVQTRVETRGRNKPPVGLVYMPWFDASRLVNKVTLDATDPLSKETDYKKCAIKIVKV

>sp_Q12YR6

MRIGVIAIQGDVSEHVESLERALAERGATAEIVNIKHKGIVPTCDGLVFPGGESTTLGRLILREGIDAEIKDAKEQGIPIMGTCAGLILTAKAGDSQVEKTHQHLLGLMDIKVNRNAFGRQFQSFEVGLEISFLDSPYNAVFIRAPAITEAGEGVKVLASIDGKIVAAEQDNVLAFAFHPELTDDMRVHQYFLDKLFN

>sp_Q12W71

MRVTKRDLKHNREGEISLTPETLDDLWHLKYIIEKGDLVFSLTKRKADNAADKLRPEKAEKKTVRLGVRVEDVEFHKFSNRLRVHGLIEHGMDAGFYHTLNIEDGTNLSITKYWKKDQLERVNEAEAASKRPKVILVAIEEGDADIGFVRHYGIEIYSHITQSSGKGEGTLREVFFSTILDQLTHAMSGTESVVVSGPGFTKDDFMKYASSKNSDLVAGILVEDTSSIGMSGFQEVLRRGAVDRIMEESRIARESSLMDSLLKEIALDGKVAYGMDEVKQAIDFGAVETLLVADEMLRLERESGNIDGLIQNVERSQGKMVVFSTEFEPGQRLHSLGGIAAILRFKV

>sp_Q480P3

MSDTSYKLGITKTFPCNYLPDQQERLLIAVDERLHNSESYGWLMTQGFRRSGDQSYRPSCPNCNACQSIRVLTSDFLPSKSQKRSKKRNSHFIIKQSSQLKDSYYPLFENYINTLHQDGSMYPASFQQFESFLSCNLTQQLFIETWAPANEENGPKEDKLVCVAVTDVLSNGLSAVYTFYHPDYKANGLGVFSILTQLSLSQQMSLPYLYLGYQIDECQKMNYKDRYFPFERFIDGQWLINTKPSVNKAKIAK

>sp_Q12X42

MLPAYLPNPFAALFGGGRPIDNGKTMSDGRRILGDGKTYRGFFVGLIFGALAGLMQMQLLEKYPVLFGVELPTFGTGGSNTTILIFALAVGSLFGDMFMSFFKRRMGLKRGAPLPVIDQLDFVLGALIFAYLASPVWFAEQFTFKVIAVILIITPLLHLATNVVGYFIGVKKEPW

>sp_Q483R0

MARAIIIVIDSLGIGYSPDAVDFGDVGANTFANLARAYYEETGKEIFLPNLSALGMIKACEQASNQTFPYQGQEPSKGAYGFAQEISTGKDTPSGHWEMAGVPVLFDWGYFTDKNSSFPTSLIDDINRETGFDGILGNCHASGTEILTRLGQEHIETGLPICYTSADSVFQIAAHEEHFGLDNLYKYCETVRELLGDLNIGRVIARPFVGDSADNFARTGNRRDYSVLPPAPTVLDKISQEGTHVISVGKIADIFAHQGIDEKTKATGLNALFDATLDHINTAQDNSLIFTNLVNFDQDFGHRRDAIGYAKELEALDVRIPELFHAMSAEDVLFLTADHGCDPTWPGTEHTREYVPIIAYHHQIDSVNLGNRKTFADLGQSVAELFNVEAMDYGTSFLSEIYSK

>sp_Q485G4

MSKRQLCILGSTGSIGCSTLDVVRLHPERFQVISLAAYTSVDVIFEQCIEFKPQQVVLVSSEHAALLTQKLNDANVSNITVLSGEQALIDIAECQNSDTVMASIVGASGLLPTLAAVNAGKRVLLANKEALVTSGAIFMAAVKASGAELLPIDSEHNAIFQCLPSQQQAEIGECQLLANGISKILLTGSGGPFRTRAIDTLESVTPSQACAHPNWDMGRKISVDSATMMNKGLEFIEAKWLFNVEAEDIQVVLHPQSTIHSMVQYKDGSVIAQMGNPDMRTPIAHALSFPERIESGVAPLDFFNTPSFEFQPVDFERYPNLELAIEACKQGQAACTALNAANEIAVAAFLDEKIKFTDIYKINETSVKKFVSQKVDNINEVIALDEQARSFAQTLLADFLQTEALSQKGNK

>sp_P47796

MGHEVDSVLPGLFRRTYGCGRPAISPVITGYSRIVNGEEAVPHSWSWQVSLQDQTGFHFCGGSLINENWVVTAAHCNVKNYHRVVLGEHDRSSNSEGVQVMTVGQVFKHPRYNGFTINNDILLVKLATPATLNMRVSPVCLAETDDVFEGGMKCVTSGWGLTRYNAADTPALLQQAALPLLTNEQCKKFWGNKISDLMICAGAAGASSCMGDSGGPLVCQKAGSWTLVGIVSWGSGTCTPTMPGVYARVTELRAWVDQTIAAN

>sp_Q47VR1

MVKALDRSLALLKQEKIAVLYGGNSAEREVSLNSGQAIAKGLEDAGFNVVLIDTKVVPLTDLVSNKIDRVFIALHGRGGEDGCLQGALEYLDIPYTGSNVLGSSLSMDKVRSKQIFKACGIPTAPFTVVNKAEFSTLSLENILADLGGRVMVKPANEGSSIGMAQAKTIAQLRNALIEAFGFDTQVLLEAWIDGPEYTVTILGDKALPAIHMETPREFYDYEAKYQSTSTQYHCPCGLNERDENEIKSLSMKAFNATGAQGWGRVDIMRNKTGEWQILEVNTVPGMTETSLVPKAAKVFGLNFSELVTQILQLSVKE

>sp_Q47ZB6

MTIKRLTTSTGERIAIVAGLRTPFAKQATAFHGVPAVDLGKIVVNELLQKHDVDPGIIDQLVFGQVVQMPEAPNIAREIVLGTGMNTRTDAYSVSRACATSFQSTVNVAESIMAGHVDVGIAGGADSSSVAPIGVSKKLARTLVDLTKARSLGQRLSLLSRLGLKDLLPVSPAVAEYSTGISMGQTAEQMAKTYQISRQDQDALAHRSHTLATKSWQEGKLAGEVMTVHAEPYKSFIDRDNCFRENSVLESYAKLKPVFDRKHGTVTAATSTPLTDGGAAILLMREGRAKELGYKPLGYIRSFGFAAIDVWQDMLMGPSYATPIALQRAGMNLADLDLIEMHEAFAAQALANMKMFGSTKFAQEQLGRDKAIGDIDMDKFNVMGGSLAYGHPFAATGARLITQTLNELNRRGGGVGLTTACAAGGLGAAMIVETD

>sp_Q488Q1

MVIKPKIRGFICTNAHPVGCEAHVNEQIAYVKAQTSATSGPKNVLVIGASTGYGLASRITSTFGHDAKTLGIFFEKPPTEKKTGSAGWYNTAAFVKAADEAGIYAKNINGDAFSHEIKAKAIEAIKADMGTVDLVVYSLASPRRTDPDTGEVYSSTLKPIGQSVTTKNLNTSKRVIDEVSVEAANDAEIQGTIDVMGGADWELWMNALGEAGVLAEGVKTVAYTYIGKELTWPIYGKATIGKAKEDLDRAATAINAATADLNGQARVTSLNAVVTQASSAIPIMPLYISAMFKVMKADGTYEGCIEQIANLFKENIYSDSPRLDEEGRFRQNYKELEDSVQKRVTDIWNSVDTDTIDELTDYVAYHQEFLKLFGFGIDGVDYDADVSPEVAINNLT

>sp_Q486Y7

MNYFQLFNIEVSFDVDLQQLSSSYQTLQKTVHPDKFAHASEQEQRIAVQKSAQINDAYQTLKNPLQRAEYILVQRSVEMPNEQHSFQDTSFLMRQMELREMLEDVRHSGDVDAALLEVQSVLSTEYLQLSQVMRTQISENNAASNSAACDNLRKLKFYQKLNIEVDRLEDSLFDD

>sp_Q47XX8

MTKFTSLLLVIFTTVLLSACDKENDSSSLSRILARGYINVGTIFGPTNYYTTANGFAGFEYELAKQYADSLNVELRIVPTYSLDELFIKLNTGEVDLLASGLSITDKRLQRFRFAPSYETISQKLVFKQGNVRPRKVADLTGTLMVTSGSSYVENLEKLKQTNSELAWQESTEFDSEELLRKVLSGEIDYTIIDSNNLAINRRYYPEISIGFSINEPEPLAWMVSENSHDDILASLVEFFGTVHHDGTLLALDDKYYGHIEQFNYVETRTFIKAVAGTLPEYQPLFEKYAQELDWRLLAAISYQESHWNPTARSYTGVRGMMMLTLATAKQMGIKSRLDTEQSIQGGAKYFKRMIAMMPDRIPTPDRIWFALASYNIGFGHLNDARIITQRQGGDPDRWVEVKSRLPLLQQKKYYKNTKHGYARGEEPVQYVDNIRRYYDTLSWLDEKAKEQAIALQLAQEKSQALLLNDIVNDSVNESTSEENATESILKEEEVTAETAQ

>sp_Q47VR0

MIKQTHVSNSSNNSTNSTAAQVPEMRRVKRIHFVGIGGAGMGGIAEVLLNEGYQISGSDIGENQVVKRLRALGATIVIGHQAENVVQASVIVVSTAINSENPELVKAKELRIPVVRRAEMLAELMRFRHGIAIAGTHGKTTTTSLIASIFAQGKLDPTFVIGGLLNSAGTNARLGSSRYLVAEADESDASFLHLQPMVSVITNIDADHMETYQGDFEKLKDTYIEFLHNLPFYGLAVVCIDNPVVRELLPRISRQVITYGFSKDADVRAVNYQQNGAVSHFTVEVEGQEPLEMSVNLPGQHNVLNALAGVAVAKDEGVNDEAICKALTEFEGIGRRFEKLTDFSTAAGDMVLVDDYGHHPSEVKATILAMRQGWPDKRLVMVFQPHRYSRTRDLYEDFVEVLSEVDCLFLLDVYAAGETPISSADSKSLARSIRLRGQIEPVYVSDVDKLPQLLATQLQDNDMVITQGAGSIGAVARNLADHSLLHTRCSVQPDAQSEVKGDK

>sp_Q487K8

MQVIIFDNAQQVAENAAEWVAELINKKSNPVLGLATGSTPISLYQELVNKYKAGELSFSNTTSFNLDEYLGINEKNQQSYRHFMNENLFNHVDINKLKTFLPTCNQGENPREQGLDYEDKIAQAGGIDLQILGIGANGHIGFNEPTSSLASRTRIKTLTQQTLNDNSRLFAADEFQPTLAMTMGIATILDARYVLLMATGKSKAKAVKEMVTGPLSAVCPASSLQLHENAIVLLDKEAASELEDHEYYVWADKQNVKINQEFGLYHNY

>sp_Q47WN0

MTTATKDLSSHTPMMRQYLTIKAEFPHTLIFYRMGDFYELFFDDAKKASDLLDISLTARGKTGGNAIPMAGVPYHAAENYLAKLVALGESVAICEQIGDPATSKGPVERKVVRVITPGTVSDEALLVDRQDNLIVAIVDNQSPNAKLKTSSAPAFGLAYLDMASGRFVLTEPQTAEQLQAELQRLSPAELLYSESLQDFSLIEQRKGLRRRPEWEFDLDTAISLLNKQFDTKELTGFGVDDKPLGLAAAGCLFQYVKDTQRTALPHIRAIVCESANKGVVLDAATRRNLELTQNLHGGLDNTLAAILDKSSTPMGSRLLKRWLHFPLRDLTVLNNRQNTVSDIIALDLIAPIQPLLKGLGDIERIVSRIALGSARPRDFARLRHALQQLPELQNELKSGLTESPTNYLATIAQQSQPMPQLEGLLVHAIVENPPVLIRDGGVIAPGYNNELDVLRDLSDGATEFLAQLEQREKERTGIHSLKVGYNRVHGFFIEMSRTAAVDVPDDYIRRQTLKNNERFITEELKQHEEKVLSAQSKFLALEKSLYQELFDKVLPDLAQLQQLSQAIAELDVLTTFAERALALNYVKPSLVEEPGISIDAGRHVVVEQMTNDAFIANPVLLTEQRKMLIITGPNMGGKSTYMRQTALIVLLAHIGCYVPADNATIGLVDRIFTRIGASDDLASGRSTFMVEMTETANILHNATDKSLVLLDEIGRGTSTYDGLSLAWACAEMLALKTKAFTLFATHYFELTLLAGQISTLANVHLDAMEHDDNIVFMHAVQEGAASKSFGLQVAQLAGVPKTVIKRAKQRLSELEQQQTPSILPAPIQNDAFEQLSFAPEEHSVVTTLIDTDINELSPRQALDLLFSLKEQL

>sp_Q485K0

MAYKVLVVDDSSFFRRRVTDILNKDPKLNVIDVAVNGQEAVDKALLLKPDVITMDIEMPILNGIEAVRKIMAQSPTSILMFSSLTHQGAKATLEALDAGALDFLPKKFSEIAKNSDEAGSLLRQRVVEIARKSEFSKQRTRTRPAAVNISPKSSRLIADQHRPLASTSSLVTKDKKVMAAITRSSGKEYKLLAIGTSTGGPVALQKILVQLEENFPLPIIIVQHMPAAFTAAFASRLNSLCKISIKEAADGDVLKPGCAYLAPGGRQMLISGSENSAKIKILDDDSPKITFKPSVDISFGSAAKTFAGKVLGVILTGMGSDGKEGARMLKAKGATIWSQDEQSCVVYGMPQAIDKAGISELSLSLDSMAASMVKEISRG

>sp_Q9S1B7

MSDSNTGTVKWFNEDKGFGFLTQDNGGADVFVHFRAIASEGFKTLDEGQKVTFEVEQGPKGLQASNVIAL

>sp_Q47XI6

MGKIIGIDLGTTNSCVAVLDGDSVRVIENAEGDRTTPSIIGYTAEGETLVGQPAKRQSVTNPENTLYAIKRLIGRRFEDKETQRDIDIMPFGIVKADNGDAWVQVKGEKIAPPQVSAEVLKKMKKTAEDFLGETVTEAVITVPAYFNDSQRQATKDAGRIAGLEVKRIINEPTAAALAYGMDKQEGDKVVAVYDLGGGTFDISIIEIDEMDGEHTFEVLATNGDTHLGGEDFDNRLINYLVAEFKKDQGMDLTSDPLAMQRLKEAAEKAKCELSSAQQTDVNLPYITADGSGPKHMNIKVTRAKLESLVEDMVKATLEPLKQALKDADLSVSKIDDVILVGGQSRMPLVQKTVTDFFGKEPRKDVNPDEAVASGAAIQAGVLSGDVTDVLLLDVTPLSLGIETMGGVMTKVIDKNTTIPTKQSQTFSTADDNQAAVTVHVCQGERKQASANKSLGQFNLEGIEPAQRGTPQIEVTFDIDADGILHVTAKDKNTGKEQKITIKASSGLSDEEVEQMVRDAEANADADAKFEELVTARNQADGMIHATRKQVEEAGEELPSEDKEKIEAALTELEEAVKGDDKEVIEAKTQALMEASAKLMEIAQAKEQAQSAPEGAQEADAAPADDVVDAEFEEVKDDK

>sp_Q12WT3

MADKPHMNLAVIGHVDHGKSTFVGRLMFETGAVPAHLIEKYKAEAKEKGKESFAFAWVMDTLKEERERGVTIDISHKRFDTDKYYFTVVDCPGHRDFVKNMITGASQADAAVLVVAAPDGVMAQTKEHVFLSRTLGINQLIVAINKMDAAKYSEDRYNEVKKEVSQLLGMVGFKADDVPFIPTSAFEGDNITKSSANTPWYTGPALLECLNNLTVPSKPDTLPLRIPVQDAYTISGIGTVPVGRVETGIMKKGQKVTFMPSGATGEVKSIEMHHEEWDQAVPGDNIGWNVRGIGKNDVRRGDVCGPADKPPSVADEFTGQIVVLQHPSAITVGYTPVFHCHTAQTACTLMAINKKLDPKSGQVKEENPTYIKAGDAAIVTIKPTRPMCIEPVSEIPQLGRFAIRDMGMTIAAGMCMSVTQKK

>sp_Q486J8

MTNKTVLHAKHLASGAKMVDFFGWDMPINYGSQIEEHHAVRTDAGMFDVSHMTIVDVQGADAKAFLRRLVINDVAKLATPGKALYTGMLNEEGGVIDDLIIYFFSDTDYRLVVNSATRVKDLAWMTKQSTGFDITITERPEFGMLAVQGPEAKAKVAKLLTAEQIEAVEGMKPFFGVQVGDLFIATTGYTGEDGYEIIVPNNSAEDFWQKLLDEGVVPCGLGARDTLRLEAGMNLYGLDMDETVSPLAANMAWTISWEPTDRDFIGRDVLTAQKAAGDQPKLVGLVLEAKGVLRSHQVVVTEFGNGEITSGTFSPTLGHSVALARVPRSVKVGDTIEVEMRKKLIKVQVTKPSFVRNGKKVF

>sp_Q483B5

MRLIEKVWFNDHPAKWLLVPMLLPLSALFWLISTLRRLSYKIGLSRSCQLSKPVIVVGNIGVGGNGKTPIVLYLVELTRLLGLTPGVISRGYGGKAPHYPYLLDEKSTSIEAGDEPILIQQRCQVPIAVGSDRIASAKLLIAQGCDIIISDDGLQHYRLARDLELVVVDGKRLFGNGLLLPAGPLREGLWRLPKSDLVIYNGKNDQDYQEKNYPCMHMTLAATELCNLLTGERIYLTDFIRLNDSVNAIAGIGAPQRFFDTLKEHQFKVINQQSFVDHHAFVLADFNEFDDNIPLLMTEKDAVKCHDFCKENWWYLPVDATFSDADRQLIIDRTQIAVQSVIQ

>sp_Q12Z94

MERTYVMVKPDGVQRCLVGEIVSRIEKKGLKIAALRMNVMTEAAAKEHYKEHSERPFFGSLVSFVTSGPSVSMVIEGNNAIKIMRAINGATNPVDALPGTIRGDLAVDMGRNVVHASDAPESAEREIGLHFEESEISGYPRADDEWLYE

>sp_P83235

DIEDFYTSETCPYKNDSQLAWDTCSGGTGNCGTVCCGQCFSFPVSQSCAGMADSNDCPNA

>sp_Q56734

MSQEFNVVVLGASGAVGQTMIEILEERNFPVAKLFPLASSRSAGGTVSFNGKQVEILDVDDFDWSQAQIGFFSAGGDVSEKWAPIAAENGCVVIDNTSQFRYDNDVPLVIPEVNPEAIADFRNRNIIANPNCSTIQMLVALKPIYDAFGISRINVATYQSVSGSGKEAITELAGQCSKLLQGLPAESKVYPKQIAFNVLPQIDKFMENGYTKEEMKMVWETQKIFGDDNIVVNPTAVRVPVFYGHSEAIHLETIQPAEAEDVKAVLREAPGIELFESNEEYPTAVTESAGTDPVYVGRVRKDISHSHGINLWVVSDNIRKGAALNSVQIAEVLIRDYY

>sp_Q12X59

MDVTHDMNGQKKWMQLPRNVVIGNGVINEVRDVCTDLKLIDNALVVTGKSTKGIAGEIVQDSLQDAGQNVELVISESASMKEVERIRKHAIESGTKYFLGVGSGKTIDVAKLAATDLEVPFISVPTAASHDGIVSSRASIKDGKTTTSVQANAPMAVIADTEIIANAPYRLLAAGCGDIISNCTAVLDWQLASRLQNVQFSEYAAALASMTAQILIDSADSIKPELESSVRMVVKALVSSGVAMSIAGSSRPASGSEHMFSHALDRVADEPALHGEQCGVGTILMMYLHGGDWKKISDALKLIGAPTTAKELGIEDKYILEALVLSHTIRPERYTILGTGLTPDAAEIVARKTKVIS

>sp_Q12V26

MPDTDNTKIIDGRAIAKKVEAEVKADVERLVREKGITPGLSAILVGEDPASKMYVRLKHKACGRVGIFAEDQHLPEDITQEELLEAISSLNARKDIHGILLQLPLPKHLNEQEAMNAIDPAKDADGFHPFNMGQLLIGVEELVPCTPKGIIRALEEYGVEIQGKHAVIVGHSNVVGKPMAAMLVNRNATVSICHVFTKDVTKFTRDADILVVATGVKHLIKEDMVKEGSVIFDVGITEENGKVYGDVDFENVIKKASRVTPVPGGVGPVTIATLMQHVLMSAQKTA

>sp_Q485G9

MAITAAMVKELRERTAAGMMDCKNALVEAEGDMELAIENMRKNGQAKAAKKAGNIAAEGAILIKTTDGLAALVEVNCQTDFVAKDDNFLGFANEVADAALASKVTIAELQAQFEEKRITLVTKIGENINIRRVEYVEGANLASYSHGATIGVVVAGEGDAESLKHIAMHVAASKPEFLTPDDVPADVVANEKRIQIEMAMNEGKPQEIAEKMVTGRMKKFTGEVSLTGQAFIMEPKKTVGVILTEKGITVSNFVRLEVGEGIEKKEEDFAAEVEAQIAAAKA

>sp_Q47Z14

MPQQNSYSKEDLVKAGTGELFGEGNSQLPSDNMLMMDRIITISEEGGENGKGFILAELDITPDLWFFDCHFKGDPVMPGCLGLDAMWQLVGFFLAWTGGPGKGRALGVGEVKFTGQILPTAKKVTFKIDFKRVIKRKLYMGLADGSVSVDGREIYTAKDLKVGLFTDTSKF

>sp_Q47WW8

MRTNIVIPMKDPQLSKTRLDPQLSSRQRQALALSMFKTTLSFFNKYFPQHHLLVVTASEFISDIACTYGASVLIETKLGLRQAVESAARWSLNNDFQSQLLIPADIAELDYREFERLLMIYRPVPSVLLCPAFDLGTNALLTTPPNAIPFLYGIDSSLAHQRVAQERDIVCQVIKLPALALDIDTPDDLELLALLSSPVTQELNKLCKTA

>sp_Q9K4Z2

MQLPQFSELYKSLILIPSISSLEKELDISNKPVIDLLSGWFSELGFSINITSVPETNGKFNLVATYGQGDGGLLLAGHTDTVPFDDDLWTKDPFKLTEKDDKWYGLGTIDMKGFFAFVLEACKNIDLTKLDKPLRILATADEETTMAGARAIAAAQSFRPDYAVIGEPTGMVPVFMHKGHMSEAIRITGRSGHSSDPANGINAIEIMHQVTGQLLQLQRKLKEQYACDHFVIPQPTLNFGHVHGGDSPNRICGSCELHIDMRPIPGVNPDELFMLLNQALLPIIKQWPGAVDVYHLHEPIPAYACNTDSALIKLAEKLTGEAVIPVNYCTEAPFIQQLGCDTIVMGPGSINQAHQPDEYLDLSAIKPTQAIIQKLIEETCKN

>sp_Q93GT8

MAAKDVLFGNDARVKMLRGVNILADAVKVTLGPKGRNVVIDKSFGGPIITKDGVTVAKEIELEDKFENMGAQMVKEVASKANDEAGDGTTTATVLAQAIVNEGLKSIAAGMNPMDLKRGIDKAVIAAVEALKESSTPVTDNKAIEQVGTISANSDETVGKIIATAMEKVGTEGVITVEEGQALTDELDVVEGMQFDRGYLSPYFINKQENGTVELENPFILLVDKKISNIRELLTTLEGVAKAGKPLLIIAEDVEGEALATLVVNNMRGIVKVAAVKAPGFGDRRKAMLQDVATLTAGTVISEEIGMELEKVTLEDLGQAKRVVISKDTTIIIDGIGVEADIQARVSQIRGQIEDSSSDYDKEKLQERLAKLAGGVAVIKIGAATEMEMKEKKSRVEDALHATRAAVEEGVVAGGGVALIRAADAIKDLEGANEDQTHGINVAIRAMEAPLRQIVANCGDEPSVVLNEVRNGKGNYGYNAGNSTYGDMIEMGILDPTKVTRSALQFAASVAGLMLTTEAMITDAPVKDAGGMPDMSGMGGGMGGMGGMM

>sp_Q485H4

MTSHPVSVHIVPSHFINQLAISADFLSTRDICQLSQSFNTWLKDVFIENDINDLLSARAVFVDAILKKLWCQHHLDEFQITLIAVGGYGRGELHPQSDVDILLLTQEEVDLELEEKISSFITQLWDIKLDIGHSVRSIKECLKQAVKEVTVATNLMEMRQVAGNETLTQQLTPLLSEDVFWTSEKFFIAKCKEQEARHQQYRGAAYTLEPNLKANPGGLRDIQTIAWVAKRHFSADSLEELVEHDYLYPNEFFELLESQDYLWRMRFALHFVAGRSENRLLFDYQADVAKMMGFGDEGKAPVERMMKRFFRIIARVTELNTMLLQHFEQAIIKKPELSNISIINQDFELVDKLINTRNDRIFMRPVKMIEMFLIIAQEPGIKGIHSHTMRLMRNARRRLISGLIDYAECRRMFMAIIRHPRGLGLALTLMHRHSILSSYLPLWRNIAGQMQFDLFHAYSVDEHSYRVIKNLHQFSQKEHNHKFPLCSKIVQKIRKPEVLYLAGFFHDIGKGRGGDHAKLGAVDALTFCLSHQLSKHDSNMVAWLVEHHLLMSVTAQRRDINDENVIRTFGEIVRDEAHLNYLYCLTVADMRGTNESLWNNWKANLLEELYFNTLSAFRHGLEKPVEVRSKIRENQQQALALLNENNVDEQSIKALWREFRIDYFLRYSPEQIARQCQNIVEHDREKPLVLISPIPYRGGTEVFIFTKEKNNTFASTVSFLVTKKLSIHDAKIITTKTGYTVNTFVVLDSRNKPLRERFYTKEMSQALVDRLQQVNICELPEPKLARHMKKFKVPLRVNFIKIHAKNRTMIEIIALDRPGLLSNISQVFLEARVNIHSAKITTFGEKADDVFTISTEEDDALTTQEKEALALRLTQEID

>sp_Q47UZ9

MAKSPQQPVIQSILDDAYHKFENDTSGKNADYIPALAKVDSAYFGLAVVTPHGKIYTKGDVSQPFSIQSISKVFTLALAMEQKGPQTIVDKIGVNATGLAFNSVTAIELNKARSVNPLVNAGAIATVSLLDGKNEKAKWSALSAWYDKFANRKLSVLEDVYKSESDTNGHNRAIAELLTSYDRFYGDVDLNLAIYTRQCSVAVTTKDLAVMASVFANNGVHPLTDKRLMSSDNVSRVLAVMTTAGLYENSGQWAYQVGLPAKSGVGGGIIAVSPGKFAVAVFSPRLDSAGNSIRAQKAIDYIAEKLHANIF

>sp_Q47VA3

MSNPELPIKFTDSAASKVLSLITEEENPALKLRVYVTGGGCSGFQYGFTFDEKVNDGDMTIEKQGVMMVIDPMSLQYLVDGEVDYLESLEGSRFVVNNPNATTTCGCGSSFSI

>sp_Q48AA9

MIDSTTELRPLTKRQQQIYDLIKAKIQDTGMPPTRAEIANFFGFKSANAAEEHLKALAKKGYIEMLAGTSRGIRLVEEMLEAEGLPLIGRVAAGEPILAQEHIEEHYKMDGNLFHPAADYLLRVNGESMKDIGILDGDLLAVHQTTEVQNGQVVVARVENDVTVKRFKREGNVVYLHAENEDFSPIKVDLANQEFNIEGIAVGIIRSGRWM

>sp_Q12XX4

MDFYQEYITTIHDFCIDKEQLVKRIEGLKVSRPASLIIPILYKEVENPPLKKIITDLNECTYLSQVVIALAAETTEQYVHVVEYFKDLKLPHIVVWCDGPRIKQIIFDMKKKGIDLTSFKGKGKDVWIATGIATLESYAIAYHDADIVTYSVDLPAKLLYPIVETELNFFFNKGYYARINLDSMTMHGRVFRLFVRPLLDTLQSESNADILRYFLAFRYTLAGEFAMTSDLAMNIRIPADWGLEVGLLAEVYRNATTKKICQIDLGYYDHKHQELGVNRSEGLCKMVSDIFTTFMRVVTESTDNRISESYLHGIHVRYKRLGQDLIRRYHADALCNGLYYNRHEEEIYVDMFARVIRKAGDDYRHDPSDVLMPDWTRALSAVPDLREKLYEACIADVKEYCEKK

>sp_Q12W31

MLEVLQESLRKAPIVIRGEYPYFIHPISDGIPSLEPELLDEIADHMIDIAGKDYDRIVSIEAMGIPLATVLSMKTGKPMSIVRKRQYGLEGEVILSQSTGYSKGELYINGISKGETVLVVDDVISTGGTLKALLPALENMGANICNVIVVISRGDGAAKMSDMGYNVNTLVKIDVDMDGVSILEVAGEQQ

>sp_Q12WQ7

MTTVLGIEGTAWNLSAAIVDEDDVIAEVTETYRPKTGGIHPREAAQHHALHASDVIERLLKEYRDKGHSPENIDAIAFSQGPGLGACLRTVATSARALALSLDIPLVGVNHCIGHVEIGRWKTPAVDPVVLYVSGGNSQVLAHRAGKYRIFGETLDIGIGNALDKFARGAGLTHPGGPKVEEYARKATNYVKMPYVVKGMDFSFSGLSTAATDALKDNSLEDVCYSFQENAFAMLVEVTERALAHTGKSEVLLAGGVGANMRLREMLDLMCEDRGASFYVPERRFMGDNGAMIAYTGLLMFNSGTTLPIENSHVDPSFRPDTVDVTWIADEKEVL

>sp_Q47UY2

MHSNQNYSLKSSNSFNIKASCSRIYFPSSLAELQQLPDLSAGNTSDNFYILGEGSNTLFVEAQAPIIIQPKFNGISIVEQDDHFVVTVGAAENWHDLVCFCLEQGIYGLENLALIPGSVGAAPVQNIGAYGVEFADFCQEIQWYEFASETLHSLTKQACRFAYRDSIFKQERYNKGLITQVTFNFPKAWQANLSYAGLDTLAKESTAKQVMAQVIALRSSKLPDPKELPNAGSFFKNPIVNDADFAQLQQQYPKIPHFPQKNGEIKLAAGWLIDQAGLKGFRHGDVGVHQQQALVLVNYGSELGAEIISLAKYIQQKVAKKFSVSLIPEVRMITHKGERSFSSLSDLNPIENITVIGDSNSIRGSSDD

>sp_Q47VQ7

MTWLTAFKDKNIVVLGAGMTGLSCLRFLHAQDLSFAVNDSRPMPFANRDEQTQYENDYPKAKFVFGQWQQSLISSADIIITSPGIDLVSEGITALIPENCLVIGDVELFCLVNNSRISPMKMLAVTGSNGKSTVVSLLASLAKAIGVNAALAGNIGEPILNLLHRENVYNSQLANQPDIVIVELSSFQLETLSSMHAIAASVLNLSDDHLDRHKTLANYQAIKQSIYPQAKIAVVSREDQASNTLVAAQEIISFGLNKPEQDCFGLQAIDNKMVMMFGEQALISIDELPLAGMHNALNYMAALALGYSAGWSLSAMTENLAGFMGLAHRCQRVASEDYIQWINDSKATNVGATLAAITGLVPTLTGQNKLILIAGGDGKGADFSALTTILNCDVNQLITLGKDGAEIASLVSDAIQVDTLREAVEQAKQIAKPGDMVLLSPACASIDMFKNYQVRGEQFIAAVQAKEDSCR

>sp_Q12UB0

MSGFLLALRTTFGFLSTIPVGMSMEGLDELVKRSYLQTFAGIVLGSMIGIFAYLTESFLPSTISAVLIMVFIYYITGLNHLDGLGDFGDGATAHGSLEKKVNALKDMSLGIGGVSYTVLALIALYASISSLQAEVLFFSDNAALIIAISLLIAEIGAKQAMLTVAAFGKPIHEGLGSMIINNTTFPRYAVSFVLGALVCVLAFGTLGIIGYISAIVTAFVILNISIRHFKGINGDCIGTSNEIARIIVLMVLTVAITAVNNGYGGLFWTPL

>sp_Q48A63

MSSTKIHLTKAAFTISAPDIRRLPADSGIEVAFAGRSNAGKSSALNTLTNQRGLARISKTPGRTQLINVFEVAENRRLIDLPGYGFAQVPLAMKKKWQKALGEYLEKRQCLKGLVVLMDIRHPLKDLDMDLIQWAADSDLPVLALLTKCDKLSQGKRSSEVLAVKKALSSLNADIQVQAFSSLKYTGKEQADAIICQWLEQEAQEYELPEEDDFDDSDEFTEEE

>sp_Q95AG0

MIFKPQSFLKAIVLSMTITFAFNMSAPIASAYPIFAQQNYENPREANGRIVCANCHLAQKPVELEVPQAVLPDTVFEAIIQIPYDTQVKQVLANGKKGDLNVGMVLILPEGFELAPADRIPEEMKKKVGNLYYQPYSPDKKNILVVGPVPGKQYSEMVVPLLSPDPATNKNVSYLKYPIYVGGNRGRGQVYPDGSKSNNTVYNSPVSGTITEILKLEGKKGGYTISITKADGVVLTEKIPGGPEVIVKEGQAIIADQPLTNNPNVGGFGQKDVEVVLQNPVRIQGLLAFFACILLAQILLVVKKKQFEKVQLAEMNF

>sp_Q12TF2

MAEIVASDEMNEYFNTLEGTLKKEIDIVNDARSRGKDPKPHVEIPLAKDLADRVENLIGVKGVAELIRKLEETMSREEAALALGREVAQGKVGEFDSKSEAIEAAIRVSVAMLTEGVVAAPIEGIDRASIGKNDDGSEYVSIFYAGPIRSAGGTAQALSVLVGDYVRRGVGIDRYKPRKEEVERYIEEIMLYKRVASLQYTPSEEEIRLIVENCPICIDGEPTEAEEVEGHRNLERIDTNRVRGGMALVLAEGLALKAPKIQKHVKNLKIDGWEWLEQLISGVKSSSESDEDEETDGKPKIKPKDKYMRDLIAGRPVFSHPSRPGGFRLRYGRSRNTSFAAAGISPAGMIVMDDFIAPGTQLKVERPGKAAGMAPVDSIEGPTVRLNNGDVIRIDTIDEAYALRSEVEEIIDIGEILINYGDFLENNHPLAPSPYCFEWWIQEYRKAAPDTETNEAELKEPTQEVALDLCKELNIPLHPKFTYLWHDIDNSQYTALADLISKDGLLEADGSFLKLPLQRTIDTGMKKVLEDLLVQHKIQGQALTIEEPLPLIHSLGLDEELSKGWDSLGHEELLENINEIAGFVVRPRAPTRIGARMGRPEKSDKRKMTPAPHALFPIAEAGGNTRSLEKAANFKVNTNSKAGTIPVEIGNRICPACGVEGFEFRCECGEYTLPKLFCPRCGISVNKEKCPKCNSKTTCTSMRKIDFKSIYQKAFESIGERDHLDSFKGVKKMMSKHMTPEPLEKGILRAKHGLFTFKDGTVRYDMSDIPLTHIRPAEIGVSCERMIELGYLKDIYGKPLIDSEQVLCLKVQDLVISYDAADYILRITQYIDDLLVKYYKVAPYYNAKHIDDIVGVLLMGLAPHTSAGVLGRLIGFTTASVGYAHPYFHAAKRRNCDGDEDCVMLLMDGLLNFSRDYLPDKRGGQMDAPLVLTTRLDPSEVDKEAHNIDMCASYPLEFYEATQNIANPKDFEGTMDLVSGRLGTTLQYEEFMFTHDTSNIAAGPLKSAYKTLGTMVEKMDAQLELAKKIRAVDAPDVAERVLTSHFLPDMFGNLRAFSRQRTRCVKCAAKFRRPPLTGSCPKCGGRVILTVHEGAVKKYLQVSIKIAEEYNVSSYTKQRIELIGYDMKSLFENDKSKQMGLSDFM

>sp_Q47UU9

MAKEKFERNKPHVNVGTIGHVDHGKTTLTAAISAVLTKVHGGEVKDFAQIDNAPEERERGITINTSHIEYDTEARHYAHVDCPGHADYIKNMITGAAQMDGAILVVAATDGPMPQTREHILLSRQVGVPFIIVFMNKCDVVDDEELLELVEMEVRELLSEYDFPGDDLPVIQGSALGALQGDEAWEAKIIELADALDTYIPEPERAIDGAFIMPIEDVFSISGRGTVVTGRVERGIIKIGEEVEVVGIRDTQKSTCTGVEMFRKLLDEGRAGENCGVLLRGLKREDVERGQVLCAPGSILPHTKFESEVYVLSKDEGGRHTPFFKGYRPQFYFRTTDITGAVELPEGVEMVMPGDNLKFVVELINPVAMDEGLRFAIREGGRTVGAGVVSKIMA

>sp_Q47W65

MQNLHIKRPLEPKQAFQYRGRFAPSPSGLLHFGSLIAALASFLDAKAFVNDHGEQGKWLIRIEDIDRPREQKGASTAILTTLEAFGLHWDETALYQSTQSQYYRDILSNLAQQKLSYYCQCTRSQIKAIGGIYQGHCRTANYKSQGNATRLVNQYGLHQFNDLFQDHVVCNKALANEDFIIHRKDGLFAYQLAVVADDIAQGITHVVRGCDLLEPTARQLTLFQTLNNSFLKCTTPRYGHIPLAITSEGYKLSKQNKAPAINNANPQPALIAALIFLGQKSIPDLVSASVEEIIQWAITHWQRDLVPKAFEINID

>sp_Q47XB6

MSNTQEKILFIDRDGTLVEEPAIDKQLDTLEKLVFEPNVIAELLKLQAKGFKLVMVSNQDGLGTNSFPQADFDLPHNKMMDLFSSQGVHFQDVLLCPHFDEDNCNCRKPKLGLVSEYLQQGRVDFANSFVIGDRETDMGLAANMGIVGIKYDPETLNWAQVSEQIITQLEQPRIATVTRTTKETDITVTVNLDKAGESSIDTGLGFFDHMLDQISTHGGFSLQCHVSGDYHIDEHHSVEDTALALGQALKQALGNKRGINRFGFTIPMDECRAECAIDLSGRPWLEFDADFTSANVGTMSTQMVPHFFRSLADSMLITLHLSTSKGNCHHQVESLFKVFGRALGQAIKVDGDAMPSSKGTL

>sp_Q12UY6

MLTKRIIPCLDVTLDASGGTVVKGVEFVDLKKAGDPVDLAKRYNEQGADELVFLDITASHEGRSTMIDVIERTANEVFIPLTVGGGINSVEDVRQILRAGADKVSVNTAAVKNPEFIREASNIFGSQCIVTAIDCKRNLDVENNQDKTILELEDGTLAWYEVVIYGGREPTGLDTVQWAKKVEELGSGEILLTSMNRDGTYDGFDIPITKKLSEELDIPIIASGGVGNPEHMYKGFVDGKADAGLAASIFHFGEYTVRDVKEALRAKNIPVRL

>sp_Q12WC7

MIRIAIPNKGRLHEPTIQMFKEAGLPVLGGSNRKLFAKTNDPEITFLFARAADIPEYVQDGAADVGITGLDLISETESDVEMLLDLKYGGADLVLAVPEESDISSANDLDGMRVATEFPGITARYFKDLGIKIDVVKVSGACEMTPHVGIADAIVDISSSGTTLVMNHLKVIEKVFSSSIYLIANHETAKTEEKIEHIKTALESVMHAKAKRYLMMNAPITVVDDLKEVLPGLAGPTIMKVESKEDIVAVHAVVDADIIFATITKLKAAGAFDILVMPIERMIP

>sp_Q482T5

MLNPITKKFQLGKHTVTLETGAIARQASAAVMASMDDTCVLVSVVGKKEAKPGQDFFPLTVNYQERAYAAGKIPGSFFKREGRPSEEETLIARLIDRPIRPLFPEGFTNEVQVIITVVSVNPEIAPDIISLIGTSAALAISGLPFSGPVGAARVGYTDGQYILNPLQSELPTSQLDLVVSGTDSAVLMVESEADVLSEEVMLGAVVYGHEQMQVAVSAIKEFKAEVNTPSWDWVAPVKNAELLAKIAELSEAQVNEAYQITEKAVRYEKIKEIRSSVLEALLAENADVDVQEAKDLFHDLEKTVVRGRITDGNPRIDGRDPESIRALDVMTGVLPRTHGSAVFTRGETQALVTATLGTQRDAQRLDTLMGDKTDPFMLHYNFPPYCVGETGFVGSPKRREIGHGRLAKRGMLAVMPSLEEFPYAVRVVSEITESNGSSSMASVCGTSLALMDAGVPIKASVAGIAMGLVKEGEKFVVLSDILGDEDHLGDMDFKVAGTTGGITALQMDIKIEGITQEIMQIALNQAKAARTHILSVMDEAIGGHRDDISEFAPRIHTMKVSQDKIRDIIGKGGATIRQLTEETGTTIEIEDDGTVKIAATSGEQAEDAINRIKALTAEIEVGTLYTGKVVRIVDFGAFVNVLPGKDGLVHISQISEERVNNVSEVLTEGQEVKVKVLEVDRQGRVRLSIKEAMEKPAAEATPAAE

>sp_Q12TH8

MSTSKRKIEHLELCAKRPVESRNVTSGFDDVMLIHKALPQIHMDEIDLSTDFLGKSLKAPFLIASITGGHPDTTPVNAALAEAAEELGVGIGVGSQRAAIEDPEQESSFSVVRDKAPNAFVYGNVGAAQIKEYGIEAIEKLVDMLDADALAVHLNFLQEAIQPEGDRDATGVLEMIKEVCSLNVPIIAKETGAGISKEDAALLKEAGVSAIDVGGVGGTSWSGVEVYRAHDSGDAISEDLGNLYWDFGIPTVSSVLECRSFVPVVATGGVRTGLDIAKSLSLGAYAASAALPFVGPALIGADEVVSSLSKMLNELRVAMFLCGCGNINELRTSSKVTVTGWTKEYITQRGFDPKDLDIRSDL

>sp_Q7X3X5

MKVAVLGAAGGIGQALALLLKTQLPAGSELSLYDIAPVTPGVAVDLSHIPTDVTITGFSGIDPTAALVGADVVLISAGVARKPGMDRSDLFNINAGIIKNLASKCAEVCPTACIGIITNPVNTTVPIAAEVLKQAGVYDKRKLFGITTLDVIRSETFVSALKGISLADVAVPVIGGHSGATILPLLSQVKGVEFTAEEIATLTTRIQNAGTEVVEAKAGGGSATLSMGHAAARFGLSLVRALQGEKGIVECTYVDGGSEHATFFAQPVLLGKNGVEEVLAYGDLSDFETNARDAMLEELKANITLGEEFVAG

>sp_Q12UU7

MKQQVEVKELKEGKYVLADDEPCVIKSIQKSKPGKHGSAKARIEAIGIFDGQKRSIISSVSAKTYVPIVERKSAQVLSISSNIAQLMDMETYTTFELTIPEDYKDRVTEGKDITYIEAMGKMKIDLR

>sp_Q485G0

MTYTLAEIAIKLDAKLIVPAALDEQNEALTQISGLATLAKAGTGQVAFLANSKYQQQLSSTNASAVIVSPDAVEACQVSALVMDNPYMGYAMLASLLDSTPKVSCGIHPNAVIADDVLIGENVSVGANTVIESGVQLADNVSIGAGCFIGHGAKIGESTILWANITIYHRVEIGHHCLIQASTVIGSDGFGYAPVKGQYKWHKIPQLGSVIIGDHVEIGASTTIDRGALDNTEIRDGVILDNQIQIAHNVIVGENTAIAGCTVIAGSTVIGKNCTIAGLVGVNGHITIADNCVFTGMSMVTKNISQAGVYSSGMPVVQNKEWNKTNARVKRLDSLTKRVKELEKLLAKN

>sp_Q483B6

MASSPKTNALSSSDSANATTWQNFKRLVSYAKPYKLGFVAAIIGMLGYAAIDVYFLSQLKPLVDEGLSGANANFMKWAPLFIIVAFTVRGIAHFIANYCLAWVGNNVVADLRQKLFEHIMSMPVAFHDQTSTGSLISKITFDTEQVLNSVSKSILTIVQQSAFIIGLLGLMFYYSWQLSLIFLLITPIIAVIVSVVSKRFRKVSKNIQGAMGEVTTAAEQTFNGHKVVLTFGGQQREFSRFAKINKHNRQQRMKMRATKSASVPIIQVIASFALAFVFYAITSDSLRDSISPGTFVSIITYMTMLLRPLKMLTNVNSEFQQGMAACTSIFSILDHEKEKDNGDKQLERASGTLSFKHVDFSYKNTNTMTTSDKEQDTKLALNDITFDLAPGETLALVGRSGSGKSTASSLLLRFYDATRGEILIDDTNIEQFQLKDLRKQFSYVSQQVVLFNDTLANNIAYGKPEATEAEIIEAAKSAHVMEFAEHMEQGLETNIGENGALLSGGQRQRVAIARALLCDTPFLILDEATSALDTESERHIQDALQTLQQNRTSIVIAHRLSTIENADKIIVMEQGKIVEQGNHQSLLAKQGAYAQLHSFQFE

>sp_Q12V31

MDIIQAKDIIIDFIGTKLEGTGIEGAVVGISGGIDSALVAYLSVEALGAENVLGIHMPEASTPKSEIEDASKVAEALGIDFKVINITNVLEVYRTAMPDIDGASAHVDGNLKARIRMSMLYYYANMFGRVVMGTGNKSEILLGYFTKYGDGGVDIEPIGDLYKTEVREMSKMLGVPESILEKAPSAGLWEGQTDEDDLGVTYETIDKVLQPILAGEGQERVHLKLGVPMEEISSILLRVRSNLHKRTTPQIAYLDDLRGDWLS

>sp_Q47XI3

MAQHYKTIGLIGKPNHDGASATIQTLHKYLLANEYKVIVEVSVAQSLDIKKMKTGTLTDIGEQADLAIVIGGDGYMLGAARVLACFDIGVIGVNRGNLGFLTDLSPSEIIKPLEQILSGKSRSEQRFIIEAEVYRHGKLKSSNSAVNEAVLHAGKVASMIEFEVYIDGTFMFSQRSDGLIISTPTGSTAYSMSAGGPILTPNLNALSLVPMFPHTLTSRPIVVDGNSEIKLILANDNHENLQVSCDGHVILTVMPGDEVIIKKSECSIRLIHPLDHEYFNVLRNKLSWGNKLY

>sp_Q12VC9

MIKLTPAMKQYYDAKKQHSDALIFFRMGDFYESFGEDAKIIAKELEITLTTRGKDIEGEKMPLAGIPYHALDNYLPRLIKKGYKVAICEQLEDPKKAKGIIKRGVVRVVTPGTAIDTSMFTDPSNNYLMSISGGDGDYGVSFLDVSTGEFLTTQFADKSPYDRIASEAARMRPSECIISRTMFSDERLVERLKELNVLVQGFKDEAFDVDSSRKLLERHFNVSTLEGMGCAGLPYATSSAGAALDYALTTQMRELGHVSELSTYSDSEFMMLDSITLRNLEIVKNVRGEGNDTSILKVLDDTNTPMGGRLLQKWLLKPLINVDSIDHRLDALECLANDTMLRFDVRSHLSFVKDIERLIGRVVYGNSNARDLIALKRSLGSVPQIVESMGDDPGCEMLINIRDGLLGFEQLENIVKLIDDAIVDEPPVSVREGGMIRSGYNEKLDELKGMSTGGKTWIASFQQKERDRTGIKSLKVGYNRVFGYYIEITKSNIAQIPDDYIRKQTMRNAERFYTPELKEWEDVILSADEKITALENELFTEITSRIASHASDLQRIAVLIGQLDCTASLAEVAVNNNFVRPNITSDCKILIREGRHPVVEKTVRGGFVPNDTEMDCVDEQFLLITGPNMAGKSTYMRQVSLIVIMAQAGSFVPASHASIGIVDRVFTRVGAFDDLASGQSTFMVEMVELANILNNATPKSLVLLDEIGRGTSTYDGYSIAKAVVEYIHNKGRVGVRSLFATHYHQLTNISSSLKRVKNYHIAVKEDGDDLVFLRKIVPGATDKSYGIHVARLAGVPHKVTQRAKEVLQDIEDESVISKESDSKRGRKKKSAQYTQLMLFDPEGSSAPVAEPDPVVEELKELDVNSMTPIEALNKLSELQKKAGKGGK

>sp_Q480D3

MNTQAKLVNELKNITTEGQNPNTLDIDLLDSLGVLKKINTEDQKVASIVGLLLPEISQGVDLIVDAFACGGRLIYIGAGTSGRLGVLDAVECPPTFSVSSEQVIGILAGGAGAMYKAVEGAEDNRQLAIDDLKAINLSSKDIVVGIAASGRTPYVISGMAFAREQGAKVIGVSCSANSSYAQNCDINICAVVGAEVLTGSTRMKSGTAQKLILNMLSTASMIRSGKSYQNLMIDVNASNKKLYARAVRIVMQATECDFDTAEMALAQANNQTKLASLMVLTGLDSTQAKAALASNKGFLRKAVEQQGC

>sp_Q12TU6

MRKVVLASASPRRKELLSKLIGNNFEVCVSSYEETPLQEMNVEELVVFHSLEKAKDVALRFDSGIIISADTVVFCDGAILGKPHTLNNAKEMLENISGKSVLAITGMTILDMDSGKCVSEYVSTDVNMKQMSSDEIASYVNSGEPLDKAGAFAIQGKGAVLVESINGDFFNVVGLPLFRLGTILEEMGISIFDDC

>sp_Q489G6

MPWIQLRLSANEDNAEKYSDWLSACGSQAVTFIDAKDTPIYEPLPGDEVIYWSNTVVMGLFEASHDMDKVISYLQSIHPDKEQMRYKLEQLEDKDWEREWMDNFHPMKFGERLWVCPSWRDVPDPEAVNVMLDPGLAFGTGTHPTTALCLTWLDGLDLQDKTVVDFGCGSGILSLAALKLGAKKVIGIDIDPQALQASLANAERNNVSDRLELYLPKDQPEFKADVVVANILAGPLRELAPVIIEYVGDKGLLALSGVLEEQAQTLQTIYGQWCDMEPVSVQEEWVRLNGQRK

>sp_Q488N5

MYCPFCSANDTKVIDSRLVSDGHQVRRRRECLACHERYTTFESAELVMPRIIKRDGSREPFNEDKMLSGLTRALEKRPVSMEQIELAVNKLKSQMRATGEREISSEMLGDLIMAQLKELDKVAYLRFASVYLSFEDISEFADEITRLGKEKNGKAKKAKPAKTAK

>sp_Q12WM5

MTEISSMVITHAKATVEEMEDSWHGDIDLVLSQLYSNELVYECAVLKTCNRVEIYVVSSKGSSVLFHYAKEMGVSAKIVEFYDHDESLRHLLRLACGLESMIIGEDQILGQIKDFFLMAKGAGTVGKVLSTAFSKAIQVGKRVRTETFINRGAVSIASAAVDLAEDILDGLNDKHILVIGTGEMGTLVTRALSHRDMHVIYLANRTYEKARDLAEELGGEAVMFDQLEKYVRAADVVISATSAPHYVLKGDLVAKVMEGRENELLLIDIASPRDIDPAVEEIPHVILRNIDGLRVINEKNLQMRMVEAKKAEIIIDDELDMVKAQYKRQKADAIISNLYSQSHGLRHNEMEHAINKLSAYHTIGEIERKVLEDLTHAITNKILAEPTKKLRNAAEYDDDKFLDSVSRLFDIRPIKKNDGITK

>sp_P20619

MIQRNINRVALIGAGSVGSSYAFALLNQSITEELVIIDVNEDKAMGDAMDLNHGKIFAPNPTKTWYGNYDDCKEADIVCICAGANQKPGETRLDLVEKNLKIFKSLVDQVMASGFDGIFLIATNPVDILTYATWKFSGLPKERVIGSGTILDSGRFRFLLGEYFDIAPANVHAHIIGEHGDTELPVWSHADIGGVPVEELITRNPEYKMEDLDQLFVNVRDAAYHIIKKKGATYYGIAMGLARITKAILNNENSVLTVSTYLDGEYGEKDVYIGVPAVVNRTGIREILELTLSETEQKQFTHSSTVLKEILNPHFKEAR

>sp_Q47UN7

MPKLRSATSTQGRNMAGARALWRATGMTDGDFGKPIIAVVNSFTQFVPGHVHLKDMGQLVAGAIEEAGGVAKEFNTIAVDDGIAMGHSGMLYSLPSRDLIADSVEYMVNAHCADAMVCISNCDKITPGMMMAAMRLNIPVIFVSGGPMEAGKTKLSDQIIKLDLVDAMIKGADPTVSDEDSDKIERSACPTCGSCSGMFTANSMNCLAEALGLALPGNGSMLATHADREQLFLKAGKQIVELTKRYYQDNDESALPRNIACKEALHNAMCLDIAMGGSTNTILHLLATAQEAEIDYTMEDMDKLSRIVPQLCKVAPSTPEYHMEDVHRAGGVISILGELSRAGLLKTDVPNVLGTTLADVIAKYDITLTDDEDIKKFYRAGPAGIRTTKAFSQDCRWDTLDDDRVNGCIRNLENAFSLEGGLAVLSGNIAVDGCVVKTAGVSDDNLVFTGPAHIFESQDDAVAGVLDGKVVAGEVVVIRYEGPKGGPGMQEMLYPTTYLKSMGLGKACALLTDGRFSGGTSGLSIGHVSPEAADGGTIALVEQGDIIHIDIPTREITLQVSEEVLEERRAAMISKGKQAWKPADRVRPISYALKNYAMLATSADKGAVRNRDLLDGLVDK

>sp_Q486R1

MEIILANPRGFCAGVDRAISIVERALDLFGAPIYVRHEVVHNKFVVDGLKDRGAIFVDELDEVPDDNTVIFSAHGVSKAVRQEAKTRALKVFDATCPLVTKVHMEVSRVSRKDIECILIGHAGHPEVEGTMGQYSSDSAGIYLVESPEDVASLVVKNPEKLYFCSQTTLSVDDTMDVIAALQAKFPLIEGPRKDDICYATQNRQDAVREIADKVDLMLVVGAKNSSNSNRLKELASKMGVTSYLIDTAENIETDWLTGVNKVGVTAGASAPAVLIKQVVEALKSYGGHQVIEHPGKEESIVFAVPAELR

>sp_Q487E9

MASTLKFIVIVPAAGVGKRMQANCPKQYLRINNETILSHTVMRLLSHPLISQVIVALGTEDQYFAESELAHHKDIIRVNGGTERVNSVLNGLKAVDSDKYPWVLVHDAARPCVSHQDIDKLITRCLRKDYGGILATPVRDTMKRGVLIKDSAKGDNTIIESTVEREQLWHALTPQMYKTDELTLAIEQALENSLKITDEASAIEQANLPSLLVSASSENIKITHPNDLALAEFYLNKQANNTN

>sp_Q12ZQ0

MELPIAQFPDVQANRPKIPINLTRVGVTGVKKLVEIKRKDKRPIVLISTFEIFVDLPSDRKGANLSRNFEAMDEVLEKAINLPVYEIEKLCNDVAKSLLRRHEYATRSEVRMKSEYVVKREAPSTKMKCQEVVDIFAEATATRLEDGDIDVKKLIGAEVVGMTACPCAQEIMRDNAKTALRELGVGLETVMNFLNKVPMATHNQRGRGIISLEVSGDVDVSLETIIRIIESSMSSSIVELLKRADEALVVERAHQNPKFVEDCVRTMAQNIVSEFAHVPDSALVTIKQINEESIHRHNAFAERVALLGDLRDEIKNN

>sp_Q480B8

MLTASNTKAPEQTKVIVGMSGGVDSSVSAYLLKEQGYQVEGLFMKNWEEDDTDEYCAASQDLEDAQAICDKLDIKLHTINFATEYWDNVFEYFLAEYKAGRTPNPDIMCNKEIKFKAFLEFACEDLGADYIATGHYVQRELRDNSWKMIRGLDNNKDQSYFLYTLDEAQLAHTLFPVGHIEKPEVRAIAEKAGLITHNKKDSTGICFIGERKFKDFLGQYLPAQPGIIESAEGVAVGHHDGLMYHTLGQRKGLRIGGLADAGEEPWYVVEKDLLRNVLIVGQGHNHPRLFSKGLIANQLHWVDRKALTSSIQCTVKTRYRQEDVSCTVTPITDSATEEYQIDFTEQQSSVTPGQSVVFYKDDVCLGGGIIDTLIR

>sp_Q47Z78

MSEQKQSLSYKDAGVDIDAGNALVENIKGAVKRTTRPEVMGGLGGFGSVCQLPTGYKEPVLVAGTDGVGTKLRLAIDLAKHDTVGIDLVAMCVNDLIVQGAEPLFFLDYYATAKLDVAVASSVVEGIAEGCIQSGCALVGGETAEMPGMYHKGDYDIAGFCVGVAEKSRLIDGTNVAAGDQLIALGASGPHSNGFSLIRKVLEVNNTDTNELLEGKKIADHLLEPTKIYVKSVLELLKNVDVHALSHITGGGFWENIPRVLPETAQAVIKGDSWQWPSIFNWLQENGNITEHEMYRTFNCGVGMVIVVPADKVAQSIEVLTAHGENAWHLGEIADKADGEEQVVFA

>sp_Q47W60

MAKITTASLLNMKQQGKKISTITAYDASFAKLFDQAGIHAILIGDSLGMVLQGQDSTLPVTIEDMAYHTRCVKRGVEETLIIADMPFMSYANEEQALANAALLMQAGASMVKIEGGAWLNGTISALVERGVPVCAHLGLTPQSVNIFGGFKVQGRDDDKAQQMIADAKALEAAGAQLLVLECIPAILGEAITQALTIPTIGIGAGKDTDGQILVMHDALGIACNYMPKFSRNFLKDTGDIKKAVELYISEVSEGNFPGDEHIFK

>sp_Q12YV7

MDNDKHLKGTTTVGIVCTDGVVLATEQRATMGHFIASKTAKKIYQIDDLVGMTTAGSVGDAQQIVRIISVESKLFKMRRQESITIKGITTLLSNMLSGQRYYPLMVQLLIGGVDKNGPAIYSLDALGGNIEETRAVSTGSGSPMAYGVLEDRYTEDMTVVEGVDLAIRALHNAMKRDSASGENIDVVVITKDKYERLDPEAVMKKRELLN

>sp_Q12UJ5

MKELKIIVINNYGQFCHLIHRTVRDLDMDTKIVANTTSVEDILDEEPDGIILSGGPSMERVGSCQEYVESIDIPILGICLGHQLIAQTFGGHTGAGKLGGYAAIDVEVIEEDDILKGLGPRTSVWASHADEVTVLPDEFIHLARSDVCEIEAMRHEERPIYGVQWHPEVAHTDKGEELFMNFFKVCEDY

>sp_Q12V48

MIDLDTLKFDGNGLIGAIAQDNRTGEVLMFAFMNREALEKTIETGIAHYWSRSRQKLWKKGESSGHMQKVHELLIDCDMDAIILKISQEGGACHTGYRSCFYRNIEGDVVGEKVFDPADVY

>sp_Q486Y6

MALLQIAEPGQSTVPHEHRLAAGIDLGTTNSLIASVQSGNASTLSDDQGRDILPSIVSYQAGNVLVGQTAQALSIEDAQNTITSAKRLIGRSLKDIQSKYPSLPYEFCGDENHPEIMTRQGAVNPVQVSAEILKSLNLRAQAALGGELTGVVITVPAHFDDAQRQSTKDAAKLAGVSVLRLLNEPTAAAVAYGLDSGQEGVIAVYDLGGGTFDISILRLNKGVFEVLATGGDSALGGDDFDVVLVDYLVEQAGLVRPLSPSLERQLMQQACFAKEQLTTKEEVDITISLDSDSDWKTSLTKAQLNKLISSLVNKTLRACRRTLKDADITIDEVIEVVMVGGSTRVPLVRSEVEKHFNKTPLTSIDPDKVVAIGAAIQADVLVGNKPDSDMLLLDVTPLSLGLETMGGLVEKVIPRNTTIPVAKAQEFTTFKDGQTAMAVHVLQGERELVEDCRSLARFELRGIPAMTAGAAHIRVTFKVDADGLLSVSAMEKSSGVESSIEVKPSFGLDDNQISQMIKDSMSNAADDMQARMLKEQQVEASRVIESVQAALLADSKLLNSDEITVIENAIKSLAQVSQGQEIKAIENALDKLNDSTAIFAERRMDSSISEALAGQAVDKI

>sp_Q47VD1

MSQFNVLNRYLFTDAHARGELVQLSSSFESIIKNHNYPVGVEKLLGELLCATCLLTATLKFEGDITVQLQGDGPVGYMSVSGNNKQQMRGIAKMAEETSADTLQTLIGKGTMIITIRPNAGEAYQGVVALDEESLADCLAHYFDVSEQIPTKIWLFCDTEQQLAAGALVQLLPDGDGSTENKEQQQSDFEHLCQLTNTIKSEEVFSLEAEALLYRLYHQEQVNIFEPQMVSYLCGCSADKCLSAISQIEPSEIKAILAEHGKISMTCDYCITTYDFDELSLKSFISKVNH

>sp_Q47WC0

MFKESPIIRRVSRQIMVGNVPVGGDAPITVQSMTNTLTTDVAATVAQIKALEAVGADIVRVSVPTMDAAEAFREIKKQVNVPLVADIHFDYRIALKVAEYGVDCLRINPGNIGNENRIRSVVECARDNNIPIRIGVNGGSLEKDIQEKYTEPTPEALLESAMRHVDILDRLNFNEFKVSVKASDVFLAVESYKLLAKQIDNPLHLGITEAGGLRSGSVKSSVGLGLLLAQGIGDTIRISLAADPIEEIKVGFDILKSLKLRSRGINLIACPSCSRQEFDVVSTVNALEQRIEDIMTPMDVSIIGCIVNGPGEAMVSDLGLTGSSKKSGYYLDGIRQKERFDNTDLVDQLEQRIRAKARSMSERLDEKNKIDILTKD

>sp_Q483B3

MTASDPSVLSKPATGDTSFVVVIPARYQSSRLPGKVLADIDGKPMIQWVVEKAQLSGARQVIVATDNDEVAAVVNSFGAEVCKTRADHQSGTERLAEVMEKYQFSDDEIIVNVQGDEPFIPPDNIAQVANNLANQQQSSHVARMSTLAINIDSVDEAFNPNAVKVILDKDGYALYFSRATIPYDRERFLNSDATTEENIRAIGDFYLRHVGIYAYRAGFIKDYVNWPTSELEQVEALEQLRVLYQGERIHVAVANSHVPVEGVDTPEDLAKARAYATSLV

>sp_Q12Y52

MISVNEKGLAIIDEMLDWEEDVKIESKVLENGATIIDCGVNVEGGYDAGMYLSRLCLADLAEISYTKVDLEGLAVPAIQIATDHPTIACMASQYAGWRIAVGDYFGMGSGPARGLGLKPKELYEEIGYKDEADAAVLVMESDKLPTEEIVEYIAKHCSVEPQNVFIAVAPTSSIAGSVQISARVVETGIHKLESIGYDINKIKSGFGVAPIAPIVGDDTKCMGSTNDCIIYCGETYYTVEDGNAEELEDFVKKAPSSTSRDFGKPFYTTFKEAGFDFFKVDAGMFAPAKITINDLKSKKSFTSGRINPGILLESFGIKNV

>sp_Q9F1N3

MLVYLAEYLTQFYSGFNVFSYVTFRAILGLMTALVFCLWWGPKMIRRLQTLQIGQVVRSDGPESHFSKSGTPTMGGILILAGIFISVLLWGDLGSRYVWVVLFVLASFGLIGFIDDYRKVVRKDTKGLIARWKYILQSLAAIIIAFYLYASADTVGETQLVVPFFKDIMPQMGAFFIVLAYFTIVGSSNAVNLTDGLDGLAIMPTVMVAAAFALIAYLSGHVQFANYLHIPYLPGAGELVIVCTAIVGAGLGFLWFNTYPAQVFMGDVGSLALGAALGAIAVLVRQEILLVIMGGVFVMETVSVILQVGSYKLRGQRIFRMAPIHHHYELKGWPEPRVIVRFWIISLFLVMLGLATLKLR

>sp_Q480P4

MGQILYQLDDNSLTFPLIECALTEPNGLLALGGDLSPERLIAAYSQGIFPWYSDNDPLMWWSPNPRAIIDIDQLRINRTLRKAINKSPYQITLNQDFSQVTQLCANAPFRTDGTWILPEMEAAYLTLHQQGYAHSIEVWYTDEHDNKALVGGLYGVAVNGFFSGESMFYKQSNASKFALIALGQLLKSVDINFIDCQLLNPFLEDMGAKETSRDIFIHKQQHALTKTMPDDFWQPRTLTVI

>sp_Q47U38

MWYQESYDVIVVGGGHAGTEASLAAARMGCKTLLLTHNIDTLGQMSCNPAIGGIGKGHLVKEIDALGGLMATAIDHSAIQFRTLNSSKGPAVRATRAQADRILYRNYVRNTLENQENLTIFQQPCDDLILENDRVVGVSTQMGLKFKGKSVVLTVGTFLSGLIHIGLNNYQGGRAGDPASVNLAAKMRDMPFRMDRLKTGTPPRLDARSLDFSVMEEQAGDTPSPVFSFMGSQADHPEQISCFITHTNEQTHQHIRDGLDRSPMYTGVIEGVGPRYCPSIEDKITRFADKSSHQIFVEPEGLTTHEVYPNGISTSLPFDVQMNLVRSIKGFENAFITRPGYAIEYDYFDPRDLKQSLESKFVQNLYFAGQINGTTGYEEAGAQGLIAGANAANRVKERDEFTLGRDQAYMGVLIDDLATLGTKEPYRMFTSRAEYRLLLREDNADIRLTEQGRKIGLVGDTRWQRFNEKMENVELERQRLRSTWVQKDHTKIDQINALLKTPMSKEASLEDLIRRPEVNYTDLMKIEGLGPAIEDSQASEQIEIQTKYAGYIDRQLDEIAKKKRNEDTKIPRDFDYQQISGLSNEVVAKLKDACPETIGKASRISGITPAAISLLLVYLKKHGLLRKLA

>sp_Q12W69

MDLKTKYYLKSKFQEYYRTAKIHLPAKLPEREWGVLSFDDMPETVMRRHKSFGSAGEVEDYLTGMAPAHVYYSVAYYTYPNAPTMKEKQWLAADLIFDLDADHIPGAPNSYSDMLDHVKKETLKLYDLLTDDFGFKEEDIGAVFSGGRGYHFHISDPRVLSLESAERREIVDYISGRGLNLDKIFVKKGVSGDAGSEKATMNVFPSEDDGGWGGRINHHMIAYLRELAAKEDAEKLFTGFDRIGKKTAKRIVEILRDETQVDLLKKGNMEALSRVNKDIIQTLAERSVTELSASVDEPVTGDIKRLIRLPGSLHGKSGMCVTSLSISQLEDFDPLNDAIVFSDKPVKLKVIRTFAVQMKGKDLHVEEGVQELPEYAAIYLMCRGAAEYGP

>sp_Q487A3

MRVSDFSFDLPEALIARYPKAERTASRLMTLNGNSGAITDGVFTDIVAQLNSGDLLVFNNTRVIPARMFGQKASGGKIEVLVERVIDQNTALAHIRASKSPKVGNELFLGNEDSDVKVKATMVARHGALFELKFNSDESVLTILDKIGHMPLPPYIDRPDEDSDKERYQTVYNEKPGAVAAPTAGLHFDEALLERIKAKGVELAFVTLHVGAGTFQPVKVDEIADHIMHAEYVEVSDEVVAQIAKTKAAGGRVVAVGTTSVRSLESAAKAALDKNKPLSAFYGDTDIFITPGCQFQIIDALVTNFHLSESTLLMLVSAFSGYDHIMSAYQHAISQEYRFFSYGDAMFLTKQELIKQDTP

>sp_Q47VJ9

MTQRIAITPGEPAGVGPDLIITIAQQDWPVEMVVIASKALLQERSKALSLPLTIIDYDQHAPAKSQKSGSLTVLDVELTEPCVPGTLNSANGSYVVETLRIASEKNISGEFDAIVTGPVHKGLINKAGIAFSGHTEYFATQANCSDVVMMLATKGLRVALVTTHIPLAYVSKAITYERLQKVTRILHKDLQEKFGIKSPKIYACGINPHAGEDGHLGREEIEIMEPAFAELRADGIDIIGPLPADTIFQEKYLAEADAILAMYHDQGLPVLKYKGFGSSVNITLGLPFIRTSVDHGTALELAGKGTADSGSFIEAMNNAINLASNK

>sp_Q47XK1

MKIYFDENMPFAKEFFSELCHLNNGIDGEEQGELVPFSGRTLTAAQVADADVLLVRSITQVNEQLLHLNDKISFVGSATIGTDHIDLSYLAKRNITFQSAPGCNAISVAEYVLSALVVLAERYLLTLSSLTVGIVGGGNTGTRLSEKLTALGIQHKICDPLLAEKQKQDKSHPPTDQRHYVPLVDVLACDVISLHVPKVVGGEHPTNKLINAENLALLREDQILISACRGDVIDNHALLALKTAGHGVKIVLDVWQGEPDVLEALIPYTEIATAHIAGYSLEGKARGSEMLYQALCQQLAITPKYQLANFLPSASIPAIEINQDFNQILLNQLVKMVYDVRRDDAIFRQQLFVQGFDSLRKNYPVRREFSAVTVNLSSTTYSDVPHRLGFNKN

>sp_Q48AC6

MITISENAQQHFIKLLSQQAEGTHIRVFVVNPGTAKAECGVSYCPPDAVEADDIQLPFEGFSAMVDADSKGFLEDAEIDFTTDQMGSQLTLKAPNAKLRKVADDAPLFERVHYFLQAEVNPQLAGHGGECTLVEITDDGYAVLQFGGGCNGCAQIDVTVKDGIEKQLIELMAGEIKGVKDATEHERGDHSYY

>sp_Q47XN8

MTVQSNNKLASQVCEACHVDAPKVSDEELKELIGLIPDWVPQVRDNVMMLEREYKFKNYKLAWAFANKVSELAESEFHHPSILLEWGKVTVTWWTHSIGGLHKNDFICAAKTDQLGD

>sp_Q47VG7

MIFMTNEIVNSTSTSQKLILASQSPRRRELLAQLGYQFSVQASDIDETVEKAETAYDYVLRLAKQKAQHVLDLLPEAERVYSYVLGSDTSVVFNGEILGKPDNEENCIDTLSLLSGNQHQVLTAIALVSHAGVKGQVITTEVTFKTLTKAEISAYWLTGEPQDKAGSYGIQGIAGQFVKTINGSYSAVVGLPLYETAQLLANAGFVGSIHTK

>sp_Q12WN0

MKPVNAKIVEIVEESPTIRTFRFDISFDDALPGQFVMVWVRGVDEVPMGSSYLNGITVQNVGDATSKLFDMKVGDSVGLRGPFGKGFTLPKKDEHILIIAGGVGAAPLGPLADYAAECGAKVTTILGSRECYELVFEDRFSSAGELHITTDDGSAGTCGFVTTVLADMDVSVYDRICVCGPEIMMANVMKALQEKDALDRSEFSMHRYFKCAIGVCGACCMDPDGLRVCKDGPVLNGSLLIDSELGDHKRNSSSQRVRV

>sp_Q48AN2

MKDYQREFIEFAIEKQVLRFGEFTLKSGRVSPYFFNAGMFKTGGDLARLGRFYAATLMDAKIDFDLVFGPAYKGIPIATTTTVALYDHHNVDVPYCFNRKEAKTHGEGGSLVGAELEGKIMLVDDVITAGTAIRESMEIIKAHGAQLSGVLIALDRQEKGQGQLSAIQEVERDFGTQVAAIVTLGDVVTYLEEKLEGKVANQPELAENLASIKKYRLNYGI

>sp_Q12ZJ3

MSHNTKGQKIRLAKAHNQNQRVPTWVIIKTNRKVVSHPKRRHWRRNSLDVK

>sp_Q47XA7

MSETNQVQNHAFASDNAKILQLMIHSLYSNKEIFLRELVSNAADAADKLRFKALSDNTLYENDGDLRVRVSCDKENNTLTISDNGIGMNVDEVIEHLGTIAKSGTAEFFSQLSGDQASDSQLIGQFGVGFYSAFIVADKVTVRTRKAGDSATDGVEWISAGEGEFTTAKIEKTNRGTDIILHLKEDESEYADDWRLKSIVTKYSDHISVSVEMLTAEVPAVEAVAEVTDEKDNVTRPAADAVDAIPALWEPVNKATALWTREKADITDEEYKEFYKHVSHDFGDPLLWEHNRVEGKTEYTSLLYVPTKAPFDMYNREKQHGLKLFVQRVFIMDDAEQFMPTYLRFVKGLLDSNDLPLNVSREILQDNKVTQAIRKGCTKRVLKMLEKLGNKDADKYQGFWDEFGQVLKEGPAEDHANKEQVAGLLRFASTHEDSTTQNVSLASYIERMKEGQDKIYFVVADSFEAAKNSPHLEVFRKKGIEVLLMSDRIDEWLVSHLTEFDGKQLQSVTRGGLDLGDMDDAETKEAQEKLEKEYDSVVKRIKASLDGKVKEVKLSQRLTDSPACIVADDDDMSSQMAKLMASVGQEVPDTLPIFEINGEHALVKHVADEQDDDMFNQWVEVLFEQAMLAERGSLKDPASFVSRLNKLMLSLTK

>sp_Q47WG3

MSNIAILAGDGIGPEVMVEAKKVLNTVASKFDFEITTQDYDIGGAAIDNHGNALPDSTMAGCIESDAILFGSVGGPKWANLPPTEQPERCALLGLRSHFDLFCNMRPATLQPALSSLSTLRSDISEQGFDVLVIRELTGDIYFGEPKGRRGEGEEETGFDSMFYSRREVKRISHLAFQAAQKRNNKVTSVDKANVLATSQLWRQVVEEVAVEYPDVELEHLYVDNAAMQLVRDPNQFDVMLCPNLFGDILSDICAMITGSMGLLPSASLNSDGFGMYEPAGGSAPDIAGLGVANPIAQILSAALMLRYSLNQGAAAKAIEDAVSNALDNGVLTADLLPANERKNAKSTSEVGDYICKQIESA

>sp_Q48AS5

MSVARLTTYNFRNLSSVAIDLHPKLNFFIGNNGSGKSSLLEALFFLGHGKSFRTSKVEHLACYETDNFVVSIKDVNDLQLGLSKNLQTGVTLIKINGERHARLSELAKNIAVQIVTPESFKLFFGGPKERRRFIELGMFHVKHDSSKQWREFNRVLKQRNACIRHNLDKATFDYWTGLFCQLSEQVAEVRSQYITNLISELPYWLEILLPNIADKVTVQYLQGWPQKKNLMDSLNDSHEREQAFGYSIYGAHKFDVKFLIAKQALESQLSRGQQKLFLLALTFAQAKLIARVNRVKPILLIDDIGAELDINSRESLSQALSILDCQVIITAIEEGVLQPFIDDVSVADKESSKKTKYHMFHVKHGGILPVNNSVKIE

>sp_Q47Y87

MTIKLIAGLGNPGPEYSKTRHNAGVWFVEELARSHNISLRPEKKYSGLYGKGLIAGNLVHLLIPTTFMNRSGQAVAPLANFYKISVDEILVAHDELDMLPGVCKIKKGGGHGGHNGLRDIIDRMANNKDFYRLRIGIDHPGHRDKVTGHVLGKAPSAEQAKIEQAIDEASRCLDIWLKDDLKKAQNRLHSFKAE

>sp_P96174

MANPLFRKHIVSINDISRNELELIVKTAAKLKKQPQPELLKNKVIASCFFEASTRTRLSFETAIQRLGGTVIGFDNASNTSLAKKGETLADSISVISSYVDAFVMRHPQEGAARLASEFSNVPVINGGDGSNQHPTQTLLDLFSIYETQGCLDNLNIALVGDLKYGRTVHSLAQALAKFSGCKFYFIAPDALAMPEYICDELDEHNVSYACYNSIEEVVPEIDVLYMTRVQKERFDETEYQHMKAGFILSASSLKHAKDNLKVLHPLPRVDEIAVDVDKTPYAYYFQQAENGVYAREALLALVLNATIEG

>sp_Q485R8

MKIISAEQVHQNLNFEELIPLLKQSFSRPFSMPQRQVYSLAPEQSENHDAFALLPSWNEEVIGNKAFTYFPDNAKKHDLPGLFSKIMLFKRQTGEPLALVDGTSVTYWRTAAISALASQLLSRKNSQHLMLFGTGNLASYLVKAHLTVRDIKQVTLWGRNAKKVSKLIADFSILYPAVTFKTSVDVNAEVASADIICCATGAKTPLFDGNSVSAGCHIDCLGNHMTDARECDTTTILRARVFVDSLTNTLNEAGELLIPMAEDAFNKDEIVGELADMCKTPSMLRQSSDEITLFKSVGTAISDLVAAHSVVEKLAD

>sp_Q12WI3

MRSILLLSSGLDSVAALAIALDNSDVFMALTFDYGQRSVKKEIEYSRMVCEHYGIEHRIITLSWLSSITNTSLVNKDLDVPELSINDLIDGPDNITEDSAKSVWVPNRNGVFINIAASFAESHGCEYVIAGFNGEEAKTFPDNSKEFVNAVDECFSYSTANGVKVLAPLIDMDKVSIVRKAMELGAPLEYSWSCYHGAEEPCGVCESCMRRKRAFLASGFEDPHIKKRADFKR

>sp_Q47YB3

MANYQNATELSKLTLGKSTQYCSEYTADLLQGVPRSLNRDDLALNQSNLPFVGEDVWYGYELSWLNGKGKPVVAVAEFRFACTSDNIVESKSFKLYLNSFNQTRFSSIKDVEKVLTKDLSKIAGSEASVNLFGVDHCPALDIAKKSDKCICIDGEDISIDNYQYDPQLLATAQDERSGSQIEEYLVSHLLKSNCLITNQPDWASIYIHYRGKAIDHSSLLKYLISFRQHNEFHEQCVERIYCDLQQFCQLDELTIFARYTRRGGLDINPFRSSHIEQAPFARTLRQ

>sp_Q47YI6

MKVKVAILGCSGRMGRNLIQAAHEHASIELVGGSVRTSSSFVDFDLGELAGIGAIGIKTSTTLAQLATADVFIDFTSIETTLENLTWCNENKKALVIGTTGFSDEQVQVIEQAGKTMSVILAPNTSVGVNLMFKLLQVTAKAIGDYTDIEIFEAHHRFKKDAPSGTAVKMGQVIADTLGRDLNKVAVYGREGITGERDRETIGFATVRAGDIVGEHTAFFADLGERLEITHKASSRMTFALGAMRAAFWLKDADAGFYDMQDVLGLKD

>sp_Q47WJ3

MTITEPYSPNNQPITFAQITDSHLFSSVDGLHHGHNVLANLKKVLLSICDNPSIKYIIFTGDLTQDHTEQSYQNFVDCVLECHITVPIYYLAGNHDEPKLLDKYFSASPFQADKEINLSHWQVQLVDSKSATPAGYVGEQALVKLKDAIQKNKNQLLMMHHHPIDVGYFIDKHGLQNKDAFWQVINSYDNIKAIACGHVHGDMTLTNAITSPINEPVVLYTCPATSIQFDPTVDGVAALSKGPGYRLFSLYADGQLNTEVVML

>sp_Q12UK3

MMLIGEALIGEAPELAHVDLMIGDKEGPVGQAFATGMTQLSAGHTPVLSVIRPNLPTKPSTLIVPKVTVKGMDQASQIFGPAQAAVSKAVADAVEEGLIPKEKAEDLVIIASVFIHPQAVDYNRIYRYNYGATKLALKRALDGFPDIDTVLHEKDRAAHAVMGFKISKLWDAPYLQVALDNPNLPVILNIIKQLPKSDHLILEAGTPLIKRYGVDVISKIREVRPDAFIVADLKTLDTGNLEARMVADATADAIVVSALAPIATLNKVIEEAHKTGIYAVMDTLNTPDPVAVLEQLDVLPDVVELHRAIDIEGTAHAWGSIEGIKALAVKRSSKVLVAVAGGVRVDTISDALGAGADILVVGRAITNSKDVRQAADRFIEGLNKPEIDQFRIMTDF

>sp_Q486C8

MFKSTLPIAAAISVALTSMVLPAKALAANEAKTNQFWWPDQLSLSPLRQHGAESNPYGEQFNYAKEFASLDLAMLKKDIQTTLTDSKSWWPADWGHYGPLMIRMAWHSAGVYRVHDGRGGASGGQQRFAPLNSWPDNVNLDKARRLLWPVKQKYGRKISWADLMVLSGNVALESMGFKTFGFAGGRTDDWEPDLVYWGPETAMLSDKRRDKKGKLKGPLAAVEMGLIYVNPEGPHGKPDPLLAANDIRMSFGRMAMNDEEIVALLAGGHTLGKAHGAKKPNGCVGAEPAAADIEAQGLGWKNKCGTGVGADTISSGLEGAWTVTPTQWSSNYLDNLMNFNWVLTKSPAGAKQWIPDNKAAANLVPDAHIPNKRHAPIMFTTDIALKEDPQFRKIVERFRADPTQFDLAFAKAWFKLTHRDMGPRARYVGAEVPSEVLMWQDPIPAINYQLITDKDIKQLKKQITNSGLTTSELVRTAWAAASSHRVTDMRGGANGARINLEPQNSWAVNNPKELGKVLAKLEGIQARFNKKSAKTKVSLADVIVLGGATAIENAAAKAGNRITVPFSPGRADASQAQTNVKSFNYLKPKADGFRNFYTDDSYSSPAEMLVDKANSLGLNVPEMTVLIGGMRALDANYDASSYGVLTNNPGVLTNDFFVNLLDMKTVWSKDKSNAGIYIGHDRASGTEKWQATPVDLIFGSSSELRAIAEVYASDDADKKFINDFTKAWVKVMQLDRFDLK

>sp_Q47WG2

MTTSTPQTMYEKLWQTHLVEATKGETPLLYVDRHLIHEVTSPQAFANLRFHNRPVRHPERTIATMDHNISTRSIKIDAAGEGAANQLRALAINCKDFGIELFDMGHKNQGIAHVIGPELGLTLPGTIIVCGDSHTATHGAFGALAFGIGTSEVEHVFATQTLRQNKAKTMKIEVKGHVGAGISAKDIILAIIGKTGSAGATGYVVEYCGEAIEALSMEERMTVCNMSIEFGAKAGLIAPDQTTFDYVEGKEYAPKGEVFEQAVVDWKNLKSDADAQFDAVLTLDAKDIKAQVTWGTNPGQVISVDGTVPSPEDFSDPVEKESCVSALNYMGLTAGTKMTDIHVNKVFIGSCTNSRIEDLRAAAGVVQKYEGQQVVKTIDAIIVPGSYRVKEQAESEGLDKIFTDAGFEWRLPGCSMCLGMNDDVLEEGDRCASTSNRNFEGRQGRGSRTHLVSPEMAAAAAITGHFVDLNA

>sp_Q47XB4

MMIPAIDLIGGEVVRLYQGDYAQKTNYQYTVQDRQQAYAESGATVMHFVDLDGAKDSTKRQLKTLKTVVNHPSMIIQVGGGVRCEDDVKQLLALGADRVVIGSLAIKQPELVTQWVKTYGCEKIVLALDIKIDAQGNKTLPTHGWIEDSGVNLEDLLAQYQDAGIKHVLCTDISKDGTLTGTNVDLYSEVCAKYPDIDWQASGGIGSLADIKALIPTGVSGVILGRSLLEGKFTLEEAIACWPKTSANNAESNTSSNTGDK

>sp_Q12WZ6

MMIRELDIPRDIIGFYEDSGIKELYPPQAEAIEMGLLEKKNLLAAIPTASGKTLLAELAMIKAIREGGKALYIVPLRALASEKFERFKELAPFGIKVGISTGDLDSRADWLGVNDIIVATSEKTDSLLRNGTSWMDEITTVVVDEIHLLDSKNRGPTLEVTITKLMRLNPDVQVVALSATVGNAREMADWLGAALVLSEWRPTDLHEGVLFGDAINFPGSQKKIDRLEKDDAVNLVLDTIKAEGQCLVFESSRRNCAGFAKTASSKVAKILDNDIMIKLAGIAEEVESTGETDTAIVLANCIRKGVAFHHAGLNSNHRKLVENGFRQNLIKVISSTPTLAAGLNLPARRVIIRSYRRFDSNFGMQPIPVLEYKQMAGRAGRPHLDPYGESVLLAKTYDEFAQLMENYVEADAEDIWSKLGTENALRTHVLSTIVNGFASTRQELFDFFGATFFAYQQDKWMLEEVINDCLEFLIDKAMVSETEDIEDASKLFLRGTRLGSLVSMLYIDPLSGSKIVDGFKDIGKSTGGNMGSLEDDKGDDITVTDMTLLHLVCSTPDMRQLYLRNTDYTIVNEYIVAHSDEFHEIPDKLKETDYEWFMGEVKTAMLLEEWVTEVSAEDITRHFNVGEGDIHALADTSEWLMHAAAKLAELLGVEYSSHAYSLEKRIRYGSGLDLMELVGIRGVGRVRARKLYNAGFVSVAKLKGADISVLSKLVGPKVAYNILSGIGVRVNDKHFNSAPISSNTLDTLLDKNQKTFNDFQ

>sp_Q47WP3

MKNIRNFSIIAHIDHGKSTLSDRLIQHCGGLQAREMEAQVLDSMDIERERGITIKAQSVTLDYKAKDGEIYQLNFIDTPGHVDFSYEVSRSLASCEGALLVVDAGQGVEAQTVANCYTALEMDLEVLPILNKIDLPQADPERVCEEIEHIIGIDATDAVTCSAKTGIGIEDVLETIVKNIPSPAGQIDAPLQALIVDSWFDNYQGVVSLVRVINGEVKKGDKMLVMSTGQVHQIDKVGIFTPKQTDTGVLRAGEVGFIIAGIKEIHGAPVGDTITISKKETANALPGFKKAQPQVYAGIFPISSDDYENFRDALNKLSLNDASLFFEPENSSALGFGFRIGFLGMLHMEIIQERLAREYDLDLITTAPTVNYEIASTNGDVISIDNPADLPAINNIEEIREPIVQANILVPQEYLGNVITLCIEKRGVQKDLIYHGNQVAVTYELPMAEVVMDFFDKLKSTSRGYASLDYHFIRFEAADMVRVDVMINGDRVDALAMITHRANSVARGRLLVDKLKELIHRQMFDIAIQAAIGNNVIARTTVKQLRKNVTAKCYGGDISRKKKLLQKQKDGKKRMKQVGNVEVPQEAFLAVLKLDS

>sp_Q12WM8

MILGTRGSALAIAQADLVTKMLEEKGHELTRNVIKTSGDVFTDRPLHEVAGVGVFVRELDDRMIEGEVDIAVHSMKDLPTVRPPELAIAAVLKRDSPYDVLLTADGSTLDELPDGAIIGTTSMRRRAQLLRYRPDLNVEDLRGNINTRIQKLKAGQYDGILLAEAGLQRMGWDMDVQRLPAEAFCPSANQGTIVVVTRADDEAERACSVLNHERSRMETEVERLLITDVEGGCIVPIGSFAQMNEDGDEIHVLVEVLAVDGTREIRIEDDIPVKNYREHALSIGRMLVEMGGKELVQEAVCEMSGCDDE

>sp_Q487V5

MTLFKHWQTLYINVNLATMTDGSESYGEISQGALAISAGKIAWLGKESDLPEHFSVTDEDIEVIDCKGQWLTPGLIDCHTHLVYGGNRANEFEMRLQGKSYQEIANAGGGIVSTVTATRRASEQELLASALPRLTALHQQGVTTVEIKSGYGLDTINEIKMLKVAGLLADELPVTIKRTFLGAHALPIEYKDNAEGYLDVVCEEMLPQVVSENLADAVDVFCEGIGFSLAQTKRVFDAAQSHDLPIKVHAEQLSNLGASELAANYNALSSDHIEFLDEAGIKAMKKSGMTAVLLPGAFYFLRETQLPPIELLRKHQVPMAVATDANPGTSPIHNIHLMLNMACTLFRLTPSEALAGITCYGAKALGLSESKGQLAVGYDADIALWNINQPAELCYQFGVNPLSRLIQNGQQVLMNESA

>sp_Q12UG6

MVLKAIVIDIDGTITNPDRSLDLDVAKRFRELNVPVILSTGNPLCYVHAAAKLIGISGIVIAENGGVISTGFDSPSIIADGKEECEKAYELLSQYHDLVKLDDAYRKTEVVLNRDVAVEDLRSTLSENGIDIEIIDTGYAIHIKSTAMNKGTGLLKVAELMGLEPTDYLAIGDSCNDAEMMQVAGFGIAVANADSDAIKAARHITKASFGKGALEAIEYALSNGLL

>sp_Q482K5

MKTLVLGSTSPFRKTILEKLQLPFHCAKPNVDETEQKNESPQALVERLAIEKAKAVSSEYPNALIIGSDQVAVCEEEILGKPHNFDNAVVQLNKFSGKSITFYTGLCVYDSEKDKTIALVEPFIVHFNQLSQKDIANYLNAEQPYNCAGSFKSEGLGICLFSKLEGDDPNTLIGLPLIKLVGLLKQHGIDVLAEQSKLTV

>sp_Q9S0K8

MTKLERQVTICNKLGLHARAATKLAILASEFDAEITIVQGEKKASAASVLGLLMLETGMGKTITLLGKGQDADAALDAICALVDAKFDEAS

>sp_Q47VU0

MLEQIKNNFTESIQTQIAASELLGPSIEHAGMMMVQCLLGGNKIISCGNGGSAGHAQHFCAQLLNKYETERPSLPAISLNSDISTITSIANDYQYDEVFSKQIRALGHNGDVLLAISTSGNSRNVVKAIESAVSRDIPIIALTGFDGGDISGLLGEGDVEIRVPSARTSRIQEVHLVVLHSLCEIIDTTLFPQGDS

>sp_Q481H3

MADASIEFKGTSFTLSVLHLKTSKLADIRADLVKKVAQAPDFFYLVPVVVNIEQLDCSIDYQAVKTLIEEFNFTFVGFTGSVDKEQRKLIRELGFSFVNTTRVDTSQKAAIAEKAIVAESKVTAAIPECNLYTDKVHRGQIRSGQQIYAKDQNLVVIGSVSAGAEVIADGNIHVYGSLRGRAIAGAKGHHKAQIYCQNLEAELVSINGNYWLSESMEQHWGSPVYIHLTDSELTSSKLI

>sp_Q486L6

MSRHFFTSESVSEGHPDKIADQISDAVLDAIIAKDKHARVACETMVKTGVAIISGEVSTNAWVDLEKLTRNVISDIGYTSSDVGFDGATCGIMNLIGQQSPEIAQGVDRSNPEEQGAGDQGLMFGYATNETPTLMPAPLYYSHRLVERQAEARKSGVLPWLRPDAKSQVTFIYEDNKPVAIDTVVLSTQHNPDIKQEDLVSAVMENIINHVLPAELLTEDTKYHINPTGRFVIGGPVGDCGLTGRKIIVDTYGGMARHGGGAFSGKDPSKVDRSAAYAGRYVAKNIVAAGLADRCEIQISYAIGVAEPTSISIDTFGTGSISEERLVEIVREHFDLRPYGITKMLDLLHPMYQQTAAYGHFGREPFEMTVGDDTFTAFSWEKTDKADDLRKAAGL

>sp_Q12TP4

MERATFAAGCFWGVEAAFSKVEGVISTKVGYTGGTLKDPTYKDVSTGSTGHAESIDIIFDESVITYGELLEVLWNTHDPTTKDSQGPDHGSQYRSAIFYHDDAQREAALRSREQLERSGKYDSTIKTEIVKASEFSPAEDYHQKYFQKLQFKR

>sp_Q47VD0

MTAPKNSIDLSQYGIDDVNEVVYNPSYELLFSEETKAGLEGFDKGIVTELGAVNVDTGIFTGRSPKDKYIVRDDVTRDTVWWSDQGKNDNKAMTPETWDHLKGLVTTQLSGQRLFVVDTFCGADEATRLKVRFITQVAWQAHFVKNMFIRPTDAELENYEPDFVVMNGAKTVNDKWEEQGLNSENFVAFNLTEKIQLIGGTWYGGEMKKGMFSMMNYYLPLQGIASMHCSANVGEDGDTAIFFGLSGTGKTTLSTDPKRQLIGDDEHGWDDNGVFNFEGGCYAKTINLSKENEPDIYNAIRRDALLENVTVDANGKIDFDDNSKTENTRVSYPIHHIDNIVKPVSRAGHAKKVIFLTADAFGVLPPVAKLTPEQTEYYFLSGFTAKLAGTERGITEPTPTFSSCFGAAFLSLHPTQYAEVLHKRMDDAGAEAYLVNTGWNGTGKRISIKATRAIIDAILDGSIDNAETETVPFFNLEVPKVIAGVEGDILDPRNTYEDPSVWNDKAVDLAKRFVNNFDKFTDTDNGKALVAAGPQL

>sp_Q12UX3

MIPKKAFVVKGTGVHKDKLASFELALRDAGIEKFNLVTVSSILPPNCSIVSRKEGLEELAPGQIVYCVMAKNQTNEPERMIAAAIGNAVPVDSNDYGYISEHHSFGEDERTAGIYAEDLAATMLATTLGIEFDADSAWGERERVYKASGHIFDTVHYCRCVKGDENGLWTTVVVSMVFVL

>sp_Q47UY5

MKAGIIGAMEPEVAILKEKLTDAKSTEHAGYTFHQGQLDGSDVVIVQSGIGKVAAALATAILIDRFQVDYVVNTGSAGGFDASLKVGDIVVSSEVRYHDVDLTAFGYEIGQLPANPAAFMPHDDLVAAAKKGIEQLSQTAGENIKAVTGLITTGDTFMTKEEDVAKARANFPTMAAVEMEGAAIAQACLQLKTPFVVIRSLSDIAGKESPHTFEEYLETAAVNSSQLVLNMLGQLKGKVLSAA

>sp_Q12ZJ7

MSETSEQDARNLAAQHRELQQNAESVNQQLGMVQMSIEDCTRAILTLEELKSASGAINTMIPLGAGALIHANIADVDKIVVSVGAGISVEKTPTEAIETLTQRKEELGKVVERLNGTLTQIGQRLASIESAVGNRPPQ

>sp_Q47VZ2

MSSKNSLSDKIKITFQYIMPKHAISRLVGKLAAAKMGWLTTKLISMFIKAYGINMNEAKLKKASDFDTFNNFFTRELEEGARIIDNDENTICYPVDGAISQQGDIIDGQLIQAKGFNYSVTSLLGGDEKTAAPFQGGKFSCIYLAPKDYHRIHMPMAATLREMIYVPGELFSVNPLTAQNVPDLFARNERVVAIFDTEMGELAMVLVGATIVASIETTWAGTITPPAGKDIFRWQYPKDGADAITFEKGDEMGRFKLGSTVVSTFAPNMISEFATDAGPGTVTRLGEIYAALDKSAS

>sp_Q47UW0

MKDLLKFLKQQNQTEDFDGIRIGLASPDLVRSWSFGEVKKPETINYRTFKPERDGLFCARIFGPVKDYECLCGKYKRLKHRGVICEKCGVEVTLTKVRRDRMGHIELASPVAHIWFLKSLPSRIGLLLDMTLRDIERVLYFESYVVTEPGMTTLEKSQILTEEEYLDALEEHGDEFDALMGAEAVLALLQQIDLDGEVAQMREELPEIGSETKRKKITKRLKLMEAFAASGNKPEWMIMNVLPILPPDLRPLVPLDGGRFATSDLNDLYRRVINRNNRLKRLLDLVAPDIIVRNEKRMLQESVDALLDNGRRGRAITGSNKRPLKSLADMIKGKQGRFRQNLLGKRVDYSGRSVITVGPTLKLHQCGLPKKMALELFKPFIYGKLEARGLATTIKAAKKLVEREGAEVWDVLDEVIREHPVMLNRAPTLHRLGIQAFEPVLIEGKAIHLHPLVCAAYNADFDGDQMAVHVPLTIEAQMEARTLMMSTNNVLAPANGEPIIVPSQDVVLGLYYLTRFRINGLGEGMYFTDEKEVEKAYRTGVAELHARIKVRITEHVKNADGEWEPVTKLRDTTVGRAIMWQVCPKGLPYDLIDKPLGKKPISKLINHAYRNLGLKETVMFADQIMYTGFHYAMIAGCSVGIDDMVIPEAKYTIVEDSEAEVAEIQAQFDQGLVTAGEKYNKVIDIWSSANEKVSKAMMDNLSKEMVMNRDGEMEEQDSFNSIFMMADSGARGSAAQIRQLAGMRGLMAKPDGSIIETPIKANFREGLTVLQYFISTHGARKGLADTALKTANSGYLTRRLVDVAQDLVVTNHDCGTHDGLLMTPLIEGGDVVEPLRERVLGRVVCDDVLIPGTDEVLLPRNTLIDEALCDVIEDNSVDQIKVRSIITCKTDFGICANCYGRDLARGHMINQGEAIGVVAAQSIGEPGTQLTMRTFHIGGAASRATAESSVQVKNTGTLKLQNAKFVTNSEKHLVITSRSSELTIIDEMGREKERYKVPYGSVLSKNDGEAVNSGDTIANWDPHTHPIITEVAGKVQFVDLADGVTMVRQTDELTGLSSIVITDAAQRNATGKEMRPALKLVDAKGKEVMIAGTEIPALYYLPGNAIVNLEDGADVGVGDALARIPQASSKTRDITGGLPRVADLFEARKPKLPAILAEKTGVVAFGKETKGKVRLLITQPSGEVYEEMIPKTRLLNIYEGEPVIKGEVIADGPESPHDILRLRGVAPVANYIVNEVQEVYRLQGVKINDKHIEVIVRQMIRKCEILDAGDSTFLKGEQVEVARVNISNRELEAEGKQPAEYEMQMMGITKASLATESFISAASFQETTRVLTEAAVAGKKDGLRGLKENVIVGRLIPAGTGYSYHQERARRKAAALAPAVESTVSADDAEKALTDALNSDLLSGNH

>sp_Q12WT0

MPNGKYAAHRLQQVRKDARWKDTGYSRRTLGLDIKSDPLSGAPQGRGIVLEKVGVEAKQPNSAIRKCVRIQLIKNGRQATAFCPGDGAINFIDEHDEVTVERIGGRMGGAMGDIPGVRFKVIAVNNVSLREMVIGRKEKPRR

>sp_Q12ZT7

MTQTVKNFGRGANECKRCGRKQGLVRKYGIYLCRHCFREIAHDMGFEKYS

>sp_Q47VJ8

MNDKKHLGHQAKKRFGQNFLHNDAVISDIVDAINPEPGENLIEIGPGLGALTEPVIERAGKLSVVELDRDLAHRLRHHPFLAKDLTIYETDALKFDFSELATEEQPLRIFGNLPYNISTPLIFHLLTFKDKVKDMHFMLQKEVVERMAAGPHCKAYGRLSIMTQYQCQVFPVMEIGPEAFKPAPKVDSAIVRLIPHAHIENPVKDINALNTVCLAAFNQRRKTIRNTFKKLITEAQLAELNIDANLRPENLSLDEYKKLADFIVDNPPEAAPVKEKRRMAKNKMTEPANNNLNENSAPEVD

>sp_Q47UT5

MSSSEKQLSTPATIQTASTITICALYKFVRLDAYEALREPLSNKMASVDVKGTLLLAAEGINGTIAGPQTGIDTVLAFLGEQPGLDNISHKESYSEENPFHRTKVKLKKEIVTMGIEGIDPNQVVGTYVKPKDWNALISDPEVVLVDTRNDYEIEIGTFKNAINPNTETFREFPDYVAKNLDKNKHKKVAMYCTGGIRCEKSTAYLKEQGFEEVYHLEGGILKYLEEVPSTETMWEGECFVFDGRVAVNHELEQGQYDQCFACRFPLTDVEKESEHYVKGVSCHRCHDKVSEQQRSRYAERQRQISLAEERGESHIGGDIQNIIEERRQEKNDKKAKQANK

>sp_Q484F1

MSHVSQETAQEISEEREARAIVYNFLSSLFAKEVTSDLVAQLTSAQGQSFLKSLALDPSLSASVNEINTKLVKLNSKESLLELAADFCGLFLVDGRTSVSPYAGQYLSVEQGGEPSEKLSNAAINESGSKSKKNKAQLFGELHQQMTEFLTDNKLQIHSDFPEPGDHIAVILAYIAHLCVTSGSEQQLNFINSYLMTWLSDFTQQVNKHDHGQFYCFVADLTFEWLKVDTEFLLSD

>sp_Q12U93

MKLDEVTISRAIIEEFSKVFLDYTDVDVALVGGGPANLVAAKYLAEAGLKTVIYEKKLAVGGGMWAGGMMFPRIVVQEDALHILDEFGISYHEYENGYYVANSIESVGKLISGATSAGAEIFNLVNVEDVMIRENDEICGLVINWTAVEIGKLHVDPLAIRSKVVVDGTGHPAVVCSTVQRKVPGAKLGELGVVGEKPMWADVGEKMLLDTTKEVYPNLYVAGMAANAVAGAPRMGPVFGGMLLSGKQVAELIIERLG

>sp_Q482T9

MADITVAELAKEIGTPVDRLVTQLADSGVNKSATDAISQDEKEALLGHLKKQHGDESEAKPNKLTLNRKTKSTLTMGHGSKAKSVNVEVRKKRTYVKRSEVEDEKLAEEAAKAEAEAAILAEADAKAKAEAAAKEAENEKGVAAAKAEVEAERKAEAKIEAAAKAKIAAVEKAKNVEQAPEKVAETEEAKKLRLAQEKETLAKVEAEAAAAAEAAKKLAEENEGRWKEQEAERKAKEKEVVHLTSSVYAQEAEDKSDSADESGRRRKKKKAPDRNARGRNSGRGKGKTLSSPQSLKHGFTKPVETKLQDIRIGETISVAELANKMSKKGAEVVKAMFKLGAMATINQVIDQETAALVAEDMGFEVVLVKENALEEAVLADRNDTGEEITRAPVVTIMGHVDHGKTSLLDHIREAKVADGEAGGITQHIGAYHVETGHGMITFLDTPGHAAFTAMRSRGAKATDIVVIVVAADDGVMPQTIEAIQHAQASEAPIIIAVNKMDKESADPDRVKSELSQHGVLSEEWGGEVQFCHVSAKTGLGIDELLDSILLQSEVLELTAVVDKMANGVVVESKLDKGRGPVATVLVQEGTLKQGDIVLCGLEYGRVRAMRDENGKTIQSAGPSIPVEIIGLSGVPISGDEATVVKDEKKAREVALFRQGKFRDVKLARQQKAKLENMFASMAEGDISEVNVVIKSDVQGSLEAISDSLLKLSTDEVKVKIIGSGVGAITETDATLAAASNAIVVGFNVRADASARKVIESENIDLRYYSVIYALIEEVKQAMSGMLAPEFKQEIIGLAQVRDVFKSPKIGAIAGCMVTEGVIKRSAPIRVLRENVVIYEGELESLRRFKDDVQEVRNGTECGIGVKNYNDVRVGDQIEVFETIEIKRSL

>sp_Q482G2

MTKSELIEKLADKLSHLSAKEVEKSIKEILELMAQSLSKGERIEIRGFGSFSLHYRAPRVGRNPKTGESVELSGKYVPHFKPGKELRERVNLSVA

>sp_Q12Z05

MIRSILDNDLYKFTMQMAVLELFPNARAEYRFINRGAQSFTNDFVNELRRIINKDISKIALSEDEYIWLKDNCPFFKPSYIEYLKNFRFNPEEVKIVLTEDNELELCIEGPWHSSILWEIVLMSTISELYFTVTDNKGEEISASNANDPENTLMEEYSSFIGDMGKELDAKGCIFSEFGTRRRRGFKLHDKVVEVLHELDSFSGTSNVYFAKKYGVRPIGTIGHEWIMGNSALVGLRNANKFAFDNWVKVYKGDLSIALSDTFGSKPFFQNFSIGLAKIYDGVRHDSGDPIKFADEVIEHYKKLGIDPMKKVLVFSDSLHVSDAVKLKEYCSGRINCSFGIGTTLTNNPDFFSYNPPLNMVIKLHKIDGIPVVKLSDSVEKATGDKDALRVANYIFGRKGLDEQF

>sp_Q484J7

MIAEQHQAVVDSGASKKGAKVGFVSLGCPKNLVDSERILTQLRTEGYDVTNSYDDAELVIVNTCGFIDSAVQESLDTIGEALAANGKVLVTGCLGVKKDEIIELHPNVLGVTGPHAYDEVLAQVHEHVAKPEHNPFIDLVPPQGVKLTPKHYAYLKISEGCNHRCTFCIIPSMRGDLDSRPVGDVLGEAKRLVDSGVKELLVISQDTSAYGVDVKHKTDFWDGMPVKTHMQQLCEELAKQGVWIRLHYVYPYPHVDKIIPLMAEGKILPYLDIPFQHANKRILKLMKRPGSSDRVLERIAKWREICPELVIRSTFIVGFPGETEEEFEELLNFLEEAQLDRVGCFKYSPVEGATANALPDHVSDEVMEDRLQRFMAVQAKISSDKLQVRIGQEYLILVDEVNGLGIVGRSYMDAPEVDGKVYLSDDYDAKPGDQIWVQIIHADEHDVWGVRVED

>sp_Q12YR1

MTRVLATGTFDLLHPGHVFFLRQARSFGDELYVLVARDSMIKHKAQPIVPEGQRLKMISAFGVVDKALLGSESDIFEPLKEINPDIIVLGHDQFFDTGELEKNLGERGFKAKVVRIDDAMKCELCSSGRIIKRVLERYGQNEE

>sp_Q47WT4

MFETNPILSKLKEIRERANLLRGYLDYDVKAERLVEVSRELELPDVWNEPERAQALGKERSSLEEVVNTIVELETGCEDIEGLVELAVEESDQETFDDAEVEADALDKVLEKLEFRRMFSGEQDANNSYLDIQSGSGGTEAQDWAEMLMRMYLRWGEAHGYKTEVIEVTDGDVAGIKGCTIKYTGEYAYGWLRTETGVHRLVRKSPFDSSGRRHTSFASAFIYPEIDDNIEIDINPADLRIDTFRASGAGGQHVNKTDSAIRITHEPTGAVVACQADRSQHKNRATAMKLLKAKLYEMEMQKQNSDKQVLEDGKSDIGWGSQIRSYVLDDSRIKDLRTGVENRNTQAVLDGDLDKFLEASLKSGL

>sp_Q47WQ1

MANYFKAAVKPKTSNQRLTVTVDKLDMNGVGVARWQNKPIFIAGVLPDEIVDVKVIEQKSKYARAKLISIDKQSASRVIPQCQHFGLCGGCDLQMLALEEQLLFKQQKITDLFSRSFSTQNITPEINTAHLPWQAAIKSSPWHYRRKARIGVQFDKNAQATIGFRQKSTNQLAAIKSCPVLVEPLSAIFPLLKKLLAQLTVKSAIGHIEVIQADISDTSSADKIQKDNQVVVVVRQLKPMNDTDIGLWQLYAQRHCWHVIIDDGNKQLPLADIKGGDSFELSYELTDTSKVYFSSNDFIQINHQVNNAMISQALAWLNILATDNVLDLFCGLGNFSLALAKHAKRVVGVEGMQTMVDKATQNSLVNGLDNCQFYQADLNSHWLLEPWVQGQVFDKVLLDPARAGAEQAVSQIAELKIPCVLYVSCDPATLARDSAILVSKGYKLEKISLMDMFSQTKHVETMILFTHTS

>sp_Q9S0L2

MKASLQLKMGQQLTMTPQLQQAIRLLQLSSLELQQEIQQALDSNPLLELDEEQVDPPVNGEDKTVDTEDFSATAESDGPIDNSAVETSEAITRDSMPEELPMDTTWDEVYTASPNSTSGAMRDDDMPFQGETSEGLYEHLEWQKNLTPFSDNDLAIATAIIEAIDERGYLTQSIEDILEAMGDPEIEQDEIEAVLKRIQHFDPIGIAARDLSECLLIQLAQYADTTPHIDNARILIRDHLDLIAGRDFRLLMRKTKLKEDALRDAIELIQTLNPRPGLAVTPGKDEYVIPDVTVTKKKGRWMVELNPDNMPKISVNQHYASMAKSTKSQADSQFIRGHLQEAKWFIKSLESRNETLLKVSKCIVKFQQGFFEFGEEAMKPMVLNDIAEAVEMHESTISRVTTQKYMHTPRGIFELKYFFSSHVATDDGGECSSTAIRAFIKKLVAAENQQKPLSDSKMALLLADQGIKVARRTIAKYREAMLIPPSNQRKSL

>sp_Q12ZB4

MDYKCTRCKRPVEIDYGYTGIRCPYCGHRILVKERPQSSIKKVKVE

>sp_Q12ZG3

MGNIRQTHIKNIAFRLVENYGDVFTTDFDKNKLLVSQYTTIEGKVIRNRVAGYVTRKIRYPKLI

>sp_Q486U3

MANSKQAKKRAGQSEKRRQHNASRRSMMRTLVKKVLAAIEAGDKEVATKELAAATPTLDRYASKGLIHKNKAARSKSRLNAAIKAL

>sp_Q489T9

MSRFFRRRKFCRFTAEGATSIDYKDIATLKNYITESGKIVPSRITGTAAKYQRQLTRAIKRARYLSLLPYTDLHK

>sp_Q47WC1

MAKQKALQAIRGMNDCLPSETNIWQMVETVLRRVASNYGFAEIRMPIVESTALFKRGIGEVTDIVEKEMYTFDDLNGDSLTLRPEGTASCVRAGNQHGLLYNQEQRLWYMGPMFRHERPQKGRYRQFHQFGLEAFGIATPDIDAEIILLTSRLWRELGINEFVTLELNSLGSNEERANYREALIAYLLEHEELLDEDSKRRMHTNPLRVLDSKNPQVQEALTNAPKLSDHFGEETQTHFDTLCARLDAAGINYVLNERLVRGLDYYNRTVFEWVTTTLGAQGTICAGGRYDGLVEQLGGKATPAFGFALGIERLVLMLTELEKVTNIRPQVDAYVVILGDDAQVAANKLAEQWRDQVPEIRLQCHCGGGNMKKQLKRADKSGAQIALILGDDEITQEKVMVKYLRGQKEQESIEFTQVSSLLSELI

>sp_Q47Z50

MRTSQYLLSTLKETPANAEVISHQLMLRAGLVRNLASGLYTWLPTGLRVLKKVEQIVREEMQRAGGNEILMPMVQPADLWQESGRLDDYGPELLRINDRHKRPFVLGPTHEEVVTKLVANELSSYKQLPLNVFQIQSKFRDEIRPRFGVMRGREFLMKDAYSFHLEDECLEKTYQIMFDAYCRIFERLELNFRPVLADTGSIGGEKSHEFHVLADSGEDDIAFSDASDFAANIEKAEALAPAGERAEPTQTLTKVATPNVKSMDDLVQCLSVDLKTTVKTLLVVGATVEGEAETVVALVLRGDHQLNEIKAEHLPQVATPITFASEEQILAAANCNAGSIGPVGLNIEVIVDRSAAHLSDFVCGANEDDAHLTGVNWQRDCNEISVHDIRNVVAGDPSPCGQGNIEIKRGIEVGHIFQLGRKYAEAMNCAVLNEGGKNQTLTMGCYGIGVSRIVAAAIEQNHDKYGIKWPKAIAPFQVAIVPMNMAKSARVKETAEALYESLNQAGIEVLFDDRKERPGVMFADHELMGTPLLLIIGERNLDAQQIELKNRITGEKSLIAIDEVMSLFN

>sp_Q48AG8

MKYSDLRDFISQLEKIGQLKRITQPISTHLTMTEISDRTLRAKGPALLFENAVSESGEPYNMPVLTNLFGTPDRVALAMGQKDVGALRDVGKLLAMLKEPEPPKGFRDALGKIPVYKQVLNMPVKVIKKPLCQQIVLSGDDVDLTKMPIQSCWPGDVAPLITWGLTVTRGPHKERQNLGIYRQQVLSKNKVIMRWLSHRGGALDFQEFKKENPGEKYPVSVALGADPATILGAVTPVPDTLSEYAFAGLLRGAKTEVAKSISNDLEVPATAEIILEGYLDPEEMAPEGPYGDHTGYYNEVDNFPVMTVTHITMRKDAIYHSTYTGRPPDEPAILGVALNEVFVPILQKQFPEIQDFYLPPEGCSYRLAVVTIKKQYAGHAKRVMMGVWSFLRQFMYTKFVIVCDDDINARDWEDVIWAMTTRMDPSRDTVLIENTPIDYLDFASPVSGLGSKMGMDATNKWPGETNREWGEPIEMTQEIKNQVDELWDELDIL

>sp_Q12VJ6

MISKQQISEIISNYDLNDLAIATVCSHSSLQIFDGARKEGLRTIGICVGQPPRFYDAFPKAKPDEYIVVESYSDIPKIAEELVRKNAIVIPHGSFVEYMGTESFAELAVPTFGNREVLEWESDRDKEREWLEGAGIHMPKIVDPEKIESPVMVKYHGAKGGRGFFIAKDYEEFKQYIDPNEKHTVQEFIVGTRYYLHFFYSPIREEGYKLSEGILEMLSMDRRVESNADEIFRLGSPKELEDAGIHPTYVVTGNVPLVARESLLPRIFALGEKVVEESLGLFGGMIGPFCLETVFTDKLEIKVFEISARIVAGTNLYTSGSPYSDMIEENLSTGKRIAQEIKLGAKTGKLDLILS

>sp_Q12WT4

MAQKARIRLSGTSPVNLDGVCDQVKAIANRTGVSISGPVPLPTKKLVVPVRKSPSGDGTATWDHWEMRVHKRLIDIAADERALRQLMRIQVPKDISIEIVLQN

>sp_Q487Z5

MINEERLLKVLLAPNISEKATTAAEANNTVVFKVATDATKAEIKAAVEKLFEVTVEGVNTLNVKGKVKRTGARFGRRNDWKKAYVTLAEGSDIDFVGAES

>sp_Q12ZU9

MNAIRYPFITEKAMMLMDENKLQFVVDTRANKTQVENDVVKMYGFTVKSVCTMTTMKGLKKALVTFNETDAAHEIATRIGLV

>sp_Q12ZU8

MAKRIISQNRGRGSPTYRAPSHKYKAELKHPRVDEESTLNGTVIGIEHDPARSAPIAMVAFENGKKQFIVVPEGISVGEKLSCGVSAEVKPGNTLPLAEIPEGIPICNIESKPNDGGQYARSSGVYATLVSRELSKVVVRMPSGVLKWFHPKCRATIGIVAGGGRVDRPFLKAGKKYHKMKARAAKYPRVSGIAMNVVDHPFGGGNRKHPGKPTTVSRNAPPGRKVGHIAARRTGKR

>sp_Q485H0

MSNVSMRDMLKAGVHFGHKTRYWNPKMKQFIFGARDKVHIINLEQTVPMFNEALAFVNNVSSKKGKVLFVGTKRAASDAIKDAAIKSDQFYVNHRWLGGMLTNWKTVRQSIKRLKDLESQSTDGTFEALTKKEALMRTREMEKLDKSLGGIKNMGGLPDVLFIIDADHEHIAIKEANNLGIPVISVVDTNSNPDGVDYVVPGNDDAIRAVTLYCDAVANSVLSGREQNIVVQAEKDGFVEAE

>sp_Q47Z58

MTLTTRFAPSPTGYLHVGGARTALYSWLYAKKNGGDFILRIEDTDLERSTQASVDAIMDGMNWLNLEWTHGPYFQTERFDRYNEAIEQLIASGNAYRCYSTSEEVDAMREEAKAKGEIEKYNGLWRDRTDHPADKPFVIRFKNPLEGDVIIKDMVKGDIAISNGQLDDLIIARSDGTPTYNLTVVVDDWDMKVSHVVRGDDHVSNTPKQINILRALGADVPQYAHIPMILGDDGKRLSKRHGAVGVMQYRDDGFLPEALLNYLVRLGWSHGDQEIFSREEMIELFDLKDCNRAPSGFNTDKLIWVNQHYMKTMDPAYVAEHLAWHMADQGINTENGPALADVVRIQADRVKTLKEMADISRYFYEDFTELDAKAVKKHLRPVVKEPMILVKEKLAALTDWSPEPIHAAINDTAVELELGMGKVGMPLRVAATGGGNSPSLDITLALLDQSKVIERIEQALVVVEARIAAG

>sp_Q12U86

MAQKKKSSGSGLMSSAGLMTYYDADKKAIHVQPKTVFIFGAICGIVILAFSAGFGLWP

>sp_Q12Z63

MQKIVILRLGHRPERDKRITTHVGLTARALGAEGMLLASNDKGIKNAIEDVAERWGGDFYVENDVNWKSEIEKWKEEGGKVCHLSMYGINLPDAAGEIKLCDKLMIVVGAEKVPTEIYDLADWNVAIGNQPHSEVAAVALTMDRIAQEEPLKREFGYAELTIVPMERGKRVINNVKEE

>sp_Q12ZB1

MSSEIPPQVQNQLAQLQQVQQQAQALAMQKNQMESMKKESEMALEELEKLSDDAIVYRAVGDLQIQSNKDDTVAKLKERLETLSLRLQSITRQEERISKRFTQLQEQLKQAMGTQGQ

>sp_Q12TF9

MATDIEQKVMEAKMASIVLASVDTQTKDNALEAMAKALDANRNKILEANKADLEEAERMKNEGKLSQALVDRLKVTDPKIDGMISGIRDVIKLEDPSGRTINTLELDKGLELYQVSSPIGLIGVIFESRPDVVPQVMSLCLKSGNATVFKGGSEALNSNRVIFNILVEALEDTPGIPKGAFQLMETREEVMDILALDEYIDLLIPRGSNDFVKFIQDNTKISVLGHADGICHVYVDTNADLNKAYDVCFDSKVQYPAVCNAMETLLINREIAEEFLPEMVRRYEEVGVELRFDEGSYAIAEKLGSANIAKATEDDWKTEYNDFILSIKLVDSIEEAIDHINKYGSHHTDAIITENKTKRKQFIALVDSSSVMVNASTRFADGFRYGKGAEVGISTNKIHARGPVGMEGLVIYKYVLLGNGDKVATYAGDTPRPFTHKELDSKLSDIINE

>sp_Q12V34

MKNIEELILKAVELQSNGLVTGQIANELNVSRETVTWLLTRSKKDVVAPAPKDISVTWNSVGQSSYRLRCISQALCDMVIEKLERTQQDADLVIGIGLSGIPIATMMAEELEIDFAIFHDYDDQKGKTNQRGIFSRNFADVEGKKCIIVDDVVSSGATVTDVAEQLREVGATPIAVAVIVDKMNADMIANVPMSSLVRITRVD

>sp_Q47WP5

MKINPHNLARLTKKLGYEFNEPLLLVQALTHRSAKGAHNERLEFLGDSILGFVIAEALYDKFPKHDEGDLTRMRSSLVKGVTLAEVARDFNLGECLILGPGELKSGGHHRESILEDAIEAIIGAVYLDSNIECCKALILSWFERRLMVIKPGNEQKDPKTRLQEFLQGRKIPLPTYEVIDTTGQSHNQEFTVRCQTSVISEVVIAKGTSRRKAEQEAAQQILALIEKEREQEKEVKIKPTKQAKLANPRHTKSNPSSSSKKSSTRK

>sp_Q12U25

MLEDEYQIDFFSDNGFVRKQCPTCGNFFWTRDIERSTCGDAPCDPYSFIGNPVFKKELELPDMREFYLNFFEEQGHTRIERYPVIARWRDDIYLTIASIADFQPFVTSGQVPPPANPLTISQPCIRLSDLDAVGKSGRHLTTFEMMAHHAFNTKNEEIYWKEHTLELCDGLLNSLGADPMAVTYKEEPWAGGGNAGACVEVLIGGLEVATLVFMNLKKDKNGDIDIKGDMYSKMENYIVDTGYGLERLVWASKGSPTIYDAIFPSIVNELMGLAGIEHELENNEYSHILSQNARLAGLMDISEKANLLELRKQVAASIGITADKLSSIMEPVENVYAIADHTRCLTFMIGDGIIPSNVKAGYLARLVIRRTLRMMKDLGIMIPISEIIQMHINNLPEYPEFQKRFDVIKDILEHEERKFAETLERGRRMMEKSARHYKESGEKMPLETIIDMYDSHGIPPEISKAVASDVGVEVDLPDNFYSLVADKHSQSEEKEEKVVPFADKIARLPKTKRLFYDEPNRMDFDAVVLEVFDNHIVLDNTLMYPEGGGQPADHGTLTVEDVVLKVVDTQMYDGVVVHTINEIEDELHIRKGDMVVGRVNEKRRMAHARHHTATHIINDAAREVLGSHIWQTGAQKFADRARLDISHYKRITQEEANQIEIIANHTVMKNKRIISDWMDRTEAEQKYGFRLYQGGVPPGKMIRVLQVGNDIEACAGTHCTNTGLVGPIKILKTERIQDGVERLEYAAGEAAIIAMQDIETLVRDSSETLRVSAEQLPSTIERFFDEWKELKKENNKLKEELAHSRVSQLVNDAEDVNGIRIITKAIPHADSEELTKTAGELTQESNVVAILISEMDGVKIVATAGDDAVKRGVNVGAIVKEMSTMVGGGGGGRPNMARGGGTDPSGMDNALSRSVELLKEQLN

>sp_Q47VL4

MKFVDEVEIRVEAGDGGNGCVSFRKEKFIEYGGPNGGDGGDGGDVYLMADEGLNTLIDYRFERFHRAKRGQNGQPQNCTGKGSEDLVLKVPVGTRAVDQDTGEQIGDLTYKGQKMLVAKGGWHGLGNLRFKSSTNRSPRQRTDGTPGEIRSLKLELLLLADVGLLGLPNAGKSTLIRSVSAATPKVADYPFTTLVPNLGVVRLDTQRSFVIADIPGIIEGAADGAGLGTQFLKHLERCRILLHVIDIMPVDGSDPLENAKVIISELEQHNEKLAGKPRWVVFNKLDLVLEEEAKEITDAIIAGLDWKGEVHSISAFNRSGTKELTQKVMTFIEELPPEEEEVIDGKTVEFKWDTYHEETIAAHSQDDDLDDDDWDEDDYDVEVEYRQ

>sp_Q487E5

MSSRIGGKSKRSGELLAQKLQSEGISNPAVLKAIAHSPRHIFVPEILAHKAYDNTALPIGQGQTISQPYIVAKMSELLLADGRPQNILEIGTGSGYQTAILAQLTDKVFSVERIKALQWQAKRCLRAMDLHNVAMKHGDGWQGWRSKGPFDAIIVTAAPSSVPPALLDQLADGGRLVIPVGEQTQILKIITREGDVYNEQQVEAVRFVPLVPGDLL

>sp_Q12WH8

MVEYKQCIIIRDDLKLSKGKLAVQVAHAAVSAAEWASRSDLENWKEGGQKKVVLRVEKLQDLFELKEKARREGLSTALITDAGLTEIAPGTVTVLGIGPARADYIDKVTGNLKLV

>sp_Q12TI6

MTVIDANGLIMGRLASNVAKMLLSGEEVSIVNAERAVISGSKVTTFEEYDVIRNMGTREFGPYFPRRPDRILKRTVRGMLPYKRARGKDAMGRLKVYVGIPYEYQDAEFVSVEGAEMTRLSSNKYVTIGDVSRQLGSKF

>sp_Q12U31

MSKGTPSMGKRQKRTHAKCRRCGSVSLNIHTKQCTSCGFGRTSRMRSYQWQRKCKF

>sp_Q487J9

MIDEELQQWFSHTEMLIAELLEDGTNDEVYHTIEHHFASSDFDLLEKAAIAAFKLGLEIEEPEEAELENGDKVFAFDIATEQMLDVNLIKKETQAMFELAKQCGVDYDGWGTYFEE

>sp_Q487Z7

MPRSLKKGPFIDLHLLTKVEKAVESGNKKPIKTWSRRSMIIPTMIGLTIAVHNGRQHVPVFVTEEMIGHKLGEFAPTRTYRGHVADKKAKK

>sp_Q487B1

MNIKNILSERVSAAMVAAGLPEGTNPAISLSNRPQFGDYQANGVMGAAKKLKTNPRELATKVVAELDLDGIASKIELAGPGFINIHLDESWLAAQLNEVAQDDFIGVAQRGTTPGDESQTVVVDYSAPNLAKEMHVGHLRSTIIGDAVVRALEFRGDKVIRQNHMGDWGTQFGMLIAHLSDKLASDEVAETALADLENFYREAKVRFDNEEGFADRARADVVKLQSGDEACAKLWQQFIDISITHSEEIYAKLNVSLKRSDIMGESAYNDDLSTVVDELMAKKIAEESQGAKVVFINEMANKDGEAPVFIVQKSGGGFLYATTDLSACRYRSGKLAANRIIIFTDARQALHFKQVEIVARKAGLLPENVGYQHCPFGMMMGDDGKPFKTRTGGTIKLAELLDESIVRAAALIKEKNPDITDTDLTEISKKVGIGAVKFADLSKNRTSDYIFDWKTMLSFEGATAPYLQYAYSRIQSIFSKAGTVKNDAVINIIEPQEKALALKLLQLEDVVDAVISECTPNLLCNYLYELASLYMSFYEACPILKEGISGEVKASRLALCNVVADTLKQGLDILGIETMERM

>sp_Q480H5

MSDTTLSEATSNTASHSLAEKRKILVTCALPYANGSIHLGHMLEHIQTDIWVRFQRMRGHETYFVCADDAHGTPIMLKAQELGITPEEMINGVREERIKEFSDFHISFDNYHTTHSDENKEYSEKIYNALHAKGHIKTRIISQLYDPEKGMFLADRFVKGTCPKCKSEDENGDSCDNCGATYSPTEVLNPRSAISGATPILKDSEHYFFDLPAFETMLSDWIRSGALQEEVANKLTEWFEQGLKQWDISRDAPYFGFEIPNAPGKFFYVWLDAPIGYMGSFKNLCDKDSTIDFDSFWNKNSDAELYHFIGKDIINFHSLFWPAMLEGADFRKPTAVFAHGFVTVNGEKMSKSKGTFIKGRTYLDHLNPEYLRYYYATKLTHKIDDLDLNLEDFVQRVNSDLVGKVVNIASRCASFITKRFDGMLSTNIDDQALADEVMAAGDSIAAHYESRDFGRGMREIMALADKVNEYIAIKEPWQLVKDETKQQEVQDICSLGINMFRTLMIYLKPVLPVLADSTAAFLNDELVWEGHKTLLTDHKINKFKALLQRVDMDKVNAMTDASKDSLGAPVEEEKKPAKKKKAAKVVDNSAALADPLAADPISEEIEFDDFAKIDLRIVKIINAEHVEKADKLIQLTLALNEEGTETRQVFAGIKSAYNPEDLIGKHTVMVANLAPRKMRFGMSEGMVLAAGPGDKDLWILNPDDGAKAGMRVK

>sp_Q12UC1

MLSLSEAIILGIVQGLAEWLPISSEGMTSLVMVTFFGRSLSEAIPISIWLHLGTLLAAIVYFREDVKVLLYGVPDYVRSFSRKQPHDPVISFLLISTALTGIVGLPLLLFVTDNVEISGGSATAVIGIMLIVTGILQRTVSRDESLSRVPGMSDSLVSGVAQGFAAIPGISRSGITMSALLLRKFDAADAIRLSFLMSIPAVLVAEIGVGLMGMVELDINSIVGLFFAFAFGLVTIDLFLKVAKKVDFSYFCIGLGVLSVLTMFL

>sp_Q47Y94

MKSSVYQKLEVLVERFEEVQALLSDPATISDQEKFRALSKEFKQLDAVTSVFNNYKSAEDDFSTAELMLKDDDPDMREMAQEEFKDAKKAVADIADELQILLLPRDPNDDNNCFVEIRAGAGGDEAAIFAGDLFRMYSRYAEKKGWKIEVMNSNESEQGGYKELIMKVNGEGVFGHMKFESGGHRVQRVPATESQGRVHTSACTVVVMPEIPEADAIEINKADLKVDTFRASGAGGQHVNKTDSAIRITHIPTGVVVECQEQRSQHKNRAQAMSVLQARLQQAEDEKRRSEEESSRRNLVASGDRSERIRTYNFPQGRMSDHRINLTLYRLNEIMEGSLQLVMEPIMQENQADLLAELAEQH

>sp_Q47WU9

MSTEEKKIILGKVGAVYGIKGWLKIHSFTDETEAILDYFPWSLKLGNNTQTVEITDWRKHNKVLIVKVAGIDDRDEAQALVGSEILTNEAALPELSEDDFYWRDLIGMSVVTNKGYDLGVVTDMMETGANDVLVVKANLKDGFSKKERLIPYLFEQVIESVSIENKQICVDWDPGF

>sp_Q12UQ0

MASVVEALIPGGKANPGPPLGPALGPLGVNIKDVIEKINEKTKDYNGMQVPVKVIVNDDKSVEIEVGTPPTSALILKELNIEKGSGESGTVVVGNLEIAQVAKIARMKKDDILSYSLKAAIKEVMGTCVPMGVTIENLDPRECQKAVDEGKFDESLTAEAW

>sp_Q12ZT2

MATGPRYKVAFRRRREGRTDYHQRLRLLLSREDRVVVRKSARHMQIQLVAPDANGDVTLSSAISKELAKYGYEGSTGNTTAAYLTGLLFGYKTLAEGYESGVLDIGIQASSAGSRVYAALKGVVDSGLDVPHNSSVFPSDERIRGEHVAEYMEGSNLPEVFDAVKEKILAEFS

>sp_Q12XH4

MPTSHGERSCTRYKLKKTVRERGLSPISKAIQDFEEGQMVHIDIDPSVQKGMPNAKFQGKTGKVLGKRGRAYLLQVTDGNSKKEVISLSQHLKPQKY

>sp_Q9S0Q8

MQGSVTEFLRPRLVDIEQVNPTRAKVTLEPLERGFGHTLGNALRRILLSSMPGCAVTEVEIDGVLHEYSSKEGVQEDILEILLNLKGLAVVIEGKDEAMLTLSKSGAGPVTAADITHDGDVTIMNPEHVVCHLTGNNEISMRIRVERGRGYVPASARAQTEDDDRPIGRLLVDASFSPVARIAYNVEAARVEQRTDLDKLVIDMTTNGTLDPEEAIRRAATILAEQLDAFVELRDVTEPEQKEEKPEFDPILLRPVDDLELTVRSANCLKAEAIHYIGDLVQRTEVELLKTPNLGKKSLTEIKDVLASRGLSLGMRLENWPPASLVDDL

>sp_Q47UB2

MARVTVEDAVDKVGNRFDLVLVASRRARQIATGGKDPLVDVENDKPTVIALREIEAGLITTDIMNTSDRAQQIQQDTAELDAVAAIVGGQQEELS

>sp_Q12ZU2

MAKDIGLDIPEPTKECDDINCPFHGELPVRGQIHVGTVVSAKMDRTVVIQQRREKLINKYQRYEKRQSKIHAHNPPCIDAKVGDIVTIAECRPLSKTKSYVVVKAEVKA

>sp_Q488J7

MTSIRLTQYSHGAGCGCKISPSVLDVMLKSSLALPVNDALLVGNSTKDDAAVFDIGNDQGVISTTDFFMPIVDDPTDFGKIAACNAISDIYAMGGKPIMAIAILGWPVNLLAPEIAQQVLDGARAICAEAGIPLAGGHSIDAPEPIFGLAVTGLINNAHIKRNNTAEVGDLLYLTKPLGIGIMTTAEKQGKLLPEHAQLAPQAMKTLNVIGQKFAELEVVTAMTDVTGFALLGHLLEMCQGSGVAAVIDFEQVPRLDFVNDYIDQGCVPGGCERNFISYGEHVGPLSPKQKILLCDPQTSGGLLVAVKPQGKEAFEALCQENGLNLQPIGELVAATSPTVSLLS

>sp_Q48A12

MFSHIHPDNYDAQLSKKQQDMAKLFSDFNLPAPDLYPSVPLNYRQRAEFRVWHEGDDLYYIMFDSKTKEKFRVDDFPVASELINNAMKALLATIKDQRELRFKLFQVDFLSTLSGELLISMLYHKPLEDNWQIEAEKLKAQLSTIAPVDIIGRAKKQKIIVDKDYVMESLNVGGKTYVYQQVENSFTQPNAGVNEQMLLWAQQATQNAGGDLIELYCGNGNFSIALAENFERVLGTEISKTSVRSAQINISENGIDNIDIVRMSSEEFSQAMNGERKFRRLEDFDLTTYNYDTVLVDPPRAGLDRDSVELVRRFNKIIYISCNPETLKDNLALLVETHQIDKFALFDQFPYTDHIETGVILTRK

>sp_Q47VZ4

MAGNDSNLIWLDLEMTGLEPVEDVILEIAIIITDSELNILAQGPIFAISQTDDVLDNMNPWCIEHHGKSGLTQRCRDSEVSLAHATKESLAFVQEWVPQGKSPMCGNSIGQDRRFINKYMPDFEDHFHYRNLDVSTIKELAKRWKPEVLESVVKTGAHLALDDIKESIAELKVYRELFFKL

>sp_Q47Y12

MISISPKALAPGSDKAIEKKLDLTNLTPEQVALDINKLNLFYGKKQALNNITMSIPKGQVTAFIGPSGCGKSTLLRSINRMNDLVDNCHISGEINLHGSNIYDKHVDVAELRRKVGMVFQRPNPFPKTIYENVVYGLRIMGENNRRRLDEAAEQSLRSAALWNEVKDRLHESALGLSGGQQQRLVIARAIAIQPEVLLLDEPTSALDPISTLTIEELINDLKKQFTVVIVTHNMQQAARVSDQTAFMYMGDLIEYSDTNTLFTTPLKKKTEDYITGRYG

>sp_Q48AM4

MKSLAKLKAEPGIWLTRTEKPKLGHNDLLIKIKKTAICGTDIHIYNWDEWAQKTVPTPMVVGHEYAGEVVGIGQEVKGFTLGDRVSGEGHITCGHCRNCRGGRTHLCRNTVGVGVNRAGSFAEYLVIPAYNAFKLPDEISDDLASIFDPFGNAVHTALSFDLVGEDVLITGAGPIGIMAAAVAKHVGARHVVITDINEYRLDLARKMGATRAVDVSKESLKDVMTDLGMTEGFDVGMEMSGVPMAFTSMLESMNNGGKIAMLGIPGSDMAIDWSQVIFKGLTIKGIYGREMFETWYKMASLIQSGLDLTPIITHHYNIDDFQQGFDMMRSGQSGKVILDWT

>sp_Q487E6

MKILLSNDDGVHALGIKVLFDELVKHFSVNVVAPDRNCSGASNSLTLLNPLRAEHLDNGFISVNGTPTDSVHLGSSQLFTDCDLVVAGINKGANLGDDTLYSGTVAAATEGRHMGMPAVAVSLAGNNEQHYQTAAIVTAKIIKRLRTHPLPADQILNINVPDIPLAELKGIKVTRLGHRHQAERMQKMQDPWQRDIYWYGVLGQELDGGEGTDFHAIANGYASVTPLTVDMTAHRSIENIKSWLTALNLSD

>sp_Q12WA2

MQQDYNSSNIEQKWQQKWNESKVFEAEADDRDKYFITIPYPYLNGNLHAGHTRTFTIGDVVARYKRMMGNNVLYPMGFHVTGTPIVGLAELIQNRDPETMKVYTEFHGIPVETLKGMDTPEKIVDYFSVEAERSMRSIGYSIDWRRKFTTTDPNYKKFIEWQFNLLYEKDLIVKGSHPVKWCPNDDNPVEDHDILHGEEATIIDYTLVKFKYDGMIIPCATLRPETVFGVTNLWINPDLEHVKIKVTFEGREEVWVVSKEAYRKLIFTDREVEFIEDVDASSLIGIKVTNPLNDAQVITLPASFVKGENGSGIVMSVPSHAPYDYLALRDLYDKDLREYGITEDLRELKFISLIKVKEFGEFPAIEAVEQFGVKDQDDPKAEEATKIVYRREFHGGVLKENTGKYSGMAVSKIKDVLTRDLIEMGIGEVFYEFSEPVVCRCGTPCVVNMVKGQWFLNYSNPEWKDKVYRCIENMDIIPEDLRVEFNNKVDWLKDKACARKKGLGTLLPFDNQWLIESLGDSTIYMSYYIIAKFIAMGIETEQLVPELFDHVLLKKCSLETAAERSGIDANIIEQISSDFEYWYPVDLRSSGKDLIPNHLLFFLFHHVAIFDEDKWPRAIAINGFVSLEGKKMSKSKGPLLTLNDAITNYGADISRMYILSSAEQMQDADWKNSGIETARKQIERFYNFSKDIIGSGIPTCNVENLKGIDKWMLSRLQQRILETNEALDTIRTRNALQNAYFLLFNDIRWYQKRGGNALLCEVLDVWIRLMAPFTPHICEEIWEAIGHTDNDLISLADYPQYDESLVDTQAEFTEELIGGTLSDVDEIIRVTKLTPKKAILYTSPEWKMETFKKALSMQKEGNLNPGILIKDLMRDPEMRSHGKEVPKFAQKVVSDITAMNEEKFDTLSNFDLDEKIALEENLEFFKNELGCSVEIYSADNAEYDPENKARFAYPLRPAIYLE

>sp_Q12XH2

MRNFVIIAHKALTTGDFSLNDLPGSAGRMDILCRCINSCLFLSHDLRRDVQVHLLLLGEPEPGKIIRFDSEHVRYLNPDERSAGSLIKKALQKTAGEYEVRSTPGVFIRSGNLGTLLNEFKDAGRRLIYLHEDGEDIRELSDLTNNAVFILGDHMGVTEEEEQLIKEHEAKTISLGPIPLHADHCIILINNEIDRNLSGKSQ

>sp_Q12X37

MNVLIIGGGGRENAIADAIARSERNPALFAVMAKKNPGIAALCEDFLLAKETDVEKVVGYAREKGIEMVFIGPEAPLAVGLADALEDAGIGAVGPRKNVARIEFDKAWARNFMKDNDIEGSPAFKVFSDKEGLQEYIEELGSVAIKPAGLTGGKGVKVMGDQLPDTGAAYDYAVSLLDGDNVVVEENLVGEEFTVQAFVDGKNLAFTPCVQDHKRAFENDFGPNTGGMGSYSDSDGLLPFVTIDDLYHAREIMKATITALGATETPFKGMLYGQFILTKNGPKVIEFNARFGDPEAMNVLPLLKTDMIDVMSAVVNGTLDELDVEFLKRATVCKYAVPAGYPDEPSKDKEVVVGNIGDALLFYSSVYEKDGKVYTTSSRAVAVVGVANSITDAEVIAQNALENITGDLHFRRDIGTPELVQRRVTHMEQIRR

>sp_Q488Y9

MARYLGPKLKLSRREGTDLFLKSGVRAIDTKCKIETIPGQHGARRGRLSDYGVQLREKQKVRRIFGVLEKQFSNYYKEAARQKGNTGENLLQLLETRLDNVVYRMGYASTRAEARQLVSHKAIVVNGVVVNIPSFTVKAEDTVSVREKSKTQARIIAALELADQREKPLWVEVDTKKLEGVFKRVPDRADLSAEINEQLIVELYSK

>sp_Q12TT3

MDINIIEDKNNALLNRHEVKFDATFKGSTPSRLDVRGKLAAMLNVPLELVILQKFENTYGMSAANGYAKIYEDAARMKVVEKEYVLKRNELPEAEVVEEAGE

>sp_Q12TN7

MTLSDEDKITIEKFALQNAVKYGKAPQLGAVMGKVMGLCPHLRPLSKEVGPVIQHVLDEVMKEVPEEWQARLEVIAPELIEELNTKKEPDKGLKPLDVKEGESVVMRFAPNPNGPPTLGSTRGIVVNSEYVKRYGGKFIIRFDDTDPQTKRPMLEAYDWYLEDCEWLDAKPDEVVIASDNMEVYYDYARQLLEMGHAYVCFCEGGDFKKFKDAKEPCPHRGHAPEVNLEHWDKMLAGEYEEKAAVVRIKTDIEHKDPAMRDFGAFRIVKTAHPRPEVDDKYVVWPLLDFEGAIEDHVLGMTHIIRGKDLMDSEKRQGYIYNYLGWEYPKTTHWGRVKMHEFGKFSTSGLRQSIEDGEYSGWDDPRLPTLRALRRRGIKPEAIRKFMIDMGVGETDVSLSMDTLYAENRKIIDTIANRYFFVWDPVELEVEDAVCCTVNPSLHPSEDRGVRCIDIGSKLLVCKSDVESAELGDMLRMKYLYNIEITSVSPLKCKCIGDSMEDAKSNKMRIIHWVPEDGIPVKVLAPQGEFIGMGEKQIVDELDNVVQFERFGFCRIDSVDDGVVAYFTHK

>sp_Q47U39

MTLSQQLSTLISKTQLVVSQEQIDLLIQYVELLNKWNKAYNLTSVRDPSEMLVKHIMDSLMVGEVLIGKNFIDVGTGPGLPGIPLAILYPERNFVLLDSLGKRITFLRQVVFQLKLSNVTPVKARVEEYQGEEPFDGVLSRAFSSLNDMVSWCKHLITTEQGRFFALKGQYPQDEISQLPENITLVDSHEIIVPDLVGERHVIVLKKLH

>sp_Q12W06

MENMDLIKRNVQEIVTEEELTKLLETKKHPSAYTGYEPSGKIHMGHVLTVNKLLDLQKAGFEITVLLADVHAYLNQKGTMDEVRKTADYNKKCFLALGLDPEMTNFVYGSDFQLSPEYMLNVLKLTQATSLNRAKRSMDEVGRKMEDPKVSQMVYPIMQAVDIALLGVDVAVGGIDQRKIHMLAREGLPGLGFKAPLCIHTPILLGLDGTKMSSSNENYISVDDDEAALKKKFKKAFCPAEDIENNPVLELFKYHITPRYDEMVFERPEKFGGDLVCKSYAELEKVFADGSLHPMDLKNGAAKYLNEILEPVRSVLE

>sp_Q12VA6

MELNGVEIEDTFAEAFPIKISRILITAATKRWATVAAQEATGFGTSVIGCPAEAGIEKYADASETPDGRPGVYIQFCTFGFKSLEEQLLERVGQCILTAPTTAVFNGLPDAEKQFDTGRKLKYFADGTESETEVGGRKMHVIPMMEGDFLVEDTLGAVTAIAGGNFFIFGDTQMTTLTAAENAVDAIGAVDGTITPFPGGIVASGSKAGANKYKFLKATANEKFCPSIKDKVEGSEIPADVNCVYEIVINGLDFESIAKATEMGIRAAVAVPGIKKITAGNYGGSLGPHKFNLHDLF

>sp_Q12U08

MARYELIKDEIGSIAKYVPGRSIEDIVKNYGLEPSSVIKLGSNENPLGPSPKAVEALIANAQGISIYPSADARELVDAISEYTDIPAANIVASGPGMDGLLDGLARLVIANGDEVVITTPTFSYYEIAARANGATTVYVQREKDFSINVDKLLAALTPNTKMIFLCSPNNPTGNVIPEEDILKIATATDALVFVDEAYVEFAEKNIAHLVLQHDNIIVGRTFSKAFGLAGMRMGYGIMPEWLREEYMKIATPFNVSTAAMAAGIAALSDTEHLNKSIELTVKGKKFLQEELPFKVYDTQANFVLVDVAPHKARDVTTELLKKGIIVRDCTSFAHAGLSLIRVTIGTKEQNEKVVKAFSDI

>sp_Q486J6

MTSKTIVNSLAELEQTQDFIRRHIGPSESETQAMLNDLGVESVDALIDEIVPSDIRLADLPNVEESKTEVQALADLKAVASLNKVNDTYIGLGYFGTLTPNVILRNVLENPGWYTAYTPYQPEIAQGRLESLLNYQQMCIDLTGLELASASLLDEGTAAAEAMALAKRVSKNKKSNLFFISDDVYPQTIDVVKQRAEMFGFDIVVAPAADAAEHDIFGALIQYPGASGQVTDVSELIAKIHDNKGIVAVAADIMSLVLLKSPGELGADAVIGSSQRFGVPMGYGGPHAAFFTTLDKYKRSLPGRIIGVSKDTRGKNALRMAMQTREQHIRREKANSNVCTAQVLLANMAAFYAVYHGPQGLKTIANRIHRLADILCLGTATKGLTAVHANYFDTLTFNVDNKDEIVARALAANANFRTDVDGQISIALDETTTRENVAQLFDILLGEGHGLNVSDLDDQIVASGHSSIPASLVRESAILTHPVFNSYHSETEMLRYIKRLENKDLALNHSMISLGSCTMKLNATAQMIPVSWPEFANMHPFAPVNQAQGYKAMIDELAKWLVELTGYDKMSMQPNSGAQGEYAGLIAISKYHESRGDSHRNICLIPASAHGTNPASAMMVDMKIVIVACDKEGNVDMADLKAKAEELADNLACIMITYPSTHGVYETTIAEICNIIHDNGGQVYLDGANMNAQVGLTSPGFIGADVSHLNLHKTFAIPHGGGGPGMGPIGVKSHLAPFLPDHALINVDEATKGNGAVSSAPFGSASILPITYLYIALLGKKGVTDATKYAITNANYVSKKLSEHYPILYSGKNGRVAHECIVDLRPLKASSGVTEVDMAKRLMDYGFHSPTMSFPVAGTFMIEPTESESKVELDRFIEAMVCIRDEVRKVESGEWASDNNPLHNAPHTLADITEPWDRPYSIQEAVFPVVAVTANKFWPTVNRIDDVFGDRNLICSCPPIESYID

>sp_Q12VH2

MVYENPDGVMVGLEVHVQLNNLNTKMFCGCSTQYHDSEPNTHVCPVCMGLPGALPVINKRAVESAIKIGMALNCEVVEQTQFHRKNYYYPDLPKGFQTTQYDFPIVGNGKVVIEGEDGEHVVRITRAHMEEDPGKLVHIGSIDKSKGTLIDYNRSGMALIEIVSEPDMRSPKEARRYLDKLRNILDYLDVFNGDLEGSMRVDANVSVMGGQRAEVKNISSHKGAERAILYEIMRQKNLLRRGGEVVMETRHFDEARGVTISMRTKEGEHDYRYFPEPDLVPMRVSDWAPAIREELPELPDAKRARLISEYDITEMHAKALTSDIRVADFYEVVAAAVEPRVAGTWVADVLKGELNYRDLSVDSFTTDDIIQIIKLVVEDKVTEQSAVDVIRTILDDGGTPMEVVTEKGLLKVKGDVVTEAVSETIAENEAAVQDYLGGAEKSLNFLVGQVMKKTKGRADARQARELLVAALKS

>sp_Q9F1N2

MELDNSHLVLGLGATGLSVVRYLCRQGITPLVMDSRDQPPGAEQLALEFPEVNLITGGFDCRYLVQASQIVISPGIAIDTPEIRAAIDMDIEVIGDVELFARAIKDRSPCVIGITGSNGKSTVTTLVGEMAKAAGLNYAVGGNIGIPVLDLLQKPVDLYILELSSFQLETTHSLNCISATCLNISEDHMDRYSDLEAYRQAKLALYDQSKRALFNREDSLTQPNDPMNQNSFGLTSPVNDEWGVKDGKIVHGTTEIASLQDVAIVGSHNHANLIAAMALAYHAGIDKEPMIQVAKNFTGLAHRCELVANIAAVAYVNDSKATNVGATVAALEGLGEHLGDIILIVGGDGKGADFTPLETVFNKVAHLITLGKDGDKIAALKEHSHKADSMADAVKQAAELATAGDIVLLSPACASLDMYKNFMARGDDFRQLAQALSVETLDSEASADV

>sp_Q47XX7

MIKNLRGAPALSDFRVKKLLAQCEQLQLPVNDIYAEFAHFTKLNEELSTSEEKVLQQLLTYGPTIEEHQPAGLFLLVTPRPGTISPWSSKSTDIAHNCGLAKVERLERGIAYYVTLENDAQLSTSQEAQLNTLLHDRMMESIFNDFAQASTLFASSEPGELTAIDIESGGKNALVQANIELGLALAEDEVNYLFENFTKLGRNPHDIELYMFAQANSEHCRHKIFNAEWTIDGVKQEKSLFKMIRNTHEINPDYVLSAYKDNAAVMVGNKGGRFFPNPETNVYGYNHEDIQILMKVETHNHPTAISPYPGAATGSGGEIRDEGATGIGSKPKAGLVGFSVSNLRIPDFVQPWETDFGKPSRIVTAFDIMIEGPLGGAAFNNEFGRPAILGYFRTYEEEVNSFNGKEVRGYHKPIMLAGGLGNIRDEHVQKREIIVGANLIALGGPAMNIGLGGGAASSMASGQSAESLDFASVQRENPEMERRCQEVIDKCWQLGEENPIAFIHDVGAGGLSNAFPELVADGGRGGIFELRNVPNDERSMAPHEIWCNESQERYVIAVSDKNLATFEQICQRERAPYSVVGRATEEEHLTVTDSHFSDNEKLNTPIDLPLDVLLGKTPKIYKDVKTATAAGDSLDLSTVTLADAADRILSLPTVAEKTFLITIGDRSVTGMVNRDQMVGPWQVPVADCGVTASALDSYHGEAMSLGERTPVALLNFGASARLAVAESLTNIAGTDIGDLNRIKLSANWMSPAGHPGEDAGLYEAVKAIGEELCPALGLTIPVGKDSMSMKTQWEENGEQKSVTSPLSLVITAFGVVEDIRKTVTPELRTDKGDTRLVAIDLSKGKKRLGGSCLAQVYKQLGSETPDVDDAEVLKGFFNAMQTLVRAEKVIAYHDISDGGLFTTVTEMAFAGHTGVDIDISKLSNGANDDLATLFNEELGGVIQIRESDVDAIHAILAQHGILENCTDIGRLNNEDTIRFSRDGEVVLENSRTYYRTVWAQTTYRMQSLRDNPECAQQEHDVKFDTEDPGLNTELTFDINEDIVADLIIRDAVKDAENSANDITNPRVAILREQGVNSHVEMAAAFDRAGFVAIDVHMSDILSGRADLADFNGLVACGGFSYGDVLGAGEGWAKSILFNANARTMFKTFFEREDTFTLGVCNGCQMLSNLKDIIPGSEHWPHFVQNKSERFEARFSLVEIQESPSVLFKGMEGSRMPIAVSHGEGHAEFSSDAAIDAANNSGTVSMRYVNNYGDVTETYPANPNGSVDGITSLTTTDGRVTIMMPHPERVFRTVANSWHPDSWGEDSPWVRMFRNARAFIG

>sp_O93631

MYKLFGKWDLTEVEVADAGIKRYVNLDPVIVPHTSGKHARQQFNKSDITIVERLVNNVMRNAQNTGKKQIALRIVDEAFDIVNSKTKKNPVQVLVEAVSNAGPREEVVRLKYGGISVPKAVDTAPQRRVDHALRNISIGSNQTAFKSKRSAAECLASELIAASNRDAKCFSINRKDGKERVAKAAR

>sp_Q47WG1

MEKFNTHTGLVVPLDVANVDTDQIIPKQFLQKTERVGFGVHLFHDSRYLDHDGTQENPDFVINKPEYKGASILLAGENFGCGSSREHAPWALQEYGFKVIIASSFADIFYGNCINVGLLPIKLTEAEIEQLFKLSPNAQLTLTVDLPNNVVTCGELSFKFSLNEFQQYSLENGVDSVGWTLNKLDTIKAFEEKMPAWQ

>sp_Q47WN3

MKDDKEKALSAALAQIERQFGKGSIMKLGDNTTMDVETISTGSLGLDIALGAGGLPMGRVVEIYGPESSGKTTLTLEVIAEAQRNGKVCAFIDAEHALDPVYAEKLGVNIDELLISQPDTGEQALEIVDMLTRSGAIDVIVVDSVAALTPKAEIEGDMGDSHMGLQARMLSQAMRKLTGNLKKSNTMLIFINQIRMKIGVMFGSPETTTGGNALKFYASVRLDIRRIGAVKNGDEIVGNETRVKVVKNKIAPPFKQAEFQILYGEGINNLGELIELGVKHEFVEKAGAWYSCNGERIGQGKANAAKYLDEHPEMAKDVDTKLRDMFLSKTVVAEDKSEVKEKEKA

>sp_Q12UG7

MTEVLLEDLDHVGPATAQKLKDAGFTTIEAIAVASPAELANSAEIGESTAAKIINAARQSADIGGFETGDLVLERRKLVGKLSTGCTEFDEMMGGGIETQSITEMYGEFGSGKTQIAHQLAVNVQLPPEQGGLGGSVIMIDTENTFRPERIAQMVKGISDKHGIEYDPEEFLKNIHVARAFNSNHQILLVDSANELANELKNTEMPVKLLIVDSLTAHFRAEYIGRGTLADRQQKLNKHLHEILRFGDLSNACVVVTNQVMSKPDAFFGDPTKPIGGHILGHTATFRLYIRKSKGEKRIVKLVDSPNLPDGEALISVTTDGIGDA

>sp_Q47W34

MLLLTLTMIIAAYLIGSISSAILVCRFSGLPDPRTTGSKNPGATNVLRISNKFTAATVLFLDILKGTIPVWGAYFLKIDSLYLGFIGVSACLGHMYPIFFNFKGGKAVATALGTLLPIGFTLGGLLILTWVLVVKLTKYSSLAAIVTVSIAPLYVYFLKPLYVYPTLMLSALILFRHRDNIKRLLKGTESKITHKI

>sp_Q484R8

MSNDLTSESKTANRAAGEKFRDADKLAHIPIKVVSSTKATMLRKPSWLRIKLPKSSERIDNIKANLRKNDLHSVCEEASCPNLSECFNHGTATFMILGDICTRRCPFCDVGHGRPLAPKSDEPKKLANSLKDMGLKYVVITSVDRDDLRDGGAQQFADCVKEIGEQAPNTKVEILVPDFRGRMDRALEILNQNPPHVFNHNMETAPRLYTKVRPGANYQWSLDLLKRFGEANPDVTTKSGLMVGLGETNEEILEVMQDLRDHGVTMLTVGQYLQPSKDHLAVERYVHPDDFAMFEREAKKMGYEHAACGPLVRSSYHADKQAAGEEVK

>sp_Q9EV47

MIFQRTVKEMVKTIGVGLHSGNKVTLSIKPAPVNSGIVLVRTDLEPAVSIPAKAELVRETTMCTALVNDDGIRISTIEHLFAALAGLGIDNAIIEVDAPEIPIMDGSASPFVFLLQSVGIQEQAAPKKYLRIKKNIRVEDGDKWVELKPYKGFKIDFTIDFEHPVIARSEQHMKMEFSSSAFIRDISRARTFGFMRDIEYLRANNLALGGSMENAVVLDEYKVLNPDGLRYEDEFVKHKILDAFGDLYVAGYAIVGEFCAYKTGHALNNRLVRALLAQQDAWELVSFETEREAPVSFSVPSGAVFA

>sp_Q12UW5

MQEFSHIKDDRAYMVDISNKDTVVRYATASGKIKLHDETVEKIRSGDVEKGNVLATARTAAILAVKRTPDLIPMCHQIPITSVDVEFEIGKSDVTANVEVKSVGRTGVEMEALTGVSVALLTIWDMVKSAEKDNTGNYPSTAIENIHVIRKVKETITNQ

>sp_Q47ZQ7

MRSAFYISDGTAITSEVFGHALLSLFPTEFEHHTISFIETTEKALAAKERINKATSRGGKPALVFHTFVNNENREIIESCDAVLYNFLEPFVAPLEKELAIKAKPTTHRTHSIHEKSYDYRIEAVNYALTNDDGSNVTNYEEADVILVGVSRSGKTPSSLYLALQYGIKAANYPFTDDDMEELKIPSFLKPFHKKLFGLTIDAQRLIDIRDGRMANSKYSSARQCRMEVREVEKLYKNEQIPFINTTKLSVEEITAKILTETGLQRYKY

>sp_Q12VV9

MYMSRMKKKQKNIIKDVASERIERLFKLAAEEYSSNPGRSDRYVHLARRIGMKYRIRFPSPLKRKMCRGCSSYLVPGSSSRVRLHGRYMTITCLKCGREMRIPYHLKE

>sp_Q12ZU1

MRGIRSKIPRALNAGAKIECVDNTGARTVEIISVKKYRGVKNRMPKGGIGDMCVVSVKKGTPEMRKQILYAVIVRQKKEFRRPDGIRVAFEDNAVVIVDDKGIPKGTDIKGPVAREAAERFPKIGTTASMIV

>sp_Q47VT1

MKTFVAKPASVQREWFLVDAEDKTLGRIATEIATRLRGKHKAEYTPHVDTGDYIVVINAEKVRVTGNKAKGKIYYSHTEFPGGLKQISFEKLIEKAPTRVLEFAVKGMLPKGPLGREMFRKLKVYAGPEHAHTAQQPQLLEL

>sp_Q12Z92

MADFKIVVSDPKTKTYQFDITGAEANQFIGRSIGQTVDGATVGLDGYTLTITGGTDNSGFVMSPTLPGPRRQKVLIANGVGYSAVAKGVRRRKFLRGSEVAPDITQINTKVTGYSDKAIEEILGGGSEEVEAPAE

>sp_Q12XU7

MSLETPRTHYTSQIDIEQIGDDKVTVCGWVHEVRDLGGICFVVVRDREGRAQITLVKKKIDKEIFDAARKLVRESIVAVTGTAKAEGKAPNGYEIIPESIVVLNEAESPLPMDTTGKVDAELDTRLDSRFMDLRRERTTAIFKIRHEVLRAVRDFLSKDGYIETCSPKVVATATEGGTSLFPITYFDREAFLNQSPQLFKQILMSGGLDKVFEIGPIFRAEEHDTRRHLNEATSIDIEASFLDHFDVMEVLEDMVAYVYEQVIENEAASLKALDIELSVPKTPFMKVPYSQAIDIVNAESEETVEWGGDLGTVAEHTIGEHVFKETGESHYFITDWPTEIKPFYAMPYEDNPLISKSFDMMHRTMELSSGAQRIHIHDMLKARIESQGLDSDGFDFYLRAFKYGMPPHSGWGIGCERLVMTMLSVENIRDTVLFPRDRKRLSP

>sp_Q12TN5

MELKFNLKGAFKTSTDPTGAKEVIAQYFDEANNTILKKGAPEGQGAKITQWDIVDGSIELTIESGRYVRAHDAIMRLKKPLAAKLGKEFRIGIRGVDVKKFTISMPAEGEIGNMNIPHVSNISKVEGGLILELNVGESELERRIPDRILTLMEEKVRAKDYGGKAEHWQILWESDKKEHTFAGDPTQEMMKHGWIKRGASRGQWIHGPQSTKMFRTFEKIVYDELLEPLGYREMIFPKLVPWEVWQKSGHAKGVYPEIYYVCPPKTRDPAYWEEVSDHYKVTHEVPTELIKSKIGDPIGGLCYAQCPPFWMYLQGETIPTDEFPIKVFDKSGTSHRYESGGIHGIERVDEFHRVEIVWLGTKEQVIETARKLHERYMHIFNEILDLEWRKAWVTPWFMAQEGLTGLSEQGEAGTTDYEAPLPYRGDDGEWLEFQNVSINGNKYPSGFNVKSQTGEELWSGCSGVGLERWASAFFAQKGLDPENWPEEFRKRVGEVPKGIRFL

>sp_Q481G3

MSVISITDVLAGNFPVNESITIHGWIRTRRDSKAGISFLALHDGSCFDAIQAIVPNELDNYESDVLKLTTGCSVKVTGILVESPGKGQAFEIQATEVEVLGFVEDPDTYPMAAKRHSIEFLREQAHLRPRTNIGGAVTRVRNCLAQAVHRFLHSKGYFWISTPLITGSDCEGAGEMFRVSTLDMENLPRNDEGKVDYNKDFFGKETFLTVSGQLNVETYCNALSKVYTFGPTFRAENSNTTRHLAEFWMVEPEIAFADLSDAADLAEEMLKYVFKAVLEERPDDMAFFQQRVDKTVLDRLNSVINTDFVRLDYTDAITILENCGKKFENQVSWGVDLNSEHERYLAEEHFNGPVVLQNYPKDIKSFYMRLNDDGKTVAAMDILAPGIGEIIGGSQREERLDVLDSRLEEMGLDIADYGWYRDLRRYGTVPHSGFGLGFERLVAYATGMQNVRDVIPFPRTPNNAAF

>sp_Q480A9

MPVITLPDGSQRSFEQAVSVMDVALDIGPGLAKATIAGRIDGNLVDACELITQDASLQLITSKDSEGLEIIRHSCAHLLGHAIKQLYPNVKMAIGPTIENGFYYDIDLDESISEDDLVKLEKRMTELARTGYEVVKKTGSWQDAYDAFTERGETYKLAILDENIEKTDTPALYHHQEYIDMCRGPHVPSMRHCHHFKLMKVAGAYWRGDSDNKMLQRIYGTAWADKKQLKAYIVRLAEAEKRDHRKIGKTLDLFHWQEEAPGMVFWHNDGWTIYTELEKFIREKLHEYDYDEVKAPMMMDRSLWEKSGHWDKYADGMFTTTSEKREYAIKPMNCPGHVQIFNQGLKSYRDLPLRIAEFGCCHRNEPSGSLHGLMRVRGFTQDDAHIFCMESQVQAEVKKCIEMVYDVYGSFGFEDVVVKLSTRPDNRIGSDEIWDKAEAGLAQALTDSNIAFEYLPGEGAFYGPKIEFTLMDCLGRAWQCGTVQLDFALPERLGATYVGEDNERYTPVMIHRAILGSLERFIGILIEEFTGKFPTWLSPIQTTIMNITDKQAPYCEKVVKKLKENGFRAKIDLRNEKIGFKIREHTLKRVPYLLVVGDKEMESGEISVRTRSGEDLGKMSVDDFIAKLSDEVKSRQ

>sp_Q487Z4

MELSLKDASGALEVSEATFGREFNEALVHQVVVAYAAGARQGTRAQKTRSEVSGGGAKPWRQKGTGRARAGTTRGPIWRTGGVTFAAKPQDHSQKVNRKMYRGAIASILSELVRQERLVVVENFSVETPKTKELVAKLKGLELKDVLIVTKEVDENLFLSARNLYKVDVRDVAAIDPVSLVGFEKVLITADAVKEIEGILA

>sp_Q485F4

MASKQIFPDFEYPIAYCIAGVDEVGRGPLVGDVVTAAVILDPDNPIEGLMDSKKLSEKKRNLLSLEIKEKAISWSLGRASPQEIDTLNILHATMLAMQRAVEGLNVEPDFVLVDGNRCPTFLCNASESNQQNLKIASQAVVKGDARVTEISAASIIAKVARDNEMIALDKLHPEYGFAKHKGYPTKLHLEKIIEHGVLDCYRQSFKPVARVLGTYHD

>sp_Q47VS0

MFGNLLTKMFGSRNDRLLKQMSKEVTKINALEPVLEALSDEELKAKTTEFKERFTQGETVEQLLVEAFAVVREASKRVFGMRHFDVQMIGGMVLNEGKIAEMRTGEGKTLTATLPSYLNALTDKGVHVITVNDYLATRDADWSRPLFEFLGLTVGCNVAGMTTQDKQAAYQSDITYGTNNEFGFDYLRDNMVFSPQERSQKPLHFAIIDEVDSILIDEARTPLIISGQAEDSSALYKIINTLVPTLEQQEEEDKEGEESTGDFTIDEKAKQVYLTERGQIHIEEIMVEKELLTAGDTLFSAANITLLHHVMAALRAHKLFQKDVDYIVKDDEIVIVDEHTGRTMEGRRWSEGLHQAVEAKEGVNIQNENQTLASITFQNYFRIYEKLSGMTGTADTEAFEFNHIYGLETVIIPTNQPMVRKDLSDLIYLTTEEKFEAILADIQDCVKRGQPVLVGTIAIETSEFLSDFLKKAKIKHKVLNAKFHQQEAEIVADAGKENAVTIATNMAGRGTDIVLGGNLDATIAKLTNPSEDDIAKAKAQWKIDHERVLELGGLHIVATERHESRRIDNQLRGRSGRQGDEGSTRFYLSMEDSLMRIFASERISNMMRKLGMEKGEAIEHPWVTRSIENAQRKVEGRNFDMRKQLLEYDDVANDQRGVIYEQRNELLDNEEIGSVVEAIRSDVINGVIDQHIPRQSLDEMWDIEGLEEQLKGEYATELTIAKWLEDDSKLHEESLREKIITEFEQAYKDKEEAVGVDVLRQFEKAVMLQSLDSHWKEHLSAMDHLRQGIGLRAHAQKNPKQEFKRESFELFTEMLDNLKYDVVGILSKVQIRAESDVEAVEEQHRKSEEVPMDFQHQSASSPSEQAQTPRVGRNEPCPCGSGKKYKQCHGKLA

>sp_Q47VZ9

MQYNTSELCDFYADLIDVVDPIFCNYGGRSSFAGKVVTLKCFEHLGLINELVATYGTGKVLVIDGGGSTRRALIDLGIAQCAANNGWEGIVCYGSVRDVDALDGVDIGIQALGAIPVGASEQNIGETDMAINFAGVTLLPDDYIYADNTGIILSPEALEIASSMA

>sp_Q485E3

MKNIEIDEELYQYIATNTQFIGESASAILRRLLNLGVEAKLETVTVNEPSDVVEVNILPESEEKAVKIETAEVKEPKSKAVVKVTAESHETVFNFINKEELAMQRGAVGRFLLILAALYRAHPDQFGVVTDISGRDRLYFANSENKLAESGSSTKPRQIPESPFWVITNSNTTRKKMMLTKASISLGYSDSDVEKIRELL

>sp_Q47WN6

MTNNENSTNLLKVNKARLKWACRRGMLELDVLFIPFVDEAYDELSTKDQFTFERLLTGQDPELFAWFMGHEVCEDTELNAMVQLILKRVKV

>sp_Q48AU5

MFDILMYLFETYIQNESEAMVDHELLTDELTRAGFHQDEIYKALNWLEKLTALQDTDAYPYLTRVGSKSVRIYTSEEMQLLDTPSRGFILFLEQVNVLDFTTREMVIDRVMELDTKYFSMDDLKWVILMVLFNVPGKESAYSQLEDLIFEEQEGPLH

>sp_Q12WQ0

MAAPIAEELAKKQQAISVAEFFEKNRQILGFDSAPRSLITTVKEAVDNSLDACEEAEILPDILLHIERVGKDNVSVIVEDNGPGIVKEQIPKVFAKLLYGSRFHALKQSRGQQGIGISASVLYAQLTAGHPTSVISKIGPDSPAHHYEVMINTSTNDPEILLDEVIDWDRPHGTRVELEMEASYVKGRRQSIYEYLKATAIVNPHARLTLIEPDGNEVIFDRATDKLPIPAKEILPHPHGIELGTLMKMLRYTDRQKLAPFLRYSFSKIGLLTAEEICKAAGLDTEMLPSKLTRDQTKKLLDAFKKVKIMAPPTDCLSPIGEELIYKGLEKEFNVDFIATTTRSPSVFSGNPFVVEVGIAYGGVLQKDDRIDIMRFANRVPLLYQQGGCATTHAVEGIKWKQYGLNQPGGGMPTGPVVLLVHVASTNVPFTSESKDAIADIPEIRDEVELAIKEVSRKLNRYLNRQVSLKKRREKEIIITKVLPKMAQKLADTLERDLPDINPVVAKVMGNLLVMRHVEHGANGDAAVTIKVKNFGSKLTEFKLHDMLPYEISDVSPEPKVISMGSDFDYVWTMKVSPEGSKAVTYSLSSMSEDEIKRLPQLIVEGLDEELVTGAKAIKGLI

>sp_Q12W26

MTLDDAALKSFGFIERPTKNMINIDPLQTGGILTEDARRALVEWGDGYSICDNCGGVLDLIKKPPVQKFIHEALPEFLGVDEVRITHGARESKFAVMHAIAQEGDTVILDGLAHYSSVVAAQRARLEIRKVPHSEKPDYHIDPEAYGTAIEETISETGKAPALALLTYPDGNYGNLADAKKIASVCHEYDVPLLLNCAYSVGRMPVDAKELGVDFIAGSGHKSMASCGPIGVLGVNNDNGDYSDIIFRKSPTNKNKEIELLGCTARSATLMTMIASFPEVVKRTRNWGNEVADARWFSEKLETMGLIQMGQKPHNHDLMFFEAPNLYEISTRVKKGRYFLYKELKSRNIHGIKAGLTKFFKLSTFEVGRENLSYIVDSFDEIIKKYE

>sp_Q48A14

MEKSFDYDVIIIGTGPGGEGAAMNLAKRQKKVAIIERYHQVGGGCTHWGTIPSKALRQSVSRLIEYNSNPLFNQNEQVKQLTFQDILSHASAVIQKQVSLRSGFYNRNRVEHIQGQASFIDAHTISISHPDGSVEKISAKQIMIATGSRPYRPDDIDFDHPRVYDSDSILSLKHAPQHVIIYGAGVIGSEYASIFRGLGVKVDLINTRERLLSFLDTEMSDSLSYHLWNSGVVIRHGEEIERVESSEDAVIVHLKSGKKMRADCLLFANGRTGNTADLNLAAAGLKADGRGQLKVNDCYQTEVDNIFAVGDVIGYPSLASAAFDQGRIAASAMVDSSSKAKLIVDIPTGIYTIPEISSVGKTEQELTEAKIPYEVGRAQFKHLARAQISNNLVGSLKILFHRETKEILGIHCFGENAAEIIHIGQAIMQQTNGGNTIEYFVETTFNYPTMAEAFRVAALNGLNRLF

>sp_Q47WP8

MSELLLGVNVDHIATLRQARGTNYPDPVYAASVAEHAGADGITVHLREDRRHIQDRDIHVLKQTLHTRMNFEMAVTDEMIAIACDVKPVFCCLVPEKREELTTEGGLDVVGQLDKITKATEQLTAAGIAVSLFIDADKAQIDAAVASKAPYIEIHTGHYADLSSEEEQLIELERLTVGIKYAHNLGLKVNAGHGLNYFNVKPIAAIKEIIELNIGHAIIARAAIDGLDKAVRDMKQLMLEARTYNT

>sp_Q12U19

MDNRHLALYPFVTEASEYVGSLGFSPDKLLSSRALESARIRGRERVIQALEGEIEKPSPSSSEEGKILTELLSYPFSRILVSCIDDPFLTRKYALAEAKAAYHLLKTQQPEFLQDLATDFRINAEIYENPETHDIFFDLHFTDYIKLASPLKDLNWKLVNRKMKKGYVKISKEELARLLQEAIRLRIQNSLPVTVPKEICEACIPHTDTISEELEKKKTEFGVGEFQRVESDLFPPCITQAIANVRAGVNLAHSMRFAMTSFLINIGMSVDEVVAMFNISPDFDEEKTRYQIEHIAGTSSGTTYKPPSCNTMRTYGNCSAPDELCKGVKHPLGYYSRRVWVKNRMQNDNEKGHEEKKEGETPPQ

>sp_Q480B3

MNLADKVLAVNNDLPIRTDSPVHSGKVRSVYWLTATDSKRLIEEKGYDVPSDTSLAIMVISDRISAFDCIWSGENDMRGVPGKGAALNAISNHWFKMFKEQGLADSHILDIPHPFVWIVQKAKPVMIEAICRQYITGSMWRSYTKGERDFCGIELPEGLAKDSKLTSLLQTPSTKGILEGIPGVPAVDDVNITRKNIEDNFEAFNFKSVDDIARYEKLLTEGFDVISTALSKIDQVFVDTKFEFGYVKDVNGDDKLIYMDEVGTPDSSRIWDGEQYRAGKVVENSKEGFRQLLLNHFPDADILLNKDRMSEREALARDNKLPLSVLMDVSKTYTDIAEKITGNKIVLSDNPKAEIIAILREQYQLID

>sp_Q482M9

MHKKNRHNQGYNFTALVKAHPGLLQFIIKNQYNNQDTIDFANPQAVKALNLSLLKSEYHVKFWDIPDGYLCPAIPGRVDYIHHLQDLLAATPKTLLPNKTPINVLDIGTGASCIYPILGQREYDWHFVASDVDPISIKVAKHIISSDKSLNRNINCRLQPNSNQIFNGIIAEDEFYHLTICNPPFHSSLAEASKGTARKIKNLNKGNHSSKNQDKTLNFGGQKAELWCPGGELAFIGKMIKESKAYQKQVLWFTCLVSKKDHLSKLKLSLKKSDAKQIKVIDMAQGQKISRFIAWSFYDVN

>sp_Q12YN9

MKIIGIDEAGKGPVIGPMCIGGVRIDEDKSNALKNLGVADSKKLSPKRRVHLAAQIKKYADGWFVYEVSPNQIDELRKLMSMNDIMVLAFGSVIEELPSDKIYADAADVKEERFGKRLFDNYMEKHPDVSPPEVISKHGADDLFPVVSAASILAKVRRDELIEKIKVDMGVDIGSGYPSDPKTKKFLENWYRENSSFPDIVRHSWKTAQKFIQ

>sp_O24744

MEHTPQSQLKLLLAKGKEQGYLTYAEVNDHLPADMVDADQIEDIVQMINDMGIRVYEQAPDADEIMMTEDSTDDDAAEEAAAALATVEAELGRTTDPVRMYMREMGTVELLTREGEIVIAKRIEEGIYTVQASVAEYPPAIASILDQFDRYEAEEVRLSDIISGFIDPNAADIAPTATHIGSDLSQSERDKDDSKDDSKDDDEDEEEEGPKGPDPEESKERFTQLREVHENTLKIIADKGRGHPEATVALFEIGELFKEFRLMPKQFDRLVKNMRATMDKVRVQERLIMKLCVEQAKMPKKNFVKGYTADESSIAWFDTELASKKPYAEGLKMIEPDVRRCRFKLDIIEKETGLAIASIKDINRRMSIGEAKARRAKKEMVEANLRLVISIAKKYTNRGLQFLDLIQEGNIGLMKAVDKFEYRRGYKFSTYATWWIRQAITRSIADQARTIRIPVHMIETINKLNRISRQMLQEMGREPTPEELAERMLMPEDKIRKVLKIAKEPISMETPIGDDEDSHLGDFIEDTTLELPLDSATGESLRNATHEVLAGLTAREAKVLRMRFGIDMNTDHTLEEVGKQFDVTRERIRQIEAKALRKLRHPSRSEILKSFLDE

>sp_Q47WU8

MVTIRLARGGAKKRPFYQVVVADSRNSRDGRFIEKVGFFNPTAQGQAEKLRLDLDRITHWVGQGATVSDRVAKLVKDATAA

>sp_Q12U23

MSKRILLTNDDGVYAAGIRAAYRSVSDLGDVTVSAPAQQQSGVGRSISIFEPLRITRTTIDGIEVHAIGGTPTDSVILGIFTIMKELPDLILSGFNIGENISTDTITTSGTIGAALEGASYGVPAIAASLQVTEEGLKFDDLRDFQHDFDVGIKFVNGVAKKVLKNGLPENVDLLNINIPHFVEEDSEVEITRLARKFFRTGVEERRDPRGRPYYWIAGDLIHTAEKGTDVNAIEKGHISVTPISLDATSPINFSEIEHLM

>sp_Q12ZG8

MVMDKLGSSLQDALKKLVGAGRIDEKTVTEVVKDIQRALLQADVNVKLVMQMSSHIKERALKEEVPPGMNPREHVIKIVYQELISIVGRSADIPLKPQTIMMIGLQGSGKTTTTSKLSRYFQRKGLKPAVICADTFRPGAYQQLKTLCDKLNVPFYGEVGNPDAVGIVERGLAELGKNDVLIVDTAGRHSLEADLIDEMEQIHEIAQPDYKLLVLDGAIGQQASEQARAFNDSVGISGVVISKLDGTAKGGGALSAVSETNSAIAFIGVGETPDDLEKFEPDRFISRLLGMGDIKSLIEKAEETLSEEDIDMEAMMRGRFTLKDMYSQLEAMNKMGPMKQIMQMLPLGGMGAKLSDDAYKVTEDKMKGYRVLMDSMTEEELLNPRLLGSSRIKRISLGSGSSPDAVRELLKYYKMMQNAMKGLRGGKFNIQKMMKKMGM

>sp_Q12U87

MREKGKLVIWPANLDKSRSRKGGRIISRKSSLEAPLLRELTAAAEKLNLNPEVEADKKYPRTWWESSGRILVDNNEAKTMVARKIAKTIKEARGG

>sp_Q484R1

MKLTLYAVGSKMPAWVSQGFAEYSRRFPRDLSFNLVEIPAGKRGKNADISRILAKEGELLLAAIPKGNRIVTLEVEGQSWTTPKLAKQLEQWQLDGRDVALLVGGPEGLAPACIKASEQKWSLSALTLPHPMVRILIAESLYRAWSVNTNHPYHRE

>sp_Q47VL3

MAHKKAAGSTRNGRDSEAKRLGVKRFGGESVLAGNIIVRQRGTRFHAGSNMGIGKDHTLFALSDGKVQFEVKGPKSRKFVSIIAD

>sp_Q488Z9

MMTDPIADMFTRIRNGQSAAKVAVQMPSSKVKVAIATLLKEEGYISEFSVSGEVKPELAVTLKYFEGKEVIEKIKRVSRPGLRIYKSCDELPKVLAGMGIAIISTSKGLMTDRAARSAGIGGEVLGFVE

>sp_Q47W36

MPVIKVRENEPFDVALRRFKRSCEKAGILSEVRRRESYEKPTWERKRKKAAAVKRAAKKVSRENARRVRMY

>sp_Q12ZD1

MIKEVTDQYNAKEIETKVHEFWEANNAYRSVREHRKGAKKFYFVDGPPYTTGHIHLGTAWNKIIKDSILRYMSMNDHDILDRAGWDMHGLPIEVKVEGALGFESKKDIETYGVGNFIEKCKEFALRQKDDMTEQFQTLGVWLDWEDPYMTLKDEYIEAAWWTLKQAQDKNLLETGKRVVNWCPRCETAIADAEVEYEDREDPSTYIKFKLKDEDNTFVVIWTTTPWTIPSNIAVAVHPEFEYSKVKAISENSATEILIIASELVENVLRIGRYLDYEILSTMSGKDLEGTVYEHPLADLVPLQAEIEHRICNADYVTADSTGCVHIAPGHGVDDFEVGVKNEFPIFCPVGPNGSYTHEAGKYCGMNILDANRVVMDDLLERGLLMAERMISHRYGHCWRCKTAIIYLATEQWFLKIGELKEDMLEEIKKVNWTPEWAGSARFKDWIEGARDWCISRQRYWGIPIPVWKCSSCNSLTVVGTRKELIERSGADPHIELHRPYVDKVTIPCECGGTMKRVEDVFDVWFDSAVASWATLRFPHQKEKFNEWWPADFITEGHDQTRGWFYSQLGASMVAFGKAPYKNVLMHGFTLDGSGKKMSKSIGNVIQPAEVIDKFGADTLRSYVLSASAPWEDLKFNWDELATVHRTNNILWNVYRFPLPYMALDDFDPQKVSYESVEAYLRSEDKWILSRMQTVIAEVNKAMDARLLHKAMRSINEFVLEDLSRWYIQLIRPRTWVEADNPDKLAVYRVLYDVFVTTAKLIAPFMPHLAEEMYQNLVRNIDENAPVTIHLCDWPVVNEALVDTSLEAQMKVARSIVEASSNARQKVGRKLRWPVSRIVVSPTDENTIAAVEGLRSVLMDQTNAKDIETTKVGESWNELGVESTPNPGAIGPVFKGNAGNISAAIGAMDAYDLKKGLAGGEMEISLADGTNVTITEKMVNFSETVPEDVGSAEFNCGVVFVDAKLTHEIESEGYSREVIRRIQDMRKELDLDVDDSIRGHIQISDERVLDLVLDFENYIAKEVRANVLVIGLDVETTGELAKEWNVEGIPMTIAISKEE

>sp_Q12ZR5

MAYPGKSTKSYDTPQHPWQAERMASEVELVKKYGLRNKRELWKSLSVLRRFRGDARRLLAESAESDLIGHSKTEADQLLTKLIRFSILKSDSNIDDVLGLQTEAILERRLQTQVHRLGLARTARQARQFITHGHIAIDGKRVTVPGMMVTREQEMNVEYYGTSPMTKESHPERPAQIAASVVEE

>sp_Q12UQ9

MSKFPSEKPVLVTCGLPYANGKAHVGHLRTYVPADIFTRSLKKTGQEVTFVCGSDTHGTPIVFNAEELKTTPTEIIKVYHKHFDEIFKKMGVMLDAFGTTDDPTNHNRTTEIVSKLIENGYVYPKTIEIAYCPSCDRSLPDRYVKGTCPHCKKEARGDECDQGCGKHLEPGELEHPACTTCNGPAEYKQQEHFFFKLSQFKDFLLEYLEGLGGTLNARNYALGWVKQELTDWCITRSLDWGIKFPGHEDLVVYVWVDAPIGYIAFTEEWAEANNESWEKFWKDDGSIIHFIGGDIIYHHCIFWPAMLKGAGYNQPDAVVASGMVKIEDRTFSKSRGYVVWVDEDYLDHGFHQDLLRYYLASYTSHTKELNFSWKVFQDKVNTELVGVFGNFLYRTLLFTHKNFGEIPEGEVKQDILDEINTTIENAKEAMENYEFKKYADTVMALASYGNTYFQSNEPWKLIKENKEACGEIVKNCAQITKALCLLFEPILPEKMEEAWKQIGMETDVHETNYMEATELVKSGTTLEKPSILFEKIEDEKTEEMEAISAARVKEAIAKENGTEEVEEVKEIEEMKDLITFDDFSKLDIRIGTIVSAEAIKKSKKLLKLQVDLGEEETRQIVAGLKESHEPEQLIGKQVAVLTNLAPAKLCGVESNGMVLAGVDAADNAILLQPEKETNPGTCIH

>sp_Q48A96

MNNSTDAVNPAKKPQTRREKREAAEAFLKNVSDQSFPNSKKVYVQGEIHDIKVGMREITLSDTLVSGSKDKPVYEKNEPLCVYDTSGFYTDENVEIDVHKGIPRLRETWIDARDDVETFTSTHSEFAQQRLDDEGVDEIRFEHLPKMRIAKKGKNVTQMHYARQGIITPEMEYIAIRENLKREEVKDATLLLQHKGQSFGASIPEQITPEFVRDEVARGRAIIPVNINHPECEPMIIGRNFLIKVNANIGNSAVTSSIEEEVEKLVWSTKWGADTVMDLSTGRNIHETREWIMRNSPVPIGTVPIYQALEKVNGVAEDLTWEIFRDTLIEQAEQGVDYFTIHAGVLLRYVPMTAKRVTGIVSRGGSIMAKWCLAHHKENFLYTHFEDICEILKQYDVSFSLGDGLRPGSVADANDEAQFAELHTLGELTKIAWKHDVQTIIEGPGHVPLHMIKENMEEQLEHCGEAPFYTLGPLTTDIAPGYDHITSGIGAANIGWYGCAMLCYVTPKEHLGLPNKEDVKEGLMTYKIAAHAGDLAKGHPGAQIRDNAMSKARFEFRWYDQFNIGLDPERARTYHDETLPQESGKVAHFCSMCGPKFCSMKISQEVREYAANLDKNAIKIQLLDETITLTSDEAIERAMQEKSAEFKATGSEIYQLAE

>sp_Q48A93

MVALNIYGQQLDSRLLIGSALYPSPHVMKQAILASGSQVVTLSLKRQNPAEKAGQQIWRYLQEVVTEVNGHLLPNTAGCKTAKEAVTLAKMSREIFQTDWIKLEVIGDDYNLQPDPIELLHATEQLINDGFKVLPYCTDDLVLCQRLYDLGCQVIMPWASPIGTGKGLMNPYNLETIRLRLPNATLILDAGIGKPSDACLAMEMGYDGVLLNSAVALADNPVLMAKAFGQALQAGEQGYVAGIMDQRQTAHPSTPTLDTPFWHQN

>sp_Q47XL7

MQVSVETTQGLERRLTISVPAEKVDVEVKNRLRQISKTQRINGFRPGKVPPSVVQKRFGKSVRQEVAGEIMQRNFVDAIVAEKINPAGRPSFVAKSNEDGKALEFEATFEIYPVVELKDLEKIAIERPEVDVTDADLDEMFVTLQKQHQTWKENKRKTKSGDKLTLDFTGRVDGEEFEGGKAEGFELELGAGRMIPGFEKEVTGMKAGDEATIKVTFPEDYHAENLKGKDAEFDIVIHKTEGPILPEIDEEFAKLFGIEEGGVEALRVEVSKNMARELTQAVKAKVKTQVIDGLLAGHEVDLPSALVTQEVDVLRQQAMQRFQGQMDPKNLPQLPAEMFTEQAERRVKIGLLLGEVIKVNELKVDETKVNELIASAASAYEDPKEVIEYYANNNELMQQMQNVALEEQAVELLVEKAKVSDKKASFNEIMNPEAK

>sp_Q47XJ9

MKKLDKTNNSNKLSYQKDGTPYCQRFDDIYFESESGYQQSDFVFIQKNKIGLRLQIAKQTFTVAETGFGTGLNFLLTLQAYQKAQQVSSFQLAPLHFISVEKYPLTKEQLVQSLSILPQLQSLALTFINSYPDCPVEEFGQEFKTTFFNGQVRLTLIFDDAAQGFSSLNCSKEGLVDAWYLDGFTPAKNPDMWSKDLFSQIGRLSKDQATLTTFTVAGFVKRQLRDIGFRLEKLLAKGKKKEMLSAVMQSNPITNKGYYLRPLITKPQHVSIIGGGIASACAAYALTKQGVKVTLYCKDTSLAQGGSSNAIGALYPLLHQQEDDISSFYQQAFWRAKALYTEIAEQGFSFAHQWCGLLEVSYKEALIKRQQAFESLNTWPNELIHGVNAKQASELANIDLPYGGLFMPNAGWMSPRDLVKQIFNAAKSTTRLKIMTDTHITKIQQVANPSSNENGTSWSLTSNQGEFNASVLVICGGADAIEIEQLKSLPLTATRGQVTSMKSNKKINKLSTVICHKGYLTPENNGIHCIGATFQKNDTNITTNKADDDYNLTMLTKCLPELSSTIDWQEQDISSSKARLRCMSQDHLPLVGAVPDIKEHVATYSHLAKDKNWKYSQAAPCIDNLYVLLGLGARGLCSAPLAADILTAELCNTPYPVDSQMLFNLSPNRFIIRDIIKRKIKS

>sp_Q47XD4

MSIIKMADLTLANQRVLIREDLNVPVTDGKITSDARLRAALPTLKLALEAGAKVMVMSHLGRPTEGQPEEKFSLKPVTDYLSAALNYPVRLVTDYLDGVDVAAGELVVFENIRFNLGEKKNDDTLAKKLAALCDVFVMDAFGTAHRAQASTHGVAKFAATACAGPLLSGELEALGKALDNPARPLVAIVGGSKVSTKLTVLDSLAGIVDQLVVGGGIANTFIAADGHNVGKSLYEADLVEEATRLTKQAKANNGDIPVPSDVVVATEFSASATATLKPVNEVTADEMIFDIGPETAKALTEIIANAGTIVWNGPVGVFEFDQFGEGTKVIAQAIADSSAFSIAGGGDTLAAVDKYEIADKISYISTGGGAFLEFLEGKKLPAVEILEQRAK

>sp_Q47W59

MKTVSQISELRAQVKAWRQQGLTVAFVPTMGNLHAGHISLVAEAHKHADKVVASIFVNPMQFGVNEDIENYPRTMINDEQKLTAAGTDLLFTPSPDIIYPKGLAKQSFVEVPNISDGYCGESRPGHFRGVATIVCKLFNLVQPDVACFGLKDYQQVQVIQRMVEDLSMPITIIPVATIREESGLALSSRNGYLTEEEKAIAPALHQSLHWLGEQIRAGYAQQDSIDFIGLAKHAAKTINDAGLHTDYLHVCHAETLQPASEDDTQLVILAAAHCGKARLIDNLQVNLA

>sp_Q48A24

MTIAILPARLANQIAAGEVVERPASVIKELIENSLDAGATSIHIDVDKGGIKKIKITDNGHGIVKEELTLALSRHATSKIKSLNDLEAIGSLGFRGEALASISSVARLTLTSKPQSQATAWQAVAEGRDMSVNIKPAAHPDGTSIEVLDLFFNTPARRKFLRTEKTEFNHIDEVVRRIALAHFEVSFSLTHNGNTVRQYRMASTHAQCIKRVAMVCGPKFIEHAVEVDCPHDNMTLSGWLAKPSFSRSQNDLCYSYVNGRMMRDKLINHAIRQAYADLLPPDTYPAFVLFLQLDHREVDVNVHPSKHEVRFHQSRYVHDFIYSVCHKALTSALAGEELFTTADSDLALVPEQSYSSLGANDRQGSHSSNTPTLNYPSADYIRPLQHVNDASNSQSTSSYSGYGQQHKTNAISKIAASNYQALMTPDKGSSAQVQNTSGSDQASAQKHETTTLQNSTDQSAFLSVHQPGYALYKTENGVRVLSLFKLAKSTYGKLVEQSWQNKTEQSDCPVECLVSQPLLLPVILSLSEQQLSFVLAEQEILSNAGIVFIQQHKNKIQIRQFPALLREQDVSNALIIIIEELIEKRSFTEGEALCETNLHQSIGLAMVLAEYDETQADILLRLTKKLFHEQLSQQLLLNSIPLDLTSHIKTLF

>sp_Q12UF9

MEQELMRIGVSLPDNLLNKFDSIIEGRGYSSRSEGIRDSIRTYINQYEWMSDIRGRRVGTITIIYDHTKRGLSNAVADIQHDYSDLIKSSVHIHLDHDNCLEVIIFDGEGELIKEMDERLMALKGVKYVKLNTAPPAEKI

>sp_Q485J1

MKPSPRRKARELAVQAVYSWQVSKNPVNDIEVNFIADNSKRRFDIEYFQLLLRGVTTNIGSIDEAIIPYVDRPLDDIDQVEKAILRVAVFELKDCTDVPYRVVINEAIELAKSFAADDSHKFVNGVLDKTVKLIRPQE

>sp_Q47WV5

MSVNFVDSCRLPTHLGEFEMYGFVEESGQEHIMLTYGEITPDKPLLIRLHSECLTGDSLFSMRCDCGYQLETALENIVDAGQGALLYLRQEGRGIGLINKIKAYHLQDDGADTVEANEQLGFAADLRRYTMCKPMLEHFRVNKVKLLTNNPKKVQALKDLGIEVVEQMPIQVGRNQYNHEYLNTKAERMGHMMTHGLLSDLG

>sp_Q488A0

MLQPKRTKFRKQFKLRNRGLAHVGSSVSFGTFGLKSMERGRMTARQIEAARRAMTRHVKRQGKIWIRVFPDKPITKKPLEVRMGKGKGSVEYWVCQILPGRVLYEMEGVSEEIAREAFALAASKLPFKTTFVTRTAL

>sp_Q485S0

MTKNIAQAAVKFEQWQPKIEQESYLTINSLECHTGGEPLRIITSGFPVLKGNTILAKANDCKQNYDQLRRALMFEPRGHADMYGAIITDAERDDSHFGAVFIHNEGYSSMCGHAVIALTKTAVESGVVARTGDVTQVVIDVPCGQIYAMAYSHNNVVKHVSFQCVPSFVYAKDQQVEVDGIGMVQFDIAYGGAFYAYVQASSLGLSLVPEQQEKLIAYGRKIKQAIIPQFEINHPTTAELSFLYGVIFIDDSPNQDVHSRNVCIFADGELDRSPTGSGVSGRIALHHAKQQIVLNETITIESILASSFSVRAIETVCFAGFDAVIPEVTGDAYVCGKGQWFINAEDPLKYGFLLR

>sp_Q47V01

MTDFNQAFAELKRGAEEILVEEELLTKLKTGKPLKIKAGFDPTAPDLHLGHTVLINKLRQFQQLGHEVIFLIGDFTGMIGDPTGKNVTRKALTKEDVLANAETYKEQVFKILDPAKTTVAFNSTWMDKLGAAGMLQLASRQTVARMMERDDFKKRYANGQAIAIHEFMYPLVQGWDSVALEADVELGGTDQKFNLLMGRELQKSEGQRPQTVLMMPLLEGLDGVQKMSKSLGNYIGITDTPTDMFGKIMSISDVLMWRYYELLSFKPLEEIEGYKTEIENGKNPRDVKIDLAKELIARFHDEAAAQAAHDEFINRFQKGALPDDMPELTITTENGEIAIANLLKDAGLVGSTSDAFRMIKQGAAKIDSEKVTDRSLVISAGTTAVYQVGKRKFARITVS

>sp_Q483C6

MTIILGIDPGSRFTGYGVIKQEKQRFTYLGSGCIKAMSQGEDLASRLQTIFAGVSELIIQFKPDMFAIEQVFMGVNPGGALKLGQARGAAIVAATNSGLTIAEYSARQIKQAVVGTGAADKNQVQHMVKSILKLPGTPQADAADALAVALCHGHSHTSAAILAGQATKIVRGRLR

>sp_Q487D7

MEHYLSIFIKTIFIENMALSFFLGMCTFLAVSKKVSTAIGLGVAVVVVLGLAVPANQIIYQAILAPGALDSLMGITDPKESIDLSFLSFITFIGVIAALVQILEMLLDKYFPALYQALGIFLPLITVNCAIFGAVSFMVAKNLTLGESVVYGVGSGIGWMLAIVLMAGLREKMKYSDVPNGLKGLGITFITAGLMAFGFLSFGGISL

>sp_Q487G8

MTLFEKLRCLFTFGQGVALPLPELSSNTTPDQIFEQWFADANKSGILLPEAMSVSSCNSDGQPSSRMVLLKDYDKEGFVFFTNYESRKSHELAENNKVALLFHWNVLQRQIRIEGTVEKVSIQESADYFHSRDRGSQVGAWASKQSQKLKYDDELKERMSHYQDKFSEGEVPHPEFWGGWRVKPHAIEFWQGRANRLHDRLCFEKDGETWLNHKLNP

>sp_Q48AT5

MTTLNAKLASQYPAHIAQLQQMTKSVLSRENLEGLVIHSGQEVKAFLDDNCYPFKVNPHFKYWLPLIDIPNSWLVVNGEDKPTLIYYQPVDFWHKVTPLAESYWGEFFNIKILTKASEVDKLLPYDKKGFAYIGSHIEVATALGFEAINPEPLLNYVHYHRGYKSKYEHECLRQSNALAVKAHQAARNAFLQGDSEYDIQQAYLKSIGYGTNDTPYGNIVALNKNCSILHYMSLDKMTPQVHQSFLIDAGANFNGYSADITRTYSYKNDKFAELIARMDQLMLNAVAGLKPGVSYVDLHIETHRAIGQVLRDFNFINVDADTAVESGIISTFFPHGLGHHLGLQVHDVGGFMADERGTHVNTPAEHPFLRTSRVIETNQVFTIEPGLYFIDSLLADLKASANADQVNWQNVDEMRCFGGIRIEDNIIVHQSHNENMTRDLGLS

>sp_Q47UQ0

MTDFNIATMAQKAKQASRVAAQLNSSEKNALLNDIATAIENNSDAIIQENSKDIAAGREKGLSQAMLDRLVLTDKGIKDMSSAIREIVALTDPVGDVDKLSLRPNGIQVGKMRIPLGVIAMIYEARPNVTAEAAALCIKSGNAVILRGGSEAIHSNLAIAHCLHQVLTTHNVDEHIVCVIPDTSRSVIEQLLQQSDTIDLVIPRGGEGLIRYVSENSRIPVIQHYKGVCHLYIDKYADLDKAVNILVNGKTQKPSACNAFETVLVHSDISAKFLPLAAKALNDAAQVKVHACKNSIGFFPNAELATNEDYQAEYLAQEIAVKVVSSFDTAIGHINEFTSDHTEVIISQDISRSQKFIRQINSSVVMVNASSRFSDGNQLGLGSEIGISTSKLHAYGPMGLEALTTEKFVVFGDGQIRQ

>sp_Q489A3

MIQMQSQLNVADNSGAKRVQCIKVLGGSHRRYARIGDIIKVAVKEASPRGKVKKGDVHTAVVVRTKKGVRRTDGSAIRFDENAAVMLNANLQPIGTRIFGPVTRELRNEKFMKIVSLAPEVL

>sp_Q12ZC4

MSKSFYGYIRDAWKNPDETYVRDLRWERLQVWRKEGSVTRVERPTRIDRARSLGYKAKQGIVVARVKVRRGSMRKSRYIRGRRTQHTGKNKITVGKSIQRISEERAARKYPNMEVLNSYWVGEDGKQKWYEVILVDPSHPVIKSDKNLNWICGKAHSGRVFRGKTSAGRKGRGMQTRGTGTEKTRPSVRSNLNRSKK

>sp_Q47WD4

MSLILSNNYQALGESFSQQTLPAPVGQPSLLLWNEPLAKALTIPFTKDNDAELLSQYFSGNQLIEGSKPVAQAYSGHQFGHFNPQLGDGRAHLLGDIADTQGQRWDIQLKGSGVSDFSRQGDGRCALGPALREYIMSEAMFALGVPTTRCLAVVTTGENVYRERPYDGAVVTRIAASHIRVGTFQYFAARGDTDSLKKLTNYAINRHFPELIANSSENSSDNSDALKADNVISSQQVLEFFSAVLSKQIPLVLSWLRVGFIHGVMNTDNTTISGETIDYGPCAMMNAYHPETVFSSIDRNSRYAFGKQISIMQWNMTRLAETLLPLVDSDEDDAVEKIEPLLTQFHQDLQQGYLTMMAAKIGIDEPAEGDGKLINDLITLMKEQKLDYTQTFTALTDSLNDNTTEEPLTDVLNEWLPHWNSRIESFKSSAHTLMVSNNPIVIPRNHHVEAFLDSCQETGDLTALNKFLAVLRQPYTEIAETKNYQDAPVDGDKDYHTFCGT

Sequences of mesophilic proteins

>Mes35

MRIKNSGILLLAAILLFSCDKKRVFDEYKSVGSAWHKDSVVTFDLPVLDSTKKYNLFVNLRDNNNYPFNNLFLIVAIETPSGFTKVDTLEYQMANPDGTLMGNGFTDIKESKLYYKEDVKFKGKYKVHIKQAVRESGKIPGVEALEGITDVGFRIEQKD

>Mes38

MAKIIGIDLGTTNSCVAIMEGNTTKVIENSEGARTTPSIIAYQEDGEVLVGASAKRQAVTNPRNTLYAVKRLIGRKFTEKEVQKDIDLMPYTIAAAENGDAWVEVRGNKLAPPQVSAEVLRKMKKTAEDYLGETVTEAVITVPAYFNDAQRQATKDAGRIAGLDVKRIINEPTAAALAFGLDKHNDGKGGDRKIAVYDLGGGTFDISIIEIADVDGEMQFEVLSTNGDTFLGGEDFDQRVIDYIIAEFKKEQGVDLSKDVLALQRLKEAAEKAKIELSNSTQTDINLPYVTADASGPKHLNIKLTRAKLEALVEELIERTIAPCRTAIKDAGVSTSQINDVILVGGMTRMPKVQEKVKEFFGQEPRKDVNPDEAVAVGAAIQGQVLAGDRKDVLLLDVTPLSLGIETLGGVMTKMITKNTTIPTKFAQTFSTADDNQPAVTIKVFQGERELASGNKMLGEFNLEGIAPAPRGMPQIEVSFDIDANGILHVGAKDKGTGKENKITIKANSGLSEEEIQKMVKDAELNAAEDKKKLELVQARNQADAMVHSVKKSLAEHGDKLDAGEKEKIESALKDAETALKGEDKAEIEAKTEALMSASQKLGEKVYADMQAAQAAAGAAAGGGEAPAAEATASKPADDNVVDAEFKEVKDQK

>Mes49

MKKYYAVTGIALAVGMLCTTQLAGATQAADPSVGSLDSSNVVTEFSAQGNVEQITFKSAIKSAPMSSARSAQTSAIIPGLKNLFVSAPGSDFSLNDSSNNYIKRFTQNIAGIPVLGSSITEVLDGQGAVTSAIGAVTSATKGAFPADLAAGQAAALASATKIASAGKDASAISLVDQKAIWFDAVLIGKGATGSVAVPAYQFSFTTGFAESRVLTVAANDGAILNDRTDRKDINRVVCDANSKVIDLEASNADALLKCGKTQANKPTRIEGQAASSVADVNSVYNFLNDTASFYGANTKANDLTALIGNDEGDGLGKAMRAVVRICVTDSQNGEQCPFANAFWYNGQMTYGQGVTTDDITGHELTHGVTEKTNGLVYANESGAINESMSDVFGEFIDLSNGSSDDTAANRWAIGEGSSLGVIRSMKDPGKYGEPAIYKGSNWKPTATNPNDNNDQGGVHSNSGVGNKLAFLITDGQTFNGQTVTGIGIAKAAQLYWAAQRQLTANATYSSLGKALNSACSANVSNNVAGTTAANCTQVANAIKAVGIK

>Mes53

MVDTPYRKMLAAKIHRATVTGADVNYEGSLTVPPELLVAAKIHPYESLHVWNVTRGTRLETYAIEGLPNSNDVCANGAAAHLIRPGDHVILAAYAMVPEADAATHKPRLIFVDDNNQLSHVGPEIAGPNLRSDSDDTHLVRSTEMTPDGQPLAEGC

>Mes56

MQDDPRYRVEVEVSPRFLAHQSTPDEGRYAFAYSIRIQNAGAVPARLVARHWQITDGNGRTEQVDGEGVVGEQPWLRPGEAFHYTSGVLLETEQGQMQGHYDMVADDGTEFIAPIAAFVLSVPRTLH

>Mes58

MFDVIVKNCRLVSSDGITEADILVKDGKVAAISADTSDVEASRTIDAGGKFVMPGVVDEHVHIIDMDLKNRYGRFELDSESAAVGGITTIIEMPITFPPTTTLDAFLEKKKQAGQRLKVDFALYGGGVPGNLPEIRKMHDAGAVGFKSMMAASVPGMFDAVSDGELFEIFQEIAACGSVIVVHAENETIIQALQKQIKAAGGKDMAAYEASQPVFQENEAIQRALLLQKEAGCRLIVLHVSNPDGVELIHQAQSEGQDVHCESGPQYLNITTDDAERIGPYMKVAPPVRSAEMNIRLWEQLENGLIDTLGSDHGGHPVEDKEPGWKDVWKAGNGALGLETSLPMMLTNGVNKGRLSLERLVEVMCEKPAKLFGIYPQKGTLQVGSDADLLILDLDIDTKVDASQFRSLHKYSPFDGMPVTGAPVLTMVRGTVVAEKGEVLVEQGFGQFVTRRNYEASK

>Mes63

MDTMVDFKTLPYRKGVGIVVFNREGQVWIGRRLITSSHTYAEVSKLWQFPQGGIDEGEEPLDAARRELYEETGMRSVNLIKEVQDWFCYDFPQELIGHVLNNQYRGQMQKWFAFQFIGETSEIVINSPENSNKAEFDQWKWINLEVLPSIVVSFKRHVYMKVVHEFRNII

>Mes66

MNDILETETPVMVSPRWDMLLDAGEDTSPSVQTQIDAEFRRVVSPYMSSSGWLCTLTIECGTIICACR

>Mes69

MPIVSKYSNERVEKIIQDLLDVLVKEEVTPDLALMCLGNAVTNIIAQVPESKRVAVVDNFTKALKQSV

>Mes71

MKEQITICGAGLVGSLLAVYLIERGFSVRVFEKRKDPRKNEADAGRSINLAISHRGIHALKDAQTGLEKEALKLAVPMYGRAIHDLHGHVSFQAYGEASQHINSIGRGALNKLLITTAENLGVHFLFEHTCTDYHAAGEQWLFSDITGNTVATQSKEIVIGADGAFSIVRSFLSKQQQPQPQIETLEYGYKELEIASAHTETITNNQALHIWPRERFMLIALPNEDGSYTATLFLPLKGEISFEALQSDQDIQLFFKKYFPDTENLFPDLTEQFYRHPTSKLFTIHSSNWFNAHTLLIGDAAHALVPFYGQGMNAGFEDCRILAEIIDGKSKTNWSEIFAEFYNQRKENADAISDLALQNFIEMRDHVADASFLLRKKIEKHLHQELEDAFIPQYTMVSFTDISYKEAMETGLLHQKILDEIMAIPDIEAAWPTEELKNKVITVTKKYI

>Mes73

MKRIIASLALSVFCAGLAFAADELTFKAKNGDVKFPHKKHQQVVGNCKKCHEKGPGKIEGFGKDWAHKTCKGCHEEMKKGPTKCGDCHKK

>Mes75

MIPLRQNIASMKGYIPGYQPPDIASWIKLNTNENPYPPSPEVVKAILEELGPDGAALRIYPSASSQKLREVAGELYGFDPSWIIMANGSDEVLNNLIRAFAAEGEEIGYVHPSYSYYGTLAEVQGARVRTFGLTGDFRIAGFPERYEGKVFFLTTPNAPLGPSFPLEYIDELARRCAGMLVLDETYAEFAESNALELVRRHENVVVTRTLSKSYSLAGMRIGLAIARPEVIAALDKIRDHYNLDRLAQAACVAALRDQAYLSECCRRIRETREWFTTELRSIGYDVIPSQGNYLFATPPDRDGKRVYDGLYARKVLVRHFSDPLLAHGMRISIGTREEMEQTLAALKEIG

>Mes77

MRLRTALLATTLMAAAPVAANATIITGPYVDLGGGYNLVQNQHGHFSNDPANASMLTKSSSQYRHDAGFTGFGAVGWGFGNGLRLEAEGLYNYSEINHRAPTAATGVTSGHDQSYGGMLNVLYDIDLKQFGIDVPVTPFVGVGAGYLWQNVSPTTTRYSNGNVSRLGGTNGGFAYQGIVGAAYDIPNMPGLQLTAQYRMVGQAFSDGPFTMTSYTNGVGKSVGHAFFDNRFNHQFILGLRYAFNTAPPPPPPAPVVVPPAPTPARTYLVFFDWDRSDLTARAREIVAEAAQASTHVQTTRIEVNGYTDNSAAHPGPRGEKYNMGLSIRRAQSVKAELIRDGVPTGAIDIHGYGEQHPLVPTGPNTREPQNRRVEIILH

>Mes78

MAHVRRKVATLNMALAGSLLMVLGAQSALAQGNFSRQEAARMAHRPGVMPRGGPLFPGRSLAGVPGFPLPSIHTQQAYDPQSDFTARWTRADALQIKAHSDATVAAGQNSLPAQLTMPNIPADFPVINPDVWVWDTWTLIDKHADQFSYNGWEVIFCLTADPNAGYGFDDRHVHARIGFFYRRAGIPASRRPVNGGWTYGGHLFPDGASAQVYAGQTYTNQAEWSGSSRLMQIHGNTVSVFYTDVAFNRDANANNITPPQAIITQTLGRIHADFNHVWFTGFTAHTPLLQPDGVLYQNGAQNEFFNFRDPFTFEDPKHPGVNYMVFEGNTAGQRGVANCTEADLGFRPNDPNAETLQEVLDSGAYYQKANIGLAIATDSTLSKWKFLSPLISANCVNDQTERPQVYLHNGKYYIFTISHRTTFAAGVDGPDGVYGFVGDGIRSDFQPMNYGSGLTMGNPTDLNTAAGTDFDPSPDQNPRAFQSYSHYVMPGGLVESFIDTVENRRGGTLAPTVRVRIAQNASAVDLRYGNGGLGGYGDIPANRADVNIAGFIQDLFGQPTSGLAAQASTNNAQVLAQVRQFLNQ

>Mes80

MDAFDVIKTPIVSEKTMKLIEEENRLVFYVERKATKEDIKEAIKQLFNAEVAEVNTNITPKGQKKAYIKLKDEYNAGEVAASLGIY

>Mes81

MIDAKSEHKIAPWKIEEVNALKELLKSANVIALIDMMEVPAVQLQEIRDKIRDQMTLKMSRNTLIKRAVEEVAEETGNPEFAKLVDYLDKGAAIVVTEMNPFKLFKTLEESKSPAPIKGGAIAPCDIEVKSGSTGMPPGPFLSELKAVGIPAAIDKGKIGIKEDKVVAKEGDVISPKLAVVLSALGIKPVTVGLNVLGVYEEGVIYTSDVLRIDEEEFLGKLQKAYTNAFNLSVNAVIPTSATIETIVQKAFNDAKAVSVESAFITEKTADAILGKAHAQMIAVAKLAGDEALDDDLKEQISSSAVVATEEAPKAETKKEEKKEEAAPAAGLGLLF

>Mes82

MSDNPADLPVLVAGGGIGGLAAALALVRRGFSVKVLEQAPEIGEIGAGIQLGPNAFHAFDALGIGEKARGRAVYTDEMVMHDAIDGSLVGRIPTGEAFRQRFGNPYAVIHRVDVHLSLLEGAQETGKVEFLTSTRALRIEQDEGSVTVYDQHGNAHKGIALIGADGVKSVVREQFVGDAARVTGHVVYRAVVDKKDFPESLQWNAASIWVGPNCHLVHYPLRGGEQYNVVVTFHSRQPEQWGVTEGSKEEVQSYFQGICPQARQLIDLPKTWKRWATADREPIGQWSFGRVTLLGDAAHPTTQYMAQGACMAMEDGVTLGEALRVNNNDFPKAFELYQRSRVARTARIVLSSREMGRIYHAQGVERLVRNDLWKGRTPERFYDAMEWLYGWNVGNCLAKD

>Mes85

MTALKQTEKSTSLPMSFDFDIEGMTCASCVRRVEKAIAAVPGVASANVNLATERATVQFNGVPETTSVLRAVEKAGYAPRIVTEEIQIEGMTCASCVSRVEKALKAVPGVADASVNLATEKATVRLVSGSAEISALAAAVKGAGYGIRKATPAEAMKEDVDHRTAELRSLKSAVTISSLMTLPLFLLEMGSHFIPGVHDFIMGTIGMRNNLYLQFALATLVLFGPGLRFFRKGVPNLLRWTPDMNSLVVLGTTAAWGYSVVTTFVPAILPSGTANVYYEAAAVIVTLILVGRYLESRAKGRTSQAIKRLVGLQPKTAFVLHSGEFVETEITEVVTGDVIRIRPGEKIPVDGTVTDGSSYVDESMITGEPVPVQKATDSAVIGGTINKTGSITFKATKVGSDTLLAQIIRLVEAAQGSKLPIQALVDRVTAWFVPVVILAALLTFAAWYVLGPSPALSFALVNAVAVLIIACPCAMGLATPTSIMVGTGRAAELGILFRKGEALQSLRDADVVAVDKTGTLTKGRPELTDLVAAEGFEPDEVLCLVASLETLSEHPIAEAIVSAAKSRGIATVAVSAFEATPGFGVSGTVSGRRVLVGADRALVKNGIDITGFADEAERLGSGGKSPLYAAIDGRLAAIVAVSDPVKESTPQAIKSLHALGLKVAMVTGDNRRTAEAIAKKLGIDEVVAEVLPEGKVDAVRKLRQGGRSVAFIGDGINDAPALAEADVGIAVGTGTDIAIESADVVLMSGDLNGVAKALALSKATIRNIKQNLFWAFVYNISLVPVAAGVLYPVNGTLLSPIFAAAAMAMSSVFVLGNALRLKSFDPA

>Mes87

MNIGEASKVSGVSSKMIRYYEQIGLISPAVRTASSYRTYGDNDVHTLRFIRRARDLGFSVEQIKELLALWRDRSRASSDVKAVALEHIAELERKIAAIQDMTRTLKHLASHCHGDGRPDCPIIEEMAKGGGAAKTEINPRFGVASLK

>Mes92

MDLNLRKAIYSNISNNDEQQLEATIVDAIQNGEEKMLPGLGVLFELIWKHSDDQEKKEMIQSLESGVKQL

>Mes93

MSKRKMGPKQQKNPELPKSPEQPYGEPLSGSKKEKKANHSGQKHNPHHGL

>Mes98

MNNMPTIKHVRAFTVRGGGADYHDQGSGHWIDDHISTPMGRYPEYRQSRQSFGINVLGTLVVEIEASDGTVGFSVTTGGELGCWIVEKHLARFIEGAKVTDIEKIWDQMFNATLYYGRKGIVLNTISGVDLALWDLLAKVRKEPVHALLGGPVRDELTFYATGARPDLAKKMGFIGGKLPLHHGPAEREEGLKKNLELLGEMRQRVGDDFWLMYDCWMSLDVEYATRLANAASEYKLKWIEEALPPDDYWGYAELRRNVPRGMLVTTGEHEATRWGFRMLLEMECCDILQPDVGWCGGITELLKISALADAHGKLVVPHGSSVYSYHFVITRHNSPFSEFLMMAPKADEVVPMFNPMLLDEPVPVNGRMKASALDAPGFGVRLNPECALQRPFPR

>Mes99

ALFGTKDTTTAHSDYEIILEGGSSSWGQVKGRAKVNVPAAIPLLPTDCNIRIDAKPLDAQKGVVRFTTKIESVVDSVKNTLNVEVDIANETKDRRIAVGEGSLSVGDFSHSFSFEGQVVNMYYYRSDAVRRNIPNPIYMQGRQFHDILMKVPLDNNDLVDTWEGFQQSISGGGANFGDWIREFWFIGPAFAAINEGGQRISPIVVNSSNVEGGEKGPVGVTRWKFSHAGSGVVDSISRWTELFPVEQLNKPASIEGGFRSDSQGIEVKVDGNLPGVSRDAGGGLRRILNHPLIPLVHHGMVGKFNDFTVDTQLKIVLPKGYKIRYAAPQFRSQNLEEYRWSGGAYARWVEHVCKGGTGQFEVLYAQ

>Mes100

AVTKADVEQYDLANGKTVYDANCASCHAAGIMGAPKTGTARKWNSRLPQGLATMIEKSVAGYEGEYRGSKTFMPAKGGNPDLTDKQVGDAVAYMVNEVL

>Mes101

MSGGGVFTDILAAAGRIFEVMVEGHWETVGMLFDSLGKGTMRINRNAYGNLGGGGGSLRGSSPEVSGFAVPTKAVESKFAK

>Mes104

MKKSLIALAVLAASGAAMAQSSVTLFGIVDTNVAYVNKDAAGDSRYGLGTSGASTSRLGLRGTEDLGGGLKAGFWLEGEIFGDDGNASGFNFKRRSTVSLSGNFGEVRLGRDLVPTSQKLTSYDLFSATGIGPFMGFRNWAAGQGADDNGIRANNLISYYTPNFGGFNAGFGYAFDEKQTIGTADSVGRYIGGYVAYDNGPLSASLGLAQQKTAVGGLATDRDEITLGASYNFGVAKLSGLLQQTKFKRDIGGDIKTNSYMLGASAPVGGVGEVKLQYALYDQKAIDSKAHQITLGYVHNLSKRTALYGNLAFLKNKDASTLGLQAKGVYAGGVQAGESQTGVQVGIRHAF

>Mes105

MNEKATVIETDVLVVGSGPAGAASTLLLATYGVKTLCVSKYATTSRTPRSHITNQRTMEVMRDLGLELECEAMASPAELMGENVYCTSLVGDELGRVLTWGTHPQRRADYELASPTHMCDLPQNLLEPIMINHAARRGADVRFHTEFVSLKQDETGVTATVRDHLLDRQYDIRAKYLIGADGANSQVVDQVGLPMEGKMGVSGSINVVFEADLTKYVGHRPSVLYWVIQPGSSVGGLGIGVIRMVRPWNKWLCIWGYDIAGGPPDLNEAHARQIVHSLLGDSTIPVKIESTSTWTVNDMYATRLFDNRVFCMGDAVHRHPPTNGLGSNTSIQDAFNLCWKLSHVLQGKAGPELLATYNEERAPVARQVVQRANKSLGDFPPILAALGLFDTKDPEQMQRNIARLKEQSPEAQEQRAALRAAIDGTQYVYNAHGVEMNQRYQSAAIVPDGTPDPGFRRDSELYHAHSGRPGAPVPHVWVTRHGRRVSTLDLCGKGRFSLLSGIAGSPWVEAAVHAAESLGIDLDVHIIGPGQELEDLYGDFARVREIEESGALLVRPDNFICWRAMRWQEGSGDELRAALKRVLSVH

>Mes106

MKIEAISTTIVDVPTRRPLQMSFTTVHKQSYVIVQVTAGGLVGIGEGGSVGGPTWGSESAETIKVIIDNYLAPLLIGKDASNLSEARALMDRAVTGNLSAKAAIDIALHDLKARALNLSIADLIGGTMRKSIPIAWTLASGDTARDIDSALEMIEARRHNRFKVKLGARTPAQDLEHIRSIVKAVGDKASVRVDVNQGWDEQTASIWIPRLEEAGVELVEQPVPRANFGALRRLTEQNGVAILADESLSSLSSAFELARDRAVDAFSLKLCNMGGIANTLKVAAIAEAAGISSYGGTMLDSTVGTAAALHVYATLPSLPYGCELIGPWVLSDRLTQQDLEIKDFEVHLPVGSGLGVDLDHDKVRHYTRAA

>Mes110

MLPMNRNTLGLAVTIATVFVSSTVTAEVDFHGYVRAGIGISGENGQQVRYQSNKVGRLGNEDDLYSEILLGKELYSQDGKSFYVDSMMALLSDGSNDFEGTNTSCELLKNSAGDVDDVSCDNDAEFAIRQFNVQAKGLIPSNPDAVSWAGKRYYQRHDIHISDFYYWDTSGSGAGVENLSVGTGKLSLAVLRQDSGDINVNNFDIRYAEIALWQDANLELGFNYGLINETDAQKAEVGEDPLMLTAEITMANVIGGLNKTIFQYATESYGEQMAGLGAGNSPDATSDDGIDGYRIINWGVIAPTKTWEIGHQIVYANSSFDSQDDHSIFNVVVRPMYKWDENMRTVFEGGWFTEEDNKVDSSGSKFTVAQAWSAGSSFWARPELRVYASYLKDYENDNAFGIGNDTEYNLGVQVEAWW

>Mes111

MKKTLVALAILTAAGSANAGINLYDADGVKTDLSGAAEVQYRQTFKEDSDAELRMDDGDLAVNTTVAISDSLNAVAAVAFEFEDGKVTNDELWVGVAGDFGTLTAGRQYMLADDAGVGKDYELGGDGIDFVQANGDQVVKYVFDNGQFYGGVGALITETNPDNNADEASVYEGRLGARFGDFDVRAYLYSGEDVNTDNFDVFGDDKVNVDIDGYQIEAEYIVNAFAFAASFGQVDYELASDSSQKIEADTAALAGSYTMNKTTFAVGYTYWSPEAKGTVKKMEEANVFYANVTQQLHSNVKVYGEIGSSDTDNSEFGYVAGMEVTF

>Mes112

MNKKLIALAVAAASISSVATAAEVYSDETSSLAVGGRFEARAVLADVNKDENVTNTASSEVSDKSRVRINVAGKTDITEDFYGVGFFEKEFSSADSDNDETRYAYAGVGSQYGQLVYGKADGSLGMLTDFTDIMAYHGNEAGNKLAAADRTDNNLSYVGSFDLNGDNLTVKANYVFGGSDENEGYSAAAMYAMDMGLGFGAGYGEQDGQSSKNGNEDKTGKQAFGAISYTISDFYFSGLYQDSRNTVVNNDLIDESTGYEFAAAYTYGKAVFITTYNFLEDSNASGDASDLRDSIAIDGTYYFNKNFRTYASYKFNLLDANSSTTKAQASDEFVLGARYDF

>Mes113

MKLNKIITTAGLSLGLLLPSIATATPTTFVHLFEWNWQDVAQECEQYLGPKGYAAVQVSPPNEHITGSQWWTRYQPVSYELQSRGGNRAQFIDMVNRCSAAGVDIYVDTLINHMAAGSGTGTAGNSFGNKSFPIYSPQDFHESCTINNSDYGNDRYRVQNCELVGLADLDTASNYVQNTIAAYINDLQAIGVKGFRFDASKHVAASDIQSLMAKVNGSPVVFQEVIDQGGEAVGASEYLSTGLVTEFKYSTELGNTFRNGSLAWLSNFGEGWGFMPSSSAVVFVDNHDNQRGHGGAGNVITFEDGRLYDLANVFMLAYPYGYPKVMSSYDFHGDTDAGGPNVPVHNNGNLECFASNWKCEHRWSYIAGGVDFRNNTADNWAVTNWWDNTNNQISFGRGSSGHMAINKEDSTLTATVQTDMASGQYCNVLKGELSADAKSCSGEVITVNSDGTINLNIGAWDAMAIHKNAKLNTSSASSTESDWQRTVIFINAQTQSGQDMFIRGGIDHAYANANLGRNCQTSNFECAMPIRHNNLKNVTTSPWKANDNYLDWYGIENGQSSEAEGSATDWTTNVWPAGWGAEKTVNTDGFGVTPLNIWGEHYWMLDVDMDCSKAVNGWFELKAFIKNGQGWETAIAQDNAPYTSTNHMAQCGKINKFEFNNSGVVIRSF

>Mes114

MTSLQHIINRRDWENPITVQVNQVKAHSPLNGFKTIEDARENTQSQKKSLNGQWDFKLFDKPEAVDESLLYEKISKELSGDWQSITVPSNWQLHGFDKPIYCNVKYPFAVNPPFVPSDNPTGCYRTEFTITPEQLTQRNHIIFEGVNSAFHLWCNGQWVGYSQDSRLPSEFDLSELLVVGTNRIAVMVIRWSDGSYLEDQDMWWLSGIFRDVNLLTKPQSQIRDVFITPDLDACYRDATLHIKTAINAPNNYQVAVQIFDGKTSLCEPKIQSTNNKRVDEKGGWSDVVFQTIAIRSPKKWTAETPYLYRCVVSLLDEQGNTVDVEAYNIGFRKVEMLNGQLCVNGKPLLIRGVNRHEHHPENGHAVSTADMIEDIKLMKQNNFNAVRTAHYPNHPLFYELCDELGLYVVDEANIETHGMFPMGRLASDPLWAGAFMSRYTQMVERDKNHASIIIWSLGNECGHGANHDAMYGWSKSFDPSRPVQYEGGGANTTATDIICPMYSRVDTDIKDDAVPKYSIKKWLSLPGETRPLILCEYAHAMGNSLGSFDDYWQAFREYPRLQGGFIWDWVDQGLSKIDENGKHYWAYGGDFGDELNDRQFCINGLLFPDRTPHPSLFEAKYSQQHLQFTLREQNQNQNQNQYSIDVFSDYVFRHTDNEKLVWQLIQNGVCVEQGEMALNIAPQSTHTLTIKTKTAFEHGAQYYLNLDVALINDSHFANANHVMDSEQFKLINSNNLNSKSFASATEKSVISVNETDSHLSIENNTFKLVFNQQSGLIEQWLQDDTQVISSPLVDNFYRAPLDNDIGVSEVDNLDPNAWEARWSRAGIGQWQRTCSSINAVQSSVDVRITCVFNYEFNGVLQAQTQWLYTLNNTGTISLNVDVNLNDTLPPMPRIGLSTTINKQSDTKVNWLGLGPFENYPDRKSAARFGYYSLSLNELYTPYIFPTDNGLRSDCQLLSINNLIVTGAFLFAASEYSQNMLTQAKHTNELIADDCIHVHIDHQHMGVGGDDSWSPSTHKEYLLEQKNYNYSLTLTGGITT

>Mes116

MEKLAVLYAEHIATLQQRTRTICEQEGLEGLVIHSGQAKRQFLDDMYYPFKVNPHFKAWLPVIDNPHCWIVVNGSDKPKLIFYRPIDFWHKVPDEPRDFWAEYFDIELLLQPDQVEKLLPYDKAKFAYIGEYLEVAQALGFSIMNPEPVLNYIHYHRAYKTQYELECLRNANRIAVDGHKAARDAFFNGGSEFDIQQAYLMATRQSENEMPYGNIVALNENCAILHYTHFEPKAPQTHNSFLIDAGANFNGYAADITRTYDFKKQGEFADLVNAMTAHQIELGKSLKPGLLYGDLHIDCHNRIAQLLSDFDIVKLPAAEIVERQITSTFFPHGLGHHLGAQVHDVGGFMRDETGAHQAPPEGHPFLRCTRLIEKNQVFTIEPGLYFIDSLLGDLAQTDNKQFINWEKVEAFKPFGGIRIEDNIIVHEDSLENMTRNLLLD

>Mes118

MSNKLLSALFAAGFAVMMMSSASFAADETLAEFHVEMGGCENCHADGEPSKDGAYEFEQCQSCHGSLAEMDDNHKPHDGLLMCADCHAPHEAKVGEKPTCDTCHDDGRTAK

>Mes121

MKKMNLAVCIATLMGTAGLMGTAVAADNLAEFHVQNQECDSCHTPDGELSNDSLTYENTQCVSCHGTLAEVAETTKHEHYNAHASHFPGEVACTSCHSAHEKSMVYCDSCHSFDFNMPYAKKWLRDEPTIAELAKDKSERQAALASAPHDTVDVVVVGSGGAGFSAAISATDSGAKVILIEKEPVIGGNAKLAAGGMNAAWTDQQKAKKITDSPELMFEDTMKGGQNINDPALVKVLSSHSKDSVDWMTAMGADLTDVGMMGGASVNRAHRPTGGAGVGAHVVQVLYDNAVKRNIDLRMNTRGIEVLKDDKGTVKGILVKGMYKGYYWVKADAVILATGGFAKNNERVAKLDPSLKGFISTNQPGAVGDGLDVAENAGGALKDMQYIQAHPTLSVKGGVMVTEAVRGNGAILVNREGKRFVNEITTRDKASAAILAQTGKSAYLIFDDSVRKSLSKIDKYIGLGVAPTADSLVKLGKMEGIDGKALTETVARYNSLVSSGKDTDFERPNLPRALNEGNYYAIEVTPGVHHTMGGVMIDTKAEVMNAKKQVIPGLYGAGEVTGGVHGANRLGGNAISDIITFGRLAGEEAAKYSKKN

>Mes125

MTQTTHHTPDTARQADPFPVKGMDAVVFAVGNAKQAAHYYSTAFGMQLVAYSGPENGSRETASYVLTNGSARFVLTSVIKPATPWGHFLADHVAEHGDGVVDLAIEVPDARAAHAYAIEHGARSVAEPYELKDEHGTVVLAAIATYGKTRHTLVDRTGYDGPYLPGYVAAAPIVEPPAHRTFQAIDHCVGNVELGRMNEWVGFYNKVMGFTNMKEFVGDDIATEYSALMSKVVADGTLKVKFPINEPALAKKKSQIDEYLEFYGGAGVQHIALNTGDIVETVRTMRAAGVQFLDTPDSYYDTLGEWVGDTRVPVDTLRELKILADRDEDGYLLQIFTKPVQDRPTVFFEIIERHGSMGFGKGNFKALFEAIEREQEKRGNL

>Mes126

MTTVRIPAGWPATEEEARAVQDELRGRVILDEPGPPPGTGRVTGVDVAYDDERDVVVAAAVVLDAATLDVVAEATAVGEVSFPYVPGLLAFREIPTVLAALDALPCPPGLIVCDGYGVAHPRRFGLASHLGVLTGLPTIGVAKNPFTFSYEDPGAPRGSAAPLLAGADEVGRALRTQSGVKPVFVSVGHRVDLDHACAHTLALTPKYRIPETTRRADSLCRRALKEATA

>Mes132

MTHPIIHDLENRYTSKKYDPSKKVSQEDLAVLLEALRLSASSINSQPWKFIVIESDAAKQRMHDSFANMHQFNQPHIKACSHVILFANKLSYTRDDYDVVLSKAVADKRITEEQKEAAFASFKFVELNCDENGEHKAWTKPQAYLALGNALHTLARLNIDSTTMEGIDPELLSEIFADELKGYECHVALAIGYHHPSEDYNASLPKSRKAFEDVITIL

>Mes133

MKNINADDTYRIINKIKACRSNNDINQCLSDMTKMVHCEYYLLAIIYPHSMVKSDISILDNYPKKWRQYYDDANLIKYDPIVDYSNSNHSPINWNIFENNAVNKKSPNVIKEAKTSGLITGFSFPIHTANNGFGMLSFAHSEKDNYIDSLFLHACMNIPLIVPSLVDNYRKINIANNKSNNDLTKREKECLAWACEGKSSWDISKILGCSERTVTFHLTNAQMKLNTTNRCQSISKAILTGAIDCPYFKN

>Mes134

MFKGIVEGIGIIEKIDIYTDLDKYAIRFPENMLNGIKKESSIMFNGCFLTVTSVNSNIVWFDIFEKEARKLDTFREYKVGDRVNLGTFPKFGAASGGHILSARISCVASIIEIIENEDYQQMWIQIPENFTEFLIDKDYIAVDGISLTIDTIKNNQFFISLPLKIAQNTNMKWRKKGDKVNVELSNKINANQCW

>Mes135

MFRRIVPVLGLALGLGLASQAAMAQEQSPPPPPAVQGTPGKDFTGVSPANLAGIMNYCVEQQYVSYDEGNPVLYGLSEKYKATEQTVGNFDYALGTAGYFDSNGKRFYLVAYTNEDDRRAACHAAVKAAQPML

>Mes136

MLNALTRDRLVSEMKQGWKLAAAIGLMAVSFGAAHAQDADEALIKRGEYVARLSDCIACHTALHGQPYAGGLEIKSPIGTIYSTNITPDPEHGIGNYTLEDFTKALRKGIRKDGATVYPAMPYPEFARLSDDDIRAMYAFFMHGVKPVALQNKAPDISWPLSMRWPLGMWRAMFVPSMTPGVDKSISDPEVARGEYLVNGPGHCGECHTPRGFGMQVKAYGTAGGNAYLAGGAPIDNWIAPSLRSNSDTGLGRWSEDDIVTFLKSGRIDHSAVFGGMADVVAYSTQHWSDDDLRATAKYLKSMPAVPEGKNLGQDDGQTTALLNKGGQGNAGAEVYLHNCAICHMNDGTGVNRMFPPLAGNPVVITDDPTSLANVVAFGGILPPTNSAPSAVAMPGFKNHLSDQEMADVVNFMRKGWGNNAPGTVSASDIQKLRTTGAPVSTAGWNVSSKGWMAYMPQPYGEDWTFSPQTHTGVDDAQ

>Mes137

MTSGLLTPIKVTKKRLLSCAAALAFSAAVPVAFAQEDTGTAITSSDNGGHPGDWLSYGRSYSEQRYSPLDQINTENVGKLKLAWHYDLDTNRGQEGTPLIVNGVMYATTNWSKMKALDAATGKLLWSYDPKVPGNIADRGCCDTVSRGAAYWNGKVYFGTFDGRLIALDAKTGKLVWSVYTIPKEAQLGHQRSYTVDGAPRIAKGKVLIGNGGAEFGARGFVSAFDAETGKLDWRFFTVPNPENKPDGAASDDILMSKAYPTWGKNGAWKQQGGGGTVWDSLVYDPVTDLVYLGVGNGSPWNYKFRSEGKGDNLFLGSIVAINPDTGKYVWHFQETPMDEWDYTSVQQIMTLDMPVNGEMRHVIVHAPKNGFFYIIDAKTGKFITGKPYTYENWANGLDPVTGRPNYVPDALWTLTGKPWLGIPGELGGHNFAAMAYSPKTKLVYIPAQQIPLLYDGQKGGFKAYHDAWNLGLDMNKIGLFDDNDPEHVAAKKDFLKVLKGWTVAWDPEKMAPAFTINHKGPWNGGLLATAGNVIFQGLANGEFHAYDATNGNDLYSFPAQSAIIAPPVTYTANGKQYVAVEVGWGGIYPFLYGGVARTSGWTVNHSRVIAFSLDGKDSLPPKNELGFTPVKPVPTYDEARQKDGYFMYQTFCSACHGDNAISGGVLPDLRWSGAPRGRESFYKLVGRGALTAYGMDRFDTSMTPEQIEDIRNFIVKRANESYDDEVKARENSTGVPNDQFLNVPQSTADVPTADHP

>Mes139

MSHPDLFSLSGARALVTGASRGIGLTLAKGLARYGAEVVLNGRNAESLDSAQSGFEAEGLKASTAVFDVTDQDAVIDGVAAIERDMGPIDILINNAGIQRRAPLEEFSRKDWDDLMSTNVNAVFFVGQAVARHMIPRGRGKIVNICSVQSELARPGIAPYTATKGAVKNLTKGMATDWGRHGLQINGLAPGYFATEMTERLVADEEFTDWLCKRTPAGRWGQVEELVGAAVFLSSRASSFVNGQVLMVDGGITVSL

>Mes140

MITRETLKSLPANVQAPPYDIDGIKPGIVHFGVGNFFRAHEAFYVEQILEHAPDWAIVGVGLTGSDRSKKKAEEFKAQDCLYSLTETAPSGKSTVRVMGALRDYLLAPADPEAVLKHLVDPAIRIVSMTITEGGYNINETTGAFDLENAAVKADLQNPEKPSTVFGYVVEALRRRRDAGGKAFTVMSCDNLRHNGNVARKAFLGYAKARDPELAKWIEENATFPNGMVDRITPTVSAEIAKKLNAASGLDDDLPLVAEDFHQWVLEDRFANGRPPLEKAGVQLVDDVTDWEHVKIRMLNAGHITLCFPGILVGYENVDDAIEDKDLRGNLENYLNKDVIPTLKAPPGMTLEGYRDSVISRFSNKAMSDQTLRIASDGCSKIQVFWTETVRRAIECKRDLSRIAFGIASYLEMLRGRDEKGGTYESSEPTYGEAQKKLAKADDFESALKLPAFDGWRDLDTSELDQKVIALRKVIREKGVKAAIPA

>Mes141

MRRPYLLATAAGLALACSPLIAHAQFAPAGAGGEPSSSVPGPGNASEPTENSPKSQSYFAGPSPYAPQAPGVNAANLPDIESIDPSQVPAMAPQQSANPARGDWVAYGRDDHQTRYSPLSEITPENASKLKVAFVYHTGSYPRPGQVNKWAAETTPIKVGDGLYTCSAMNDIIKLDPATGKQIWRRNVDVKYHSIPYTAACKGVTYFTSSVVPEGQPCHNRLIEGTLDMRLIAVDAETGDFCPNFGHGGQVNLMQGLGESVPGFVSMTAPPPVINGVVVVNHEVLDGQRRWAPSGVIRGYDAESGKFVWAWDVNNSDDHSQPTGNRHYSRGTPNSWATMTGDNEEGLVYVPTGNSAADYYSALRSDAENKVSSAVVAIDVKTGSPRWVFQTAHKDVWDYDIGSQATLMDMPGPDGQTVPALIMPTKRGQTFVLDRRTGKPILPVEERPAPSPGVIPGDPRSPTQPWSVGMPALRVPDLKETDMWGMSPIDQLFCRIKFRRANYVGEFTPPSVDKPWIEYPGYNGGSDWGSMSYDPQSGILIANWNITPMYDQLVTRKKADSLGLMPIDDPNFKPGGGGAEGNGAMDGTPYGIVVTPFWDQYTGMMCNRPPYGMITAIDMKHGQKVLWQHPLGTARANGPWGLPTGLPWEIGTPNNGGSVVTGGGLIFIGAATDNQIRAIDEHTGKVVWSAVLPGGGQANPMTYEANGHQYVAIMAGGHHFMMTPVSDQLVVYALPDAIKQ

>Mes142

MPNLQGNRTLTEWLTLLLGVIVLLVGLFFVIGGADLAMLGGSTYYVLCGILLVASGVFMLMGRTLGAFLYLGALAYTWVWSFWEVGFSPIDLLPRAFGPTILGILVALTIPVLRRMESRRTLRGAV

>Mes146

MSVRKILRMGDPILRKISEPVTEDEIQTKEFKKLIRDMFDTMRHAEGVGLAAPQIGILKQIVVVGSEDNERYPGTPDVPERIILNPVITPLTKDTSGFWEGCLSVPGMRGYVERPNQIRMQWMDEKGNQFDETIDGYKAIVYQHECDHLQGILYVDRLKDTKLFGFNETLDSSHNVLD

>Mes149

MIINHNLSAVNAHRSLKFNELAVDKTMKALSSGMRINSAADDASGLAVSEKLRTQINGLRQAERNTEDGMSFIQTAEGFLEQTSNIIQRIRVLAIQTSNGIYSNEDRQLVQVEVSALVDEVDRIASQAEFNKFKLFEGQFARGSRVASMWFHMGPNQNQRERFYIGTMTSKALKLVKADGRPIAISSPGEANDVIGLADAALTKIMKQRADMGAYYNRLEYTAKGLMGAYENMQASESRIRDADMAEEVVSLTTKQILVQSGTAMLAQANMKPNSVLKLLQQI

>Mes150

MNETGSIGIIETKYAEFKELILNNGSVLSPVVIAYETYGTLSSSKNNAILICHALSGDAHAAGYHSGSDKKPGWWDDYIGPGKSFDTNQYFIICSNVIGGCKGSSGPLSIHPETSTPYGSRFPFVSIQDMVKAQKLLVESLGIEKLFCVAGGSMGGMQALEWSIAYPNSLSNCIVMASTAEHSAMQIAFNEVGRQAILSDPNWKNGLYDENSPRKGLALARMVGHITYLSDDKMREKFGRNPPRGNILSTDFAVGSYLIYQGESFVDRFDANSYIYVTKALDHYSLGKGKELTAALSNATCRFLVVSYSSDWLYPPAQSREIVKSLEAADKRVFYVELQSGEGHDSFLLKNPKQIEILKGFLENPN

>Mes151

MRRDWQNFLMACGIKNFDDSELNPLDITIAVYENEEIIGTGSIAGDVIKYVAVQETTMSGHSTLFNQLMTKLENFMAVEGRFHQFVLRNQFTKKVLNTLASKRWLSVNKEFCWKKDYQILRNTCQQFPSQTPIDKVASVVINANPFTNGHRFLIEEASRNNELVYVFVLNQEASLFHTDERIALVKAGVQDLSNVIVVNGGAYIISYLTFPAYFLKHNDSAIDYQTTIDVRLFKYKIASALGITSRYVGSEPLSHTTNLYNQKLISELNPQIEVHVIQRKLAAGDLGVISARTVREAIDKGDEAVWQKMVTETTQHFISNNLLELQQRIRKGQKINGN

>Mes152

MTKKRVALIFGGNSSEHDVSKRSAQNFYNAIEATGKYEIIVFAIAQNGFFLDTESSKKILALEDEQPIVDAFMKTVDASDPLARIHALKSAGDFDIFFPVVHGNLGEDGTLQGLFKLLDKPYVGAPLRGHAVSFDKALTKELLTVNGIRNTKYIVVDPESANNWSWDKIVAELGNIVFVKAANQGSSVGISRVTNAEEYTEALSDSFQYDYKVLIEEAVNGARELEVGVIGNDQPLVSEIGAHTVPNQGSGDGWYDYNNKFVDNSAVHFEIPAQLSPEVTKEVKQMALDAYKVLNLRGEARMDFLLDENNVPYLGEPNTLPGFTNMSLFKRLWDYSDINNAKLVDMLIDYGFEDFAQNKKLSYSFVSLGEEKIGKFN

>Mes154

MVSEIKTLVTFFGGTGDLAKRKLYPSVFNLYKKGYLQKHFAIVGTARQALNDDEFKQLVRDSIKDFTDDQAQAEAFIEHFSYRAHDVTDAASYAVLKEAIEEAADKFDIDGNRIFYMSVAPRFFGTIAKYLKSEGLLADTGYNRLMIEKPFGTSYDTAAELQNDLENAFDDNQLFRIDHYLGKEMVQNIAALRFGNPIFDAAWNKDYIKNVQVTLSEVLGVEERAGYYDTAGALLDMIQNHTMQIVGWLAMEKPESFTDKDIRAAKNAAFNALKIYDEAEVNKYFVRAQYGAGDSADFKPYLEELDVPADSKNNTFIAGELQFDLPRWEGVPFYVRSGKRLAAKQTRVDIVFKAGTFNFGSEQEAQEAVLSIIIDPKGAIELKLNAKSVEDAFNTRTIDLGWTVSDEDKKNTPEPYERMIHDTMNGDGSNFADWNGVSIAWKFVDAISAVYTADKAPLETYKSGSMGPEASDKLLAANGDAWVFKG

>Mes155

KGKGFWSWASKATSWLTGPQQPGSPLLKKHR

>Mes156

KNYGNGVHCTKKGCSVDWGYAWTNIANNSVMNGLTGGNAGWHN

>Mes157

MTNMKSVEAYQQLDNQNLKKVVGGKYYGNGVHCTKSGCSVNWGEAASAGIHRLANGGNGFW

>Mes158

MEIQNKAMLITYADSLGKNLKDVHQVLKEDIGDAIGGVHLLPFFPSTGDRGFAPADYTRVDAAFGDWADVEALGEEYYLMFDFMINHISRESVMYQDFKKNHDDSKYKDFFIRWEKFWAKAGENRPTQADVDLIYKRKDKAPTQEITFDDGTTENLWNTFGEEQIDIDVNSAIAKEFIKTTLEDMVKHGANLIRLDAFAYAVKKVDTNDFFVEPEIWDTLNEVREILTPLKAEILPEIHEHYSIPKKINDHGYFTYDFALPMTTLYTLYSGKTNQLAKWLKMSPMKQFTTLDTHDGIGVVDARDILTDDEIDYASEQLYKVGANVKKTYSSASYNNLDIYQINSTYYSALGNDDAAYLLSRVFQVFAPGIPQIYYVGLLAGENDIALLESTKEGRNINRHYYTREEVKSEVKRPVVANLLKLLSWRNESPAFDLAGSITVDTPTDTTIVVTRQDENGQNKAVLTADAANKTFEIVENGQTVMSSDNLTQN

>Mes159

MLILTRRVGETLMIGDEVTVTVLGVKGNQVRIGVNAPKEVSVHREEIYQRIQAEKSQPTSY

>Mes161

MKRKLLTTSIALSLAMLATPSYSVDFSGYFRSGVGVSNHGKQQTADKSYVGRLGNEDDTYGEIQLGQQLYNENGKTFYFDSMISMFSNSSNDNETTKNDDAEFGLRQLNLQAKGFVPGLPDATVWAGKRYYQRHDLHIIDTKYWNISGAGAGIENVKAGEGAFSFAWIRADAENMDVDCGNSLNSQECSSREDTYNDLNINYLDARYAGWKPWDGAWTEFGISYAMPNEADTQKNIFLAEGQKFDPKNSMMITGELSHYFSGLKSNQKLVLQYADKGLAHNMVDQGGGWYDVWSINDSAKGYRVIQAGDLPITDHISLSHVLTYGKADEISRWRDSTELLSAVGRGQYAWTKNQKTYLEAGTYQKKDSWKAGTETKYSGQKYTLAHAFSADIPMLTRPELRFFVSYLDGGNENRNRFNDDRSNTVNFGIQAEAWW

>Mes162

MNGENLNLGTLVSETRNPATMTLDQLSTLEMMQVFNQEDRKVPEAIAQVLPAIAEAVDLATASLQAGGRLIYLGAGTSGRLGVLDASECPPTFGVPHGLVIGLIAGGPGALLKAVEGAEDDPALGEADLKALSLTATDMVIGLAASGRTPYVIGALRYARDVGCRTAAISCNPHSPIAQEAQVAISPVVGPEALTGSTRLKSGTAQKLVLNMISTGVMVKLGKVYQNLMVDVKATNVKLLDRACRIVVEATGADLDSARQALEQSDNDVKPAILMLLANIGVEAARERLKQHNGYLREALLGG

>Mes163

MKYLLPSAAAGLLLLAAQPTMAANTGGYATTDGGDVSGAVKKTARSLQEIVDIIEAAKKDSSGKVVKGGAFPLVITYNGNEDALIKAAEANICGQWSKDPRGVEIKEFTKGITILGTNGSSANFGIWVVNSSNVVVRNMRFGYMPGGAKDGDAIRIDNSPNVWIDHNEIFAKNFECAGTPDNDTTFESAVDIKKASTNVTVSYNFIHGVKKVGLSGSSNTDTGRNLTYHHNIYSDVNSRLPLQRGGQVHAYNNLYDGIKSSGFNVRQKGIALIESNWFENALNPVTARNDDSNFGTWELRNNNITSPSDFAKYKITWGKPSTPHINADDWKSTGKFPAVSYSYSPVSAQCVKDKLANYAGVGKNQAVLTAANCK

>Mes165

MKRAVVVFSGGQDSTTCLIQALQDYDDVHCITFDYGQRHRAEIEVAQELSQKLGAAAHKVLDVGLLNELATSSLTRDSIPVPDYDANAQGIPNTFVPGRNILFLTLASIYAYQVGAEAVITGVCETDFSGYPDCRDEFVKALNQAIVLGIARDIRFETPLMWLNKAETWALADYYQQLDTVRYHTLTCYNGIKGDGCGQCAACHLRANGLAQYQKDAATVMASLKQKVGLR

>Mes167

MSTQSVSSHNIESSSFSANQHAAEPSVATGGIISEIVYNADQPIVTHLLLPLLQQLGTQSRWLLWLSPQQRLSRPWVQQSGLPLDKMVQLHHINPLFTVDAMERALLTGNYSAVLCWLPHELTEEEKVRLRHAAQAGNTYGFIMRPESAGDDAYRLFPSLKIHSTLYH

>Mes168

MQKIKLTGLSLIISGLLMAQAQAAEPVYPDQLRLFSLGQGVCGDKYRPVNREEAQSVKSNIVGMMGQWQISGLANGWVIMGPGYNGEIKPGTASNTWCYPTNPVTGEIPTLSALDIPDGDEVDVQWRLVHDSANFIKPTSYLAHYLGYAWVGGNHSQYVGEDMDVTRDGDGWVIRGNNDGGCDGYRCGDKTAIKVSNFAYNLDPDSFKHGDVTQSDRQLVKTVVGWAVNDSDTPQSGYDVTLRYDTATNWSKTNTYGLSEKVTTKNKFKWPLVGETELSIEIAANQSWASQNGGSTTTSLSQSVRPTVPARSKIPVKIELYKADISYPYEFKADVSYDLTLSGFLRWGGNAWYTHPDNRPNWNHTFVIGPYKDKASSIRYQWDKRYIPGEVKWWDWNWTIQQNGLSTMQNNLARVLRPVRAGITGDFSAESQFAGNIEIGAPVPLAADSKVRRARSVDGAGQGLRLEIPLDAQELSGLGFNNVSLSVTPAANQ

>Mes169

MMKGWMKCGLAGAVVLMASFWGGSVRAAGMSLTQVSGPVYVVEDNYYVQENSMVYFGAKGVTVVGATWTPDTARELHKLIKRVSRKPVLEVINTNYHTDRAGGNAYWKSIGAKVVSTRQTRDLMKSDWAEIVAFTRKGLPEYPDLPLVLPNVVHDGDFTLQEGKVRAFYAGPAHTPDGIFVYFPDEQVLYGNCILKEKLGNLSFADVKAYPQTLERLKAMKLPIKTVIGGHDSPLHGPELIDHYEALIKAAPQS

>Mes171

MPNFFRNGCIALVGSVAAMGAAHAEGGIAEAAGKALDSAQSDVTITAPKVMMVVATVVGVGILINMMRKA

>Mes172

MKKWFVCLLGLVALTVQAADSRPAFSRIVMFGDSLSDTGKMYSKMRGYLPSSPPYYEGRFSNGPVWLEQLTNEFPGLTIANEAEGGPTAVAYNKISWNPKYQVINNLDYEVTQFLQKDSFKPDDLVILWVGANDYLAYGWNTEQDAKRVRDAISDAANRMVLNGAKEILLFNLPDLGQNPSARSQKVVEAASHVSAYHNQLLLNLARQLAPTGMVKLFEIDKQFAEMLRDPQNFGLSDTENACYGGSYVWKPFASRSASTDSQLSAFNPQERLAIAGNPLLAQAVASPMAARSASTLNCEGKMFWDQVHPTTVVHAALSEPAATFIESQYEFLAH

>Mes176

MKKKLIYAAVVSALLAGCGGSDDNKGDTSSYLDYLLTGSNAVGPSALAARAWDGTLKFSTETADLSNPVSAMSTLDGWSTTQAIQIVPVTSSGITVQAPTTAEFGASVAPLYLLEVTFDSTALRPSGVKKVLTYGVDFVVAASAWQAEPGSAQAVEPLPCLANDSGHRTAERQSRRCLKAGSDYGNYKNNAGSNAQEQTINGLIALQEGLFKAATGIATDHVIFSDWFGTQSGADVLVAVKGAAASVLKADPVTLDAAKLWKQDAWEHQPARHLYPGRDRPTCLPDPAGCRAVPAAEQKDAIATAFGPVLRSTRLLKRPRSIPVPSSCLTSSPHRRPQVPGARPRPSPGTVPSQPVRHRQCAEGVTRSDRRAGGGGRGSGPAGDADCRSDPPERAAGRGEQADWGDAHLRRQAAGRRAEHWSLQPAADAGRGAIRADACLRQGCPQHHHGCHHLSARRDLGQRERLRPGAGPDLEDLCRHAGGQEGGAGGDRSSAARRAWLRLSGSMDTVTTSDNPTPYLNLSYLTVARDNLKQSVAICWACVWRLAWPTPRAIGTAGSLKVHFLGHSLGASRVPTCCGRQPDHRQRASGCPVQVRYRWPGHAGSHSAAAAELADFGPTIKMGVLTSGSAELKAGFTAYAPNCTDGGAYLLRQRVPAEPGRGHSATAATRCRVQLCGPVGAGFG

>Mes177

MSGKARLHYPVTRQSEQLDHYFGQAVADPYRWLEDDRSPETEAWVKAQNRVTQDYLAQIPFRDAIKGKLATSWNYAKEGAPFREGRYHYFFKNDGLQNQNVLCGQLAGKPAEVFLDPNLLSPDGTTALDQLSFSRDGKTLAYSLSLAGSDWREIHLMDVESKQPLETPLRDVKFSGISWLGNEGFFYSSYDKPDGSELSARTDQHKLYFHRLGTAQEEDRLVFGAIPAQRHRYVGATVTEDDRYLLISAADSTSGNRLYVKDLTREGAPLLTVQGDLAADVSLVDNKGSRLYLLTNRDAPNRRLVTVEADNPGPEQWRDLIPERQQVLTVHSGGGYLFAEYMVDATARVEQFDHDGKRVREVGLPGLGSVSGFNGKQDDPALYFGFENYAQPPTLYKFEPNSGAISLYRASAAPFKPEDYVSEQRFYRSKDGTRVPLIISYRKGLKLDGSNPTILYGYGGFDVSLTPSFSVSVANWLDLGGVYAVANLRGGGEYGQAWHLAGTRMNKQNVFDDFIAAAEYLKAEGYTRTDRLAIRGGSNGGLLVGAVMTQRPDLMRVACQAVGVLDMLRYHTFTAGAGWAYDYGTSADSEAMFDYLKGYSPLHSVRAGVSYPSTLVTTADHDDRVVPAHSFKFAATLQADDAGPHPQLIRIETNAGHGAGTPVAKLIEQSADIYAFTLFEMGYRQLPRQP

>Mes178

MKQQEVRQRAFAMPLTSPAFPPGPYRFVNREYMIITYRTDPAAIEAVLPEPLQMAEPVVRYEFIRMPDSTGFGDYSESGQVIPVTFRGERGSYTLAMFLDDQPPLAGGRELWGFPKKAGKPRLEVHQDTLVGSLDFGPVRIATGTMGYKYEALDRSALLASLAEPNFLLKIIPHVDGSPRICELVRYHTTDVAIKGAWSAPGSLELHPHALAPVAALPVLEVLSARHFVCDLTLDLGTVVFDYLRQ

>Mes179

MTHSPSFLQHALSSSDTRAEWPLPGGLAARWLAPGCVELNGDARGADSVLLSCGVHGNETAPIEVVDGMLTDIAAGQLALNCRLLVMFANLDAIRQGVRYGNYDMNRLFNGAHARHPELPESVRAAELETLAAEFFAGARARKLHYDLHTAIRGSVFEKFAIYPFLHDGRTHKREQLAWLQRCGIEAVLLHTQPANTFSYFTSQYCEADAFTLELGKARPFGQNDLSRFSGIDGALRGLLSNPQANVPDLDEDKLPLFRAKYDLVKHSEAFKLNLADSVENFTLLPDGMLIAEDGAVRYQATGGEERILFPNPAVKPGLRAGIVVEPARLPSR

>Mes181

MTPDVLKNIADTLEARREAAPQSSYVASLFHKGEDAILKKVAEEAAETLMASKDKDKLHLVREVADLWFHTMVLLTYHGLRPEDVVMELHRREGISGLDEKASRKPTA

>Mes182

MNDRADFVVPDITTRKNVGLSHDANDFTLPQPLDRYSAEDHATWATLYQRQCKLLPGRACDEFMEGLERLEVDADRVPDFNKLNQKLMAATGWKIVAVPGLIPDDVFFEHLANRRFPVTWWLREPHQLDYLQEPDVFHDLFGHVPLLINPVFADYLEAYGKGGVKAKALGALPMLARLYWYTVEFGLINTPAGMRIYGAGILSSKSESIYCLDSASPNRVGFDLMRIMNTRYRIDTFQKTYFVIDSFKQLFDATAPDFAPLYLQLADAQPWGAGDVAPDDLVLNAGDRQGWADTEDV

>Mes183

MKHSSDICIVGAGISGLTCASHLLDSPACRGLSLRIFDMQQEAGGRIRSKMLDGKASIELGAGRYSPQLHPHFQSAMQHYSQKSEVYPFTQLKFKSHVQQKLKRAMNELSPRLKEHGKESFLQFVSRYQGHDSAVGMIRSMGYDALFLPDISAEMAYDIVGKHPEIQSVTDNDANQWFAAETGFAGLIQGIKAKVKAAGARFSLGYRLLSVRTDGDGYLLQLAGDDGWKLEHRTRHLILAIPPSAMAGLNVDFPEAWSGARYGSLPLFKGFLTYGEPWWLDYKLDDQVLIVDNPLRKIYFKGDKYLFFYTDSEMANYWRGCVAEGEDGYLEQIRTHLASALGIVRERIPQPLAHVHKYWAHGVEFCRDSDIDHPSALSHRDSGIIACSDAYTEHCGWMEGGLLSAREASRLLLQRIAA

>Mes184

MSILDFPRIHFRGWARVNAPTANRDPHGHIDMASNTVAMAGEPFDLARHPTEFHRHLRSLGPRFGLDGRADPEGPFSLAEGYNAAGNNHFSWESATVSHVQWDGGEADRGDGLVGARLALWGHYNDYLRTTFNRARWVDSDPTRRDAAQIYAGQFTISPAGAGPGTPWLFTADIDDSHGARWTRGGHIAERGGHFLDEEFGLARLFQFSVPKDHPHFLFHPGPFDSEAWRRLQLALEDDDVLGLTVQYALFNMSTPPQPNSPVFHDMVGVVGLWRRGELASYPAGRLLRPRQPGLGDLTLRVSGGRVALNLACAIPFSTRAAQPSAPDRLTPDLGAKLPLGDLLLRDEDGALLARVPQALYQDYWTNHGIVDLPLLREPRGSLTLSSELAEWREQDWVTQSDASNLYLEAPDRRHGRFFPESIALRSYFRGEARARPDIPHRIEGMGLVGVESRQDGDAAEWRLTGLRPGPARIVLDDGAEAIPLRVLPDDWALDDATVEEVDYAFLYRHVMAYYELVYPFMSDKVFSLADRCKCETYARLMWQMCDPQNRNKSYYMPSTRELSAPKARLFLKYLAHVEGQARLQAPPPAGPARIESKAQLAAELRKAVDLELSVMLQYLYAAYSIPNYAQGQQRVRDGAWTAEQLQLACGSGDRRRDGGIRAALLEIAHEEMIHYLVVNNLLMALGEPFYAGVPLMGEAARQAFGLDTEFALEPFSESTLARFVRLEWPHFIPAPGKSIADCYAAIRQAFLDLPDLFGGEAGKRGGEHHLFLNELTNRAHPGYQLEVFDRDSALFGIAFVTDQGEGGALDSPHYEHSHFQRLREMSARIMAQSAPFEPALPALRNPVLDESPGCQRVADGRARALMALYQGVYELMFAMMAQHFAVKPLGSLRRSRLMNAAIDLMTGLLRPLSCALMNLPSGIAGRTAGPPLPGPVDTRSYDDYALGCRMLARRCERLLEQASMLEPGWLPDAQMELLDFYRRQMLDLACGKLSREA

>Mes185

MKRAIIVGGGLAGGLTAIYLAKRGYEVHVVEKRGDPLRDLSSYVDVVSSRAIGVSMTVRGIKSVLAAGIPRAELDACGEPIVAMAFSVGGQYRMRELKPLEDFRPLSLNRAAFQKLLNKYANLAGVRYYFEHKCLDVDLDGKSVLIQGKDGQPQRLQGDMIIGADGAHSAVRQAMQSGLRRFEFQQTFFRHGYKTLVLPDAQALGYRKDTLYFFGMDSGGLFAGRAATIPDGSVSIAVCLPYSGSPSLTTTDEPTMRAFFDRYFGGLPRDARDEMLRQFLAKPSNDLINVRSSTFHYKGNVLLLGDAAHATAPFLGQGMNMALEDARTFVELLDRHQGDQDKAFPEFTELRKVQADAMQDMARANYDVLSCSNPIFFMRARYTRYMHSKFPGLYPPDMAEKLYFTSEPYDRLQQIQRKQNVWYKIGRVN

>Mes186

MKILVIGAGPAGLVFASQLKQARPLWAIDIVEKNDEQEVLGWGVVLPGRPGQHPANPLSYLDAPERLNPQFLEDFKLVHHNEPSLMSTGVLLCGVERRGLVHALRDKCRSQGIAIRFESPLLEHGELPLADYDLVVLANGVNHKTAHFTEALVPQVDYGRNKYIWYGTSQLFDQMNLVFRTHGKDIFIAHAYKYSDTMSTFIVECSEETYARARLGEMSEEASAEYVAKVFQAELGGHGLVSQPGLGWRNFMTLSHDRCHDGKLVLLGDALQSGHFSIGHGTTMAVVVAQLLVKALCTEDGVPAALKRFEERALPLVQLFRGHADNSRVWFETVEERMHLSSAEFVQSFDARRKSLPPMPEALAQNLRYALQR

>Mes188

MTKKIKCALIGPGNIGTDLLAKLQRSPVLEPIWMVGIDPESDGLKRAREMGIKTTADGVDGLIPHMQADGVQIVFDATSAYVHADNSRKVNALGALMIDLTPAAIGPFCVPTVNLKEHVGKGEMNVNMVTCGGQATIPMVAAVSRVQPVAYGEIVATVSSKSAGPGTRKNIDEFTRTTAGAVEKVGGAKKGKAIIILNPAEPPLIMRDTVHCLLESEPDQAKITESIHAMIKEVQKYVPGYKLVNGPVFDGLRVSVYLEVEGLGDYLPKYAGNLDIMTAAAARTAEMFAEEILAGQLTLQPVHA

>Mes189

MSSAIKEVQGAPVKWVTNWTPEAIRGLVDQEKGLLDPRIYADQSLYELELERVFGRSWLLLGHESHVPETGDFLATYMGEDPVVMVRQKDKSIKVFLNQCRHRGMRICRSDAGNAKAFTCSYHGWAYDIAGKLVNVPFEKEAFCDKKEGDCGFDKAEWGPLQARVATYKGLVFANWDVQAPDLETYLGDARPYMDVMLDRTPAGTVAIGGMQKWVIPCNWKFAAEQFCSDMYHAGTTTHLSGILAGIPPEMDLSQAQIPTKGNQFRAAWGGHGSGWYVDEPGSLLAVMGPKVTQYWTEGPAAELAEQRLGHTGMPVRRMVGQHMTIFPTCSFLPTFNNIRIWHPRGPNEIEVWAFTLVDADAPAEIKEEYRRHNIRNFSAGGVFEQDDGENWVEIQKGLRGYKAKSQPLNAQMGLGRSQTGHPDFPGNVGYVYAEEAARGMYHHWMRMMSEPSWATLKP

>Mes190

MKLKGEAVLITGGASGLGRALVDRFVAEGAKVAVLDKSAERLAELETDHGDNVLGIVGDVRSLEDQKQAASRCVARFGKIDTLIPNAGIWDYSTALVDLPEESLDAAFDEVFHINVKGYIHAVKACLPALVASRGNVIFTISNAGFYPNGGGPLYTAAKHAIVGLVRELAFELAPYVRVNGVGSGGINSDLRGPSSLGMGSKAISTVPLADMLKSVLPIGRMPEVEEYTGAYVFFATRGDAAPATGALLNYDGGLGVRGFFSGAGGNDLLEQLNIHP

>Mes191

MSIRSLGYMGFAVSDVAAWRSFLTQKLGLMEAGTTDNGDLFRIDSRAWRIAVQQGEVDDLAFAGYEVADAAGLAQMADKLKQAGIAVTTGDASLARRRGVTGLITFADPFGLPLEIYYGASEVFEKPFLPGAAVSGFLTGEQGLGHFVRCVPDSDKALAFYTDVLGFQLSDVIDMKMGPDVTVPAYFLHCNERHHTLAIAAFPLPKRIHHFMLEVASLDDVGFAFDRVDADGLITSTLGRHTNDHMVSFYASTPSGVEVEYGWSARTVDRSWVVVRHDSPSMWGHKSVRDKAAARNKA

>Mes192

MTALTESSTSKFVKINEKGFSDFNIHYNEAGNGETVIMLHGGGPGAGGWSNYYRNVGPFVDAGYRVILKDSPGFNKSDAVVMDEQRGLVNARAVKGLMDALDIDRAHLVGNSMGGATALNFALEYPDRIGKLILMGPGGLGPSMFAPMPMEGIKLLFKLYAEPSYETLKQMLQVFLYDQSLITEELLQGRWEAIQRQPEHLKNFLISAQKAPLSTWDVTARLGEIKAKTFITWGRDDRFVPLDHGLKLLWNIDDARLHVFSKCGHWAQWEHADEFNRLVIDFLRHA

>Mes193

MTNPSPHFFKTFEWPSKAAGLELQNEIEQFYYREAQLLDHRAYEAWFALLDKDIHYFMPLRTNRMIREGELEYSGDQDLAHFDETHETMYGRIRKVTSDVGWAENPPSRTRHLVSNVIVKETATPDTFEVNSAFILYRNRLERQVDIFAGERRDVLRRADNNLGFSIAKRTILLDASTLLSNNLSMFF

>Mes194

MKFTRVCDRRDVPEGEALKVESGGTSVAIFNVDGELFATQDRCTHGDWSLSDGGYLEGDVVECSLHMGKFCVRTGKVKSPPPCEALKIFPIRIEDNDVLVDFEAGYLAP

>Mes196

MKLEGKKVTVHDMTLRDGMHPKRHQMTLEQMKSIACGLDAAGIPLIEVTHGDGLGGSSVNYGFPAHSDEEYLGAVIPLMKQAKVSALLLPGIGTVEHLKMAKDLGVNTIRVATHCTEADVSEQHITQSRKLGLDTVGFLMMAHMASPEKLVSQALLMQGYGANCIYVTDSAGYMLPDDVKARLSAVRAALKPETELGFHGHHNLAMGVANSIAAIEAGATRIDAAAAGLGAGAGNTPMEVFIAVCARMGIETGVDVFKIQDVAEDLVVPIMDHVIRIDRDSLTLGYAGVYSSFLLFAKRASAKYGVPARDILVELGRRGMVGGQEDMIEDTAMTMARERGLTLTAA

>Mes198

MKSSEPASVSAAERRAETFQHKLEQFNPGIVWLDQHGRVTAFNDVALQILGPAGEQSLGVAQDSLFGIDVVQLHPEKSRDKLRFLLQSKDVGGCPVKSPPPVAMMINIPDRILMIKVSSMIAAGGACGTCMIFYDVTDLTTEPSGLPAGGSAPSPRRLFKIPVYRKNRVILLDLKDIVRFQGDGHYTTIVTRDDRYLSNLSLADLELRLDSSIYLRVHRSHIVSLQYAVELVKLDESVNLVMDDAEQTQVPVSRSRTAQLKELLGVV

>Mes200

MDRKEIFERIEQVLAEQLGIPAEQITEEADLREDLGMDSLDLVELVSALEDEVGMRVEQSQLEGIETVGHVMELTLDLVARLATASAADKPEAAS

>Mes201

MTTAIASDRLKKRFDRWDFDGNGALERADFEKEAQHIAEAFGKDAGAAEVQTLKNAFGGLFDYLAKEAGVGSDGSLTEEQFIRVTENLIFEQGEASFNRVLGPVVKGTWGMCDKNADGQINADEFAAWLTALGMSKAEAAEAFNQVDTNGNGELSLDELLTAVRDFHFGRLDVELLG

>Mes202

EAAGVVSASPYLYNGWGNPPSPTEVMNASGIKNFTLAFILADGTCNPAWDGNRPLDGQDKATIDAIRGAGGDVIPSIGGYSGSKLGEVCQDSQSLAGAYQKVIDAYGLKAIDVDIEATEFENDASETRVLEALKIVKEANPGLRTVVTFPTLVNGPNDVGKRMIDKAARIGSDVDVWTQMPFNFGGGDMAADTITSTEGLVAHLKSAFGYDDATAYAHAGISSMNGKSDTGETVDQAAFQKMADYAGEKGLGRLSFWSVNRDRPCDGAPDACGGIDQQWDFTKIVAGLQS

>Mes204

MTTGEVPDLLAFDDAFAQDRHNRYARMREEPVQRIRTVNGLDAWLITRYEDVKQALLDPRIAKDFGRTQQIIEKRLADAERRPGFSPDLGPHMLNTDPPDHTRLRKLVVKAFTARRVEGLRPRIEQITDDLLDRLAGRSEVDLIDEFAFPLPITVISELMGVEDSRRDDFRSWTNVLVDGSQPEAQAQASVAMVEYLTELIAKKRTEPGDDLLTALLEAVEDGDRLSEGELIAMVFLLLVAGHETTVNLIGNCVLSLLGNPDQLAALRNDPSLLPGAIEETLRYESPVANGTFRHTAEAVRFGDVVIPEGELVWVALGAANRDGERFEDPDRFDITRETTGHVAFGHGIHFCVGAALARLEAQIAVGRLLERFPDLRMAASPDDLRWRFSVLMRGLEKLPVRPGA

>Mes205

MTTIDEVPGMADETALLDWLGTMREKQPVWQDRYGVWHVFRHADVQTVLRDTATFSSDPTRVIEGASPTPGMIHEIDPPEHRALRKVVSSAFTPRTISDLEPRIRDVTRSLLADAGESFDLVDVLAFPLPVTIVAELLGLPPMDHEQFGDWSGALVDIQMDDPTDPALAERIADVLNPLTAYLKARCAERRADPGDDLISRLVLAEVDGRALDDEEAANFSTALLLAGHITTTVLLGNIVRTLDEHPAHWDAAAEDPGRIPAIVEEVLRYRPPFPQMQRTTTKATEVAGVPIPADVMVNTWVLSANRDSDAHDDPDRFDPSRKSGGAAQFSFGHGVHFCLGAPLARLENRVALEEIIARFGRLTVDRDDERLRHFEQIVLGTRHLPVLAGSSPRQSA

>Mes206

MSGPRSRTTSRRTPVRIGAVVVASSTSELLDGLAAVADGRPHASVVRGVARPSAPVVFVFPGQGAQWAGMAGELLGESRVFAAAMDACARAFEPVTDWTLAQVLDSPEQSRRVEVVQPALFAVQTSLAALWRSFGVTPDAVVGHSIGELAAAHVCGAAGAADAARAAALWSREMIPLVGNGDMAAVALSADEIEPRIARWDDDVVLAGVNGPRSVLLTGSPEPVARRVQELSAEGVRAQVINVSMAAHSAQVDDIAEGMRSALAWFAPGGSEVPFYASLTGGAVDTRELVADYWRRSFRLPVRFDEAIRSALEVGPGTFVEASPHPVLAAALQQTLDAEGSSAAVVPTLQRGQGGMRRFLLAAAQAFTGGVAVDWTAAYDDVGPNPALCRSSRRPRRKTSRPSPASTGTRHRTCCERLLAVVNGETAALAGREADAEATFRELGLDSVLAAQLRAKVSAAIGREVNIALLYDHPTPRALAEALAAGTEVAQRETRARTNEAAPGEPVAVVAMACRLPGGVSTPEEFWELLSEGRDAVAGLPTDRGWDLDSLFHPDPTRSGTAHQRGGGFLTEATAFDPAFFGMSPREALAVDPQQRLMLELSWEVLERAGIPPTSLQASPTGVFVGLIPQEYGPRLAEGGEGVEGYLMTGTTTSVASGRIAYTLGLEGPAISVDTACSSSLVAVHLACQSLRRGESSLAMAGGVTVMPTPGMLVDFSRMNSLAPDGRCKAFSAGANGFGMAEGAGMLLLERLSDARRNGHPVLAVLRGTAVNSDGASNGLSAPNGRAQVRVIQQALAESGLGPADIDAVEAHGTGTRLGDPIEARALFEAYGRDREQPLHLGSVKSNLGHTQAAAGVAGVIKMVLAMRAGTLPRTLHASERSKEIDWSSGAISLLDEPEPWPAGARPRRAGVSSFGISGTNAHAIIEEAPQVVEGERVEAGDVVAPWVLSASSAEGLRAQAARLAAHLREHPGQDPRDIAYSLATGRAALPHRAAFAPVDESAALRVLDGLATGNADGAAVGTSRAQQRAVFVFPGQGWQWAGMAVDLLDTSPVFAAALRECADALEPHLDFEVIPFLRAEAARREQDAALSTERVDVVQPVMFAVMVSLASMWRAHGVEPAAVIGHSQGEIAAACVAGALSLDDAARVVALRSRVIATMPGNKGMASIAAPAGEVRARIGDRVEIAAVNGPRSVVVAGDSDELDRLVASCTTECIRAKRLAVDYASHSSHVETIRDALHAELGEDFHPLPGFVPFFSTVTGRWTQPDELDAGYWYRNLRRTVRFADAVRALAEQGYRTFLEVSAHPILTAAIEEIGDGSGADLSAIHSLRRGDGSLADFGEALSRAFAAGVAVDWESVHLGTGARRVPLPTYPFQRERVWLEPKPVARRSTEVDEVSALRYRIEWRPTGAGEPARLDGTWLVAKYAGTADETSTAAREALESAGARVRELVVDARCGRDELAERLRSVGEVAGVLSLLAVDEAEPEEAPLALASLADTLSLVQAMVSAELGCPLWTVTESAVATGPFERVRNAAHGALWGVGRVIALENPAVWGGLVDVPAGSVAELARHLAAVVSGGAGEDQLALRADGVYGRRWVRAAAPATDDEWKPTGTVLVTGGTGGVGGQIARWLARRGAPHLLLVSRSGPDADGAGELVAELEALGARTTVAACDVTDRESVRELLGGIGDDVPLSAVFHAAATLDDGTVDTLTGERIERASRAKVLGARNLHELTRELDLTAFVLFSSFASAFGAPGLGGYAPGNAYLDGLAQQRRSDGLPATAVAWGTWAGSGMAEGAVADRFRRHGVIEMPPETACRALQNALDRAEVCPIVIDVRWDRFLLAYTAQRPTRLFDEIDDARRAAPQAPAEPRVGALASLPAPEREEALFELVRSHAAAVLGHASAERVPADQAFAELGVDSLSALELRNRLGAATGVRLPTTTVFDHPDVRTLAAHLAAELGGATGAEQAAPATTAPVDEPIAIVGMACRLPGEVDSPERLWELITSGRDSAAEVPDDRGWVPDELMASDAAGTRAHGNFMAGAGDFDAAFFGISPREALAMDPQQRQALETTWEALESAGIPPETLRGSDTGVFVGMSHQGYATGRPRPEDGVDGYLLTGNTASVASGRIAYVLGLEGPALTVDTACSSSLVALHTACGSLRDGDCGLAVAGGVSVMAGPEVFTEFSRQGALSPDGRCKPFSDEADGFGLGEGSAFVVLQRLSDARREGRRVLGVVAGSAVNQDGASNGLSAPSGVAQQRVIRRAWARAGITGADVAVVEAHGTGTRLGDPVEASALLATYGKSRGSSGPVLLGSVKSNIGHAQAAAGVAGVIKVLLGLERGVVPPMLCRGERSGLIDWSSGEIELADGVREWSPAADGVRRAGVSAFGVSGTNAHVIIAEPPEPEPVPQPRRMLPATGVVPVVLSARTGAALRAQAGRLADHLAAHPGIAPADVSWTMARARQHFEERAAVLAADTAEAVHRLRAVADGAVVPGVVTGSASDGGSVFVFPGQGAQWEGMARELLPVPVFAESIAECDAVLSEVAGFSVSEVLEPRPDAPSLERVDVVQPVLFAVMVSLARLWRACGAVPSAVIGHSQGEIAAAVVAGALSLEDGMRVVARRSRAVRAVAGRGSMLSVRGGRSDVEKLLADDSWTGRLEVAAVNGPDAVVVAGDAQAAREFLEYCEGVGIRARAIPVDYASHTAHVEPVRDELVQALAGITPRRAEVPFFSTLTGDFLDGTELDAGYWYRNLRHPVEFHSAVQALTDQGYATFIEVSPHPVLASSVQETLDDAESDAAVLGTLERDAGDADRFLTALADAHTRGVAVDWEAVLGRAGLVDLPGYPFQGKRFWLLPDRTTPRDELDGWFYRVDWTEVPRSEPAALRGRWLVVVPEGHEEDGWTVEVRSALAEAGAEPEVTRGVGGLVGDCAGVVSLLALEGDGAVQTLVLVRELDAEGIDAPLWTVTFGAVDAGSPVARPDQAKLWGLGQVASLERGPRWTGLVDLPHMPDPELRGRLTAVLAGSEDQVAVRADAVRARRLSPAHVTATSEYAVPGGTILVTGGTAGLGAEVARWLAGRGAEHLALVSRRGPDTEGVGDLTAELTRLGARVSVHACDVSSREPVRELVHGLIEQGDVVRGVVHAAGLPQQVAINDMDEAAFDEVVAAKAGGAVHLDELCSDAELFLLFSSGAGVWGSARQGAYAAGNAFLDAFARHRRGRGLPATSVAWGLWAAGGMTGDEEAVSFLRERGVRAMPVPRALAALDRVLASGETAVVVTDVDWPAFAESYTAARPRPLLDRIVTTAPSERAGEPETESLRDRLAGLPRAERTAELVRLVRTSTATVLGHDDPKAVRATTPFKELGFDSLAAVRLRNLLNAATGLRLPSTLVFDHPNASAVAGFLDAELGTEVRGEAPSALAGLDALEGALPEVPATEREELVQRLERMLAALRPVAQAADASGTGANPSGDDLGEAGVDELLEALGRELDGD

>Mes207

MTDSEKVAEYLRRATLDLRAARQRIRELESDPIAIVSMACRLPGGVNTPQRLWELLREGGETLSGFPTDRGWDLARLHHPDPDNPGTSYVDKGGFLDDAAGFDAEFFGVSPREAAAMDPQQRLLLETSWELVENAGIDPHSLRGTATGVFLGVAKFGYGEDTAAAEDVEGYSVTGVAPAVASGRISYTMGLEGPSISVDTACSSSLVALHLAVESLRKGESSMAVVGGAAVMATPGVFVDFSRQRALAADGRSKAFGAGADGFGFSEGVTLVLLERLSEARRNGHEVLAVVRGSALNQDGASNGLSAPSGPAQRRVIRQALESCGLEPGDVDAVEAHGTGTALGDPIEANALLDTYGRDRDADRPLWLGSVKSNIGHTQAAAGVTGLLKVVLALRNGELPATLHVEEPTPHVDWSSGGVALLAGNQPWRRGERTRRARVSAFGISGTNAHVIVEEAPEREHRETTAHDGRPVPLVVSARTTAALRAQAAQIAELLERPDADLAGVGLGLATTRARHEHRAAVVASTREEAVRGLREIAAGAATADAVVEGVTEVDGRNVVFLFPGQGSQWAGMGAELLSSSPVFAGKIRACDESMAPMQDWKVSDVLRQAPGAPGLDRVDVVQPVLFAVMVSLAELWRSYGVEPAAVVGHSQGEIAAAHVAGALTLEDAAKLVVGRSRLMRSLSGEGGMAAVALGEAAVRERLRPWQDRLSVAAVNGPRSVVVSGEPGALRAFSEDCAAEGIRVRDIDVDYASHSPQIERVREELLETTGDIAPRPARVTFHSTVESRSMDGTELDARYWYRNLRETVRFADAVTRLAESGYDAFIEVSPHPVVVQAVEEAVEEADGAEDAVVVGSLHRDGGDLSAFLRSMATAHVSGVDIRWDVALPGAAPFALPTYPFQRKRYWLQPAAPAAASDELAYRVSWTPIEKPESGNLDGDWLVVTPLISPEWTEMLCEAINANGGRALRCEVDTSASRTEMAQAVAQAGTGFRGVLSLLSSDESACRPGVPAGAVGLLTLVQALGDAGVDAPVWCLTQGAVRTPADDDLARPAQTTAHGFAQVAGLELPGRWGGVVDLPESVDDAALRLLVAVLRGGGRAEDHLAVRDGRLHGRRVVRASLPQSGSRSWTPHGTVLVTGAASPVGDQLVRWLADRGAERLVLAGACPGDDLLAAVEEAGASAVVCAQDAAALREALGDEPVTALVHAGTLTNFGSISEVAPEEFAETIAAKTALLAVLDEVLGDRAVEREVYCSSVAGIWGGAGMAAYAAGSAYLDALAEHHRARGRSCTSVAWTPWALPGGAVDDGYLRERGLRSLSADRAMRTWERVLAAGPVSVAVADVDWPVLSEGFAATRPTALFAELAGRGGQAEAEPDSGPTGEPAQRLAGLSPDEQQENLLELVANAVAEVLGHESAAEINVRRAFSELGLDSLNAMALRKRLSASTGLRLPASLVFDHPTVTALAQHLRARLVGDADQAAVRVVGAADESEPIAIVGIGCRFPGGIGSPEQLWRVLAEGANLTTGFPADRGWDIGRLYHPDPDNPGTSYVDKGGFLTDAADFDPGFFGITPREALAMDPQQRLMLETAWEAVERAGIDPDALRGTDTGVFVGMNGQSYMQLLAGEAERVDGYQGLGNSASVLSGRIAYTFGWEGPALTVDTACSSSLVGIHLAMQALRRGECSLALAGGVTVMSDPYTFVDFSTQRGLASDGRCKAFSARADGFALSEGVAALVLEPLSRARANGHQVLAVLRGSAVNQDGASNGLAAPNGPSQERVIRQALAASGVPAADVDVVEAHGTGTELGDPIEAGALIATYGQDRDRPLRLGSVKTNIGHTQAAAGAAGVIKVVLAMRHGMLPRSLHADELSPHIDWESGAVEVLREEVPWPAGERPRRAGVSSFGVSGTNAHVIVEEAPAEQEAARTERGPLPFVLSGRSEAVVAAQARALAEHLRDTPELGLTDAAWTLATGRARFDVRAAVLGDDRAGVCAELDALAEGRPSADAVAPVTSAPRKPVLVFPGQGAQWVGMARDLLESSEVFAESMSRCAEALSPHTDWKLLDVVRGDGGPDPHERVDVLQPVLFSIMVSLAELWRAHGVTPAAVVGHSQGEIAAAHVAGALSLEAAAKVVALRSQVLRELDDQGGMVSVGASRDELETVLARWDGRVAVAAVNGPGTSVVAGPTAELDEFFAEAEAREMKPRRIAVRYASHSPEVARIEDRLAAELGTITAVRGSVPLHSTVTGEVIDTSAMDASYWYRNLRRPVLFEQAVRGLVEQGFDTFVEVSPHPVLLMAVEETAEHAGAEVTCVPTLRREQSGPHEFLRNLLRAHVHGVGADLRPAVAGGRPAELPTYPFEHQRFWPRPHRPADVSALGVRGAEHPLLLAAVDVPGHGGAVFTGRLSTDEQPWLAEHVVGGRTLVPGSVLVDLALAAGEDVGLPVLEELVLQRPLVLAGAGALLRMSVGAPDESGRRTIDVHAAEDVADLADAQWSQHATGTLAQGVAAGPRDTEQWPPEDAVRIPLDDHYDGLAEQGYEYGPSFQALRAAWRKDDSVYAEVSIAADEEGYAFHPVLLDAVAQTLSLGALGEPGGGKLPFAWNTVTLHASGATSVRVVATPAGADAMALRVTDPAGHLVATVDSLVVRSTGEKWEQPEPRGGEGELHALDWGRLAEPGSTGRVVAADASDLDAVLRSGEPEPDAVLVRYEPEGDDPRAAARHGVLWAAALVRRWLEQEELPGATLVIATSGAVTVSDDDSVPEPGAAAMWGVIRCAQAESPDRFVLLDTDAEPGMLPAVPDNPQLALRGDDVFVPRLSPLAPSALTLPAGTQRLVPGDGAIDSVAFEPAPDVEQPLRAGEVRVDVRATGVNFRDVLLALGMYPQKADMGTEAAGVVTAVGPDVDAFAPGDRVLGLFQGAFAPIAVTDHRLLARVPDGWSDADAAAVPIAYTTAHYALHDLAGLRAGQSVLIHAAAGGVGMAAVALARRAGAEVLATAGPAKHGTLRALGLDDEHIASSRETGFARKFRERTGGRGVDVVLNSLTGELLDESADLLAEDGVFVEMGKTDLRDAGDFRGRYAPFDLGEAGDDRLGEILREVVGLLGAGELDRLPVSAWELGSAPAALQHMSRGRHVGKLVLTQPAPVDPDGTVLITGGTGTLGRLLARHLVTEHGVRHLLLVSRRGADAPGSDELRAEIEDLGASAEIAACDTADRDALSALLDGLPRPLTGVVHAAGVLADGLVTSIDEPAVEQVLRAKVDAAWNLHELTANTGLSFFVLFSSAASVLAGPGQGVYAAANESLNALAALRRTRGLPAKALGWGLWAQASEMTSGLGDRIARTGVAALPTERALALFDSALRRGGEVVFPLSINRSALRRAEFVPEVLRGMVRAKLRAAGQAEAAGPNVVDRLAGRSESDQVAGLAELVRSHAAAVSGYGSADQLPERKAFKDLGFDSLAAVELRNRLGTATGVRLPSTLVFDHPTPLAVAEHLRDRLFAASPAVDIGDRLDELEKALEALSAEDGHDDVGQRLESLLRRWNSRRADAPSTSAISEDASDDELFSMLDQRFGGGEDL

>Mes208

MSGDNGMTEEKLRRYLKRTVTELDSVTARLREVEHRAGEPIAIVGMACRFPGDVDSPESFWEFVSGGGDAIAEAPADRGWEPDPDARLGGMLAAAGDFDAGFFGISPREALAMDPQQRIMLEISWEALERAGHDPVSLRGSATGVFTGVGTVDYGPRPDEAPDEVLGYVGTGTASSVASGRVAYCLGLEGPAMTVDTACSSGLTALHLAMESLRRDECGLALAGGVTVMSSPGAFTEFRSQGGLAADGRCKPFSKAADGFGLAEGAGVLVLQRLSAARREGRPVLAVLAGSAVNQDGASNGLTAPSGPAQQRVIRRALENAGVRAGDVDYVEAHGTGTRLGDPIEVHALLSTYGAERDPDDPLWIGSVKSNIGHTQAAAGVAGVMKAVLALRHGEMPRTLHFDEPSPQIEWDLGAVSVVSQARSWPAGERPRRAGVSSFGISGTNAHVIVEEAPEADEPEPAPDSGPVPLVLSGRDEQAMRAQAGRLADHLAPEPRNSLRDTGFTLATRASAMEHRAVVVGDRDEALAGLRAVADRRIADRTATGQGPNSPRRVAMVFPGQGAQWQGMARDLLRESQVFADSIRDCERALAPHVDWSLTDLLSGARPLDRVDVVQPALFAVMVSLAALWRSHGVEPAAVVGHSQGEIAAAHVAGALTLEDAAKLVAVRSRVLRRLGGQGGMASFGLGTEQAAERIGRFAGALSIASVNGPRSVVVVAGESGPLDELIAECEAEAHKARRIPVDYASHSPQVESLREELLTELAGISPVSADVALYSTTTGQPIDTATMDTAYWYANLREQVRFQDATRQLAEAGFDAFVEVSPHPVLTVGIEATLDSALPADAGACVVGTLRRDRGGLADFHTALGEAYAQGVEVDWSPAFADARPVELPVYPFQRYWLPIPTGGRARDEDDDWRYQVVWREAEWESASLAGRVLLVTGPGVPSELSDAIRSGLEQSGATVLTCDVESRSTIGTALEAADTDALSTVGVAAVPHGEAVDPSLDALALVQALGAAGVEAPLWVLTRNAVQVADGELVDPAQAMVGGLGRVVGIEQPGRWGGLVDLVDADAASIRSLAAVLADPRGEEQVAIRADGIKVARLVPAPARARTHPLEPLAGTVLVTGGTGGIGAHLARWLARSGAEHLVLLGRRGADAPGASELREELTALGTGVTIAACDVADRARLEAVLAAEAAAEGRTVSAVMHAAGVSTSTPLDDLTEAEFTEIADVKVRGTVNLDELCPDLDAFVLFSSNAGVWGSPGLASYAAANAFLDGFARAARSEGAPVTSIAWGLWAGQNMAGDEGGEYLRSQGLRAMDPDRAVEELHITLDHGQTSVSVVDMDRRRFVELFTAARHRPLFDEIAGARAEARQSEEGPALAQRLAALLCDGREREHLAHLIRAEVAAVLGHGDDAAIDRDRAFRDLGFDSMTAVDLRNRLAAVTGVREAATVVFDHPTITRLADHYLERLVGAAEAEQAPALVREVPPKDADDPIAIVGMACRFPGGVHNPGELWEFIVGGGDAVTEMPTDRGWDLDALFDPDPQRHGTSYSRHGAFLDGAADFDAAFFGISPREALAMDPQQRQVLETTWELFENAGIDPHSVRGSDTGVFLGAAYQGYGQDAVVPEDSEGYLLTGNSSAVVSGRVAYVLGLEGPAVTVDTACSSSLVALHSACGSLRDGDCGLAVAGGVSVMAGPEVFTEFSRQGGLAVDGRCKAFSAEADGFGLPEGVAVVQLQRLSDGPAEGGRQVLGVVAGSAINQDGATNGLAAPSGVAQQRVIRKAWARAGITGADVAVVEAHGTGTRLGDPVEASALLATYGKSRGSSGPVLLGSVKSNIGHAQAAAGVAGVIKVVLGLNRGLVPPMLCRGERSPLIEWSSGGVELAEAVSPWPPAADGVRRAGVSAFGVSGTNAHVIIAEPPEPEPLPEPGPVGVLAAANSVPVLLSARTETALAAQARLLESAVDDSVPLTALASALATGRAHLPRRAALLAGDHEQLRGQLRAVAEGVAAPGATTGTASAGGVVFVFPGQGAQWEGMARGLLSVPVFAESIAECDAVLSEVAGFSASEVLEQRPDAPSLERVDVVQPVLFSVMVSLARLWGACGVSPSAVIGHSQGEIAAAVVAGVLSLEDGVRVVALRAKALRALAGKGGMVSLAAPGERARALIAPWEDRISVAAVNSPSSVVVSGDPEALAELVARCEDEGVRAKTLPVDYASHSRHVEEIRETILADLDGISARRAAIPLYSTLHGERRDMGPRYWYDNLRSQVRFDEAVSAQSPDGHATFVEMSPHPVLTAAVQEIAADAVAIGSLHRDTAEEHLIAELARAHVHGVAVDWRNVFPAAPPVALPNYPFEPQRYWLAPEVSDQLADSRYRVDWRPLATTPVDLEGGFLVHGSAPESLTSAVEKAGGVVPVASADREALAAALREVPGEVAGVLSVHTGAANALALHQSLGEAGVRAPLWLVTSRAVALGESEPVDPEQAMVWGLGRVMGLETPERWGGLVDLPAEPAPGDGEAFVACLGADGHEDQVAIRDHARYGRRLVRAPLGTRESSWEPAGTALVTGGTGALGGHVARHLARCGVEDLVLVSRRGVDAPAAAELEAELVALGPKTTITACDVADREQLSKLLEELRGQGRPVRTVVHTAGVPESRPLHEIGELESVCAAKVTGARLLDELCPDAETFVLFSSGAGVWGSANLGAYSAANAYLDALAHRRRAEGRAATSVAWGAWAGEGMATGDLEGLTRRGLRPMAPDRAIRALHQALDNGDTCVSIADVDWEAFAVGFTAARPRPLLDELVTPAVGAVPAVQAAPAREMTSQELLEFTHSHVAAILGHSSPDAVGQDQPFTELGFDSLTAVGLRNQLQQATGLALPATLVFEHPTVRRLADHIGQQLDSGTPAREASSALRDGYRQAGVSGRVRSYLDLLAGLSDFREHFDGSDGFSLDLVDMADGPGEVTVICCAGTAAISGPHEFTRLAGALRGIAPVRAVPQPGYEEGEPLPSSMAAVAAVQADAVIRTQGDKPFVVAGHSAGALMAYALATELLDRGHPPRGVVLIDVYPPGHQDAMNAWLEELTATLFDRETVRMDDTRLTALGAYDRLTGQWRPRETGLPTLLVSAGEPMGPWPDDSWKPTWPFEHDTVAVPGDHFTMVQEHADAIARHIDAWLGGGNS

>Mes209

MTYVIAEPCVDVLDKACIEECPVDCIYEGGRMLYIHPDECVDCGACEPVCPVEAIYYEDDVPDEWAAYTKANVDFFDELGSPGGAAKVGKVDRDVEPVSSLPPQGE

>Mes210

MFRTMLKSKIHRATVTQADLHYVGSVTVDADLMDAADLLEGEQVAIVDVTNGARLETYVITGERGSGVIGINGAAAHLIEPGDLVILISYGVMDELEARSVRPKVIFVDADNRIVERGQDPGHAPAGSGLAGTAASVTSAITEAAAETDDAAKLDALLQQPEH

>Mes211

EAPCGDTSGFEQVRLADLPPEATDTYELIEKGGPYPYPEDGTVFENREGILPDCAEGYYHEYTVKTPSGDDRGARRFVVGDGGEYFYTEDHYESFRLTIVN

>Mes212

MSREEVLVSTDWAEQNLNTDGVVFAEVDEDTTAYDGGHIPGAIKLDWKNELQDHVRRDFVNREGFEKLLSAKGIGNDDTVILYGGNNNWFAAYAYWYFKLYGHSDVKLLDGGRKKWELDGRELTKEEPNRAATAYKAQEPDASIRAFRDEVVDAIGNKNLVDVRSPDEFAGKLLAPAHLPQESAQRAGHIPSAINVPWSKAANEDGTFKSDEELKQVYGEAGLDTDKDTIAYCRIGERSSHTWFVLRELLGHTNVKNYDGSWTEYGSLVGVPIENPQEQGA

>Mes213

IVGGEDANVQDHPFTVALVTPDGQQFCGGTLAAPNKVVTAAHCTVGSQPADINVVSGRTVMSSNIGTVSKVTNVWVHPEYQDAAKGFDVSVLTLEAPVKEAPIELAKADDAGYAPDTAATILGWGNTSEGGQQADHLQKATVPVNSDDTCKQAYGEYTPNAMVCAGVPEGGVDTCQGDSGGPMVVNNKLIGVTSWGEGCARPGKPGVYARVGAYYDVLMEQINAGAV

>Mes215

MRIVDINERLAISGQPNTDEFINFARRGYRSIINLRPDGEEPNQPGNDAEQAAARRAGLAYNFVPVIGTSITEADIQAFQRAIATTEGSVLVHCKSGTRALMLYALSEVIDGRMKRDEVEALGHAHGFDLGRAVTWLERQAIQTPRVSGFFDPRTSSIQYVVTDQTTKRCAIIDPVLDFDEKSGATATTNADAILAHVEQQGLTVEWILDTHPHADHFSAAQYLKQRTGAPTAIGTHVTEVQRLWREIYNWPTLSANGSQWDHLFADGDVFNVGSIKGRVMFSPGHTLASVTYVIGDTAFVHDTIFMPDAGTARADFPGGSARALWSSIQTILSLPDETRLFTGHDYQPSGRHPRWESTVGEQKKANPHLAGVDETTFVALREARDKTLPMPKLILHALQVNVLGGRLPEPETNGRRYLKFPLNALEGAAW

>Mes222

MSEYWYPILGGILLGLSTVMLLLLNGRIAGISGIVGRLLQGGNPAQNIPFVVGLVLGPLLFTVIFDRFPSVTVAATWPTIIVAGLLVGLGTRMGAGCTSGHGIVGIARHSPRSIVATAIFLISGMATATFMGVYQ

>Mes223

MNLHFYSTLRFTVALAAGLLFGFGLALSEMINPIRVLSFLNVASGHWNPSLLFVLGSALAVAFPGMALQRRLKRPLLDECFHLPSKKVIDRRIVFGSAIFGTGWGLTGLCPGPAIASLSTGLGSVLLFVAAMAAGMIIHDRIVVRSLS

>Mes224

MTIQSLIVTIGSGGLVGFALGLLGGGGSILATPLLLYVVGVTNPHIAIGTSAVAVSVNAYANLIAHAWKGHVWWRSAVIFALVGTLGAFLGSSIGMLIDGQRLLLLFGLLMAMVGLLMLRGRATAPRAEHHQTVLRMCMKTSSVAILTGAASGFFGIGGGFLIVPALIFATRMPTINAIGSSLLAVGSFGLITTLNYARHDLVNWTIAMEFIVGGITGGGLGTLLATRLSASKHLLNRVFGLIVIAVAIYVIWRSWASLVA

>Mes228

MKAAVITKDHTIEVKDTKLRPLKYGEALLEMEYCGVCHTDLHVKNGDFGDETGRITGHEGIGIVKQVGEGVTSLKVGDRASVAWFFKGCGHCEYCVSGNETLCRNVENAGYTVDGAMAEECIVVADYSVKVPDGLDPAVASSITCAGVTTYKAVKVSQIQPGQWLAIYGLGGLGNLALQYAKNVFNAKVIAIDVNDEQLAFAKELGADMVINPKNEDAAKIIQEKVGGAHATVVTAVAKSAFNSAVEAIRAGGRVVAVGLPPEKMDLSIPRLVLDGIEVLGSLVGTREDLKEAFQFAAEGKVKPKVTKRKVEEINQIFDEMEHGKFTGRMVVDFTHH

>Mes229

MASSTFYIPFVNEMGEGSLEKAIKDLNGSGFKNALIVSDAFMNKSGVVKQVADLLKAQGINSAVYDGVMPNPTVTAVLEGLKILKDNNSDFVISLGGGSPHDCAKAIALVATNGGEVKDYEGIDKSKKPALPLMSINTTAGTASEMTRFCIITDEVRHVKMAIVDRHVTPMVSVNDPLLMVGMPKGLTAATGMDALTHAFEAYSSTAATPITDACALKAASMIAKNLKTACDNGKDMPAREAMAYAQFLAGMAFNNASLGYVHAMAHQLGGYYNLPHGVCNAVLLPHVLAYNASVVAGRLKDVGVAMGLDIANLGDKEGAEATIQAVRDLAASIGIPANLTELGAKKEDVPLLADHALKDACALTNPRQGDQKEVEELFLSAF

>Mes232

MTAIVSIHGRQVVDSRGNPTVEVDVTLEDGSFGRAAVPSGASTGVHEAVELRDGDKTRWGGKGVTKAVHAVNNEIANAIIGLEAEDQELIDQTMIKLDGTPNKGKFGANAILGVSLAVAKAAAEARGLPLYRYVGGTAAHVLPVPMMNIVNGGMHADNPIDFQEFMIAPVGASSINEAVRIGTEVFHTLKKELSAKGMNTNVGDEGGFAPSLDSASSALDFIVDSISKAGYKPGEDVFIALDAASSEFYNKDQNIYDLKGEGRKLTSAQLVDYYVELCGKYPIYSIEDGLAEDDFEGWKILTEKLGDKVQLVGDDLFVTNVKRLSDGIERGIANSLLVKFNQIGSLSETLAAVNMANDASYTAVMSHRSGETEDTTIADLAVATNCGQIKTGSLCRSERIAKYNQLMRIEEELGSVAKYAGRSVLRKAK

>Mes233

MTNKISSSDNLSNAVSATDDNASRTPNLTRRALVGGGVGLAAAGALASGLQAATLPAGASQVPTTPAGRPMPYAIRPMPEDRRFGYAIVGLGKYALNQILPGFAGCQHSRIEALVSGNAEKAKIVAAEYGVDPRKIYDYSNFDKIAKDPKIDAVYIILPNSLHAEFAIRAFKAGKHVMCEKPMATSVADCQRMIDAAKAANKKLMIGYRCHYDPMNRAAVKLIRENQLGKLGMVTTDNSDVMDQNDPAQQWRLRRELAGGGSLMDIGIYGLNGTRYLLGEEPIEVRAYTYSDPNDERFVEVEDRIIWQMRFRSGALSHGASSYSTTTTSRFSVQGDKAVLLMDPATGYYQNLISVQTPGHANQSMMPQFIMPANNQFSAQLDHLAEAVINNKPVRSPGEEGMQDVRLIQAIYEAARTGRPVNTDWGYVRQGGY

>Mes234

MTTGRMSRRECLSAAVMVPIAAMTATATITGSAQAAKNNMNGSTIGKITKFSPRLDAILDVSTPIEVIASDIQWSEGPVWVKNGNFLLFSDPPANIMRKWTPDAGVSIFLKPSGHAEPIPAGQFREPGSNGMKVGPDGKIWVADSGTRAIMKVDPVTRQRSVVVDNYKGKRFNSPNDLFFSKSGAVYFTDPPYGLTNLDESDIKEMNYNGVFRLSPDGRLDLIEAGLSRPNGLALSPDETKLYVSNSDRASPNIWVYSLDSNGLPTSRTLLRNFRKEYFDQGLAGLPDGMNIDKQGNLFASAPGGIYIFAPDGECLGLISGNPGQPLSNCCFGEKGQTLFISASHNVVRVRTKTFG

>Mes235

MPTLVLSRHGQSEWNLENRFTGWWDVNLTEQGVQEATAGGKALAEKGFEFDIAFTSVLTRAIKTTNLILEAGKTLWVPTEKDWRLNERHYGGLTGLNKAETAAKHGEEQVHIWRRSYDVPPPPMEKGSKFDLSGDRRYDGVKIPETESLKDTVARVLPYWEERIAPELKAGKRVLIGAHGNSLRALVKHLSKLSDEEIVKFELPTGQPLVYELNDDLTPKDRYFLNER

>Mes237

MSYTVGTYLAERLVQIGLKHHFAVAGDYNLVLLDNLLLNKNMEQVYCCNELNCGFSAEGYARAKGAAAAVVTYSVGALSAFDAIGGAYAENLPVILISGAPNNNDHAAGHVLHHALGKTDYHYQLEMAKNITAAAEAIYTPEEAPAKIDHVIKTALREKKPVYLEIACNIASMPCAAPGPASALFNDEASDEASLNAAVEETLKFIANRDKVAVLVGSKLRAAGAEEAAVKFADALGGAVATMAAAKSFFPEENPHYIGTSWGEVSYPGVEKTMKEADAVIALAPVFNDYSTTGWTDIPDPKKLVLAEPRSVVVNGIRFPSVHLKDYLTRLAQKVSKKTGALDFFKSLNAGELKKAAPADPSAPLVNAEIARQVEALLTPNTTVIAETGDSWFNAQRMKLPNGARVEYEMQWGHIGWSVPAAFGYAVGAPERRNILMVGDGSFQLTAQEVAQMVRLKLPVIIFLINNYGYTIEVMIHDGPYNNIKNWDYAGLMEVFNGNGGYDSGAGKGLKAKTGGELAEAIKVALANTDGPTLIECFIGREDCTEELVKWGKRVAAANSRKPVNKLL

>Mes238

MLNKAGIAEPSLWTRADAMKVHTDDPTATMPTIDYDFPVMTDKYWVWDTWPLRDINGQVVSFQGWSVIFALVADRTKYGWHNRNDGARIGYFYSRGGSNWIFGGHLLKDGANPRSWEWSGCTIMAPGTANSVEVFFTSVNDTPSESVPAQCKGYIYADDKSVWFDGFDKVTDLFQADGLYYADYAENNFWDFRDPHVFINPEDGKTYALFEGNVAMERGTVAVGEEEIGPVPPKTETPDGARYCAAAIGIAQALNEARTEWKLLPPLVTAFGVNDQTERPHVVFQNGLTYLFTISHHSTYADGLSGPDGVYGFVSENGIFGPYEPLNGSGLVLGNPSSQPYQAYSHYVMTNGLVTSFIDTIPSSDPNVYRYGGTLAPTIKLELVGHRSFVTEVKGYGYIPPQIEWLAEDESSNSAAALSLLNK

>Mes239

MESPSYKNLIKAEDAQKKAGKRLLSSEWYPGFHVTPLTGWMNDPNGLIFFKGEYHLFYQYYPFAPVWGPMHWGHAKSRDLVHWETLPVALAPGDSFDRDGCFSGCAVDNNGVLTLIYTGHIVLSNDSLDAIREVQCMATSIDGIHFQKEGIVLEKAPMPQVAHFRDPRVWKENNHWFMVVGYRTDDEKHQGIGHVALYRSENLKDWIFVKTLLGDNSQLPLGKRAFMWECPDFFSLGNRSVLMFSPQGLKASGYKNRNLFQNGYILGKWQAPQFTPETSFQELDYGHDFYAAQRFEAKDGRQILIAWFDMWENQKPSQRDGWAGCMTLPRKLDLIDNKIVMTPVREMEILRQSEKIESVVTLSDAEHPFTMDSPLQEIELIFDLEKSSAYQAGLALRCNGKGQETLLYIDRSQNRIILDRNRSGQNVKGIRSCPLPNTSKVRLHIFLDRSSIEIFVGDDQTQGLYSISSRIFPDKDSLKGRLFAIEGYAVFDSFKRWTLQDANLAAFSSDAC

>Mes240

MVEATAQETDRPRFSFSIAAREGKARTGTIEMKRGVIRTPAFMPVGTAATVKALKPETVRATGADIILGNTYHLMLRPGAERIAKLGGLHSFMGWDRPILTDSGGYQVMSLSSLTKQSEEGVTFKSHLDGSRHMLSPERSIEIQHLLGSDIVMAFDECTPYPATPSRAASSMERSMRWAKRSRDAFDSRKEQAENAALFGIQQGSVFENLRQQSADALAEIGFDGYAVGGLAVGEGQDEMFRVLDFSVPMLPDDKPHYLMGVGKPDDIVGAVERGIDMFDCVLPTRSGRNGQAFTWDGPINIRNARFSEDLTPLDSECHCAVCQKWSRAYIHHLIRAGEILGAMLMTEHNIAFYQQLMQKIRDSISEGRFSQFAQDFRARYFARNS

>Mes241

MTVFKHIAIIGLGLIGSSAARATKAYCPDVTVSLYDKSEFVRDRARALNLGDNVTDDIQDAVREADLVLLCVPVRAMGIVAAAMAPALKKDVIICDTGSVKVSVIKTLQDNLPNHIIVPSHPLAGTENNGPDAGFAELFQDHPVILTPDAHTPAQAIAYIADYWEEIGGRINLMSAEHHDHVLALTSHLPHVIAYQLIGMVSGYEKKSRTPIMRYSAGSFRDATRVAASEPRLWQDIMLENAPALLPVLDHFIADLKKLRTAIASQDGDYLLEHFKESQKARLALKTDHDIRP

>Mes242

MATIRPDDKAIDAAARHYGITLDKTARLEWPALIDGALGSYDVVDQLYADEATPPTTSREHAVPSASENPLSAWYVTTSIPPTSDGVLTGRRVAIKDNVTVAGVPMMNGSRTVEGFTPSRDATVVTRLLAAGATVAGKAVCEDLCFSGSSFTPASGPVRNPWDRQREAGGSSGGSAALVANGDVDFAIGGDQGGSIRIPAAFCGVVGHKPTFGLVPYTGAFPIERTIDHLGPITRTVHDAALMLSVIAGRDGNDPRQADSVEAGDYLSTLDSDVDGLRIGIVREGFGHAVSQPEVDDAVRAAAHSLTEIGCTVEEVNIPWHLHAFHIWNVIATDGGAYQMLDGNGYGMNAEGLYDPELMAHFASRRIQHADALSETVKLVALTGHHGITTLGGASYGKARNLVPLARAAYDTALRQFDVLVMPTLPYVASELPAKDVDRATFITKALGMIANTAPFDVTGHPSLSVPAGLVNGLPVGMMITGRHFDDATVLRVGRAFEKLRGAFPTPAERASNSAPQLSPA

>Mes244

MPEAIEVRKVPLHSVSDASELAKLIDDGVLEADRVIAVIGKTEGNGGVNDYTRIIADRAFREVLSAKGNRSPEEVAEVPIVWSGGTDGVISPHATIFATVPADKVTKTDEPRLTVGVAMSEQLLPEDIGRTAMITKVAAAVKDAMADAGITDPADVHYVQTKTPLLTIHTIRDAKSRGKTVWTEQTHESMDLSNGGTALGIAVALGEIDMPTDEDVMHSRELFSSVASCSSGVELDRAQIVVVGNARGVGGRYRIGHSVMKDPLDQDGIWAAIRDAGLELPERPHSNDLDGQLVNVFLKCEASQDGTVRGRRNAMLDDSDVHWHRQIKSCVGGVTAAVTGDPAVFVSVSAAHQGPEGGGPVAAIVDLGQ

>Mes246

MTSKIEQPRWASKDSAAGAASTPDEKIVLEFMDALTSNDAAKLIEYFAEDTMYQNMPLPPAYGRDAVEQTLAGLFTVMSIDAVETFHIGSSNGLVYTERVDVLRALPTGKSYNLSILGVFQLTEGKITGWRDYFDLREFEEAVDLPLRG

>Mes247

MTDDFGRVDFGAFLAPWHRADSDANFAIHQDLELVEHLDRLGFAEFWLGEHHSGGVEIVASPEMFMAAAAQRTQRIKLGLGVVSLPYHHPFLVADRLVLLDHLSRGRMIFGAGPGQLADDAKMLGIDPIDSRRKMEEAFDVIHRLLAGETVTQKTDWFTCQDAYLHVAPYSNIQKAVTATVSPTGPKLAGKYGSGILSLAATNPVGVEKLAEHWKIAEDIAAENGQTVDRADWRLSGIMHVAETEEQARADVRHGLLYLMNYLSNITPGFAAAPDVDSLIDGINDAGLAVIGTPEMAVTQIRRLQEKSGGFGKFLVLHGEWASTTAALHSFELIAQQVAPHFNGDLGPRLRGYNQTMNSNRSAADITQAAQEEAQKRFEAERAIRTN

>Mes248

MARVEGQVALITGAARGQGRSHAIKLAEEGADVILVDVPNDVVDIGYPLGTADELDQTAKDVENLGRKAIVIHADVRDLESLTAEVDRAVSTLGRLDIVSANAGIASVPFLSHDIPDNTWRQMIDINLTGVWHTAKVAVPHILAGERGGSIVLTSSAAGLKGYAQISHYSAAKHGVVGLMRSLALELAPHRVRVNSLHPTQVNTPMIQNEGTYRIFSPDLENPTREDFEIASTTTNALPIPWVESVDVSNALLFLVSEDARYITGAAIPVDAGTTLK

>Mes249

AIELNQIWDFPIKEFHPFPRALMGVGAHDIIGVEAKNLGFKRTLLMTTGLRGSGIIEELVGKIEYQGVEVVLYDKVESNPKDYNVMEAAALYQKEKCDSIISIGGGSSHDAAKGARVVIAHDGRNINEFEGFAKSTNKENPPHIAVSTTAGTGSETSWAYVITDTSDMNNPHKWVGFDEATIVTLAIDDPLLYYTCPQHFTAYCGFDVLAHGSEPFVSRLDFAPSLGNAIYSVELVAKNLREAVFEPRNLKAREGMMNAQYIAGQAFNSGGLGIVHSISHAVSAFFDSHHGLNNAIALPRVWEYNLPSRYERYAQLAGALGVDTRNLTTVQAADAAVEAAIRLAKDVGIPDNFGQVRTDSYAKNQMNTKKYEGRGDVIKGDEKTVRAISEHIQDDWCTPGNPREVTVESMIPVVDHAINKSYF

>Mes250

MSSTENPDSVAAAEELHALRVEAQVLRRQLAQSPEQVRELESKVDSLSIRNSKLMDTLKEARQQLIALREEVDRLGQPPSGYGVLLSVHEDKTVDVFTSGRKMRLTCSPNIDTDTLALGQTVRLNEALTIVEAGTYEQVGEISTLREVLDDGLRALVVGHADEERIVWLAAPLAAVFADPEADIIAYDADSPTRKLRPGDSLLVDTKAGYAFERIPKAEVEDLVLEEVPDVHYDDIGGLGRQIEQIRDAVELPFLHKDLFHEYSLRPPKGVLLYGPPGCGKTLIAKAVANSLAKKIAEARGQDSKDAKSYFLNIKGPELLNKFVGETERHIRMIFQRAREKASEGTPVIVFFDEMDSIFRTRGSGVSSDVETTVVPQLLSEIDGVEGLENVIVIGASNREDMIDPAILRPGRLDVKIKIERPDAESAQDIFSKYLVDGLPINADDLAEFGGDRTACLKAMIVRVVDRMYAESEENRFLEVTYANGDKEVLFFKDFNSGAMIQNIVDRAKKYAIKSVLDTGAPGLRVQHLFDSIVDEFAENEDLPNTTNPDDWARISGKKGERIVYIRTLVTGKNASASRAIDTESNTGQYL

>Mes251

MSVTIDHTTENAAPAQAPVSDRAWALFRALDGKGLVPDGYVEGWKKTFEEDFSPRRGAELVARAWTDPEFRQLLLTDGTAAVAQYGYLGPQGEYIVAVEDTPTLKNVIVCSLCSCTAWPILGLPPTWYKSFEYRARVVREPRKVLSEMGTEIASDIEIRVYDTTAETRYMVLPQRPAGTEGWSQEQLQEIVTKDCLIGVAIPQVPTV

>Mes252

MDGVHDLAGVQGFGKVPHTVNADIGPTFHAEWEHLPYSLMFAGVAELGAFSVDEVRYVVERMEPRHYMMTPYYERYVIGVATLMVEKGILTQDELESLAGGPFPLSRPSESEGRPAPVETTTFEVGQRVRVRDEYVPGHIRMPAYCRGRVGTISHRTTEKWPFPDAIGHGRNDAGEEPTYHVKFAAEELFGSDTDGGSVVVDLFEGYLEPAA

>Mes253

MTKYARPGTADAIMSFQSRYDNWIGNEWVAPVKGQYFENPTPVTGQNFCDVARSTAEDIELALDAAHAAAPAWGKTSVAERAIILNKIADRMEENLESIALAESWDNGKPIRETLNADIPLAIDHFRYFAGAIRAQEGSLSEINSDTVAYHFHEPLGVVGQIIPWNFPILMAVWKLAPALAAGNAIVLKPAEQTPVSILHLIGIIGDLLPAGVLNIVNGFGVEAGKPLASSPRIKKIAFTGETTTGRLIMQYASQNLIPVTLELGGKSPNVFFSDVLASNDDYQDKALEGFTMFALNQGEVCTAPSRALIQEDIFDEFLAMAAIRTKAVRQGDPLDTDTMIGAQASNDQLEKILSYIEIGKAEGAKVITGGERAELGGDLSGGYYVQPTVFTGNNKMRIFQEIFGPVVSVTSFKDYDEAIEIANDTLYGLGAGVWSRDGGVAYRAGRDIQAGRVWTNTYHQYPAHAAFGGYKQSGIGRENHLMMLSHYQQTKNLLVSYAQKAQGFF

>Mes254

MTVDHAPEGVKSPTGCPVSGMAADFDPFRGAYQVDPSSSLRQARKDEPVFFSPLLDYWVVTRYEDIKQIFKTPSVFSPSITVDQITPISDEALQILGSYQFAAGRMLVNEDEPIHTERRRLLMQPFEADNVATLEPKIREVVNTYLDRVIKDGRADLIGDLLYEVPCIVALIFLGVPDEDIETCRQYGMQQTLFTWGHPTGDEQTRVATGMGKFWEFAGGLVDKLKADPNAKGWIPHAIEMQRQHPDLFDDNYLQNIMFGGVFAAHETTTNATGNAFRTLLENRSSWDEICADPTLIPKAIEECLRYSGSVVAWRRKAVVDTTVGEVDIPAGGRLLIVMASANRDDSMFPEPDDFDIHRGNAQRHLTFGIGSHTCLGATLARLEMKVFLEEVSRRLPHMSLVAGQEFSYLPNTSFRGPEHVLVEWDPQQNPVPADRP

>Mes255

MPTVTYVHPDGTKHEVEVPTGKRVMQAAIGAGIDGIVAECGGQAMCATCHVYVESPWADKFPSISEEEDEMLDDTVSPRTEASRLSCQLVVSDDVDGLIVRLPEEQV

>Mes256

MSIVIIGSGQAGFEAAVSLRSHGFSGTITLVGDEPGVPYQRPPLSKAYLHSDPDRESLALRPAQYFDDHRITLTCGKPVVRIDRDAQRVELIDATAIEYDHLILATGARNRLLPVPGANLPGVHYLRTAGEAESLTSSMASCSSLVVIGAGFIGLEVAAAARKKGLDVTVVEAMDRPMARALSSVMSGYFSTAHTEHGVHMRLSTGVKTINAADGRAAGVTTNSGDVIHADAVVVGIGVVPNIELAALTGLPVDNGIVVDEYLRTPDENISAIGDCAAYPIPGKAGLVRLESVQNAVDQARCLAAQLTGTSTHYRSVPWFWSEQYESKLQMAGLTAGADTHVVRGSVDSGVFSIFCFLGTRLLGVESVNKPRDHMAARKILATEMPLTPEQAADTDFDLKLAIARHKDTHKDEVASADIGERQVVAS

>Mes258

MMIFRALIAAATLAIAIATTLPAAADEVAVKMLNSGPGGMMVFDPALVRLKPGDSIKFLPTDKGHNVETIKGMAPDGADYVKTTVGQEAVVKFDKEGVYGFKCAPHYMMGMVALVVVGDKRDNLEAAKSVQHNKLTQKRLDPLFAQIQ

>Mes259

MMNRVKIGTALLGLTLAGIALPALAQPQSGPQTGVVFRNTVTGEALDVSQGKEGGRDTPAVKKFLETGENLYIDDKSCLRNGESLFATSCSGCHGHLAEGKLGPGLNDNYWTYPSNTTDVGLFATIFGGANGMMGPHNENLTPDEMLQTIAWIRHLYTGPKQDAVWLNDEQKKAYTPYKQGEVIPKDAKGQCKPLDE

>Mes260

MSPNPTNIHTGKTLRLLYHPASQPCRSAHQFMYEIDVPFEEEVVDISTDITERQEFRDKYNPTGQVPILVDGEFTVWESVAIARYVNEKFDGAGNWFGRGTQERAQINQFLQWYAYTLRLGGGAFHWNIFGCLIYGEKPYSPKFTAEQNKGRTLLYEAMGTLENYWLRDREYVCGDEVSYADLAAFHEFVSHEAGKIIPDRVWQGFPKIAAWFKKLSERPHAKTVSEWQYTNVGKIIRGELTASMFKRKTAVLKGTEVFSGHNHGIPYLNEKAEDYFKRVEKEGAAVA

>Mes261

MTKKVVFLDRESLDATVREFNFPHEYKEYESTWTPEEIVERLQGAEIAMINKVPMRADTLKQLPDLKLIAVAATGTDVVDKAAAKAQGITVVNIRNYAFNTVPEHVVGLMFALRRAIVPYANSVRRGDWNKSKQFCYFDYPIYDIAGSTLGIIGYGALGKSIAKRAEALGMKVLAFDVFPQDGLVDLETILTQSDVITLHVPLTPDTKNMIGAEQLKKMKRSAILINTARGGLVDEAALLQALKDGTIGGAGFDVVAQEPPKDGNILCDADLPNLIVTPHVAWASKEAMQILADQLVDNVEAFVAGKPQNVVEA

>Mes266

MAKITKVQVGEALVGDGNEVAHIDLIIGPRGSPAETAFCNGLVNNKHGFTSLLAVIAPNLPCKPNTLMFNKVTINDARQAVQMFGPAQHGVAMAVQDAVAEGIIPADEADDLYVLVGVFIHWEAADDAKIQKYNYEATKLSIQRAVNGEPKASVVTEQRKSATHPFAANA

>Mes267

MAGNETIETFLDGLASSAPTPGGGGAAAISGAMGAALVSMVCNLTIGKKKYVEVEADLKQVLEKSEGLRRTLTGMIADDVEAFDAVMGAYGLPKNTDEEKAARAAKIQEALKTATDVPLACCRVCREVIDLAEIVAEKGNLNVISDAGVAVLSAYAGLRSAALNVYVNAKGLDDRAFAEERLKELEGLLAEAGALNERIYETVKSKVN

>Mes268

MPSDIEIARAATLKPIAQVAEKLGIPDEALHNYGKHIAKIDHDFIASLEGKPEGKLVLVTAISPTPAGEGKTTTTVGLGDALNRIGKRAVMCLREPSLGPCFGMKGGAAGGGKAQVVPMEQINLHFTGDFHAITSAHSLAAALIDNHIYWANELNIDVRRIHWRRVVDMNDRALRAINQSLGGVANGFPREDGFDITVASEVMAVFCLAKNLADLEERLGRIVIAETRDRKPVTLADVKATGAMTVLLKDALQPNLVQTLEGNPALIHGGPFANIAHGCNSVIATRTGLRLADYTVTEAGFGADLGAEKFIDIKCRQTGLKPSAVVIVATIRALKMHGGVNKKDLQAENLDALEKGFANLERHVNNVRSFGLPVVVGVNHFFQDTDAEHARLKELCRDRLQVEAITCKHWAEGGAGAEALAQAVVKLAEGEQKPLTFAYETETKITDKIKAIATKLYGAADIQIESKAATKLAGFEKDGYGGLPVCMAKTQYSFSTDPTLMGAPSGHLVSVRDVRLSAGAGFVVVICGEIMTMPGLPKVPAADTIRLDANGQIDGLF

>Mes269

MSDFTLNGIKVEDTFAEAFDVAGTAIIVTNDTPKWAMIAATVMTGFATSVIGCGAEAGIDAELSPDETPDGRPGVRILLFGFEPNGLKDQLLKRVGQCILTCPGTACFAGVEGPTKIKLGGAIRYFGDGFAVAKRLPDHEGKMRRYWRIPVMDGEFLCEDSVRAVDGAVGGGNLLFLGRKHADTLIVAEIAVEAAKAIPGAILPFPGGIVRSGSKVGGRTKGMMASTNDAYCPTLKGRAGSALPPECGVVLEIVIDALTSAAVAESMRAALHAATEIGAQHGLVAVTAGNYGGNLGRHHYHLRDLLEKPAA

>Mes270

MSSNTSAPSLNALAGPLVESLVADAAKLRLIVAQENGARTVDAGANARGSIEAGRRIAEICLGGLGTVTIAPIGPVASWPYTVVVHSADPVLACLGSQYAGWSLADEEGDSGFFALGSGPGRAVAVVEELYKELGYRDNATTTALVLESGSAPPASVVNKVAAATGLAPENVTFIYAPTQSLAGSTQVVARVLEVALHKAHTVGFDLHKILDGIGSAPLSPPHPDFIQAMGRTNDAIIYGGRVQLFVDADDADAKQLAEQIPSTTSADHGAPFAEIFSRVNGDFYKIDGALFSPAEAIVTSVKTGKSFRGGRLEPQLVDASFV

>Mes271

MSKKLLFQFDTDATPSVFDVVVGYDGGADHITGYGNVTPDNVGAYVDGTIYTRGGKEKQSTAIFVGGGDMAAGERVFEAVKKRFFGPFRVSCMLDSNGSNTTAAAGVALVVKAAGGSVKGKKAVVLAGTGPVGMRSAALLAGEGAEVVLCGRKLDKAQAAADSVNKRFKVNVTAAETADDASRAEAVKGAHFVFTAGAIGLELLPQAAWQNESSIEIVADYNAQPPLGIGGIDATDKGKEYGGKRAFGALGIGGLKLKLHRACIAKLFESSEGVFDAEEIYKLAKEMA

>Mes272

MARSILHMLTPLKHMSPFDVNMAIDAGFETLIPYTGVDLTDVVSLTQDSIFSRAPQDGVRTGIFIGGKNAELALDMVDRAKKAFVPPFVNHVFADPAGSFTTGAAMVAEVNRALKARFSTDLKGKRIVIFGGAGVVAYVAAVIGALEGAQTVLVGHDGEERVSKIAFTMKWRFGIDVGAVDGTLPEARRAAITDADVILSAGPAGVSILTAEDLESAPKLLVASDVNAVPPAGIAGIDVNAVDVPLPTGKGVGIGALAVGNVKYQTQCRMFRKMLEAQEPLCLDFRDAYKLAVEIAG

>Mes273

MTATFDKVADIIAETSEIDRATITPESHTIDDLGIDSLDFLDIVFAIDKEFGIKIPLEKWTQEVNEGKVSTEEYFVLKNLCAKIDELKAAKA

>Mes275

MNIKQEDDHHHSHAHGDNHCHCGHDQEKAADAIVRDPICGMTVDPQAGKPSLGHGGRIYHFCSEHCRTKFAAAPEDYLTAKDPVCGMSVDRSTARYFLKAEGEKFYFCSAACQAKFEADPAAYRDGQRPTAKPAPKGTLYTCPMHPEVVSDRPGDCPKCGMALEPMGIPPTDEGPNPELVDFVRRLWVSAILALPLLALGMGPMLGLPLREAIGEPQATFIELLLATPVVLWAALPFFRRAWASVVNRSPNMWTLIGLGVGTAYLYSVVATLAPGIFPMSFRGHGAAVPVYFEAAAVIVALVFVGQVLELKARERTGSAIRALLDLAPKTARRIDAEGNESDVPVDDINVADRLRVRPGERVPVDGSVLEGQSTVDESMISGEPLPVEKSKGDPLTGGTINKNGTFVMSAEKVGADTVLSRIVDMVAKAQRSRAPIQGAVDRVSAVFVPAVVAVALLAFLAWAAIGPEPRMANGLLAAVAVLIIACPCALGLATPMSIMIATGRGAGEGVLIKDAEALERFSKGDTLIVDKTGTLTEGKPKLTDIAAFGRVGEDRLLSLAASLERGSEHPLAEAIVSGAEERGVPFVEVTGFEAKTGKGVQGIADGTMVALGNSAMLADLGIDPAALSEKTEALRGDGKTVMFVVFDGALAGLVAVADRIKPTTAAAIQALHDSGLKIIMATGDNERTARAVAKSLGIDEVRADVLPEGKKALIDELRSKGAIIAMAGDGVNDAPALAAADVGIAMGTGADVAMESAGITLVKGDLTGIVRARRLAEATMRNIRQNLGFAFGYNALGVPVAAGVLYPILGLLLSPMIAAAAMSLSSVSVISNALRLRFAKL

>Mes283

MTKFKLEYIWLDGYTPVPNLRGKTQIKEFDEFPTLEQLPLWGFDGSSTMQAEGSSDCVLKPVAIYPDPARTNGALVMCEVMMPDGHAHASNARATILDDEDAWFGFEQEYFFYQNGRPLGFPEQGYPAPQPYYTGVGYSNVGDVAREIVEEHLDLCLAAGINHEGINAEVAKGQWEFQIFGKGSKKAADQIWMARYLLQRLTEKYGIDIEYHCKPLGDTDWNGSGMHCNFSTKYLREVGGKEYFEALMASSDKNLMDHIAVYGPDNDKRLTGKHETAPWNKFSYGVADRGASIRVPHSFIKNDYKGYLEDRRPNSQGDPYQIVRRF

>Mes284

MTLDLAAFARDKSIKYFMISYTDLFGGQRAKLVPAEAIADMQKDGAGFAGFATWLDLTPAHPDLFAVPDASSVIQLPWKKDVAWVAADCVMDDRPVEQAPRVVLKRLVAEAAKEGLRVKTGVEPEFFLISADGSVISDQFDTAEKPCYDQQAVMRRYDVIAEICDYMLELGWKPYQNDHEDANGQFEMNWEYDDVLKTADKHSFFKFMVKSVAEKHGLRATFMPKPFKGLTGNGCHAHISVWDVDGRVNAFADKEMAFGLSAQGKTFLGGIMKHAPALAAITNPTVNSYKRINAPRTTSGATWSPNTVTWTGNNRTHMVRVPGPGRFELRLPDGAVNPYLLQAIIIAAGLEGIRSQADPGQHYDIDMYAEGHLVKDAPRLPLNLLDALRAFDADEGLKAAIGAEFSSAYLKLKHLEWNAYCSHFTQWERDSTLDI

>Mes285

MADQLTLEIISAINKLVKAENGERTSVALGEITTDTELTSLGIDSLGLADVLWDLEQLYGIKIEMNTADAWSNLNNIGDVVEAVRGLLTKEV

>Mes286

MNIKGSDNGSFIKGSPENDIIDGGKKNDWIDAGNGDDRIKAGDGQDSITAGPGHDIVWAGKGSDVIHADGGDDLLYSDASYPLYVTDPHRVIPHSGEGDDVLYAGPGSDILVAGDGADVLTGGDDGDAFVFRFHDPMVGTTHCYTSVMDFDTKQDRFVLDAADFGGDRNLFDANFINHSKGFPGEFVDTFYNGAAEGAHGEHVVVITDRGFASAAAAATAIDHEARGDIIVFHDQKTLGQDGETHGATLAYVDSANHAHAFAHVDNLHDMSDLTSLTAENFGFI

>Mes287

MNIRMVLLASAAAFAASTPVLAADAIVAAEPEPVEYVRVCDAYGTGYFYIPGTETCLKIEGYIRFQVNVGDNPGGDNDSDWDAVTAVRFSSRKSDTEYGPLTGVIVMQFNADNASDQDAILDSAYLDVAGFRAGLFYSWWDDGLSGETDDIGSVVTLHNSIRYQYESGTFYAGLSVDELEDGVYQGTFTPGVIPGTTDFTADDGPNNVGVAFGIGGTAGAFSYQVTGGWDVDNEDGAIRAMGTVEIGPGTFGLAGVYSSGPNSYYSSAEWAVAAEYAIKATDKLKITPGRWHGHVPEDFDGLGDAWKVGLTVDYQIVENFYAKASVQYLDPQDGEDSTSGYFACSVRSNHLVDAPGLRIGSTTISF

>Mes288

MIQTTFPDRAVMAELLAKMLWEIKAVHFNAAQPYKLASGMASPVYIDCRKLLSFPRIRSTVMDFAASTLLRDAGFEQFDCIAGGETAGIPFAALLADRLGLPMIYVRKQPKGHGRNAQIEGNMPEGSRVLVIEDLTTAGGSMFKFIDAVRAAGGIVDHGIALFFYGIFGEQRFADGKVRLHHIATWRNVLPSPGSRSSSTTRRCRKSSPSSMRRWLGRERMVA

>Mes289

MTLEKHAFKMQLNPGMEAEYRKRHDEIWPELVDLLHQSGASDYSIHLDRETNTLFGVLTRPKDHTMASLPDHPVMKKWWAHMADIMATNPDNSPVQSDLVTLFHMP

>Mes290

MSLHVSYVDKEMTDHARASQPGSAALAQGTQYSLLLKNQSAQPWTFYVYQKMPQPVANVFSLAWFCSPYQIRVGNQIKFTWELAYNFVWSDTGQLIPGVDFFASGVEDCSPSGRNTTTFSLSDGPGLTAPIKGDPAGSLVINDAGNVPNNRFSVGIGMSGTGTYVAQAGTNLLHTFTPTPSYWIAAGTNVTIGSVLSIDTITQTREAKFPSAVFNLVGVLQEDNTWDINPA

>Mes291

MDNKVAKNVEVKKGSIKATFKAAVLKSKTKVDIGGSRQGCVA

>Mes292

MNLTPREKDKLLISMAAMVARRRLERGVKLNYPEAIALISDFVVEGARDGRPVAELMEAGAHVIGRSQVMEGVAEMIHDVQVEATFPDGTKLVTVHEPIR

>Mes294

MNSPQPPDTTAAGSVHTAPTYTLRQLVMYFLRLGTLGFGGPVALAGYMHRDLVEAKQWITDADYKEGLALAQLAPGPLAAQLAIYLGYVHYRIVGATLVGVAFVLPSFLMVLALGWAYVRFGGLTWMQSVFYGVGAAVIGIIAISAYKLTKKSVGNDKLLWFIYLVLVAVTVITESEVAWLFLAAGVLVWFWRAPPKWLRQGKMNAFAATPLPAASGMMSTLDWPLLSQIGVFFAKAGAFVFGSGLAIVPFLYGGVVTEYHWLNDKQFVDAVAVAMITPGPVVITVGFIGYLVAGLPGACVAAAATFLPCYLFTVLPAPYFKKYGKLPAILAFVDGVTAAAIGAITGAVIVLAKRSIVDIPTALLALVTVALLLKFKKLSEPMIVAGAALIGLVAYPLLHH

>Mes295

MNALPSSPETAWLLLVVSLPTSASTARMRFWRGIKALGATALRDGAYLLPNLPGLRAPLQTLATDAASEDGKVWMLSVQAADDQQEAEYRALFDRSTEYAEWMVELSSARSTLSDSDEAELLRVARRHGRGIDAIRKVDFFPNEASARAELQWRDFNAAIDILLSPGEPHGVAGNIPRRDPTQYQGRQWATRQHLWVDRVACAWLIRRFIDPHATFLWLEDVRQCPDDALGFDFDGATFTHIGDRVSFEVLLASFGLDEDKGLARLGQMIHVLDVGGTPVAEASGFEAVLAGARERLPNDDALLDEVGYVLDSLYTHFSSPRKR

>Mes296

MIESILSGSVRYRWLVLFLTAVVAVIGAWQLNLLPIDVTPDITNKQVQINSVVPTMSPVEVEKRVTYPIETAIAGLNGVESTRSMSRNGFSQVTVIFKESANLYFMRQQVSERLAQARPNLPENVEPQMGPVSTGLGEVFHYSVEYQYPDGTGASIKDGEPGWQSDGSFLTERGERLDDRVSRLAYLRTVQDWIIRPQLRTTPGVADVDSLGGYVKQFVVEPDTGKMAAYGVSYADLARALEDTNLSVGANFIRRSGESYLVRADARIKSADEISRAVIAQRQNVPITVGQVARVKIGGELRSGAASRNGNETVVGSALMLVGANSRTVAQAVGDKLEQISKTLPPGVVIVPTLNRSQLVIATIETVAKNLIEGALLVVAILFALLGNWRAATIAALVIPLSLLVSAIGMNQFHISGNLMSLGALDFGLIIDGAVIIVENSLRRLAERQHREGRLLTLDERLQEVVQSSREMVRPTVYGQLVIFMVFLPCLTFQGVEGKMFSPMVITLMLALASAFVLSLTFVPAMVAVMLRKKVAETEVRVIVATKESYRPWLEHAVARPMPFIGAGIATVAVATVAFTFVGREFMPTLDELNLNLSSVRIPSTSIDQSVAIDLPLERAVLSLPEVQTVYSKAGTASLAADPMPPNASDNYIILKPKSEWPEGVTTKEQVIERIREKTAPMVGNNYDVTQPIEMRFNELIGGVRSDVAVKVYGENLDELAATAQRIAAVLKKTPGATDVRVPLTSGFPTFDIVFDRAAIARYGLTVKEVADTISTAMAGRPAGQIFDGDRRFDIVIRLPGEQRENLDVLGALPVMLPLSEGQARASVPLRQLVQFRFTQGLNEVSRDNGKRRVYVEANVGGRDLGSFVDDAAARIAKEVKLPPGMYIEWGGQFQNLQAATKRLAIIVPLCFILIAATLYMAIGSAALTATVLTAVPLALAGGVFALLLRGIPFSISAAVGFIAVSGVAVLNGLVLISAIRKRLDDGMAPDAAVIEGAMERVRPVLMTALVASLGFVPMAIATGTGAEVQKPLATVVIGGLVTATVLTLFVLPALCGIVLKRRTAGRPEAQAALEA

>Mes297

MMKNERRSVNWPMIAGVAAVAAAVGFGAAHLPVSEKSPASTQAPEAQKPQSAPVKPGLKEVKIPATYLAAANIAVEPVASAAVGTEILAPATVAALPGSEAVIVSRAAGAVQRVQRRLGDVVKAGDVLALVDSPEAAGMAAERKVAQAKADLARKTYEREASLFQQGVTPRQEMEAAKAALDVAQAEALRAATVAQSAHLASDGRSVAVVSPIAGKITAQSVTLGAFVAPQAELFRVAGTGAVQVEAAVTAADTSRIVAGSEATILLANGSPLSARVQAVTPTVTGSARVATVVVVPAQPTDRLVVGEGVQVRLRTAVADAAALSVPEDAVQNLDGRDVLFVRTQEGFRPMPVLVGTRSGGSAQILSGVQAGEQVATRNAFLVKAEMNKGGGDEE

>Mes298

MKQVISSFLCRPRFVGSAIWLLPVALSHAAEAPPFPNLLQQSLALAPAMVAQAANVRAAGADAAQAQAWLNPRIDTVLENLGAPSSDGLSQRQNTYSITQPFELGGKRGARIEVGERNFAAAQARERQAQVAYAAELAVAYATAEAALGRKILATENLARANEELAAARALVDSGKEASLRSAQAKASVAAAQAAEAAATNDATQALARLSAMSGASEPYTAVTSSLLTTQAVVPNAPAALAESPSVRAAEAERNALDAQVDVERKRWIPDVGVSAGVRRYGWTNSSGYVVGVTASIPLFDQNRNGINAAVERVAAAQARLDSVRLEANVARQSAISQVATADKQLAAASEGEQAAAEAYRMGRIGYESGKTPLMELLAVRRALVDARQLTIDARLARVRALAALAQADGRLAFEESR

>Mes299

MNPEDADRILAAQAASGNQRAFGQLVARHGVALAQAARSFGIPETDVDDVVQDTFVAAWHALDDFDPDRPFRAWLFRIGLNKMRDLYRFRRVRQFLFGAENLGDLELAGGVANDEPGPEQQVAARLELARVASTLGKLDTGSREVIVLTAIVGMSQPEAAAVLGLSVKAVEGRIGRARAKLSALLDADSEK

>Mes300

MMKSRTRRLSLSTLFGALLGVSVAAAWLYYSHRNEAGHGDLHEILHEAVPLDANEREILELKEDAFAQRRREIETRLRAANGKLADAIAKNPAWSPEVEAATQEVERAAGDLQRATLVHVFEMRAGLKPEHRPAYDRVLIDALRRGSQ

>Mes301

MADVEEWLTHARKVTQEASIGVDVTSIQECISAEPAQRVLVARRDAWRAICCAAFAALVAFAAINRVATIMLEKPAPTWVATPSAASPFGLLIGK

>Mes302

MKQKLMVGAFIAAVSLSAAAVDMSNVVKTYDLQDGSKVHVFKDGKMGMENKFGKSMNMPEGKVMETRDGTKIIMKGNEIFRLDEALRKGHSEGG

>Mes303

MFERIISFAIQQRWLVLLAVFGMAGLGIFSYNRLPIDAVPDITNVQVQVNTSAPGYSPLETEQRATYPIEVVMAGLPGLEQTRSLSRYGLSQVTVIFKDGTDVYFARQLVNQRIQEAKDNLPEGVVPAMGPISTGLGEIYLWTVEAEEGARKADGTAYTPTDLREIQDWVVRPQLRNVPGVTEINTIGGFNKQYLVAPSLERLASYGLTLTDVVNALNKNNDNVGAGYIERRGEQYLVRAPGQVASEDDIRNIIVGTAQGQPIRIRDIGDVEIGKELRTGAATENGKEVVLGTVFMLIGENSRAVSKAVDEKVASINRTMPEGVKIVTVYDRTRLVDKAIATVKKNLLEGAVLVIVILFLFLGNIRAALITATIIPLAMLFTFTGMVNYKISANLMSLGALDFGIIIDGAVVIVENCVRRLAHAQEHHGRPLTRSERFHEVFAAAKEARRPLIFGQLIIMIVYLPIFALTGVEGKMFHPMAFTVVLALLGAMILSVTFVPAAVALFIGERVAEKENRLMLWAKRRYEPLLEKSLANTAVVLTFAAVSIVLCVAIAARLGSEFIPNLNEGDIAIQALRIPGTSLSQSVEMQKTIETTLKAKFPEIERVFARTGTAEIASDLMPPNISDGYIMLKPEKDWPEPKKTHAELLSAIQEEAGKIPGNNYEFSQPIQLRFNELISGVRSDVAVKIFGDDNNVLSETAKKVSAVLQGIPGAQEVKVEQTTGLPMLTVKIDREKAARYGLNMSDVQDAVATGVGGRDSGTFFQGDRRFDIVVRLPEAVRGEVEALRRLPIPLPKGVDARTTFIPLSEVATLEMAPGPNQISRENGKRRIVISANVRGRDIGSFVPEAEAAIQSQVKIPAGYWMTWGGTFEQLQSATTRLQVVVPVALLLVFVLLFAMFNNIKDGLLVFTGIPFALTGGILALWIRGIPMSITAAVGFIALCGVAVLNGLVMLSFIRSLREEGHSLDSAVRVGALTRLRPVLMTALVASLGFVPMAIATGTGAEVQRPLATVVIGGILSSTALTLLVLPVLYRLAHRKDEDAEDTREPVTQTHQPDQGRQPA

>Mes304

MAISNKQKAAIAAIVLVGGVATGGVLLSGRSAPEEQGGHSESKGHGDTEHHGKQAAEADHKDDKSHGDGEHHEVKKGPNGGALFSRDGYDVEIGTAESKGEARIRLWVSKSGKAVANGVAATGQLVRATGESQALKFVVSGDALESQQPVAEPHVFDVTANVTLPGSSSPLAVRLSKEEGKIELTADQLAKTGVVVQTAGSAKVQAGVQFPGEIRFNEDKTAHVVPRLAGVVESVPANIGQQVKKGQVLAVIASTGLSDQRSELLAAQKRLDLARVTYDREKKLWEQKISAEQDYLSARNALQEAQISVQNAQQKLTAIGASNSSTALNRYELRAPFDGMIVEKHISLGEAVADNANVFTLSDLSSVWAEFVVSAKDVERVRIGEKASINSASSDVKADGTVSYVGSLLGEQTRTAKARVTLTNPQMAWRPGLFVTVDVFGADVEVPVAVKTEAVQDVNGESVVFVAVQGGFVPQPVKVGRTNGKVIEIVEGLKPGARYAAANSFVLKAELGKSSAEHGH

>Mes305

MRRLFLPLGLAVAFLSPNFAVAQSDTGTSMVPVFPREAAGPLTLEAALSLAAGSNFNLSAAAKELDSTEGGIMQARVIPNPELKTLVEDTRKSTRTSTAQMNIPIELGGKRSARINAAERTRELAQATLAGVRGDIRAQVIESFFSVLIAQERVKLATGSADIAARGAQAASRRVAAGKISPVDETKARVEQANAELELAEATASLQSARQALTALWGNASPQFAEAQGNLDALPSRPAPELLQKELENSPLVAASRAELDRRQALVGVERSRQYPDLTVSLGAKRDTEANRNMAVIGVAIPLPIFDRNQGNLYSAIRQADKAQDEYLANRISLTRNLLMASNQLSVSRASAQTLKQTVLPGAEQAFNAATIGFEAGKFNYLDVLDAQRTLFQARIRYLGVLGQTYQAATTIDRILGR

>Mes306

MGAGHSHDHPGGNERSLKIALALTGTFLIAEVVGGVMTKSLALISDAAHMLTDTVALAIALAAIAIAKRPADKKRTFGYYRFEILAAAFNALLLFGVAIYILYEAYLRLKSPPQIESTGMFVVAVLGLIINLISMRMLSSGQSSSLNVKGAYLEVWSDLLGSVGVIAGAIIIRFTGWAWVDSAIAVLIGLWVLPRTWILLKSSLNVLLEGVPDDVDLAEVEKQILATPGVKSFHDLHIWALTSGKASLTVHVVNDTAVNPEMEVLPELKQMLADKFDITHVTIQFELAPCEQADAAQHFNASPALVGSKSLAAGGN

>Mes307

MRRFVLIFVLLILPFQFSWAAAARYCQHEKATATWHLGHHEHRHQQPEGKTDAEKKPFVDTDCGVCHLVSLPFVYGQTQDVLIANRVEVTDTQHSSEFSSLNARAPDRPQWQRLA

>Mes308

MRVLVVEDEPRTAEYLQKGLSESGFVVDIANNGGDGLHMAEETDYDVIILDVMLPGMDGWTVIKSIRSKSETPVLFLTALDDVADRVRGFELGADDYLVKPFAFAELLARIRRCLRQSTSKESERLRIADLDIDVLGRRVFRGTTRIELTNQEFSLLHLLMRRRGEVLSRTTIASQVWGVNFDTDTNVVDVAIRRLRSKVDDPFDQKLIHTVRGMGYVLDPERGR

>Mes309

MRPGTSITPLSLTRRLGLFFALVLSIALASMGAFAYYSLAAQLEARDDEVVKGKLEQVEHFLREVDGVQGVPAAQHRFDDLVRGYSDLIVRVTALDGRLLFRTGNDALLEGTDQAAVTGKSSLMFQSADAVLGRDGTRATVFVAKSGEDRKQVTARFRTTLVLGTTVGVILTALVGAAITRRELEPAHVLIKQINRISVERLSYRVDMPPKPTEVRDIASAFNAMLQRLEDGYQKLSRFSADLAHDLRTPLNNLIGHAEVALSRDRTGPEYVALVEESLVEYQRLARMIDAMLFLARADSANVALELTELQLNAELRKLSAYFSVLAEERSVVIRVSGDATLVADAILFQRAINNVLSNAVRHAWPNSMIDLVVRREAAHCCIDITNVGDPIPERELSLIFDRFFRGDRARSNSSQSTGLGLAIVLSIMELHGGDASAVSGLDGKTRFTLRFPLNGAEASARVSVGRPSQDRPVVG

>Mes311

MPQAPQLHDGRRIWDISPAVSPATPVWPGDTPFQHDPAWQLDEHCPVNVGRITMSPHTGAHADAPLHYAADGAPIGAVPLDAYLGPCRVIHCIGAAPRVEPQHIAHALAGTPPRVLLRTYAQAPQGKWDSAFCAVAPETISLLARHGVRLIGIDTPSLDPETSKTMDAHHAVRDHQLAILEGIVLDEVPAGDYELIALPLRLATLDASPVRAVLRELP

>Mes312

MLYEMKPLGCEPAKLTGLSEKLIFSHYENNYGGAVKRLNAITATLAELDMATAPVFTLNGLKREELIATNSMILHEVYFDSLGDGGSLDGALKTAIERDFGSVERWQAEFTAMGKALGGGSGWVLLTYSPRDGRLVNQWASDHAHTLAGGTPVLALDMYEHSYHMDYGAKAAAYVDAFMQNIHWQRAATRFAAAVRD

>Mes313

MSEFKGCPMGHGAAPQNGDGGDSGDTGNGWHGAQMDFARDMSYGDYLGLDQILSAQHPLSPDHNEMLFIVQHQTTELWMKLMLHELRAARDGVKSDQLQPAFKMLARVSRIMDQLVQAWNVLATMTPPEYSAMRPYLGASSGFQSYQYREIEFILGNKNAAMLRPHAHRPEHLELVETALHTPSMYDEAIRLMARRGFQIDPEVVERDWTQPTQYNASVEAAWLEVYRNPSAHWELYELGEKFVDLEDAFRQWRFRHVTTVERVIGFKRGTGGTEGVSYLRRMLDVVLFPELWKLRTDL

>Mes314

MSLKHFLNTQDWSRAELDALLTQAALFKRNKLGSELKGKSIALVFFNPSMRTRTSFELGAFQLGGHAVVLQPGKDAWPIEFNLGTVMDGDTEEHIAEVARVLGRYVDLIGVRAFPKFVDWSKDREDQVLKSFAKYSPVPVINMETITHPCQELAHALALQEHFGTPDLRGKKYVLTWTYHPKPLNTAVANSALTIATRMGMDVTLLCPTPDYILDERYMDWAAQNVAESGGSLQVSHDIDSAYAGADVVYAKSWGALPFFGNWEPEKPIRDQYQHFIVDERKMALTNNGVFSHCLPLRRNVKATDAVMDSPNCIAIDEAENRLHVQKAIMAALVGQSRP

>Mes319

MTQTNGFDALHAHAQRLRGAAIPALLAAEPERPTQYARQVGPLYFNFARQKYDRAALDALFAIARERDLSGAFQRLFRGEQVNVTEQRAALHTALRGDLTDAPVASEAYATAEEVRQRMGSLIQQLEATDVTDIVSVGIGGSDLGPRLVADALRAPSGARFRVHFVSNVDGAAMQRTLATLDPARTAGILISKTFGTQETLLNGSILHAWLGGSERLYAVSANPERAAKAFDIAPGRVLPMWDWVGGRYSLWSAVGFPIALAIGFERFEQLLEGAAQFDAHVLNTPLEENVAVLHGLTAVWNRNLLGSATHAVMTYDQRLALLPAYLQQLVMESLGKRVKLDGSAVDSDTVSVWWGGAGTDVQHSFFQALHQGTSVVPADFIGTVHNDDPYAENHTALMANVLAQTEALANGQDSSDPHRSYPGGRPSTVILLDALTPQALGALISMYEHSVYVQSVMWGINAFDQFGVELGKQLASQLLPALKGESVDVADPVTRELLNKLRG

>Mes320

MSERMTPRLFPVSLLIGLLAGCATTPPPDVRRDARLDPQVGAAGATQTTAEQRADGNASAKPTPVIRRGSGTMINQSAAAAPSPTLGMASSGSATFNFEGESVQAVVKAILGDMLGQNYVIAPGVQGTVTLATPNPVSPAQALNLLEMVLGWNNARMVFSGGRYNIVPADQALAGTVAPSTASPSAARGFEVRVVPLKYISASEMKKVLEPYARPNAIVGTDASRNVITLGGTRAELENYLRTVQIFDVDWLSGMSVGVFPIQSGKAEKISADLEKVFGEQSKTPSAGMFRFMPLENANAVLVITPQPRYLDQIQQWLDRIDSAGGGVRLFSYELKYIKAKDLADRLSEVFGGRGNGGNSGPSLVPGGVVNMLGNNSGGADRDESLGSSSGATGGDIGGTSNGSSQSGTSGSFGGSSGSGMLQLPPSTNQNGSVTLEVEGDKVGVSAVAETNTLLVRTSAQAWKSIRDVIEKLDVMPMQVHIEAQIAEVTLTGRLQYGVNWYFENAVTTPSNADGSGGPNLPSAAGRGIWGDVSGSVTSNGVAWTFLGKNAAAIISALDQVTNLRLLQTPSVFVRNNAEATLNVGSRIPINSTSINTGLGSDSSFSSVQYIDTGVILKVRPRVTKDGMVFLDIVQEVSTPGARPAACTAAATTTVNSAACNVDINTRRVKTEAAVQNGDTIMLAGLIDDSTTDGSNGIPFLSKLPVVGALFGRKTQNSDRREVIVLITPSIVRNPQDARDLTDEYGSKFKSMRPMDVHK

>Mes321

MEQRSAETRIVEALLERRRLKDTDLVRARQLQAESGMGLLALLGRLGLVSERDHAETCAEVLGLPLVDARQLGDTPPEMLPEVQGLSLRFLKQFHLCPVGERDGRLDLWIADPYDDYAIDAVRLATGLPLLLQVGLRSEIDDLIERWYGQGRSAMGTIVETADGDASSTDDIEALRDLASEAPVIRLVNLVIQHAVELRASDIHIEPFESRLKVRYRVDGVLVEGESPPAKLTAAVISRIKIMAKLNIAERRLPQDGRIMLRVQGKELDLRVSTVPTAHGESVVMRLLDRETVVFDFYKLGFTEDFLPQFRKVLEQPHGIMLVTGPTGSGKTTTLYTALSQLNTSDVKIITVEDPVEYQIEGINQIQAKPQIGLDFANALRSIVRQDPDIIMIGEMRDLETARIAIQSALTGHLVLSTLHTNNAAGGITRLLDMGVEDYLLTSTINGILAQRLVRKLDLANAERYAASPEEIERFDLRRLQPDGEIFLYRPRATAAAPTGYLGRTTIVEFLVMNDELRRAVMRRAGMGEIEQLARKSGMRTMYEDGLSKALRGETTIEEVLRVTEDA

>Mes322

MKLYTKPGACSLADHIVLRWSCLPFELTVVDAATMKSPDYLRLNPAGAVPLLVVDQWALTQNAAILNYIADTAPLTGLGGDGTARSRAEINRWIAFVNADLHPTFKPLFGSTAYLQEDALIQRSHEDARTKLRTLYTRVDAHLQGRNWLAGDTHTGADAYLFVTLRWAHKAGVDLSGLSALDAFFQRMLADADVQAALQAEGLN

>Mes323

MSIFRTASTLALATALALAAGPAFSYSINNSRQIVDDSGKVVQLKGVNVFGFETGNHVMHGLWARNWKDMIVQMQGLGFNAVRLPFCPATLRSDTMPASIDYSRNADLQGLTSLQILDKVIAEFNARGMYVLLDHHTPDCAGISELWYTGSYTEAQWLADLRFVANRYKNVPYVLGLDLKNEPHGAATWGTGNAATDWNKAAERGSAAVLAVAPKWLIAVEGITDNPVCSTNGGIFWGGNLQPLACTPLNIPANRLLLAPHVYGPDVFVQSYFNDSNFPNNMPAIWERHFGQFAGTHALLLGEFGGKYGEGDARDKTWQDALVKYLRSKGINQGFYWSWNPNSGDTGGILRDDWTSVRQDKMTLLRTLWGTAGNTTPTPTPTPTPTPTPTPTPTPTPTPGTSTFSTKVIVDNSWNGGYCNRVQVTNTGTASGTWSIAVPVTGTVNNAWNATWSQSGSTLRASGVDFNRTLAAGATAEFGFCAAS

>Mes324

MDLTDTQQAILALIAERIDADGVPPSQTEIARAFGFKGIRAAQYHLEALEHAGAIRRVPGQARGIRLAGQGAQTRTAPVSEVARDDVLRLPVLGRVAAGLPIGADIGSDDFVVLDRVFFSPSPDYLLKVQGDSMRDEGIFNGDLIGVHRTRDARSGQIVVARIDEEITVKLLKIGKDRIRLLPRNPDYAPIEVLPDQDFAIEGLYCGLLRPNR

>Mes325

MADNVAGNDRLIWIDLEMTGLDTDRDSIIEIATIVTDAQLNVLAEGPELAIAHSLETLEAMDEWNRNQHRRSGLWQRVLDSQVTHAQAEAQTVAFLSEWIRAGASPMCGNSICQDRRFLHRQMSRLERYFHYRNLDVSTIKELARRWAPAVASGFAKSSAHTALSDVRDSIDELRHYRQFMGTLGGDNGGGVQN

>Mes326

MRTLYPEITPYQQGSLKVDDRHTLYFEQCGNPHGKPVVMLHGGPGGGCNDKMRRFHDPAKYRIVLFDQRGSGRSTPHADLVDNTTWDLVADIERLRTHLGVDRWQVFGGSWGSTLALAYAADPSAAGHQLVLRGIFLLRRFELEWFYQEGASRLFPDAWEHYLNAIPPVERADLMSAFHRRLTSDDEATRLAAAKAWSVWEGATSFLHVDEDFVTGHEDAHFALAFARIENHYFVNGGFFEVEDQLLRDAHRIADIPGVIVHGRYDVVCPLQSAWDLHKAWPKAQLQISPASGHSAFEPENVDALVRATDGFA

>Mes327

MSTISRDSCPALRAGVRLQHDRARDQWVLLAPERVVELDDIALVVAQRYDGTQSLAQIAQTLAAEFDADASEIETDVIELTTTLHQKRLLRL

>Mes328

MSSSIVVIAAGERSTEAVLAAEALRRAATAAGRSVTIEIRSDQGVLGALPTELTNGAAHVLIVGDADADTARFGDAQLLHLSLGAVLDDPAAAVSQLAATTAPASTSATTDASGAGGKRIVAITSCPTGIAHTFMAAEGLQQAAKKLGYQMRVETQGSVGAQDALTDEEIRAADVVIIAADREVDLARFGGKRLFKSGTKPAINDGPALIQKALAEAGVHGGAAPVAGANATSDAKGNARTGAYKHLMTGVSFMLPFVTAGGLLIALAFALGGIYAGDDAHQGTLAWSLFQIGAKAGFTLMVPALAGYIAYSIADRPGIAPGMIGGLVAANLNAGFLGGIIAGFIAGYGVAALNRYIKLPRNLEGLKPVLILPVLGTLLVGLAMMYVFGQPVADLLAWLTAWLRGMQGSSALLLGLLLGGMMAFDMGGPVNKAAYAFSTGLIASQVYTPMAAAMVAGMTPPLGIALATWVFRNRFTVEERGSATAAGVLGLAFVTEGAIPYAARDPLRTIPALVIGSAVAGAISMTAGAELKAPHGGIFVLLIPNAVTHLLNYVLALVVGVVVTAVALRLLKKPVADVIA

>Mes329

MSELSYRRILLKLSGEALMGDGDYGIDPKVINRLAHEVIEAQQAGAQVALVIGGGNIFRGAGLAASGMDRVTGDHMGMLATVINALAMQDALEKLGAKVRVMSAIKINDVCEDFIRRRAIRHLEKGRIAIFAAGTGNPFFTTDSGAALRAIEIGADLLLKATKVDGVYDKDPKKHSDAVRYDSLTYDEVIMQGLEVMDTAAFALARDSDLPLRIFGMSEPGVLLRILHGAQIGTLVQGRS

>Mes330

MSEQAPAPKRGRRFKEQTPVQRALGLLVRREHSKKELNRKLQARGIEPEAAQAAVERLAGEGWQDDVRFAASVVRNRASSGYGPLHIRAELGTHGLDSDAVSAAMATFEGDWTENALDLIRRRFGEDGPVDLAQRRKAADLLARRGFDGNSIRLATRFDLED

>Mes332

MHEQLSPRDQELEARLVELETRLSFQEQALTELSEALADARLTGARNAELIRHLLEDLGKVRSTLFADAADEPPPPHY

>Mes334

MRLEMIGLRTWLLATVVGWALLVCVLAVAGLGKRVELLPDDPALVQRLPALPAPAPERLGPFEKYAEIAAHPAFAEDRLPHPFFLSGNDGSGAASTVRLTGVLLTSTFKMATLTLDPADSVRVQLGGDAVKGYRLLALQPRSATIEGPGGTQTLELQVFNGQGGQPPTAIGGRPQAPGAVPPLPPNVPPAPATPAPPPAEVPQQQPGGQAPPTVPPQRSDGAQEAPRPSDEQMRAIRERIEARRRQLQQQRQGGSTPGQTQ

>Mes336

MNPQTDNVFYATNAFTGEALPLAFPVHTEVEVNQAATAAAKVARDFRRLNNSKRASLLRTIASELEARSDDIIARAHLETALPEVRLTGEIARTANQLRLFADVVNSGSYHQAILDTPNPTRAPLPKPDIRRQQIALGPVAVFGASNFPLAFSAAGGDTASALAAGCPVIVKGHTAHPGTSQIVAECIEQALKQEQLPQAIFTLLQGNQRALGQALVSHPEIKAVGFTGSVGGGRALFNLAHERPEPIPFYGELGAINPTFIFPSAMRAKADLADQFVASMTMGCGQFCTKPGVVFALNTPETQAFIETAQSLIRQQSPSTLLTPGIRDSYQSQVVSRGSDDGIDVTFSQAESPCVASALFVTSSENWRKHPAWEEEIFGPQSLIVVCENVADMLSLSEMLAGSLTATIHATEEDYPQVSQLIPRLEEIAGRLVFNGWPTGVEVGYAMVHGGPYPASTHSASTSVGAEAIHRWLRPVAYQALPESLLPDSLKAENPLEIARAVDGKAAHS

>Mes337

MLKHSLIAASVITTLAGCSSLQSSEQQVVNSLADNLDIQYEVLTNHGANEGLACQDMGAEWASCNKVNMTLVNQGEAVDSKDWAIYFHSIRLILDVDNEQFKISRVTGDLHKLEPTDKFDGFAAGEEVVLPLVGEYWQLFETDFMPGAFVSAPNAEPKMIASLNTEDVASFVTGLEGNNLKRTPDDNNVFANAVSRFEKNEDLATQDVSTTLLPTPMHVEAGKGKVDIADGIALPKDAFDATQFAAIQDRAEVVGVDVRGDLPVSITVVPADFTGELAKSGAYEMSIKGDGIVIKAFDQAGAFYAVQSIFGLVDSQNADSLPQLSIKDAPRFDYRGVMVDVARNFHSKDAILATLDQMAAYKMNKLHLHLTDDEGWRLEIPGLPELTEVGANRCFDTQEKSCLLPQLGSGPTTDNFGSGYFSKADYVEILKYAKARNIEVIPEIDMPAHARAAVVSMEARYDRLMEEGKEAEANEYRLMDPQDTSNVTTVQFYNKQSFINPCMESSTRFVDKVISEVAAMHQEAGAPLTTWHFGGDEAKNIKLGAGFQDVNAEDKVSWKGTIDLSKQDKPFAQSPQCQTLITDGTVSDFAHLPSHFAEEVSKIVAEKGIPNFQAWQDGLKYSDGEKAFATENTRVNFWDVLYWGGTSSVYEWSKKGYDVIVSNPDYVYMDMPYEVDPKERGYYWATRATDTRKMFGFAPENMPQNAETSVDRDGNGFTGKGEIEAKPFYGLSAQLWSETVRNDEQYEYMVFPRVLAAAQRAWHRADWENDYKVGVEYSQNSNLVDKASLNQDYNRFANVLGQRELAKLEKSGIDYRLPVPGAKVEDGKLAMNVQFPGVTLQYSLDGENWLTYADNARPNVTGEVFIRSVSATGEKVSRITSVK

>Mes338

MSDKPKTKPLPSFVEGRLDFYIQDLIEQNENQKHLVLGKRPQQGAVVMQSNDYLSLSHNLQIQQAHRDAIYEHDDNVVMSAIFLQDDDSKPAFETQLAEYVGMGSCLLSQSGWAANIGLLQTICPPETPVYIDFFAHMSLWEGIRAAGAQAHPFMHNNMNHLRKQIQRNGSGVIVVDSVYSTIGTIAPLRDIYEMAREFDCALVVDESHSLGTHGPNGSGLVKALELTEQVDFITVSLAKTFAYRAGAILGPEKLARTLPFVAFPAIFSSTVLPQEIVRLEKTLEVIRSADDKRTMLFKRAKELRTGLKQIGFHIRSESQIVALECGSERNTERVRDFLEERNVFGAVFCRPATGKNKNIIRFSINADMTSRDIDHVLTACQEAYNHPELEFA

>Mes342

MTIQCKVKSIQPLACNTYQILLHPESPVPFKAGQYLMVVMGEKDKRPFSIASSPCRHEGELELHIGAAEHNAYALEVVEAMQAALETDGHIEIDAPHGDAWVQEESERPLLLIAGGTGFSYVRSILDHCVAQNKTNPIYLYWGARDNCQLYAKEELVEIADKFANVHFVPVVEEAPADWQGKVGNVLQAVSEDFESLENYDIYIAGRFEMAGAAREQFTQNKKAKSERMFADAYAFI

>Mes343

MNNTIETILAHRSIRKFTAVPITDEQRQTIIQAGLAASSSSMLQVVSIVRVTDSEKRNELAQFAGNQAYVESAAEFLVFCIDYQRHATINPDVQADFTELTLIGAVDSGIMAQNCLLAAESMGLGGVYIGGLRNSAAQVDELLGLPENSAVLFGMCLGHPDQNPEVKPRLPAHVVVHENQYQELNLDDIQSYDQTMQAYYASRTSNQKLSTWSQEVTGKLAGESRPHILPYLNSKGLAKR

>Mes344

MELRMKKVSVIAAAVAATLAAGSAFAVDFHGYMRAGVGVSADGGQQVTFEKQKIGRLGNEGDIYGEIQLGQEVYNNNGKTFYVDSMIAVTSNGSNDWEGTASNCGTTVSHPDGQDPSATTKCVDDAEFALRQFNVQAKGVLDFAPEATLWAGKRYYQRHDIHISDFYYWNISGAGAGVEGIEAGPGQLSFAWVRNDRNDNFKLGENNPGDTPDAGNDGGAANVNTLDLRYAGLPVWENGSLELGLNYALVNETDDASDAAKDAKDGVMFTAELTQGLDSGFNKTVFQYGTEGYSKTMAFYGDGSWYGAEANDGASGYRLINWGVIGMGQNWEMGHQLVYGVGEDMWDGQDKLETMSVVVRPMYKWDDNHKTIFEAGYAIDDNDGAENKFGKLTVAQAWSAGSSFWARPEIRVYASYLTADKDDNSNAFDGGRSDDTFQFGVQAEAWW

>Mes345

MKFGNFLLTYQPPELSQTEVMKRLVNLGKASEGCGFDTVWLLEHHFTEFGLLGNPYVAAAHLLGATETLNVGTAAIVLPTAHPVRQAEDVNLLDQMSKGRFRFGICRGLYDKDFRVFGTDMDNSRALMDCWYDLMKEGFNEGYIAADNEHIKFPKIQLNPSAYTQGGAPVYVVAESASTTEWAAERGLPMILSWIINTHEKKAQLDLYNEVATEHGYDVTKIDHCLSYITSVDHDSNRAKDICRNFLGHWYDSYVNATKIFDDSDQTKGYDFNKGQWRDFVLKGHKDTNRRIDYSYEINPVGTPEECIAIIQQDIDATGIDNICCGFEANGSEEEIIASMKLFQSDVMPYLKEKQ

>Mes346

MKFGLFFLNFMNSKRSSDQVIEEMLDTAHYVDQLKFDTLAVYENHFSNNGVVGAPLTVAGFLLGMTKNAKVASLNHVITTHHPVRVAEEACLLDQMSEGRFAFGFSDCEKSADMRFFNRPTDSQFQLFSECHKIINDAFTTGYCHPNNDFYSFPKISVNPHAFTEGGPAQFVNATSKEVVEWAAKLGLPLVFRWDDSNAQRKEYAGLYHEVAQAHGVDVSQVRHKLTLLVNQNVDGEAARAEARVYLEEFVRESYSNTDFEQKMGELLSENAIGTYEESTQAARVAIECCGAADLLMSFESMEDKAQQRAVIDVVNANIVKYHS

>Mes347

MNNQCKTIAHVLRVNNGQELHVWETPPKENVPFKNNTILIASGFARRMDHFAGLAEYLSENGFHVFRYDSLHHVGLSSGSIDEFTMTTGKNSLCTVYHWLQTKGTQNIGLIAASLSARVAYEVISDLELSFLITAVGVVNLRDTLEKALGFDYLSLPIDELPNDLDFEGHKLGSEVFVRDCFEHHWDTLDSTLDKVANTSVPLIAFTANNDDWVKQEEVYDMLAHIRTGHCKLYSLLGSSHDLGENLVVLRNFYQSVTKAAIAMDGGSLEIDVDFIEPDFEQLTIATVNERRLKAEIESRTPEMA

>Mes348

MKLMLSLGSLSANSLPIEKKQQVLIDLVIRTYQSHERTELFKAITEYRKNQLIALFPEHANKSYSIIFELMDYRDLIERYPSTLSEEATLLEKVVGQCFMHWLDFWCECEIAAIKAKFPLKENELPAPQLLFEDSAYYGALVERVEDTQLMVQIPSHPQAMPLSDAITLSNLELFIQGEKWYEMLSLLSLSQVGKHFIVLKHPVQDSCPTLVASALIQNWSVRDTWLSYAPQFSNEQWNYCFPSYGYSEFTRLQLFTPSSLSKCYSLPEFDNEFKLQLSDTQAVCEVLRLTVSGNAQQKLYFLYLAQKELMSVLHQAGYKIGFTIIEQPFMLNFYRAIDAKAYFHSGYCDLNDDGKQTYRGFWNFEMMVKAFSNIDFRGYKRAVRASRKRGSLERDEHV

>Mes349

MFDFSLEAIVYAKAITLLATVAVVMMWLFYYCYRLKQKNEVIFGTHHAAYIAYSVCIIAWISSNAYFHTDLLPELGASAGMFMAKFANLASFFAFAFAYYFSCQLAAEQRKGKVHRWQQGIFVSLTVYSLFINLRPGLTVEHVDIVGPSQFIIEFGPHTSYFFIGLVSFVVLTLVNLVAMRTNSSKLTLAKTNYMIAGILVFMLSTAVIHLGMTYFMGDFSLTWLPPALSISEMLFVGYALLTSRFYSVKYIAYLALSVLLVCAIFVLPLGAIFIPLTESNQWLIAIPICALIGITWQLLYKKTSRYASFLIYGDKKTPVQQILSLEEDFKLSIDDAMRRLGKLLQIPNDKLRLVTSNYNETFYEEYLSSNRSVLVFDELSEELEYKVSAKRSMKALYDKMSSNNTALVMPLFGQGKSVTHLLISPHKSNNQMFSNEEISAVQTLLTRVQSTIEADRRIRQSRALANSIAHEMRNPLAQVQLQFEALKQHIENHAPVEQITLDIENGQAAIQRGRQLIDIILREVSDSSPEHEPIAMTSIHKAVDQAVSHYGFENEKIIERIRLPQHTDFVAKLNETLFNFVIFNLIRNAIYYFDSYPDSQIEISTKTGPYENTLIFRDTGPGIDETISHKIFDDFFSYQKSGGSGLGLGYCQRVMRSFGGRIECKSKLGTFTEFHLYFPVVPNAPKADTLRTPYFNDWKQNKRSNEHKVAPNVQINNQSPTVLIVDDKEVQRALVQMYLNQLGVNSLQANNGENAVEVFKANHVDLILMDVQMPVMNGFDASQRIKELSPQTPIVALSGESGERELDMINKLMDGRLEKPTTLNALRHVLGNWLNKNTASSACEAERE

>Mes351

MKKALLFSLISMVGFSPASQATQVLNGYWGYQEFLDEFPEQRNLTNALSEAVRAQPVPLSKPTQRPIKISVVYPGQQVSDYWVRNIASFEKRLYKLNINYQLNQVFTRPNADIKQQSLSLMEALKSKSDYLIFTLDTTRHRKFVEHVLDSTNTKLILQNITTPVREWDKHQPFLYVGFDHAEGSRELATEFGKFFPKHTYYSVLYFSEGYISDVRGDTFIHQVNRDNNFELQSAYYTKATKQSGYDAAKASLAKHPDVDFIYACSTDVALGAVDALAELGREDIMINGWGGGSAELDAIQKGDLDITVMRMNDDTGIAMAEAIKWDLEDKPVPTVYSGDFEIVTKADSPERIEALKKRAFRYSDN

>Mes352

MTTTRSNIKKRRSLATLITKIIILVLAPIILGIFIQSYYFSKQIIWQEVDRTKQQTSALIHNIFDSHFAAIQIHHDSNSKSEVIRDFYTDRDTDVLNFFFLSIDQSDPSHTPEFRFLTDHKGIIWDDGNAHFYGVNDLILDSLANRVSFSNNWYYINVMTSIGSRHMLVRRVPILDPSTGEVLGFSFNAVVLDNNFALMEKLKSESNVDNVVLVANSVPLANSLIGDEPYNVADVLQRKSSDKRLDKLLVIETPIVVNAVTTELCLLTVQDNQSVVTLQIQHILAMLASIIGMIMIALMSREWIESKVSAQLESLMSYTRSAREEKGFERFGGSDIEEFDHIGSTLESTFEELEAQKKSFRDLFNFALSPIMVWSEESVLIQMNPAARKELVIEDDHEIMHPVFQGFKEKLTPHLKMAAQGATLTGVNVPIGNKIYRWNLSPIRVDGDISGIIVQGQDITTLIEAEKQSNIARREAEKSAQARADFLAKMSHEIRTPINGILGVAQLLKDSVDTQEQKNQIDVLCHSGEHLLAVLNDILDFSKIEQGKFNIQKHPFSFTDTMRTLENIYRPICTNKGVELVIENELDPNVEIFTDQVRLNQILFNLVSNAVKFTPIGSIRLHAELEQFYGAENSVLVVELTDTGIGIESDKLDQMFEPFVQEESTTTREYGGSGLGLTIVKNLVDMLEGDVQVRSSKGGGTTFVITLPVKDRERVLRPLEVSQRIKPEALFDESLKVLLVEDNHTNAFILQAFCKKYKMQVDWAKDGLDAMELLSDTTYDLILMDNQLPHLGGIETTHEIRQNLRLGTPIYACTADTAKETSDAFMAAGANYVMLKPIKENALHEAFVDFKQRFLVERT

>Mes353

MDSIAKRPRTRLSPLKRKQQLMEIALEVFARRGIGRGGHADIAEIAQVSVATVFNYFPTREDLVDEVLNHVVRQFSNFLSDNIDLDIHARENIANITNAMIELVSQDCHWLKVWFEWSASTRDEVWPLFVTTNRTNQLLVQNMFIKAIERGEVCDQHEPEHLANLFHGICYSIFVQANRSKSEAELTNLVSAYLDMLCIYNREHH

>Mes354

MNTDVLNQQKIEELSAEIGSDNVPVLLDIFLGEMDSYIGTLTELQGSEQLLYLKEISHALKSSAASFGADRLCERAIAIDKKAKANQLQEQGMETSEMLALLHITRDAYRSWTN

>Mes355

MASNNDSIKKTLGVVVGLSLVCSIIVSTAAVGLRDQQKANAVLDKQSKIVEVAGIDAEGKKVPELFAEYIEPRLVDFKTGDFVEKAEDGSTAANYDQRKAAKDPAESIKLTADEDKAKILRRANTGIVYLVKNGDDISKVIIPVHGNGLWSMMYAFVAVETDGNTVSGITYYEQGETPGLGGEVENPVWRAQFVGKKLFDENHKPAIKIVKGGAPEGSEHGVDGLSGATLTGNGVQGTFDFWLGDMGFGPFLAKVRDGGLN

>Mes356

MKFVDEAVVKVQAGDGGSGVVSFWREKFITKGGPDGGDGGDGGDVYIQADENLNTLIDYRFQRFYEAERGENGRGGNCTGKRGKDITLRVPVGTRAVDIHTNEIVAEVAEHGKKVMVAKGGWHGLGNTRFKSSVNRAPRQRTLGTKGEIREIRLELLLLADVGMLGLPNAGKSTFIRAVSAAKPKVADYPFTTLIPSLGVVSVVPEKSFVVADIPGLIEGAADGAGLGIRFLKHLERCRVLLHMIDIMPIDQSDPIQNALTIIDELEQYSEKLAGKPRWLVFNKTDLMPEEEANEKIQEILDALGWEDEYFKISAINRNGTKELCYKLADFMENLPREEEEVAEEDKVNFMWDDYHKDAIAGKDVITEEDDDDWDDCDDEDDDGHVVYVRD

>Mes358

MKGLRKSILCLVLSAGVIAPVTSGMIQSPQKCYAYSINQKAYSNTYQEFTNIDQAKAWGNAQYKKYGLSKSEKEAIVSYTKSASEINGKLRQNKGVINGFPSNLIKQVELLDKSFNKMKTPENIMLFRGDDPAYLGTEFQNTLLNSNGTINKTAFEKAKAKFLNKDRLEYGYISTSLMNVSQFAGRPIITKFKVAKGSKAGYIDPISAFAGQLEMLLPRHSTYHIDDMRLSSDGKQIIITATMMGTAINPK

>Mes361

MPFVNKQFNYKDPVNGVDIAYIKIPNAGQMQPVKAFKIHNKIWVIPERDTFTNPEEGDLNPPPEAKQVPVSYYDSTYLSTDNEKDNYLKGVTKLFERIYSTDLGRMLLTSIVRGIPFWGGSTIDTELKVIDTNCINVIQPDGSYRSEELNLVIIGPSADIIQFECKSFGHDVLNLTRNGYGSTQYIRFSPDFTFGFEESLEVDTNPLLGAGKFATDPAVTLAHELIHAEHRLYGIAINPNRVFKVNTNAYYEMSGLEVSFEELRTFGGHDAKFIDSLQENEFRLYYYNKFKDVASTLNKAKSIIGTTASLQYMKNVFKEKYLLSEDTSGKFSVDKLKFDKLYKMLTEIYTEDNFVNFFKVINRKTYLNFDKAVFRINIVPDENYTIKDGFNLKGANLSTNFNGQNTEINSRNFTRLKNFTGLFEFYKLLCVRGIIPFKTKSLDEGYNKALNDLCIKVNNWDLFFSPSEDNFTNDLDKVEEITADTNIEAAEENISLDLIQQYYLTFDFDNEPENISIENLSSDIIGQLEPMPNIERFPNGKKYELDKYTMFHYLRAQEFEHGDSRIILTNSAEEALLKPNVAYTFFSSKYVKKINKAVEAFMFLNWAEELVYDFTDETNEVTTMDKIADITIIVPYIGPALNIGNMLSKGEFVEAIIFTGVVAMLEFIPEYALPVFGTFAIVSYIANKVLTVQTINNALSKRNEKWDEVYKYTVTNWLAKVNTQIDLIREKMKKALENQAEATKAIINYQYNQYTEEEKNNINFNIDDLSSKLNESINSAMININKFLDQCSVSYLMNSMIPYAVKRLKDFDASVRDVLLKYIYDNRGTLVLQVDRLKDEVNNTLSADIPFQLSKYVDNKKLLSTFTEYIKNIVNTSILSIVYKKDDLIDLSRYGAKINIGDRVYYDSIDKNQIKLINLESSTIEVILKNAIVYNSMYENFSTSFWIKIPKYFSKINLNNEYTIINCIENNSGWKVSLNYGEIIWTLQDNKQNIQRVVFKYSQMVNISDYINRWIFVTITNNRLTKSKIYINGRLIDQKPISNLGNIHASNKIMFKLDGCRDPRRYIMIKYFNLFDKELNEKEIKDLYDSQSNSGILKDFWGNYLQYDKPYYMLNLFDPNKYVDVNNIGIRGYMYLKGPRGSVVTTNIYLNSTLYEGTKFIIKKYASGNEDNIVRNNDRVYINVVVKNKEYRLATNASQAGVEKILSALEIPDVGNLSQVVVMKSKDDQGIRNKCKMNLQDNNGNDIGFIGFHLYDNIAKLVASNWYNRQVGKASRTFGCSWEFIPVDDGWGESSL

>Mes364

MPITINNFNYSDPVDNKNILYLDTHLNTLANEPEKAFRITGNIWVIPDRFSRNSNPNLNKPPRVTSPKSGYYDPNYLSTDSDKDPFLKEIIKLFKRINSREIGEELIYRLSTDIPFPGNNNTPINTFDFDVDFNSVDVKTRQGNNWVKTGSINPSVIITGPRENIIDPETSTFKLTNNTFAAQEGFGALSIISISPRFMLTYSNATNDVGEGRFSKSEFCMDPILILMHELNHAMHNLYGIAIPNDQTISSVTSNIFYSQYNVKLEYAEIYAFGGPTIDLIPKSARKYFEEKALDYYRSIAKRLNSITTANPSSFNKYIGEYKQKLIRKYRFVVESSGEVTVNRNKFVELYNELTQIFTEFNYAKIYNVQNRKIYLSNVYTPVTANILDDNVYDIQNGFNIPKSNLNVLFMGQNLSRNPALRKVNPENMLYLFTKFCHKAIDGRSLYNKTLDCRELLVKNTDLPFIGDISDVKTDIFLRKDINEETEVIYYPDNVSVDQVILSKNTSEHGQLDLLYPSIDSESEILPGENQVFYDNRTQNVDYLNSYYYLESQKLSDNVEDFTFTRSIEEALDNSAKVYTYFPTLANKVNAGVQGGLFLMWANDVVEDFTTNILRKDTLDKISDVSAIIPYIGPALNISNSVRRGNFTEAFAVTGVTILLEAFPEFTIPALGAFVIYSKVQERNEIIKTIDNCLEQRIKRWKDSYEWMMGTWLSRIITQFNNISYQMYDSLNYQAGAIKAKIDLEYKKYSGSDKENIKSQVENLKNSLDVKISEAMNNINKFIRECSVTYLFKNMLPKVIDELNEFDRNTKAKLINLIDSHNIILVGEVDKLKAKVNNSFQNTIPFNIFSYTNNSLLKDIINEYFNNINDSKILSLQNRKNTLVDTSGYNAEVSEEGDVQLNPIFPFDFKLGSSGEDRGKVIVTQNENIVYNSMYESFSISFWIRINKWVSNLPGYTIIDSVKNNSGWSIGIISNFLVFTLKQNEDSEQSINFSYDISNNAPGYNKWFFVTVTNNMMGNMKIYINGKLIDTIKVKELTGINFSKTITFEINKIPDTGLITSDSDNINMWIRDFYIFAKELDGKDINILFNSLQYTNVVKDYWGNDLRYNKEYYMVNIDYLNRYMYANSRQIVFNTRRNNNDFNEGYKIIIKRIRGNTNDTRVRGGDILYFDMTINNKAYNLFMKNETMYADNHSTEDIYAIGLREQTKDINDNIIFQIQPMNNTYYYASQIFKSNFNGENISGICSIGTYRFRLGGDWYRHNYLVPTVKQGNYASLLESTSTHWGFVPVSE

>Mes367

MPVAINSFNYNDPVNDDTILYMQIPYEEKSKKYYKAFEIMRNVWIIPERNTIGTNPSDFDPPASLKNGSSAYYDPNYLTTDAEKDRYLKTTIKLFKRINSNPAGKVLLQEISYAKPYLGNDHTPIDEFSPVTRTTSVNIKLSTNVESSMLLNLLVLGAGPDIFESCCYPVRKLIDPDVVYDPSNYGFGSINIVTFSPEYEYTFNDISGGHNSSTESFIADPAISLAHELIHALHGLYGARGVTYEETIEVKQAPLMIAEKPIRLEEFLTFGGQDLNIITSAMKEKIYNNLLANYEKIATRLSEVNSAPPEYDINEYKDYFQWKYGLDKNADGSYTVNENKFNEIYKKLYSFTESDLANKFKVKCRNTYFIKYEFLKVPNLLDDDIYTVSEGFNIGNLAVNNRGQSIKLNPKIIDSIPDKGLVEKIVKFCKSVIPRKGTKAPPRLCIRVNNSELFFVASESSYNENDINTPKEIDDTTNLNNNYRNNLDEVILDYNSQTIPQISNRTLNTLVQDNSYVPRYDSNGTSEIEEYDVVDFNVFFYLHAQKVPEGETNISLTSSIDTALLEESKDIFFSSEFIDTINKPVNAALFIDWISKVIRDFTTEATQKSTVDKIADISLIVPYVGLALNIIIEAEKGNFEEAFELLGVGILLEFVPELTIPVILVFTIKSYIDSYENKNKAIKAINNSLIEREAKWKEIYSWIVSNWLTRINTQFNKRKEQMYQALQNQVDAIKTAIEYKYNNYTSDEKNRLESEYNINNIEEELNKKVSLAMKNIERFMTESSISYLMKLINEAKVGKLKKYDNHVKSDLLNYILDHRSILGEQTNELSDLVTSTLNSSIPFELSSYTNDKILIIYFNRLYKKIKDSSILDMRYENNKFIDISGYGSNISINGNVYIYSTNRNQFGIYNSRLSEVNIAQNNDIIYNSRYQNFSISFWVRIPKHYKPMNHNREYTIINCMGNNNSGWKISLRTVRDCEIIWTLQDTSGNKENLIFRYEELNRISNYINKWIFVTITNNRLGNSRIYINGNLIVEKSISNLGDIHVSDNILFKIVGCDDETYVGIRYFKVFNTELDKTEIETLYSNEPDPSILKNYWGNYLLYNKKYYLFNLLRKDKYITLNSGILNINQQRGVTEGSVFLNYKLYEGVEVIIRKNGPIDISNTDNFVRKNDLAYINVVDRGVEYRLYADTKSEKEKIIRTSNLNDSLGQIIVMDSIGNNCTMNFQNNNGSNIGLLGFHSNNLVASSWYYNNIRRNTSSNGCFWSSISKENGWKE

>Mes368

MPVNIKXFNYNDPINNDDIIMMEPFNDPGPGTYYKAFRIIDRIWIVPERFTYGFQPDQFNASTGVFSKDVYEYYDPTYLKTDAEKDKFLKTMIKLFNRINSKPSGQRLLDMIVDAIPYLGNASTPPDKFAANVANVSINKKIIQPGAEDQIKGLMTNLIIFGPGPVLSDNFTDSMIMNGHSPISEGFGARMMIRFCPSCLNVFNNVQENKDTSIFSRRAYFADPALTLMHELIHVLHGLYGIKISNLPITPNTKEFFMQHSDPVQAEELYTFGGHDPSVISPSTDMNIYNKALQNFQDIANRLNIVSSAQGSGIDISLYKQIYKNKYDFVEDPNGKYSVDKDKFDKLYKALMFGFTETNLAGEYGIKTRYSYFSEYLPPIKTEKLLDNTIYTQNEGFNIASKNLKTEFNGQNKAVNKEAYEEISLEHLVIYRIAMCKPVMYKNTGKSEQCIIVNNEDLFFIANKDSFSKDLAKAETIAYNTQNNTIENNFSIDQLILDNDLSSGIDLPNENTEPFTNFDDIDIPVYIKQSALKKIFVDGDSLFEYLHAQTFPSNIENLQLTNSLNDALRNNNKVYTFFSTNLVEKANTVVGASLFVNWVKGVIDDFTSESTQKSTIDKVSDVSIIIPYIGPALNVGNETAKENFKNAFEIGGAAILMEFIPELIVPIVGFFTLESYVGNKGHIIMTISNALKKRDQKWTDMYGLIVSQWLSTVNTQFYTIKERMYNALNNQSQAIEKIIEDQYNRYSEEDKMNINIDFNDIDFKLNQSINLAINNIDDFINQCSISYLMNRMIPLAVKKLKDFDDNLKRDLLEYIDTNELYLLDEVNILKSKVNRHLKDSIPFDLSLYTKDTILIQVFNNYISNISSNAILSLSYRGGRLIDSSGYGATMNVGSDVIFNDIGNGQFKLNNSENSNITAHQSKFVVYDSMFDNFSINFWVRTPKYNNNDIQTYLQNEYTIISCIKNDSGWKVSIKGNRIIWTLIDVNAKSKSIFFEYSIKDNISDYINKWFSITITNDRLGNANIYINGSLKKSEKILNLDRINSSNDIDFKLINCTDTTKFVWIKDFNIFGRELNATEVSSLYWIQSSTNTLKDFWGNPLRYDTQYYLFNQGMQNIYIKYFSKASMGETAPRTNFNNAAINYQNLYLGLRFIIKKASNSRNINNDNIVREGDYIYLNIDNISDESYRVYVLVNSKEIQTQLFLAPINDDPTFYDVLQIKKYYEKTTYNCQILCEKDTKTFGLFGIGKFVKDYGYVWDTYDNYFCISQWYLRRISENINKLRLGCNWQFIPVDEGWTE

>Mes378

MSSERTFLPNGNYKIKSLFSDSLYLTYSSGALSFSNTSSLDNQKWKLEYISSSNGFRFSNVAEPNKYLAYNDYGFIYLSSSSNNSLWNPIKIAINSYIICTLSIVNVTDYAWTIYDNNNNITDQPILNLPNFDINNSNQILKLEKL

>Mes379

MSQTNANDLRNNEVFFISPSNNTNKVLDKISQSEVKLWNKLSGANQKWRLIYDTNKQAYKIKVMDNTSLILTWNAPLSSVSVKTDTNGDNQYWYLLQNYISRNVIIRNYMNPNLVLQYNIDDTLMVSTQTSSSNQFFKFSNCIYEALNNRNCKLQTQLNSDRFLSKNLNSQIIVLWQWFDSSRQKWIIEYNETKSAYTLKCQENNRYLTWIQNSNNYVETYQSTDSLIQYWNINYLDNDASKYILYNLQDTNRVLDVYNSQIANGTHVIVDSYHGNTNQQWIINLI

>Mes380

MSLSIKELYYTKDKSINNVNLADGNYVVNRGDGWILSRQNQNLGGNISNNGCTAIVGDLRIRETATPYYYPTASFNEEYIKNNVQNVFANFTEASEIPIGFEFSKTAPSNKSLYMYLQYTYIRYEIIKVLQNTVTERAVLYVPSLGYVKSIEFNSEEQIDKNFYFTSQDKCILNEKFIYKKIDDTITVKESKNSNNNINFNTSQTILPYPNGLYVINKGDGYMRTNDKDLIGTLLIESSTSGSIIQPRLRNTTRPLFNTSNPTIFSQEYTEARLNDAFNIQLFNTSTTLFKFVEEAPTNKNISMKVYNTYEKYELINYQNGNIDDKAEYYLPSLGKCEVSDAPSPQAPVVETPVDQDGFIQTGPNENIIVGVINPSENIEEISTPIPDDYTYNIPTSIQNNACYVLFKVNTTGVYKITTKNNLPPLIIYEAIGSSNRNMNSNNLSNDNIKAIKYITGLNRSDAKSYLIVSLFKDKNYYIRIPQISSSTTSQLIFKRELGNISDLADSTVNILDNLNTSGTHYYTRQSPDVGNYISYQLTIPGDFNNIASSIFSFRTRNNQGIGTLYRLTESINGYNLITINNYSDLLNNVEPISLLNGATYIFRVKVTELNNYNIIFDAYRNS

>Mes381

MIANSSTDVSVADQKFLNVAKSNQIDPDAVPISRLDSEGHSIFAEWRPKRPFLRREDGIFLVLRADHIFLLGTDPRTRQIETELMLNRGVKAGAVFDFIDHSMLFSNGETHGKRRSGLSKAFSFRMVEALRPEIAKITECLWDDLQKVDDFNFTEMYASQLPALTIASVLGLPSEDTPFFTRLVYKVSRCLSPSWRDEEFEEIEASAIELQDYVRSVIADSGRRMRDDFLSRYLKAVREAGTLSPIEEIMQLMLIILAGSDTTRTAMVMVTALALQNPALWSSLRGNQSYVAAAVEEGLRFEPPVGSFPRLALKDIDLDGYVLPKGSLLALSVMSGLRDEKHYEHPQLFDVGRQQMRWHLGFGAGVHRCLGETLARIELQEGLRTLLRRAPNLAVVGDWPRMMGHGGIRRATDMMVKLSFDL

>Mes382

MEERRVSISSITWRFPMLFAPVDDVTTIDDLTLDPYPIYRRMRVQNPVVHVASVRRTFLTKAFDTKMVKDDPSRFSSDDPSTPMKPAFQAHTLMRKDGTEHARERMAMARAFAPKAIADHWAPIYRDIVNEYLDRLPRGDTVDLFAEICGPVAARILAHILGICEASDVEIIRWSQRLIDGAGNFGWRSELFERSDEANAEMNCLFNDLVKKHRSAPNPSAFATMLNAPDPIPLSQIYANIKIAIGGGVNEPRDALGTILYGLLTNPEQLEEVKRQQCWGQAFEEGLRWVAPIQASSRLVREDTEIRGFIVPKGDIVMTIQASANRDEDVFEDGESFNVFRPKSAHQSFGSGPHHCPGAQISRQTVGAIMLPILFDRFPDMILPHPELVQWRGFGFRGPINLPVTLR

>Mes384

MTRQMILAVGQQGPIARAETREQVVGRLLDMLTNAASRGVNFIVFPELALTTFFPRWHFTDEAELDSFYETEMPGPVVRPLFETAAELGIGFNLGYAELVVEGGVKRRFNTSILVDKSGKIVGKYRKIHLPGHKEYEAYRPFQHLEKRYFEPGDLGFPVYDVDAAKMGMFICNDRRWPETWRVMGLKGAEIICGGYNTPTHNPPVPQHDHLTSFHHLLSMQAGSYQNGAWSAAAGKVGMEEGCMLLGHSCIVAPTGEIVALTTTLEDEVITAAVDLDRCRELREHIFNFKAHRQPQHYGLIAEF

>Mes385

MAKADFYETLGVSKTADEKELKSAFRKLAMKYHPDKNPDDADSERKFKEINEAYETLKDPQKRAAYDRFGHAAFENGGMGGGGGGFGGGGFANGGFSDIFEDIFGEMMGGGRARRSSGGRERGADLRYNMEITLEEAFTGKTAQIRVPTSITCDVCSGSGAKPGTQPKTCATCQGSGRVRAAQGFFSVERTCPTCHGRGQTISDPCGKCHGQGRVTEERSLSVNIPSGIEDGTRIRLQGEGEAGMRGGPAGDLYIFLSVRPHEFFQRDGADLYCTVPISMTTAALGGTFDVTTLDGTKSRVTVPEGTQPGKQFRLKGKGMPVLRSAQTGDLYIQIQIETPQKLSKRQRELLQEFEQLSSKENNPESTGFFARMKKFFDG

>Mes386

MNVLSVSSEIYPLIKTGGLADVVGALPIALEAHGVRTRTLIPGYPAVKAAVTDPVKCFEFTDLLGEKADLLEVQHERLDLLILDAPAYYERSGGPYLGQTGKDYPDNWKRFAALSLAAARIGAGVLPGWRPDMVHAHDWQAAMTPVYMRYAETPEIPSLLTIHNIAFQGQFGANIFSKLALPAHAFGMEGIEYYNDVSFLKGGLQTATALSTVSPSYAEEILTAEFGMGLEGVIGSRAHVLHGIVNGIDADVWNPATDHLIHDNYSAANLKNRALNKKAVAEHFRIDDDGSPLFCVISRLTWQKGIDLMAEAVDEIVSLGGRLVVLGAGDVALEGALLAAASRHHGRVGVAIGYNEPLSHLMQAGCDAIIIPSRFEPCGLTQLYALRYGCIPVVARTGGLADTVIDANHAALASKAATGVQFSPVTLDGLKQAIRRTVRYYHDPKLWTQMQKLGMKSDVSWEKSAGLYAALYSQLISKGH

>Mes387

MSEKRVQPLARDAMAYVLAGGRGSRLKELTDRRAKPAVYFGGKARIIDFALSNALNSGIRRIGVATQYKAHSLIRHLQRGWDFFRPERNESFDILPASQRVSETQWYEGTADAVYQNIDIIEPYAPEYMVILAGDHIYKMDYEYMLQQHVDSGADVTIGCLEVPRMEATGFGVMHVNEKDEIIDFIEKPADPPGIPGNEGFALASMGIYVFHTKFLMEAVRRDAADPTSSRDFGKDIIPYIVEHGKAVAHRFADSCVRSDFEHEPYWRDVGTIDAYWQANIDLTDVVPDLDIYDKSWPIWTYAEITPPAKFVHDDEDRRGSAVSSVVSGDCIISGAALNRSLLFTGVRANSYSRLENAVVLPSVKIGRHAQLSNVVIDHGVVIPEGLIVGEDPELDAKRFRRTESGICLITQSMIDKLDL

>Mes388

MDYSRLLKRSVSAALTAAALLCSTAAFAGEVTIWCWDPNFNVAIMKEAAERYTAKHPDTTFNIVDFAKADVEQKLQTGLASGMTDTLPDIVLIEDYGAQKYLQSFPGSFAALTDKIDFSGFAKYKVDLMTLEGQVYGVPFDSGVTGLYYRTDYLEQAGFKPEDMQNLTWDRFIEIGKEVKAKTGHEMMALDANDGGLIRIMMQSGGQWYFNEDGSLNITGNAALKAALETQARIVNERVAKPTSGSNDGIRALTSGDVASVLRGVWITGTVKSQPDQAGKWALTAIPKLNIEGATAASNLGGSSWYVLEASAEKDEAIDFLNEIYAKDLDFYQKILTERGAVGSLLAARTGEAYQKPDDFFGGQTVWQNFADWLVQVPAVNYGIFTNELDTAVTANFPALVKGTPVDEVLKAIEDQAAGQIQ

>Mes389

MATTSRSSLKRYYDVNGWLFVAPAIALISVFMLYPILRSLVLSLYTGRGMMLKFSGTGNLVRLWNDPVFWQALQNTVIFFVVQVPIMITMALILAAMLNNPKLRYSGLFRTMIFLPCVSSLVAYSILFKSMFSLDGVVNNTLLAIGIIGEPIGWLTDPFWAKVLIIIAITWRWTGYNMIFYLAALQNIDRSIYEAAKIDGVPSWGRFAFLTIPMLKPVILFTTITSTIGTLQLFDEVYNFTEGTGGPANSTLTLSLYIYNLTFRFMPSFSYAATVSYVIVLMVAVLSFLQFYAARERK

>Mes390

MMTTLRRRLPDIVQYSVLSLAAFLSIFPFIWMVIGTTNTTSQIIRGKVTFGTALFDNIASFFAQVDVPLVFWNSVKIALVGTALTLLVSSLAGYGFEMFRSKLRERVYTVILLTLMVPFAALMIPLFMLMGQAGLLNTHIAIMLPMIASAFIIFYFRQASKAFPTELRDAAKVDGLKEWQIFFYIYVPVMRSTYAAAFVIVFMLNWNNYLWPLIVLQSNDTKTITLVVSSLASAYSPEYGTVMIGTILATLPTLLVFFAMQRQFVQGMLGSVK

>Mes391

MTLFQVYTRALRYLTVHKWRVAVVVIANVILAAITIAEPVLFGRIIDAISSGTNVTPILILWAGFGVFNTVAYVAVAREADRLAHGRRATLLTEAFGRIISMPLSWHHLRGTSNALHTLLRASETLFGLWLEFMRTHLATFVALVLLIPTAMAMDLRLSFVLIGLGIVYWFIGKWVMGRTKDGQASVEEHYHSVFAHVSDSISNVSVLHSYNRIEAETKALKSFTEKLLSAQYPVLDWWAFASALNRTASTVSMMIILVIGTVLVKNGELRVGDVIAFIGFANLLIGRLDQMRQFVTQIFEARAKLEDFFVLEDAVKEREEPGDARELSNVSGTVEFRNINFGFANTKQGVHDVSFTAKAGETVAIVGPTGAGKTTLINLLQRVYDPDSGQILIDGTDISTVTKNSLRNSIATVFQDAGLLNRSIRENIRLGRETATDAEVVEAAAAAAATDFIDSRINGYLTQVGERGNRLSGGERQRIAIARAILKNAPILVLDEATSALDVETEARVKAAVDALRKNRTTFIIAHRLSTVRDADLVLFLDQGRIIEKGTFDELTQRGGRFTSLLRTSGLLTEDEGQQPRPKAIAS

>Mes392

MPFDPAFLWQTFVALLSGIPLALQLAVFSVALGTVLAFGLALMRVSRLWWLDLPARFYIFAFRGTPLLVQIYIIYYGLSQFPDVRHSFIWPFLRDAYWCAMAALALNTAAYTAEIMRGGLLSVPAGQIEAAKACGMGRVKLFRRIVIPQAIRQMLPGYSNEVILMVKSTSLASTITIMEITGIAAKLISESYRTVEVFSCAGAIYLILNFIVARLFTLLEWALWPERRNNRLTTDPVDRKGELHA

>Mes393

MPNPVRPAVQLKDIRKNFGNLEVLHGVSLSANEGEVISILGSSGSGKSTLLRCVNMLEVPNAGSVAIMGEEIALEHRAGRLARPKDLKQVNRLRERAAMVFQGFNLWSHQTILQNVMEAPVHVQGRDRKACRDEAEALLERVGIASKRDAYPSELSGGQQQRAAIARALAMRPDVMLFDEPTSALDPELVGEVLKVMRDLAAEGRTMLIVTHEMDFARDVSSRTVFLHQGVIAEEGPSSEMFAHPRTDRFRQFLRRDGGTSH

>Mes394

MDYSQLMGFGPDGWGYDMLRATAMTMAVAFSGFTIGLVFGCLGAAASLSSSGALQAAASGYTTALRGIPDLLVIYLFYFGSSSVISNVASLFGSSGFVGASTFLIGALAIGVVSGAYQTQVLRGAVLALNKGEIEAGRAYGMGALLLFRRIVLPQAARYALPGVGNVWQLVLKESALISVIGLVELMRQAQVGSGSTRQPFSFYLTAAALYLLITFVSGQVFRLAETRSMRGLQRGV

>Mes395

MKLKTILCAALLLVAGQAAAQEKSITIATEGGYAPWNFSGPGGKLDGFEIDLANALCEKMKAKCQIVAQNWDGIMPSLTGKKYDAIMAAMSVTPKRQEVIGFSIPYAAGINGFAVMGDSKLAEMPGLGETYSLDSQADAAKKAIADISSFLNGTTVGVQGSTTASTFLDKYFKGSVDIKEYKSVEEHNLDLTSGRLDAVLANATVLAAAIEKPEMKGAKLVGPLFSGGEFGVVAVGLRKEDTALKADFDAAIKAASEDGTIKTLSLKWFKVDVTPQ

>Mes396

MALATRATGGAGRRKPVRARCARGLHLVSCHKTQLLGFTIRNAASWTIHPQGCEDLTAAASTIIAPHDSPNTDGFNPESCRNVMISGVRFSVGDDCIAVKAGKRGPDGEDDHLAETRGITVRHCLMQPGHGGLVIGSEMSGGVHDVTVEDCDMIGTDRGLRLKTGARSGGGMVGNITMRRVLLDGVQTALSANAHYHCDADGHDDWVQSRNPAPVNDGTPFVDGITVEDVEIRNLAHAAGVFLGLPDVPSATSLSATSPIVSHDPSAVATPPIMADRVRPMRMRLVFEQADVVCDDPALLNDAPVSISSYFD

>Mes397

MIKTIKTTPYQDQKPGTSGLRKKVPVFAQENYAENFIQSIFDALEGFEGQTLVIGGDGRYYNREVIQKAIKMAAAAGFGKVLVGQGGILSTPAASNVIRKYKAFGGIVLSASHNPGGPTEDFGIKYNIGNGGPAPEKITDAIYARSKVIDSYKISDAADIDLDKIGSFKVDELTVDVIDPVADYAALMEELFDFGAIRSLIAGGFKVVVDSMSAVTGPYAVEILEKRLGAPKGSVRNATPLPDFGGHHPDPNLVHAKELYDDVMSPEGPDFGAASDGDGDRNMVVGKGMFVTPSDSLAIIAANAKLAPGYAAGISGIARSMPTSAAADRVAEKLGLGMYETPTGWKFFGNLMDAGKVTICGEESFGTGSNHVREKDGLWAVLYWLNIVAARKESVKDIVTKHWAEYGRNYYSRHDYEEVDSDAANTLVAILREKLATLPGTSYGNLKVAAADDFAYHDPVDQSVSKNQGIRILFEGGSRIVLRLSGTGTAGATLRLYVERYEPDAARHGIETQSALADLISVADTIAGIKAHTADSEPTVIT

>Mes398

MGLPFQIEYGQTTGRPELIEDALRQFSAALALTADAGGLYVHGYDESRNQRWANPASGKSPAIWARAVGWLAMALVDALVILPDDSATAELRERTRRLLAGIIARQTQAGLWMQVLDNQGLAGNYAETSASAMFAYALLRAARLGLLRGEEAKAALSAGRQALAALLETRLELDEQGVARLTGIVHVAGLGGFDGNYRDGTPDYYLTEPVVSDDAKGVGPLMMAYAESLLLAR

>Mes399

MTETAYGNAQDLLVELTADIVAAYVSNHVVPVTELPGLISDVHTALSGTSAPASVAVNVEKQKPAVSVRKSVQDDHIVCLECGGSFKSLKRHLTTHHSMTPEEYREKWDLPVDYPMVAPAYAEARSRLAKEMGLGQRRKANR

>Mes400

MSGTILITGATSGFGQATAQRFVKEGWKVIGTGRRAERLEALSAELGSAFHGVAFDITDEEATKKALAGLPDGFRDIDILVNNAGLALGTAPAPQVPLKDWQTMVDTNITGLLNVTHHLLPTLIERKGIVINLSSVAAHYPYLGGNVYGGTKAFLRQFSLGLRSDLHGKGVRVTSIEPGMCETEFTLVRTGGNQEASDNLYKGVNPITADDIANTIHWVASQPKHININSLELMPVNQSFAGFQVYRES

>Mes401

MELEDANVTKKVELRPLIGLTRGLPPTDLETITIDAIRTHRRLVEKADELFQALPETYKTGQACGGPQHIRYIEASIEMHAQMSALNTLYSILGFIPKVVVN

>Mes402

MQHWLDKLTDLAAIEGDECILKTGLADIADHFGFTGYAYLHIQHRHITAVTNYHRQWQSTYFDKKFEALDPVVKRARSRKHIFTWSGEHERPTLSKDERAFYDHASDFGIRSGITIPIKTANGFMSMFTMASDKPVIDLDREIDAVAAAATIGQIHARISFLRTTPTAEDAAWLDPKEATYLRWIAVGKTMEEIADVEGVKYNSVRVKLREAMKRFDVRSKAHLTALAIRRKLI

>Mes403

MKYCLLCLVVALSGCQTNDTIASCKGPIFPLNVGRWQPTPSDLQLRNSGGRYDGA

>Mes404

MTRKALFILACLFAAATGAEAEDTPMAGKLDPRMRYLAYNPDQVVRLSTAVGATLVVTFATNETVTSVAVSNSKDLAALPRGNYLFFKASQVLTPQPVIVLTASDSGMRRYVFSISSKTLSHLDKEQPDLYYSVQFAYPADDAAARRREAQQRAVVDRLHAEAQYQRKAEDLLDQPVTALGATDSNWHYVAQGDRSLLPLEVFDNGFTTVFHFPGNVRIPSIYTINPDGKEAVANYSVKGSDVEISSVSRGWRLRDGHTVLCIWNAAYDPVGQRPQTGTVRPDVKRVLKGAKG

>Mes405

MAIIKPHANKNRTTSPIERPESLIEEMSGSNPPIGFTSLDLAMIELEDFVHRCPLPGDNLAGQKE

>Mes406

MSEKIYDVPAEWAKRAWVDQAKYKEMYARSISDPNGFWAEQAKRIDWMKAPTKIENVSFAPGNVSIKWFEDGVLNVAHNCIDRHLHKRANQTAIIWEGDDPSQSRHITYKELHDEVCRMANILRTRNVKKGDRVTIYLPMIPEAAYAMLACARIGAIHSVVFAGFSPDSLAQRINDCQSKVIITADEGLRGGKKVPLKANVDAALAKADGVDWVVVVKRTGGKIDMNPTRDLWYHEAAAMVTTECPVEHMHAEDPLFILYTSGSTGQPKGVLHTSAGYLVYAAMTHQYVFDYHDGDIYWCTADVGWVTGHSYILYGPLANGATTLMFEGVPNYPDNSRFWNVIDKHKVNTFYTAPTAIRALMQGGDEPVKKTSRASLRLLGSVGEPINPEAWEWYHRVVGEDRCPIVDTWWQTETGGILITPLPGATKLKPGSATQPFFGVVPEIVDADGKVLEGETTGNLCLTRAWPGMMRTVYGDHARFEQTYFSTYKGKYFTGDGCRRDADGYYWITGRVDDVINVSGHRMGTAEVESALVAHEKVSEAAVVGFPHDIKGQGIYAYVTLMAGVQPTEDLRKELVTWVRKEIGPIASPDQIQFAPGLPKTRSGKIMRRILRKIAEDEPGSLGDTSTLADPAVVDDLVKNRQNKKSA

>Mes407

MKRTMIVVTTLLLGAGAVMAQQEVAVQQDNLMRSQARSLYTVILKMTKGDIPYDQKAADEAIANLETDVAKIAKTFEVNPKQDVVNATYGASPKVWKNKADFDSKIPPVQKAIAQVKGKITDVASLKAAYTAINDRCTDCHETYRLKLK

>Mes408

MHFRPLHDRVLVRRIDAEEKTAGGIIIPDTAKEKPQEGEIIAAGSGGRNEQGQLIPIDVKPGDRVLFGKWSGTEVKIDGQDYLIMKESDLLGVVDKTGSVKKAA

>Mes412

MSAKEVKFGVDARDRMLRGVDILHNAVKVTLGPKGRNVVLDKSFGAPRITKDGVTVAKEIELEDKFENMGAQMVREVASKSADAAGDGTTTATVLAAAIVREGAKSVAAGMNPMDLKRGIDMAVEAVVADLVKNSKKVTSNEEIAQVGTISANGDAEIGKFISDAMKKVGNEGVITVEEAKSLETELEVVEGMQFDRGYISPYFVTNADKMRVEMDDAYVLINEKKLSQLNELLPLLEAVVQSGKPLVIIAEDVEGEALATLVVNRLRGGLKVAAVKAPGFGDRRKAMLQDIAILTGGQAISEDLGIKLENVTLNMLGRAKKVMIDKENTTIVSGAGKKADIEARVAQIKAQIEETTSDYDREKLQERLAKLAGGVAVIRVGGATEVEVKERKDRVDDAMHATRAAVEEGILPGGGVALLRASEHLKGIRTKNDDQKTGVEIVRKALSYPARQIAINAGEDGSVIVGKILEKDQYSYGYDSQTGEYGNLVSKGIIDPTKVVRVAIQNAASVAALLITTEAMVAEVPKKNTGAGGMPPGGGGMGGMGGMDF

>Mes415

MAVALILLLIAIGSVLFHLFSPWWWTPIATNWGYIDDTINITFWITGFVFTAVILFMAYCVFRFHHKEGRQAAYNPENKKLEWWLSVGTGVGVAAMLAPGLVVWHQFVTVPADATEVEIMGQQWQWSFRLPGKDGRLGTSDVRNISPENPMGLNRDDPHGQDDVVIENGDLHLPIGKPVKVLLRSVDVLHDFYVPEFRAKMDMVPGMVTYFWIRPIRTGTFDVLCAELCGAAHYQMRAKVIVEAESDYHAWLEQQKTFAGLSGRNAVVRAKYNSGDD

>Mes416

MVDVPYDRIADIPPAEVPDVELYHPRSWWTRYVFSQDAKVIAIQYSLTASAIGLVALVLSWLMRLQLGFPGTFSFIDANQYLQFITMHGMIMVIYLLTALFLGGFGNYLIPLMVGARDMVFPYVNMLSYWVYLLAVLVLASAFFVPGGPTGAGWTLYPPQAILSGTPGQDWGIVLMLSSLILFIIGFTMGGLNYVVTVLQARTRGMTLMRLPLTVWGIFTATVMALLAFPALFVGSVMLLLDRLLGTSFFMPTLVEMGQLSKYGGGSPLLFQHLFWFFGHPEVYIVALPAFGIVSDLISTHARKNIFGYRMMVWAIVAIGALSFVVWAHHMYVSGMYPYFGFFFATTTLIIAIPTAIKVYNWVLTLWHGDIHLTVPMLFALGFIITFVNGGLTGLFLGNVVVDVPLSDTMFVVAHFHMVMGVAPIMVVLGAIYHWYPKVTGRMLNDVLGKFHFWVTFLGAYLIFFPMHYLGLLGVPRRYFELGDAAFIPPSAHSLNAFITVVALTVGFAQMVFLFNLVWSLFEGEPSGGNPWRATTLEWQTPETPPGHGNWGKQLPIVYRWAYDYSVPGAAQDFIPQNQPPPTGAVQGVAP

>Mes417

MTKLTFGALVALAMTAAASTAMSSKAMAQDAAAGKTSFNKCLACHAIGEGAKNKVGPELNGLNGRKSGTAPDYSYSDANKNSGITWDEATFKEYIKDPKAKIPGTKMAFAGIKNETEINNLWTFVSQFDKDGKIKQ

>Mes418

MHLHLRGICLVLAVASSSSSALAADAGHGADLAKRWCASCHVVANGQAVASADVPSFASVARRPDFSSEKLAFFLLDPHPKMPSFPLSRTEAGDIAAYIGSLRP

>Mes419

MSGPSDYQPSNPALQWIERRLPILGLMHSSFVAYPTPRNLNYWWTFGAILSFMLGMQILTGVILAMHYTPHADLAFKSVELIVRDVNYGWLLRNMHACGASMFFFAVYVHMLRGLYYGSYKEPREVLWILGVIIYLLMMATGFMGYVLPWGQMSFWGATVITNLFSAIPYFGESIVTLLWGGYSVGNPTLNRFFSLHYLLPFLIAGVVVLHVWALHVAGQNNPEGVEPKSEKDTVPFTPHATIKDMFGVACFLLLYAWFIFYMPNYLGDADNYIPANPGVTPPHIVPEWYYLPFYAILRSIPNKLAGVIGMFSAIIILCFLPWLDAAKTRSSKYRPLAKQFFWIFVAVCILLGYLGAQPPEGIYVIAGRVLTVCYFAYFLIVLPLLSRIETPRPVPNSISEAILAKGGKAVASVAIALVAAGALFLGSLQDARANEGSDKPPGNKWSFAGPFGKFDRGALQRGLKVYKEVCASCHGLSYIAFRNLAEAGGPSYSVAQVAAFASDYKIKDGPNDAGDMFERPGRPADYFPSPFPNEQAARAANGGAAPPDLSLITKARSYGRGFPWFIFDFFTQYQEQGPDYVSAVLQGFEEKVPEGVTIPEGSYYNKYFPGHAIKMPKPLSDGQVTYDDGSPATVAQYSKDVTTFLMWTAEPHMEARKRLGFQVFVFLIIFAGLMYFTKKKVWADSH

>Mes420

MSEQSTSANPQRRTFLMVLPLIAFIGLALLFWFRLGSGDPSRIPSALIGRPAPQTALPPLEGLQADNVQVPGLDPAAFKGKVSLVNVWASWCVPCHDEAPLLTELGKDKRFQLVGINYKDAADNARRFLGRYGNPFGRVGVDANGRASIEWGVYGVPETFVVGREGTIVYKLVGPITPDNLRSVLLPQMEKALK

>Mes423

MAPTRVTHPPDDGRGEHFRVRIEGFGVGTWDLDLKTWALDWSDTARTLLGIGQDQPASYDLFLSRLEPDDRERVESAIKRVSERGGGFDVSFRVAGTSNAGQWIRARAGLIRDEAGTARHLSGIFLDIDEEKQVEGALRTRETHLRSILHTIPDAMIVIDGHGIIQLFSTAAERLFGWSELEAIGQNVNILMPEPDRSRHDSYISRYRTTSDPHIIGIGRIVTGKRRDGTTFPMHLSIGEMQSGGEPYFTGFVRDLTEHQQTQARLQELQSELVHVSRLSAMGEMASALAHELNQPLAAISNYMKGSRRLLAGSSDPNTPKVESALDRAAEQALRAGQIIRRLRDFVARGESEKRVESLSKLIEEAGALGLAGAREQNVQLRFSLDPGADLVLADRVQIQQVLVNLFRNALEAMAQSQRRELVVTNTPAADDMIEVEVSDTGSGFQDDVIPNLFQTFFTTKDTGMGVGLSISRSIIEAHGGRMWAESNASGGATFRFTLPAADEN

>Mes424

MSQPSISKSMTIGESGLAVVFAATAFLCVIAAAKALDAPFAFHAALSAAASVAAVFCIVNRYFERPAALPPAEINGRPNYNMGPIKFSSFMAMFWGIAGFLVGLIIASQLAWPALNFDLPWISFGRLRPLHTSAVIFAFGGNVLIATSFYVVQKSCRVRLAGDLAPWFVVVGYNFFILVAGTGYLLGVTQSKEYAEPEWYADLWLTIVWVVYLLVFLATIIKRKEPHIFVANWFYLAFIVTIAVLHLGNNPALPVSAFGSKSYVAWGGIQDAMFQWWYGHNAVGFFLTAGFLAIMYYFIPKRAERPIYSYRLSIIHFWALIFLYIWAGPHHLHYTALPDWTQTLGMTFSIMLWMPSWGGMINGLMTLSGAWDKLRTDPVLRMLVVSVAFYGMSTFEGPMMSIKVVNSLSHYTDWTIGHVHSGALGWVGFVSFGALYCLVPWAWNRKGLYSLKLVNWHFWVATLGIVLYISAMWVSGILQGLMWRAYTSLGFLEYSFIETVEAMHPFYIIRAAGGGLFLIGALIMAYNLWMTVRVGEAEVQMPVALQPAE

>Mes425

MLHIPSSSERPASQPEPERAPPGEPSHESALAGIYEISKILNAPGRLEVTLANVLGLLQSFVQMRHGLVSLFNDDGVPELTVGAGWSEGTDERYRTCVPQKAIHEIVATGRSLMVENVAAETAFSAADREVLGASDSIPVAFIGVPIRVDSTVVGTLTIDRIPEGSSSLLEYDARLLAMVANVIGQTIKLHRLFAGDREQSLVDKDRLEKQTVDRGPPARERKQLQAHGIIGDSPALSALLEKIVVVARSNSTVLLRGESGTGKELVAKAIHESSVRAKRPFVKLNCAALPETVLESELFGHEKGAFTGAVSARKGRFELADKGTLFLDEIGEISPPFQAKLLRVLQEQEFERVGSNHTIKVDVRVIAATNRNLEEAVARSEFRADLYYRISVVPLLLPPLRERRSDIPLLAREFLRKFNSENGRSLTLEASAIDVLMSCKFPGNVRELENCIERTATLSAGTSIVRSDFACSQGQCLSTTLWKSTSYGKTDPAAPMQPVPAKSIIPLAETAPPPQAVCEPGSLAPSGTVLVSGARMADRERVVAAMEKSGWVQAKAARLLGLTPRQVGYALRKYGIEIKRF

>Mes426

MNRATPRRRRWRIGELAEATGVTVRTLHHYEHTGLLAATERTEGGHRMYDRESGQRVHQIRALRELGFSLVEIRKAMEGTTSLTDLLRKHLERIEVQVARTTLLRDRLRNMTIDSEAQVSVDELPATLNAMSRAETRSQTSRCTCNLAAEREDRWRRIRDDLRDCMDGGEHPCGERAKAVAVAARLLISEIAGDDSRVSMILKVLARLSAPRSLAGWDPCLMQYLDLALGGLEDQPY

>Mes427

MNAHTGTVRGKERYRSGVMEYKRMGYWEPDYTPKDTDVIALFRVTPQEGVDPIEASAAVAGESSTATWTVVWTDRLTAAEKYRAKCHRVDPVPGTPGSYFAYIAYDLDLFEPGSIANLSASIIGNVFGFKPLKALRLEDMRFPVAYVKTFQGPATGIVVERERLDKFGRPLLGATVKPKLGLSGRNYGRVVYEALKGGLDFTKDDENINSQPFMHWRDRFLYCIEAVNRAQAASGEVKGTYLNITAGTMEDMYERAEFAKELGSCIVMIDLVIGYTAIQSMAKWARRNDMILHLHRAGHSTYTRQKSHGVSFRVIAKWMRLAGVDHIHAGTVVGKLEGDPNTTRGYYDVCREDFNPTKLEHGLFFDQSWASLNKMMPVASGGIHAGQMHQLLDLLGEDVVLQFGGGTIGHPMGIAAGAIANRVALEAMILARNEGRDYVHEGPEILAKAAQTCTPLKSALEVWKDVTFNYQSTDTPDFVPTALETV

>Mes429

MALTQRLEFRQSQSLVMTPQLMQAIKLLQLSNLDLTTFVEEELERNPLLERANDEASGGEAPAEAGQFSDSDGGHNDEPGGGPGEAFEPGQEEWMSKDLGTRAEIEQTLDTGLDNVFSEEPAEAAARNAQDAAPTTYTEWGGGASGDEDYNLEAFVAAEVTLGDHLAEQLSVAFTAPAQRMIGQYLIDLVDEAGYLPPDLGQAAERLGASQQEVEDVLAVLQKFDPPGVCARNLSECLAIQLRELDRYDPAMQALVEHLDLLAKRDIAGLRKVCGVDDEDIADMIGEIRRLNPKPGMKFGAARLQTMVPDVYVRPGPDGGWHVELNSDTLPRVLVNQTYYSELSKKIGKDGDKSYFTDALQNATWLVRALDQRARTILKVATEIVRQQDGFFTHGVAHLRPLNLKAVADAIQMHESTVSRVTANKYMATNRGTFELKYFFTASIASADGGEAHSAEAVRHHIKQLIDSEAPAAILSDDTIVERLRASGIDIARRTVAKYREAMRIPSSVQRRRDKQSALGNVLSTAMSDRSRNPEPA

>Mes430

MLDTKPSATRRIPLVIATVAVGGLAGFAALYGLGLSRAPTGDPACRAAVATAQKIAPLAHGEVAALTMASAPLKLPDLAFEDADGKPKKLSDFRGKTLLVNLWATWCVPCRKEMPALDELQGKLSGPNFEVVAINIDTRDPEKPKTFLKEANLTRLGYFNDQKAKVFQDLKAIGRALGMPTSVLVDPQGCEIATIAGPAEWASEDALKLIRAATGKAAAAL

>Mes432

MKIRSTALVKGFRQSTPYVNAHRGKTMVIMLGGEAVAHNNFGNIINDIALMHSLGIKVVVVYGARPQINQLLEKQDLTTPYHKNIRITDEAALSVVMQAAGQLQLAITARLSMSLNNTPMAGTQLNVVSGNFVIAQPLGVDDGVDYCHSGRIRRIDTDAINRTLDQGSIVLLGPIASSVTGECFNLLSEEVATQLAIKLGADKLIGFCSEQGVIDDNGNAVAELLPIEAEHVIKTLSENHASDSDYNTGTLRFLKGSIAACRAGVPRSHLISYKVDGALIQELFSFDGIGTQVVMASAEQVRQAGIDDIGGILELIHPLEEQGILVRRSREQLEQEIGKFTIIEKDGLIIGCAALYPYSEERKAEMACVAIHPDYRDGNRGLLLLNYMKHRSKSENINQIFVLTTHSLHWFREQGFYEVGVDYLPGAKQGLYNFQRKSKILALDL

>Mes433

MTKSLFRQSFLTDTLDVHIDVAPAEQVLSNGVQLKLYQRGVLEVIPENPTQETKNIIISCGIHGDETAPMELVDSIIKDIESGFQKVDARCLFIIAHPESTLAHTRFLEENLNRLFDEKEHEPTKELAIADTLKLLVRDFYQDTEPKTRWHLDLHCAIRGSKHYTFAVSPKTRHPVRSKALVDFLDSAHIEAVLLSNSPSSTFSWYSAENYSAQALTMELGRVARIGENALDRLTAFDLALRNLIAEAQPEHLSKPCIKYRVSRTIVRLHDDFDFMFDDNVENFTSFVHGEVFGHDGDKPLMAKNDNEAIVFPNRHVAIGQRAALMVCEVKTRFEEGELVYD

>Mes434

MSHIRFFPRHRLALACMLASVSSFSFAQNQCAVADLQQSRDLAAAVSGAEYDCYHAWFSAPSATLNDIYSEASLSRIQVALDQEIARYRGEAEQARVLENLGEFVRAAYYVRYNAGTGTPEFSEALSQRFAQSTNLFLNNPHALDQGREQVGAMKSLTLMVDNVKQLPLTMDSMMAALMHFNRDTAKDTQWVDGLNNLFRSMAGHAANDAFYRYMANNTHHIDTLARFASDNAWALDTDANFIVFNALRETGRLLASPDQETKRKALAVMQQVMQRYPLGSEHDKLWLAAVEMMSYYAPEGLNGLNLEQAKQDLAARVMPNRFECQGPAIIRSEDLTDAQAAKACEVLAAKEADFHQVANTGNQPVADDLNDRVEVAVFASNDSYVDYSSFLFGNTTDNGGQYLEGTPSRADNTARFVAYRYANGEDLSILNLEHEYTHYLDARFNQYGSFSDNLAHGHIVWWLEGFAEYMHYKQGYKAAIDLIPSGKLSLSTVFDTTYSHDSNRIYRWGYLAVRFMLENHPQDVESLLALSRSGQFAQWAQQVTVLGQQYDAEFERWLDTLEVVVEPEQPGTDPEEPSEPTDPEVQVTELAANQSLQLSGEAYSEKLFYVDVPANTVRFNVSIEGAGDADLYMSYNKVAHYYDFEMSQYADGSNEEIQFAPEQNGYVKAGRYYISLTGRDSYDSVNLVAALEVEAQTPPTQVQDDLAPVVLESGEAKVLTVHQQRYAAVYVPEGVKEVRVWMSSQSNANDPYGAGNVDLYASRKHWPTAEQHEYASNYAGSNEYLAIPVTEAGYVHFSLQAPQQGDDVEMLVYFF

>Mes436

MMKKTITLLTALLPLASAVAEEPTLSPEMVSASEVISTQENQTYTYVRCWYRTSYSKDDPATDWEWAKNEDGSYFTIDGYWWSSVSFKNMFYTNTSQNVIRQRCEATLDLANENADITFFAADNRFSYNHTIWSNDAAMQPDQINKVVALGDSLSDTGNIFNASQWRFPNPNSWFLGHFSNGFVWTEYIAKAKNLPLYNWAVGGAAGENQYIALTGVGEQVSSYLTYAKLAKNYKPANTLFTLEFGLNDFMNYNRGVPEVKADYAEALIRLTDAGAKNFMLMTLPDATKAPQFKYSTQEEIDKIRAKVLEMNEFIKAQAMYYKAQGYNITLFDTHALFETLTSAPEEHGFVNASDPCLDINRSSSVDYMYTHALRSECAASGAEKFVFWDVTHPTTATHRYVAEKMLESSNNLAEYRF

>Mes437

MKHQYFAKKSFLFISMLAAFKTSAFELPSVPFPAPGSDEILFVVRDTTFNTQAPVNVKVSDFWTNRNVKRKPYEDVYGQSVFTTSGTKWLTSYMTVNINDKDYTMAAVSGYKSGHSAVFVKSGQVQLQHSYNSVANFVGEDEGSIPSKMYLDETPEYFVNVEAYESGSGNILVMCISNKESFFECKHQQ

>Mes440

MKRIMLFLATNLAVVLVLSVVLNIVYATTGMQPGSLSGLLVMAAVFGFGGALISLMMSKGMALRSVGGMVIESPRNETEHWLLETVGRQAQQAGIGMPTVAIYDSADINAFATGAKRDDSLVAVSTGLLHNMTRDEAEAVLAHEVSHIANGDMVTMTLMQGVVNTFVIFLSRFIANIVASNDDEEGQGTNMMVYFGVSMVLELVFGFLASFITMWYSRHREFHADAGAARLVGKEKMIAALERLKMSQESKLDGTMMAFGINGKQSLTELLMSHPPLDKRIAALRNQ

>Mes441

MTKQQMIAMFIAMIITSALVSAATIMGGIWYLNKQAQDSGETSSLLENSPLSFLVTEQPTSKGPSFHPLDKVVLSIKGKKQTHFVMLELAIETRRPERIKDIDNYMPMVQNSLLKLFSDKTFDELQQTGAIDILQNEVKQTLLVAFAKTDIVRDIDDVLLTKYVVQ

>Mes442

MLDMNPQETYTAPEEVNTPSRPIDENALLQRHQVMVKRVVNQLRVHATSHCSIEDMQQIGLIALVEAGRRYGDIDDTHFPAFAVCRVRGAILDELRRLDWRSRKTRQQAHELNDVTRDLTRSLGRMPTDSEIIKALGTDEQDYYNRQNAALAGEMQSLDQLMENSTDSHFGGQYDGMEHEHIRRSLDSALGRLSKRDQLLLTLFYQHELNLHEIALVLDLTPPRICQLHKQALKQLNQLMSS

>Mes443

MQKFLGVLTILVCVFGGYMWAGGKLGAIWQPAEFLIIIGAAAGSLIIGNPPHVLKEMRQQVPATIKGPTEEYEYYMELMALLNNLLETARSRGFKFLDSHIEAPEQSSIFLMYPLVSEDHRLISFITDNLRLMAMGQMSPHELEGLLEQEIEAIQNELLLPSRSLQRTAEALPGFGILAAVGGIIITMQAIDGSIALIGYHVAAALVGTFIGIFGCYCGLDPLSNAMAQRVKRNMTAFECVRATLVAYVAKKPTLLAIDAGRKHIQLDIKPTFNQMEKWLAEQEG

>Mes444

MQKQEHVVFKRAKAHGHDEPHGGAWKVAFADFMIALMALFLVLWVMQVVDKEERKAIVAHLHSSSVFDKSYGNPFDTSQSISPIDLAQDSSVPSKHNSNHVVSSYFQGDGDGPEINSLVPGTFDTQEQLAALAKVIEEMTAQINAQGNVNVTVTPQGLRIVLQDDYKQHMFSRGGAELTPFFEDLLLALAPLFEQVTNPLIISGHTDAIPFKKRFGRQSNWALSASRADVARKTLVEGGMPDDRVMQVTGMSDRALLNPDEPDSSENRRIELFILTTPAAKVLETLFGNQDDSELQKAKQKAEFNQPVIRQEVIRYSADAEKQEAKIQAL

>Mes446

MIEIKNVNKVFYQGSKEILALKDINLHIAKGTIFGVIGSSGAGKSTLIRCVNMLEAPSSGSIIVDGVDLTTLSKKQLVETRRNIGMIFQHFNLLSSRTVFDNVALPLELAGKDKSQITTKVTELLKLVGLADKHESYPSNLSGGQKQRVAIARALASDPSVLLCDEATSALDPATTQSILELLKEINRKLNITILLITHEMEVVKSICHEVAIIGGGELVEKGTVGDIFAHPKTELAHEFIRSTLDLSIPEDYQARLQPNRVEGSYPLVRMEFTGATVDAPLMSQISRKYNIDVSILSSDLDYAGGVKFGMMVAELFGNEQDDSAAIEYLREHNVKVEVLGYVL

>Mes447

MNDVIRDFFKMESAGGILLVIAAAIAMTIANSPLGETYQSLLHTYVFGMSVSHWINDGLMAVFFLLIGLEVKRELLEGALKSKETAIFPAIAAVGGMLAPALIYVAFNANDPEAISGWAIPAATDIAFALGIMALLGKRVPVSLKVFLLALAIIDDLGVVVIIALFYTGDLSSMALLVGFVMTGVLFMLNAKEVTKLTPYMIVGAILWFAVLKSGVHATLAGVVIGFAIPLKGKQGEHSPLKHMEHALHPYVAFGILPLFAFANAGISLEGVSMSGLTSMLPLGIALGLLIGKPLGIFSFSWAAVKLGVAKLPEGINFKHIFAVSVLCGIGFTMSIFISSLAFGNVSPEFDTYARLGILMGSTTAAVLGYALLHFSLPKKAQD

>Mes449

MHRYKEEASSLIKLATPVLIASVAQTGMGFVDTVMAGGVSATDMAAVSVASSIWLPSILFGIGLLMALVPVVAQLNGSARREKIPFEIQQGVVLALLISIPIIGVLLQTQFILQLMDVEAVMAGKTVGYIHAVIFAVPAFLLFQTLRSFTDGMSLTKPAMVIGFIGLLLNIPLNWIFVYGKFGAPELGGVGCGVATTIVYWVMFALLLAYVMTSSRLKSINVFGEYHKPQWKAQVRLFKLGFPVAAALFFEVTLFAVVALLVSPLGPIIVAAHQVAINFSSLVFMLPMSVGAAVSIRVGHRLGEENVDGARVASRVGIMVGLALATITAIITVLSRELIAELYTNNPEVISLAMQLLLFAAVYQCTDAVQVIAAGALRGYKDMRAIFNRTFIAYWILGLPTGYILGRTDWIVEPMGAQGFWLGFIIGLTAAALMLGVRLRWMHRQEPDVQLNFSLQ

>Mes450

MINEIKKDAQERMDKSVEALKNNLSKVRTGRAHPSLLSGISVEYYGAATPLNQVANVVAEDARTLAITVFDKELTQKVEKAIMMSDLGLNPMSAGTIIRVPLPPLTEERRKDLVKIVRGEAEGGRVAVRNIRRDANNDLKALLKDKEISEDEDRKAQEEIQKLTDVAVKKIDEVLAAKEKELMEV

>Mes451

MSNIEHGLSFIDIMVFAIYVAIIIGVGLWVSRDKKGTQKSTEDYFLAGKSLPWWAVGASLIAANISAEQFIGMSGSGYSIGLAIASYEWMSAITLIIVGKYFLPIFIEKGIYTIPEFVEKRFNKKLKTILAVFWISLYIFVNLTSVLYLGGLALETILGIPLMYSILGLALFALVYSIYGGLSAVVWTDVIQVFFLVLGGFMTTYMAVSFIGGTDGWFAGVSKMVDAAPGHFEMILDQSNPQYMNLPGIAVLIGGLWVANLYYWGFNQYIIQRTLAAKSVSEAQKGIVFAAFLKLIVPFLVVLPGIAAYVITSDPQLMASLGDIAATNLPSAANADKAYPWLTQFLPVGVKGVVFAALAAAIVSSLASMLNSTATIFTMDIYKEYISPDSGDHKLVNVGRTAAVVALIIACLIAPMLGGIGQAFQYIQEYTGLVSPGILAVFLLGLFWKKTTSKGAIIGVVASIPFALFLKFMPLSMPFMDQMLYTLLFTMVVIAFTSLSTSINDDDPKGISVTSSMFVTDRSFNIAAYGIMIVLAVLYTLFW

>Mes453

MISFGNVSALQAAMPQARNEILNEGKLSIGGKEYTINAATQEFTRANPTSGAVARFFEATGKLFREGSTQSVAKAITKAVFDNEQGQAQRLQTSSSVEHGQMLFKDANLKTPSDVLNAFAKLDSKMVKSHAAELSQLAERAMTEVMLETDSGKNLKALIGDDAVKSLAVRVVKDYGGGVAAAQKNPEVRINQMQAVFDMEVMHLKAAQRHIEGLASTDLNQGVYAEGLPEDAFNKAGVTNNVERAAAWIINASNSKGNDAENITSLLKEYATNGKDLLNMDNLKELHARLVPNVERDYRGPNISGGTLPSSIGGEGMLKQHIEGFLKENPVADKDLGKHLFAGVIGYHGFTDGNGRMGRMLYAIAELRNDSFNPLAMNAENSLHGIK

>Mes454

MRQGTFFCIDAHTCGNPVRLVAGGVPPLEGNTMSEKRQYFLEHYDWIRQALMFEPRGHSMMSGSVVLPPCSDNADASILFIETSGCLPMCGHGTIGTVTTAIENRLITPKEEGRLILDVPAGQIEVHYQTKGDKVTSVKIFNVPAYLAHQDVTVEIEGLGEITVDVAYGGNYYVIVDPQENYAGLEHYSPDEILMLSPKVRTAVSKAVECIHPNDPTVCGVSHVLWTGKPTQEGATARNAVFYGDKALDRSPCGTGTSARMAQWHAKGKLKSGEDFVHESIIGSLFNGRIEGITEVNGQTAILPSIEGWAQVYGHNTIWVDDEDPYAYGFEVK

>Mes455

MPITSKYTDEQVEKILAEVALVLEKHAASPELTLMIAGNIATNVLNQRVAASQRKLIAEKFAQALMSSLETPKTH

>Mes456

MTTHKFEHPLNEKTRIYLRVESLLRQAHLASGFADNHQYQLFFRALFDMVEIFEQIQLKSELAKDLEKQRLSYRHWLNVEGVDQEALNSLLNEIDVVHSQLMGAERFGQALKEDRFLSSIRQRFNLPGGSCCFDLPALHYWLHLPIERKKHDANQWQKSLKPLSDALTLWLKLARETGHFKAQIARAGFFQSDADEANILRLHIPMKYGVYPMISGHKNRFAIKFMAFENGQACSQDVEFELAVCS

>Mes457

MSIKENSYFAGGVKSLGFNQHGQDVSVGVMLPGEYTFGTQAPERMTVVKGALVVKRVGEADWTTYSSGESFDVEGNSSFELQVKDATAYLCEYL

>Mes459

MAKNNLGTITQVTGAVVDVKFEGELPSILSALETDNHGNRLVLEVAQHLGESVVRTIAMDSTEGLVRGQQVTSTGGPITVPVGPQVLGRIMNVIGEPVDERGPVVTAQRYPIHRQAPTFAEQATETEILVTGIKVIDLIAPYTKGGKVGLFGGAGVGKTVLIQELINNVAKGHGGYSVFAGVGERTREGNDLYHEMIDAGIIDLEGDKSKVALVYGQMNEPPGARARVALAGLTQAEYFRDEEGQDVLFFVDNIFRFTQAGSEVSALLGRIPSAVGYQPTLATDMGALQERITSTKKGSITSVQAIYVPADDLTDPAPAASFAHLDATTTLNRSIAELGIYPAVDPLDSTSRALDPLVVGEEHYKVAREVQRVLQTYKSLQDIIAILGMDELSEEDRLVVARARKIQRFLSQPFHVAEVFTGSPGKLVSLEDTIKGFKGLVEGEYDHLPEQAFYMVGNMAEAIEKAKKMAAEAA

>Mes460

MISLALAAETAEHGGEAASHGGLFADPAFWVSIAFLMVVGFVYIKAKNKILGALDGRGAAVKAKLDEARKLRDDAQALLAEYQRRQRDAMKEADEIIRHAKDEAARLRAKAEADLEASIRRREQQAVDRIAQAEAQALAQVRNEAVDVAVSAARSLMAGSLAKADQNRLIDAAIADLPGKLH

>Mes461

MPPIKENVIIYANPDHCLSCHSCELACAVAHSGGHDMIEAIAANLPLHARNKVVSVDGTAMPMQCRQCEDAPCTFACPTGACRQADGQVQIVEQHCIGCKLCVMVCPFGAITVRSETVVEQGACTNRGVAKKCDLCVDWRASTGKTAPACVEACPTKAIRMVDLDAYRIALREARAREIAKSHRHMRVQF

>Mes462

MTHHDCAHCSSDACATEMLNLAEANSIETAWHRYEKQQPQCGFGSAGLCCRICLKGPCRIDPFGEGPKYGVCGADRDTIVARHLVRMIAAGTAAHSEHGRHIALAMQHISQGELHDYSIRDEAKLYAIAKTLGVATEGRGLLAIVGDLAAITLGDFQNQDYDKPCAWLAASLTPRRVKRLGDLGLLPHNIDASVAQTMSRTHVGCDADPTNLILGGLRVAMADLDGSMLATELSDALFGTPQPVVSAANLGVMKRGAVNIAVNGHNPMLSDIICDVAADLRDEAIAAGAAEGINIIGICCTGHEVMMRHGVPLATNYLSQELPILTGALEAMVVDVQCIMPSLPRIAECFHTQIITTDKHNKISGATHVPFDEHKAVETAKTIIRMAIAAFGRRDPNRVAIPAFKQKSIVGFSAEAVVAALAKVNADDPLKPLVDNVVNGNIQGIVLFVGCNTTKVQQDSAYVDLAKSLAKRNVLVLATGCAAGAFAKAGLMTSEATTQYAGEGLKGVLSAIGTAAGLGGPLPLVMHMGSCVDNSRAVALATALANKLGVDLSDLPLVASAPECMSEKALAIGSWAVTIGLPTHVGSVPPVIGSQIVTKLVTETAKDLVGGYFIVDTDPKSAGDKLYAAIQERRAGLGL

>Mes466

MKRMMIVAALAALTTTTVAQAADPAAYVEYRKSVLSATSNYMKAIGITLKEDLAVPNQTADHAKAIASIMETLPAAFPEGTAGIAKTEAKAAIWKDFEAFKVASKKSQDAALELASAAETGDKAAIGAKLQALGGTCKACHKEFKAD

>Mes468

MTGPSVHDRALGAFLGLAVGDALGATVEFMTKGEIAQQYGIHRKMTGGGWLRLKPGQITDDTEMSLALGRSLAAKGTLDVADICEEFALWLKSRPVDVGNTCRRGIRRYMHEGTTTAPYSEGDAGNGAAMRCLPAALATLGHPADLEPWVLAQARITHNHPLSDAACLTLGRMVHHLIGGRGMKACREEANRLVHQHRDFHFEPYKGQSSAYIVDTMQTVLHYYFVTDTFKSCLIQTVNQGGDADTTGALAGMLAGATYGVDDIPSGWLSKLDMKVEREIRRQVDALLALAGLD

>Mes469

MKDMGEDRPGIGHSTNLVGLPTDLLASAWFNQAAPEIHIAGVREMNRSLFEMLAEAPDLESAGEAFYKYMIAMFGLDPEQQDHRPGQGGAVRRFHASYLRLLKGWGYDTNAKEGAVLKGWVESRFGLFPTFHREPITKFASKAWITYIEEKMTSRFHNNSIYVQLDLMYEFCQWALARFAAPGESALLLYRGVNDFTEHQMIERIDNRQVVVRMNNLVSFSSDRGVADCFGDTILETRVPVSKIVFFNTLLTSHPLKGEGEYLVIGGDYLVKASYL

>Mes471

PYVVTENCIKCKYQDCVEVCPVDCFYEGENFLVINPDECIDCGVCNPECPAEAIAGKWLEINRKFADLWPNITRKGPALADADDWKDKPDKTGLLSENPGKGTVCH

>Mes473

MWRIWQLFDPRQALVGLATFLFVLALLIHFILLSTERFNWLEGASTKPVQTSMVMPSSDLAV

>Mes474

MAEVKQESLSGITEGEAKEFHKIFTSSILVFFGVAAFAHLLVWIWRPWVPGPNGYSALETLTQTLTYLS

>Mes476

MSALRQIAFYGKGGIGKSTTSQNTLAALVEMGQRILIVGCDPKADSTRLILNTKLQDTVLHLAAEAGSVEDLDVADVVKIGYKGIKCTESGGPEPGVGCAGRGVITAINFLEENGAYDDLDYVSYDVLGDVVCGGFAMPIRENKAQEIYIVMSGEMMALYAANNIAKGILKYAHTGGVRLGGLICNERQTDKEVELAEALAGRLGCRLIHFVPRDNGVQHAELRRQTVIQYAPDSKQAGEYRTLATKIHNNSGQGVVPTPITMEDLEEMLMEFGIMKSDEEALAELEAKESAAAN

>Mes477

MKIAIPKERRPGEDRVAISPEVVKKLVGLGFEVIVEQGAGVGASITDDALTAAGATIASTAAQALSQADVVWKVQRPMTAEEGTDEVALIKEGAVLMCHLGALTNRPVVEALTKRKITAYAMELMPRISRAQSMDILSSQSNLAGYRAVIDGAYEFARAFPMMMTAAGTVPPARVLVFGVGVAGLQAIATAKRLGAVVMATDVRAATKEQVESLGGKFITVDDEAMKTAETAGGYAKEMGEEFRKKQAEAVLKELVKTDIAITTALIPGKPAPVLITEEMVTKMKPGSVIIDLAVEAGGNCPLSEPGKIVVKHGVKIVGHTNVPSRVAADASPLFAKNLLNFLTPHVDKDTKTLVMKLEDETVSGTCVTRDGAIVHPALTGQGA

>Mes479

MTHSLTMAAYIVAGVLFILALRGLSNPESARNGNRMGMVGMAIAILTTLLSPSVQAYAWIVLAIAIGGAIGTVIAKKVLMTALPQLVAAFHSLVGMAAVLVATGALLNPEAYGIGSAGAIHAGSLVEMSLGLAVGAITFSGSVIAFGKLQGLIAGKPVTFPMQHPLNAVLGILLVVLLVVFAATESHTAYFALMILAFALGFLLIIPIGGADMPVVISMLNSYSGWAAAGIGFTLGNPLLIIAGALVGSSGAILSYIMCKGMNRSIFNVILGGFGSEGGVAAAGGAAGDRSVKAGSAEDAAFIMKNASKVIIVPGYGMAVAQAQHALREMADVLKKEGVEVSYAIHPVAGRMPGHMNVLLAEANVPYDEVFELEEINSSFQTADVAFVIGANDVTNPAAKTDPSSPIYGMPILDVEKAGTVLFIKRSMASGYAGVENELFFRNNTMMLFGDAKKMTEQIVQAMN

>Mes481

MDQSSRYVNLALKEEDLIAGGEHVLCAYIMKPKAGYGYVATAAHFAAESSTGTNVEVCTTDDFTRGVDALVYEVDEARELTKIAYPVALFHRNITDGKAMIASFLTLTMGNNQGMGDVEYAKMHDFYVPEAYRALFDGPSVNISALWKVLGRPEVDGGLVVGTIIKPKLGLRPKPFAEACHAFWLGGDFIKNDEPQGNQPFAPLRDTIALVADAMRRAQDETGEAKLFSANITADDPFEIIARGEYVLETFGENASHVALLVDGYVAGAAAITTARRRFPDNFLHYHRAGHGAVTSPQSKRGYTAFVHCKMARLQGASGIHTGTMGFGKMEGESSDRAIAYMLTQDEAQGPFYRQSWGGMKACTPIISGGMNALRMPGFFENLGNANVILTAGGGAFGHIDGPVAGARSLRQAWQAWRDGVPVLDYAREHKELARAFESFPGDADQIYPGWRKALGVEDTRSALPA

>Mes483

MSEYQNILTGVQVRTAPHSAPIAKGIFPRLGKPGFSYWLGKIGDAQIGPIYLGTTGVLSLVFGFFAIEIIGFNLLASVNWSPMEFGRQFFWLGLEPPAAEYGLGFAPLAEGGWWQIAGFFLTTSILLWWVRMYRRARALKMGTHTAWAFASAIFLFLSLGFIRPLLMGNFSESVPFGIFPHLEWTNSFSLNYGNFFYNPFHMLSIAFLYGSALLSAMHGATILAVSRLGGDREVEQITDRGTAAERAALFWRWTMGFNATMESIHRWAWWFAVLCTFTGAIGILLTGTVVDNWFEWGVKHGLAPAP

>Mes486

MASKEEILAGLAEIVNEETGLDTAEVQPEKSFTDDLDIDSISMMTIVVNAEDKFGVKIPDEEVKNLKTVQDAVDFIXGA

>Mes487

MERRVLIVESEHDFALSMATVLKGAGYQTALAETAADAQRELEKRRPDLVVLRAELKDQSGFVLCGNIKKGKWGQNLKVLLLSSESGVDGLAQHRQTPQAADGYLAIPFEMGELAALSHGIVPPGTDDTGASLDAALNGTREAPPPMPPSLKAAAGGPPKLPKRERRSAMTEEDRAFLDRTFQSIADRKAELLAESRQLKRPPPRRELMGTPEGKIQILRDELKTREAQLARLSEIWNVRERELLSGEDRIHEKDVELQGLKMQVDDLLRRFNEAQQATIQKEREHGATVDDLLLQKFSAEKDLIEVVASKEKDINLLRREVSRAEEELSRRAGELEHGRNEYDKLEKHLGVVTLEFEVKEQKLQDTVLANEGEIARLTKRGDDFEAELNRTISERDQRFAELDGEIQALQERLQQTEQERDTTVRGLEARAARAEEHGTQADAEIHRLNAERDALEAKLSQQVADLEADLARTMGERDQLRLDKDAQEAELTQRIEERDAKLGTLERELSETIARNEHTEAELNANIQQQLERIGELEGEVEAVKTHLEDRENELTAELQALGQAKDELETDLNDRLQALSQAKDALEADLSRQLEELRSAKAELEADLTGQIQALTSQLEETQRQLDDSQRTGEQLSARVAQLEDTVSQRESTIESLQGDVAARDQRISELSGDLEATSQTLAQTQQTLAQTEQQLADTQNTLASTEGALAETRGELDATSQTLQQTQQTLAQTEGALAETRGELDATSQTLAQTQQTLAQTEQQLADTQNTLASTEGTLAETRGELEATSQTLQQTHAALEDTRGALQETSDTLAHTTRERDQRIAELADLGAAKDALEQELTGQIGHLRSELSETQGNYEAERAAHEKLAAESSAHIGDLTSERDGLRSELEATSQTLEQTHGQLAATRDALAREQHAHQESRKAAASTQTTLEGQLAEARAHGEDLGEHLTLTKHELGTRVAELTQLTATLAQTENTRAHLEERLHTLTEESQRREELLQNDLTQKGTELSDTLRKLTHVTQEKMRQAEVLNREVATRTEQLKAMEAKLQTQATEARRQAEGLGQQITGLNEQLEQGRKALAGREDQLRAAGAAQQKLTAERDGLAGQLQQAEARLQQQAQQANQERADAKRAADELAAKLAKTEQRITQFAQDAQTQATEADARAKDLQGQLSARAKKIQDLELAVENAQGAKSRAEKELNAKVAAAESKAHEASTRLAAAQKERKDLEARHAKEQEDLAAKQKAELERRDAIKAQEVARLQQSVQEKSKALKVAELELARYKSKSATTATPAKAAAKPAAAEDDELAVRTQLNQVIAPAAAAQAPAPAKKPAAKPAAQAPAKKAPAPAPAPPAALSDESEPTDRTLVIQLPTAKEDDDWTALVDELDK

>Mes488

MSDTLPALRATLTELVAMDTTSFRPNVPLIDYAQARLEAAGFSAERQKFLDDAGVEKVNLVAVKGGSGSGRAALALVGHSDCVPYDAAWTDALRLTEKDGRLYARGACDTKGFIACALHAALNAEQLKAPLMVVLTADEEVGLTGAKKLVEAGLGRARHAIVGEPTRLIPVRANKGYCLAEVEVRGKEGHSAYPDSGASAIFRAGRFLQRLEHLALTVLREDLDEGFQPPFTTVNVGVIQGGKAKNVIPGACRFVVEWRPIPGQPPERVSQLLETIRQELVRDEPAFEAQIRVVRTDRGVNTRADAEVVRFLAEASGNAPETVSFGTEAPQMTELGAEAVVFGPGDIRVAHQTGEYVPVEDLVRCEAVLARAVAHFCGGR

>Mes489

MEIRADEISRIIREQIKDYGKKVTVAETGTVLSVGDGIARIYGLEGALAGELVEFANGVQGLVLNLEEDNVGVAIMGDFQAIREGDTVKRTQQIASVPVGKELLGRVVDPLGKPLDGKGPIAATETRRLEVKAPGIVSRKSVHEPLQTGIKALDALVPVGRGQRELIIGDRQTGKTAVAIDTIINQKGLNVYCIYVAIGQKQSTVAQVVEKLNRYGAMEYTTVVASNASDPAPMQFFAPYAGVAMGEYFRDNKMHALIVYDDLSKQAVAYRQLSLLLRRPPGREAYPGDVFYVHSRLLERAAKLSDEEGAGSLTALPIIETQAGDVSAYIPTNVISITDGQIFLETDLFFAGVRPAINVGLSVSRVGSAAQIKAMKQVAGTMKLELAQYRELAAFAQFGSDLDKATQETLARGARMVELLKQGQYEPMPVEKQVMQIYAATNRDDPKKRGWIRDIPTADVVRWMREFLEFADGKHPNVAKDLASKRELTADIKTALSKAITEFNEVFQPTPGAKV

>Mes490

MERFRDGAQDAFEDLFARHAPRVQGFLARMVRNGALAEDLLQATFLSVIRSRGRYEPGTRFIPWLMTIAANAARDALRHQRHVDAYASREDTATPASAAPDDSDPSLRRHLLDALQQLHPDHREAVVLSKVEGWSFEEIGALRGISPGAARLRAHRGYEKLRELLGELELEVAR

>Mes491

MKPPMDLDSLLTQTPAKDNAALERVLAAARGELALRRPVRRWRTQAVGLMAASAGLGLLAAVVLLAVGAVTGPLLLARAPLLAMLVGTSAVCAWGALSPKGRWMRRLGVGLAVVSAAALVLARGAPHSPPSFPGWVCTVSHLAIGVVPLVVALFALRGAFFQPLRAVVAGLSVGSTGALLGELACEQDWRHVLSHHLLAWVVITVVLVVISKSLKPRSYAP

>Mes492

MIQDPSLIICHDVDGAPVRIGAKVKVVPHSEDGTISQRFLGQTGIVVGLVFDDPATQYPDDPLIQVLVEGLGEDLFFPEELELAPEWARNRIAQHRQAVRTGGRSSLERLP

>Mes493

MASEGGSVRHVIVVGAGPGGLSAAINLAGQGFRVTVVEKDAVPGGRMKGLTLGASGEYAVDTGPSILQLPGVLEQIFRRAARRLEDYVKLLPLDVNTRVHFWDGTHLDTTRHLDRMEAELAKFGPRQASALRQWMEDGREKYGIAYQKFICTSADNLGYYAPWRLAPTLRFKPWQTLYRQLDGFFHDDRVTYALAYPSKYLGLHPTTCSSVFSVIPFLELAFGVWHVEGGFRELSRGMMRCARDLGATFRMGTPVEKVRVDAGRAVGVKLVGGEVLDADAVVVNADLAYAARSLIPAEAREGSRLTDAALERAKYSCSTFMAYYGLDTVYADLPHHLIYLSESARRTDRDALEDRHVDLEDPPFYVCNPGVTDPSGAPAGHSTLYVLVPTPNTGRPVDWVKTEQALRERIPAMLEKVGLKGVREHIREERYFTAETWRDDFNVFRGAVFNLSHTWLQLGPLRPKVKNRDIEGLYFVGGGTHPGSGLLTIMESANIAADYLTREAGKGPLPGWPYVPPLEPESPVQARAG

>Mes494

MRAFATNVCTGPVDVLINNAGVSGLWCALGDVDYADMARTFTINALGPLRVTSAMLPGLRQGALRRVAHVTSRMGSLAANTDGGAYAYRMSKAALNMAVRSMSTDLRPEGFVTVLLHPGWVQTDMGGPDATLPAPDSVRGMLRVIDGLNPEHSGRFFDYQGTEVPW

>Mes495

MANITVFYNEDFQGKQVDLPPGNYTRAQLAALGIENNTISSVKVPPGVKAILYQNDGFAGDQIEVVANAEELGPLNNNVSSIRVISVPVQPRARFFYKEQFDGKEVDLPPGQYTQAELERYGIDNNTISSVKPQGLAVVLFKNDNFSGDTLPVNSDAPTLGAMNNNTSSIRIS

>Mes496

MSLHVFAAFVTPLGTAANNRGLTEGNITSLQKLVWNGQVHTTVSAESIRFALRRRLNEQEPCNRTYDDASRANAWKDAAFSAWSGKSKEKTYIDDDLLGFMSAEGAKQEKEKGTAKVRRAVLEVSRAVSLTPWSGDVTFNAASPGATPSAQKKGSNPVPYGTEMHATRYQYGVALTPEALRVPARAVTALNQLCALGPVAGNHGRFLFDFSPESVVFRLTQEAAPRILYAFEPSSRAGGVELAALLRKVKSGDVPAKELVLGGQVVEGLGAEEREVLSGAELHTGVVAACRAACKRLEVRKK

>Mes498

MSLDTPNEKPAGKARARKAPASKAGATNAASTSSSTKAITDTLLTVLSGNLQARVPKELVGESGVELAHLLNQVLDQFAASEHRKHVAAQEIDQALDALIGLVREGDLSRWNTTTEDPQLGPLLEGFGKVIETLRTFVREINEAALRLSSSANQVLAASTQHETSSTEQAAAIHETTATMEELKHASAQIAENAGSVARVAEETLGAARAGRGAIGEFIQAMQQIRSDGVAVADSIAKLSKRVERIGTVVEVIDEIADRSDLLALNAALEGSRAGEAGKGFSIVAAEMRRLAENVLDSTKEIKNLITEIREATAAAAGAAEASKSATESGEKLGAVAAQAVEGILAGVQETSDAARVINLATQQQRTATEQVVASMAEIEDVTRQTTQASKQATGAAAELTQLAGRLAELIKRFKAD

>Mes499

MDTEALKKSLLKKFQEVTADRLQKIQLGVLDLEKETADQAAEDVARELHTMKGEARMLGLAAIGQLAHAAEDVLRAEREGKTATEVATDVLLRACDVLSDLNEDLSGANTGNPASEEMVRMLAEVSGQTPPAIAGARPVAPPPAPPPAPVAAPVVTPAAVAAPPAPVQAPVAPPPTQAPVAEPGAHAAAAAPHPAAAHGRDEEAPSAAKSAVADRSIRVNVEVLDALGLLAGDLLVESARGRLRSSETEALFERFSRLGDRFLRLAEEIDISNEVREQLDRVESDLHMLRDDAFRFVRRNDDGINTLHGNLAKMADHVAEARLVPLSTVFDAFPRAVREMSRTQGKEVDLVIENADIGVDRSMLGDVRDALVHLLRNSVDHGVESPDTRQQLGKPLNGRIRIRVRVDGDMLHIEVEDDGRGIDPERLRQAAISKRLINAVQAAALSEREAIELIFRPGFSTRDQVSELSGRGVGMDVVKRKVETLGGSVGVSSRIGRGSTITLRLPQSLALMKVLLVRLGDDVYGMPAADVEAVMRVKPDDRLEIFGTLAVRHRGKPTALVALGPLLGLNGGNRFDKPPAVVVRHGEDHAALVVDGFVDEREVAVKPCGGEFLKAAPFIAGTAALEDGRIAVLLHVPDIMAEVRRMARPVTQAPAAKRLRVLLVDDSPIARATEGALVKALGHSVEEAQDGEEAYVKVQNNTYDLILTDVQMPKLDGFSLARRLKSTPAVARIPVIILSSLASPEDKRRGLDAGADAYLVKGELGVEVLAQAIDRLT

>Mes500

MIREQRSSRGGSRDQRTNRRIRAREVRVVGSDGSQLGVMPLEAALDRARTEGLDLVEISPMASPPVCKIMDYGKFKYEEKKKASEAKRAQVTVLLKEVKLRPKTEEHDYEFKVRNTRRFIEDGNKAKVVIQFRGREITHREQGTAILDDVAKDLKDVAVVEQMPRMEGRLMFMILAPTPKVAQKARELVRQAATAAKRPPPPGAPGAGKSAAGASSGAEEKAEETAEEKKEAQAAPAAAEAQSPTAS

>Mes502

MSDEKKKGSAASAMPTAMAPPGLINKEDIPQVLPILPLRNSVFFPGGVLPLAVGRQKTIALIKDAVRDDQVIGVVTQRRAEEEDPGAADLYTMGTVARIVKLLKMGEDNYSLVVQGLARFRVVELVQEAPYLKARVDAVEDKTSSENVEVEALGINLKKLAREVIELMPELPAAATELVESITHPGHLADLIAANVDVPIEEKQAVLETVDLKARMKLVLELLNRKREILKLSNKIDSAVKGEMSKTQREYYLRQQLKAIKEELGEMGEEEEELDELQERLKKAGLPPDVEKVANKELNRLKTIPAASSEYTVARTYLDWIADLPWAKISEDNLDIENARQQLDKDHFGIKKVKKRILEYLAVRKLKNDMRGPILCLVGPPGVGKTSLGQSVAKATGRKFVRLSLGGVRDEAEIRGHRRTYVGALPGRFIQSMKKAGTKNPVMMLDEIDKLGADFRGDPSAALLEVLDPEQNNTFSDHYLDVPFDLSKVMFVATANQLDPIPGPLRDRMEIIELTGYTFEEKQSIARIHLVPKQLKEHGLSPDHIDITDEALLTLTTAYTREAGVRNLERRIADICRAVAVEVAGGKTEKQTINADRVKEILGPEMFYSEVAERTEVPGVATGLAWTAAGGDLLFIEATKMAGKGGMTLTGQLGDVMKESATAALSYLRSKAEQLGISPNFLEKTDLHLHFPAGSIPKDGPSAGVTILTALTSLLTGIRVRHDTAMTGEATLRGLVLPVGGIKEKVLAAHRAGIKRVILPERCRKDLIDVPDQARNELEFIFVTHMDDVLKAALETPPVGVAGTPGGEPGKEAPLPKPAESAPEVRA

>Mes503

MSPPQTTLPVTEAGLVPLLQPYGPYVLVRKLAEGGMAEIFLAKLLGADGFERNVVIKRMLPHLTNNPDFVEMFRDEARLAAKLAHPNIVQIQELGFAEGCYYICMEYLAGEDFSTTLRLAGRKRHYVPLPVVLRVLIDAARGLHFAHEFTNEAGQPLNVVHRDISPSNLYLTYQGQVKVLDFGIAKAESRLVNTRTGVVKGKYMYMAPEQARGKEVDRRADIFALGVSLYEALTHVRPFSRENDLAVLNALLQGELKPPRELRPDLPEELEAILLKAMAFKPEDRYPTAEAFADALETFLSEHLSGSGAMPLGAFLKGHFGEERFTERSRIPTLATLTATYGGAAAGAQGQAPGAEPHGTNLYGVLAREGDATSAQRPGMSMRPSSPGVPAHGAASRGSTSPESAPTAGGRRWRTLAVGLAGGLMLAAAGIVGYRQWMTTPASVSLVPATVPVVEAVAPEAAAAQVGAPMEAVAPVGAAAQAGSLTDAVANGAGGDVGETDSAQLSVDAAGVTETDEAGLAGAASDVEAEADEEGADAAPVRSKKASSQKRVTLGIDDVQRVVSRGRARITTCFERYKADLPSSQGEVQVQLTIVSSGKVRAGTRGPLASSGVGRCLEAQAERLRFPPHRDQEVTVVMPFSWRVTQ

>Mes504

MAAYLVQNQWGGSQATWNPGGLWLIGARDKQNVVALDIKSDDGGKTLKGTMTYNGEGPIGFRGTLSSANNYTVENQWGGTSAPWQPGGVWVLGARDKQNIVAVSIKSNDGGKTLTGTTTYNGEGPIGFKSEVTDGDTYSVENQWGGSAAPWHSGGVWVLGTRGKQNVINVDAKSNDGGKTLSGTMTYNGEGPIGFRGTLTSPDTYTVENQWGGSTAPWNPGGFWMIGARNGQNVVALNVASSDGGKTLAGTMIYNGEGPIGFRARLG

>Mes505

MSFINYSSREINCKIVYYGPGLCGKTTNLQYIYNKTAAETKGKLISLSTETDRTLFFDFLPLSLGEIRGFKTRFHLYTVPGQVFYDASRKLILKGVDGVVFVADSQIERMEANMESLENLRINLAEQGYDLNKIPYVIQYNKRDLPNAVTVEEMRKALNHRNIPEYQAVAPTGVGVFDTLKAVAKLVLTELKKGG

>Mes507

MHLNRVLRETGVVVAAGLLYGSAAFAQSSTIIGTVIDAQSRQPAADVVVTATSPNLQGEQTVVTDAQGNYRIPQLPPGDYTLRFEKEQFKPYARSAIQLRLNRTIRVNVELLPEALGEVVEIVGAPPTIDVGSTTMGVNVDQEFIKRIAVARPGGKGGATRSFESLAELAPGAQNDNYGVSINGSTSPENGYVVDGLSTNDPAFGVNASPLSIEFVQDVNIITGGYMPEFGRSTGGVINAVTRSGSNEFHGSVFANWTPGTLEGTRKQIREEGTVITGQNQLQNLGDFGATLGGPILKDKLWFFAGFAPSFTRYQHTRTLNALRVDDEGNTIKDETDFTVADAIPGSARKYYADSRTIQYMGKLTYLINQDHNVSFALNGTPTSTGGLGKLSVNPQSGGLPGVLATRPGDFGLTETKANTTSLALKYAGAFADKKVLVDANLGWFHQTASTLPGDGSNLGDRTGLAGYSRMVYTTPRALTLFEALPEGQEGACGSTPEEQLVRSPVTGYGVGGPGFMSDQTLDRYQANAKATYLLNALGTHVFKAGVDVELLSFDQVKAYGGGVFFQEGSNYGVAGQGPAVHDARRYGYQTGPDSAVTQFTQVAKTTSTTVGGFLQDSWSIANRVTLNLGVRYDVQALYGGNGDLSLLLGNQWSPRIGAIVDPFANGRAKVFVNFARYYEQVPLNLMDRAFPGENRISARRSLAEPGQGTATSCDPSSFESQQATCNTDSNLLAIPESSRNVNRFYTGGTVGGTPVDPDIKAQSSDEIVVGAEYEVLANTRLGASYTHKDMNSVIEDMSRDDGNTYFLGNPGSGFAGEFPTPVRNYDNVTVYLNRTFADGWLAQANYTWSRLYGNYPGLFRPETGQLDPNILSDFDLIELLENRTGLLPFDRTHQIKVFGAKEFNISNALSASVGVSYRGSSGTPINYWGSHWAYLQDESFVLPRGAGGRTPWINTIDSNIGVNYRVSKDSVVSFTLDVFNLFNFQGVNTVDQTYTLRDIKPIPGGTPADLENLPGRVEFQDQAPRDEPFGSVDGDVNKNFKNPLSYQAPRQVRFGIRYTF

>Mes509

MRTLTCVPDESTAKVHTCRAPFMLHRVMIPPDPIQRFAELFERAKQAIAVDPNAMVVATVGDDGRPSARVVLLKDFDARGFVFYTNHESRKGREARAHPYAALCFYWQPLNEQVRVEGRVERVTDAEADAYFQSRARGSQVGAWASLQSQPLATREELEARVAEVEQKYAGQPVPRPPHWSGFRVVPDRIEFWHAQESRLHDRHVYLREDGGWRTQMLYP

>Mes510

MRVSRFNPRNRGFTLIELMIVVAIIGILAAIAIPNFIKFQARSKQSEAKTNLKALYTAQKSFFSEKDRYSDFANEIGFAPERGNRYGYRVSAAAGDCEVRNAADLPVPAAGVPCISNDSFRFGANSAIDDPTPVVARFVPQGAAGWNTTLGVQPTIADCPNCNFFAGARGNADNEATFDDWVIAGFEGSGQVGPCSEAGNVASGTPYNTRNDVACDGAAQ

>Mes511

MPEVSSGGGCGACGRRHGADASCPTLVRADVRAGGTAHPRCAPVVEAQDPLVGVRCGSFRLVRRLGRGGMGAVYLGEHVSIGSRVAVKVLHAHLTMYPELVQRFHAEARAVNLIGHENIVSIFDMDATPPRPYLIMEFLDGAPLSAWVGTPLAAGAVVSVLSQVCDALQAAHARGIVHRDLKPDNIFLVRRNGNAPFVKVLDFGIAKLADAHMPQTHAGIIVGTPEYMAPEQSLGRGVDGRADLYALGVIAYQLLTGRLPFNDEGLAAQLVAHQLRPPPPPSSVYPAVSAALEHVILRALAKKPEDRYASIAAFRNALQVALAEHVRVSARKTRPGGLAVLERAPVAPDMPTEGQSRGRLGVDARAGHVPSSLASTSQRRLAPAAPAVPRASLVEVPVQVVLRPGESPVRLRGSGLSRGGLFLHGGRVLPPLCSRLPVVLELASGPLSVMCEVVRVVPPAQARVWGMPTGFGVQFVEATAVLKAAVDALLQGEPVRAVPQVPLTEDPAVARLLEAWRQRSAGDAYAVLALEPDSDMGTVRLRTREAWRSLESLEQHSLTPPQRAQVDALRVRVREAAEALGATVQRALYDAWRGNHRGVAKCLEAGLTAEQLESLRREFLARRPQAMGTARSHFQSGGALERDGQLSQALDQYERGLKLAPLEVDMLQRYRRLRRVLGGRATAPTGHDRARSP

>Mes512

MPLKVIGPYRVLETLGSGGAGTVYRALDRRTTDEVALKLLSAGPARDARAARRLAREFDTLVDLSHPNVVKVFESGVHQGVPYLAMELIEGLTLRHYLDLSSGDRQTPPGSHTPRSPLSVLRTADDDFGPLSRSFSDSMDDSEDSPFDGTFGLEAFAEEAPSEDLESFASSASPHVGIGSDDSLEGFDLPPPMPRPAEPEEEPGRVVREEDLNRPERMGRLKDAMLQICEALAYIHGHGLVHRDLKPSNIMVDDDRQVRLMDFGLAKFLADDAAITEAGKLVGTYRYMAPEQILGEPLDGRADLYSLGVILYELLSGRPPFDAKTPHELWRQVLETEPPPVLALNLHGDPQLARVAHRLIRKEPDDRFQTAEEVYEALSE

>Mes513

MQIGKYQLVRKLASGGMAEVFLAKAAGPRGFEKTLVLKRILPHLAEDAAFVEMFLGEARLAAQLEHPNIVQIFDFGEAEGSFFLAMEFIDGPNLRKLVKRAAEEALPPAFCAKVVAAAAEGLAYAHEFRDVETGEPLGLIHRDVSPDNILVSRQGAVKVVDFGIAKVAGQGHRTLTGVVKGKVAYMPPEQLQAKAMDRRVDVYALGVVLYELLTGKRPFDATTDVSVMQAILFESFIPVSARRPDVPVALQQVLDKALAKDRERRYADCRALQDDLERFVLSTGEPVGAYQIAQRIAQWVPEVAAAPAMTPSQGGSKGAVASQAKADARSASMVSPPVDSTSPTTPMPRSLVAPVEVPADSTSPTTPMPVAIGGVVQALEPRSSPQQDTLQSYPVVVKTPALRADASARGASRPRAQSRASGVKVQAPQARDEDVVAMAAASSPPSGGASPAPTTPEDADDAVHTRSTEYAATVPSGRPGGRIAGIVGAVVALLVGGAVTVMRGDDSEVSPVRVNPPPLTHLPREPAVPSQGGRNVPQEKPAANVNAGARDSNDGAQAKPQVSTDVSVVPQEPHVARDATTPEAGLPTVKDAPPENGGAASKEGSAVVAKREPAKASGDPEPNPSRVRERAPTQKVAAVAKGRLEFRIRPYAVVSLDGKVLGQTPFAAVEVPEGRHTVRLVNKELGKDVTRTVDVKAGQATVFKLNLEAE

>Mes514

MHHMPRTTGMNVAVVGGGISGLAVAHHLRSRGTDAVLLESSARLGGAVGTHALAGYLVEQGPNSFLDREPATRALAAALNLEGRIRAADPAAKRRYVYTRGRLRSVPASPPAFLASDILPLGARLRVAGELFSRRAPEGVDESLAAFGRRHLGHRATQVLLDAVQTGIYAGDVEQLSVAATFPMLVKMEREHRSLILGAIRAQKAQRQAALPAGTAPKLSGALSTFDGGLQVLIDALAASLGDAAHVGARVEGLAREDGGWRLIIEEHGRRAELSVAQVVLAAPAHATAKLLRPLDDALAALVAGIAYAPIAVVHLGFDAGTLPAPDGFGFLVPAEEQRRMLGAIHASTTFPFRAEGGRVLYSCMVGGARQPGLVEQDEDALAALAREELKALAGVTARPSFTRVFRWPLGIPQYNLGHLERVAAIDAALQRLPGLHLIGNAYKGVGLNDCIRNAAQLADALVAGNTSHAP

>Mes515

MADQKQRVTVIGGGLAGTECAYQLSRRGVPVVLREMKPQKRSPAHKSDTLAELVCSNSLRSDNPESAIGLLHAELRALGSLVLSAADANRVPAGDALAVERERFSAAITESLLRQPGVELVAGEVEQLPEDGPVVIATGPLTSDALTRELERHVGTRLYFYDSIAPILSADSIDMNVAFRQSRYGKGGGDDYLNLPMTKDEYYRFIAEVKAGQKVVPHAFEEPKYFEGCLPIEVMAERGDDTLAYGPMKPVGLRDPRTGQEPYAVVQLRMEDVGGTSWNMVGFQTRLTWGEQKRIFSSFIPGLQQAEFLRMGQIHRNTFIDSPRLLAKDLSLKTEPRLYFAGQISGVEGYVESAACGYLVALALHARLTGTEFVPPPATTAMGALLRHVTGEAHPPDYPHQPSNISFGIFSPLTGRMKKAEKRAAYSARAKQDLAAWLPHAGVPAAGAPEHVDQRSA

>Mes516

MISATKIRSCLAACVLAAFGATGALADKATIPSESPFAAAEVADGAIVVDIAKMKYETPELHVKVGDTVTWINREAMPHNVHFVAGVLGEAALKGPMMKKEQAYSLTFTEAGTYDYHCTPHPFMRGKVVVE

>Mes518

MTSKTTASLLAICVACAASAIAGTALCADRRNAPAQAGAGAAAAVSGDAHEQPAAEAPAEEEEETPAVAATDGKLVLPNGQDITPDHMENGRWYTAEDIPTYKIAEEGAVDWATFSGYRRYSAECHVCHGPDGEGSTYAPALRKSVLTMGYYDFLEIAASGKQEVNTAANLVMPAFGTNKNVWCYIDDIYAYLLARGTGDLPRGRPAKREDKSDEFVAQEDSCMSG

>Mes519

MNLLAVRDLAVARGGLRAVEGVCFNLNAGGALVLRGPNGIGKTTLLRTLAGLQPLVSGVIEAAPDAIAYAGHSDGLKPALTVTENLRFWAEIFGGRNIDAALEAMNLRDLANRPAHALSAGQKRRLGLARLMVTGRPVWLLDEPTVSLDRDSVALFAAMLRAHLGRGGAAVIATHIDLGLPEAEILELGPFRASELRRQSRPAGFNEAFG

>Mes521

MADAAVHGHGDHHDTRGFFTRWFMSTNHKDIGILYLFTAGIVGLISVCFTVYMRMELQHPGVQYMCLEGARLIADASAECTPNGHLWNVMITYHGVLMMFFVVIPALFGGFGNYFMPLHIGAPDMAFPRLNNLSYWMYVCGVALGVASLLAPGGNDQMGSGVGWVLYPPLSTTEAGYSMDLAIFAVHVSGASSILGAINIITTFLNMRAPGMTLFKVPLFAWSVFITAWLILLSLPVLAGAITMLLMDRNFGTQFFDPAGGGDPVLYQHILWFFGHPEVYIIILPGFGIISHVISTFAKKPIFGYLPMVLAMAAIGILGFVVWAHHMYTAGMSLTQQAYFMLATMTIAVPTGIKVFSWIATMWGGSIEFKTPMLWAFGFLFLFTVGGVTGVVLSQAPLDRVYHDTYYVVAHFHYVMSLGAVFGIFAGVYYWIGKMSGRQYPEWAGQLHFWMMFIGSNLIFFPQHFLGRQGMPRRYIDYPVEFAYWNNISSIGAYISFASFLFFIGIVFYTLFAGKRVNVPNYWNEHADTLEWTLPSPPPEHTFETLPKREDWDRAHAH

>Mes522

MMAIATKRRGVAAVMSLGVATMTAVPALAQDVLGDLPVIGKPVNGGMNFQPASSPLAHDQQWLDHFVLYIITAVTIFVCLLLLICIVRFNRRANPVPARFTHNTPIEVIWTLVPVLILVAIGAFSLPILFRSQEMPNDPDLVIKAIGHQWYWSYEYPNDGVAFDALMLEKEALADAGYSEDEYLLATDNPVVVPVGKKVLVQVTATDVIHAWTIPAFAVKQDAVPGRIAQLWFSVDQEGVYFGQCSELCGINHAYMPIVVKAVSQEKYEAWLAGAKEEFAADASDYLPASPVKLASAE

>Mes523

MAHVKNHDYQILPPSIWPFFGAIGAFVMLTGAVAWMKGITFFGLPVEGPWMFLIGLVGVLYVMFGWWADVVNEGETGEHTPVVRIGLQYGFILFIMSEVMFFVAWFWAFIKNALYPMGPDSPIKDGVWPPEGIVTFDPWHLPLINTLILLLSGVAVTWAHHAFVLEGDRKTTINGLIVAVILGVCFTGLQAYEYSHAAFGLADTVYAGAFYMATGFHGAHVIIGTIFLFVCLIRLLKGQMTQKQHVGFEAAAWYWHFVDVVWLFLFVVIYIWGR

>Mes524

MASHHEITDHKHGEMDIRHQQATFAGFIKGATWVSILSIAVLVFLALANS

>Mes525

MKISIYATLAAITLALPAAAQDGDAAKGEKEFNKCKACHMIQAPDGTDIIKGGKTGPNLYGVVGRKIASEEGFKYGEGILEVAEKNPDLTWTEADLIEYVTDPKPWLVKMTDDKGAKTKMTFKMGKNQADVVAFLAQNSPDAGGDGEAAAEGESN

>Mes526

MFDTMTVTKAAGALIGSLLFLLLMSWAASGIFHVGTSGHGAEGEEHAQAYTYPVESAGGAEGEAVDEGPDFATVLASADPAAGEKVFGKCKACHKLDGNDGVGPHLNGVVGRTVAGVDGFNYSDPMKAHGGDWTPEALQEFLTNPKAVVKGTKMAFAGLPKIEDRANLIAYLEGQQ

>Mes528

MNRNTPKARGASSLAMAVAMGLAVLTTAPATANDQLVELAKDPANWVMTGRDYNAQNYSEMTDINKENVKQLRPAWSFSTGVLHGHEGTPLVVGDRMFIHTPFPNTTFALDLNEPGKILWQNKPKQNPTARTVACCDVVNRGLAYWPGDDQVKPLIFRTQLDGHIVAMDAETGETRWIMENSDIKVGSTLTIAPYVIKDLVLVGSSGAELGVRGYVTAYDVKSGEMRWRAFATGPDEELLLAEDFNAPNPHYGQKNLGLETWEGDAWKIGGGTNWGWYAYDPEVDLFYYGSGNPAPWNETMRPGDNKWTMAIWGREATTGEAKFAYQKTPHDEWDYAGVNVMMLSEQEDKQGQMRKLLTHPDRNGIVYTLDRTNGDLISADKMDDTVNWVKEVQLDTGLPVRDPEFGTRMDHKARDICPSAMGYHNQGHDSYDPERKVFMLGINHICMDWEPFMLPYRAGQFFVGATLTMYPGPKATAERAGAGQIKAYDAISGEMKWEKMERFSVWGGTMATAGGLTFYVTLDGFIKARDSDTGDLLWKFKLPSGVIGHPMTYKHDGRQYVAIMYGVGGWPGVGLVFDLADPTAGLGSVGAFKRLQEFTQMGGGVMVFSLDGESPYSDPNVGEYAPGEPT

>Mes529

MKRILTLTVAALALGTPALAYDGTNCKAPGNCWEPKPDYPAKVEGSKYDPQHDPAELSKQGESLAVMDARNEWRVWNMKKTGKFEYDVKKIDGYDETKAPPAE

>Mes530

MALPPNFMPLFRASLIGLGLGCSALALAASAQDAPEAETQAQETQGQAAARAAAADLAAGQDDEPRILEAPAPDARRVYVNDPAHFAAVTQQFVIDGEAGRVIGMIDGGFLPNPVVADDGSFIAHASTVFSRIARGERTDYVEVFDPVTLLPTADIELPDAPRFLVGTYPWMTSLTPDGKTLLFYQFSPAPAVGVVDLEGKAFKRMLDVPDCYHIFPTAPDTFFMHCRDGSLAKVAFGTEGTPEITHTEVFHPEDEFLINHPAYSQKAGRLVWPTYTGKIHQIDLSSGDAKFLPAVEALTEAERADGWRPGGWQQVAYHRALDRIYLLVDQRDEWRHKTASRLLVVLDAKTGERLAKFEMGHEIDSINVSQDEKPLLYALSTGDKTLYIHDAESGEELRSVNQLGHGPQVITTADMG

>Mes531

MLGNFRFDDMVEKLSRRVAGQTSRRSVIGKLGTAMLGIGLVPLLPVDRRGRVSRANAADAPAGTDPRAKWVPQDNDIQACDYWRHCSIDGNICDCSGGSLTNCPPGTKLATASWVASCYNPTDGQSYLIAYRDCCGYNVSGRCPCLNTEGELPVYRPEFANDIIWCFGAEDDAMTYHCTISPIVGKAS

>Mes533

MAVLLLGEVTNGALNRDATAKAVAAVKALGDVTVLCAGASAKAAAEEAAKIAGVAKVLVAEDALYGHRLAEPTAALIVGLAGDYSHIAAPATTDAKNVMPRVAALLDVMVLSDVSAILDADTFERPIYAGNAIQVVKSKDAKKVFTIRTASFDAAGEGGTAPVTETAAAADPGLSSWVADEVAESDRPELTSARRVVSGGRGLGSKESFAIIEELADKLGAAVGASRAAVDSGYAPNDWQVGQTGKVVAPELYVAVGISGAIQHLAGMKDSKVIVAINKDEEAPIFQIADYGLVGDLFSVVPELTGKL

>Mes534

MKVLVPVKRLIDYNVKARVKSDGSGVDLANVKMSMNPFDEIAVEEAIRLKEKGQAEEIIAVSIGVKQAAETLRTALAMGADRAILVVAADDVQQDIEPLAVAKILAAVARAEGTELIIAGKQAIDNDMNATGQMLAAILGWAQATFASKVEIEGAKAKVTREVDGGLQTIAVSLPAVVTADLRLNEPRYASLPNIMKAKKKPLDEKTAADYGVDVAPRLEVVSVREPEGRKAGIKVGSVDELVGKLKEAGVI

>Mes535

MRTRAAVALEAGKPLEVMEVNLEGPKAGEVMVEIKATGICHTDEFTLSGADPEGLFPSILGHEGAGVVVEVGPGVTSVKPGNHVIPLYTPECRQCASCLSGKTNLCTAIRATQGQGLMPDGTSRFSMLDGTPIFHYMGCSTFSNYTVLPEIAVAKVREDAPFDKICYIGCGVTTGIGAVINTAKVEIGAKAVVFGLGGIGLNVLQGLRLAGADMIIGVDLNDDKKPMAEHFGMTHFINPKNCENVVQEIVNLTKTPFDQIGGADYSFDCTGNVKVMRDALECTHRGWGQSIIIGVAPAGAEISTRPFQLVTGRVWKGTAFGGARGRTDVPQIVDWYMDGKIEIDPMITHTLSLDDINKGFDLMHAGESIRSVVLY

>Mes536

MVDTSGVKIHPAVDNGIKPAQPGFAGGTLHCKCSTNPVRVAVRAQTAHNHVCGCTKCWKPEGAIFSQVAVVGRDALEVLEGAEKLEIVNAEAPIQRHRCRDCGVHMYGRIENRDHPFYGLDFVHTELSDEDGWSAPEFAAFVSSIIESGVDPSRMEAIRARLRELGLEPYDALSPPLMDAIATHIAKRSGALAA

>Mes537

MAINIILLGPPGAGKGTQARRLIDERGLVQLSTGDMLREARSSGTEMGKRVAEVMDRGELVTDEIVIGLIREKLGQGGKGFIFDGFPRTLAQADALQALMAEMDQRIDAVIEMRVDDAALVSRISGRFTCGNCGEVYHDVTKPTKEPGKCDVCGSTDLRRRADDNEESLKTRLMEYYKKTSPLIGYYYVKGNLNPVDGLAEIDEVAAQVAKVMDKIPA

>Mes538

MESKQEKGLSRRALLGATAGGAAVAGAFGGRLALGPAALGLGTAGVATVAGSGAALAASGDGSVAPGQLDDYYGFWSSGQSGEMRILGIPSMRELMRVPVFNRCSATGWGQTNESVRIHERTMSERTKKFLAANGKRIHDNGDLHHVHMSFTEGKYDGRFLFMNDKANTRVARVRCDVMKCDAILEIPNAKGIHGLRPQKWPRSNYVFCNGEDETPLVNDGTNMEDVANYVNVFTAVDADKWEVAWQVLVSGNLDNCDADYEGKWAFSTSYNSEKGMTLPEMTAAEMDHIVVFNIAEIEKAIAAGDYQELNGVKVVDGRKEASSLFTRYIPIANNPHGCNMAPDKKHLCVAGKLSPTATVLDVTRFDAVFYENADPRSAVVAEPELGLGPLHTAFDGRGNAYTSLFLDSQVVKWNIEDAIRAYAGEKVDPIKDKLDVHYQPGHLKTVMGETLDATNDWLVCLSKFSKDRFLNVGPLKPENDQLIDISGDKMVLVHDGPTFAEPHDAIAVHPSILSDIKSVWDRNDPMWAETRAQAEADGVDIDNWTEEVIRDGNKVRVYMSSVAPSFSIESFTVKEGDEVTVIVTNLDEIDDLTHGFTMGNYGVAMEIGPQMTSSVTFVAANPGVYWYYCQWFCHALHMEMRGRMLVEPKEA

>Mes539

MIGLTHYLVVGAILFVTGIFGIFVNRKNVIVILMSIELMLLAVNINFVAFSTHLGDLAGQVFTMFVLTVAAAEAAIGLAILVVFFRNRGTIAVEDVNVMKG

>Mes541

MLRRLSPIQPDSFEFTPANLEWARAQMTKYPEGRQQSAIIPVLWRAQEQEGWLSRPAIEYCADLLGMPYIRALEVATFYFMFQLQPVGSVAHIQICGTTTCMICGAEDLIRVCKEKIAPEPHALSADGRFSWEEVECLGACTNAPMAQIGKDFYEDLTVEKLAALIDRFAAGEVPVPGPQNGRFSAEALGGPTALADLKGGEAHNASVARALRLGDSIKRIDGTEVPITTPWLATQNGV

>Mes542

MADLRKIKIDDTIIEVDPNMTLIQACEMAGIEVPRFCYHERLSIAGNCRMCLVEVVGGPPKPAASCAMQVKDLRPGPEGAPSEIRTNSPMVKKAREGVMEFLLINHPLDCPICDQGGECDLQDQAMAYGVDFSRYREPKRATEDLNLGPLVETHMTRCISCTRCVRFTTEVAGITQMGQTGRGEDSEITSYLNQTLESNMQGNIIDLCPVGALVSKPYAFTARPWELTKTESIDVMDALGSSIRIDTKGREVMRILPRNHDGVNEEWISDKTRFVWDGLRRQRLDRPYIRENGRLRPASWPEALEAAARAMKGKKIAGLIGDLVPAEAAFSLKQLVEGLGGKVECRVDGARLPAGNRSAYVGTARIEDIDDAEMIQLIGTNPRDEAPVLNARIRKAWSKGAKVGLVGEPVDLTYDYAHVGTDRAALESLSSREISDETKARPSIVIVGQGAIARRDGEAVLAHAMKLAENSNSGLLILHTAAGRVGAMDVGAVTEGGLLAAIDGAEVVYNLGADEVDIDQGPFVIYQGSHGDRGAHRDIILPGACYTEESGLFVNTEGRPQLAMRANFAPGEGKENWAILRALSAELGATQPWDSLAGLRRKLVEAVPHLAQIDQVPQNEWQPLGRFDLGQASFRYAIRDFYLTNPIARSSPLMGELSAMAAARKAPAPLAAE

>Mes543

MDGDIRKNSLDDGSMDALTGEQSIRNFNINFGPQHPAAHGLLRMVLELDGEIVERADPHIGLLHRGTEKLMESRTYLQNLPYLDRLDYVAPMNQEHAWCLAIERLTGTVIPRRASLIRVLYSEIGRILNHLMGVTTGAMDVGALTPPLWGFEAREELMIFYERACGARLHAAYFRPGGVHQDLPPDLLDDIEEWCERFPKLVDDLDTLLTENRIFKQRLVDIGIVTEADALDWGYTGVMVRGSGLAWDLRRSQPYECYDEFDFQIPVGRNGDCYDRYLCRMAEMRESCKIMQQAVQKLRAEPAGDVLARGKLTPPRRAEMKRDMESLIHHFKLYTEGFKVPAGEVYAAVEGPKGEFGVYLVADGTNKPWRAKLRAPGFAHLQSIDWMSRGHMLADVPAIIATLDIVFGEVDR

>Mes544

MSEALSDEALLELAEHIAVRRENDVISTQAVGELTVNATLSGVIGLIEFLRNDPNCRFSTLIDITAVDNPARPARFDVVYHLLSMYQNQRIRVKVQVREDELVPSLIGVFPGANWYEREVFDLFGILFSGHSDLRRILTDYGFRGHPLRKDFPTTGYVEVRWSDIEKRVVYEPVNLVQEYRQFDFLSPWEGAKYVLPGDEKAPEAKK

>Mes545

MNALVGCTTSFDPGWEVDAFGAVSNLCQPMEADLYGCADPCWWPAQVADTLNTYPNWSAGADDVMQDWRKLQSVFPETKGSS

>Mes546

MTLAYETVSENRSFGGIQGVYRHQSQATGTPMTFAIYLPPDARHGKVPVLWYLSGLTCTHENAMTKAGAQEWAAEYGIAVIFPDTSPRGEGVANDETYDLGQGAGFYVDATEAPWAPHFRMWHYVTHELPELVFNNFPLDREAQGITGHSMGGHGALTIAMTFPERYRSVSAFAPIAHPSESDWGRKQFAAYLGDDKAAWKRHDSTILMREKGYPGEVLIDQGASDQFLDLLKPEALAHAMAERRQPGTFRMQQGYDHSYFFVQSFMADHIRWHAERLG

>Mes547

MLGNLKPQAPDKILALMGEFRADPRQGKIDLGVGVYKDATGHTPIMRAVHAAEQRMLETETTKTYAGLSGEPEFQKAMGELILGDGLKSETTATLATVGGTGALRQALELARMANPDLRVFVSDPTWPNHVSIMNFMGLPVQTYRYFDAETRGVDFEGMKADLAAAKKGDMVLLHGCCHNPTGANLTLDQWAEIASILEKTGALPLIDLAYQGFGDGLEEDAAGTRLIASRIPEVLIAASCSKNFGIYRERTGCLLALCADAATRELAQGAMAFLNRQTYSFPPFHGAKIVSTVLTTPELRADWMAELEAVRSGMLRLREQLAGELRDLSGSDRFGFVAEHRGMFSRLGATPEQVKRIKEEFGIYMVGDSRINIAGLNDNTIPILARAIIEVGV

>Mes548

MSHADEHAGDHGATRRDFLYYATAGAGTVAAGAAAWTLVNQMNPSADVQALASIQVDVSGVETGTQLTVKWLGKPVFIRRRTEDEIQAGREVDLGQLIDRSAQNSNKPDAPATDENRTMDEAGEWLVMIGVCTHLGCVPIGDGAGDFGGWFCPCHGSHYDTSGRIRRGPAPQNLHIPVAEFLDDTTIKLG

>Mes549

MTARASQDSAALPLDKETLLTVYRKMRTIRDFEERLHVDFGRGDIPGFVHLYAGEEAAGVGILHHLNDGDRIASTHRGHGHCIAKGVDPVAMMKEIYGKKGGSCNGKGGSMHIADLSKGMMGANGILGAGAPLICGAALAAKFRGKGEVGITFCGDGASNQGTFLESLNLAAVWNLPVIFVIENNGYAESTSRDYGTAVDSYVDRAAGFGIPGVTVDGTDFFAVHEAAGEVIRRAREGGGPSLLECKMVRFYGHFEGDAQTYRAAGELDDIRANKDCLKLFGRAVTQAGVVAREELDTIDREVAALIEHAVQEAKAAPQPGPEDLLTDVYVSY

>Mes550

MARKLSIKLAINEAIDQEMTRDPSVIMLGEDIVGGAGADGEKDAWGGVLGVTKGLYAKHGDRLLDTPLSESAYVGAAIGAAACGMRPIAELMFIDFMGVCFDQIFNQAAKFRYMFGGKAETPVVIRAMVGAGFRAAAQHSQMLTPLFTHIPGLKVVCPSTPYDTKGLLIQAIRDNDPVIFCEHKNLYGLEGEVPEGAYAIPFGEANIVRDGKDVSIVTYGLMVHRALEAAATLAKEGIEAEIVDLRTLSPLDMDTVLESVENTGRLVVVDEASPRCNIATDISAQVAQQAFGALKAGIEMVCPPHTPVPFSPTLEDLYIPSAAQIAAAARKTMKGGKH

>Mes551

MATEISPTIIPIVMPKWGLSMKEGTVNAWLVDEGTEITVGLPILDVETDKIANAVEAPDAGTLRRKVAQAGDVLPVKALLGVLAPAEVSDAQIDDYVAAYETPADDAGEEDAAAAYQFADVDGIRVRYARKGGGAETVLFIHGFGGDLDNWLFNLDPLADAYTVVALDLPGHGQSSPRLAGTTLAQMAGFVARFMDETGIEAAHVVGHSMGGGVAAQLAVDAPQRVLSVALVSPVGFGDAVNSGYTEGFVSAQSRRELKPVVELLFADAGLVSRQMLDDLLRYKRLDGVTEALTALGQGLFGGGRQSEQPGQRLANSGKRVLVVWGGQDQIIPAAHAEAAPPGATVKVFADAGHMSQMEKANDFNALLKKHLGG

>Mes552

MDLRQREHIETVVQATTYLAPPAVLADRIAHDAIIQNSWRRCVHQYGLDPSRMQEARILPQPRLREHQERIDDFARIARHGLQSLYGQVAGLGYVVLLTDAQGVTVDYIGEARSDAALRHAGLYLGAEWSESGAGTCAVGTALATGQALTVHQADHFDATHIPLTCTAAPLFDTHGNLHAILDISALTSPQAKDSQGLALQMVRIYAAHIENANFLRAHRRDWILKLNVAPEFVDVNPEYLLALDEAGRIVGHNHRARLMLEGELGGAPGATVLGQRFETLFDARLEDLGHYVYSRPSEQRLVALTRSGGLLYLSVLPPALRWQAPPAETQVAMPDALAALTGGDAALQLQLQRAARLVDSPINLLIHGETGSGKEFLAKALHLASARRGGPFVAVNCAAIPETLIESELFGHLPNSFSGAGPRGKRGLIQEADGGTLFLDEIGDMPRELQSRLLRVLAEGEVLPVGAARPVPVRLRVISATHHSLEQLVADGRFREDLYYRLNGARFTLPPLRARTDLDWLVRKLLQEGSAEGSEITLSPAARERLHRHRWPGNLRELRNVLEYARAVCADGYIDVPDLPDSLAGPAPSAALPQPGPAQSPAAAPFDPHQLPPEGMLLMQYLRASGWNLSAVARQIGVSRMTLYRRMERYGIQSPNRRDGGPEPTDA

>Mes553

MLYLVRMDVNLPHDMPAAQADDIKAREKAYAQQLQHEGKWQQLYRVVGEYANYSIFDVGSHDELHTLLSGLPLFPYMKIHVTPLAKHPSSIR

>Mes557

MHSTQIPPQQKQKRRLRLTVLAAAASMLAAACVSGDDNNNGNGSNPNTKPANIGTVTINSYNGTTDDLLTAGLGKDGLASATAPLPANPTAPTAAELRRYAIHTNYRAIVDTTASGGYGSLYGPNVDAQGNVTGSDGKVAGVEYLAFSDDGSGQQNVTMLVQIPASFNTSKPCMITATSSGSRGVYGAIATGEWGLKRGCAVAYTDKGTGAAPHDLDTDTVPLIDGTRATRAAAGKNAQFAAPAGATSLADFTAANPHRLAFKHAHSQRNPEKDWGKFTLQAVEFAIWAINDRFGAVSANGTRQRTLDKDRIVVIASSVSNGGGAAVAAAEQDAGGLIDGVAVGEPNLNMPPNTGIVVQRGATPVAASGRTLYDYTTTANLLQHCAARATALTQAPFYTNPATATFFANRCQTLAEKGLVSGANTDEQSASALQALHDAGWEAESDDLHPSLAVFDVAAAISVNYANAYAQASVTDRLCGYSFASTLTDLKPAAIAPAALASMFATGNGVPPQPPVQLINDLDPQHGPYLNLASVSPSTLREDLNYDGANCLRSLLAGSDAAARALQAGQALTLRNGNLRGKPAVIVHGRSDGLLPVNHTSRPYLGLNRQQEGVTSKLSYVEVENAQHFDAFIGLVPGYSNRYVPLHVYLNRALDAVYDNLTAGKALPPSQVLRTTPRGGTLNTPAPALLPSNVPPFAASPAAGNAITVNANAVQVPD

>Mes558

MSVVDPSLIERIIRDDVRAMGAYHVPDSHGLVKLDAMENPYRLPPALRSELAARLGEVALNRYPVPSSEALRAKLKEVMQVPAGMEVLLGNGSDEIISMLALAAARPGAKVMAPVPGFVMYAMSAQFAGLEFVGVPLRADFTLDRGAMLAAMAEHQPAIVYLAYPNNPTGNLFDAADMEAIVRAAQGSVCRSLVVVDEAYQPFAQESWMSRLTDFGNLLVMRTVSKLGLAGIRLGYVAGDPQWLEQLDKVRPPYNVNVLTEATALFALEHVAVLDEQAAQLRAERSRVAEGMAAHGGVTVFPSAANFLLARVPDAAQTFDRLLARKVLIKNVSKMHPLLANCLRVTVSTPEENAQFLEAFAASLQD

>Mes559

MLTQKTKDIVKATAPVLAEHGYDIIKCFYQRMFEAHPELKNVFNMAHQEQGQQQQALARAVYAYAENIEDPNSLMAVLKNIANKHASLGVKPEQYPIVGEHLLAAIKEVLGNAATDDIISAWAQAYGNLADVLMGMESELYERSAEQPGGWKGWRTFVIREKRPESDVITSFILEPADGGPVVNFEPGQYTSVAIDVPALGLQQIRQYSLSDMPNGRSYRISVKREGGGPQPPGYVSNLLHDHVNVGDQVKLAAPYGSFHIDVDAKTPIVLISGGVGLTPMVSMLKVALQAPPRQVVFVHGARNSAVHAMRDRLREAAKTYENLDLFVFYDQPLPEDVQGRDYDYPGLVDVKQIEKSILLPDADYYICGPIPFMRMQHDALKNLGIHEARIHYEVFGPDLFAE

>Mes560

MDSRITTILERYRSDRTRLIDILWDVQHEYGHIPDAVLPQLGAGLKLSPLDIRETASFYHFFLDKPSGKYRIYLCNSVIAKINGYQAVREALERETGIRFGETDPNGMFGLFDTPCIGLSDQEPAMLIDKVVFTRLRPGKITDIIAQLKQGRSPAEIANPAGLPSQDIAYVDAMVESNVRTKGPVFFRGRTDLRSLLDQCLLLKPEQVIETIVDSRLRGRGGAGFSTGLKWRLCRDAESEQKYVICNADEGEPGTFKDRVLLTRAPKKVFVGMVIAAYAIGCRKGIVYLRGEYFYLKDYLERQLQELREDGLLGRAIGGRAGFDFDIRIQMGAGAYICGDESALIESCEGKRGTPRVKPPFPVQQGYLGKPTSVNNVETFAAVSRIMEEGADWFRAMGTPDSAGTRLLSVAGDCSKPGIYEVEWGVTLNEVLAMVGARDARAVQISGPSGECVSVAKDGERKLAYEDLSCNGAFTIFNCKRDLLEIVRDHMQFFVEESCGICVPCRAGNVDLHRKVEWVIAGKACQKDLDDMVSWGALVRRTSRCGLGATSPKPILTTLEKFPEIYQNKLVRHEGPLLPSFDLDTALGGYEKALKDLEEVTR

>Mes561

MSRKLVIDPVTRIEGHGKVVVHLDDDNKVVDAKLHVVEFRGFEKFVQGHPFWEAPMFLQRICGICFVSHHLCGAKALDDMVGVGLKSGIHVTPTAEKMRRLGHYAQMLQSHTTAYFYLIVPEMLFGMDAPPAQRNVLGLIEANPDLVKRVVMLRKWGQEVIKAVFGKKMHGINSVPGGVNNNLSIAERDRFLNGEEGLLSVDQVIDYAQDGLRLFYDFHQKHRAQVDSFADVPALSMCLVGDDDNVDYYHGRLRIIDDDKHIVREFDYHDYLDHFSEAVEEWSYMKFPYLKELGREQGSVRVGPLGRMNVTKSLPTPLAQEALERFHAYTKGRTNNMTLHTNWARAIEILHAAEVVKELLHDPDLQKDQLVLTPPPNAWTGEGVGVVEAPRGTLLHHYRADERGNITFANLVVATTQNNQVMNRTVRSVAEDYLGGHGEITEGMMNAIEVGIRAYDPCLSCATHALGQMPLVVSVFDAAGRLIDERAR

>Mes562

MFQLLAGVRMNSTGRPRAKIILLYALLIAFNIGAWLCALAAFRDHPVLLGTALLAYGLGLRHAVDADHLAAIDNVTRKLMQDGRRPITAGLWFSLGHSSVVVLASVLIAVMATTLQERLDAFHEVGSVIGTLASALFLFAIAAINLVILRSAYRAFRRVRRGGIYVEEDFDLLFGNRGFLARIFRPLFRFITRSWHMYPLGMLFALGFDTATEVALLGISTMEASRGVPIWSILVFPALFTAGMALIDTIDSILMCGAYAWAYAKPVRKLYYNMTITFVSAIVALIVGGIETLGLLADKFMLKGVFWNAVGALNENFCQLGFVIIGIFTVCWVVSIVVYRLRRYDDSEVRA

>Mes563

MSIQITIDGKTLTTEEGRTLVDVAAENGVYIPTLCYLKDKPCLGTCRVCSVKVNGNVAAACTVRVSKGLNVEVNDPELVDMRKALVEFLFAEGNHNCPSCEKSGRCQLQAVGYEVDMMVSRFPYRFPVRVVDHASEKIWLERDRCIFCQRCVEFIRDKASGRKIFSISHRGPESRIEIDAELANAMPPEQVKEAVAICPVGTILEKRVGYDDPIGRRKYEIQSVRARALEGEDK

>Mes564

MRAPHKDEIASHELPATPMDPALAANREGKIKVATIGLCGCWGCTLSFLDMDERLLPLLEKVTLLRSSLTDIKRIPERCAIGFVEGGVSSEENIETLEHFRENCDILISVGACAVWGGVPAMRNVFELKDCLAEAYVNSATAVPGAKAVVPFHPDIPRITTKVYPCHEVVKMDYFIPGCPPDGDAIFKVLDDLVNGRPFDLPSSINRYD

>Mes565

MSERYPIIAITGSSGAGTTSVTRTFENIFCREGVKSVVIEGDSFHRYDRAEMKVKMAEAERTGNMNFSHFGAENNLFGDLESLFRSYAESGTGMRRRYLHSTEEAAPFGQQPGTFTAWEPLPADTDLLFYEGLHGGVVTDEVNVAQYPNLLIGVVPVINLEWIQKLWRDKKQRGYSTEAVTDTILRRMPDYVNYICPQFSRTHVNFQRVPCVDTSNPFISREIPAPDESMVVIRFANPKGIDFQYLLSMIHDSFMSRANTIVVPGGKMELAMQLIFTPFVLRMMERRKRAAL

>Mes567

MKISLTSARQLARDILAAQQVPADIADDVAEHLVESDRCGYISHGLSILPNYRTALDGHSVNPQGRAKCVLDQGTLMVFDGDGGFGQHVGKSVMQAAIERVRQHGHCIVTLRRSHHLGRMGHYGEMAAAAGFVLLSFTNVINRAPVVAPFGGRVARLTTNPLCFAGPMPNGRPPLVVDIATSAIAINKARVLAEKGEPAPEGSIIGADGNPTTDASTMFGEHPGALLPFGGHKGYALGVVAELLAGVLSGGGTIQPDNPRGGVATNNLFAVLLNPALDLGLDWQSAEVEAFVRYLHDTPPAPGVDRVQYPGEYEAANRAQASDTLNINPAIWRNLERLAQSLNVAVPTA

>Mes568

MSAYATQGFNLDDRGRRIVVDPVTRIEGHMRCEVNVDANNVIRNAVSTGTMWRGLEVILKGRDPRDAWAFVERICGVCTGCHALASVRAVENALDIRIPKNAHLIREIMAKTLQVHDHAVHFYHLHALDWVDVMSALKADPKRTSELQQLVSPAHPLSSAGYFRDIQNRLKRFVESGQLGPFMNGYWGSKAYVLPPEANLMAVTHYLEALDLQKEWVKIHTIFGGKNPHPNYLVGGVPCAINLDGIGAASAPVNMERLSFVKARIDEIIEFNKNVYVPDVLAIGTLYKQAGWLYGGGLAATNVLDYGEYPNVAYNKSTDQLPGGAILNGNWDEVFPVDPRDSQQVQEFVSHSWYKYADESVGLHPWDGVTEPNYVLGANTKGTRTRIEQIDESAKYSWIKSPRWRGHAMEVGPLSRYILAYAHARSGNKYAERPKEQLEYSAQMINSAIPKALGLPETQYTLKQLLPSTIGRTLARALESQYCGEMMHSDWHDLVANIRAGDTATANVDKWDPATWPLQAKGVGTVAAPRGALGHWIRIKDGRIENYQCVVPTTWNGSPRDYKGQIGAFEASLMNTPMVNPEQPVEILRTLHSFDPCLACSTHVMSAEGQELTTVKVR

>Mes569

MVETFYEVMRRQGISRRSFLKYCSLTATSLGLGPSFLPQIAHAMETKPRTPVLWLHGLECTCCSESFIRSAHPLAKDVVLSMISLDYDDTLMAAAGHQAEAILEEIMTKYKGNYILAVEGNPPLNQDGMSCIIGGRPFIEQLKYVAKDAKAIISWGSCASWGCVQAAKPNPTQATPVHKVITDKPIIKVPGCPPIAEVMTGVITYMLTFDRIPELDRQGRPKMFYSQRIHDKCYRRPHFDAGQFVEEWDDESARKGFCLYKMGCKGPTTYNACSTTRWNEGTSFPIQSGHGCIGCSEDGFWDKGSFYDRLTGISQFGVEANADKIGGTASVVVGAAVTAHAAASAIKRASKKNETSGSEH

>Mes570

MPIQLECLSHTPLHGYVDPAPEVVAEVERVQAAARDRVRAFDPELVVVFAPDHFNGFFYDVMPPFCIGAAATAIGDFKSLAGKLPVPADLALSLAESVMAADIDVALSHRMQVDHGCADALAALTGSLHRYPVIPVFINSVAPPMATLRRARLLGDAVGRFLSRAGKRVLVVGSGGISHEPPVPELAGASEEVAERLIAGRNPSPESAARQARTVAAAKSFVAGDSHLHPLNPEWDRAFLSLLASGELTAVDGMTNDAITRDGGKSAHEIRTWVAAFGALAAYGPYRASLDFYRAIPEWIAGFATMHAEPAAV

>Mes571

MKISRRDFIKQTAITATASVAGVTLPAGAANFVTDSEVTKLKWSKAPCRFCGTGCGVTVAVKDNKVVATQGDPQAEVNKGLNCVKGYFLSKIMYGQDRLTRPLMRMKNGKYDKNGDFAPVTWDQAFDEMERQFKRVLKEKGPTAVGMFGSGQWTVWEGYAAAKLYKAGFRSNNIDPNARHCMASAAAGFMRTFGMDEPMGCYDDFEAADAFVLWGSNMAEMHPILWTRVTDRRLSHPKTRVVVLSTFTHRCFDLADIGIIFKPQTDLAMLNYIANYIIRNNKVNKDFVNKHTVFKEGVTDIGYGLRPDHPLQKAAKNASDPGAAKVITFDEFAKFVSKYDADYVSKLSAVPKAKLDQLAELYADPNIKVMSLWTMGFNQHTRGTWANNMVYNLHLLTGKIATPGNSPFSLTGQPSACGTAREVGTFSHRLPADMVVTNPKHREEAERIWKLPPGTIPDKPGYDAVLQNRMLKDGKLNAYWVQVNNNMQAAANLMEEGLPGYRNPANFIVVSDAYPTVTALAADLVLPSAMWVEKEGAYGNAERRTQFWHQLVDAPGEARSDLWQLVEFAKRFKVEEVWPPELIAKKPEYKGKTLYDVLYRNGQVDKFPLKDVNAEYHNAEAKAFGFYLQKGLFEEYATFGRGHGHDLAPFDAYHEARGLRWPVVNGKETRWRYREGSDPYVKAGTGFQFYGNPDGKAVIFALPYEPPAESPDKEYPYWLVTGRVLEHWHSGSMTRRVPELYRSFPNAVVFMHPEDAKALGLRRGVEVEVVSRRGRMRSRIETRGRDAPPRGLVFVPWFDASQLINKVTLDATCPISLQTDFKKCAVKIVKV

>Mes572

MKPSRSWASLLAVCAVLLAALAMQAIFFPAPARAQGLVDAMRGPTAIANEPRAPLLYPTENKDIRRTRNYTMQPPTIPHKIDGYQLDKDFNRCMFCHARTRTEETQAIPVSITHYMDRDNNVLADVSPRRYFCTQCHVPQADTKPLIGNNFVDVDTILKRRPGAKGAAK

>Mes575

MATGKGAAASTQEGKSQPFKVTPGPFDPATWLEWSRQWQGTEGNGHAAASGIPGLDALAGVKIAPAQLGDIQQRYMKDFSALWQAMAEGKAEATGPLHDRRFAGDAWRTNLPYRFAAAFYLLNARALTELADAVEADAKTRQRIRFAISQWVDAMSPANFLATNPEAQRLLIESGGESLRAGVRNMMEDLTRGKISQTDESAFEVGRNVAVTEGAVVFENEYFQLLQYKPLTDKVHARPLLMVPPCINKYYILDLQPESSLVRHVVEQGHTVFLVSWRNPDASMAGSTWDDYIEHAAIRAIEVARDISGQDKINVLGFCVGGTIVSTALAVLAARGEHPAASVTLLTTLLDFADTGILDVFVDEGHVQLREATLGGGAGAPCALLRGLELANTFSFLRPNDLVWNYVVDNYLKGNTPVPFDLLFWNGDATNLPGPWYCWYLRHTYLQNELKVPGKLTVCGVPVDLASIDVPTYIYGSREDHIVPWTAAYASTALLANKLRFVLGASGHIAGVINPPAKNKRSHWTNDALPESPQQWLAGAIEHHGSWWPDWTAWLAGQAGAKRAAPANYGNARYRAIEPAPGRYVKAKA

>Mes579

MRITQGTFSFLPELTDEQITKQLEYCLNQGWAVGLEYTDDPHPRNTYWEMFGLPMFDLRDAAGILMEINNARNTFPNHYIRVTAFDSTHTVESVVMSFIVNRPADEPGFRLVRQEEPGRTLRYSIESYAVQARPEGSRY

>Mes580

MSVVANPLHPLFAAGVEDIDLREALGSTEVREIERLMDEKSVLVFRGQPLSQDQQIAFARNFGPLEGGFIKVNQRPSRFKYAELADISNVSLDGKVAQRDAREVVGNFANQLWHSDSSFQQPAARYSMLSAVVVPPSGGDTEFCDMRAAYDALPRDLQSELEGLRAEHYALNSRFLLGDTDYSEAQRNAMPPVNWPLVRTHAGSGRKFLFIGAHASHVEGLPVAEGRMLLAELLEHATQREFVYRHRWNVGDLVMWDNRCVLHRGRRYDISARRELRRATTLDDAVV

>Mes582

MTGDLNEFVAHFWPVRVVFGAGSTERIPAEVKRLGARRALVLCTPDQRDLAQRVLGDLGDLGAGFHDGAVMHVPEASVTRAAQAARDADADLLVAVGGGSTIGLAKALALHHGMRFVALPTTYAGSEMTPIWGLTADGAKRTGRDPRVLPSTVLYDPHHLTSLPPEVTGPSGMNAIAHAVESMYAPDRNPITMLLAEESIRAMAQGLPVAVDSPGDLDARTRTLYAAWLAGTVLGMVSMGLHHKLCHVLGGRFNLPHAPMHAVLLPHVAAFNEVAAPAELGRVAAALGAPGPGGAGAALHALLRFTCTERSLAAIGMPAQGIYDAAEHALADAYANPRQASREDIARLLRAAFTGEMPA

>Mes583

MEFRQLRYFVAAAEEGNVGAAARRLHISQPPVTRQIHALEQHLGVLLFERSARGVQLTPAGAAFLEDARRMLELGRTSVDRSRAASRGEIGQLDIGYLGTAIYQTVPALLHAFTQAVPGATLSLALMPKVRQIEALRAGTIHLGVGRFYPQEPGITVEHLHYERLYIAAGSSIARQLRQDPTLLRLKSESLVLFPKEGRPSFADEVIALMRRAGVEPRVTAIVEDVNAALGLVAAGAGVTLVPASVAAIRRPFVRTMEMADASDKVPVSLTYLTDSRVPVLRAFLDVARRGKGQK

>Mes585

MMFTFGKPLNFQRWLDDHSDLLRPPVGNQQVWQDSDFIVTVVGGPNFRTDFHDDPMEEFFYQFKGNAYLNIMDRGQMDRVELKEGDIFLLPPHLRHSPQRPEAGSRCLVIERQRPKGMLDGFEWYCLSCNGLVYRVDVQLNSIVTDLPPLFDIFYGNVGLRKCPQCGQVHPGKAAIEAVARGDQP

>Mes591

MAMITGGELVVRTLIKAGVEHLFGLHGAHIDTIFQACLDHDVPIIDTRHEAAAGHAAEGYARAGAKLGVAGHGGRGIYQCGHAHCQRLAGSQGRCIPHPGSGALRDDETNTLQAGIDQVAMAAPITKWAHRVMATEHIPRLVMQAIRAALSAPRGPVLLDLPWDILMNQIDEDSVIIPDLVLSAHGARPDPADLDQALALLRKAERPVIVLGSEASRTARKTALSAFVAATGVPVFADYEGLSMLSGLPDAMRGGLVQNLYSFAKADAAPDLVLMLGARFGLNTGHGSGQLIPHSAQVIQVDPDACELGRLQGIALGIVADVGGTIEALAQATAQDAAWPDRGDWCAKVTDLAQERYASIAAKSSSEHALHPFHASQVIAKHVDAGVTVVADGALTYLWLSEVMSRVKPGGFLCHGYLGSMGVGFGTALGAQVADLEAGRRTILVTGDGSVGYSIGEFDTLVRKQLPLIVIIMNNQSWGATLHFQQLAVGPNRVTGTRLENGSYHGVAAAFGADGYHVDSVESFSAALAQALAHNRPACINVAVALDPIPPEELILIGMDPFA

>Mes593

MSQKFDVVVIGAGPGGYVAAIRAAQLGLKTACIEKYIGKEGKVALGGTCLNVGCIPSKALLDSSYKYHEAKEAFKVHGIEAKGVTIDVPAMVARKANIVKNLTGGIATLFKANGVTSFEGHGKLLANKQVEVTGLDGKTQVLEAENVIIASGSRPVEIPPAPLSDDIIVDSTGALEFQAVPKKLGVIGAGVIGLELGSVWARLGAEVTVLEALDKFLPAADEQIAKEALKVLTKQGLNIRLGARVTASEVKKKQVTVTFTDANGEQKETFDKLIVAVGRRPVTTDLLAADSGVTLDERGFIYVDDHCKTSVPGVFAIGDVVRGAMLAHKASEEGVMVAERIAGHKAQMNYDLIPSVIYTHPEIAWVGKTEQTLKAEGVEVNVGTFPFAASGRAMAANDTTGLVKVIADAKTDRVLGVHVIGPSAAELVQQGAIGMEFGTSAEDLGMMVFSHPTLSEALHEAALAVNGHAIHIANRKKR

>Mes597

MTEPLILQPAKPADACVIWLHGLGADRYDFMPVAEALQESLLTTRFVLPQAPTRPVTINGGYEMPSWYDIKAMSPARSISLEELEVSAKMVTDLIEAQKRTGIDASRIFLAGFSQGGAVVFHTAFINWQGPLGGVIALSTYAPTFGDELELSASQQRIPALCLHGQYDDVVQNAMGRSAFEHLKSRGVTVTWQEYPMGHEVLPQEIHDIGAWLAARLG

>Mes599

MQPIRLGLVGYGKIAQDQHVPAINANPAFTLVSVATQGKPCPGVENFQSLGELLENGPPVDAIAFCTPPQGRFALVQQALAAGKHVLVEKPPCATLGKAALWIKREQASAPCSPCIAYAPAIAAARDWLATRTLQSVQIDWKEDVRKWHPGQAWIWQPGLGVFDPGINALSIVTHLLPLPLFVESAELRVPSNCQSPIAASIKMSDPRLLDVRAEFDFDHGHDELWSIQIRCAEGTLRLDNGGALLSIDGVRQTVAEEGEYAAVYRHFQQLIGDKTSDVDVQPLRLVADSFFVGSRVSVEAFYD

>Mes600

MKKKILAATAILLAAIANTGVADNTPFYVGADLSYVNEMESCGATYRDQGKKVDPFQLFADKGADLVRVRLWHNATWTKYSDLKDVSKTLKRAKNAGMKTLLDFHYSDTWTDPEKQFIPKAWAHITDTKELAKALYDYTTDTLASLDQQQLLPNLVQVGNETNIEILQAEDTLVHGIPNWQRNATLLNSGVNAVRDYSKKTGKPIQVVLHIAQPENALWWFKQAKENGVIDYDVIGLSYYPQWSEYSLPQLPDAIAELQNTYHKPVMIVETAYPWTLHNFDQAGNVLGEKAVQPEFPASPRGQLTYLLTLTQLVKSAGGMGVIYWEPAWVSTRCRTLWGKGSHWENASFFDATRKNNALPAFLFFKADYQASAQAE

>Mes601

MNLLSGWVRPLMLGCGLLGAALSAGSIQAAVCEYRVTNEWGSGFTASIRITNNGSSTINGWSVSWNYTDGSRVTSSWNAGLSGANPYSATPVGWNTSIPIGSSVEFGVQGNNGSSRAQVPAVTGAICGGQGSSAPSSVASSSSSSSVVSSTPRSSSSSVSSSVPGTSSSSSSSVLTGAQACNWYGTLTPLCNNTSNGWGYEDGRSCVARTTCSAQPAPYGIVSTSSSTPLSSSSSSRSSVASSSSLSSATSSSASSVSSVPPIDGGCNGYATRYWDCCKPHCGWSANVPSLVSPLQSCSANNTRLSDVSVGSSCDGGGGYMCWDKIPFAVSPTLAYGYAATSSGDVCGRCYQLQFTGSSYNAPGDPGSAALAGKTMIVQATNIGYDVSGGQFDILVPGGGVGAFNACSAQWGVSNAELGAQYGGFLAACKQQLGYNASLSQYKSCVLNRCDSVFGSRGLTQLQQGCTWFAEWFEAADNPSLKYKEVPCPAELTTRSGMNRSILNDIRNTCP

>Mes602

MGHVTSPSKRYPASFKRAGSILGVSIALAAFSNVAAAGCEYVVTNSWGSGFTAAIRITNSTSSVINGWNVSWQYNSNRVTNLWNPNLSGSNPYSASNLSWNGTIQPGQTVEFGFQGVTNSGTVESPTVNGAACTGGTSSSVSSSSVVSSSSSSRSSVSSSSVVSSSSSVVSSSSSSVVSGGGQCNWYGTLYPLCVSTTSGWGYENNRSCISPSTCSAQPAPYGIVGGSSSPSSISSSSVRSSSSSSVVPPSSSSSSSVPSSSCSSVSSSSVVSSSSSSVSVPGTGVFRVNTKGNLTKDGQLLPARCGNWFGLEGRHEPSNDADNPSGAPMELYAGNMWWVNNSQGSGRTIQQTMTELKQQGITMLRLPIAPQTLDANDPQGRSPNLKNHQSIRQSNARQALEDFIKLADQNDIQIFIDIHSCSNYVGWRAGRLDARPPYVDANRVGYDFTREEYSCSATNNPSSVTRIHAYDKQKWLANLREIAGLSAKLGVSNLIGIDVFNEPYDYTWAEWKGMVEEAYQAINEVNPNMLIIVEGISANANTQDGTPDTSVPVPHGSTDLNPNWGENLYEAGANPPNIPKDRLLFSPHTYGPSVFVQRQFMDPAQTECAGLEGDEAAQARCRIVINPTVLEQGWEEHFGYLRELGYGILIGEFGGNMDWPGAKSSQADRNAWSHITTNVDQQWQQAAASYFKRKGINACYWSMNPESADTMGWYLTPWDPVTANDMWGQWTGFDPRKTQLLHNMWGL

>Mes603

MSAFNTTLPSLDYDDDTLREHLQGADIPTLLLTVAHLTGDLQILKPNWKPSIAMGVARSGMDLETEAQVREFCLQRLIDFRDSGQPAPGRPTSDQLHILGTWLMGPVIEPYLPLIAEEAVTAEEDLRAPRWHKDHVASGRDFKVVIIGAGESGMIAALRFKQAGVPFVIYEKGNDVGGTWRENTYPGCRVDINSFWYSFSFARGIWDDCFAPAPQVFAYMQAVAREHGLYEHIRFNTEVSDAHWDESTQRWQLLYRDSEGQTQVDSNVVVFAVGQLNRPMIPAIPGIETFKGPMFHSAQWDHDVDWSGKRVGVIGTGASATQFIPQLAQTAAELKVFARTTNWLLPTPDLHEKISDSCKWLLAHVPHYSLWYRVAMAMPQSVGFLEDVMVDVGYPPTELAVSARNDRLRQDISAWMEPQFADRPDLREVLIPDSPVGGKRIVRDNGTWISTLKRDNVSMIRQPIEVITPKGICCVDGTEHEFDLIVYGTGFHASKFLMPINVTGRDGVALHDVWKGDDARAYLGMTVPQFPNMFCMYGPNTGLVVYSTVIQFSEMTASYIVDAVRLLLEGGHQSMEVKTPVFESYNQRVDEGNALRAWGFSKVNSWYKNSKGRVTQNFPFTAVEFWQRTHSVEPTDYQLG

>Mes604

MTATDNARQVTIIGAGLAGTLVARLLARNGWQVNLFERRPDPRIETGARGRSINLALAERGAHALRLAGLEREVLAEAVMMRGRMVHVPGTPPNLQPYGRDDSEVIWSINRDRLNRILLDGAEAAGASIHFNLGLDSVDFARQRLTLSNVSGERLEKRFHLLIGADGCNSAVRQAMASVVDLGEHLETQPHGYKELQITPEASAQFNLEPNALHIWPHGDYMCIALPNLDRSFTVTLFLHHQSPAAQPASPCFAQLVDGHAARRFFQRQFPDLSPMLDSLEQDFEHHPTGKLATLRLTTWHVGGQAVLLGDAAHPMVPFHGQGMNCALEDAVALAEHLQSAADNASALAAFTAQRQPDALAIQAMALENYVEMSSKVASPTYLLERELGQIMAQRQPTRFIPRYSMVTFSRLPYAQAMARGQIQEQLLKFAVANHSDLTSINLDAVEHEVTRCLPPLSHLC

>Mes605

MTTRNDCLALDAQDSLAPLRQQFALPEGVIYLDGNSLGARPVAALARAQAVIAEEWGNGLIRSWNSAGWRDLSERLGNRLATLIGARDGEVVVTDTTSINLFKVLSAALRVQATRSPERRVIVTETSNFPTDLYIAEGLADMLQQGYTLRLVDSPEELPQAIDQDTAVVMLTHVNYKTGYMHDMQALTALSHECGALAIWDLAHSAGAVPVDLHQAGADYAIGCTYKYLNGGPGSQAFVWVSPQLCDLVPQPLSGWFGHSRQFAMEPRYEPSNGIARYLCGTQPITSLAMVECGLDVFAQTDMASLRRKSLALTDLFIELVEQRCAAHELTLVTPREHAKRGSHVSFEHPEGYAVIQALIDRGVIGDYREPRIMRFGFTPLYTTFTEVWDAVQILGEILDRKTWAQAQFQVRHSVT

>Mes606

MGVFDYKNLGTEASKTLFADATAITLYTYHNLDNGFAVGYQQHGLGLGCRHTGRGVARQHRLPGSDPPAFPGILTRKRPPWTRCTQPVGRQSSASALGYGGKVDARGTFFGEKAGYTTAQAEVLGKYDDAGKLLEIGIGFRGTSGPRESLITTPCRSGQRPARRAGPQGLCEKLCRRTFGGLLKTVADYAGAHGLSGKDVLVSGHSLGGLAVNSMADLSTSKWAGFYKDANYLAYASPTQSAGDKVLNIGYENDPVFRALDGSTFNLSSLGVHDKAHESTTDNIVSFNDHYASTLWNVLPFSIANLSTWVSHLPSAYGDGMTRVLESGFYEQMTRDSTIILCPTWSDPARANTWVQDLNRNAEPHTGNTFIIGSDGNDLIQGGKGADFIEGGKGNDTIRDNSGHNTFLFSGHFGQDRIIGYQPTGWCSRAPTAAPTCATTRRPWGPIRC

>Mes607

MKTITTARLPWAAQSFALGICLIALLGCNHAANKSSASRADVKPVTVKLVDSQATMETRSLFAFMQEQRRHSIMFGHQHETTQGLTITRTDGTQSDTFNAVGDFAAVYGWDTLSIVAPKAEGDIVAQVKKAYARGGIITVSSHFDNPKTDTQKGVWPVGTSWDQTPAVVDSLPGGAYNPVLNGYLDQVAEWANNLKDEQGRLIPVIFRLYHENTGSWFWWGDKQSTPEQYKQLFRYSVEYLRDVKGVRNFLYAYSPNNFWDVTEANYLERYPGDEWVDVLGFDTYGPVADNADWFRNVVANAALVARMAEARGKIPVISEIGIRAPDIEAGLYDNQWYRKLISGLKADPDAREIAFLLVWRNAPQGVPGPNGTQVPHYWVPANRPENINNGTLEDFQAFYADEFTAFNRDIEQVYQRPTLIVK

>Mes608

MATIHVDGKELEVDGADNLLQACLSLGLDIPYFCWHPALGSVGACRQCAVKQYTDENDKRGRIVMSCMTPATDGSWISIDDEEAKVFRASVVEWLMTNHPHDCPVCEEGGHCHLQDMTVMTGHNERRYRFTKRTHQNQDLGPFISHEMNRCIACYRCVRFYKDYAGGTDLGVFGAHDNVYFGRVEDGTLESEFSGNLTEVCPTGVFTDKTHSERYNRKWDMQFSPSICHGCSSGCNISPGERYGELRRIENRFNGSVNQYFLCDRGRFGYGYVNRKDRPRQPLLANGAKLSLDQALDKAAELLRGRNIVGIGSPRASLESNYALRELVGAEHFYSGIEAGELERIRLVLQVLKDSPLPVPNMRDIEDHDAVFVLGEDLTQTAARMALALRQSVKGKAEDMADAMRVQPWLDAAVKNIGQHALNPLFIASLAETKLDDVAEECVHAAPDDLARIGFAVAHALDASAPAVDGLDSEAAALAQRIADALLAAKRPLIIAGTSLGSKALIEAAANIAKALKLREKNGSISLIVPEANSLGLAMLGGESVDAALQAVIDGSADAIVVLENDLYTRTDKAKVDAALNAAKVLIVADHQKTATTDRAHLVLPAASFAEGDGTLVSQEGRAQRFFQVFDPQYLDASILVHEGWRWLHALRATLLDQPIDWTQLDHVTAAVASSSPQLAAIVDAAPSASFRIKGLKLAREPLRYSGRTAMRADISVHEPRTSQDNDTAFSFSMEGYSGSTEPRSQVPFAWSPGWNSPQAWNKFQDEVGGHLRAGDPGTRLIESQGDHLSWFASVPRAFNPAPGTWQVVPFHHLFGSEENSSKAAPVQERIPAAYVSLAKSEADRLGVNDGALLSLNVAGQTLRLPLRINEELGAGLVALPAGLAGIPPAIFGKTVDGLQEAAQ

>Mes609

MFKYIGDIVKGTGTQLRSLVMIFGHGFRKRDTLQYPEEPVYLAPRYRGRIVLTRDPDGEERCVACNLCAVACPVGCISLQKAETEDGRWYPDFFRINFSRCIFCGLCEEACPTTAIQLTPDFEMAEFKRQDLVYEKEDLLISGPGKNPDYNFYRVAGMAIAGKPKGAAQNEAEPINVKSLLP

>Mes610

MPLRTLLCGLLLAVCLGQHALAASRCSERPRTLLRPAEVSCSYQSTWLDSGLVGQRKIIYQTPLGTPPAGGWPVVLIYQGSFFPLNDFSYHSNLPFGGYYEGKLVQNLLDHGYAVIAPSAPADLFWQTNIPGLAQAYELSTDYDFLGNVLAAIASGHFGPLNAQRQYATGISSGGYNTSRMAVSFPGKFRALAVQSGSYATCSGPLCVVPDQLPADHPPTLFLHGFVDAVVPWWSMDLYYDRLLHQGIETARYTEPLGGHEWFAASPGKVLAWFNAHP

>Mes611

MKTQVAIIGAGPSGLLLGQLLHKAGIDNVILERQTPDYVLGRIRAGVLEQGMVDLLREAGVDRRMARDGLVHEGVEIAFAGQRRRIDLKRLSGGKTVTVYGQTEVTRDLMEAREACGATTVYQAAEVRLHDLQGERPYVTFERDGERLRLDCDYIAGCDGFHGISRQSIPAERLKVFERVYPFGWLGLLADTPPVSHELIYANHPRGFALCSQRSATRSRYYVQVPLTEKVEDWSDERFWTELKARLPAEVAEKLVTGPSLEKSIAPLRSFVVEPMQHGRLFLAGDAAHIVPPTGAKGLNLAASDVSTLYRLLLKAYREGRGELLERYSAICLRRIWKAERFSWWMTSVLHRFPDTDAFSQRIQQTELEYYLGSEAGLATIAENYVGLPYEEIE

>Mes612

MHNYVIIDAFASVPLEGNPVAVFFDADDLPPAQMQRIAREMNLSESTFVLKPRNGGDALIRIFTPVNELPFAGHPLLGTAIALGAHTDNHRLYLETQMGTIAFELERQNGSVIAASMDQPIPTWTALGRDAELLKALGISDSTFPIEIYHNGPRHVFVGLPSIDALSALHPDHRALSNFHDMAINCFAGAGRRWRSRMFSPAYGVVEDAATGSAAGPLAIHLARHGQIEFGQPVEILQGVEIGRPSLMFAKAEGRAEQLTRVEVSGNGVTFGRGTIVL

>Mes613

MNGSIQGKPLLGKGMSESLTGTLDAPFPEYQTLPADPMSVLHNWLERARRVGIREPRALALATADSQGRPSTRIVVISEISDAGVVFSTHAGSQKGRELLHNPWASGVLYWRETSQQIILNGQAVRLPNAKADDAWLKRPYATHPMSSVSRQSEELQDVQAMRNAARQLAELQGPLPRPEGYCVFELRLESLEFWGNGQERLHERLRYDRSDTGWNVRRLQP

>Mes614

MKLKNTLGFAIGSIIAATSFGALAQGQGAVEGELFYKKQYNDSVKHIEDGFNPGARIGYFLTDDLSLNLSYDKTNHTRSNDGTGSQKIGGDTSSLTAQYHFGQAGVDSLRPYVEGGFGHQSRGNVKADGHSGRDQSTLAIAGAGVKYYFTNNVYARAGVEADYALDNGKWDYSALVGLGVNFGGNAGAAAPAPTPAPAPEPTPEPEAPVAQVVRVELDVKFDFDKSVVKPNSYGDVKNLADFMAQYPATNVEVAGHTDSIGPDAYNQKLSQRRADRVKQVLVKDGVAPSRITAVGYGESRPVADNATEAGRAVNRRVEASVEAQAQ

>Mes615

MTTFTTRDGTQIYYKDWGSGQPIVFSHGWPLNADSWESQMIFLAAQGYRVIAHDRRGHGRSSQPWSGNDMDTYADDLAQLIEHLDLRDAVLFGFSTGGGEVARYIGRHGTARVAKAGLISAVPPLMLKTEANPGGLPMEVFDGIRQASLADRSQLYKDLASGPFFGFNQPGAKSSAGMVDWFWLQGMAAGHKNAYDCIKAFSETDFTEDLKKIDVPTLVVHGDADQVVPIEASGIASAALVKGSTLKIYSGAPHGLTDTHKDQLNADLLAFIKG

>Mes616

MTPLETKRPLQLNDQGQLQHFLSLDGLRRELLTEILDTADSFLEVGARAVKKVPLLRGKTVCNVFFENSTRTRTTFELAAQRLSADVITLNVSTSSASKGETLLDTLRNLEAMAADMFVVRHGDSGAAHFIAEHVCPQVAIINGGDGRHAHPTQGMLDMLTIRRHKGSFENLSVAIVGDILHSRVARSNMLALKTLGCPDIRVIAPKTLLPIGVEQYGVKVYTDMTEGLKDVDVVIMLRLQRERMTGGLLPSEGEFYRLFGLTTARLAGAKPDAIVMHPGPINRGVEIESAVADGPHSVILNQVTYGIAIRMAVLSMAMSGQTAQRQFDQENAQ

>Mes617

MAVYNYDVVVLGSGPAGEGAAMNAAKAGRKVAMVDSRRQVGGNCTHLGTIPSKALRHSVRQIMQFNTNPMFRAIGEPRWFSFPDVLKSAEKVISKQVASRTGYYARNRVDLFFGTGSFADEQTVEVVCANGVVEKLVAKHIIIATGSRPYRPADIDFHHPRIYDSDTILSLGHTPRKLIIYGAGVIGCEYASIFSGLGVLVELVDNRDQLLSFLDSEISQALSYHFSNNNITVRHNEEYDRVEGLDNGVILHLKSGKKIKADALLWCNGRTGNTDKLGMENIGVKVNSRGQIEVDENYRTCVTNIYGAGDVIGWPSLASAAHDQGRSAAGSIVDNGSWRYVNDVPTGIYTIPEISSIGKNEHELTKAKVPYEVGKAFFKSMARAQIAGEPQGMLKILFHRETLEVLGVHCFGYQASEIVHIGQAIMNQPGEQNTLKYFVNTTFNYPTMAEAYRVAAYDGLNRLF

>Mes618

MSTEGSGPVRFPAMEDAVLERWEKEKTFEQSISAREGKPVYVFYDGPPFATGLPHYGHILTSYIKDVIPRYQTMLGKQVPRRWGWDCHGLPVEFEVEKAMGFKSKRDILEFGVEQFNDECRELVLKYADDWRGFVNRMGRWVDFDGAYKTMDNDYMESVLWGFKTLHDKGHVYEGGKIVPYCVRCQTVLSNFEARLDDAFRPRRDMSAYVKFRQQDRPDTFFLAWTTTPWTLPANVALAVAADENYVCIEHGEERLWLAEGCLGGLFDEPVILERCTGAELAGLRYLPVVGEVIDASAHRVVTADFVQMGDGSGIVHIAPAFGEDDALLGQQYELPAPNPVRDDGTFSDAVAQYAGQNIFEATPRILADLKSSGLLFKQEQIEHNYPHCWRCDNPLIYRAVESWFIRASALREQLVENNSQVNWVPEHVKEGRFGDWIRNARDWAVSRNRFWGAPIPVWRCDQCGTVEVMGSIAQIEARSGRKVEDLHVPHIDEHRFACQCCEGTMSRVTGVFDCWFESGAMPFASRHYPFENKQEFEQTFPADFIVEYLAQTRGWFYTMMVISTGCFEQNPFKNAMCHGVILAKDGRKMSKRLKNYPNPMDLMQTHGSDALRVALLASPVCKGEDIKFSEESVRDVVRRYHLLFWNCLQFYKTFTEIDQFSPSGDLGQPLDNVLDHYLLHELAALESDIKMWMESLDFSKIYSRIEVFINVLSTWYLRLNKARIWRDGLDDDKRQCYEVLHYALSNFARLLAPFMPFLAEAVYTELGYADSVHLQDWPSIDRQYLSYELADEMSSLRNLIASVRNVRETNGVSQKFPLRSIRVAGIEQAVLERYAQFLEEELNVKQVQWAADADEWAQPVVVLIFSLLGKRLGPAMKAVTTAVKAGEYVIDEQGGLVAAGQTIQPHEFERRLTVRDTLNNVGIVENMVVWLDLDIDASLKREGAVRELNRRLQDLRKKAKLGYTEKVDIAVLGGAYVDEILVHHEDWLKSQLLVQSLLRSDLEAPLAVDEVELPEGDPVRIQLRRSVLA

>Mes619

MRTAMAKSLGAAAFLGAALFAHTLAAQTATCSYNITNEWNTGYTGDITITNRGSSAINGWSVNWQYATNRLSSSWNANVSGSNPYSASNLSWNGNIQPGQSVSFGFQVNKNGGSAERPSVGGSICSGSVASSSAPASSVPSSIASSSPSSVASSVISSMASSSPVSSSSVASSTPGSSSGNQQCNWYGTLYPLCVTTTNGWGWEDQRSCIARSTCAAQPAPFGIVGSGSSTPVSSSSSSLSSSSVVSSIRSSSSSSSSSVATGNGLASLADFPIGVAVAASGGNADIFTSSARQNIVRAEFNQITAENIMKMSYMYSGSNFSFTNSDRLVSWAAQNGQTVHGHALVWHPSYQLPNWASDSNANFRQDFARHIDTVAAHFAGQVKSWDVVNEALFDSADDPDGRGSANGYRQSVFYRQFGGPEYIDEAFRRARAADPTAELYYNDFNTEENGAKTTALVNLVQRLLNNGVPIDGVGFQMHVMNDYPSIANIRQAMQKIVALSPTLKIKITELDVRLNNPYDGNSSNDYTNRNDCAVSCAGLDRQKARYKEIVQAYLEVVPPGRRGGITVWGIADPDSWLYTHQNLPDWPLLFNDNLQPKPAYQGVVEALSGR

>Mes620

MTISASDYRHPGNFLKRTTALLCVGTALTALAFNASAACTYTIDSEWSTGFTANITLKNDTGAAINNWNVNWQYSSNRMTSGWNANFSGTNPYNATNMSWNGSIAPGQSISFGLQGEKNGSTAERPTVTGAACNSATTSSVASSSSTPTTSSSSASSVASALLLQEAQAGFCRVDGTIDNNHTGFTGSGFANTNNAQGAAVVWAIDATSSGRRTLTIRYANGGTANRNGSLVINGGSNGNYTVSLPTTGAWTTWQTATIDVDLVQGNNIVQLSATTAEGLPNIDSLSVVGGTVRAGNCGSVSSSSSVQSSSSSSSSSAASAKKFIGNITTSGAVRSDFTRYWNQITPENESKWGSVEGTRNVYNWAPLDRIYAYARQNNIPVKAHTFVWGAQSPSWLNNLSGPEVAVEIEQWIRDYCARYPDTAMIDVVNEAVPGHQPAGYAQRAFGNNWIQRVFQLARQYCPNSILILNDYNNIRWQHNEFIALAKAQGNYIDAVGLQAHELKGMTAAQVKTAIDNIWNQVGKPIYISEYDIGDTNDQVQLQNFQAHFPVFYNHPHVHGITSGICGGQDLDRRLRFDPGQWHTAPGNDVVD

>Mes622

MDTKLLDILACPICKGPLKLSADKTELISKGAGLAYPIRDGIPVMLESEARTLTTEERLDK

>Mes623

MSDIAERVKKIVIDHLGVDAEKVSEGASFIDDLGADSLDTVELVMAFEEEFGVEIPDDAADSILTVGDAVKFIEKAQA

>Mes624

MTTGKPNILIIMVDQLNGKLFPDGPADFLHAPNLKALAKRSARFHNNYTSSPLCAPARASFMAGQLPSRTRVYDNAAEYQSSIPTYAHHLRRAGYYTALSGKMHFVGPDQLHGFEERLTTDIYPADFGWTPDYRKPGERIDWWYHNLGSVTGAGVAEITNQMEYDDEVAFLANQKLYQLSRENDDESRRPWCLTVSFTHPHDPYVARRKFWDLYEDCEHLTPEVGAIPLDEQDPHSQRIMLSCDYQNFDVTEENVRRSRRAYFANISYLDEKVGELIDTLTRTRMLDDTLILFCSDHGDMLGERGLWFKMNFFEGSARVPLMIAGPGIAPGLHLTPTSNLDVTPTLADLAGISLEEVRPWTDGVSLVPMVNGVERTEPVLMEYAAEASYAPLVAIREGKWKYVYCALDPEQLFDLEADPLELTNLAENPRGPVDQATLTAFRDMRAAHWDMEAFDAAVRESQARRWVVYEALRNGAYYPWDHQPLQKASERYMRNHMNLDTLEESKRYPRGE

>Mes625

MRSVTSFNDSWVFSEASTRDAERSGRVSRSACRTNAVELPFNYFDERCYQRAFTYQRVLAWRPDFSQGSRSSSTRQWPMRSCISTAKRSSRIRDGYTPFEARLTDRLLEGDNLITVKIDGSENPEIPPFGAGIDYLTYAGIYRDVWLKVTDPVSIANIKIETRDVLSDHKAVSLRCDLSNPQGLSFSGTISALLKNAAGEVLAEVAGETTGQSLAFEMDGLRGLSLWDIDDPVLYVIEVELRTGQGFRLLRRAFRLPHGEFTTEGFRLNGRPLKIRGLNRHQSFPYVGLRMGRTAKGSAHADIMNAHRLHCNLVRTSHYPQSKWFLDHCDRIGLLVFARNPRLAAYRWGGMETGGNPERPPHRSSATGTTRLSYIWGVRINESQDSHDFYAETNRLARELDPTRQTGGVRYITDSEFLEDVYTMNDFILGNEELPGANRPGTALRPQQECTGLPRKVPYLITEFGGHMYPTKIYDQEQRQAEHVRRHLEVLNAAYARNPGISGAIGWCMFDYNTTRISAPATGSAITASWTCSASPKFAAYVYASQCDPSEEIVMKPVTFWARGDDDIGGVLPLIVLTNCDEIELKYGSLTKRVGPDRENFPHLPHPPVVIDHRHFTKDELGVWGMKWESAEFTGFIAGKPVADLRMAADPVPTTLQVEADSKTLRREGRDTVRLILRALDQAGNVLPFLNDAVDIEIHGPARLVGPARIVLQGGSGFLAGVHGRRRHASSRSRRRGSAAAKLDLVALADGAASA

>Mes626

MQIENRLNAAAASGDGLGNLAGRSADPTGAADKGESGVPVPPTGFVDPTPRISLSADALLYLGRAKRTPEKLPPLTKDEWNNRLSPQLAAREHQAFGRLAETGDYRAYYRAFIDYYDGLRPEDQNSLRYFGTREAAVAGLRSLDYDADSGLDMDAEFENLVSVFLEEDKIAPSPATTTMSPAERAFFAWDASNISYEVDAPEPRPMTEIERLYSELL

>Mes627

MAKKPSAPNNTKPATIHDQKATRGNGGELHQIAEGDTPVLTTAQGGPVADDQNSLRAGERGPTLIEDFHFREKIFHFDHERIPERVVHARGYGVHGFFETYESLAAYTRADLFQRPGERTPAFVRFSTVAGSKGSFDLARDVRGFAVKIYTKEGNWDLVGNNIPVFFIQDAIKFPDVIHSVKPEPDREFPQAQSAHDNFWDFISLTPESMHMIMWVMSDRAIPRSFRFMEGFGVHTFRFVNAKDESTFVKFHWKPKLGLQSVVWNEAVKINGADPDFHRRDMWQAIQSGNFPEWDLHVQLFDQDFADKFDFDILDPTKIIPEEVLPTKPVGRLVLDRMPENFFAETEQVAFMTQNVPPGIDFSDDPLLQGRNFSYLDTQLKRLGSPNFTHLPINAPKCPFQHFQQDGHMAMRNPVGRVNYQPNSWGEGPRESPMKGFRHFPSEEQGPKLRIRAESFADHYSQARQFFISQTPPEQRHIADALTFELSKVETPVIRERMVAHLLNIDETLGKKVGHALGLETMPKPADAAVATRQDLDPSPALSIIQRGPKRFEGRKLGILATDGADGALLDALIAAVEKEKAAFELIAPKVGGFTASDGKRIAAHQMLDGGPSVLYDAVVLLPSAEAVTDLIDVATARDFVADAFAHCKYIGYAGAAVPLLERAGIAELLDEGTIELTDAASAAAFLTEIGKLRVWGREPSVKLK

>Mes633

MADNGQADRKERSNGVIVGTCLAFVAGMIGMAYAAVPLYDMFCRVTGYNGTTQRVEQASDLILDEKIKVTFDANVAAGLPWEFVPVQRDIDVRIGETVQIMYRAKNLASTPTTGQATFNVTPMAAGAYFNKVQCFCFTETTLEPGEEMEMPVVFFVDPEIVKPVETQGIKTLTLSYTFYPREPSKPVAQVKAKAENKL

>Mes634

MNSGTVKWFNSTKGFGFIQPDDGATDVFVHASAVERAGMRSLVEGQKVTYDIVRDTKSGKSSADNLRAA

>Mes636

MIIEHSAEVRGKTPLYRHLYVQVLAAIAAGILLGHFYPDIGTELKPLGDAFIRLVKMIIAPVIFLTVATGIAGMTDLAKVGRVAGKAMIYFLAFSTLALVVGLVVANVVQPGAGMHIDPASLDAKAVATYAEKAHEQSITGFLMNIIPTTLVGAFAEGDILQVLFISVLFGISLAIVGKKAEPVVDFLQALTLPIFRLVAILMKAAPIGAFGAMAFTIGKYGIASIANLAMLIGTFYLTSFLFVFIVLGAVARYNGFSILSLIRYIKEELLLVLGTSSSEAALPGLMNKMEKAGCKRSVVGLVIPTGYSFNLDGTNIYMTLAALFIAQATDTPLSYGDQILLLLVAMLSSKGAAGITGAGFITLAATLSVVPSVPVAGMALILGIDRFMSECRALTNFVGNAVATIVVAKWEGELDQAQLSAALGGEASVEAIPAVVQPAE

>Mes637

MHHVRMVKLPAEASDPHALRSRARRSWLVFAAVALVLLAAGLLLARDYGRSQALAGLAGQSRIDASLKASLLRAVVERQRALPLVLADDAAIRGALLSPDRPSLDRINRKLEALATSAEAAVIYLIDRSGVAVAASNWQEPTSFVGNDYAFRDYFRLAVRDGMAEHFAMGTVSNRPGLYISRRVDGPGGPLGVIVAKLEFDGVEADWQASGKPAYVTDRRGIVLITSLPSWRFMTTKPIAEDRLAPIRESLQFGDAPLLPLPFRKIEARPDGSSTLDALLPGDSTAAFLRVETMVPSTNWRLEQLSPLKAPLAAGAREAQLLTLAALVPLLALAALLLRRRQVVAMRSAEERLARNALEASVEERTRDLRMARDRLETEIADHRQTTEKLQAVQQDLVQANRLAILGQVAAGVAHEINQPVATIRAYADNARTFLHRGQTVTAAENMESIAELTERVGAITDELRRFARKGHFAAGPTAMKEVVEGALMLLRSRFAGRMDAIRLDLPPDGLQALGNRIRLEQVLINLLQNALEAIGDSEDGAIQVRCEEAAGGIALTVADNGPGIAADVREELFTPFNTSKEDGLGLGLAISKEIVSDYGGTIEVESGPSGTTFAVNLKKA

>Mes638

MSAAPSVFLIDDDRDLRKAMQQTLELAGFTVSSFASATEALAGLSADFAGIVISDIRMPGMDGLALFRKILALDPDLPMILVTGHGDIPMAVQAIQDGAYDFIAKPFAADRLVQSARRAEEKRRLVMENRSLRRAAEAASEGLPLIGQTPVMERLRQTLKHIADTDVDVLVAGETGSGKEVVATLLHQWSRRRTGNFVALNCGALPETVIESELFGHEPGAFTGAVKKRIGRIEHASGGTLFLDEIEAMPPATQVKMLRVLEAREITPLGTNLTRPVDIRVVAAAKVDLGDPAARGDFREDLYYRLNVVTLSIPPLRERRDDIPLLFSHFLARASERFGREVPAISAAMRAYLATHSWPGNVRELSHFAERVALGVEGNLGVPAAAPASSGATLPERLERYEADILKQALTAHCGDVKETLQALGIPRKTFYDKLQRHGINRADYVERAGPGRPNAISKT

>Mes640

MTIDRYRRFARLAFIATLPLAGLATAAAAQEGANGKSFKDDFDTLDTRVWFVSDGWNNGGHQNCTWSKKQVKTVDGILELTFEEKKVKERNFACGEIQTRKRFGYGTYEARIKAADGSGLNSAFFTYIGPADKKPHDEIDFEVLGKNTAKVQINQYVSAKGGNEFLADVPGGANQGFNDYAFVWEKNRIRYYVNGELVHEVTDPAKIPVNAQKIFFSLWGTDTLTDWMGTFSYKEPTKLQVDRVAFTAAGDECQFAESVACQLERAQSE

>Mes641

MSKTVLNAVGTPLYYSGSSTAWFSATGSGPTLHGTAGNDSMWGDSSVNVTMIGGRGDDIYYLYSSINRAYEAAGEGVDTISTWMSYTLPANFENLTVTGSGRFAFGNEADNIIKGGSGTQTIDGRGGNDVLIGAGGADTFVFARGNGSDLITDFNYDDIVRLDGYGFTSFEQILSNVAQEGADLRLHLADGESLVFANTTADELQAHQFRLSLDRSVLSQTFSDEFNTLQLRNGTSGVWDAKFWWAPEKGATLSSNGEQQWYINPSYEPTASVNPFSVNNGVLTITAAPASEAIQAEINGYDYTSGMLTTYSSFAQTYGYFEMRADMPDDQGVWPAFWLLPADGSWPPELDVVEMRGQDSNTVIATVHSNETGSRTSIENSVKVADASGFHTYGVLWTEEEIVWYFDDAAIARADTPSDMHDPMYMLVNLAVGGIAGTPRDGLADGSEMKIDYIKAYSLDADWQI

>Mes642

MTDYTVHIVDDEEPVRKSLAFMLTMNGFAVKMHQSAEAFLAFAPDVRNGVLVTDLRMPDMSGVELLRNLGDLKINIPSIVITGHGDVPMAVEAMKAGAVDFIEKPFEDTVIIEAIERASEHLVAAEADVDDANDIRARLQTLSERERQVLSAVVAGLPNKSIAYDLDISPRTVEVHRANVMAKMKAKSLPHLVRMALAGGFGPS

>Mes645

MLDGKTIIVVAADQGLRRSVAFALEVEGYYTESYDSVQKSEASCREALCAIVDDDILRTEPQAAAQFLSNRGGRAILLVDGLSALQPPVDYATLTKPFTGADLLGVINSLVVAAK

>Mes646

MLEKAEKQRRAGSGQQRAAGYMPGFGNDFETESLPGALPQGQNSPQKCNYGLYAEQLSGSPFTAPRGTNERSWLYRIRPSVRHTGRFRRVDYPHWKTAPHVGEHSLALGQLRWSPLPAPSEALDFLQGIRTMTTAGDALTQAGMAAHAYAFNADMVDDYFFNADGELLIVPETGAIQVFTELGRMDVEPSEICLIPRGMMFKVTRLGEEKVWRGYICENYGAKFTLPDRGPIGANCLANPRDFKTPVAAYEDKETPCRVQVKWCGSFHMVEIGHSPLDVVAWHGNYAPYKYDLKTFSPVGAILFDHPDPSIFTVLTAPSGEEGTANVDFVIFPPRWLVAEHTFRPPWYHRNIMSEFMGLIYGRYDAKEEGFVPGGMSLHNMMLAHGPDFSGFEKASNGELKPVKLDNTMAFMFETRFPQQLTTFAAELDTLQDDYMDCWSGLERKFDGTPGIK

>Mes647

MTVILRPGSVPLSDLETIYWTGAPARLDAAFDAGIAKAAARIAEIVAGNAPVYGINTGFGKLASIKIDSSDVATLQRNLILSHCCGVGQPLTEDIVRLIMALKLISLGRGASGVRLELVRLIEAMLDKGVIPLIPEKGSVGASGDLAPLAHMAAVMMGHGEAFFAGERMKGDAALKAAGLSPVTLAAKEGLALINGTQVSTALALAGLFRAHRAGQAALITGALSTDAAMGSSAPFHPDIHTLRGHKGQIDTAAALRQLLTGSPIRQSHIEGDERVQDPYCIRCQPQVDGACLDLLRSVAATLTIEANAVTDNPLVLSDNSVVSGGNFHAEPVAFAADQIALAVCEIGAISQRRIALLVDPALSYGLPAFLAKKPGLNSGLMIAEVTSAALMSENKQLSHPASVDSTPTSANQEDHVSMACHGARRLLQMTENLFSIIGIEALAAVQGIEFRAPLTTSPELQKAAAAVRGVSSSIEEDRYMADDLKAAGDLVASGRLAAAVSAGILPKLEN

>Mes648

MNTGDKAKSQAVPASGDIDQQALFFHRYPRPGKLEIQPTKPLGNQRDLALAYSPGVAAPCLAIKDNPETAADFTARANLVAVVSNGTAVLGLGNIGPLASKPVMEGKAVLFKKFAGIDVFDIEIDAPTVDRMVDVISALEPTFGGINLEDIKAPECFEVERRLREKMEIPVFHDDQHGTAIIVAAAVLNGLELAGKDIAEAKIVASGAGAAALACLNLLVTLGARRENIWVHDIEGLVYKGREALMDEWKAVYAQESDNRVLADSIGGADVFLGLSAAGVLKPELLARMAEKPLIMALANPTPEIMPEVARAARPDAMICTGRSDFPNQVNNVLCFPHIFRGALDCGARTINEEMKMAAVRAIAGLAREEPSDVAARAYSGETPVFGPDYLIPSPFDQRLILRIAPAVAKAAAESGVATRPIQDFDAYLDKLNRFVFRSGFIMKPVFAAAKNAAKNRVIFAEGEDERVLRAAQVLLEEGTAKPILIGRPQIIETRLRRYGLRIRPDVDFEVVNPEGDPRYRDYVDDYFALVGRLGVIPEAARTIVRTNTTVIGALAVKRGEADALICGVEGRYSRHLRDVSQIIGKRSGVLDFSALSLLISQRGATFFTDTYVSFSPSAEEIAQTTVMAANEIRRFGITPRAALVSHSNFGSRDSESAFKMRTALQLVRELAPDLEVDGEMHGDSAISEVLRQRVMPDSTLNGEANLLVFPNLDAANITLGVVKTMTDSLHVGPILLGSALPAHILSPSVTSRGVVNMAALAVVESSHPV

>Mes650

MTVRFGLLGAGRIGKVHAKAVSGNADARLVAVADAFPAAAEAIAGAYGCEVRTIDAIEAAADIDAVVICTPTDTHADLIERFARAGKAIFCEKPIDLDAERVRACLKVVSDTKAKLMVGFNRRFDPHFMAVRKAIDDGRIGEVEMVTITSRDPSAPPVDYIKRSGGIFRDMTIHDFDMARFLLGEEPVSVTATAAVLIDKAIGDAGDYDSVSVILQTASGKQAIISNSRRATYGYDQRIEVHGSKGAVAAENQRPVSIEIATGDGYTRPPLHDFFMTRYTEAYANEIESFIAAIEKGAEIAPSGNDGLAALALADAAVRSVAEKRQISIA

>Mes651

MFEGSITALVTPFADDRIDEVALHDLVEWQIEEGSFGLVPCGTTGESPTLSKSEHEQVVEITIKTANGRVPVIAGAGSNSTAEAIAFVRHAQNAGADGVLIVSPYYNKPTQEGIYQHFKAIDAASTIPIIVYNIPGRSAIEIHVETLARIFEDCPNVKGVKDATGNLLRPSLERMACGEDFNLLTGEDGTALGYMAHGGHGCISVTANVAPALCADFQQACLNGDFAAALKLQDRLMPLHRALFLETNPAGAKYALQRLGRMRGDLRLPLVTISPSFQEEIDDAMRHAGDPFMMDNARFAERIEMDLIGANNQRRKQGGTCMGLDSGEAPCTS

>Mes653

MSLKVQWKLCWENQLERADHQELSEFFRKSYGPTGAFHAKPFEGGRSWAGARPERRAIAYDSVGIASHMGVLRRFIKVGETDLLVAELGLYAVRPDLERMGIAHSVGALTPTLRELGVPFAFGTVRHAMRNHVERYCQNGMASILTGVRVRSSIAEVNADLPSTRTEDPLVVIFPVGRPLNEWPPGTLIERNGSEL

>Mes654

MCGIVGIVGHQPVSERLVEALEPLEYRGYDSAGVATMDAGTLQRRRAEGKLGNLREKLKEAPLSGTIGIAHTRWATHGAPTERNAHPHFTEGVAVVHNGIIENFAELKDELAAGGAEFQTETDTEVVAHLLAKYRRDGLGRREAMHAMLKRVKGAYALAVLFEDDPSTIMAARTGPLAIGHGNGEMFLGSDAIALAPFTNEITYLIDGDWAVIGKTGVHIFDFDGNVVERPRQISTAAAFLVDKGNHRHFMEKEIYEQPEVIAIALGHYVNVIDKSCRSDSDAIDFAGVESLAISCCGTAYLAGLIGKYWFERYARLPVEIAVASEFRYREIPLSPQSALFISQSGETADTLASLRYCKAHGLRIGAVVNARESTMARESDAVFPILAGPEIGVARTKAFTCQLAVLAALRAGAGKARGTISGDEEQALIKSLAEMPAIMGQVLNSIQPEIEVLSRELSNCRDVLYLGRGTSFPLAMEGALKLKEISYIQPKSYAAGQLKHGPYALIDENMPVIVIAPHDRFFDKTVTNMQEVARGGRIILITDEKGAAASKLDTMHTIVLPEVDEIIAPMIFSLPLQLLAYHTAVFMGTDVDQPRNLAKSVTVE

>Mes655

MTGATILVADDDAAIRTVLNQALSRAGYDVRITSNAATLWRWIAAGDGDLVVTDVVMPDENAFDLLPRIKKARPDLPVLVMSAQNTFMTAIKASEKGAYDYLPKPFDLTELIGIIGRALAEPKRRPSKLEDDSQDGMPLVGRSAAMQEIYRVLARLMQTDLTLMITGESGTGKELVARALHDYGKRRNGPFVAINMAAIPRDLIESELFGHEKGAFTGAQTRSTGRFEQAEGGTLFLDEIGDMPMDAQTRLLRVLQQGEYTTVGGRTPIRSDVRIVAATNKDLKQSINQGLFREDLYYRLNVVPLRLPPLRDRAEDIPDLVRHFVQQAEKEGLDVKRFDQEALELMKAHPWPGNVRELENLVRRLTALYPQDVITREIIENELRSEIPDSPIEKAAARSGSLSISQAVEENMRQYFASFGDALPPSGLYDRVLAEMEYPLILAALTATRGNQIKAADLLGLNRNTLRKKIRELGVSVYRSSRSA

>Mes656

MKFFNYRRVPYAEIRAFSVHILTASGSFLAFLGVVAAAEHRFVDMFWWLGLALLVDGIDGPIARKVQVKEVLPNWSGDTLDNVIDYVTYVLLPAFALYQSGMIGEPWSFVAAGAIVVSSAIYYADMGMKTDEYFFSGFPVVWNMVVFTLFVIQASEVTASIVVFLSVILTFLPINFLHPVRVKRLRPLNLGIFLVWSVLGMYALLLHFETPPWVVVGVVATGLYLYVIGFILQIFPKLGRA

>Mes657

MIQKNWQELIKPNKVEFASSGRTKATLVAEPLERGFGLTLGNALRRVLLSSLRGAAVTAVQIDGVLHEFSSIPGVREDVTDIVLNIKEIAIKMDGDDAKRMVVRKQGPGVVTAGDIQTVGDIEILNPNHVICTLDEGAEIRMEFTVNNGKGYVPADRNRSEDAPIGLIPVDSLYSPVKKVSYKVENTREGQVLDYDKLTMSIETDGSVTGEDAIAFAARILQDQLSVFVNFDEPQKETEEEAVTELAFNPALLKKVDELELSVRSANCLKNDNIVYIGDLIQKTEAEMLRTPNFGRKSLNEIKEVLASMGLHLGMEVPSWPPENIEDLAKRYEDQY

>Mes659

MPNDPLLQPYQLKHLTLRNRIIVTAHEPAYPEDGMPKERYRAYTVERARGGVAMTMTAGSAAVSKDSPPVFNNLLAYRDEIVPWIREMTDAVHEEGAVIMIQLTHLGRRTRWDKGDWLPVVAPSHHREAAHRAFPKKIEDWDIDRIIKDFADAAERMKAGGMDGVELEAYGHLIDQFASPLTNELDGPYGGSLDNRMRFCFDVLKAIRARVGDEFILGVRYTADECLPGGTDKAEGLEISKRLKESGLIDYLNIIRGHIDTDPGLTDVIPIQGMANSPHLDFAGEIRAATNFPTFHAAKIPDVATARHAIASGKVDMVGMTRAHMTDPHIVRKIIEKREEDIRPCVGANYCLDRIYQGGAAYCIHNAATGRELTMPHSIAKAHCRRKVVVVGTGPAGLEAARVAGERGHEVIVFEAASDPGGQVRLTAQSPRRREMISIIDWRMSQCEKLGVTFHFNTWAEAEAIQAESPDVVIIATGGLPHTEVLSRGNELVVSAWDIISGDAKPGTNVLIFDDAGDHAALQAAEFLATAGARVEIMTPDRSFAPEVMAMNLVPYMRCLQKLDVTFTVTYRLEAVEKSGNELVAHVGSDYGGISKQRTFDQVVVNHGTIPLDELYFELKPFSSNLGEIAHDQMIAGEPQSVVRNAEGKFQLFRIGDAVAARNTHAAIYDALRLLKDI

>Mes660

MKKTLLASSLIACLSIASVNVYAASESSISIGYAQSHVKENGYTLDNDPKGFNLKYRYELDDNWGVIGSFAYTHQGYDFFYGSNKFGHGDVDYYSVTMGPSFRINEYVSLYGLLGAAHGKVKASVFDESISASKTSMAYGAGVQFNPLPNFVIDASYEYSKLDSIKVGTWMLGAGYRF

>Mes662

MNQTISSRAPQKRLAPRLLCVMIGAALGTLSASSWAAAATDSTAENAKKTSATAATAKAEDSKTNDTITVVGAQETFRAGGNDLIPTYLDGQVANGGRIGFLGQQDARNVPFNVIGYTSKMIEDQQANSIADVVKNDASVQNVRGYGNPSQNYRIRGYNLDGDDISFGGLFGVLPRQIVSTSMVERVEVFKGANAFINGISPSGSGVGGMINLEPKRAGDTPLTRVTVDYGSASQVGGALDVGRRYGDDDQFGVRVNVLHREGESAIHDQKERTTAVSTGLDYRGDRARTSLDVGYQKQTIHHMRTDVAIGGATVIPEPPSSTLNYGQSWVYTDMETTFGMLRSEYDVSQNWTVYGSVGASRNEETGQYGAPMLTNNNGDATISRLYVPYVADSVAGLGGIRGHFDTGPITHKVNLGYAANYRTTKSAWNMSGQEDTNIYNPGVIGFPQTVMGSDSQDPQLTSQVRASGLSLSDTLSMMDDKVSLMLGVRRQEVTIRNFDSGVPNSAGSLDAMKVTPIYGIMVKPWEKVSLYANHIEALGPGKSAPYQYNGKPVVNAGQIPGIIHSKQNEIGVKFDNQRYGGTLALFEITRPTGMVDPATNVYGFYGEQRNRGIELNVFGEPVFGTRLLASATWLDPKLTKAADSANNGNDAVGVANYQLVFGGEYDIPVVEGLTATGTVVRSGSQYANEANTLKLKPWTRLDLGVRYTMPMKDTSLTWRANIENVTNERYWESVEDSGTYIYQGDPRALKLSVSMDF

>Mes663

MKMTRLYPLALGGLLLPAIANAQTSQQDESTLEVTASKQSSRSASANNVSSTVVSAPELSDAGVTASDKLPRVLPGLNIENSGNMLFSTISLRGVSSAQDFYNPAVTLYVDGVPQLSTNTIQALTDVQSVELLRGPQGTLYGKSAQGGIINIVTQQPDSTPRGYIEGGVSSRDSYRSKFNLSGPIQDGLLYGSVTLLRQVDDGDMINPATGSDDLGGTRASIGNVKLRLAPDDQPWEMGFAASRECTRATQDAYVGWNDIKGRKLSLSDGSPDPYMRRCTDSQTLSGKYTTDDWVFNLISAWQQQHYSRTFPSGSLIVNMPQRWNQDVQELRAATLGDARTVDMVFGLYRQNTREKLNSAYNMPTMPYLSSTGYTTAETLAAYSDLTWHLTDRFDIGGGVRFSHDKSSTQYHGSMLGNPFGDQGKSNDDQVLGQLSAGYMLTDDWRVYTRIAQGYKPSGYNIVPTAGLDAKPFVAEKSINYELGTRYETADVTLQAATFYTHTKDMQLYSGPVGMQTLSNAGKADATGVELEAKWRFAPGWSWDINGNVIRSEFTNDSELYHGNRVPFVPRYGAGSSVNGVIDTRYGALMPRLAVNLVGPHYFDGDNQLRQGTYATLDSSLGWQATERINISVHVDNLFDRRYRTYGYMNGSSAVAQVNMGRTVGINTRIDFF

>Mes664

MPRSTSDRFRWSPLSLAIACTLSLAVQAADTSSTQTNSKKRIADTMVVTATGNERSSFEAPMMVTVVEADTPTSETATSATDMLRNIPGLTVTGSGRVNGQDVTLRGYGKQGVLTLVDGIRQGTDTGHLNSTFLDPALVKRVEIVRGPSALLYGSGALGGVISYETVDAADLLLPGQNSGYRVYSAAATGDHSFGLGASAFGRTDDVDGILSFGTRDIGNIRQSDGFNAPNDETISNVLAKGTWRIDQIQSLSANLRYYNNSALEPKNPQTSAASSTNLMTDRSTIQRDAQLKYNIKPLDQEWLNATAQVYYSEVEINARPQGTPEEGRKQTTKGGKLENRTRLFTDSFASHLLTYGTEAYKQEQTPSGATESFPQADIRFGSGWLQDEITLRDLPVSILAGTRYDNYRGSSEGYADVDADKWSSRGAVSVTPTDWLMLFGSYAQAFRAPTMGEMYNDSKHFSMNIMGNTLTNYWVPNPNLKPETNETQEYGFGLRFNDLMMAEDDLQFKASYFDTNAKDYISTGVTMDFGFGPGGLYCKNCSTYSTNIDRAKIWGWDATMTYQTQWFNLGLAYNRTRGKNQNTNEWLDTINPDTVTSTLDVPVANSGFAVGWIGTFADRSSRVSSSGTPQAGYGVNDFYVSYKGQEQFKGMTTTVVLGNAFDKGYYGPQGVPQDGRNAKFFVSYQW

>Mes665

MSKSIYEQYLQAKADNPGKYARDLATLMGISEAELTHSRVSHDAKRLKGDARALLAALEAVGEVKAITRNTYAVHEQMGRYENQHLNGHAGLILNPRNLDLRLFLNQWASAFTLTEETRHGVRHSIQFFDHQGDALHKVYVTEQTDMPAWEALLAQFITTEIPELQLEPLSAPEVTEPTATDEAVDAEWRAMTDVHQFFQLLKRNNLTRQQAFRAVGNDLAYQVDNSSLTQLLNIAQQEQNEIMIFVGNRGCVQIFTGMIEKVTPHQDWINVFNQRFTLHLIETTIAESWITRKPTKDGFVTSLELFAADGTQIAQLYGQRTEGQPEQTQWRDEIARLNNKDIAA

>Mes666

MVDTAVVDTALLEANQLSYHVQGQKLINNVSLQIASGEMVAIIGPNGAGKSTLLRLLTGYLAPSEGHCQLLGKNLNSWQPQALARTRAVMRQYSDLAFPFSVSEVIQMGRAPYGAAQNRQALQEVMAQTDCLALAQRDYRALSGGEQQRVQLARVLAQLWQPEPTSRWLFLDEPTSALDLYHQQHTLRLLRQLTLEEPLAVCCVLHDLNLAALYADRILLLAQGELVACGTPEEVLNAETLTRWYQADLGISRHPESALPQIYLRQ

>Mes669

MKKIVFVLTLMLFSFGTLGQETASGQVGDVSSSTIATEVSEAECGTQSATTQGENDWDWCCELCCNPACFGC

>Mes670

MTTLRKLPIALAVAAGVLSTQAMAVDFHGYARSGIGWTGSGGEQQCFKTTGAQSKYRLGNECETYAELKLGQELWKEGDKSFYLDTNVAYSVSQRDDWESTDPAFREANVQGKNLIESLPGSTMWAGKRFYQRHDVHMIDFYYWDISGPGAGLEAIDLGFGKLSVAATRNSEAGGSSAWINNQRKDADKTINDVYDIRLAGLETNPGGSLEFGVDYGRANTQDDYSLAPNASKDGVLLTAEHTQSMMGGFNKFVVQYATDSMTSWNSGHSQGTSVNNNGHMLRVIDHGAINLAEKWDMMYVALYQDTDWDNNNGTTWYSVGVRPMYKWTPIMSTLLEAGYDNVKSQRTGDRNGQYKLTLAQQWQAGDSIWSRPAIRVFATYANWDEKWGYNNVDKSPDNGLAQNGTIGTDSRGKSNEVTFGAQFEAWW

>Mes671

MNMKKFVKKPLAIAVLMLASGGMVNMVHAEPTVINSKDISATKTVKEGGSFSVEFKATENEIVSGKLDADTPAFHLVMSDSGEHKGWNVRPTGASEGGQMVSADGTRVDLHTNELSWDNDHWWIDDGSERVEATFFLAAGDEVKAGEYQFTGRVEEYVE

>Mes673

MIREERLLKVLRAPHVSEKASAAMEKNNTIVLKVAKDATKAEIKAAVQKLFEVEVEDVNTLLVKGKSKRHGQRVGRRSDWKKAYVTLKEGQNLDFIGGAE

>Mes674

MLIKLLTKVFGSRNDRTLRRMRKVVDLINRMEPEVEKLTNEELRAKTDEFRERLANGAVLETLIPEAFAVVREASKRVFGMRHFDVQLLGGMVLNERCIAEMRTGEGKTLTATLPAYLNALSGRGVHVVTVNDYLAQRDAENNRPLFEFLGLSVGINLPNMPAPAKRAAYAADITYGTNNEFGFDYLRDNMAFSPEERVQRKLHYALVDEVDSILIDEARTPLIISGPAEDSSEMYIRVNKLIPKLIRQEKEDSDTFQGEGHFSVDEKSRQVHLTERGLIKIEEMLVEAGIMEEGESLYSPANIMLMHHVTAALRAHVLFTRDVDYIVKDGEVIIVDEHTGRTMQGRRWSDGLHQAVEAKEGVEIQNENQTLASITFQNYFRLYEKLAGMTGTADTEAFEFSSIYKLDTIVVPTNRPMIRKDLADLVYMTEQEKIGAIIEDIRERTANGQPVLVGTISIEKSEVVSAELTKAGIEHKVLNAKFHAMEAEIVSQAGQPGAVTIATNMAGRGTDIVLGGSWQSEIALLENPTEDQIAAIKAAWQIRHDAVLASGGLHIIGTERHESRRIDNQLRGRAGRQGDAGSSRFYLSMEDALMRIFASDRVSGMMRKLGMKPGEAIEHPWVTKAIANAQRKVESRNFDIRKQLLEYDDVASDQRRAIYSQRNELLDVADVSETINSIREDVFKTVIDSYIPTQSLEEMWDVEGLEQRLKNDFDLDMPIAQWLEDEPQLHEETLRERILQLAIADYQRKEEVVGFDMMRNFEKGVMLQTLDSLWKEHLAAMDYLRQGIHLRGYAQKDPKQEYKRESFAMFAAMLESLKYEVISVLSKVQVRMPEEVEALEVQRREEAERLAKQQQLSHESDNSALMSQEEANVAASLERKVGRNDPCPCGSGKKYKQCHGRLQ

>Mes675

MKKWLCAASLGLALAASASVQAAKIAIVNVSRIFQQLPESETVAKQLENEFKGRATELQGMESDLQTKMQKLQRDGSTMKASDRTKLENDVMKQRETFSTKAQAFEQDNRRRQMEERNKILSRIQDAVKSVASKGGYDVVIDANAVAYADPSKDITADVLKQVK

>Mes677

MSWIEPIISHFCQDLGVPTSSPLSPLIQLEMAQSGTLQLEQHGATLTLWLARSLAWHQCEDAMVKALTLTAAQKSGALPLRAGWLGENQLVLFVSLDERSLTLPLLHQAFEQLLRLQQEVLAP

>Mes678

MQTTFTELMQQLFLKLGLNHQVNENDVYTFEVDGHIQVLIACYHQQWVQLFSELGADLPTNDNLFGEHWPAHVQGRLDGKSILWSQQSLVGLDIDEMQAWLERFIDDIEQRKEPQNTKFQPNSTSPILFI

>Mes679

MSTKTNSTKATSEKTDSLKTNRGTKSSAGYSDQNIPLGGCILADTPITFNENKPVTKVKVRNTGDRPIQVGSHFHFFEANRALEFDRAAAYGKRLNISSTTAIRFEPGDETEVPLIPFGGKQTLYGFNNLVDGWTGEGVVPNSERPDKLEAIRRAAERGFKSSK

>Mes680

MASLEIIKLEWATPIFKVVEHSQDGLYILLQGQISWQNSSQTYDLDEGNMLFLRRGSYAVRCGTKEPCQLLWIPLPGSFLSTFLHRFGSLLSEIRRDNATPKPLLIFNISPILSQSIQNLCAILERSDFPSVLTQLRIEELLLLLAFSSQGALFLSALRHLGNRPEERLQKFMEENYLQGWKLSKFAREFGMGLTTFKELFGTVYGISPRAWISERRILYAHQLLLNGKMSIVDIAMEAGFSSQSYFTQSYRRRFGCTPSQARLTKIATTG

>Mes681

MTKDFKISVSAALISALFSSPYAFADDYDGIPNLTAVQISPNADPALGLEYPVRPPVPGAGGLNASAKGIHSIAIGATAEAAKGAAVAVGAGSIATGVNSVAIGPLSKALGDSAVTYGAASTAQKDGVAIGARASTSDTGVAVGFNSKADAKNSVAIGHSSHVAANHGYSIAIGDRSKTDRENSVSIGHESLNRQLTHLAAGTKDTDAVNVAQLKKEIEKTQENTNKRSAELLANANAYADNKSSSVLGIANNYTDSKSAETLENARKEAFAQSKDVLNMAKAHSNSVARTTLETAEEHANSVARTTLETAEEHANKKSAEALASANVYADSKSSHTLKTANSYTDVTVSNSTKKAIRESNQYTDHKFRQLDNRLDKLDTRVDKGLASSAALNSLFQPYGVGKVNFTAGVGGYRSSQALAIGSGYRVNENVALKAGVAYAGSSDVMYNASFNIEW

>Mes683

MYSFEQAITQLFQQLSLSIPDTIEPVIGVKVGEFACHITEHPVGQILMFTLPSLDNNNEKETLLSHNIFSQDILKPILSWDEVGGHPVLWNRQPLNNLDNNSLYTQLEMLVQGAERLQTSSLISPPRSFS

>Mes685

MKKNMKLIAITAVLSSVLVLSGCGAMSTAIKKRNLEVKTQMSETIWLEPSSQKTVYLQIKNTSDKNMLGLAPKITKAVQDKGYTVTSSPEDAHYWIQANVLKADKMDLREAEGFLSQGYQGAALGAALGAGITGYNSNSAGASLGVGLAAGLVGMVADAMVEDINYTMVTDVQISEKTDTPLQTDNVAALKQGTSGYKVQTSTQTGNKHQYQTRVVSSANKVNLKFEEAQPVLEDQLAKSIANIL

>Mes686

MKISSFISTSLPLPASVSGSSSVGEMSGRSVSQQKSDQYANNLAGRTESPQGSSLASRIIERLSSMAHSVIGFIQRMFSEGSHKPVVTPALTPAQMPSPTSFSDSIKQLAAETLPKYMQQLSSLDAETLQKNHDQFATGSGPLRGSITQCQGLMQFCGGELQAEASAILNTPVCGIPFSQWGTVGGAASAYVASGVDLTQAANEIKGLGQQMQQLLSLM

>Mes687

MNLSLSDLHRQVSRLVQQESGDCTGKLRGNVAANKETTFQGLTIASGARESEKVFAQTVLSHVANIVLTQEDTAKLLQSTVKHNLNNYELRSVGNGNSVLVSLRSDQMTLQDAKVLLEAALRQESGARGHVSSHSHSVLHAPGTPVREGLRSHLDPRTPPLPPRERPHTSGHHGAGEARATAPSTVSPYGPEARAELSSRLTTLRNTLAPATNDPRYLQACGGEKLNRFRDIQCCRQTAVRADLNANYIQVGNTRTIACQYPLQSQLESHFRMLAENRTPVLAVLASSSEIANQRFGMPDYFRQSGTYGSITVESKMTQQVGLGDGIMADMYTLTIREAGQKTISVPVVHVGNWPDQTAVSSEVTKALASLVDQTAETKRNMYESKGSSAVADDSKLRPVIHCRAGVGRTAQLIGAMCMNDSRNSQLSVEDMVSQMRVQRNGIMVQKDEQLDVLIKLAEGQGRPLLNS

>Mes688

MTTLHNLSYGNTPLRNEHPEIASSQIVNQTLGQFRGESVQIVSGTLQSIADMAEEVTFVFSERKELSLDKRKLSDSQARVSDVEEQVNQYLSKVPELKQKQNVSELLSLLSNSPNISLSQLKAYLEGKSEEPSEQFKMLCGLRDALKGRPELAHLLHLVEQALVSMVEEQEEAIVLGARITPEAYRESQSGVNPLQPLRDTYRDAVMGYQGINAIWSDLQKRFPNGDIDSVILFLQKALSADLQSQQSGSEREKLEIVISDLQKLKEFRSVSDQVKGFWQLFSEGITNGLRPF

>Mes689

MSQITTKHITVLFRRWMAIICCLIIKIAYLAY

>Mes690

MQNLLKNLATSLGRKPFVADKQGVYRLTIDKHLVMLTPHGSELVLRTPIDAPMLREGNNVNVTLLRSLMQQALAWAKRYPQTLVLDDCGQLVLEARLRLQELDTHGLQEVINKQLALLEHLIPQLTPFSVASRVGWN

>Mes691

MSWVCRFYQGKHRGVEVELPHGRCVFGSDPLQSDIVLSDSEIAPVHLVLMVDEEGIRLTDSAEPLLQEGLPVPLGTLLRAGTCLEVGFLLWTFVAVGQPLPETLQVPTQRKEPTDRLPRSRLGVGLGVLSLLLLLTFLGMLGHGLWREYNQDGQLVEQEVRRLLATAAYKDVVLTSPKEGEPWLLTGYIQDNHARLSLQNFLESHGIPFRLELRSMEELRQGAEFILQRLGYHGIEVSLAPQAGWLQLNGEVSEEIQKQKIDSLLQAEVPGLLGVENKVRIAGNQRKRLDALLEQFGLDSDFTVNVKGELIELRGQVNDEKLSSFNQLQQTFRQEFGNRPKLELVNVGGQPQHDELNFEVQAISLGKVPYVVLDNHQRYPEGAILNNGVRILAIRRDAVIVSKGKREFVIQLNGGKPR

>Mes692

MTQLEEQLHNVETVRSITMQLEMALAKLKKDMMRGGDAKQYQVWQSESKAIESAIAIIHYVAGGLK

>Mes693

MSNFSGFTKGNDIADLDAVAQTLKKPADDANKAVNDSIAALKDTPDNPALLADLQHSINKWSVIYNISSTIVRSMKDLMQGILQKFP

>Mes694

MKYKLNVLLAEIALIGTGNHCHEEANCIAEWLHLKGEEEAVQLIQLSSLMNRGDYASALQQGNKSTYPDLEPWLALCEYRLGLGNALESRLNRLATSQDPRIQTFVNGMKEQLKT

>Mes695

MTVTLNRGSITSLMSSSQAVSTLQPAASELKTQLEHKLKSESAEKTREVLWQQYYASNPPDHAVLEVLATPVREALLARFGQHQGPVVPAIDLPELRSVLQQFDSFGKRREAILLQVLEGIKPNESQVGLPYLSELINKELMILLPYNSIVDSLLHNSHQIDMET

>Mes696

MPNIEIAQADEVIITTLEELGPVEPTTEQIMRFDAAMSEDTQGLGHSLLKEVSDIQKTFKTAKSDLHTKLAVSVDNPNDLMLMQWSLIRITIQEELIAKTAGRMSQNVETLSKGG

>Mes697

MKVKTSLSTLILILFLTGCKVDLYTGISQKEGNEMLALLRQEGLSADKEPDKDGKIKLLVEESDVAQAIDILKRKGYPHESFSTLQDVFPKDGLISSPIEELARLNYAKAQEISRTLSEIDGVLVARVHVVLPEEQNNKGKKGVAASASVFIKHAADIQFDTYIPQIKQLVNNSIEGLAYDRISVILVPSVDVRQSSHLPRNTSILSIQVSEESKGRLIGLLSLLILLLPVTNLAQYFWLQRKK

>Mes698

MMENYITSFQLRFCPAAYLHLEQLPSLWRSILPYLPQWRDSAHLNAALLDEFSLDTDYEEPHGLGALPLQPQSQLELLLCRLGLVLHGEAIRRCVLASPLQQLLTLVNQETLRQIIVQHELLIGPWPTNWQRPLPTEIESRTMIQSGLAFWLAAMEPQPQAWCKRLSLRLPLATPSEPWLVAESQRPLAQTLCHKLVKQVMPTCSHLFK

>Mes699
[truncated: 411,709 more chars]
